# Umpolung Synthesis of Pyridyl Ethers by Bi^V^‐Mediated O‐Arylation of Pyridones

**DOI:** 10.1002/anie.202212873

**Published:** 2022-11-17

**Authors:** Katie Ruffell, Liliana C. Gallegos, Kenneth B. Ling, Robert S. Paton, Liam T. Ball

**Affiliations:** ^1^ School of Chemistry University of Nottingham Nottingham NG7 2RD UK; ^2^ Department of Chemistry Colorado State University Fort Collins CO 80523 USA; ^3^ Syngenta Jealott's Hill International Research Centre Bracknell RG42 6EY UK

**Keywords:** Ambident Nucleophile, Arylation, Bismuth, Density-Functional Calculations, Synthetic Methods

## Abstract

We report that O‐selective arylation of 2‐ and 4‐pyridones with arylboronic acids is affected by a modular, bismacycle‐based system. The utility of this umpolung approach to pyridyl ethers, which is complementary to conventional methods based on S_N_Ar or cross‐coupling, is demonstrated through the concise synthesis of Ki6783 and picolinafen, and the formal synthesis of cabozantib and golvatinib. Computational investigations reveal that arylation proceeds in a concerted fashion via a 5‐membered transition state. The kinetically‐controlled regioselectivity for O‐arylation—which is reversed relative to previous Bi^V^‐mediated pyridone arylations—is attributed primarily to the geometric constraints imposed by the bismacyclic scaffold.

## Graphical abstract

We demonstrate that sterically‐ and electronically‐diverse pyridyl ethers can be accessed via Bi^V^‐mediated C−O coupling of 2‐ or 4‐pyridones with arylboronic acids. Use of a bismacyclic reagent not only confers modularity on our methodology, but also results in highly regioselective O‐arylation that is unprecedented for Bi^V^.

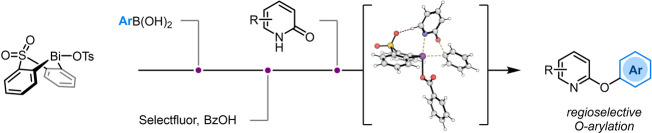

## Introduction

**Scheme 1:**
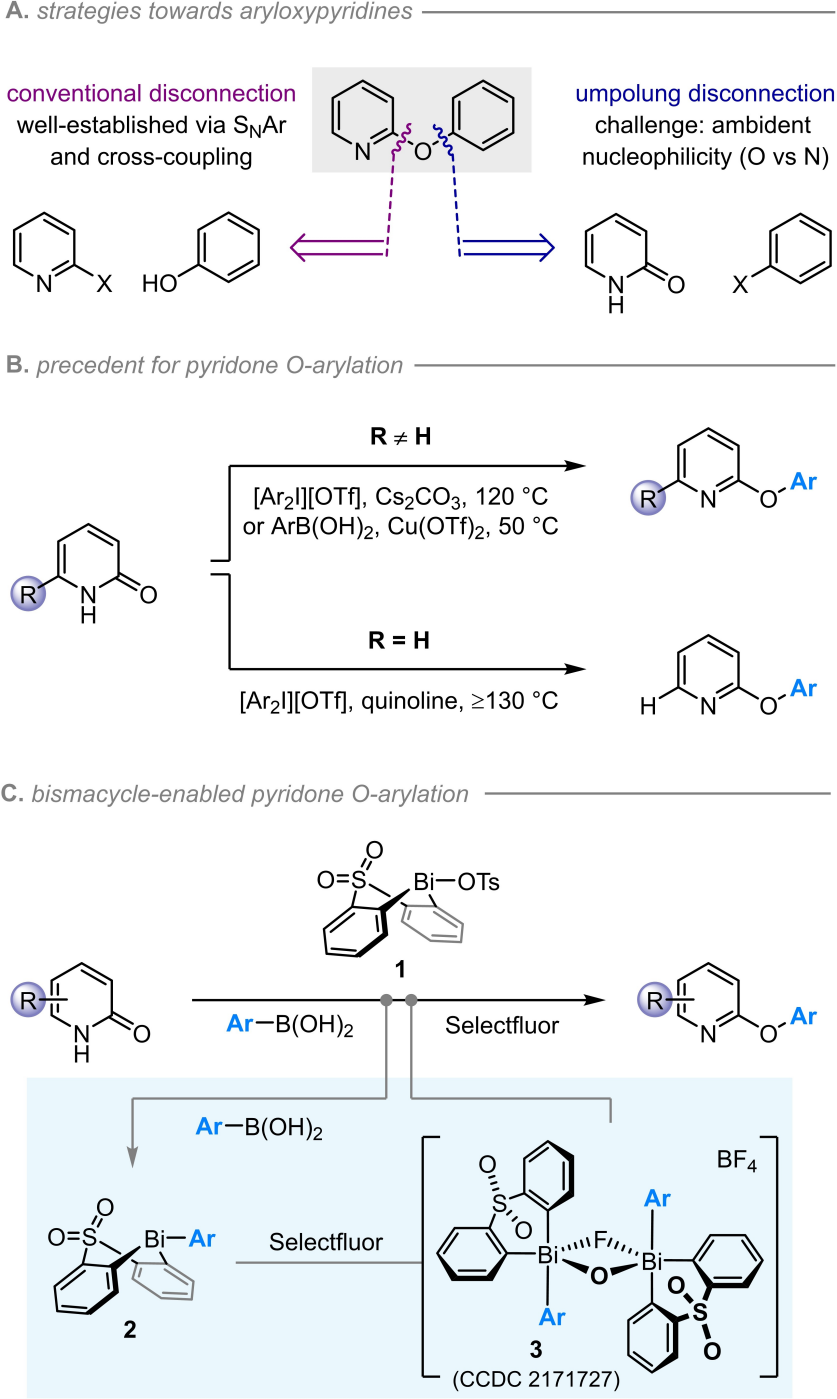
a) Complementary disconnections of the 2‐aryloxypyridine motif. b) Limited methods exist for the O‐selective arylation of pyridones.[^12^, ^13^, ^14^] c) Modular, O‐selective Bi^V^‐mediated pyridone arylation.

The 2‐ and 4‐aryloxypyridine motifs are common to numerous drugs and agrochemicals, including six tyrosine kinase inhibitors approved in the last decade.^[1]^ Aryloxypyridines are conventionally prepared via S_N_Ar (Scheme 1A, left),^[2]^ a strategy which—although extremely well‐established—typically requires forcing conditions and is inherently limited by the innate preference for an electron‐poor pyridine and an electron‐rich phenol partner.[^3^, ^4^] The use of cross‐coupling[^5^, ^6^, ^7^] largely overcomes these electronic preferences, thereby allowing the installation of electron‐poor phenols that are valued for their increased metabolic stability.^[8]^ However, C−O couplings remain less general than analogous C−C and C−N couplings, and the regioselectivity of both S_N_Ar and cross‐coupling can be unpredictable and/or uncontrollable for polyhalopyridines.[^9^, ^10^, ^11^]

The alternative disconnection requires O‐arylation of a pyridone (Scheme 1A, right), an umpolung strategy that potentially places complementary electronic demands on the reacting partners. However, this approach is rendered challenging by competing O‐ and N‐arylation, which results from the ambident nucleophilicity of pyridones.

While the factors affecting the regioselectivity of pyridone *alkylation* are well studied[^15^, ^16^] and increasingly well understood,[^17^, ^18^] the *arylation* of pyridones is less highly developed. The N‐arylation of both 2‐ and 4‐pyridones can be achieved selectively^[19]^ using electrophilic arylating agents based on I^III^ [^14^, ^20^, ^21^] or Bi^V^,[^22^, ^23^] by copper‐catalysed couplings with arylhalides,[^24^, ^25^, ^26^] arylboronic acids^[27]^ or triarylbismuth(III) reagents,[^28^, ^29^] or by S_N_Ar with an appropriately activated aryl halide.[^30^, ^31^] In contrast, achieving selectivity for O‐arylation is more challenging (Scheme 1B). For example, diaryliodonium salts^[12]^ or Chan–Lam couplings^[13]^ typically afford N‐arylated 2‐pyridones as the major product, with O‐arylation favored only when the 6‐position of the pyridone is substituted. Indeed, there is currently only one method for the O‐arylation of 2‐pyridones that do not feature a 6‐substituent, but this requires high temperatures (130–140 °C) and has been demonstrated for the installation of only one *ortho*‐substituted, and no electron‐rich, aryl moieties.^[14]^ The O‐selective arylation of 4‐pyridones is even less well developed, with just a single example reported in each of three papers.[^14^, ^32^, ^33^] There thus remains an unmet need for a convenient, mild and general method for the O‐selective arylation of 2‐ and 4‐pyridones, which would provide a powerful, umpoled complement to well‐established S_N_Ar and cross‐coupling strategies.

Here we report that a sulfone‐bridged bismacycle enables the O‐selective arylation of 2‐ and 4‐pyridones with arylboronic acids (Scheme 1C). High yields and complete O‐selectivity are observed across a broad range of electronically and sterically diverse partners, with none of the apparent limitations or substrate‐dependence associated with existing approaches. The use of a recoverable bismacyclic scaffold confers both modularity and atom‐economy on our strategy, and—as demonstrated computationally—is responsible for the reversal in regioselectivity relative to the previously‐reported Bi^V^‐mediated pyridone arylations.[^22^, ^23^]

## Results and Discussion

We sought to develop a modular and convenient strategy in which a reactive Bi^V^ arylating agent is prepared from bench‐stable precursor **1** via sequential B‐to‐Bi transmetallation and oxidation (Scheme 1C). Having previously established the key transmetallation step,[^34^, ^35^] our initial studies focused on the oxidation/arylation procedure. Disappointingly, conditions optimized for the arylation of phenols^[34]^ and acidic diones^[35]^ proved unsuitable for the arylation of pyridones: <5 % of the desired aryloxypyridine was formed at room temperature using either *m*CPBA or Selectfluor as the oxidant (Figure S1). Given that both *m*CPBA and Selectfluor rapidly effect the oxidation of arylbismacycle **2**, this observation suggests that the issue lies in either (1) formation of a key Bi^V^‐pyridonate intermediate, or (2) the subsequent product‐forming arylation process. Due to the greater thermal stability of the Bi^V^ species formed with Selectfluor—the O,F‐bridged dimer **3** (Scheme 1C), which was previously isolated and characterized crystallographically^[35]^—we further explored the use of this fluoronium reagent in combination with isolated bismacycles bearing both *ortho*‐substituted and *ortho*‐unsubstituted aryl moieties (Table 1).

**Table 1:** Optimization of the Bi^V^‐mediated O‐arylation of 2‐pyridone from isolated arylbismacycle **2**.

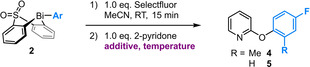				
Entry	Additive (1.0 equiv)	*T* [°C]	Yield **4** [%]	Yield **5** [%]
1	None	25	<5	<5
2	None	60	61	16
3	None	80	84	36
4^[b]^	None	100	85	52
5	DBU, BTMG, K_2_CO_3_, KO^*t*^Bu, or NaOH	80	4–62	2–25
6	NaOBz	80	89	56
7	BzOH	80	94	76
8	BzOH and NaOBz	80	92	65
9	AcOH, TFA, PivOH, 4‐MeO‐C_6_H_4_CO_2_H, or 4‐F_3_C‐C_6_H_4_CO_2_H	80	90–93	55–72
10	1.5 or 2.0 equiv BzOH	80	93	75 or 76

[a] Reactions performed on a 0.02 mmol scale. Yields determined by ^19^F NMR spectroscopy vs internal standard (4,4′‐bis(trifluoromethyl)biphenyl). [b] Reaction performed in a sealed tube; reaction temperature refers to that of the heating block. BTMG=2‐*tert*‐butyl‐1,1,3,3‐tetramethylguanidine; Piv=pivaloyl.

While the yield of aryloxypyridines **4** and **5** increased with reaction temperature (Table 1, entries 1–4), the addition of base was detrimental (entry 5; see also Table S2). The addition of NaOBz resulted in a substantial increase in the yield of *ortho*‐unsubstituted **5** (entry 3 vs entry 6), but—in contrast to our previous work^[35]^—BzOH alone proved more effective than either NaOBz or a combination of NaOBz and BzOH (entries 6–8).

Further optimization revealed that the yield was not improved by using acid additives other than BzOH (entry 9; see also Table S3) or by altering the stoichiometry of acid (entry 10; see also Table S4). Variation of the bismacycle scaffold—a strategy used to great effect by Cornella in C_Ar_‐OTf couplings^[36]^—indicated that electron‐withdrawing substituents afforded a minor benefit in the synthesis of **5** but not **4** (Table S6). Unfortunately, this benefit was not general across different substrate combinations (Table S7), and the preferred arylation conditions are thus represented in Table 1, entry 7.

Combining the optimized oxidation/arylation step with B‐to‐Bi transmetallation indicated that the yield was reduced by residual sodium tosylate and boric acid from the transmetallation, partly through competitive formation of aryl tosylate side‐products (see Figure S2 and Table S5).[^37^, ^38^] A basic wash was therefore incorporated between the transmetallation and oxidation/arylation steps, allowing both halves of the process to be telescoped into a single operation. Importantly, addition of TsOH during final work‐up enables the direct recovery of bismacycle tosylate **1** by filtration (80 % yield, >95 % purity), with no detriment to the yield of the arylated pyridone (see Supporting Information, Section S5).

Notably, N‐arylation was not observed under any of the conditions investigated. This stands in contrast to the previous reports that simple (non‐bismacyclic) triarylbismuth(V) reagents give N‐selectivity, with O‐arylation observed as a side‐reaction in only a limited number of specific examples.[^22^, ^23^] The origin of this reversal in regioselectivity is discussed in detail later.

**Scheme 2:**
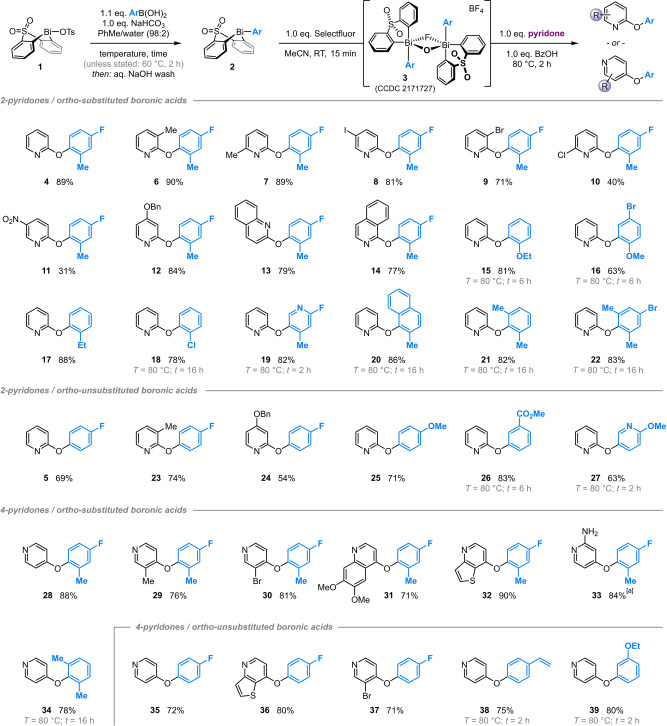
Scope of the Bi^V^‐mediated O‐arylation of 2‐ and 4‐pyridones. Reactions performed on a 0.80 mmol scale. Yields refer to material isolated following purification. [a] Reaction performed using Boc‐protected 2‐amino‐4‐pyridone; 5 equiv TsOH⋅H_2_O added prior to work‐up (see Supporting Information, Section S6(iii), for details).

The scope of our methodology extends to diverse 2‐ and 4‐pyridones (Scheme 2), with high yields typically obtained for pyridones substituted proximal (2‐pyridones: **6**, **9**, **14**, **23**; 4‐pyridones: **29**–**32**, **36**–**37**) or distal (2‐pyridones: **7**, **8**, **12**, **13**, **24**; 4‐pyridones: **33**) to the site of arylation. Complete O‐selectivity is observed across all substrates, with no requirement for the 6‐substituent that confers O‐selectivity on other 2‐pyridone arylation methods.[^12^, ^13^] The connectivity of the products was apparent from characteristic spectroscopic features (NMR, IR), and by comparison to literature data for both the O‐ and N‐arylated isomers (NMR, IR, m.p.).

While only modest yields were obtained for 6‐chloro‐2‐pyridone (**10**) and 5‐nitro‐2‐pyridone (**11**), these motifs are readily accessible via facile S_N_Ar of the corresponding halopyridine. Crucially, however, the Bi‐mediated arylation methodology is highly effective for electron‐rich pyridyl partners (**12**, **24**, **33**), which are traditionally challenging to engage in S_N_Ar. For example, the 4‐alkoxy substructure of **12** and **24** is accessed in only modest yields at >110 °C via S_N_Ar^[39]^ or at 130 °C under Cu catalysis,^[40]^ whereas aminopyridines analogous to **33** are accessible via S_N_Ar only at very high temperatures (140–220 °C).[^41^, ^42^, ^43^] These results highlight the valuable complementarity that results from the opposing electronic demands of our method and S_N_Ar.

The scope with respect to the boronic acid arylating agent is similarly broad. Notably, aryl moieties featuring both electron‐donating (**15**–**17**) and withdrawing (**18**) *ortho* substituents are tolerated, as is di‐*ortho*‐substitution (**20**–**22**, **34**). This compares favorably to the previously reported method for O‐arylation of pyridones with boronic acids, which is not only limited to 6‐substituted pyridones, but which also does not tolerate *ortho*‐substitution on the boronic acid component.^[13]^ The ability to transfer electron‐poor aryl moieties (**18**, **19**, **26**, **27**, **39**), including from pyridylboronic acids (**19**, **27**), further illustrates the complementarity between our pyridone arylation approach and S_N_Ar.

**Scheme 3:**
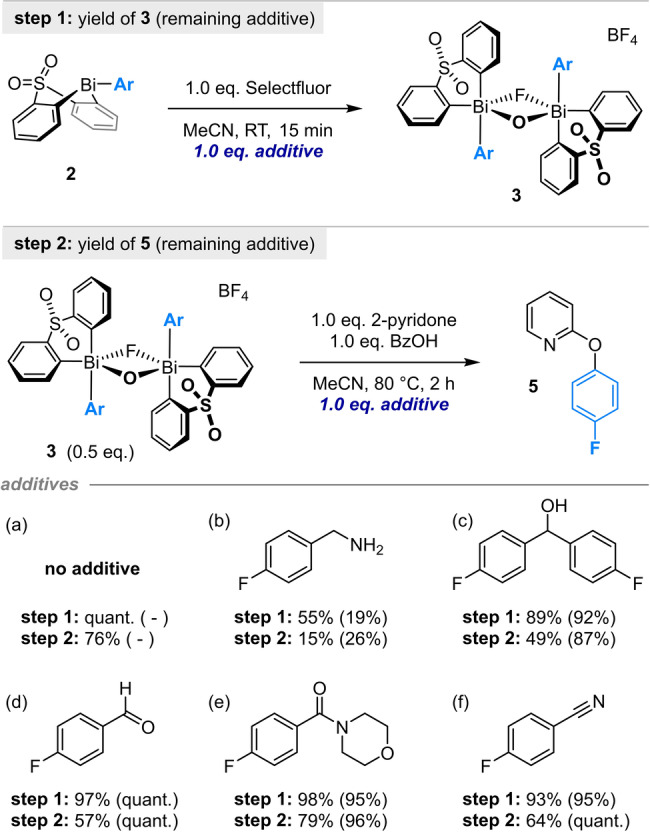
Robustness screen of the oxidation and arylation steps. Yields determined by ^19^F NMR spectroscopic analysis vs internal standard.

**Scheme 4:**
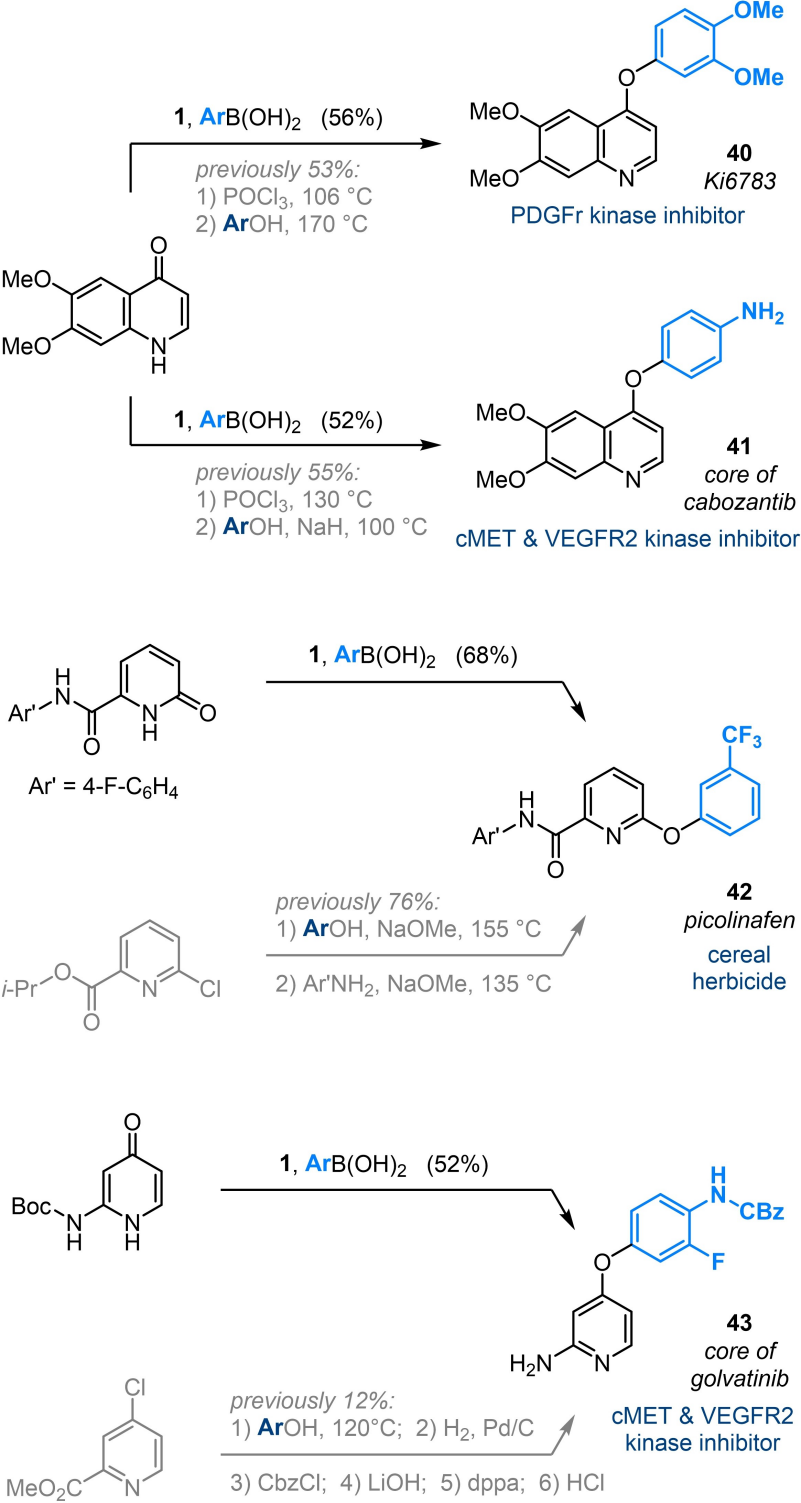
Application of Bi^V^‐mediated arylation to the synthesis of Active Ingredients, or their key intermediates, and comparison to literature routes.[^47^, ^48^, ^49^, ^50^, ^51^, ^52^] See Scheme 2 for detailed conditions of Bi^V^‐mediated arylation; yields for literature routes are calculated over all steps from the indicated starting material. dppa=diphenylphosphoryl azide.

Synthetically valuable substituents are tolerated on both components, including halogens (**8**, **9**, **16**, **18**, **22**, **30**), the 2‐fluoropyridyl moiety (**19**), esters (**26**), alkenes (**38**), and the Cbz protecting group (**43**, Scheme 4). The compatibility of our methodology with arylhalides and 2‐halopyridines demonstrates its complementarity to the cross‐coupling and S_N_Ar strategies typically employed in the synthesis of aryloxy pyridines. Tolerance towards additional, common functional groups was probed using a Glorius‐type robustness screen (Scheme 3).[^44^, ^45^]

By separating the oxidation and arylation processes, we sought to replicate the introduction of functional groups as part of (a) the arylboronic acid, which is exposed directly to Selectfluor, and (b) the pyridone, which is exposed only to Bi^V^ dimer **3**. As anticipated,[^35^, ^46^] a primary amine is not tolerated in the oxidation step (Scheme 3, entry b), and is also incompatible with the subsequent arylation. However, in contrast to our recently‐reported methodology for Bi^V^‐mediated dione arylation,^[35]^ a secondary benzylic alcohol is tolerated in the oxidation (entry c); this difference in reactivity may reflect the distinct speciation of the Bi^V^ centres being formed in each case. Benzaldehydes, amides and nitriles are tolerated in both steps (entries d–f).

The utility of our umpolung approach to aryloxypyridines is further demonstrated through the concise synthesis of Active Ingredients or their key intermediates. As illustrated in Scheme 4, bismuth‐mediated O‐arylation achieves comparable or better yields in fewer steps and under milder conditions than have been reported previously. For example, preparation of Ki6783 **40**,[^47^, ^48^] and the core of cabozantib **41**,[^49^, ^50^] via O‐arylation of a common quinolone negates the need for sequential chlorodeoxygenation and high‐temperature S_N_Ar. Similarly, synthesis of the herbicide picolinafen **42** using our methodology avoids S_N_Ar etherification and amidation under forcing conditions,^[51]^ whereas our synthesis of the core of golvatinib **43** contrasts the low‐yielding, 6‐step synthesis reported previously.^[52]^

**Scheme 5:**
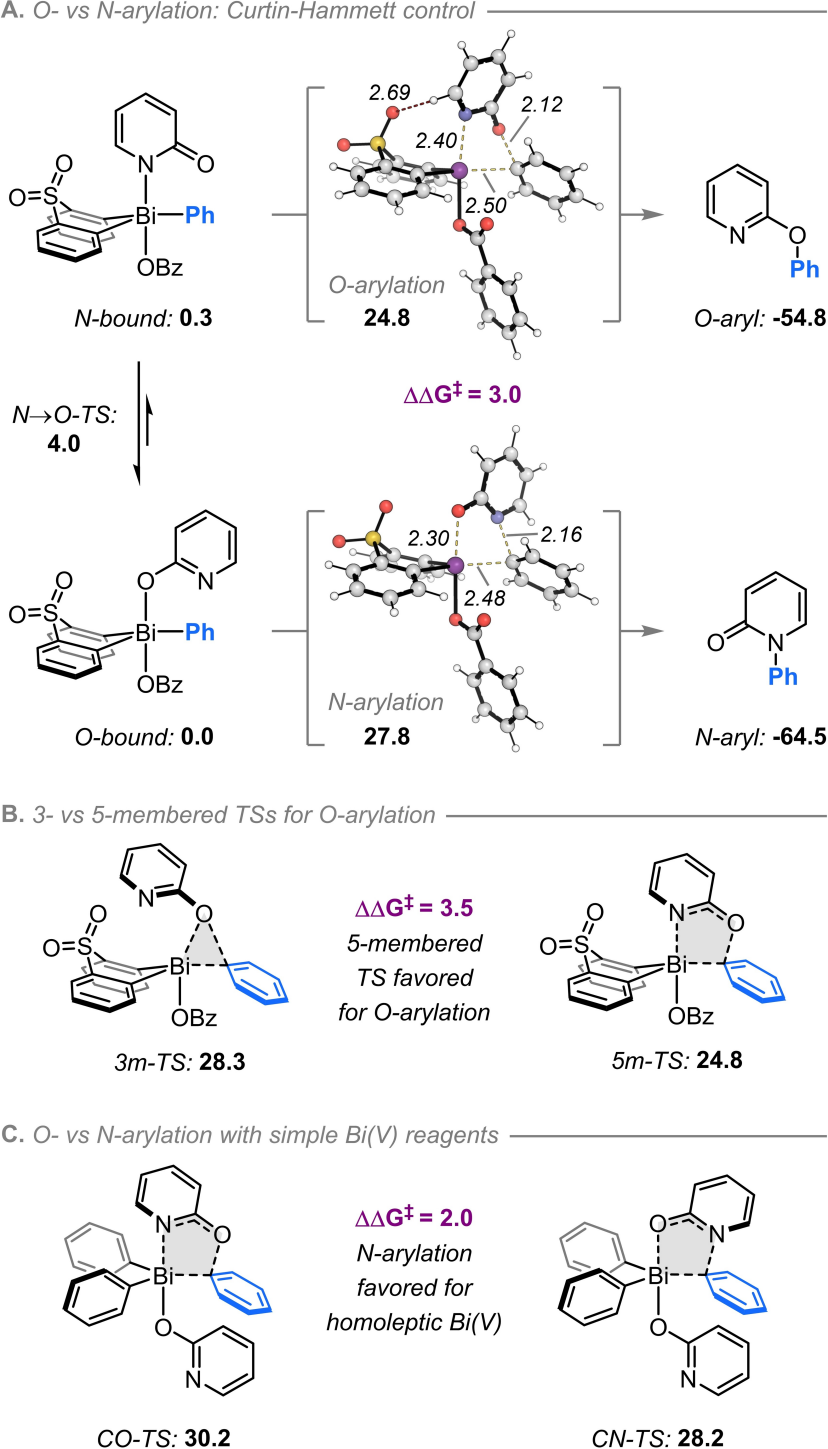
Computational investigation of ligand coupling pathways from Bi^V^‐2‐pyridonate intermediates. a) O‐ vs N‐arylation pathways for bismacyclic reagents. b) Comparison of 3‐ and 5‐membered O‐arylation transition states. c) O‐ vs N‐arylation pathways for non‐bismacyclic reagents. Calculations performed using ωB97XD/def2‐QZVPP//ωB97XD/6‐31+G(d,p) and def2SVP (Bi); all energies are quoted in kcal mol^−1^, and all bond lengths in Å.

The regioselectivity observed for pyridone O‐arylation with the present bismacyclic system stands in stark contrast to the N‐selectivity reported previously using simple triarlybismuth(V) reagents.[^22^, ^23^] In order to elucidate the origin of these differences, DFT calculations (ωB97XD/def2 QZVPP//ωB97XD/6‐31+G(d,p) and def2SVP (Bi)) with the SMD‐solvation model applied during all calculations, were used to investigate the key features of each arylation pathway (Scheme 5; see Supporting Information Section S8 for computational details and a full discussion of alternative intermediates and transition states).^[49]^

For the bismacyclic system, the N‐ and O‐bound Bi^V^‐pyridonate isomers both adopt trigonal bipyramidal geometries in which the pyridonate and benzoate ligands are located apically, and the aryl substituents are equatorial (Scheme 5A). Arylation proceeds preferentially via cyclic 5‐membered transition structures (TSs) such that the N‐bound Bi^V^‐pyridonate leads to O‐arylation and *vice versa*. In both TSs, cleavage of the Bi−C_*ipso*_ bond and formation of the new C_*ipso*_−O or C_*ipso*_−N bond occur concertedly. While there is a slight preference for the pyridonate intermediate to be O‐bound (0.3 kcal mol^−1^), the barrier for conversion between the isomers is small (4.0 kcal mol^−1^) relative to the barriers for arylation. Regioselectivity is therefore determined in the irreversible arylation step in which—consistent with experiment—O‐arylation is kinetically favored (by 3.0 kcal mol^−1^).

In addition to 5‐membered TSs, 3‐membered cyclic TSs were also located (Scheme 5B). While this alternative pathway rationalizes the ability of 4‐pyridones to undergo arylation, it is less favorable than the 5‐membered TS for 2‐pyridone (by 3.5 kcal mol^−1^).

The analogous arylation TSs for a simple triphenylbismuth‐derived reagent were also calculated (Scheme 5c). While 5‐membered TSs are again favored, N‐arylation is now kinetically favored over O‐arylation (by 2 kcal mol^−1^), consistent with Mukaiyama's observations.[^22^, ^23^] Relative to this acyclic system, the bismacyclic scaffold preferentially stabilizes the O‐arylation TS by several kcal mol^−1^. Favorable non‐covalent interactions between the sulfone bridge and C6‐H of the pyridine are present for O‐arylation, whereas for N‐arylation the carbonyl oxygen is oriented unfavorably towards the SO_2_ group (Scheme 5A). The geometry of the bismacycle is also a major factor; for example, computationally deleting the sulfone group but retaining the geometry of the resulting BiPh_2_ unit is still predicted to favor O‐arylation (see Supporting Information, Section S8(ix)).

## Conclusion

In conclusion, we have developed a convenient, telescoped procedure for the Bi^V^‐mediated O‐arylation of 2‐ and 4‐pyridones with arylboronic acids. Complete O‐selectivity is observed across a broad range of substrates; the origin of this regioselectivity, which is reversed relative to previous Bi^V^‐mediated arylations, is attributed primarily to the geometry that the cyclic scaffold imposes at the bismuth center. High atom economy is achieved with respect to both the coupling partners and—following its recovery—the bismacycle tosylate mediator. Due to its tolerance towards valuable carbon‐halogen bonds and electron‐rich pyridyls, this umpoled method represents a powerful complement to conventional syntheses of aryloxypyridines, such as S_N_Ar and cross‐coupling. The utility of our methodology is showcased in the synthesis of four biologically relevant compounds in good yield and fewer steps than was previously possible.

## Experimental Section

Experimental and computational data (including absolute energy values and Cartesian coordinates) that support the findings of this study are available in the Supporting Information of this article.

## Conflict of interest

The authors declare no conflict of interest.

1

## Supporting information

As a service to our authors and readers, this journal provides supporting information supplied by the authors. Such materials are peer reviewed and may be re‐organized for online delivery, but are not copy‐edited or typeset. Technical support issues arising from supporting information (other than missing files) should be addressed to the authors.

Supporting Information

Supporting Information

## Data Availability

The data that support the findings of this study are available in the Supporting Information of this article.
