# Supplementary material for: Umpolung Synthesis of Pyridyl Ethers by BiV‐Mediated O‐Arylation of Pyridones
Source: Angew Chem Int Ed Engl. 2022 Nov 17;61(51):e202212873. doi: 10.1002/anie.202212873 (PMC10099949; doi:10.1002/anie.202212873)

Angewandte Chemie
Eine Zeitschrift der Gesellschaft Deutscher Chemiker

Supporting Information

Umpolung Synthesis of Pyridyl Ethers by Bi^{V}-Mediated O-Arylation of Pyridones

K. Ruffell, L. C. Gallegos, K. B. Ling, R. S. Paton, L. T. Ball**

1. Table of Contents

2. General Information 3

3. Starting Material Synthesis 5

i. Unsubstituted Bismacycles 5

ii. Sulfone Ligands 9

iii. Substituted Bismacycles 14

iv. Substrates 28

4. Optimisation 32

i. Optimisation of Conditions and Additives 32

ii. Bismacycle Ligand Screen 44

5. General Procedure for Pyridone Arylation 48

6. Characterisation Data for Arylation Products 50

i. Confirmation of Regioselectivity for O-Arylation 50

ii. 2-Pyridone Substrates 55

iii. 4-Pyridone Substrates 71

iv. Active Ingredient Targets 79

7. Robustness Screen 83

8. Computational Details 86

i. Computational Methods 86

ii. Absolute Energy Values 87

iii. Molecular Coordinates 87

iv. Bismacycle-based Arylation: O- and N-Arylation Pathways 89

v. More O'Ferrall–Jencks Plot: High Degree of Concert 91

vi. Ph_3BiCl_2-Based Arylation: O- and N-Arylation Pathways 91

vii. Transition State Stability Analysis 92

viii. Bi(V) Intermediates 95

ix. Favorable TS Interactions Determining Selectivity 96

x. Comparing Non-Covalent Interactions (NCI) for Both Systems 97

9. References 99

10. NMR Spectra 101

2. General Information

Procedures employing oxygen- and/or moisture-sensitive materials were performed with anhydrous solvents (*vide infra*) using standard inert-atmosphere techniques (atmosphere of anhydrous dinitrogen). Analytical thin-layer chromatography was performed on precoated aluminium-backed plates (Silica Gel 60 F254; Merck) and visualized using a combination of UV light (254 nm) and aqueous basic potassium permanganate stains. Manual flash column chromatography was performed using Scharlab 60 silica gel (35-70 mesh); automated flash column chromatography was performed on disposable columns pre-packed with 50 μm spherical silica gel using a Büchi C-850 or C-815 equipped with a UV-vis DAD (200-800 nm) and an ELSD.

NMR spectra were recorded at 25 °C on a Bruker Avance 500 or 400 spectrometer (^{1}H, 500 / 400 MHz; ^{13}C{^{1}H}, 125 / 100 MHz; ^{19}F NMR, 471 / 376 MHz). Chemical shifts are reported in ppm; coupling constants, *J*, are reported in Hz and are uncorrected for digitization. The following abbreviations (and their combinations) are used to label the multiplicities: s (singlet), d (doublet), t (triplet), q (quartet), quint (quintet), sept (septet), m (multiplet), br (broad) and app. (apparent). ^{1}H and ^{13}C{1H} chemical shifts are reported relative to tetramethylsilane, and are referenced to the appropriate residual solvent peaks:

- $CDCl_3$: δ_H = 7.26 ppm, δ_C = 77.16 ppm
- CD_3OD: δ_H = 3.31 ppm, δ_C = 49.00 ppm
- CD_3CN: δ_H = 1.94 ppm, δ_C = 118.26 ppm
- DMSO-d_6: δ_H = 2.50 ppm, δ_C = 39.52 ppm

Infrared spectra of neat compounds were recorded over the range 4000-600 cm^{-1} using either a PerkinElmerSpectrum 1000 Series FTIR spectrometer with an ATR diamond cell, or a Bruker Alpha FTIR spectrometer fitted with a Bruker Platinum ATR Quicksnap™ diamond cell. Melting points were measured using Stuart SMP10 or Gallenkamp melting point apparatus in open capillaries.

High resolution electrospray ionization mass spectra (HRMS) were recorded using a Bruker ESITOF MicroTOF II spectrometer.

Reagent grade solvents (Fisher Technical) were employed. THF was dried using an Inert PureSolv Grubbs-type system (alumina columns, argon atmosphere). CH_2Cl_2 was distilled

from 4Å molecular sieved under an atmosphere of anhydrous dinitrogen. Unless stated otherwise, all reagents were used as received from commercial sources.

3. Starting Material Synthesis

i. Unsubstituted Bismacycles

Tris(4-fluorophenyl)bismuthine (S1)

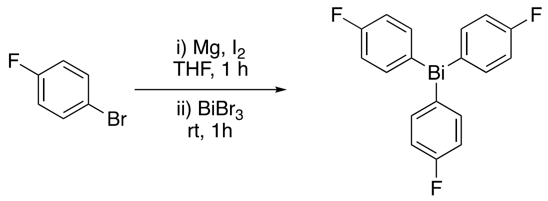

In a flame dried flask under an atmosphere of N_2, magnesium turnings (4.01 g, 165 mmol) and ~3 iodine crystals were heated until a purple vapour developed and stirred for 20 mins. Anhydrous THF (60 mL) was added followed by dropwise addition of 1-bromo-4-fluorobenzene (17.0 mL, 155 mmol) at a rate that maintained reflux. The mixture was then stirred for 1 h, allowing time to cool to room temperature. In a separate flame dried flask under an atmosphere of N_2, a solution of $BiBr_3$ (22.4 g, 50.0 mmol) in anhydrous THF (75 mL) was prepared to which the Grignard reagent was added dropwise. The mixture was stirred for 1 h before quenching with water (100 mL), filtering through a pad of silica gel and extracting with Et_2O (3 × 100 mL). The combined organic portions were dried over $MgSO_4$ and concentrated to dryness. Recrystallisation from hot ethanol afforded the title compound (22.2 g, 44.8 mmol, 90%) as a colourless solid. Characterisation data were consistent with literature values: ^{1}H NMR, ν_{max}.[1]

^{1}H NMR (500 MHz, $CDCl_3$): δ 7.66 (dd, *J* = 8.4, 6.2 Hz, 6H), 7.09 (app. t, *J* = 9.0 Hz, 6H).
^{13}C{^{1}H} NMR (126 MHz, $CDCl_3$): δ 163.0 (d, *J* = 247.3 Hz), 149.6 (br), 139.3 (d, *J* = 7.0 Hz), 118.1 (d, *J* = 19.7 Hz).
^{19}F NMR (471 MHz, $CDCl_3$): δ -112.67 (tt, *J* = 9.4, 6.1 Hz).
ν_{max} (ATR)/cm^{-1}: 3058, 1571, 1482, 1384, 1212, 1159, 1016, 816, 506.
HRMS calcd. for $C_{18}H_{12}BiF_3$+Na^+: 517.0587 [M+Na]$^+$; found (ESI$^+$) 517.0583.
m.p./°C: 96–97.

5,5-Dioxido-10*H*-dibenzo[*b*,*e*][1,4]thiabismin-10-yl 4-methylbenzenesulfonate (1)

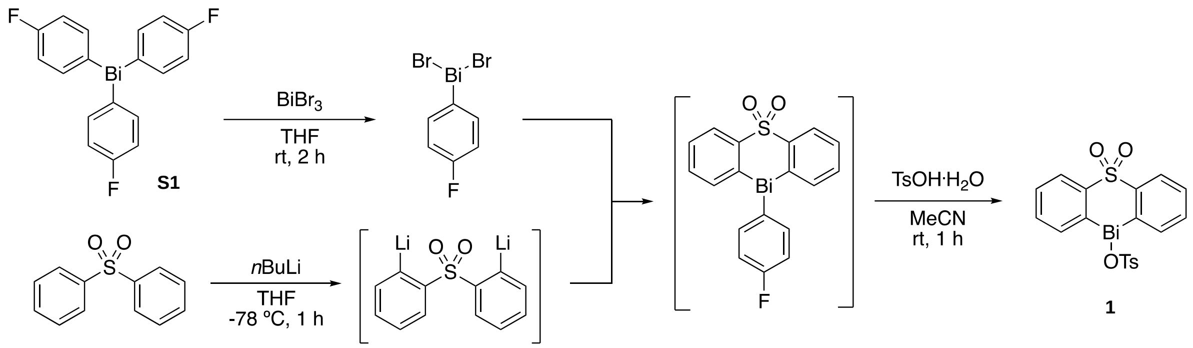

In a flame dried flask under an atmosphere of N_2, a solution of tri(4-fluorophenyl)bismuth **S1** (9.49 g, 19.2 mmol) and $BiBr_3$ (17.2 g, 38.4 mmol) in anhydrous THF (100 mL) was stirred at room temperature for 2 h.

In a flame dried flask under an atmosphere of N_2, *n*-butyllithium (2.5 M in hexanes; 48 mL, 120 mmol) was added dropwise to a solution of diphenylsulfone (13.1 g, 60.0 mmol) in anhydrous THF (120 mL) at -78 °C. The resulting solution was stirred at -78 °C for 1 h.

The bromodiarylbismuth solution was added dropwise *via* cannula to the dilithiodiphenylsulfone mixture and the resulting suspension was allowed to warm to room temperature overnight. The mixture was quenched with MeOH (10 mL) and concentrated to dryness then dissolved in CH_2Cl_2 (100 mL) and filtered through a pad of silica gel. The filtrate was stirred with sat. aq. $NaHCO_3$ solution (100 mL) and water (100 mL) for 1 h. The phases were separated and the aqueous portion extracted with CH_2Cl_2 (3 × 100 mL). The combined organic portions were dried over $MgSO_4$, filtered through a pad of silica gel and concentrated to dryness affording a crude pale yellow solid.

p-Toluenesulfonic acid monohydrate (12.1 g, 63.4 mmol) was added to a suspension of the crude aryl bismacycle (29.6 g, 57.6 mmol) in MeCN (80 mL). The resulting suspension was stirred at room temperature for 1 h then cooled to 0 °C. The product was collected by filtration and washed with ice cold MeCN and Et_2O and dried under a flow of air to afford the title compound (22.7 g, 38.1 mmol, 66% over 2 steps) as a colourless solid. Characterisation data were consistent with literature values.[2]

^{1}H NMR (400 MHz, $CDCl_3$): δ 8.93 (dd, *J* = 7.4, 1.1 Hz, 2H), 8.40 (dd, *J* = 7.7, 1.2 Hz, 2H), 7.84 (d, *J* = 8.0 Hz, 2H), 7.79 (app. td, *J* = 7.5, 1.2 Hz, 2H), 7.49 (app. td, *J* = 7.6, 1.1 Hz, 2H), 7.29 (d, *J* = 8.0 Hz, 2H), 2.42 (s, 3H).

$^{13}C\{^1H\}$ NMR (126 MHz, $CDCl_3$): δ 189.4, 142.6, 139.8, 138.5, 136.4, 135.7, 129.6 (2C), 129.1, 126.6, 21.6.

ν_{max} (ATR)/cm^{-1}: 3051, 1565, 1302, 1223, 1151, 1123, 739, 588, 567.

HRMS calcd. for $C_{19}H_{15}BiO_5S_2$+Na^+: 619.0057 [M+Na]$^+$; found (ESI$^+$): 619.0020.

m.p./°C: 224–225.

10-(4-Fluoro-2-methylphenyl)-10*H*-dibenzo[*b,e*][1,4]thiabismine 5,5-dioxide (2a)

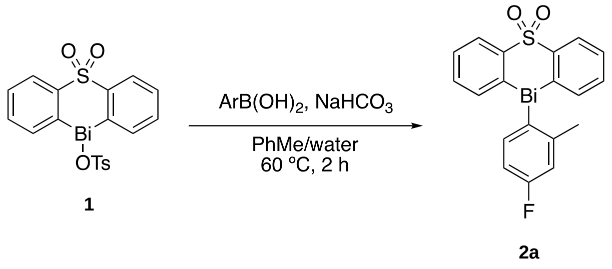

A suspension of bismacycle tosylate **1** (238 mg, 0.400 mmol), 4-fluoro-2-methylphenylboronic acid (67.7 mg, 0.440 mmol) and sodium bicarbonate (33.6 mg, 0.400 mmol) in toluene (8 mL) and water (0.4 mL) was heated at 60 °C for 2 h. The mixture was cooled to room temperature before diluting with Et_2O (20 mL) and washing with 2M aq. NaOH (3 × 10 mL). The organic portion was dried over $MgSO_4$ and concentrated to dryness affording the title compound (211 mg, 0.394 mmol, 99%) as a colourless solid.

^1H NMR (500 MHz, $CDCl_3$): δ 8.38 (dd, *J* = 7.8, 1.4 Hz, 2H), 7.85 (dd, *J* = 7.3, 1.3 Hz, 2H), 7.48 (dd, *J* = 8.3, 6.6 Hz, 1H), 7.41 (app. td, *J* = 7.6, 1.3 Hz, 2H), 7.35 (app. td, *J* = 7.3, 1.4 Hz, 2H), 7.09 (dd, *J* = 10.3, 2.7 Hz, 1H), 6.72 (app. td, *J* = 8.5, 2.6 Hz, 1H), 2.54 (s, 3H).

$^{13}C\{^1H\}$ NMR (126 MHz, $CDCl_3$): δ 163.2 (d, *J* = 247.9 Hz), 162.2 (br), 157.9 (br), 147.4 (d, *J* = 6.9 Hz), 143.4 (d, *J* = 7.4 Hz), 141.9, 137.8, 133.6, 128.5, 127.3, 117.6 (d, *J* = 19.9 Hz), 116.8 (d, *J* = 19.7 Hz), 26.6.

^{19}F NMR (471 MHz, $CDCl_3$): δ -112.08 (ddd, *J* = 10.5, 8.8, 6.6 Hz).

ν_{max} (ATR)/cm^{-1}: 3050, 2973, 1566, 1303, 1223, 1151, 1088, 763, 740, 588, 567.

HRMS calcd. For $C_{19}H_{14}BiFO_2S$+Na^+: 557.0395 [M+Na]$^+$; found (ESI$^+$) 557.0392.

m.p./°C: 219–220.

10-(4-Fluorophenyl)-10*H*-dibenzo[*b*,*e*][1,4]thiabismine 5,5-dioxide (2b)

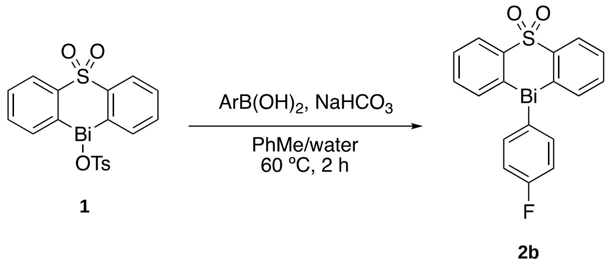

A suspension of bismacycle tosylate **1** (238 mg, 0.400 mmol), 4-fluorophenylboronic acid (61.6 mg, 0.440 mmol) and sodium bicarbonate (33.6 mg, 0.400 mmol) in toluene (8 mL) and water (0.4 mL) was heated at 60 °C for 2 h. The mixture was cooled to room temperature before diluting with Et_2O (20 mL) and washing with 2M aq. NaOH (3 × 10 mL). The organic portion was dried over $MgSO_4$ and concentrated to dryness affording the title compound (207 mg, 0.398 mmol, 99%) as a colourless solid. Characterisation data were consistent with literature values: ^{1}H, ^{13}C and ^{19}F NMR, $\nu_{max.}$[2]

^{1}H NMR (500 MHz, $CDCl_3$): δ 8.38 (dd, *J* = 7.6, 1.4 Hz, 1H), 7.85 (dd, *J* = 7.2, 1.3 Hz, 1H), 7.71 (dd, *J* = 8.3, 6.2 Hz, 1H), 7.41 (app. td, *J* = 7.6, 1.3 Hz, 1H), 7.36 (app. td, *J* = 7.3, 1.4 Hz, 1H), 7.07 (app. t, *J* = 9.0 Hz, 1H).

^{13}C{^{1}H} NMR (126 MHz, $CDCl_3$): δ 163.1 (d, *J* = 248.4 Hz), 160.7, 158.8, 141.8, 141.0 (d, *J* = 7.1 Hz), 137.6, 133.7, 128.4, 127.3, 118.4 (d, *J* = 19.9 Hz).

^{19}F NMR (471 MHz, $CDCl_3$): -111.15 (tt, *J* = 9.3, 6.1 Hz).

ν_{max} (ATR)/cm^{-1}: 3052, 1573, 1485, 1302, 1221, 1151, 1088, 818, 763, 739, 588, 567

HRMS calcd. for $C_{18}H_{12}BiFO_2S+Na^+$: 543.0238 $[M+Na]^+$; found (ESI^+) 543.0227.

m.p./°C: 183-184.

ii. Sulfone Ligands

4,4'-Sulfonylbis((trifluoromethyl)benzene) (L1)

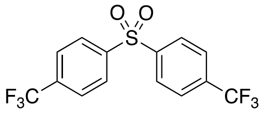

Prepared according to literature procedure, characterisation data were consistent with literature values: ^{1}H, ^{13}C and ^{19}F NMR, m.p.[3]

^{1}H NMR (400 MHz, $CDCl_3$): δ 8.09 (d, *J* = 8.2 Hz, 4H), 7.81 (d, *J* = 8.2 Hz, 4H).
^{13}C{^{1}H} NMR (101 MHz, $CDCl_3$): δ 144.3, 135.6 (q, *J* = 33.4 Hz), 128.6, 126.9 (q, *J* = 3.7 Hz), 123.1 (q, *J* = 273.2 Hz).
^{19}F NMR (377 MHz, $CDCl_3$): δ -63.30.
ν_{max} (ATR)/cm^{-1}: 3015, 1406, 1328, 1320, 1299, 1185, 1171, 1150, 1132, 1108, 1072, 1062, 1016, 843, 736, 720, 701, 622, 601, 562, 428.
HRMS calcd. for $C_{14}H_8F_6O_2S+Na^+$: 377.0041 $[M+Na]^+$; found (ESI^+) 377.0041.
m.p./°C: 143-144.

4,4'-Sulfonylbis(methylbenzene) (L2)

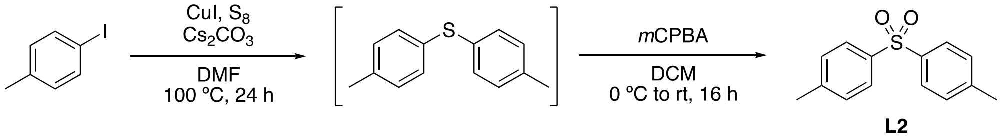

Di-*p*-tolylsulfane was prepared according to literature procedure (7.5 mmol scale),[4] and was used following workup but without purification in the subsequent oxidation. The crude material was dissolved in CH_2Cl_2 (30 mL) and cooled to 0 °C before *m*CPBA (89% purity; 4.36 g, 22.5 mmol) was added portion wise. The reaction was allowed to warm to room temperature overnight then diluted with CH_2Cl_2 (50 mL), washed with 1M aq. NaOH (3 × 30 mL), dried over $MgSO_4$ and concentrated to dryness. The crude material was recrystallised from hot cyclohexane to afford the title compound (1.18 g, 4.80 mmol, 64%) as a colourless solid. Characterisation data were consistent with literature values: ^{1}H and ^{13}C NMR, HRMS, m.p.[5]

^{1}H NMR (400 MHz, $CDCl_3$): δ 7.81 (d, *J* = 8.0 Hz, 4H), 7.28 (d, *J* = 8.0 Hz, 4H), 2.39 (s, 6H).

^{13}C{^{1}H} NMR (101 MHz, $CDCl_3$): δ 144.0, 139.2, 130.0, 127.7, 21.7.

ν_{max} (ATR)/cm^{-1}: 3066,3038, 2976, 2922, 1597, 1318, 1302, 1290, 1153, 1121, 1108, 1072, 819, 710, 679, 633, 558, 546.

HRMS calcd. for $C_{14}H_{14}O_2S$+Na^+: 269.0607 $[M+Na]^+$; found (ESI^+) 269.0611.

m.p./°C: 159-160.

4,4'-Sulfonylbis(methoxybenzene) (L3)

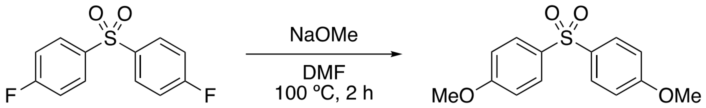

L3

In a flame-dried flask under an atmosphere of N_2, anhydrous MeOH (1.80 mL, 45 mmol) was added dropwise to a suspension of NaH (1.80 g, 45 mmol) in anhydrous DMF (50 mL) and stirred at room temperature for 30 mins. 4,4'-Sulfonylbis(fluorobenzene) (3.81 g, 15 mmol) was added and stirred for 10 mins until the exotherm subsided and then the reaction mixture was heated at 100 °C for 2 h. After cooling to room temperature, the mixture was diluted with EtOAc (100 mL), washed with water (5 × 50 mL), dried over $MgSO_4$ and concentrated to dryness to afford the title compound (3.98 g, 14.3 mmol, 95%) as a colourless solid. Characterisation data were consistent with literature values: ^{1}H and ^{13}C NMR, HRMS, m.p.[6]

^{1}H NMR (400 MHz, $CDCl_3$): δ 7.84 (d, *J* = 8.6 Hz, 4H), 6.94 (d, *J* = 8.6 Hz, 4H), 3.83 (s, 6H).

^{13}C{^{1}H} NMR (101 MHz, $CDCl_3$): δ 163.2, 134.1, 129.7, 114.5, 55.8.

ν_{max} (ATR)/cm^{-1}: 3010, 2842, 1594, 1579, 1496, 1461, 1441, 1414, 1320, 1309, 1295, 1257, 1176, 1149, 1106, 1075, 1024, 834, 806, 718, 682, 627, 560.

HRMS calcd. for $C_{14}H_{14}O_4S$+H^+: 279.0686 $[M+H]^+$; found (ESI^+) 279.0699.

m.p./°C: 129-130.

3,3'-Sulfonylbis((trifluoromethyl)benzene) (L4)

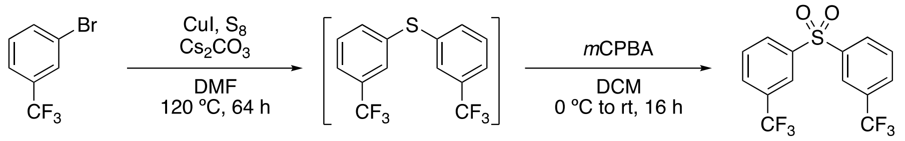

In a flame-dried flask under an atmosphere of N_2, 1-bromo-3-(trifluoromethyl)benzene (5.58 mL, 40.0 mmol) was added to a mixture of CuI (762 mg, 4.00 mmol), sulfur powder (1.28 g, 40.0 mmol) and Cs_2CO_3 (14.3 g, 44.0 mmol) in anhydrous DMF (30 mL). The mixture was heated at 120 °C for 64 h then cooled to room temperature, filtered through a pad of silica gel and eluted with EtOAc (100 mL). The solution was washed with brine (30 mL) and water (3 × 30 mL), dried over $MgSO_4$ and concentrated to dryness. The crude material was dissolved in CH_2Cl_2 (80 mL) and cooled to 0 °C before *m*CPBA (89% purity; 11.6 g, 60.0 mmol) was added portion wise. The reaction was allowed to warm to room temperature overnight then washed with 2M aq. NaOH (3 × 30 mL), dried over $MgSO_4$ and concentrated to dryness. Recrystallisation from hot cyclohexane afforded the title compound (3.92 g, 11.1 mmol, 55%) as a colourless solid.

^{1}H NMR (400 MHz, $CDCl_3$): δ 8.23 (s, 2H), 8.15 (d, *J* = 7.9 Hz, 2H), 7.87 (d, *J* = 7.8 Hz, 2H), 7.71 (app. t, *J* = 7.9 Hz, 2H).

^{13}C{^{1}H} NMR (101 MHz, $CDCl_3$): δ 142.2, 132.5 (q, *J* = 33.7 Hz), 131.3, 130.63 (q, *J* = 3.3 Hz), 130.61, 125.0 (q, *J* = 3.8 Hz), 123.1 (q, *J* = 272.9 Hz).

^{19}F NMR (376 MHz, $CDCl_3$): δ -62.90.

ν_{max} (ATR)/cm^{-1}: 2925, 2855, 1433, 1324, 1307, 1283, 1324, 1307, 1283, 1161, 1129, 1108, 1070, 805, 732, 693, 654, 633, 576, 555.

HRMS calcd. for $C_{14}H_8F_6O_2S+Na^+$: 377.0041 $[M+Na]^+$; found (ESI^+) 377.0045.

m.p./°C: 97–98.

3,3'-Sulfonylbis(methoxybenzene) (L5)

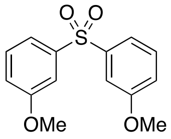

Prepared according to literature procedure, characterisation data were consistent with literature values: ^{1}H and ^{13}C NMR, ν_{max}, HRMS.[7]

^{1}H NMR (400 MHz, CDCl$_3$): δ 7.51 (ddd, *J* = 7.7, 1.7, 1.0 Hz, 2H), 7.45 (dd, *J* = 2.6, 1.7 Hz, 2H), 7.40 (app. t, *J* = 8.0 Hz, 2H), 7.08 (ddd, *J* = 8.2, 2.6, 1.0 Hz, 2H), 3.84 (s, 6H).
^{13}C{^{1}H} NMR (101 MHz, CDCl$_3$): δ 160.1, 142.8, 130.5, 120.1, 119.7, 112.4, 55.8.
ν_{max} (ATR)/cm^{-1}: 2941, 1597, 1580, 1478, 1433, 1307, 1287, 1241, 1184, 1150, 1098, 1037, 863, 786, 706, 687, 614, 528.
HRMS calcd. for $C_{14}H_{14}O_4S+H^+$: 279.0686 $[M+H]^+$; found (ESI$^+$) 279.0688.
m.p./°C: 90-91.

3,3'-Sulfonylbis(chlorobenzene) (L6)

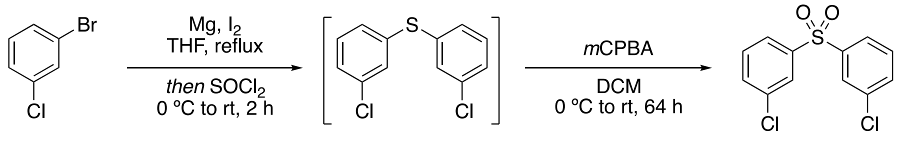

In a flame dried flask under an atmosphere of N_2, magnesium turnings (1.60 g, 66.0 mmol) and ~2 iodine crystals were heated until a purple vapour developed and stirred for 30 mins. Anhydrous THF (60 mL) was added followed by dropwise addition of 1-bromo-3-chlorobenzene (7.05 mL, 60.0 mmol) at a rate that maintained reflux. After cooling to room temperature over 1 h, the mixture was added dropwise to a solution of thionyl chloride (2.19 mL, 30.0 mmol) in anhydrous THF (15 mL) at 0 °C and then stirred at room temperature for 2 h. The reaction mixture was quenched with brine (20 mL) and water (20 mL) and extracted with EtOAc (3 × 30 mL). The combined organic portions were dried over $MgSO_4$, filtered through a pad of silica gel and concentrated to dryness. The crude material was dissolved in CH_2Cl_2 (90 mL) and cooled to 0 °C before *m*CPBA (89% purity; 11.6 g, 60.0 mmol) was added portion wise. The reaction was allowed to warm to room temperature over 3

days and then diluted with EtOAc (150 mL), washed with 2M aq. NaOH (3 × 50 mL), dried over $MgSO_4$ and concentrated to dryness. Recrystallisation from hot EtOH afforded the title compound (4.43 g, 15.4 mmol, 51%) as a colourless solid.

^{1}H NMR (400 MHz, $CDCl_3$): δ 7.92 (app. t, *J* = 1.9 Hz, 2H), 7.83 (app. dt, *J* = 7.6, 1.3 Hz, 2H), 7.56 (ddd, *J* = 8.0, 2.0, 1.1 Hz, 2H), 7.47 (app. t, *J* = 7.9 Hz, 2H).

^{13}C{^{1}H} NMR (101 MHz, $CDCl_3$): δ 142.8, 135.8, 133.9, 130.9, 128.0, 126.1.

ν_{max} (ATR)/cm^{-1}: 3068, 1579, 1461, 1417, 1327, 1296, 1161, 1126, 1075, 813, 789, 782, 679, 601, 584, 511, 488.

HRMS calcd. for $C_{12}H_8Cl_2O_2S+Na^+$: 308.9514 [M+Na]$^+$; found (ESI$^+$) 308.9515.

m.p./°C: 110-111.

iii. Substituted Bismacycles

10-(4-Fluorophenyl)-2,8-bis(trifluoromethyl)-10*H*-dibenzo[*b*,*e*][1,4]thiabismine 5,5-dioxide (S2b)

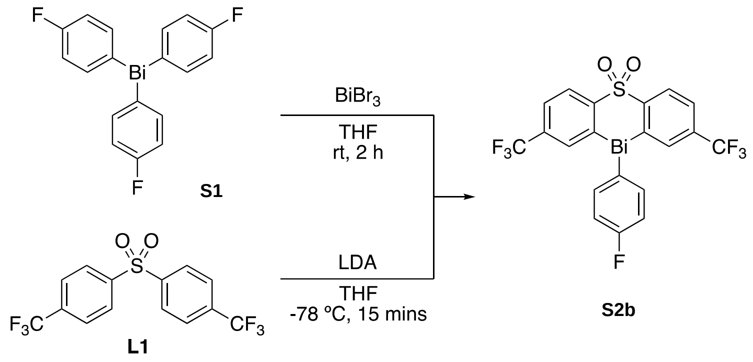

In a flame dried flask under an atmosphere of N_2, a solution of tri(4-fluorophenyl)bismuth **S1** (4.45 g, 9.00 mmol) and $BiBr_3$ (8.08 g, 18.0 mmol) in anhydrous THF (50 mL) was stirred at room temperature for 2 h.

In a flame dried flask under an atmosphere of N_2, *n*-butyllithium (2.5 M in hexanes; 22.7 mL, 56.7 mmol) was added dropwise to a solution of diisopropylamine (7.95 mL, 56.7 mmol) in anhydrous THF (15 mL) at 0 °C. After stirring for 15 mins, the LDA solution was added dropwise to a solution of sulfone ligand **L1** (9.57 g, 27.0 mmol) in anhydrous THF (35 mL) at -78 °C. The resulting solution was stirred at -78 °C for 15 mins.

The bromodiarylbismuth solution was added dropwise *via* cannula to the dilithiated sulfone ligand and the resulting suspension was allowed to warm to room temperature overnight. The mixture was quenched with MeOH (10 mL) before filtering through a pad of silica gel, eluting with CH_2Cl_2 (200 mL) and then concentrating to dryness. Purification by silica gel column chromatography (0-10% EtOAc in cyclohexane) afforded the title compound (6.18 g, 9.41 mmol, 35%) as a colourless solid.

^{1}H NMR (400 MHz, $CDCl_3$): δ 8.49 (d, *J* = 8.1 Hz, 2H), 8.09 (s, 2H), 7.79 – 7.66 (m, 4H), 7.16 (app. t, *J* = 8.6 Hz, 2H).

^{13}C{^{1}H} NMR (101 MHz, $CDCl_3$): δ 163.5 (d, *J* = 250.0 Hz), 162.1 (br), 160.5 (br), 144.6 (q, *J* = 1.7 Hz), 140.9 (d, *J* = 7.4 Hz), 135.5 (q, *J* = 32.6 Hz), 134.5 (q, *J* = 3.6 Hz), 127.9, 125.9 (q, *J* = 3.7 Hz), 123.2 (q, *J* = 273.7 Hz), 119.2 (d, *J* = 20.1 Hz).

^{19}F NMR (376 MHz, CDCl$_3$): δ -62.98 (s, 6F), -109.39 (tt, *J* = 9.1, 5.9 Hz, 1F).

ν_{max} (ATR)/cm^{-1}: 3089, 3059, 1576, 1487, 1385, 1316, 1230, 1174, 1161, 1130, 1066, 906, 820, 733, 718, 628, 568.

HRMS calcd. for $C_{20}H_{10}BiF_7O_2S+Na^+$: 678.9986 [M+Na]$^+$; found (ESI$^+$) 678.9969.

m.p./°C: 182-183.

General Procedure 1 (GP1) – Synthesis of Substituted Bismacycles

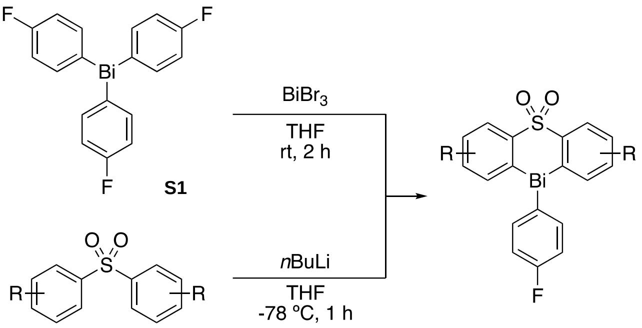

In a flame dried flask under an atmosphere of N_2, a solution of tri(4-fluorophenyl)bismuth (0.33 eq.) and $BiBr_3$ (0.67 eq.) in anhydrous THF (0.4 M) was stirred at room temperature for 2 h.

In a flame dried flask under an atmosphere of N_2, *n*-butyllithium (2.5 M in hexanes; 2.00 eq.) was added dropwise to a solution of the sulfone ligand (1.00 eq.) in anhydrous THF (0.3 M) at -78 °C. The resulting solution was stirred at -78 °C for 1 h.

The bromodiarylbismuth solution was added dropwise *via* cannula to the dilithiated sulfone ligand at -78 °C, then the resulting suspension was allowed to warm to room temperature overnight. The mixture was quenched with MeOH (10 mL) before filtering through a pad of silica gel, eluting with CH_2Cl_2 (200 mL) and then concentrating to dryness. Purification was achieved as described for individual entries.

10-(4-Fluorophenyl)-2,8-dimethyl-10*H*-dibenzo[*b*,*e*][1,4]thiabismine 5,5-dioxide (S3b)

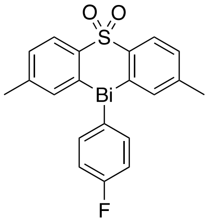

Synthesised according to **GP1** using sulfone ligand **L2** (1.11 g, 4.50 mmol). Purification by silica gel column chromatography (10% EtOAc in cyclohexane) afforded the title compound (1.47 g, 2.68 mmol, 60%) as a colourless solid.

^{1}H NMR (400 MHz, CDCl$_3$): δ 8.24 (d, *J* = 7.9 Hz, 2H), 7.73 (app. td, *J* = 6.2, 1.7 Hz, 2H), 7.62 – 7.61 (m, 2H), 7.19 (dd, *J* = 7.9, 1.7 Hz, 2H), 7.12 – 7.05 (m, 2H), 2.26 (s, 6H).
^{13}C{^{1}H} NMR (101 MHz, CDCl$_3$): δ 163.1 (d, *J* = 248.1 Hz), 160.4 (br), 158.3 (br), 144.2, 141.0 (d, *J* = 7.2 Hz), 139.3, 138.1, 129.0, 127.0, 118.4 (d, *J* = 19.9 Hz), 21.5.
^{19}F NMR (377 MHz, CDCl$_3$): δ -111.38 (tt, *J* = 9.2, 6.1 Hz).
ν_{max} (ATR)/cm^{-1}: 2924, 2855, 1575, 1485, 1446, 1382, 1301, 1283, 1223, 1153, 1135, 1104, 1082, 1020, 910, 810, 733, 710, 677, 632, 553, 525, 503.
HRMS calcd. for $C_{20}H_{16}BiFO_2S+Na^+$: 571.0551 [M+Na]$^+$; found (ESI$^+$) 571.0541.
m.p./°C: 126-127.

10-(4-Fluorophenyl)-2,8-dimethoxy-10*H*-dibenzo[*b,e*][1,4]thiabismine 5,5-dioxide (S4b)

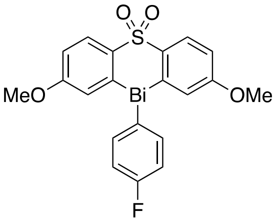

Synthesised according to **GP1** using sulfone ligand **L3** (3.34 g, 12.0 mmol). Purification by silica gel column chromatography (20% EtOAc in cyclohexane) and then recrystallisation from hot EtOH afforded the title compound (3.64 g, 6.28 mmol, 52%) as a colourless solid.

^{1}H NMR (400 MHz, $CDCl_3$): δ 8.28 (d, *J* = 8.6 Hz, 2H), 7.73 (dd, *J* = 8.1, 6.1 Hz, 2H), 7.34 (d, *J* = 2.5 Hz, 2H), 7.09 (app. t, *J* = 8.8 Hz, 2H), 6.84 (dd, *J* = 8.6, 2.5 Hz, 2H), 3.70 (s, 6H).
^{13}C{^{1}H} NMR (101 MHz, $CDCl_3$): δ 164.4, 162.8 (d, *J* = 171.8 Hz), 161.5 (br), 160.1 (br), 141.0 (d, *J* = 7.2 Hz), 134.2, 128.6, 123.6, 118.4 (d, *J* = 19.9 Hz), 112.7, 55.7.
^{19}F NMR (377 MHz, $CDCl_3$): δ -111.14 (tt, *J* = 9.4, 6.1 Hz).
ν_{max} (ATR)/cm^{-1}: 2939, 1572, 1485, 1460, 1428, 1290, 1266, 1222, 1160, 1129, 1104, 1078, 1028, 817, 731, 682, 570, 540, 504.
HRMS calcd. for $C_{20}H_{16}BiFO_4S+Na^+$: 603.0450 [M+Na]$^+$; found (ESI$^+$) 603.0443.
m.p./°C: 169-170.

2,8-Difluoro-10-(4-fluorophenyl)-10*H*-dibenzo[*b*,*e*][1,4]thiabismine 5,5-dioxide (S5b)

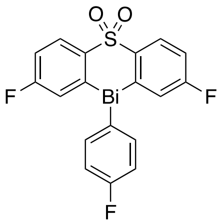

Synthesised according to **GP1** using 4,4'-sulfonylbis(fluorobenzene) (3.81 g, 15.0 mmol). Purification by silica gel column chromatography (0-6% EtOAc in cyclohexane) and then recrystallisation from hot cyclohexane afforded the title compound (2.56 g, 4.60 mmol, 31%) as a colourless solid.

^{1}H NMR (400 MHz, CDCl$_3$): δ 8.38 (dd, *J* = 8.6, 4.7 Hz, 2H), 7.74 (dd, *J* = 8.1, 5.9 Hz, 2H), 7.56 (dd, *J* = 7.0, 2.5 Hz, 2H), 7.21 – 7.04 (m, 4H).

^{13}C{^{1}H} NMR (101 MHz, CDCl$_3$): δ 166.3 (d, *J* = 261.0 Hz), 163.3 (d, *J* = 249.6 Hz), 162.3 (br), 161.8 (br), 140.9 (d, *J* = 7.3 Hz), 137.6 (d, *J* = 2.9 Hz), 129.7 (d, *J* = 8.4 Hz), 124.7 (d, *J* = 20.9 Hz), 119.0 (d, *J* = 20.0 Hz), 115.6 (d, *J* = 22.8 Hz).

^{19}F NMR (377 MHz, CDCl$_3$): δ -105.62 (app. td, *J* = 7.9, 4.8 Hz, 2F), -110.01 (tt, *J* = 9.2, 5.9 Hz, 1F).

ν_{max} (ATR)/cm^{-1}: 3080, 3054, 1570, 1486, 1439, 1307, 1288, 1251, 1227, 1201, 1161, 1150, 1101, 1073, 855, 820, 733, 713, 680, 558, 532, 500.

HRMS calcd. for $C_{18}H_{10}BiF_3O_2S+Na^+$: 579.0050 $[M+Na]^+$; found (ESI$^+$) 579.0039.

m.p./°C: 156-157.

10-(4-Fluorophenyl)-3,7-bis(trifluoromethyl)-10*H*-dibenzo[*b*,*e*][1,4]thiabismine 5,5-dioxide (S6b)

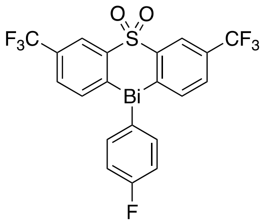

Synthesised according to **GP1** using sulfone ligand **L4** (3.19 g, 9.00 mmol). Purification by silica gel column chromatography (0-10% EtOAc in cyclohexane) afforded the title compound (1.57 g, 2.40 mmol, 27%) as a colourless solid.

^{1}H NMR (400 MHz, $CDCl_3$): δ 8.63 (d, *J* = 1.8 Hz, 2H), 8.01 (d, *J* = 7.7 Hz, 2H), 7.71 (dd, *J* = 8.1, 5.9 Hz, 2H), 7.63 (dd, *J* = 7.7, 1.8 Hz, 2H), 7.14 (app. t, *J* = 8.8 Hz, 2H).

^{13}C{^{1}H} NMR (101 MHz, $CDCl_3$): δ 164.0 (br), 163.4 (d, *J* = 250.0 Hz), 161.8 (br), 142.3, 141.0 (d, *J* = 7.3 Hz), 138.4, 131.6 (q, *J* = 33.7 Hz), 130.3 (q, *J* = 3.5 Hz), 123.2 (q, *J* = 273.0 Hz), 124.4 (q, *J* = 3.9 Hz), 119.1 (d, *J* = 20.0 Hz).

^{19}F NMR (377 MHz, $CDCl_3$): δ -62.85 (s, 6F), -109.63 (tt, *J* = 9.1, 5.9 Hz, 1F).

ν_{max} (ATR)/cm^{-1}: 3080, 3058, 2923, 1594, 1576, 1487, 1384, 1325, 1253, 1229, 1175, 1131, 1095, 1072, 909, 840, 820, 733, 713, 664, 574, 559.

HRMS calcd. for $C_{20}H_{10}BiF_7O_2S+Na^+$: 678.9986 [M+Na]$^+$; found (ESI$^+$) 678.9967.

m.p./°C: 199-200.

10-(4-Fluorophenyl)-1,9-dimethoxy-10*H*-dibenzo[*b*,*e*][1,4]thiabismine 5,5-dioxide (S7b)

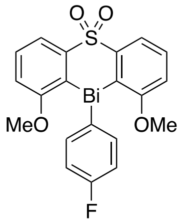

Synthesised according to **GP1** using sulfone ligand **L5** (1.67 g, 6.00 mmol). Purification by recrystallisation from hot cyclohexane afforded the title compound (854 mg, 1.47 mmol, 25%) as a colourless solid.

^{1}H NMR (400 MHz, $CDCl_3$): δ 8.08 (dd, *J* = 7.7, 0.9 Hz, 2H), 7.52 (dd, *J* = 8.1, 6.1 Hz, 2H), 7.42 (app. t, *J* = 7.9 Hz, 2H), 6.96 (dd, *J* = 8.2, 0.9 Hz, 2H), 6.87 (app. t, *J* = 8.8 Hz, 2H), 3.34 (s, 6H).

^{13}C{^{1}H} NMR (101 MHz, $CDCl_3$): δ 162.0 (d, *J* = 245.3 Hz), 161.1, 157.0 (br), 144.4 (br), 142.4, 138.9 (d, *J* = 6.8 Hz), 130.4, 119.6, 117.1 (d, *J* = 19.7 Hz), 115.2, 55.8.

^{19}F NMR (377 MHz, $CDCl_3$): δ -114.12 (tt, *J* = 9.4, 6.1 Hz).

ν_{max} (ATR)/cm^{-1}: 2937, 1567, 1485, 1456, 1429, 1296, 1254, 1219, 1178, 1160, 1118, 1038, 1016, 858, 816, 786, 731, 720, 619, 507.

HRMS calcd. for $C_{20}H_{16}BiFO_4S+Na^+$: 603.0450 $[M+Na]^+$; found (ESI^+) 603.0449.

m.p./°C: 215-216.

1,9-Dichloro-10-(4-fluorophenyl)-10*H*-dibenzo[*b*,*e*][1,4]thiabismine 5,5-dioxide (S8b)

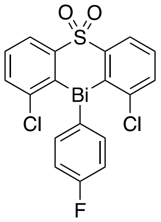

Synthesised according to **GP1** using sulfone ligand **L6** (2.15 g, 7.50 mmol). Purification by silica gel column chromatography (0-10% EtOAc in cyclohexane) and then recrystallisation from hot MeCN afforded the title compound (2.09 mg, 3.55 mmol, 47%) as a colourless solid.

^{1}H NMR (400 MHz, $CDCl_3$): δ 8.41 (dd, *J* = 7.5, 1.4 Hz, 2H), 7.61 (dd, *J* = 8.1, 5.9 Hz, 2H), 7.48 (dd, *J* = 8.0, 1.4 Hz, 2H), 7.43 (app. t, *J* = 7.7 Hz, 2H), 6.94 (app. t, *J* = 8.7 Hz, 2H).
^{13}C{^{1}H} NMR (101 MHz, $CDCl_3$): δ 162.4 (d, *J* = 247.4 Hz), 160.3 (br), 158.3 (br), 142.7, 141.6, 140.0 (d, *J* = 7.3 Hz), 134.2, 130.4, 125.3, 118.3 (d, *J* = 20.1 Hz).
^{19}F NMR (377 MHz, $CDCl_3$): δ -112.03 (tt, *J* = 9.3, 5.9 Hz).
ν_{max} (ATR)/cm^{-1}: 3072, 2927, 2856, 1574, 1485, 1422, 1310, 1275, 1224, 1160, 1133, 1113, 1097, 1014, 910, 816, 793, 774, 732, 697, 602, 590, 529, 509.
HRMS calcd. for $C_{18}H_{10}BiCl_2FO_2S+Na^+$: 610.9459 $[M+Na]^+$; found (ESI^+) 610.9465.
m.p./°C: 255-256.

General Procedure 2 (GP2) – Protodebismuthation / Transmetallation

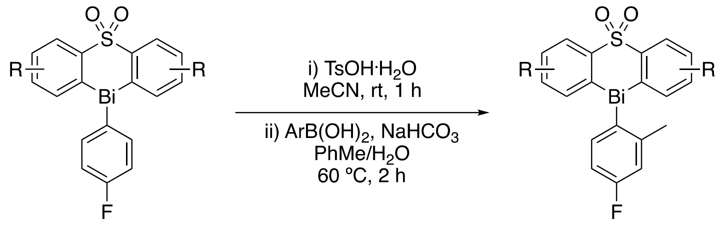

p-Toluenesulfonic acid monohydrate (1.1 eq.) was added to a suspension of crude aryl bismacycle (1.0 eq.) in MeCN (0.1 M) and stirred at room temperature for 1 h before concentrating to dryness. 4-Fluoro-2-methylphenylboronic acid (1.1 eq.), $NaHCO_3$ (2.0 eq.), toluene (0.1 M) and water (2 vol%) were added and the reaction mixture was heated at 60 °C for 2 h. After cooling to room temperature, the mixture was diluted with EtOAc (100 mL), washed with 1M aq. NaOH (3 × 20 mL), dried over $MgSO_4$ and concentrated to dryness.

10-(4-Fluoro-2-methylphenyl)-2,8-bis(trifluoromethyl)-10*H*-dibenzo[*b*,*e*][1,4]thiabismine 5,5-dioxide (S2a)

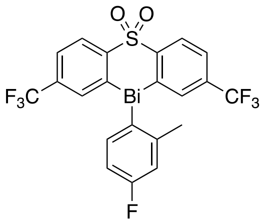

Using aryl bismacycle **S2b** (328 mg, 0.500 mmol) in **GP2** afforded the title compound (334 mg, 0.498 mmol, quant.) as a colourless solid.

^{1}H NMR (400 MHz, CDCl$_3$): δ 8.47 (d, *J* = 8.0 Hz, 2H), 8.21 – 8.02 (m, 2H), 7.71 (dd, *J* = 8.2, 1.7 Hz, 2H), 7.44 (dd, *J* = 8.3, 6.5 Hz, 1H), 7.16 (dd, *J* = 10.2, 2.7 Hz, 1H), 6.78 (app. td, *J* = 8.5, 2.7 Hz, 1H), 2.60 (s, 3H).
^{13}C{^{1}H} NMR (101 MHz, CDCl$_3$): δ 163.7 (br), 163.5 (d, *J* = 249.6 Hz), 159.9 (br), 147.5 (d, *J* = 7.3 Hz), 144.7 (q, *J* = 1.6 Hz), 143.4 (d, *J* = 7.8 Hz), 135.3 (q, *J* = 32.5 Hz), 134.8 (q, *J* = 3.5 Hz), 127.8, 125.9 (q, *J* = 3.7 Hz), 123.2 (q, *J* = 273.6 Hz), 118.5 (d, *J* = 20.2 Hz), 117.7 (d, *J* = 20.0 Hz), 26.3 (d, *J* = 1.8 Hz).
^{19}F NMR (376 MHz, CDCl$_3$): δ -62.95 (s, 6F), -110.41 (ddd, *J* = 10.4, 8.7, 6.5 Hz, 1F).
ν_{max} (ATR)/cm^{-1}: 3082, 2978, 2923, 1571, 1384, 1314, 1292, 1224, 1173, 1158, 1129, 1082, 1065, 1019, 907, 842, 810, 733, 718, 628, 568, 439.
HRMS calcd. for $C_{21}H_{12}BiF_7O_2S+Na^+$: 693.0142 [M+Na]$^+$; found (ESI$^+$) 693.0111.
m.p./°C: 164-165.

10-(4-Fluoro-2-methylphenyl)-2,8-dimethyl-10*H*-dibenzo[*b*,*e*][1,4]thiabismine 5,5-dioxide (S3a)

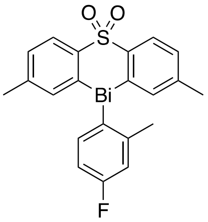

Using aryl bismacycle **S3b** (281 mg, 0.500 mmol) in **GP2** afforded the title compound (270 mg, 0.480 mmol, 96%) as a colourless solid.

^{1}H NMR (400 MHz, CDCl$_3$): δ 8.23 (d, *J* = 7.9 Hz, 2H), 7.62 (d, *J* = 1.7 Hz, 2H), 7.51 (dd, *J* = 8.3, 6.6 Hz, 1H), 7.22 – 7.14 (m, 2H), 7.09 (dd, *J* = 10.3, 2.7 Hz, 1H), 6.74 (app. td, *J* = 8.6, 2.7 Hz, 1H), 2.54 (s, 3H), 2.26 (s, 6H).

^{13}C{^{1}H} NMR (101 MHz, CDCl$_3$): δ 163.2 (d, *J* = 247.4 Hz), 162.0 (br), 157.4 (br), 147.4 (d, *J* = 6.9 Hz), 144.1, 143.5 (d, *J* = 7.3 Hz), 139.4, 138.3, 129.0, 126.9, 117.5 (d, *J* = 19.8 Hz), 116.7 (d, *J* = 19.5 Hz), 26.7 (d, *J* = 2.0 Hz), 21.5.

^{19}F NMR (377 MHz, CDCl$_3$): δ -112.33 (ddd, *J* = 10.3, 8.8, 6.6 Hz).

ν_{max} (ATR)/cm^{-1}: 2922, 1577, 1467, 1444, 1379, 1301, 1283, 1223, 1152, 1135, 1104, 1082, 1021, 939, 910, 821, 732, 710, 677, 632, 553, 525.

HRMS calcd. for $C_{21}H_{18}BiFO_2S+Na^+$: 585.0708 [M+Na]$^+$; found (ESI$^+$) 585.0702.

m.p./°C: 230-231.

10-(4-Fluoro-2-methylphenyl)-2,8-dimethoxy-10*H*-dibenzo[*b*,*e*][1,4]thiabismine 5,5-dioxide (S4a)

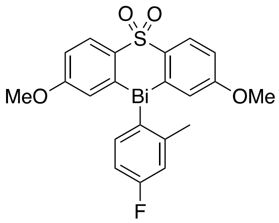

Using aryl bismacycle **S4b** (580 mg, 1.00 mmol) in **GP2** afforded the title compound (590 mg, 0.993 mmol, 99%) as a colourless solid.

^{1}H NMR (400 MHz, $CDCl_3$): δ 8.27 (d, *J* = 8.6 Hz, 2H), 7.59 (dd, *J* = 8.3, 6.6 Hz, 1H), 7.34 (d, *J* = 2.5 Hz, 2H), 7.08 (dd, *J* = 10.3, 2.6 Hz, 1H), 6.85 (dd, *J* = 8.6, 2.5 Hz, 2H), 6.76 (app. td, *J* = 8.5, 2.7 Hz, 1H), 3.71 (s, 6H), 2.55 (s, 3H).

$^{13}C\{^1H\}$ NMR (101 MHz, $CDCl_3$): δ 163.5, 163.2 (d, *J* = 247.9 Hz), 163.0 (br), 159.2 (br), 147.3 (d, *J* = 7.0 Hz), 143.6 (d, *J* = 7.4 Hz), 134.3, 128.6, 123.8, 117.6 (d, *J* = 19.9 Hz), 116.8 (d, *J* = 19.6 Hz), 112.6, 55.7, 26.6 (d, *J* = 1.9 Hz).

^{19}F NMR (376 MHz, $CDCl_3$): δ -112.10 (ddd, *J* = 10.3, 8.8, 6.6 Hz).

ν_{max} (ATR)/cm^{-1}: 2938, 1568, 1460, 1427, 1288, 1265, 1220, 1151, 1128, 1102, 1077, 1027, 1016, 908, 860, 813, 728, 681, 653, 569, 539, 504.

HRMS calcd. for $C_{21}H_{18}BiFO_4S+Na^+$: 617.0606 $[M+Na]^+$; found (ESI^+) 617.0590.

m.p./°C: 170-171.

2,8-Difluoro-10-(4-fluoro-2-methylphenyl)-10*H*-dibenzo[*b*,*e*][1,4]thiabismine 5,5-dioxide (S5a)

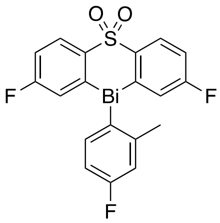

Using aryl bismacycle **S5b** (556 mg, 1.00 mmol) in **GP2** afforded the title compound (558 mg, 0.978 mmol, 98%) as a colourless solid.

^{1}H NMR (400 MHz, CDCl$_3$): δ 8.37 (dd, *J* = 8.6, 4.8 Hz, 2H), 7.63 – 7.42 (m, 3H), 7.19 – 6.99 (m, 3H), 6.81 (app. td, *J* = 8.5, 2.7 Hz, 1H), 2.56 (s, 3H).

^{13}C{^{1}H} NMR (101 MHz, CDCl$_3$): δ 166.1 (d, *J* = 260.9 Hz), 163.8 (br), 163.4 (d, *J* = 248.8 Hz), 161.0 (br), 147.4 (d, *J* = 7.2 Hz), 143.5 (d, *J* = 7.6 Hz), 137.7 (d, *J* = 2.9 Hz), 129.6 (d, *J* = 8.4 Hz), 124.8 (d, *J* = 20.7 Hz), 118.2 (d, *J* = 20.0 Hz), 117.5 (d, *J* = 19.7 Hz), 115.6 (d, *J* = 22.9 Hz), 26.4 (d, *J* = 1.8 Hz).

^{19}F NMR (376 MHz, CDCl$_3$): δ -105.63 (ddd, *J* = 8.4, 6.9, 4.7 Hz, 2F), -110.99 (ddd, *J* = 10.2, 8.7, 6.5 Hz, 1F).

ν_{max} (ATR)/cm^{-1}: 3079, 3051, 2976, 2923, 1568, 1468, 1439, 1307, 1287, 1251, 1223, 1200, 1151, 1119, 1101, 1073, 940, 909, 865, 827, 733, 713, 680, 557, 530, 499.

HRMS calcd. for $C_{19}H_{12}BiF_3O_2S+Na^+$: 593.0206 $[M+Na]^+$; found (ESI^+) 593.0193.

m.p./°C: 197-198.

10-(4-Fluoro-2-methylphenyl)-3,7-bis(trifluoromethyl)-10*H*-dibenzo[*b*,*e*][1,4]thiabismine 5,5-dioxide (S6a)

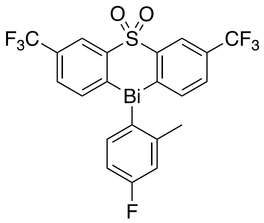

Using aryl bismacycle **S6b** (131 mg, 0.200 mmol) in **GP2** afforded the title compound (127 mg, 0.189 mmol, 95%) as a colourless solid.

^{1}H NMR (400 MHz, CDCl$_3$): δ 8.65 – 8.60 (m, 2H), 8.01 (d, *J* = 7.6 Hz, 2H), 7.62 (dd, *J* = 7.6, 1.8 Hz, 2H), 7.43 (dd, *J* = 8.3, 6.5 Hz, 1H), 7.15 (dd, *J* = 10.3, 2.6 Hz, 1H), 6.77 (app. td, *J* = 8.5, 2.7 Hz, 1H), 2.61 (s, 3H).

^{13}C{^{1}H} NMR (101 MHz, CDCl$_3$): δ 163.4 (d, *J* = 249.3 Hz), 163.3 (br), 163.2 (br), 147.4 (d, *J* = 7.2 Hz), 143.6 (d, *J* = 7.5 Hz), 142.4, 138.6, 131.6 (q, *J* = 33.7 Hz), 130.2 (q, *J* = 3.5 Hz), 123.3 (q, *J* = 273.1 Hz), 124.4 (q, *J* = 3.7 Hz), 118.3 (d, *J* = 20.1 Hz), 117.7 (d, *J* = 19.9 Hz), 26.5 (d, *J* = 1.8 Hz).

^{13}C NMR (101 MHz, CDCl$_3$): δ 163.7 (br), 163.5 (d, *J* = 249.6 Hz), 159.9 (br), 147.5 (d, *J* = 7.3 Hz), 144.7 (q, *J* = 1.6 Hz), 143.4 (d, *J* = 7.8 Hz), 135.3 (q, *J* = 32.5 Hz), 134.8 (q, *J* = 3.5 Hz), 127.8, 125.9 (q, *J* = 3.7 Hz), 123.2 (q, *J* = 273.6 Hz), 118.5 (d, *J* = 20.2 Hz), 117.7 (d, *J* = 20.0 Hz), 26.3 (d, *J* = 1.8 Hz).

^{19}F NMR (377 MHz, CDCl$_3$): δ -62.84 (s, 6F), -110.62 (ddd, *J* = 10.3, 8.7, 6.5 Hz, 1F).

ν_{max} (ATR)/cm^{-1}: 2925, 1594, 1571, 1383, 1325, 1253, 1224, 1175, 1132, 1095, 1072, 910, 840, 734, 713, 664, 574, 559.

HRMS calcd. for $C_{21}H_{12}BiF_7O_2S+Na^+$: 693.0142 [M+Na]$^+$; found (ESI$^+$) 693.0154.

m.p./°C: 201-202.

iv. Substrates

(2-Methylnaphthalen-1-yl)boronic acid

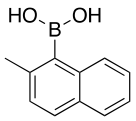

Prepared according to literature procedure.[8] Characterisation data were consistent with literature values: ^{1}H and ^{13}C NMR, m.p.[9]

^{1}H NMR (400 MHz, CDCl$_3$): δ 7.81 (m, 2H), 7.76 (d, *J* = 8.4 Hz, 1H), 7.50 – 7.38 (m, 2H), 7.30 (d, *J* = 8.4 Hz, 1H), 4.92 (br s, 2H), 2.57 (s, 3H).

^{13}C{^{1}H} NMR (101 MHz, CDCl$_3$): δ 138.2, 135.1, 131.4, 129.0, 128.43, 128.38, 127.5, 126.4, 125.1, 22.7.*

ν_{max} (ATR)/cm^{-1}: 3530 (br), 3356 (br), 3049, 1592, 1509, 1425, 1396, 1356, 1336, 1303, 1260, 1218, 1149, 1094, 1030, 978, 811, 783, 741.

HRMS calcd. for $C_{11}H_{11}BO_2+Na^+$: 209.0744 [M+Na]$^+$; found (ESI$^+$) 209.0744.

m.p./°C: 123-124.

* Carbon bonded to boron not observed.

tert-Butyl (_tert_-butoxycarbonyl)(4-hydroxypyridin-2-yl)carbamate

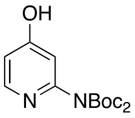

Prepared according to literature procedure.[10]

^{1}H NMR (400 MHz, $CDCl_3$): δ 7.97 (d, *J* = 6.3 Hz, 1H), 6.68 (dd, *J* = 6.3, 2.3 Hz, 1H), 6.58 (d, *J* = 2.3 Hz, 1H), 1.42 (s, 18H).

^{13}C{^{1}H} NMR (101 MHz, $CDCl_3$): δ 171.1 (br), 150.8, 150.7 (br), 145.4 (br), 112.9, 111.4, 84.1, 28.0.

ν_{max} (ATR)/cm^{-1}: 2980, 2934, 2652 (br), 1771, 1729, 1607, 1524, 1458, 1394, 1370, 1276, 1253, 1154, 1116, 1007, 916, 849, 777, 733.

HRMS calcd. for $C_{15}H_{22}N_2O_5$-H^+: 309.1456 [M-H]$^-$; found (ESI$^-$) 309.1448.

m.p./°C: 167-168.

tert-Butyl (4-hydroxypyridin-2-yl)carbamate

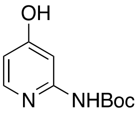

Prepared according to literature procedure.[10]

^{1}H NMR (400 MHz, CD_3CN): δ 8.75 (br s, 1H), 7.86 (d, *J* = 6.2 Hz, 1H), 6.99 (br s, 1H), 6.38 (dd, *J* = 6.2, 2.3 Hz, 1H), 1.49 (s, 9H).

^{13}C{^{1}H} NMR (101 MHz, CD_3CN): δ 170.4, 153.8, 153.1, 146.2, 109.9, 100.0, 81.8, 28.4.

ν_{max} (ATR)/cm^{-1}: 3185 (br), 2978, 2929, 1717, 1650, 1599, 1504, 1453, 1393, 1368, 1310, 12789, 1255, 1231, 1192, 1156, 1109, 1055, 998, 862, 774, 733, 542.

HRMS calcd. for $C_{10}H_{14}N_2O_3$-H^+: 209.0932 [M-H]$^-$; found (ESI$^-$) 209.0926.

m.p./°C: 147-148.

(4-(((Benzyloxy)carbonyl)amino)-3-fluorophenyl)boronic acid

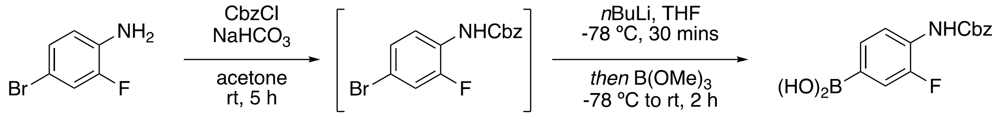

Benzyl chloroformate (1.50 mL, 10.5 mmol) was added to a suspension of 4-bromo-2-fluoroaniline (1.90 g, 10.0 mmol) and $NaHCO_3$ (1.68 g, 20.0 mmol) in acetone (20 mL) at 0 °C. The mixture was stirred at room temperature for 5 h, then diluted with EtOAc (50 mL) and washed with 2M aq. NaOH (3 × 10 mL) and 4M HCl (3 × 10 mL). The organic portion was dried over $MgSO_4$ and concentrated to dryness giving a solid which was washed with pentane (3 × 5 mL) and dried under a flow of air. In a flame dried flask under an atmosphere of N_2, the crude material was dissolved in anhydrous THF (50 mL) and cooled to -78 °C. *n*-Butyllithium (2.5 M in hexanes; 8.40 mL, 21.0 mmol) was added dropwise and the mixture was stirred at -78 °C for 30 mins before trimethyl borate (3.34 mL, 30.0 mmol) was added. After warming to room temperature over 2 h, the reaction mixture was quenched with 1M HCl (10 mL), diluted with EtOAc (100 mL) and washed with brine (3 × 30 mL). The organic portion was dried over $MgSO_4$ and concentrated to dryness. The crude solid was washed with 9:1 pentane/CH_2Cl_2 (3 × 5 mL) and 3:2 EtOH/water (3 × 5 mL) and dried under a flow of air to afford the title compound (818 mg, 2.83 mmol, 28%) as a colourless solid.

^{1}H NMR (400 MHz, DMSO-*d*$_6$): δ 9.51 (s, 1H), 8.12 (s, 2H), 7.68 (t, *J* = 8.0 Hz, 1H), 7.58 – 7.50 (m, 2H), 7.46 – 7.32 (m, 5H), 5.16 (s, 2H).

^{13}C{^{1}H} NMR (101 MHz, DMSO-*d*$_6$): δ 153.7, 153.3 (d, *J* = 245.4 Hz), 136.5, 131.2 (br), 130.3 (d, *J* = 3.3 Hz), 128.5, 128.0, 128.0, 127.8 (d, *J* = 11.6 Hz), 122.5 (br), 120.4 (d, *J* = 16.9 Hz), 66.1.

^{19}F NMR (376 MHz, DMSO-*d*$_6$): δ -126.65 (br app. s).

ν_{max} (ATR)/cm^{-1}: 3434, 3358, 1742, 1621, 1590, 1542, 1493, 1420, 1390, 1344, 1323, 1238, 1120, 1062, 826, 766, 732, 694, 669, 548.

HRMS calcd. for $C_{14}H_{13}BFNO_4$-H^+: 288.0849 [M-H]$^-$; found (ESI$^-$) 288.0848.

m.p./°C: 201-202.

***N*-(4-Fluorophenyl)-6-hydroxypicolinamide**

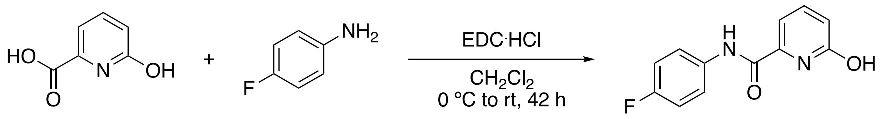

EDC·HCl (1.05 g, 5.50 mmol) was added to a solution of 6-hydroxypicolinic acid (696 mg, 5.00 mmol) and 4-fluoroaniline (474 μL, 5.00 mmol) in CH_2Cl_2 (50 mL) at 0 °C. The reaction mixture was stirred at 0 °C for 2 h then room temperature for 40 h before concentrating to dryness. Water (40 mL) was added and stirred for 10 mins before the precipitate was collected by filtration. The solid was washed with water (10 mL) and pentane (10 mL) and dried under a flow of air to afford the title compound (793 mg, 3.42 mmol, 68%) as an off-white solid.

^{1}H NMR (400 MHz, DMSO-*d*$_6$): δ 11.26 (s, 1H), 10.29 (s, 1H), 7.96 – 7.59 (m, 3H), 7.40 (d, *J* = 7.1 Hz, 1H), 7.21 (app. t, *J* = 8.9 Hz, 2H), 6.82 (d, *J* = 8.5 Hz, 1H).

^{13}C{^{1}H} NMR (126 MHz, DMSO-*d*$_6$): δ 162.4, 161.5, 158.5 (d, *J* = 240.8 Hz), 145.4 (br), 140.6, 134.6 (d, *J* = 2.5 Hz), 121.9 (d, *J* = 7.9 Hz), 116.4 (br), 115.4 (d, *J* = 22.2 Hz), 111.8 (br).

^{19}F NMR (376 MHz, DMSO-*d*$_6$): δ -118.39 (br app. s).

ν_{max} (ATR)/cm^{-1}: 3277, 2771 (br), 2731 (br), 1673, 1647, 1611, 1544, 1508, 1467, 1408, 1318, 1215, 1163, 1007, 942, 830, 813, 657, 557, 524, 494.

HRMS calcd. for $C_{12}H_9FN_2O_2$-H^+: 231.0575 [M-H]$^-$; found (ESI$^-$) 231.0572.

m.p./°C: 260-261.

4. Optimisation

i. Optimisation of Conditions and Additives

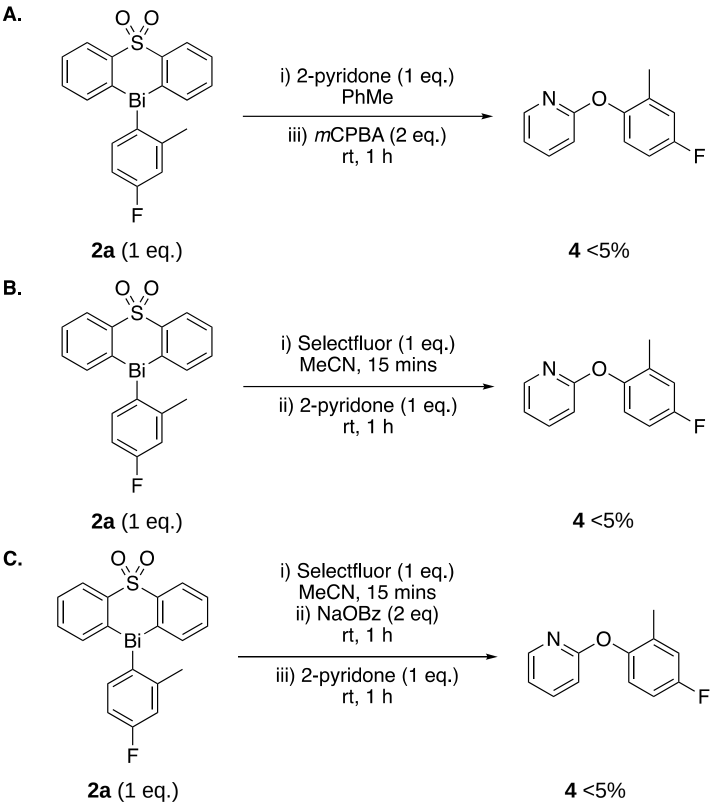

Figure S1. Initial evaluation of potential oxidants and systems. Mass balance is composed of unreacted pyridone and arylbismacycle (from Bi(V) reduction) / arene (from formal protodebismuthation) / arylbenzoate (from C-O ligand coupling).

Procedure for A: A stock solution of aryl bismacycle **2a** (1 eq.), Selectfluor (1 eq.) and 4,4′-bis(trifluoromethyl)-1,1′-biphenyl (internal standard for ^{19}F NMR spectroscopy) in $CDCl_3$ (0.04 M) was stirred for 15 mins. An aliquot was taken and analysed by quantitative ^{19}F NMR spectroscopy. 2-Pyridone (1.90 mg, 0.020 mmol) was added to an aliquot of the stock solution (0.5 mL, 0.020 mmol scale) in an NMR tube followed by *m*CPBA (89% purity; 7.76 mg, 0.040 mmol) and mixed for 1 h before analysing by quantitative ^{19}F NMR spectroscopy.

Procedure for B & C: A stock solution of aryl bismacycle **2a** (1 eq.), Selectfluor (1 eq.) and 4,4′-bis(trifluoromethyl)-1,1′-biphenyl (internal standard for ^{19}F NMR spectroscopy) in

CD_3CN (0.04 M) was stirred for 10 mins. An aliquot was taken and analysed by quantitative ^{19}F NMR spectroscopy.

B: 2-Pyridone (1.90 mg, 0.020 mmol) was added to an aliquot of the stock solution (0.5 mL, 0.020 mmol scale) in an NMR tube and mixed for 1 h before analysing by quantitative ^{19}F NMR spectroscopy.

C: NaOBz (5.76 mg, 0.040 mmol) was added to an aliquot of the stock solution (0.5 mL, 0.020 mmol scale) in an NMR tube and mixed for 1 h. 2-Pyridone (1.90 mg, 0.020 mmol) was added and mixed for 1 h before analysing by quantitative ^{19}F NMR spectroscopy.

Commentary: Use of *m*CPBA as the oxidant afforded a complex mixture. Use of Selectfluor as the oxidant with or without NaOBz addition showed no reaction after 1 h at room temperature.

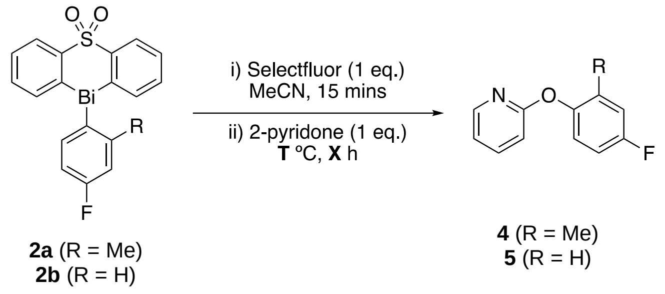

Entry	T	X	5 (%)	4 (%)
1	80	2	30	72
2	60	6	16	61
3	80	4	36	84
4	100	2	52	85

Table S1. Initial optimisation. Yields determined by ^{19}F NMR spectroscopy against an internal standard. Mass balance is composed of unreacted pyridone and arylbismacycle (from Bi(V) reduction) / arene (from formal protodebismuthation) / arylbenzoate (from C-O ligand coupling).

Procedure for R = H: A stock solution of aryl bismacycle **2b** and 4,4′-bis(trifluoromethyl)-1,1′-biphenyl (internal standard for ^{19}F NMR spectroscopy) in 1:1 MeCN/CD_3CN (0.04 M) was prepared. An aliquot was taken and analysed by quantitative ^{19}F NMR spectroscopy. Selectfluor (7.09 mg, 0.020 mmol) was added to an aliquot of the stock solution (0.5 mL, 0.020 mmol scale) and mixed for 15 mins before a solution of 2-pyridone (1.90 mg, 0.020 mmol) in MeCN (0.1 mL) was added and heated at **T** °C for **X** h before analysing by quantitative ^{19}F NMR spectroscopy.

Procedure for R = Me: A stock solution of aryl bismacycle **2a** (1 eq.), Selectfluor (1 eq.) and 4,4'-bis(trifluoromethyl)-1,1'-biphenyl (internal standard for ^{19}F NMR spectroscopy) in 1:1 MeCN/CD_3CN (0.04 M) was stirred for 15 mins. An aliquot was taken and analysed by quantitative ^{19}F NMR spectroscopy. A solution of 2-pyridone (1.90 mg, 0.020 mmol) in MeCN (0.1 mL) was added to an aliquot of the stock solution (0.5 mL, 0.020 mmol scale) and heated at **T** °C for **X** h before analysing by quantitative ^{19}F NMR spectroscopy.

Commentary: Following oxidation with Selectfluor, heating at 80 °C for 2 h in the presence of 2-pyridone afforded the O-arylated products for both *ortho*- and non-*ortho*-substituted examples in good and moderate yields, respectively (entry 1). Reducing the reaction temperature resulted in slower reaction and thus lower yields (entry 2). Increasing the reaction time afforded an excellent yield for the *ortho*-substituted example (entry 3). Increasing the reaction temperature to 100 °C gave a similar result to entry 3 for the *ortho*-substituted example and also gave a moderate increase in yield for the non-*ortho*-substituted example (entry 4).

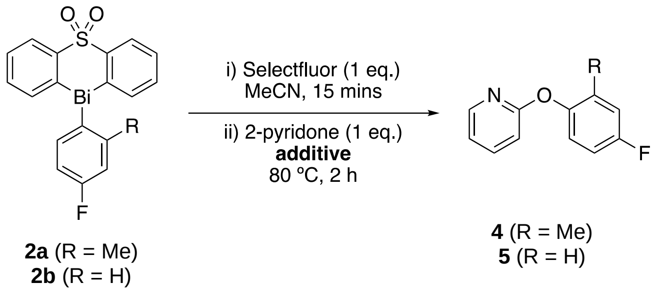

2a (R = Me)
2b (R = H)

4 (R = Me)
5 (R = H)

Entry	Additive	5 (%)	4 (%)
1	none	30	72
2	DBU (1 eq.)	2	5
3	*t*BuOK (1 eq.)	7	35
4	BTMG (1 eq.)	2	4
5	$NaHCO_3$ (1 eq.)	25	60
6	K_2CO_3 (1 eq.)	12	7
7	NaOH (1 eq.)	24	62
8	NaOBz (1 eq.)	56	89
10	BzOH (1 eq.)	76	94
11	NaOBz (1 eq.) & BzOH (1 eq.)	65	92

Table S2. Additive screen. Yields determined by ^{19}F NMR spectroscopy against an internal standard. Mass balance is composed of unreacted pyridone and arylbismacycle / arene (from formal protodebismuthation).

Procedure for R = H: A stock solution of aryl bismacycle **2b** and 4,4′-bis(trifluoromethyl)-1,1′-biphenyl (internal standard for ^{19}F NMR spectroscopy) in 1:1 MeCN/CD_3CN (0.04 M) was prepared. An aliquot was taken and analysed by quantitative ^{19}F NMR spectroscopy. Selectfluor (7.09 mg, 0.020 mmol) was added to an aliquot of the stock solution (0.5 mL, 0.020 mmol scale) and mixed for 15 mins before a solution of 2-pyridone (1.90 mg, 0.020 mmol) in MeCN (0.1 mL) was added followed by **additive** and heated at 80 °C for 2 h before analysing by quantitative ^{19}F NMR spectroscopy.

Procedure for R = Me: A stock solution of aryl bismacycle **2a** (1 eq.), Selectfluor (1 eq.) and 4,4′-bis(trifluoromethyl)-1,1′-biphenyl (internal standard for ^{19}F NMR spectroscopy) in 1:1 MeCN/CD_3CN (0.04 M) was stirred for 15 mins. An aliquot was taken and analysed by quantitative ^{19}F NMR spectroscopy. A solution of 2-pyridone (1.90 mg, 0.020 mmol) in MeCN (0.1 mL) was added to an aliquot of the stock solution (0.5 mL, 0.020 mmol scale) followed by **additive** and heated at 80 °C for 2 h before analysing by quantitative ^{19}F NMR spectroscopy.

Commentary: Use of a variety of bases (entries 2-7) in the reaction all proved detrimental and produced poorer yields compared to using no additive (entry 1). Use of NaOBz as an additive afforded improved yields (entry 8) and BzOH proved to be the best additive yielding excellent yields for both the *ortho*- and non-*ortho*-substituted examples (entry 10). Using NaOBz alongside BzOH degraded the yield slightly (entry 11).

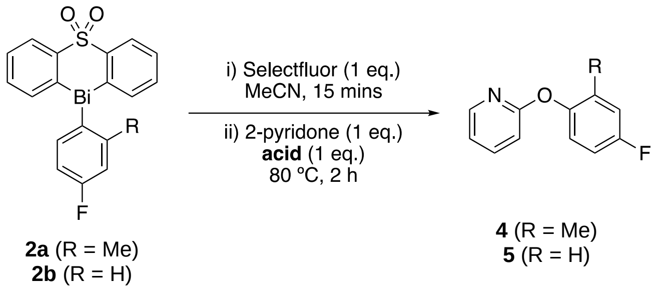

Entry	Additive	5 (%)	4 (%)
1	BzOH	76	94
2	Acetic acid	59	92
3	TFA	55	90
4	Pivalic acid	71	92
5	4-Methoxybenzoic acid	66	91
6	4-(Trifluoromethyl)benzoic acid	72	93
7	2,6-Dichlorobenzoic acid	74	94

Table S3. Acid screen. Yields determined by ^{19}F NMR spectroscopy against an internal standard. Mass balance is composed of unreacted pyridone and arylbismacycle (from Bi(V) reduction) / arene (from formal protodebismuthation) / arylbenzoate (from C-O ligand coupling).

Procedure for R = H: A stock solution of aryl bismacycle **2b** and 4,4′-bis(trifluoromethyl)-1,1′-biphenyl (internal standard for ^{19}F NMR spectroscopy) in 1:1 MeCN/CD_3CN (0.04 M) was prepared. An aliquot was taken and analysed by quantitative ^{19}F NMR spectroscopy. Selectfluor (7.09 mg, 0.020 mmol) was added to an aliquot of the stock solution (0.5 mL, 0.020 mmol scale) and mixed for 15 mins before a solution of 2-pyridone (1.90 mg, 0.020 mmol) in MeCN (0.1 mL) was added followed by **acid** (0.020 mmol) and heated at 80 °C for 2 h before analysing by quantitative ^{19}F NMR spectroscopy.

Procedure for R = Me: A stock solution of aryl bismacycle **2a** (1 eq.), Selectfluor (1 eq.) and 4,4′-bis(trifluoromethyl)-1,1′-biphenyl (internal standard for ^{19}F NMR spectroscopy) in 1:1 MeCN/CD_3CN (0.04 M) was stirred for 15 mins. An aliquot was taken and analysed by

quantitative ^{19}F NMR spectroscopy. A solution of 2-pyridone (1.90 mg, 0.020 mmol) in MeCN (0.1 mL) was added to an aliquot of the stock solution (0.5 mL, 0.020 mmol scale) followed by **acid** (0.020 mmol) and heated at 80 °C for 2 h before analysing by quantitative ^{19}F NMR spectroscopy.

Commentary: All acids screened gave comparable yields for the *ortho*-substituted example (entries 1-4, R = Me), while changing the electronics and sterics of the benzoic acid (entries 5-7, R = Me) had no noticeable effect. Benzoic acid remained the best acid for the non-*ortho*-substituted example (entries 1-7, R = H).

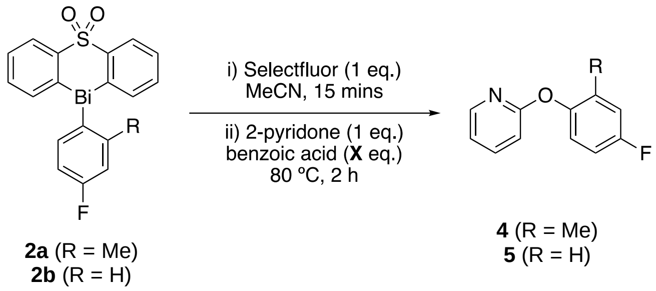

Entry	X	5 (%)	4 (%)
1	1.0	76	94
2	1.5	75	93
3	2.0	76	93

Table S4. Optimisation of benzoic acid stoichiometry. Yields determined by ^{19}F NMR spectroscopy against an internal standard. Mass balance is composed of unreacted pyridone and arylbismacycle / arene (from formal protodebismuthation).

Procedure for R = H: A stock solution of aryl bismacycle **2b** and 4,4′-bis(trifluoromethyl)-1,1′-biphenyl (internal standard for ^{19}F NMR spectroscopy) in 1:1 MeCN/CD_3CN (0.04 M) was prepared. An aliquot was taken and analysed by quantitative ^{19}F NMR spectroscopy. Selectfluor (7.09 mg, 0.020 mmol) was added to an aliquot of the stock solution (0.5 mL, 0.020 mmol scale) and mixed for 15 mins before a solution of 2-pyridone (1.90 mg, 0.020 mmol) in MeCN (0.1 mL) was added followed by benzoic acid (**X** eq.) and heated at 80 °C for 2 h before analysing by quantitative ^{19}F NMR spectroscopy.

Procedure for R = Me: A stock solution of aryl bismacycle **2a** (1 eq.), Selectfluor (1 eq.) and 4,4′-bis(trifluoromethyl)-1,1′-biphenyl (internal standard for ^{19}F NMR spectroscopy) in 1:1 MeCN/CD_3CN (0.04 M) was stirred for 15 mins. An aliquot was taken and analysed by quantitative ^{19}F NMR spectroscopy. A solution of 2-pyridone (1.90 mg, 0.020 mmol) in MeCN (0.1 mL) was added to an aliquot of the stock solution (0.5 mL, 0.020 mmol scale) followed by benzoic acid (**X** eq.) and heated at 80 °C for 2 h before analysing by quantitative ^{19}F NMR spectroscopy.

Commentary: Increasing the stoichiometry of benzoic acid gives no appreciable increase in yield for both non-*ortho*- and *ortho*-substituted examples.

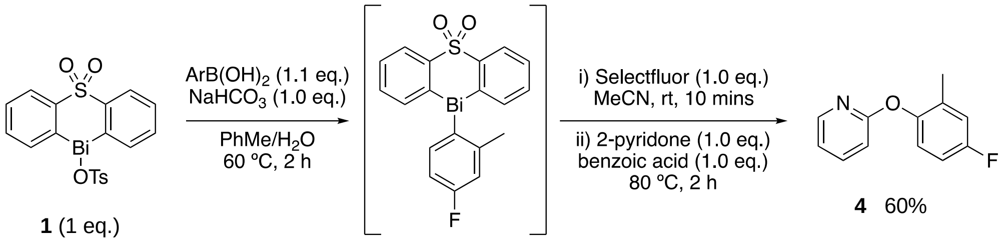

Figure S2. One-pot telescoped procedure.

Procedure: A suspension of bismacycle tosylate **1** (298 mg, 0.500 mmol), 4-fluoro-2-methylphenylboronic acid (84.7 mg, 0.500 mmol), $NaHCO_3$ (41.0 mg, 0.500 mmol) and 4,4′-bis(trifluoromethyl)-1,1′-biphenyl (internal standard for ^{19}F NMR spectroscopy) in toluene (5 mL) and water (100 μL) was heated at 60 °C for 2 h then cooled to room temperature and concentrated to dryness. Selectfluor (177 mg, 0.500 mmol) was added, followed by MeCN (5 mL). The reaction mixture was stirred at room temperature for 10 mins, then 2-pyridone (47.6 mg, 0.500 mmol) and benzoic acid (61.1 mg, 0.500 mmol) were then added and the reaction mixture was heated at 80 °C for 2 h before analysing by quantitative ^{19}F NMR spectroscopy.

Commentary: Telescoping the transmetallation step into the bismuth mediated arylation, with just a solvent swap between the two steps, resulted in a significantly degraded yield in comparison to the reaction from isolated aryl bismacycle. Alongside only 60% product, 32% 3-fluorotoluene and 17% 4-fluoro-2-methylphenyl tosylate were observed.

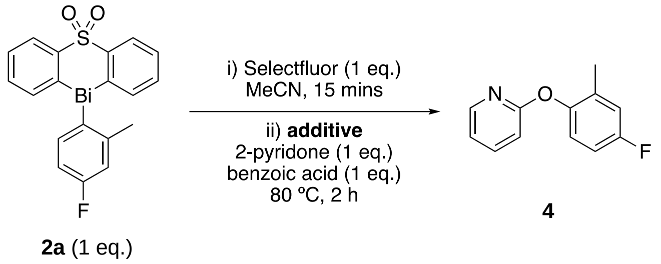

Entry	Additive	4 (%)
1	None	94
2	4-Fluoro-2-methylphenylboronic acid (0.1 eq.)	91
3	NaOTs (1.0 eq.)	80
4	$NaHCO_3$ (1.0 eq.)	93
5	$B(OH)_3$ (1.0 eq.)	79
6	Water (1 vol%)	92
7	Toluene (5 vol%)	95
8	NaOTs (1.0 eq.) & $B(OH)_3$ (1.0 eq.)	65

Table S5. Impact of transmetallation components on the bismuth mediated arylation step. Yields determined by ^{19}F NMR spectroscopy against an internal standard. Mass balance is composed of unreacted pyridone and arylbismacycle (from Bi(V) reduction) / arene (from formal protodebismuthation) / arylbenzoate (from C-O ligand coupling), unless noted otherwise in the following *commentary*.

Procedure: A stock solution of aryl bismacycle **2a** (1 eq.), Selectfluor (1 eq.) and 4,4′-bis(trifluoromethyl)-1,1′-biphenyl (internal standard for ^{19}F NMR spectroscopy) in MeCN (0.04 M) was stirred for 15 mins. An aliquot was taken and analysed by quantitative ^{19}F NMR spectroscopy. The **additive** was added to an aliquot of the stock solution (0.5 mL, 0.020 mmol scale) followed by a solution of 2-pyridone (1.90 mg, 0.020 mmol) in MeCN (0.1 mL) and benzoic acid (2.44 mg, 0.020 mmol). The resulting mixture was heated at 80 °C for 2 h before analysing by quantitative ^{19}F NMR spectroscopy.

Commentary: Residual boronic acid, $NaHCO_3$, toluene and water had little to no impact on the yield (entries 2, 4, 6 & 7). The presence of NaOTs resulted in a lower yield, with the formation of 4-fluoro-2-methylphenyl tosylate observed (entry 3). The presence of $B(OH)_3$ also degraded the yield with higher levels of 3-fluorotoluene observed (entry 5). Carrying out the reaction in the presence of both NaOTs and $B(OH)_3$ (entry 8) afforded a similar yield to the telescoped reaction (Figure S2). As NaOTs and $B(OH)_3$ are unavoidable co-products of the transmetallation, a 2M aq. NaOH wash was introduced after the transmetallation step to remove these undesirable species (Figure S3). The wash can be performed in the reaction flask and avoids the need to fully isolate the aryl bismacycle while still affording an excellent yield of the arylated pyridone.

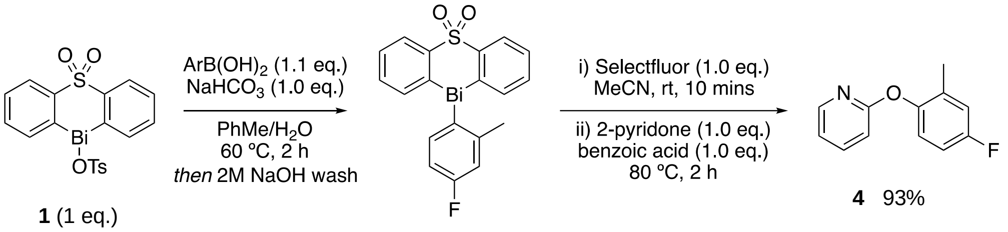

Figure S3. Telescoped procedure with incorporation of 2M aq. NaOH wash.

Procedure: A suspension of bismacycle tosylate **1** (477 mg, 0.800 mmol), 4-fluoro-2-methylphenylboronic acid (135 mg, 0.880 mmol) and $NaHCO_3$ (67.2 mg, 0.800 mmol) in toluene (8 mL) and water (160 μL) was heated at 60 °C for 2 h then cooled to room temperature. EtOAc (5 mL) and 2M aq. NaOH (5 mL) were added to the reaction mixture and the biphasic system was stirred vigorously for 5 mins then allowed to settle. The organic layer was removed by syringe and filtered through a pad of $MgSO_4$ into a 50 mL round-bottom flask. The aqueous portion was extracted twice more with EtOAc (2 × 5 mL) and the combined organic portions were concentrated to dryness. Selectfluor (283 mg, 0.800 mmol) was added followed by MeCN (8 mL) and the reaction mixture was stirred at room temperature for 10 mins. 2-Pyridone (76.1 mg, 0.800 mmol) and benzoic acid (97.7 mg, 0.800 mmol) were then added and the reaction mixture was heated at 80 °C for 2 h. After cooling to room temperature, benzotrifluoride (internal standard for ^{19}F NMR spectroscopy) was added before analysing by quantitative ^{19}F NMR spectroscopy.

ii. Bismacycle Ligand Screen

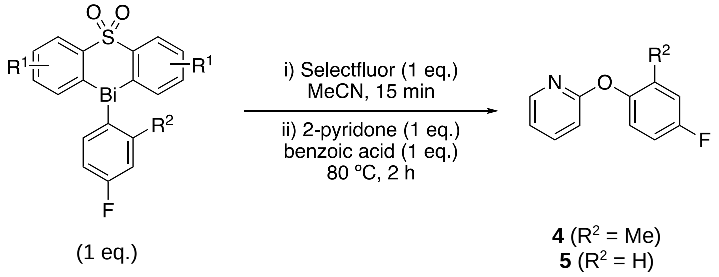

Entry	R^1		5 (%)	4 (%)
1	H	**2a/b**	76	94
2	*m*-Me	**S3a/b**	64	86
3	*m*-OMe	**S4a/b**	65	88
4	*m*-F	**S5a/b**	77	94
5	*m*-CF_3	**S2a/b**	83	94
6	*p*-CF_3	**S6a/b**	58	94
7	*o*-OMe	**S7a/b**	13	n/a
8	*o*-Cl	**S8a/b**	n/a	n/a

Table S6. Ligand screening. *o*-, *m*- and *p*- are relative to bismuth. Yields determined by ^{19}F NMR spectroscopy against an internal standard. Mass balance is composed of unreacted pyridone and arylbismacycle (from Bi(V) reduction) / arene (from formal protodebismuthation) / arylbenzoate (from C-O ligand coupling).

Procedure for entry 1, R^2 = Me: A stock solution of the aryl bismacycle (1 eq.), Selectfluor (1 eq.) and 4,4′-bis(trifluoromethyl)-1,1′-biphenyl (internal standard for ^{19}F NMR spectroscopy) in 1:1 MeCN/CD_3CN (0.04 M) was stirred for 15 mins. An aliquot was taken and analysed by quantitative ^{19}F NMR spectroscopy. A solution of 2-pyridone (1.90 mg, 0.020 mmol) in MeCN (0.1 mL) was added to an aliquot of the stock solution (0.5 mL, 0.020 mmol scale) followed by benzoic acid (2.44 mg, 0.020 mmol) and heated at 80 °C for 2 h before analysing by quantitative ^{19}F NMR spectroscopy.

Procedure for other entries: A stock solution of the aryl bismacycle and 4,4′-bis(trifluoromethyl)-1,1′-biphenyl (internal standard for ^{19}F NMR spectroscopy) in 1:1 MeCN/CD_3CN (0.04 M) was prepared. An aliquot was taken and analysed by quantitative ^{19}F NMR spectroscopy. Selectfluor (7.09 mg, 0.020 mmol) was added to an aliquot of the stock solution (0.5 mL, 0.020 mmol scale) and mixed for 15 mins before a solution of 2-pyridone (1.90 mg, 0.020 mmol) in MeCN (0.1 mL) was added followed by benzoic acid (2.44 mg, 0.020 mmol) and heated at 80 °C for 2 h before analysing by quantitative ^{19}F NMR spectroscopy.

Commentary: More electron rich ligands resulted in poorer yields (entries 2 & 3) while more electron poor ligands generally afforded comparable yields (entries 4-6). For R^2 = Me, no ligands yielded any gains, however for R^2 = H the *m*-CF_3 substituted bismacycle achieved an improved yield (entry 5). Bismacycles possessing *ortho*-substituents relative to bismuth proved poor candidates (entries 7 & 8). The *o*-OMe ligand gave a poor yield for R^2 = H and the 4-fluoro-2-methylphenyl bismacycle (R^2 = Me) was not synthesised due to instability of the bismacycle towards TsOH (entry 7). Results were not obtained for the *o*-Cl bismacycle as it exhibited extremely poor solubility which meant complete oxidation with Selectfluor could not be achieved (entry 8).

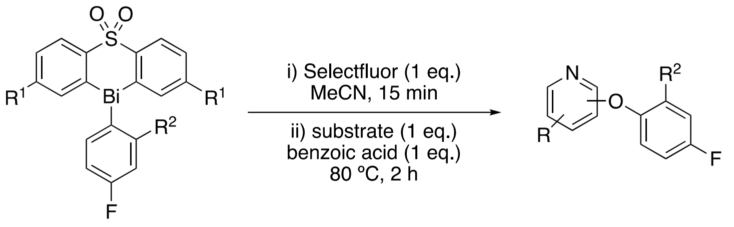

2a (R^1 = H; R^2 = Me)
2b (R^1 = H; R^2 = H)
S2a (R^1 = CF_3; R^2 = Me)
S2b (R^1 = CF_3; R^2 = H)

Entry	Product	R^1	Yield (%)
1	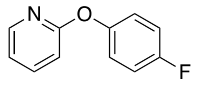	H	76
2		CF_3	83
3	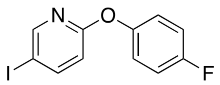	H	49
4		CF_3	56
5	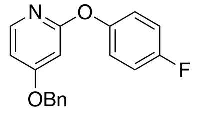	H	60
6		CF_3	51
7	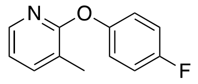	H	76
8		CF_3	74
9	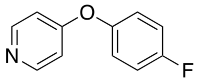	H	79
10		CF_3	76
11	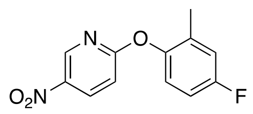	H	35
12		CF_3	38

Table S7. Comparison of CF_3 substituted bismacycle *vs* unsubstituted bismacycle. Yields determined by ^{19}F NMR spectroscopy against an internal standard. Mass balance is composed of unreacted pyridone and arylbismacycle (from Bi(V) reduction) / arene (from formal protodebismuthation) / arylbenzoate (from C-O ligand coupling).

Procedure: A stock solution of the aryl bismacycle and 4,4′-bis(trifluoromethyl)-1,1′-biphenyl (internal standard for ^{19}F NMR spectroscopy) in MeCN (0.03 M) was prepared. An aliquot was taken and analysed by quantitative ^{19}F NMR spectroscopy. Selectfluor (7.09 mg, 0.020 mmol)

was added to an aliquot of the stock solution (0.6 mL, 0.020 mmol scale) and mixed for 15 mins before the pyridone substrate (0.020 mmol) and benzoic acid (2.44 mg, 0.020 mmol) were added. The mixture was heated at 80 °C for 2 h before analysing by quantitative ^{19}F NMR spectroscopy.

Commentary: While initial results indicated that the CF_3 substituted bismacycle may afford improved yields, particularly for non-*ortho* substituted examples (Table S6), further testing with more substrates revealed inconsistent results. While the CF_3 substituted bismacycle enhanced the yields in two examples (entries 1-4), it afforded either worse or comparable yields for a number of other substrates when compared to the unsubstituted bismacycle (entries 5-12). Given the inconsistent results and extra steps required to synthesise the CF_3 substituted bismacycle, it was decided to proceed with the unsubstituted bismacycle which can be synthesised in fewer steps and isolated more easily and in higher yields.

5. General Procedure for Pyridone Arylation

General Procedure 3 (GP3) – Pyridone Arylation

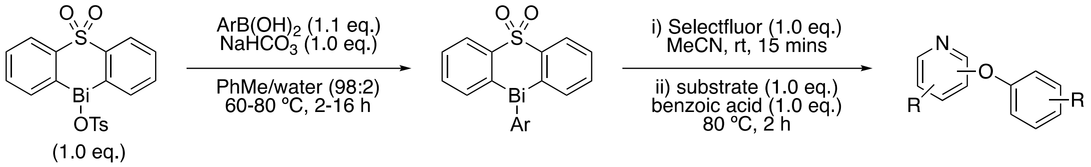

A suspension of bismacycle tosylate **1** (1.0 eq.), aryl boronic acid (1.1 eq.) and $NaHCO_3$ (1.0 eq.) in toluene (0.1 M) and water (2 vol%) was heated at **X** °C for **Y** h (until determined complete by ^{1}H NMR spectroscopy) then cooled to room temperature. EtOAc (5 mL) and 2M aq. NaOH (5 mL) were added to the reaction mixture and the biphasic system was stirred vigorously for 5 mins then allowed to separate.* The organic layer was removed by syringe and filtered through a pad of $MgSO_4$ into a 50 mL round-bottom flask. The aqueous portion was extracted with EtOAc (2 × 5 mL) and the combined organic portions were concentrated to dryness.

Selectfluor (1.0 eq.) was added to the round-bottom flask, followed by MeCN (0.1 M). The reaction mixture was stirred at room temperature for 15 mins, then the pyridone substrate (1.0 eq.) and benzoic acid (1.0 eq.) were added. The reaction mixture was heated at 80 °C for 2 h before cooling to room temperature. *p*-Toluenesulfonic acid monohydrate (2 eq.) was added and the mixture was stirred for 1 h at room temperature before cooling to 0 °C. The mixture was filtered into a separatory funnel and the solid washed with ice-cold MeCN (5 mL) and Et_2O (5 mL) to afford the recovered bismacycle tosylate **1**.[†]

Workup A: The filtrate was diluted with Et_2O (50 mL), washed with 2M aq. NaOH (3 × 10 mL) and extracted with 4 M HCl (3 × 15 mL). The combined acidic aqueous portions were basified with Na_2CO_3 and then extracted with EtOAc (3 × 50 mL). The combined organic portions were dried over $MgSO_4$ and concentrated to dryness.

Workup B: The filtrate was diluted with Et_2O (50 mL), washed with 2M aq. NaOH (3 × 10 mL), dried over $MgSO_4$ and concentrated to dryness. The crude material was purified by silica gel column chromatography.

***** If the phases are not clear and an emulsion/solid persists it indicates poor solubility of the aryl bismacycle; in these cases an alternative workup is employed: the mixture was transferred

to a separating funnel, diluted with EtOAc (~100 mL) until everything was solubilised, washed with 2 M aq. NaOH (3 × 5 mL), dried over $MgSO_4$ and concentrated to dryness in a 50 mL round-bottom flask.

† Reaction affording 2-(4-fluoro-2-methylphenoxy)pyridine **4**, carried out on a 0.800 mmol scale, gave the recovered bismacycle tosylate **1** (381 mg, 0.639 mmol, 80%) as a colourless solid in >95% purity.

6. Characterisation Data for Arylation Products

i. Confirmation of Regioselectivity for O-Arylation

At no point during this study did we observe the products of N-arylation. The absence of N-aryl isomers from crude reaction mixtures is apparent from NMR spectroscopic analysis, which was performed on all reactions prior to work-up. Not only are the ^{1}H and ^{19}F NMR spectra highly distinctive for each of the different isomers (see Table S8 for a comparison of spectroscopic data for representative O-aryl and N-aryl isomers), but there are also no signals unaccounted for in the spectra of the crude mixtures. Illustrative spectra are presented below for the synthesis of **4**, **5** and **28** (Figures S4-S6), which cover *ortho*-substituted/unsubstituted boronic acids and both 2- and 4-pyridone substrates; as shown, all species observed in the NMR spectra of the crude reaction mixturess can be attributed to known compounds (the desired aryloxypyridine, or the side-products arylbismacycle(III), arene or aryl benzoate).

2-(4-Fluorophenoxy)pyridine	1-(4-Fluorophenyl)pyridin-2(1*H*)-one
Manuscript compound **5**	Literature compound; data from:[4]
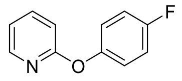	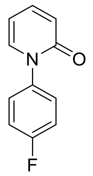
**^{1}H NMR (400 MHz, $CDCl_3$):** δ 8.18 (ddd, *J* = 5.0, 2.0, 0.8 Hz, 1H), 7.69 (ddd, *J* = 8.3, 7.2, 2.0 Hz, 1H), 7.15 – 7.04 (m, 4H), 6.99 (ddd, *J* = 7.2, 5.0, 0.9 Hz, 1H), 6.91 (app. dt, *J* = 8.3, 0.9 Hz, 1H).	**^{1}H NMR (700 MHz, $CDCl_3$):** δ 7.41−7.39 (m, 1H), 7.37−7.35 (m, 2H), 7.31 (dd, *J* = 7.0, 1.4 Hz, 1H), 7.18−7.16 (m, 2H), 6.64 (d, *J* = 9.1 Hz, 1H), 6.25 (t, *J* = 7.0 Hz, 1H).
**^{13}C{^{1}H} NMR (101 MHz, $CDCl_3$):** δ 163.8, 159.7 (d, *J* = 242.8 Hz), 149.9 (d, *J* = 2.8 Hz), 147.7, 139.6, 122.9 (d, *J* = 8.4 Hz), 118.6, 116.4 (d, *J* = 23.3 Hz), 111.5.	**^{13}C{^{1}H} NMR (175 MHz, $CDCl_3$):** δ 162.9 (d, $J_{C–F}$ = 318.5 Hz), 161.7, 140.3, 138.1, 137.1 (d, $J_{C–F}$ = 12.6 Hz), 128.6 (d, $J_{C–F}$ = 35.0 Hz), 122.1, 116.5 (d, $J_{C–F}$ = 91.7 Hz), 106.3.
**^{19}F NMR (376 MHz, $CDCl_3$):** δ -118.53 (tt, *J* = 7.7, 5.0 Hz).	**^{19}F NMR (376 MHz, $CDCl_3$):** δ −112.7.
**ν_{max} (ATR)/cm^{-1}:** 3075, 3056, 3016, 1590, 1573, 1501, 1465, 1426, 1287, 1266, 1248, 1224, 1190, 1143, 1089, 991, 884, 851, 818, 777, 757, 735, 528, 499.	**ν_{max} (KBr)/cm^{-1}:** 3038, 1653, 1582, 1136, 762.
**HRMS** calcd. for $C_{11}H_8FNO+H^+$: 190.0663 $[M+H]^+$; found (ESI^+) 190.0670.	**HRMS** calcd. for $C_{11}H_8FNO+H^+$: 190.0663 $[M+H]^+$; found (ESI^+) 190.0664.
**m.p./°C:** 53-54.	**m.p./°C:** 140−142.

Table S8. Characterisation data for aryloxypyridine **5** and the isomeric N-aryl pyridone.[4]

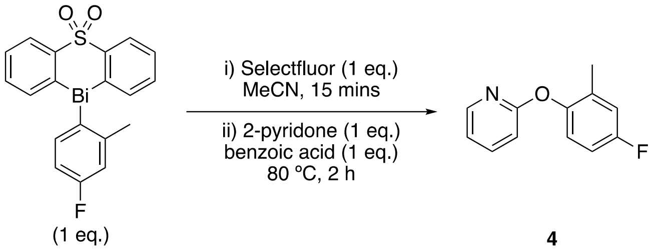

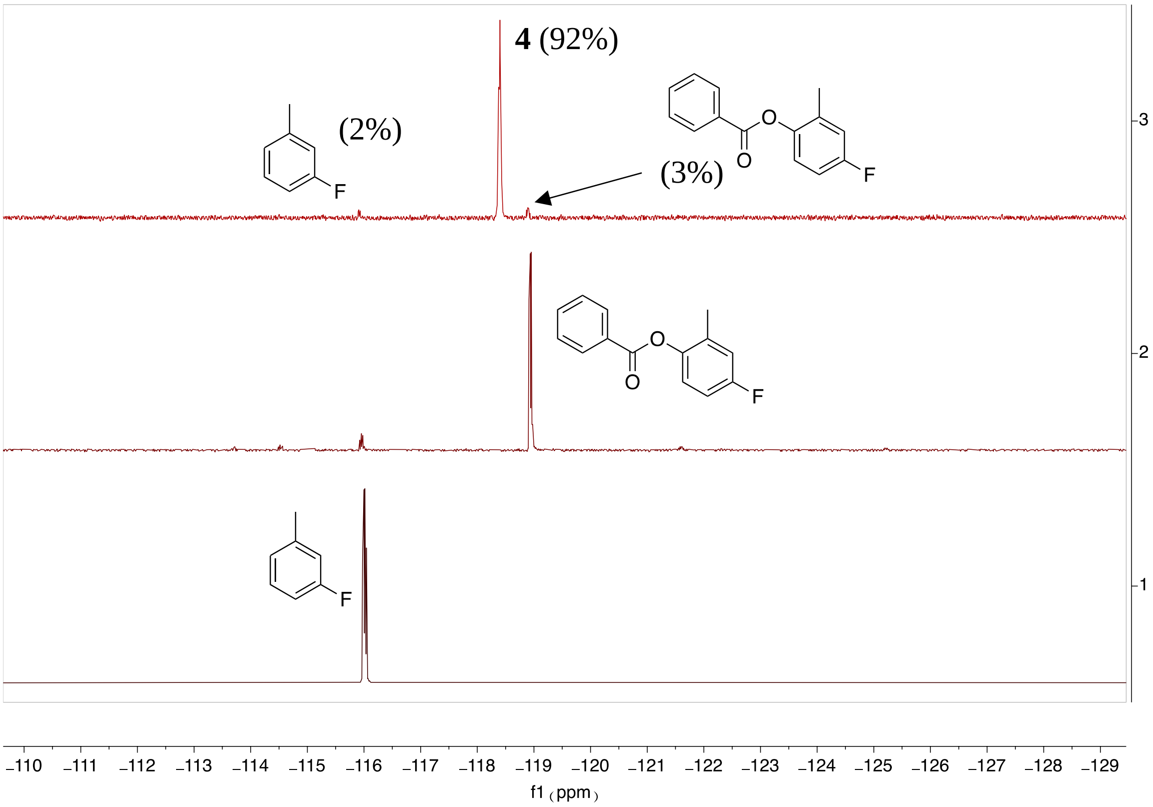

Figure S4. ^{19}F NMR spectrum (376 MHz) of the crude reaction mixture from the synthesis of **4** (top; prepared by dilution of a reaction aliquot into *d*3-MeCN), and comparison to known side-products.

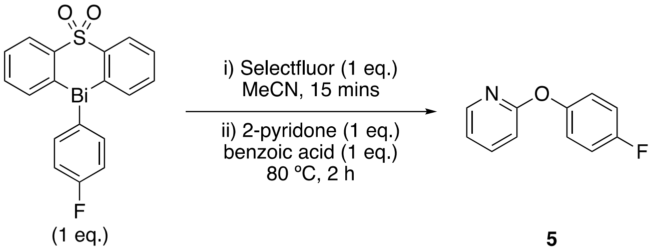

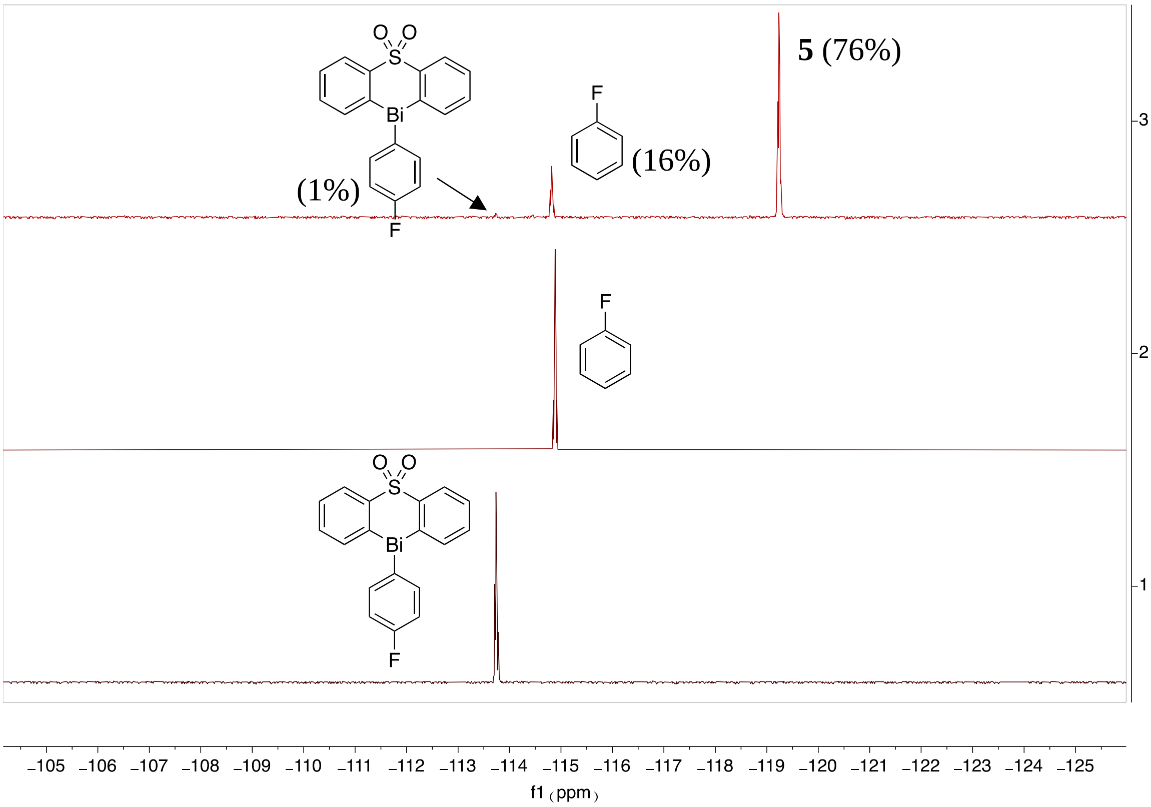

Figure S5. ^{19}F NMR spectrum (376 MHz) of the crude reaction mixture from the synthesis of **5** (top; prepared by dilution of a reaction aliquot into *d*3-MeCN), and comparison to known side-products.

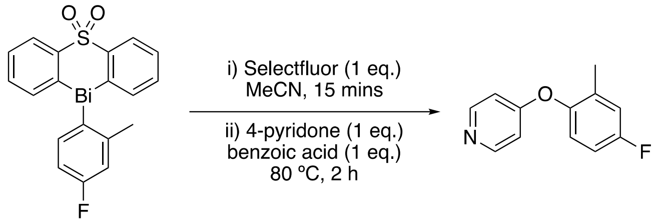

(1 eq.) **28**

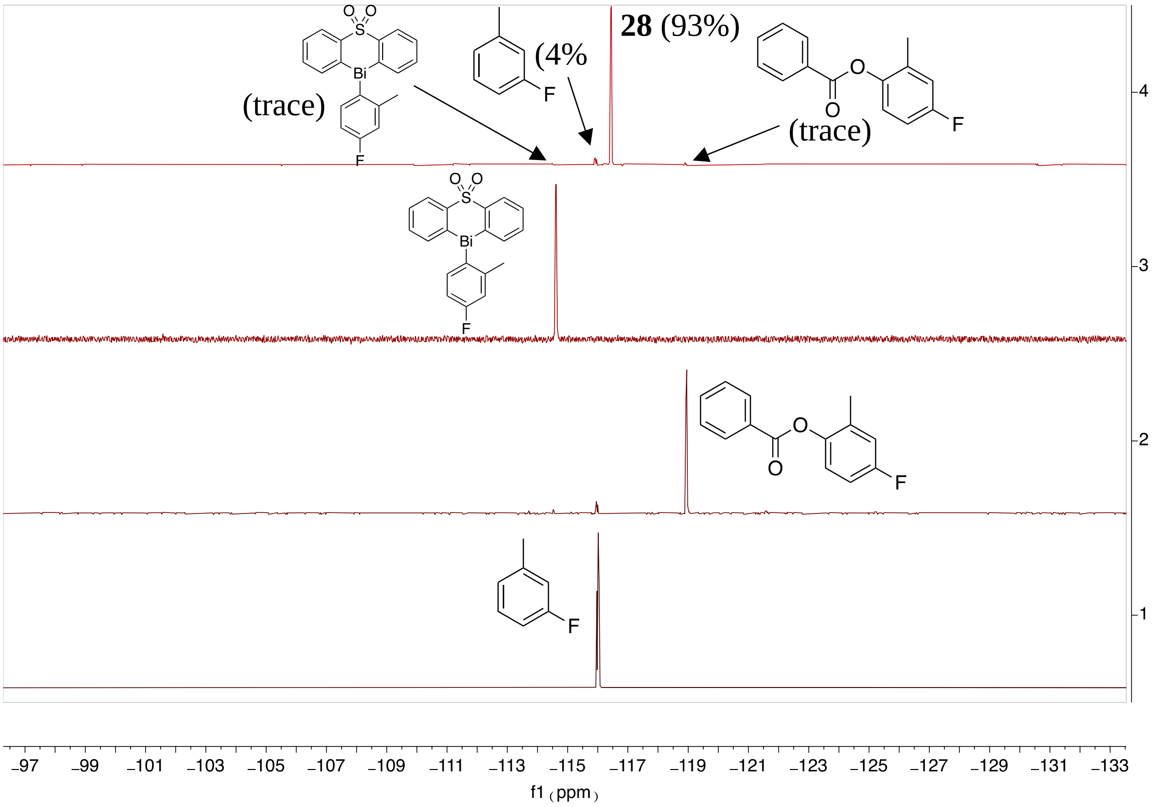

Figure S6. ^{19}F NMR spectrum (376 MHz) of the crude reaction mixture from the synthesis of **28** (top; prepared by dilution of a reaction aliquot into *d*$_3$-MeCN), and comparison to known side-products.

ii. 2-Pyridone Substrates

2-(4-Fluoro-2-methylphenoxy)pyridine (4)

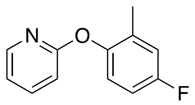

Synthesised according to **GP3** (**X** = 60, **Y** = 2). Using 4-fluoro-2-methylphenylboronic acid (135 mg, 0.880 mmol), 2-pyridone (76.1 mg, 0.800 mmol) and **workup A** afforded the title compound (144 mg, 0.709 mmol, 89%) as an oil which solidified on standing to an off-white solid.

^{1}H NMR (400 MHz, $CDCl_3$): δ 8.16 (dd, *J* = 5.2, 1.9 Hz, 1H), 7.68 (ddd, *J* = 8.8, 7.1, 1.9 Hz, 1H), 7.07 – 6.84 (m, 5H), 2.16 (s, 3H).

^{13}C{^{1}H} NMR (126 MHz, $CDCl_3$): δ 163.7, 159.9 (d, *J* = 242.9 Hz), 148.0 (d, *J* = 2.7 Hz), 147.8, 139.7, 133.0 (d, *J* = 8.2 Hz), 123.3 (d, *J* = 9.0 Hz), 118.3, 117.9 (d, *J* = 23.0 Hz), 113.8 (d, *J* = 23.0 Hz), 110.8, 16.7.

^{19}F NMR (377 MHz, $CDCl_3$): δ -118.40 (ddd, *J* = 9.0, 8.0, 5.1 Hz).

ν_{max} (ATR)/cm^{-1}: 3058, 3015, 2925, 1595, 1573, 1494, 1467, 1428, 1380, 1287, 1260, 1238, 1185, 1143, 991, 870, 823, 778, 736.

HRMS calcd. for $C_{12}H_{10}FNO+H^+$: 204.0819 [M+H]$^+$; found (ESI$^+$) 204.0825.

m.p./°C: 57-58.

2-(4-Fluoro-2-methylphenoxy)-3-methylpyridine (6)

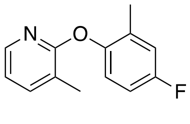

Synthesised according to **GP3** (**X** = 60, **Y** = 2). Using 4-fluoro-2-methylphenylboronic acid (135 mg, 0.880 mmol), 3-methylpyridin-2-ol (87.3 mg, 0.800 mmol) and **workup A** afforded the title compound (157 mg, 0.723 mmol, 90%) as a yellow oil.

^{1}H NMR (400 MHz, $CDCl_3$): δ 7.97 (d, *J* = 2.4 Hz, 1H), 7.49 (dd, *J* = 8.4, 2.4 Hz, 1H), 7.02 – 6.94 (m, 2H), 6.90 (app. td, *J* = 8.4, 3.1 Hz, 1H), 6.77 (d, *J* = 8.4 Hz, 1H), 2.26 (s, 3H), 2.16 (s, 3H).

^{13}C{^{1}H} NMR (101 MHz, $CDCl_3$): δ 162.0, 159.7 (d, *J* = 242.7 Hz), 148.4 (d, *J* = 2.7 Hz), 147.3, 140.6, 132.9 (d, *J* = 8.2 Hz), 127.5, 123.0 (d, *J* = 8.8 Hz), 117.8 (d, *J* = 22.8 Hz), 113.7 (d, *J* = 23.1 Hz), 110.2, 17.6, 16.7.

^{19}F NMR (376 MHz, $CDCl_3$): δ -118.67 (app. td, *J* = 8.5, 5.1 Hz).

ν_{max} (ATR)/cm^{-1}: 2953, 1605, 1579, 1497, 1474, 1416, 1378, 1279, 1262, 1251, 1238, 1219, 1185, 1145, 1126, 876, 822.

HRMS calcd. for $C_{13}H_{12}FNO+H^+$: 218.0976 $[M+H]^+$; found (ESI^+) 218.0980.

2-(4-Fluoro-2-methylphenoxy)-6-methylpyridine (7)

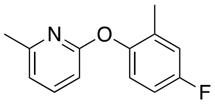

Synthesised according to **GP3** (**X** = 60, **Y** = 2). Using 4-fluoro-2-methylphenylboronic acid (135 mg, 0.880 mmol), 6-methylpyridin-2-ol (87.3 mg, 0.800 mmol) and **workup A** afforded the title compound (154 mg, 0.709 mmol, 89%) as a yellow oil.

^{1}H NMR (400 MHz, $CDCl_3$): δ 7.52 (dd, *J* = 8.2, 7.4 Hz, 1H), 7.03 – 6.94 (m, 2H), 6.89 (app. td, *J* = 8.4, 3.1 Hz, 1H), 6.84 (d, *J* = 7.3 Hz, 1H), 6.43 (d, *J* = 8.2 Hz, 1H), 2.45 (s, 3H), 2.18 (s, 3H).

^{13}C{^{1}H} NMR (101 MHz, $CDCl_3$): δ 163.2, 159.7 (d, *J* = 242.7 Hz), 157.7, 148.4 (d, *J* = 2.8 Hz), 139.9, 132.9 (d, *J* = 8.1 Hz), 122.9 (d, *J* = 8.9 Hz), 117.8 (d, *J* = 23.0 Hz), 117.7, 113.7 (d, *J* = 23.1 Hz), 106.3, 24.2, 16.7 (d, *J* = 1.4 Hz).

^{19}F NMR (377 MHz, $CDCl_3$): δ -118.58 – -118.71 (m).

ν_{max} (ATR)/cm^{-1}: 2925, 1597, 1576, 1494, 1446, 1414, 1294, 1269, 1256, 1226, 1185, 1149, 989, 940, 864, 822, 804, 788.

HRMS calcd. for $C_{13}H_{12}FNO+H^+$: 218.0976 $[M+H]^+$; found (ESI^+) 218.0985.

2-(4-Fluoro-2-methylphenoxy)-5-iodopyridine (8)

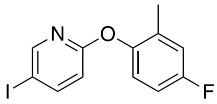

Synthesised according to **GP3** (**X** = 60, **Y** = 2). Using 4-fluoro-2-methylphenylboronic acid (135 mg, 0.880 mmol), 5-iodopyridin-2-ol (177 mg, 0.800 mmol) and **workup B** afforded, after purification by silica gel column chromatography (100% pentane), the title compound (212 mg, 0.644 mmol, 81%) as a colourless oil.

^{1}H NMR (400 MHz, $CDCl_3$): δ 8.32 (dd, *J* = 2.4, 0.7 Hz, 1H), 7.91 (dd, *J* = 8.6, 2.4 Hz, 1H), 7.05 – 6.95 (m, 2H), 6.92 (app. td, *J* = 8.3, 3.1 Hz, 1H), 6.74 (dd, *J* = 8.6, 0.7 Hz, 1H), 2.14 (s, 3H).
^{13}C{^{1}H} NMR (101 MHz, $CDCl_3$): δ 163.2, 160.0 (d, *J* = 243.4 Hz), 153.7, 147.7 (d, *J* = 2.8 Hz), 147.5, 133.0 (d, *J* = 8.3 Hz), 123.2 (d, *J* = 9.0 Hz), 117.9 (d, *J* = 22.8 Hz), 113.8 (d, *J* = 23.2 Hz), 113.1, 83.9, 16.6.
^{19}F NMR (376 MHz, $CDCl_3$): δ -117.77 (ddd, *J* = 8.9, 7.9, 5.1 Hz).
ν_{max} (ATR)/cm^{-1}: 3072, 3045, 2958, 2924, 1621, 1571, 1492, 1454, 1416, 1359, 1277, 1260, 1236, 1182, 1145, 1126, 1077, 998, 952, 870, 822, 778, 713, 567.
HRMS calcd. for $C_{12}H_9FINO+H^+$: 329.9786 $[M+H]^+$; found (ESI^+) 329.9791.

3-Bromo-2-(4-fluoro-2-methylphenoxy)pyridine (9)

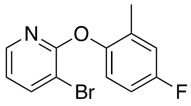

Synthesised according to **GP3** (**X** = 60, **Y** = 2). Using 4-fluoro-2-methylphenylboronic acid (135 mg, 0.880 mmol), 3-bromopyridin-2-ol (139 mg, 0.800 mmol) and **workup B** afforded, after purification by silica gel column chromatography (100% pentane), the title compound (160 mg, 0.567 mmol, 71%) as an oil which solidified on standing to a colourless solid.

^{1}H NMR (400 MHz, $CDCl_3$): δ 8.03 (dd, *J* = 4.8, 1.7 Hz, 1H), 7.93 (dd, *J* = 7.7, 1.7 Hz, 1H), 7.05 (dd, *J* = 8.8, 5.0 Hz, 1H), 7.02 – 6.90 (m, 2H), 6.87 (dd, *J* = 7.7, 4.8 Hz, 1H), 2.15 (s, 3H).

^{19}F NMR (376 MHz, $CDCl_3$): δ -117.93 (ddd, *J* = 9.0, 8.0, 5.0 Hz).

^{13}C{^{1}H} NMR (101 MHz, $CDCl_3$): δ 160.0 (d, *J* = 243.3 Hz), 159.6, 147.8 (d, *J* = 2.8 Hz), 146.1, 142.7, 132.9 (d, *J* = 8.3 Hz), 123.5 (d, *J* = 8.9 Hz), 119.5, 117.8 (d, *J* = 23.0 Hz), 113.7 (d, *J* = 23.3 Hz), 107.2, 16.6.

ν_{max} (ATR)/cm^{-1}: 3055, 2925, 1621, 1578, 1494, 1445, 1415, 1296, 1254, 1236, 1179, 1028, 872, 822, 788, 744, 534.

HRMS calcd. for $C_{12}H_9BrFNO+H^+$: 281.9924 $[M+H]^+$; found (ESI^+) 281.9931.

m.p./°C: 77-78.

2-Chloro-6-(4-fluoro-2-methylphenoxy)pyridine (10)

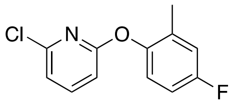

Synthesised according to **GP3** (**X** = 60, **Y** = 2). Using 4-fluoro-2-methylphenylboronic acid (135 mg, 0.880 mmol), 6-chloropyridin-2-ol (104 mg, 0.800 mmol) and **workup B** afforded, after purification by silica gel column chromatography (100% pentane), the title compound (77.0 mg, 0.324 mmol, 40%) as a colourless oil.

^{1}H NMR (400 MHz, $CDCl_3$): δ 7.60 (dd, *J* = 8.2, 7.6 Hz, 1H), 7.06 – 6.95 (m, 3H), 6.91 (app. td, *J* = 8.3, 3.1 Hz, 1H), 6.68 (dd, *J* = 8.1, 0.6 Hz, 1H), 2.16 (s, 3H).

^{13}C{^{1}H} NMR (101 MHz, $CDCl_3$): δ 163.2, 160.0 (d, *J* = 243.2 Hz), 149.4, 147.7 (d, *J* = 2.8 Hz), 141.6, 132.8 (d, *J* = 8.3 Hz), 123.0 (d, *J* = 8.9 Hz), 118.3, 117.9 (d, *J* = 23.0 Hz), 113.8 (d, *J* = 23.2 Hz), 108.3, 16.6 (d, *J* = 1.4 Hz).

^{19}F NMR (376 MHz, $CDCl_3$): δ -117.88 (ddd, *J* = 9.0, 7.9, 4.9 Hz).

ν_{max} (ATR)/cm^{-1}: 3084, 2926, 1582, 1567, 1493, 1428, 1288, 1260, 1184, 1160, 1145, 987, 914, 865, 822, 788, 724.

HRMS calcd. for $C_{12}H_9ClFNO+H^+$: 238.0429 $[M+H]^+$; found (ESI^+) 238.0436.

2-(4-Fluoro-2-methylphenoxy)-5-nitropyridine (11)

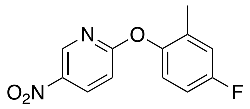

Synthesised according to **GP3** (**X** = 60, **Y** = 2). Using 4-fluoro-2-methylphenylboronic acid (135 mg, 0.880 mmol), 5-nitropyridin-2-ol (112 mg, 0.800 mmol) and **workup B** afforded, after purification by silica gel column chromatography (100% pentane), the title compound (61.1 mg, 0.246 mmol, 31%) as a colourless oil.

^{1}H NMR (400 MHz, $CDCl_3$): δ 9.02 (d, *J* = 2.8 Hz, 1H), 8.49 (dd, *J* = 9.1, 2.8 Hz, 1H), 7.12 – 6.91 (m, 4H), 2.14 (s, 3H).
^{13}C{^{1}H} NMR (101 MHz, $CDCl_3$): δ 166.7, 160.4 (d, *J* = 244.6 Hz), 147.1 (d, *J* = 2.9 Hz), 145.3, 140.5, 135.2, 132.8 (d, *J* = 8.4 Hz), 123.2 (d, *J* = 8.9 Hz), 118.2 (d, *J* = 23.0 Hz), 114.1 (d, *J* = 23.3 Hz), 111.0, 16.6.
^{19}F NMR (377 MHz, $CDCl_3$): δ -116.60 (ddd, *J* = 9.3, 7.7, 4.9 Hz).
ν_{max} (ATR)/cm^{-1}: 3100, 2960, 2970, 2858, 1603, 1579, 1520, 1494, 1464, 1391, 1348, 1302, 1262, 1245, 1178, 1113, 883, 840, 824, 764, 717.
HRMS calcd. for $C_{12}H_9FN_2O_3+H^+$: 249.0670 $[M+H]^+$; found (ESI^+) 249.0660.

4-(Benzyloxy)-2-(4-fluoro-2-methylphenoxy)pyridine (12)

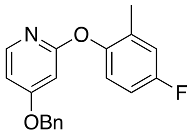

Synthesised according to **GP3** (**X** = 60, **Y** = 2). Using 4-fluoro-2-methylphenylboronic acid (135 mg, 0.880 mmol), 4-(benzyloxy)pyridin-2-ol (161 mg, 0.800 mmol) and **workup A** afforded the title compound (208 mg, 0.673 mmol, 84%) as a colourless solid.

^{1}H NMR (400 MHz, $CDCl_3$): δ 7.99 (d, *J* = 5.9 Hz, 1H), 7.46 – 7.32 (m, 5H), 7.04 – 6.94 (m, 2H), 6.90 (app. td, *J* = 8.3, 3.1 Hz, 1H), 6.62 (dd, *J* = 5.9, 2.2 Hz, 1H), 6.38 (d, *J* = 2.2 Hz, 1H), 5.10 (s, 2H), 2.14 (s, 3H).

$^{13}C\{^1H\}$ NMR (101 MHz, $CDCl_3$): δ 167.8, 165.5, 159.9 (d, *J* = 243.0 Hz), 148.5, 148.0 (d, *J* = 2.7 Hz), 135.6, 133.0 (d, *J* = 8.3 Hz), 128.9, 128.6, 127.7, 123.3 (d, *J* = 8.9 Hz), 117.9 (d, *J* = 22.9 Hz), 113.7 (d, *J* = 23.1 Hz), 107.2, 95.6, 70.3, 16.7.

^{19}F NMR (376 MHz, $CDCl_3$): δ -118.30 (app. td, *J* = 8.5, 5.1 Hz).

ν_{max} (ATR)/cm^{-1}: 3034, 2925, 1599, 1572, 1494, 1454, 1406, 1382, 1334, 1297, 1265, 1248, 1188, 1161, 1113, 1094, 1024, 947, 865, 827, 739, 697.

HRMS calcd. for $C_{19}H_{16}FNO_2+H^+$: 310.1238 $[M+H]^+$; found (ESI^+) 310.1240.

m.p./°C: 83-84.

2-(4-Fluoro-2-methylphenoxy)quinoline (13)

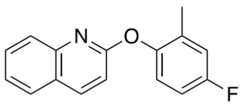

Synthesised according to **GP3** (**X** = 60, **Y** = 2). Using 4-fluoro-2-methylphenylboronic acid (135 mg, 0.880 mmol), quinolin-2-ol (116 mg, 0.800 mmol) and **workup B** afforded, after purification by silica gel column chromatography (100% pentane), the title compound (160 mg, 0.632 mmol, 79%) as an oil which solidified on standing to an off-white solid.

^1H NMR (400 MHz, $CDCl_3$): δ 8.12 (dd, *J* = 9.0, 0.7 Hz, 1H), 7.82 – 7.70 (m, 2H), 7.60 (ddd, *J* = 8.6, 6.9, 1.5 Hz, 1H), 7.41 (ddd, *J* = 8.0, 6.9, 1.3 Hz, 1H), 7.12 (dd, *J* = 8.8, 5.0 Hz, 1H), 7.06 (d, *J* = 8.8 Hz, 1H), 7.01 (dd, *J* = 9.0, 3.1 Hz, 1H), 6.95 (app. td, *J* = 8.3, 3.1 Hz, 1H), 2.19 (s, 3H).

$^{13}C\{^1H\}$ NMR (101 MHz, $CDCl_3$): δ 161.6, 159.9 (d, *J* = 242.7 Hz), 148.0 (d, *J* = 2.7 Hz), 146.6, 140.0, 133.0 (d, *J* = 8.3 Hz), 129.9, 128.0, 127.5, 125.7, 124.9, 123.4 (d, *J* = 8.8 Hz), 117.7 (d, *J* = 22.9 Hz), 113.5 (d, *J* = 23.1 Hz), 112.1, 16.8.

^{19}F NMR (376 MHz, $CDCl_3$): δ -118.55 (ddd, *J* = 9.0, 8.0, 5.0 Hz).

ν_{max} (ATR)/cm^{-1}: 3064, 2958, 2925, 1619, 1603, 1574, 1492, 1469, 1424, 1390, 1312, 1274, 1254, 1229, 1208, 1181, 1144, 1111, 976, 950, 918, 864, 821, 806, 780, 757, 585.

HRMS calcd. for $C_{16}H_{12}FNO+H^+$: 254.0976 $[M+H]^+$; found (ESI^+) 254.0980.

m.p./°C: 79-80.

1-(4-Fluoro-2-methylphenoxy)isoquinoline (14)

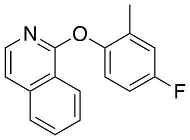

Synthesised according to **GP3** (**X** = 60, **Y** = 2). Using 4-fluoro-2-methylphenylboronic acid (135 mg, 0.880 mmol), isoquinolin-1-ol (116 mg, 0.800 mmol) and **workup B** afforded, after purification by silica gel column chromatography (100% pentane with 1% Et_3N), the title compound (156 mg, 0.616 mmol, 77%) as a colourless oil.

^{1}H NMR (400 MHz, $CDCl_3$): δ 8.47 (d, *J* = 8.3 Hz, 1H), 7.94 (d, *J* = 5.8 Hz, 1H), 7.82 (d, *J* = 8.2 Hz, 1H), 7.75 (ddd, *J* = 8.2, 6.8, 1.3 Hz, 1H), 7.65 (ddd, *J* = 8.3, 6.8, 1.3 Hz, 1H), 7.31 (d, *J* = 5.8 Hz, 1H), 7.14 (dd, *J* = 8.7, 5.0 Hz, 1H), 7.06 – 6.94 (m, 2H), 2.18 (s, 3H).
^{13}C{^{1}H} NMR (101 MHz, $CDCl_3$): δ 160.3, 160.0 (d, *J* = 242.8 Hz), 148.0 (d, *J* = 2.6 Hz), 140.0, 138.6, 133.1 (d, *J* = 8.3 Hz), 131.1, 127.3, 126.5, 124.3, 123.7 (d, *J* = 8.8 Hz), 119.6, 117.8 (d, *J* = 22.9 Hz), 116.3, 113.7 (d, *J* = 23.2 Hz), 16.7 (d, *J* = 1.4 Hz).
^{19}F NMR (376 MHz, $CDCl_3$): δ -118.28 (ddd, *J* = 9.0, 8.0, 5.0 Hz).
ν_{max} (ATR)/cm^{-1}: 3058, 2957, 2925, 2857, 1630, 1593, 1572, 1490, 1369, 1341, 1270, 1248, 1204, 1183, 1145, 1059, 897, 865, 816, 750, 668.
HRMS calcd. for $C_{16}H_{12}FNO+H^+$: 254.0976 $[M+H]^+$; found (ESI$^+$) 254.0990.

2-(2-Ethoxyphenoxy)pyridine (15)

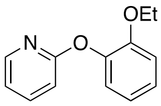

Synthesised according to **GP3** (**X** = 80, **Y** = 6). Using (2-ethoxyphenyl)boronic acid (146 mg, 0.880 mmol), 2-pyridone (76.1 mg, 0.800 mmol) and **workup A** afforded the title compound (139 mg, 0.646 mmol, 81%) as an oil which solidified on standing to a yellow solid. Characterisation data were consistent with literature values: ^{1}H and ^{13}C NMR, HRMS.[11]

^{1}H NMR (400 MHz, $CDCl_3$): δ 8.14 (ddd, *J* = 5.0, 2.0, 0.8 Hz, 1H), 7.65 (ddd, *J* = 8.3, 7.2, 2.0 Hz, 1H), 7.20 – 7.14 (m, 2H), 7.03 – 6.96 (m, 2H), 6.96 – 6.89 (m, 2H), 3.99 (q, *J* = 7.0 Hz, 2H), 1.15 (t, *J* = 7.0 Hz, 3H).

$^{13}C\{^1H\}$ NMR (101 MHz, $CDCl_3$): δ 164.1, 151.2, 147.6, 143.2, 139.1, 126.0, 123.2, 121.3, 118.0, 114.5, 110.8, 64.6, 14.7.

ν_{max} (ATR)/cm^{-1}: 2980, 1597, 1586, 1573, 1500, 1468, 1455, 1428, 1305, 1270, 1239, 1196, 1117, 1043, 880, 774, 748.

HRMS calcd. for $C_{13}H_{13}NO_2+H^+$: 216.1019 $[M+H]^+$; found (ESI^+) 216.1041.

m.p./°C: 56-57.

2-(5-Bromo-2-methoxyphenoxy)pyridine (16)

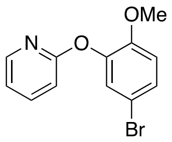

Synthesised according to **GP3** (**X** = 80, **Y** = 6). Using (5-bromo-2-methoxyphenyl)boronic acid (203 mg, 0.880 mmol), 2-pyridone (76.1 mg, 0.800 mmol) and **workup A** afforded the title compound (142 mg, 0.507 mmol, 63%) as an oil which solidified on standing to a yellow solid. Characterisation data were consistent with literature values: ^{1}H and ^{13}C NMR, HRMS, m.p..[11]

^{1}H NMR (400 MHz, $CDCl_3$): δ 8.14 (ddd, *J* = 5.0, 2.0, 0.8 Hz, 1H), 7.68 (ddd, *J* = 8.3, 7.2, 2.0 Hz, 1H), 7.33 – 7.27 (m, 2H), 6.98 (ddd, *J* = 7.2, 5.0, 0.9 Hz, 1H), 6.94 (dt, *J* = 8.3, 0.9 Hz, 1H), 6.88 (d, *J* = 8.6 Hz, 1H), 3.74 (s, 3H).

$^{13}C\{^1H\}$ NMR (101 MHz, $CDCl_3$): δ 163.4, 151.3, 147.6, 143.4, 139.5, 128.8, 126.4, 118.6, 114.3, 112.5, 111.0, 56.3.

ν_{max} (ATR)/cm^{-1}: 3058, 3011, 2941, 2837, 1599, 1583, 1571, 1495, 1467, 1429, 1401, 1270, 1237, 1200, 1178, 1132, 1027, 906, 877, 803, 779, 622.

HRMS calcd. for $C_{12}H_{10}BrNO_2+H^+$: 279.9968 $[M+H]^+$; found (ESI^+) 280.0012.

m.p./°C: 85-86.

2-(2-Ethylphenoxy)pyridine (17)

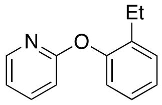

Synthesised according to **GP3** (**X** = 60, **Y** = 2). Using (2-ethylphenyl)boronic acid (132 mg, 0.880 mmol), 2-pyridone (76.1 mg, 0.800 mmol) and **workup A** afforded the title compound (140 mg, 0.703 mmol, 88%) as an orange oil.

^{1}H NMR (400 MHz, CDCl$_3$): δ 8.19 (ddd, *J* = 5.0, 2.0, 0.8 Hz, 1H), 7.66 (ddd, *J* = 8.3, 7.2, 2.0 Hz, 1H), 7.31 (dd, *J* = 7.4, 1.9 Hz, 1H), 7.25 – 7.21 (m, 1H), 7.18 (app. td, *J* = 7.4, 1.5 Hz, 1H), 7.04 (dd, *J* = 7.8, 1.5 Hz, 1H), 6.96 (ddd, *J* = 7.2, 5.0, 1.0 Hz, 1H), 6.85 (d, *J* = 8.3 Hz, 1H), 2.59 (q, *J* = 7.6 Hz, 2H), 1.18 (t, *J* = 7.6 Hz, 3H).

^{13}C{^{1}H} NMR (101 MHz, CDCl$_3$): δ 164.1, 151.9, 147.9, 139.5, 136.6, 129.9, 127.2, 125.5, 122.0, 118.2, 110.8, 23.4, 14.3.

ν_{max} (ATR)/cm^{-1}: 2968, 2933, 1596, 1572, 1490, 1467, 1452, 1427, 1285, 1264, 1244, 1216, 1177, 1142, 1117, 990, 886, 816, 769, 751.

HRMS calcd. for $C_{13}H_{13}NO+H^+$: 200.1070 $[M+H]^+$; found (ESI$^+$) 200.1073.

2-(2-Chlorophenoxy)pyridine (18)

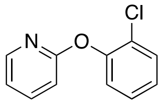

Synthesised according to **GP3** (**X** = 80, **Y** = 16). Using (2-chlorophenyl)boronic acid (138 mg, 0.880 mmol), 2-pyridone (76.1 mg, 0.800 mmol) and **workup A** afforded the title compound (128 mg, 0.622 mmol, 78%) as an oil which solidified on standing to an off-white solid. Characterisation data were consistent with literature values: ^{1}H and ^{13}C NMR.[12]

^{1}H NMR (400 MHz, CDCl$_3$): δ 8.16 (ddd, *J* = 5.0, 2.0, 0.8 Hz, 1H), 7.71 (ddd, *J* = 8.3, 7.2, 2.0 Hz, 1H), 7.48 (dd, *J* = 8.0, 1.6 Hz, 1H), 7.32 (ddd, *J* = 8.0, 7.3, 1.6 Hz, 1H), 7.25 – 7.16 (m, 2H), 7.02 – 6.96 (m, 2H).

$^{13}C\{^1H\}$ NMR (101 MHz, $CDCl_3$): δ 163.1, 150.0, 147.6, 139.7, 130.8, 128.0, 127.5, 126.3, 124.0, 118.7, 111.2.

ν_{max} (ATR)/cm^{-1}: 3063, 3015, 1598, 1584, 1572, 1477, 1464, 1446, 1427, 1286, 1266, 1241, 1219, 1142, 1061, 991, 882, 813, 774, 755, 736, 723, 683.

HRMS calcd. for $C_{11}H_8ClNO+H^+$: 206.0367 $[M+H]^+$; found (ESI^+) 206.0375.

m.p./°C: 84-85.

2-Fluoro-4-methyl-5-(pyridin-2-yloxy)pyridine (19)

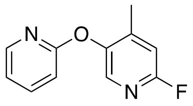

Synthesised according to **GP3** (**X** = 80, **Y** = 2). Using (6-fluoro-4-methylpyridin-3-yl)boronic acid (136 mg, 0.880 mmol), 2-pyridone (76.1 mg, 0.800 mmol) and **workup A** afforded the title compound (134 mg, 0.656 mmol, 82%) as a pale yellow oil.

^1H NMR (400 MHz, $CDCl_3$): δ 8.11 (ddd, *J* = 5.0, 2.0, 0.8 Hz, 1H), 7.94 (d, *J* = 1.3 Hz, 1H), 7.72 (ddd, *J* = 8.3, 7.2, 2.0 Hz, 1H), 7.04 – 6.95 (m, 2H), 6.84 (d, *J* = 2.7 Hz, 1H), 2.20 (s, 3H).

$^{13}C\{^1H\}$ NMR (101 MHz, $CDCl_3$): δ 163.1, 160.5 (d, *J* = 236.2 Hz), 147.6, 147.3 (d, *J* = 4.7 Hz), 146.3 (d, *J* = 8.9 Hz), 140.8 (d, *J* = 16.4 Hz), 139.9, 118.9, 111.0 (d, *J* = 39.7 Hz), 111.0, 16.7 (d, *J* = 2.4 Hz).

^{19}F NMR (376 MHz, $CDCl_3$): δ -73.66 (br app. s).

ν_{max} (ATR)/cm^{-1}: 3059, 1613, 1595, 1574, 1485, 1466, 1428, 1387, 1374, 1290, 1273, 1236, 1198, 1143, 960, 866, 776, 734.

HRMS calcd. for $C_{11}H_9FN_2O+H^+$: 205.0772 $[M+H]^+$; found (ESI^+) 205.0766.

2-((2-Methylnaphthalen-1-yl)oxy)pyridine (20)

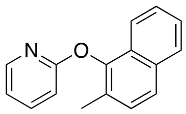

Synthesised according to **GP3** (**X** = 80, **Y** = 16). Using (2-methylnaphthalen-1-yl)boronic acid (164 mg, 0.880 mmol), 2-pyridone (76.1 mg, 0.800 mmol) and **workup A** afforded the title compound (161 mg, 0.685 mmol, 86%) as a brown solid.

^{1}H NMR (400 MHz, $CDCl_3$): δ 8.14 (ddd, *J* = 5.0, 2.0, 0.8 Hz, 1H), 7.91 – 7.82 (m, 2H), 7.69 (d, *J* = 8.4 Hz, 1H), 7.65 (ddd, *J* = 8.3, 7.2, 2.0 Hz, 1H), 7.49 – 7.33 (m, 3H), 6.95 (ddd, *J* = 7.2, 5.0, 0.9 Hz, 1H), 6.84 (app. dt, *J* = 8.3, 0.9 Hz, 1H), 2.31 (s, 3H).
^{13}C{^{1}H} NMR (101 MHz, $CDCl_3$): δ 164.1, 148.2, 146.7, 139.6, 133.7, 129.3, 128.1, 128.0, 127.2, 126.4, 125.6, 125.4, 121.9, 118.0, 109.8, 16.7.
ν_{max} (ATR)/cm^{-1}: 3054, 1596, 1570, 1466, 1427, 1373, 1284, 1262, 1243, 1177, 1142, 1079, 809, 777, 740.
HRMS calcd. for $C_{16}H_{13}NO+H^+$: 236.1070 $[M+H]^+$; found (ESI^+) 236.1080.
m.p./°C: 112-113.

2-(2,6-Dimethylphenoxy)pyridine (21)

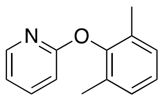

Synthesised according to **GP3** (**X** = 80, **Y** = 16). Using (2,6-dimethylphenyl)boronic acid (132 mg, 0.880 mmol), 2-pyridone (76.1 mg, 0.800 mmol) and **workup A** afforded the title compound (130 mg, 0.652 mmol, 82%) as a pale yellow oil. Characterisation data were consistent with literature values: ^{1}H and ^{13}C NMR.[13]

^{1}H NMR (400 MHz, $CDCl_3$): δ 8.16 (ddd, *J* = 5.0, 2.0, 0.8 Hz, 1H), 7.65 (ddd, *J* = 8.3, 7.1, 2.0 Hz, 1H), 7.14 – 7.04 (m, 3H), 6.93 (ddd, *J* = 7.1, 5.0, 1.0 Hz, 1H), 6.81 (app. dt, *J* = 8.3, 0.9 Hz, 1H), 2.13 (s, 6H).

^{13}C{^{1}H} NMR (101 MHz, $CDCl_3$): δ 163.3, 150.5, 148.0, 139.6, 131.3, 128.9, 125.5, 117.7, 109.8, 16.7.

ν_{max} (ATR)/cm^{-1}: 2922, 1587, 1573, 1465, 1425, 1302, 1284, 1269, 1245, 1182, 1142, 1090, 990, 881, 816, 770, 736.

HRMS calcd. for $C_{13}H_{13}NO+H^+$: 200.1070 $[M+H]^+$; found (ESI^+) 200.1081.

2-(4-Bromo-2,6-dimethylphenoxy)pyridine (22)

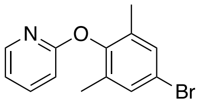

Synthesised according to **GP3** (**X** = 80, **Y** = 16). Using (4-bromo-2,6-dimethylphenyl)boronic acid (201 mg, 0.880 mmol), 2-pyridone (76.1 mg, 0.800 mmol) and **workup A** afforded the title compound (184 mg, 0.662 mmol, 83%) as an oil which solidified on standing to an off-white solid.

^{1}H NMR (400 MHz, $CDCl_3$): δ 8.13 (ddd, *J* = 5.0, 2.0, 0.8 Hz, 1H), 7.67 (ddd, *J* = 8.3, 7.2, 2.0 Hz, 1H), 7.24 (s, 2H), 6.95 (ddd, *J* = 7.2, 5.0, 1.0 Hz, 1H), 6.87 (app. dt, *J* = 8.3, 0.9 Hz, 1H), 2.09 (s, 6H).

^{13}C{^{1}H} NMR (101 MHz, $CDCl_3$): δ 162.9, 149.7, 148.0, 139.7, 133.5, 131.6, 118.3, 118.1, 110.1, 16.6.

ν_{max} (ATR)/cm^{-1}: 3055, 2955, 2922, 1592, 1571, 1464, 1426, 1377, 1303, 1285, 1274, 1241, 1181, 1141, 991, 887, 859, 838, 778, 736.

HRMS calcd. for $C_{13}H_{12}BrNO+H^+$: 278.0175 $[M+H]^+$; found (ESI^+) 278.0173.

m.p./°C: 62-63.

2-(4-Fluorophenoxy)pyridine (5)

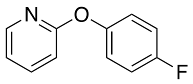

Synthesised according to **GP3** (**X** = 60, **Y** = 2). Using 4-fluorophenylboronic acid (123 mg, 0.880 mmol), 2-pyridone (76.1 mg, 0.800 mmol) and **workup A** afforded the title compound (104 mg, 0.550 mmol, 69%) as an oil which solidified on standing to an off-white solid. Characterisation data were consistent with literature values: ^{1}H, ^{13}C and ^{19}F NMR, ν_{max}, m.p..[12,14]

^{1}H NMR (400 MHz, $CDCl_3$): δ 8.18 (ddd, *J* = 5.0, 2.0, 0.8 Hz, 1H), 7.69 (ddd, *J* = 8.3, 7.2, 2.0 Hz, 1H), 7.15 – 7.04 (m, 4H), 6.99 (ddd, *J* = 7.2, 5.0, 0.9 Hz, 1H), 6.91 (app. dt, *J* = 8.3, 0.9 Hz, 1H).
^{13}C{^{1}H} NMR (101 MHz, $CDCl_3$): δ 163.8, 159.7 (d, *J* = 242.8 Hz), 149.9 (d, *J* = 2.8 Hz), 147.7, 139.6, 122.9 (d, *J* = 8.4 Hz), 118.6, 116.4 (d, *J* = 23.3 Hz), 111.5.
^{19}F NMR (376 MHz, $CDCl_3$): δ -118.53 (tt, *J* = 7.7, 5.0 Hz).
ν_{max} (ATR)/cm^{-1}: 3075, 3056, 3016, 1590, 1573, 1501, 1465, 1426, 1287, 1266, 1248, 1224, 1190, 1143, 1089, 991, 884, 851, 818, 777, 757, 735, 528, 499.
HRMS calcd. for $C_{11}H_8FNO+H^+$: 190.0663 $[M+H]^+$; found (ESI^+) 190.0670.
m.p./°C: 53-54.

2-(4-Fluorophenoxy)-3-methylpyridine (23)

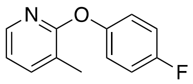

Synthesised according to **GP3** (**X** = 60, **Y** = 2). Using 4-fluorophenylboronic acid (123 mg, 0.880 mmol), 3-methylpyridin-2-ol (87.3 mg, 0.800 mmol) and **workup A** afforded the title compound (121 mg, 0.595 mmol, 74%) as a colourless oil.

^{1}H NMR (400 MHz, $CDCl_3$): δ 7.99 (d, *J* = 2.5 Hz, 1H), 7.50 (dd, *J* = 8.4, 2.5 Hz, 1H), 7.15 – 6.99 (m, 4H), 6.81 (d, *J* = 8.4 Hz, 1H), 2.28 (s, 3H).

$^{13}C\{^1H\}$ NMR (101 MHz, $CDCl_3$): δ 162.0, 159.5 (d, *J* = 242.4 Hz), 150.4 (d, *J* = 2.8 Hz), 147.4, 140.5, 128.0, 122.5 (d, *J* = 8.4 Hz), 116.3 (d, *J* = 23.3 Hz), 111.1, 17.6.

^{19}F NMR (377 MHz, $CDCl_3$): δ -118.98 (tt, *J* = 7.4, 5.4 Hz).

ν_{max} (ATR)/cm^{-1}: 3013, 2926, 1602, 1578, 1503, 1474, 1377, 1271, 1249, 1227, 1191, 1148, 1126, 1090, 1026, 888, 825, 780, 590, 514, 499.

HRMS calcd. for $C_{12}H_{10}FNO+H^+$: 204.0819 $[M+H]^+$; found (ESI^+) 204.0825.

4-(Benzyloxy)-2-(4-fluorophenoxy)pyridine (24)

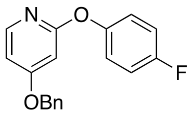

Synthesised according to **GP3** (**X** = 60, **Y** = 2). Using 4-fluorophenylboronic acid (123 mg, 0.880 mmol), 4-(benzyloxy)pyridin-2-ol (161 mg, 0.800 mmol) and **workup A** afforded the title compound (128 mg, 0.433 mmol, 54%) as an off-white solid.

^1H NMR (400 MHz, $CDCl_3$): δ 8.00 (d, *J* = 5.9 Hz, 1H), 7.47 – 7.33 (m, 5H), 7.11 – 7.00 (m, 4H), 6.63 (dd, *J* = 5.9, 2.2 Hz, 1H), 6.43 (d, *J* = 2.2 Hz, 1H), 5.10 (s, 2H).

$^{13}C\{^1H\}$ NMR (101 MHz, $CDCl_3$): δ 167.7, 165.7, 159.7 (d, *J* = 242.8 Hz), 149.9 (d, *J* = 2.7 Hz), 148.5, 135.7, 128.9, 128.6, 127.7, 122.9 (d, *J* = 8.4 Hz), 116.4 (d, *J* = 23.4 Hz), 107.6, 96.4, 70.3.

^{19}F NMR (376 MHz, $CDCl_3$): δ -118.54 (tt, *J* = 7.3, 5.4 Hz).

ν_{max} (ATR)/cm^{-1}: 3070, 1605, 1570, 1503, 1479, 1464, 1407, 1379, 1333, 1248, 1224, 1196, 1164, 1147, 1090, 1011, 990, 972, 854, 832, 813, 746, 697, 514.

HRMS calcd. for $C_{18}H_{14}FNO_2+H^+$: 296.1081 $[M+H]^+$; found (ESI^+) 296.1094.

m.p./°C: 87-88.

2-(4-Methoxyphenoxy)pyridine (25)

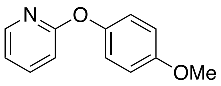

Synthesised according to **GP3** (**X** = 60, **Y** = 2). Using (4-methoxyphenyl)boronic acid (134 mg, 0.880 mmol), 2-pyridone (76.1 mg, 0.800 mmol) and **workup A** afforded the title compound (115 mg, 0.571 mmol, 71%) as a yellow oil. Characterisation data were consistent with literature values: ^{1}H and ^{13}C NMR, HRMS.[15]

^{1}H NMR (400 MHz, $CDCl_3$): δ 8.18 (ddd, *J* = 5.0, 2.0, 0.8 Hz, 1H), 7.65 (ddd, *J* = 8.3, 7.2, 2.0 Hz, 1H), 7.11 – 7.04 (m, 2H), 6.97 – 6.91 (m, 3H), 6.86 (dt, *J* = 8.3, 0.9 Hz, 1H), 3.81 (s, 3H).

^{13}C{^{1}H} NMR (101 MHz, $CDCl_3$): δ 164.4, 156.7, 147.8, 147.5, 139.4, 122.5, 118.2, 114.9, 111.2, 55.7.

ν_{max} (ATR)/cm^{-1}: 3056, 3007, 2953, 2908, 2835, 1611, 1590, 1573, 1504, 1465, 1427, 1286, 1267, 1235, 1200, 1180, 1143, 1101, 1034, 883, 842, 810, 779, 515.

HRMS calcd. for $C_{12}H_{11}NO_2$+H^+: 202.0863 [M+H]$^+$; found (ESI$^+$) 202.0877.

Methyl 3-(pyridin-2-yloxy)benzoate (26)

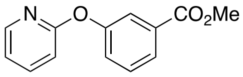

Synthesised according to **GP3** (**X** = 80, **Y** = 6). Using (3-(methoxycarbonyl)phenyl)boronic acid (158 mg, 0.880 mmol), 2-pyridone (76.1 mg, 0.800 mmol) and **workup B** afforded, after purification by silica gel column chromatography (0-10% EtOAc in cyclohexane with 1% Et_3N), the title compound (152 mg, 0.663 mmol, 83%) as a colourless oil.

^{1}H NMR (400 MHz, $CDCl_3$): δ 8.18 (ddd, *J* = 5.0, 2.0, 0.8 Hz, 1H), 7.88 (ddd, *J* = 7.8, 1.6, 1.1 Hz, 1H), 7.80 (dd, *J* = 2.4, 1.6 Hz, 1H), 7.71 (ddd, *J* = 8.3, 7.2, 2.0 Hz, 1H), 7.47 (app. t, *J* = 7.9 Hz, 1H), 7.35 (ddd, *J* = 8.1, 2.4, 1.1 Hz, 1H), 7.02 (ddd, *J* = 7.2, 5.0, 1.0 Hz, 1H), 6.95 (app. dt, *J* = 8.3, 0.9 Hz, 1H), 3.90 (s, 3H).

$^{13}C\{^1H\}$ NMR (101 MHz, $CDCl_3$): δ 166.6, 163.4, 154.3, 147.8, 139.7, 131.9, 129.8, 126.0, 125.9, 122.4, 119.0, 111.9, 52.4.

ν_{max} (ATR)/cm^{-1}: 3072, 3012, 2952, 1720, 1587, 1572, 1486, 1467, 1444, 1427, 1282, 1267, 1243, 1202, 1143, 1098, 1076, 991, 908, 776, 756, 694.

HRMS calcd. for $C_{13}H_{11}NO_3+H^+$: 230.0812 $[M+H]^+$; found (ESI^+) 230.0815.

2-Methoxy-5-(pyridin-2-yloxy)pyridine (27)

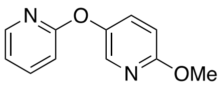

Synthesised according to **GP3** (**X** = 80, **Y** = 2). Using (6-methoxypyridin-3-yl)boronic acid (135 mg, 0.880 mmol), 2-pyridone (76.1 mg, 0.800 mmol) and **workup B** afforded, after purification by silica gel column chromatography (0-10% EtOAc in cyclohexane with 1% Et_3N), the title compound (102 mg, 0.504 mmol, 63%) as a colourless oil.

^1H NMR (400 MHz, $CDCl_3$): δ 8.14 (ddd, *J* = 5.0, 2.0, 0.8 Hz, 1H), 8.04 (dd, *J* = 2.9, 0.6 Hz, 1H), 7.68 (ddd, *J* = 8.3, 7.2, 2.0 Hz, 1H), 7.42 (dd, *J* = 8.9, 2.9 Hz, 1H), 6.98 (ddd, *J* = 7.2, 5.0, 1.0 Hz, 1H), 6.93 (app. dt, *J* = 8.3, 0.9 Hz, 1H), 6.78 (dd, *J* = 8.9, 0.6 Hz, 1H), 3.94 (s, 3H).

$^{13}C\{^1H\}$ NMR (101 MHz, $CDCl_3$): δ 163.7, 161.2, 147.6, 145.1, 139.6, 139.6, 133.1, 118.7, 111.4 (2C)*, 53.8.

ν_{max} (ATR)/cm^{-1}: 3057, 3014, 2978, 2944, 2843, 1593, 1572, 1485, 1464, 1425, 1380, 1262, 1235, 1211, 1142, 1116, 1022, 991, 914, 883, 838, 822, 777, 737, 581, 522.

HRMS calcd. for $C_{11}H_{10}N_2O_2+H^+$: 203.0815 $[M+H]^+$; found (ESI^+) 203.0821.

* Determined by HSQC.

iii. 4-Pyridone Substrates

4-(4-Fluoro-2-methylphenoxy)pyridine (28)

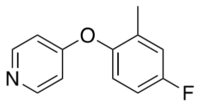

Synthesised according to **GP3** (**X** = 60, **Y** = 2). Using 4-fluoro-2-methylphenylboronic acid (135 mg, 0.880 mmol), 4-pyridone (76.1 mg, 0.800 mmol) and **workup A** afforded the title compound (143 mg, 0.704 mmol, 88%) as a colourless oil.

^{1}H NMR (400 MHz, CDCl$_3$): δ 8.44 (d, *J* = 5.5 Hz, 2H), 7.07 – 6.88 (m, 3H), 6.74 – 6.72 (m, 2H), 2.14 (s, 3H).
^{13}C{^{1}H} NMR (101 MHz, CDCl$_3$): δ 164.8, 160.2 (d, *J* = 244.5 Hz), 151.5, 147.7 (d, *J* = 2.8 Hz), 133.0 (d, *J* = 8.2 Hz), 122.9 (d, *J* = 8.9 Hz), 118.3 (d, *J* = 23.0 Hz), 114.3 (d, *J* = 23.2 Hz), 111.3, 16.3 (d, *J* = 1.2 Hz).
^{19}F NMR (376 MHz, CDCl$_3$): δ -116.92 (ddd, *J* = 8.8, 7.6, 5.0 Hz).
ν_{max} (ATR)/cm^{-1}: 3035, 2925, 1620, 1586, 1574, 1487, 1416, 1278, 1259, 1243, 1205, 1184, 1146, 992, 863, 821, 526, 451.
HRMS calcd. for $C_{12}H_{10}FNO+H^+$: 204.0819 $[M+H]^+$; found (ESI$^+$) 204.0829.

4-(4-Fluoro-2-methylphenoxy)-3-methylpyridine (29)

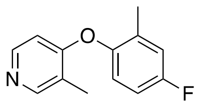

Synthesised according to **GP3** (**X** = 60, **Y** = 2). Using 4-fluoro-2-methylphenylboronic acid (135 mg, 0.880 mmol), 3-methylpyridin-4-ol (87.3 mg, 0.800 mmol) and **workup A** afforded the title compound (132 mg, 0.608 mmol, 76%) as an orange oil.

^{1}H NMR (400 MHz, CDCl$_3$): δ 8.37 (s, 1H), 8.22 (d, *J* = 5.6 Hz, 1H), 7.00 (dd, *J* = 8.8, 2.5 Hz, 1H), 6.98 – 6.90 (m, 2H), 6.32 (d, *J* = 5.6 Hz, 1H), 2.35 (s, 3H), 2.13 (s, 3H).

$^{13}C\{^1H\}$ NMR (101 MHz, CDCl$_3$): δ 162.9, 160.0 (d, *J* = 244.0 Hz), 152.1, 149.3, 148.2 (d, *J* = 2.8 Hz), 132.8 (d, *J* = 8.3 Hz), 122.7 (d, *J* = 8.9 Hz), 122.5, 118.3 (d, *J* = 22.9 Hz), 114.2 (d, *J* = 23.2 Hz), 108.6, 16.2 (d, *J* = 1.4 Hz), 13.2.

^{19}F NMR (377 MHz, CDCl$_3$): δ -117.41 (ddd, *J* = 8.8, 7.3, 5.6 Hz).

ν_{max} (ATR)/cm^{-1}: 2925, 1591, 1576, 1486, 1277, 1259, 1189, 1145, 999, 872, 823.

HRMS calcd. for $C_{13}H_{12}FNO+H^+$: 218.0976 [M+H]$^+$; found (ESI$^+$) 218.0981.

3-Bromo-4-(4-fluoro-2-methylphenoxy)pyridine (30)

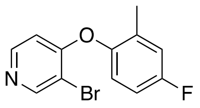

Synthesised according to **GP3** (**X** = 60, **Y** = 2). Using 4-fluoro-2-methylphenylboronic acid (135 mg, 0.880 mmol), 3-bromopyridin-4-ol (139 mg, 0.800 mmol) and **workup B** afforded, after purification by silica gel column chromatography (0-10% EtOAc in cyclohexane), the title compound (182 mg, 0.645 mmol, 81%) as an oil which solidified on standing to a colourless solid.

^1H NMR (400 MHz, CDCl$_3$): δ 8.69 (s, 1H), 8.28 (d, *J* = 5.6 Hz, 1H), 7.11 – 6.86 (m, 3H), 6.42 (d, *J* = 5.6 Hz, 1H), 2.15 (s, 3H).

$^{13}C\{^1H\}$ NMR (101 MHz, CDCl$_3$): δ 161.2, 160.4 (d, *J* = 245.1 Hz), 153.4, 150.1, 147.6 (d, *J* = 2.8 Hz), 132.8 (d, *J* = 8.4 Hz), 122.8 (d, *J* = 9.1 Hz), 118.5 (d, *J* = 23.1 Hz), 114.4 (d, *J* = 23.4 Hz), 110.3, 110.2, 16.2.

^{19}F NMR (376 MHz, CDCl$_3$): δ -116.26 (ddd, *J* = 8.8, 7.7, 5.0 Hz).

ν_{max} (ATR)/cm^{-1}: 3042, 2925, 1591, 1570, 1492, 1470, 1401, 1287, 1263, 1243, 1187, 1146, 1084, 1028, 872, 823, 728, 687, 530.

HRMS calcd. for $C_{12}H_9BrFNO+H^+$: 281.9924 [M+H]$^+$; found (ESI$^+$) 281.9925.

m.p./°C: 81-82.

4-(4-Fluoro-2-methylphenoxy)-6,7-dimethoxyquinoline (31)

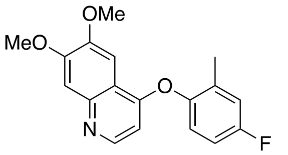

Synthesised according to **GP3** (**X** = 60, **Y** = 2). Using 4-fluoro-2-methylphenylboronic acid (135 mg, 0.880 mmol), 6,7-dimethoxyquinolin-4-ol (164 mg, 0.800 mmol) and **workup A** afforded the title compound (179 mg, 0.571 mmol, 71%) as an oil which solidified on standing to a beige solid.

^{1}H NMR (400 MHz, $CDCl_3$): δ 8.45 (d, *J* = 5.3 Hz, 1H), 7.59 (s, 1H), 7.42 (s, 1H), 7.10 – 7.01 (m, 2H), 6.97 (app. td, *J* = 8.3, 3.1 Hz, 1H), 6.24 (d, *J* = 5.3 Hz, 1H), 4.06 (s, 3H), 4.04 (s, 3H), 2.17 (s, 3H).

^{13}C{^{1}H} NMR (101 MHz, $CDCl_3$): δ 160.3, 160.2 (d, *J* = 244.5 Hz), 153.0, 149.7, 149.1, 148.2 (d, *J* = 2.7 Hz), 147.0, 133.1 (d, *J* = 8.3 Hz), 123.2 (d, *J* = 9.0 Hz), 118.3 (d, *J* = 22.8 Hz), 115.7, 114.3 (d, *J* = 23.2 Hz), 108.1, 102.2, 99.5, 56.29, 56.26, 16.2 (d, *J* = 1.4 Hz).

^{19}F NMR (376 MHz, $CDCl_3$): δ -116.94 (ddd, *J* = 8.8, 7.6, 4.9 Hz).

ν_{max} (ATR)/cm^{-1}: 2931, 1621, 1592, 1578, 1503, 1475, 1430, 1348, 1305, 1276, 1247, 1208, 1186, 1165, 1146, 1081, 1032, 995, 889, 854, 832, 729, 523.

HRMS calcd. for $C_{18}H_{16}FNO_3+H^+$: 314.1187 $[M+H]^+$; found (ESI^+) 314.1181.

m.p./°C: 92-93.

7-(4-Fluoro-2-methylphenoxy)thieno[3,2-*b*]pyridine (32)

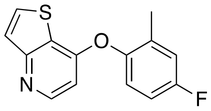

Synthesised according to **GP3** (**X** = 60, **Y** = 2). Using 4-fluoro-2-methylphenylboronic acid (135 mg, 0.880 mmol), thieno[3,2-*b*]pyridin-7-ol (121 mg, 0.800 mmol) and **workup A** afforded the title compound (186 mg, 0.717 mmol, 90%) as a yellow oil.

^{1}H NMR (400 MHz, CDCl$_3$): δ 8.47 (d, *J* = 5.4 Hz, 1H), 7.74 (d, *J* = 5.5 Hz, 1H), 7.57 (d, *J* = 5.5 Hz, 1H), 7.08 (dd, *J* = 8.8, 4.9 Hz, 1H), 7.03 (dd, *J* = 8.9, 3.1 Hz, 1H), 6.96 (app. td, *J* = 8.3, 3.1 Hz, 1H), 6.37 (d, *J* = 5.4 Hz, 1H), 2.17 (s, 3H).
^{13}C{^{1}H} NMR (101 MHz, CDCl$_3$): δ 160.4 (d, *J* = 244.9 Hz), 160.2, 159.2, 149.4, 147.7 (d, *J* = 2.9 Hz), 133.2 (d, *J* = 8.4 Hz), 130.7, 125.6, 123.1 (d, *J* = 9.0 Hz), 122.1, 118.3 (d, *J* = 22.9 Hz), 114.2 (d, *J* = 23.4 Hz), 103.2, 16.2 (d, *J* = 1.4 Hz).
^{19}F NMR (376 MHz, CDCl$_3$): δ -116.42 (ddd, *J* = 8.9, 7.8, 4.9 Hz).
ν_{max} (ATR)/cm^{-1}: 3103, 3055, 2924, 1585, 1545, 1486, 1452, 1378, 1289, 1261, 1181, 1146, 1128, 1026, 950, 867, 843, 818, 795, 781, 722, 553, 541, 455.
HRMS calcd. for $C_{14}H_{10}FNOS+H^+$: 260.0540 $[M+H]^+$; found (ESI$^+$) 260.0544.

4-(4-Fluoro-2-methylphenoxy)pyridin-2-amine (33)

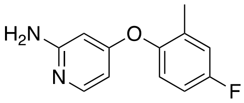

Synthesised according to **GP3** (**X** = 60, **Y** = 2)*. Using 4-fluoro-2-methylphenylboronic acid (135 mg, 0.880 mmol), (*tert*-butoxycarbonyl)(4-hydroxypyridin-2-yl)carbamate (248 mg, 0.800 mmol) and **workup A** afforded the title compound (147 mg, 0.674 mmol, 84%) as an off-white solid.

* 5 eq. of TsOH·H_2O used and stirred overnight before cooling to 0 °C and filtering to allow full deprotection of the Boc group alongside recovery of the bismacycle tosylate.

^{1}H NMR (400 MHz, CDCl$_3$): δ 7.91 (d, *J* = 5.9 Hz, 1H), 7.06 – 6.94 (m, 2H), 6.91 (app. td, *J* = 8.3, 3.0 Hz, 1H), 6.20 (dd, *J* = 5.9, 2.2 Hz, 1H), 5.82 (d, *J* = 2.2 Hz, 1H), 4.38 (s, 2H), 2.15 (s, 3H).

^{19}F NMR (376 MHz, CDCl$_3$): δ -117.42 (ddd, *J* = 8.8, 7.8, 5.1 Hz).

^{13}C{^{1}H} NMR (101 MHz, CDCl$_3$): δ 166.5, 160.3, 160.0 (d, *J* = 243.8 Hz), 149.9, 148.0 (d, *J* = 2.9 Hz), 133.1 (d, *J* = 8.2 Hz), 123.0 (d, *J* = 8.9 Hz), 118.1 (d, *J* = 23.0 Hz), 114.0 (d, *J* = 23.3 Hz), 103.5, 94.3, 16.3 (d, *J* = 1.4 Hz).

ν_{max} (ATR)/cm^{-1}: 3472, 3318 (br), 3178 (br), 1607, 1574, 1484, 1444, 1266, 1197, 998, 974, 948, 867, 824, 805, 455.

HRMS calcd. for $C_{12}H_{11}FN_2O+H^+$: 219.0928 $[M+H]^+$; found (ESI$^+$) 219.0932.

m.p./°C: 95-96.

4-(2,6-Dimethylphenoxy)pyridine (34)

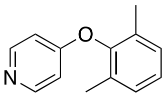

Synthesised according to **GP3** (**X** = 80, **Y** = 16). Using 2,6-dimethylphenylboronic acid (132 mg, 0.880 mmol), 4-pyridone (76.1 mg, 0.800 mmol) and **workup A** afforded the title compound (125 mg, 0.627 mmol, 78%) as an oil which solidified on standing to a yellow solid.

^{1}H NMR (400 MHz, CDCl$_3$): δ 8.42 (d, *J* = 5.7 Hz, 2H), 7.16 – 7.06 (m, 3H), 6.71 – 6.66 (m, 2H), 2.10 (s, 6H).

^{13}C{^{1}H} NMR (101 MHz, CDCl$_3$): δ 164.1, 151.6, 149.9, 131.1, 129.3, 126.0, 110.6, 16.3.

ν_{max} (ATR)/cm^{-1}: 3032, 2922, 1597, 1576, 1493, 1471, 1417, 1268, 1254, 1205, 1180, 1088, 991, 877, 822, 807, 771, 526.

HRMS calcd. for $C_{13}H_{13}NO+H^+$: 200.1070 $[M+H]^+$; found (ESI$^+$) 200.1081.

m.p./°C: 58-59.

4-(4-Fluorophenoxy)pyridine (35)

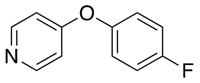

Synthesised according to **GP3** (**X** = 60, **Y** = 2). Using 4-fluorophenylboronic acid (123 mg, 0.880 mmol), 4-pyridone (76.1 mg, 0.800 mmol) and **workup A** afforded the title compound (109 mg, 0.576 mmol, 72%) as a yellow oil.

^{1}H NMR (400 MHz, CDCl$_3$): δ 8.47 – 8.44 (m, 2H), 7.18 – 6.96 (m, 4H), 6.82 – 6.77 (m, 2H).
^{13}C{^{1}H} NMR (101 MHz, CDCl$_3$): δ 165.0, 160.1 (d, *J* = 244.5 Hz), 151.6, 149.9 (d, *J* = 2.9 Hz), 122.5 (d, *J* = 8.4 Hz), 117.0 (d, *J* = 23.6 Hz), 112.0.
^{19}F NMR (377 MHz, CDCl$_3$): δ -116.97 (tt, *J* = 7.9, 4.6 Hz).
ν_{max} (ATR)/cm^{-1}: 3037, 1601, 1573, 1501, 1489, 1416, 1265, 1229, 1204, 1188, 1148, 1090, 992, 881, 851, 817, 754, 705, 598, 523, 497.
HRMS calcd. for $C_{11}H_8FNO+H^+$: 190.0663 $[M+H]^+$; found (ESI$^+$) 190.0676.

7-(4-Fluorophenoxy)thieno[3,2-*b*]pyridine (36)

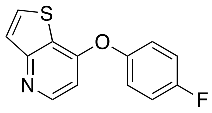

Synthesised according to **GP3** (**X** = 60, **Y** = 2). Using 4-fluorophenylboronic acid (123 mg, 0.880 mmol), thieno[3,2-*b*]pyridin-7-ol (121 mg, 0.800 mmol) and **workup A** afforded the title compound (157 mg, 0.640 mmol, 80%) as a yellow oil. Characterisation data were consistent with literature values: ^{1}H and ^{13}C NMR.[16]

^{1}H NMR (400 MHz, CDCl$_3$): δ 8.51 (d, *J* = 5.4 Hz, 1H), 7.74 (d, *J* = 5.5 Hz, 1H), 7.57 (d, *J* = 5.5 Hz, 1H), 7.22 – 7.08 (m, 4H), 6.52 (d, *J* = 5.4 Hz, 1H).
^{13}C{^{1}H} NMR (101 MHz, CDCl$_3$): δ 160.5, 160.3 (d, *J* = 244.9 Hz), 159.3, 149.7 (d, *J* = 2.9 Hz), 149.4, 130.8, 125.5, 122.7 (d, *J* = 8.6 Hz), 122.7, 117.0 (d, *J* = 23.6 Hz), 104.0.
^{19}F NMR (377 MHz, CDCl$_3$): δ -116.44 (tt, *J* = 7.5, 4.9 Hz).
ν_{max} (ATR)/cm^{-1}: 3059, 1582, 1546, 1495, 1453, 1377, 1289, 1266, 1232, 1192, 1127, 1090, 1027, 849, 826, 790, 755, 708, 505.
HRMS calcd. for $C_{13}H_8FNOS+H^+$: 246.0383 $[M+H]^+$; found (ESI$^+$) 246.0394.

3-Bromo-4-(4-fluorophenoxy)pyridine (37)

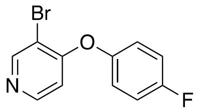

Synthesised according to **GP3** (**X** = 60, **Y** = 2). Using 4-fluorophenylboronic acid (123 mg, 0.880 mmol), 3-bromopyridin-4-ol (139 mg, 0.800 mmol) and **workup B** afforded, after purification by silica gel column chromatography (0-4% EtOAc in cyclohexane with 1% Et_3N), the title compound (152 mg, 0.567 mmol, 71%) as a colourless oil.

^{1}H NMR (400 MHz, $CDCl_3$): δ 8.68 (s, 1H), 8.30 (d, *J* = 5.6 Hz, 1H), 7.20 – 6.99 (m, 4H), 6.57 (d, *J* = 5.6 Hz, 1H).

^{13}C{^{1}H} NMR (101 MHz, $CDCl_3$): δ 161.6, 160.3 (d, *J* = 245.2 Hz), 150.0, 153.5, 149.6 (d, *J* = 2.9 Hz), 122.4 (d, *J* = 8.5 Hz), 117.2 (d, *J* = 23.6 Hz), 111.3, 110.7.

^{19}F NMR (377 MHz, $CDCl_3$): δ -116.22 (tt, *J* = 7.9, 4.6 Hz).

ν_{max} (ATR)/cm^{-1}: 3044, 1570, 1498, 1467, 1400, 1273, 1229, 1187, 1149, 1084, 1027, 1013, 886, 849, 820, 757, 727, 687, 560, 531, 506.

HRMS calcd. for $C_{11}H_7BrFNO+H^+$: 267.9768 $[M+H]^+$; found (ESI^+) 267.9758.

4-(4-Vinylphenoxy)pyridine (38)

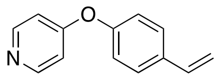

Synthesised according to **GP3** (**X** = 60, **Y** = 2). Using (4-vinylphenyl)boronic acid (130 mg, 0.880 mmol), 4-pyridone (76.1 mg, 0.800 mmol) and **workup A** afforded the title compound (118 mg, 0.598 mmol, 75%) a yellow oil.

^{1}H NMR (400 MHz, $CDCl_3$): δ 8.55 – 8.37 (m, 2H), 7.52 – 7.38 (m, 2H), 7.07 – 7.02 (m, 2H), 6.88 – 6.79 (m, 2H), 6.72 (dd, *J* = 17.6, 10.9 Hz, 1H), 5.73 (dd, *J* = 17.6, 0.8 Hz, 1H), 5.27 (dd, *J* = 10.9, 0.8 Hz, 1H).

^{13}C{^{1}H} NMR (101 MHz, $CDCl_3$): δ 164.8, 153.7, 151.6, 135.8, 135.1, 128.1, 121.0, 114.2, 112.3.

ν_{max} **(ATR)/cm^{-1}:** 3034, 1578, 1504, 1504, 1490, 1416, 1257, 1210, 1164, 1013, 990, 909, 879, 854, 820, 523, 481.

HRMS calcd. for $C_{13}H_{11}NO+H^+$: 198.0913 $[M+H]^+$; found (ESI^+) 198.0921.

4-(3-Ethoxyphenoxy)pyridine (39)

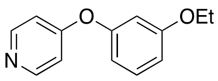

Synthesised according to **GP3** (**X** = 80, **Y** = 2). Using (3-ethoxyphenyl)boronic acid (146 mg, 0.880 mmol), 4-pyridone (76.1 mg, 0.800 mmol) and **workup A** afforded the title compound (138 mg, 0.641 mmol, 80%) as a brown oil.

^{1}H NMR (400 MHz, $CDCl_3$): δ 8.49 – 8.43 (m, 2H), 7.30 (app. t, *J* = 8.2 Hz, 1H), 6.89 – 6.81 (m, 2H), 6.78 (ddd, *J* = 8.4, 2.4, 0.9 Hz, 1H), 6.66 (ddd, *J* = 8.0, 2.2, 0.9 Hz, 1H), 6.62 (app. t, *J* = 2.3 Hz, 1H), 4.02 (q, *J* = 7.0 Hz, 2H), 1.41 (t, *J* = 7.0 Hz, 3H).

^{13}C{^{1}H} NMR (101 MHz, $CDCl_3$): δ 164.8, 160.7, 155.2, 151.5, 130.7, 112.8, 112.4, 111.8, 107.3, 63.8, 14.8.

ν_{max} **(ATR)/cm^{-1}:** 2980, 1578, 1488, 1474, 1445, 1416, 1268, 1250, 1206, 1165, 1137, 1114, 1047, 992, 978, 856, 822, 777, 691.

HRMS calcd. for $C_{13}H_{13}NO_2+H^+$: 216.1019 $[M+H]^+$; found (ESI^+) 216.1022.

iv. Active Ingredient Targets

4-(3,4-Dimethoxyphenoxy)-6,7-dimethoxyquinoline (Ki6783) (40)

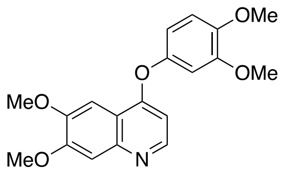

Synthesised according to **GP3** (**X** = 60, **Y** = 2). Using (3,4-dimethoxyphenyl)boronic acid (100 mg, 0.550 mmol), 6,7-dimethoxyquinolin-4-ol (103 mg, 0.500 mmol) and **workup A** afforded the title compound (96.1 mg, 0.281 mmol, 56%) as an off-white solid. Characterisation data were consistent with literature values: ^{1}H NMR, m.p..[17]

^{1}H NMR (400 MHz, CDCl$_3$): δ 8.48 (d, *J* = 5.3 Hz, 1H), 7.57 (s, 1H), 7.42 (s, 1H), 6.97 – 6.87 (m, 1H), 6.81 – 6.71 (m, 2H), 6.44 (d, *J* = 5.3 Hz, 1H), 4.05 (s, 3H), 4.05 (s, 3H), 3.93 (s, 3H), 3.86 (s, 3H).
^{13}C{^{1}H} NMR (101 MHz, CDCl$_3$): δ 161.2, 152.9, 150.2, 149.6, 149.1, 148.0, 147.0, 146.9, 116.0, 112.7, 111.8, 108.0, 105.5, 103.1, 99.6, 56.4, 56.3, 56.21, 56.18.
ν_{max} (ATR)/cm^{-1}: 2937, 1623, 1600, 1577, 1506, 1479, 1431, 1349, 1303, 1249, 1229, 1209, 1191, 1167, 1153, 1122, 1027, 996, 948, 913, 884, 852, 823, 767, 729, 561.
HRMS calcd. for $C_{19}H_{19}NO_5$+H^{+}: 342.1336 [M+H]$^{+}$; found (ESI^{+}) 342.1335.
m.p./°C: 185-186.

4-((6,7-Dimethoxyquinolin-4-yl)oxy)aniline (41)

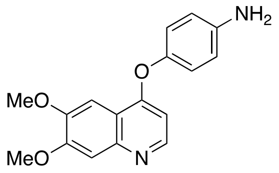

Synthesised according to **GP3** (**X** = 60, **Y** = 2)*. Using (4-((*tert*-butoxycarbonyl)amino)phenyl)boronic acid (130 mg, 0.550 mmol), 6,7-dimethoxyquinolin-4-ol (103 mg, 0.500 mmol) and **workup B** afforded, after purification by silica gel column

chromatography (40-80% EtOAc in cyclohexane with 1% Et_3N), the title compound (76.8 mg, 0.259 mmol, 52%) as a pale pink-brown solid.

* 4 eq. of TsOH·H_2O used and stirred overnight before cooling to 0 °C and filtering to allow full deprotection of the Boc group alongside recovery of the bismacycle tosylate.

^{1}H NMR (400 MHz, $CDCl_3$): δ 8.46 (d, *J* = 5.3 Hz, 1H), 7.58 (s, 1H), 7.40 (s, 1H), 7.06 – 6.95 (m, 2H), 6.81 – 6.67 (m, 2H), 6.42 (d, *J* = 5.3 Hz, 1H), 4.05 (s, 3H), 4.04 (s, 3H), 3.71 (br s, 2H).
^{13}C{^{1}H} NMR (101 MHz, $CDCl_3$): δ 161.6, 152.8, 149.5, 149.2, 146.9, 146.3, 144.3, 122.4, 116.4, 116.1, 108.0, 102.9, 99.8, 56.3, 56.2.
ν_{max} (ATR)/cm^{-1}: 3370 (br), 3210 (br), 1623, 1580, 1507, 1478, 1431, 1350, 1303, 1270, 1250, 1214, 1200, 1167, 995, 895, 853, 821, 731, 514.
HRMS calcd. for $C_{17}H_{16}N_2O_3+H^+$: 297.1234 $[M+H]^+$; found (ESI$^+$) 297.1244.
m.p./°C: 202-203.

_N_-(4-Fluorophenyl)-6-(3-(trifluoromethyl)phenoxy)picolinamide (Picolinafen) (42)

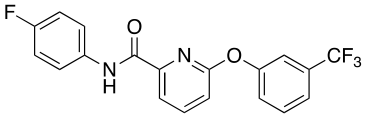

Synthesised according to **GP3** (**X** = 80, **Y** = 6). Using (3-(trifluoromethyl)phenyl)boronic acid (104 mg, 0.550 mmol), *N*-(4-fluorophenyl)-6-hydroxypicolinamide (116 mg, 0.500 mmol) and **workup B** afforded, after purification by silica gel column chromatography (0-5% EtOAc in cyclohexane), the title compound (128 mg, 0.341 mmol, 68%) as a colourless solid. Characterisation data were consistent with literature values: ^{1}H, ^{13}C and ^{19}F NMR, m.p..[18,19]

^{1}H NMR (400 MHz, $CDCl_3$): δ 9.25 (br s, 1H), 8.04 (dd, *J* = 7.4, 0.9 Hz, 1H), 7.96 (t, *J* = 7.8 Hz, 1H), 7.68 – 7.53 (m, 3H), 7.52 – 7.45 (m, 2H), 7.41 (app. dt, *J* = 7.6, 2.1 Hz, 1H), 7.18 (dd, *J* = 8.1, 0.9 Hz, 1H), 7.02 (app. t, *J* = 8.7 Hz, 2H).
^{13}C{^{1}H} NMR (101 MHz, $CDCl_3$): δ 161.5, 161.1, 159.5 (d, *J* = 243.6 Hz), 153.7, 147.5, 141.7, 133.6 (d, *J* = 2.9 Hz), 132.3 (q, *J* = 32.9 Hz), 130.5, 125.1, 123.3 (q, *J* = 272.5 Hz)*,

122.1 (q, J = 3.8 Hz), 121.1 (d, J = 7.9 Hz), 119.0 (q, J = 3.8 Hz), 117.9, 115.9 (d, J = 22.6 Hz), 115.1.

^{19}F NMR (376 MHz, $CDCl_3$): δ -62.52 (s, 3F), -117.79 (tt, J = 8.5, 4.7 Hz, 1F).

ν_{max} (ATR)/cm^{-1}: 3353, 2925, 1690, 1594, 1575, 1531, 1510, 1441, 1408, 1327, 1281, 1262, 1227, 1212, 1171, 1129, 1066, 979, 901, 833, 762, 698.

HRMS calcd. for $C_{19}H_{12}F_4N_2O_2+H^+$: 377.0908 $[M+H]^+$; found (ESI$^+$) 377.0907.

m.p./°C: 100-101.

* Partly obscured by signal at 125.1.

Benzyl (4-((2-aminopyridin-4-yl)oxy)-2-fluorophenyl)carbamate (43)

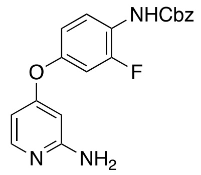

Synthesised according to **GP3** (**X** = 60, **Y** = 2)*. Using (4-(((benzyloxy)carbonyl)amino)-3-fluorophenyl)boronic acid (159 mg, 0.550 mmol), *tert*-butyl (4-hydroxypyridin-2-yl)carbamate (105 mg, 0.500 mmol) and **workup B** afforded, after purification by silica gel column chromatography (0-50% EtOAc in cyclohexane with 1% Et_3N), the title compound (91.1 mg, 0.258 mmol, 52%) as a colourless solid. Characterisation data were consistent with literature values: ^{1}H and ^{13}C NMR, HRMS.[20]

* 5 eq. of TsOH·H_2O used and stirred overnight before cooling to 0 °C and filtering to allow full deprotection of the Boc group alongside recovery of the bismacycle tosylate.

^{1}H NMR (400 MHz, $CDCl_3$): δ 8.12 (br s, 1H), 7.96 (d, J = 5.8 Hz, 1H), 7.48 – 7.33 (m, 5H), 6.99 – 6.94 (m, 1H), 6.94 – 6.81 (m, 2H), 6.29 (dd, J = 5.8, 2.2 Hz, 1H), 5.97 (d, J = 2.2 Hz, 1H), 5.25 (s, 2H), 4.46 (br s, 2H).

^{13}C{^{1}H} NMR (126 MHz, $CDCl_3$): δ 166.1, 160.2, 153.2, 152.4 (d, J = 245.9 Hz), 149.9, 149.8 (d, J = 10.4 Hz), 135.7, 128.7, 128.6, 128.4, 123.5 (d, J = 10.3 Hz), 121.3, 116.9 (d, J = 3.6 Hz), 108.5 (d, J = 21.7 Hz), 104.1, 95.4, 67.5.

^{19}F NMR (376 MHz, $CDCl_3$): δ -128.09 (br app. s).

ν_{max} (ATR)/cm^{-1}: 3380 (br), 3190 (br), 1718, 1600, 1528, 1486, 1444, 1428, 1307, 1258, 1225, 1187, 1142, 1106, 1048, 1029, 999, 981, 940, 736, 698.

HRMS calcd. for $C_{19}H_{16}FN_3O_3+H^+$: 354.1248 $[M+H]^+$; found (ESI^+) 354.1243.

m.p./°C: 143-144.

7. Robustness Screen

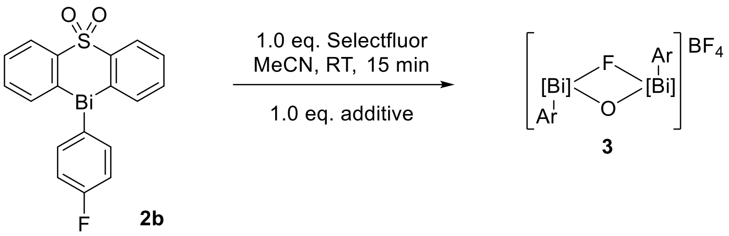

Entry	Additive	3 (%)	Additive Remaining (%)
1	none	Quant.	n/a
2	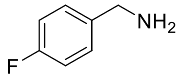	55	19
3	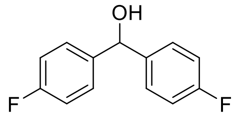	89	92
4	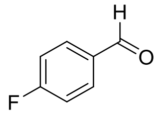	97	Quant.
5	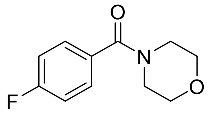	98	95
6	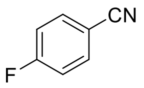	93	95

Table S9. Robustness screen of Bi(III) → Bi(V) oxidation. Yields of **3** and remaining additive determined by ^{19}F NMR spectroscopy against an internal standard.

Bismuth(III) stock solution: A solution of aryl bismacycle **2b** (57.2 mg, 0.110 mmol) and 4,4'-bis(trifluoromethyl)-1,1'-biphenyl (internal standard for ^{19}F NMR spectroscopy) in CH_2Cl_2 (1.1 mL) was prepared. An aliquot was analysed by quantitative ^{19}F NMR spectroscopy to confirm the initial bismacycle : internal standard ratio.

Procedure: An aliquot of the bismuth(III) stock solution (0.20 mL of a 0.10 M solution in CH_2Cl_2, 0.020 mmol) was added to a 2 mL vial and concentrated to dryness under a stream of

nitrogen. The appropriate additive (1.0 equiv.) was added, followed by Selectfluor (0.20 mL of a 0.10 M solution in MeCN, 0.02 mmol). The mixture was stirred at RT for 15 mins, then diluted with d_3-MeCN (0.30 mL) prior to analysis by quantitative ^{19}F NMR spectroscopy.

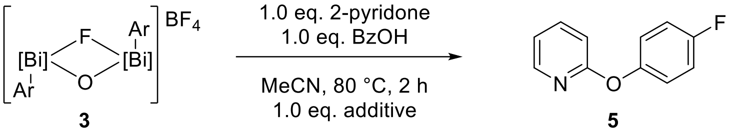

Entry	Additive	5 (%)	Additive Remaining (%)
1	none	76	n/a
2	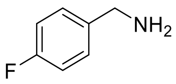	15	26
3	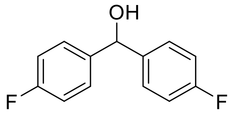	49	87
4	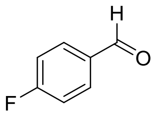	57	Quant.
5	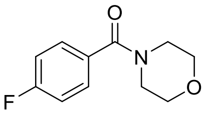	79	96
6	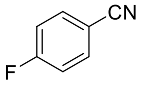	64	Quant.

Table S10. Robustness screen of Bi(V)-mediated arylation of 2-pyridone. Yields of **5** and remaining additive determined by ^{19}F NMR spectroscopy against an internal standard.

Bismuth(V) stock solution: A solution of aryl bismacycle **2b** (57.2 mg, 0.110 mmol), Selectfluor (39.0 mg, 0.110 mmol) and 4,4'-bis(trifluoromethyl)-1,1'-biphenyl (internal standard for ^{19}F NMR spectroscopy) in MeCN (1.10 mL) was mixed for 15 mins. An aliquot was analysed by quantitative ^{19}F NMR spectroscopy to confirm the initial bismacycle : internal standard ratio.

2-Pyridone stock solution: 2-Pyridone (10.46 mg, 0.110 mmol) and benzoic acid (13.43 mg, 0.110 mmol) were dissolved in CH_2Cl_2 (1.10 mL).

Procedure: An aliquot of the 2-pyridone stock solution (0.20 mL of a 0.10 M solution in CH_2Cl_2, 0.020 mmol) was added to a 2 mL vial and concentrated to dryness under a stream of nitrogen. The appropriate additive (1.0 equiv.) was added, followed by an aliquot of the bismuth(V) stock solution (0.20 mL of a 0.10 M solution in MeCN, 0.02 mmol). The mixture was stirred at 80 °C for 2 h, then allowed to cool to RT and diluted with d_3-MeCN (0.30 mL) prior to analysis by quantitative ^{19}F NMR spectroscopy.

8. Computational Details

i. Computational Methods

Geometry optimizations of ground-state reactants, products, and intermediates (INT) and transition state (TS) structures were carried out with *Gaussian 16 rev. C.01*[21] with density functional theory (DFT). The range-separated, dispersion-corrected ωB97X-D functional[22] was used with the 6-31+G(d,p) basis set for all atoms and def2SVP for bismuth to optimize the geometries of all ground and transition states. Single point electronic energies were evaluated at optimized stationary point geometries at the ωB97X-D/def2-QZVPP level of theory. The SMD implicit solvation model[23] (solvent = acetonitrile (MeCN) for the bismacycle system and solvent = tetrahydrofuran (THF) for the Ph_3BiCl_2 system) was used throughout. All DFT calculations used a "superfine" pruned (175,974) grid for numerical integration. A default "ultrafine" grid is not recommended for this system due to lack of rotational invariance, resulting in orientational dependence of Gibbs energy differences.[24]

Conformations of starting materials, intermediates, and TS structures were surveyed in terms of rotations about single bonds and the apical/equatorial positioning of substituents about Bi. Vibrational frequency calculations were used to characterize GS and TS structures on the potential energy surface (PES), which possess zero and one imaginary frequency, respectively. Intrinsic Reaction Coordinate (IRC) calculations were performed to connect TS structures with corresponding intermediates on the PES.

Atomic charges and Wiberg bond indices were calculated using natural population analysis (NPA) with *NBO 7.0*[25] in acetonitrile for the bismacycle system and tetrahydrofuran for the Ph_3BiCl_2 system with the superfine grid, interfaced to *Gaussian 16*. *NCIPLOT*[26] created the non-covalent interaction plots. Molecular representations were created with *PyMol*[27] for which the display settings are openly accessible.[28]

ii. Absolute Energy Values

Quasi-harmonic corrections were applied to the computed vibrational entropies using the *GoodVibes*[29] program. Standard state corrections were evaluated at 1M concentration and a reaction temperature of 80 °C (for the bismacyle system) and 60 °C (for Ph_3BiCl_2). Reported Gibbs free energies (kcal/mol) are Boltzmann weighted over all the conformers found in each reaction step. All thermochemical data, including absolute energies and zero-point energies (ZPE) were generated and tabulated separately as ESI along with the keywords for creating the potential energy profile.

iii. Molecular Coordinates

An xyz file containing all the geometries studied is provided separately along with the ESI.

We evaluated the selected level of theory for describing the structures of bismuth(III) and bismuth(V) species by comparison to the X-ray crystallographic structures of the aryl bismacycle(III) and the bismuth(V) dimer (**Figure S7**).[5] The computed structures for both methods shown in **Table S11** resulted in a high level of agreement between theory and experiment as all angles and dihedrals are well reproduced, and: (i) with def2SVP for the Bi atom, the Bi-C bond distances differ by less than 1.5% and Bi-O distances by less than 2.5%; (ii) with def2TZVP for the Bi atom, the Bi-C and Bi-O bond distances differ by less than 0.5%. Given that using the def2TZVP basis set for the Bi atom resulted in higher accuracy (relative to the X-ray crystallographic structures) for both the Bi(III) and Bi(V) species, subsequent optimizations were performed using this method to evaluate the selectivity determining steps for the arylation of 2-pyridiones by both the bismacyclic system and Ph_3BiCl_2. These additional Gibbs free energy values are reported in parentheses under the relevant reaction steps.

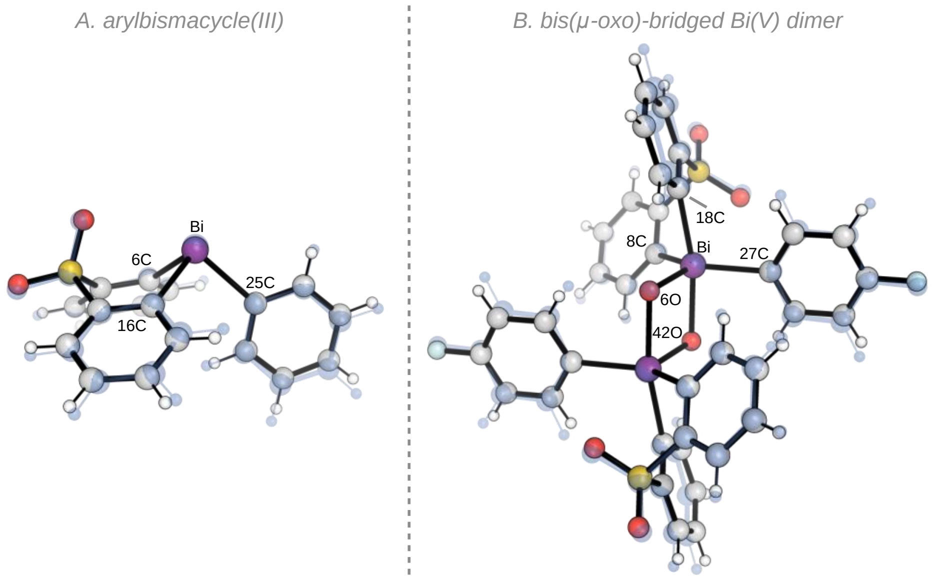

Figure S7. Overlay of the X-ray structure of aryl bismacycles (Crystallography Open Database, COD, identifier: 1904063) (left) and bis(μ-oxo)-bridged Bi(v) dimer (COD identifier: 1904073) (right). Crystal structures are represented with standard colors and optimized geometries with (SMD-acetonitrile) ωB97X-D/6-31+G(d,p), def2SVP (Bi atom) are shown in blue.

Bonds	Bond length (Å) Bi-crystal	Bond length (Å) and Bond Variation (%) (SMD-MeCN) WB97XD/ 6-31+G(d,p) (C,H,S,O,F)	
		def2SVP (Bi)	def2TZVP (Bi)
Bi-25C	2.249	2.270 (0.9)	2.248 (0.0)
Bi-16C	2.266	2.293 (1.2)	2.271 (0.2)
Bi-6C	2.272	2.293 (0.9)	2.271 (0.0)
Bi-27C	2.201	2.218 (0.8)	2.198 (-0.1)
Bi-18C	2.282	2.299 (0.8)	2.287 (0.2)
Bi-8C	2.240	2.248 (0.3)	2.231 (-0.4)
Bi-42O	2.202	2.249 (2.2)	2.204 (0.1)
Bi-6O	2.030	2.053 (1.1)	2.029 (-0.1)

Table S11. Tabulation of the bond lengths (Å) and the percentage of bond variation from the X-ray crystal structures shown in parenthesis. Bond lengths were determined experimentally from the Bi(III) and Bi(V) dimer X-ray structures and computationally using optimized structures from different levels of theories for bismuth.

iv. Bismacycle-based Arylation: O- and N-Arylation Pathways

We evaluated the reaction pathways for 2-pyridone O- and N-arylation (**Figure S8, A and B**) starting from the proposed bismuth(V) intermediate that is complexed to 2-pyridone and benzoate. In the most stable conformers of the Bi(V) intermediate, the benzoate and pyridone substituents occupy apical positions. 2-Pyridone can coordinate to the bismuth(V) complex through either the nitrogen, **Int-N**, or oxygen atom, **Int-O**, and there is a slight difference in stability (0.3 kcal/mol) between these two coordination modes. For the arylation step, we surveyed 3- and 5-membered transition state structures (see below), finding the 5-membered TS most stable for O- and N- arylation pathways (**TS-CO** and **TS-CN**). This step is highly exergonic and selectivity-determining. We confirmed that interconversion between intermediates **Int-N** and **Int-O** is facile (via a small barrier of 4.0 kcal/mol, **Figure S8 C**), such that a Curtin-Hammett scenario is operative. Consistent with experiment, O-arylation is predicted to be kinetically favored ($\Delta\Delta G^{\ddagger}$ = 3.0 kcal/mol).

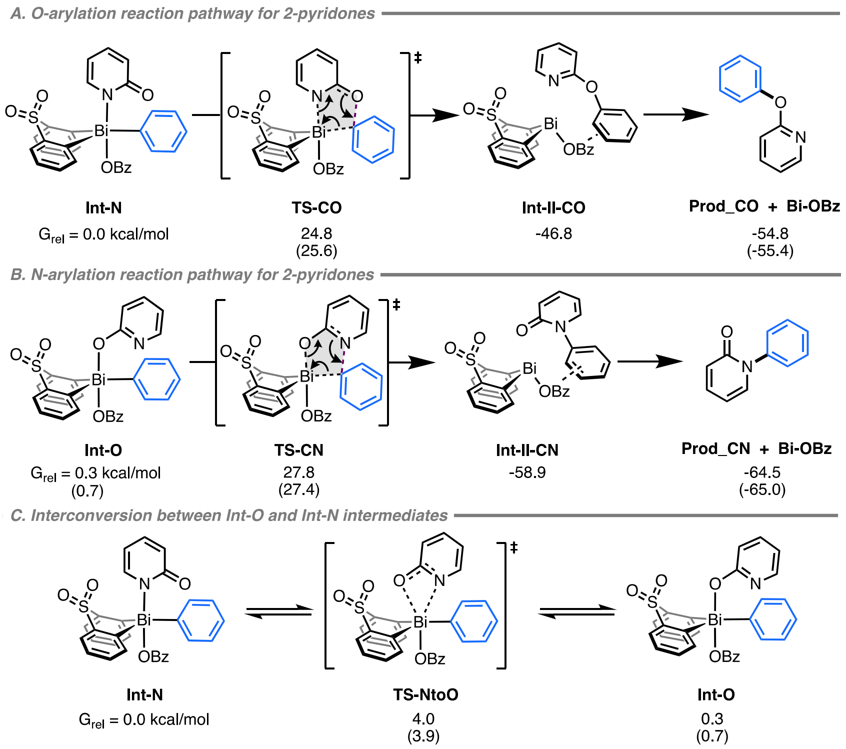

Figure S8. Computed O- (A) and N-arylation (B) reaction pathways with the bismacycle scaffold. Small barrier for interconversion between the O-bound and N-bound coordination modes (C). Boltzmann weighted G of relevant reaction steps calculated using the SMD solvation model (solvent = MeCN) with ωB97XD/def2-QZVPP// ωB97XD/6-31+G(d,p) and def2SVP (Bi) at 80 °C. Gibbs free energy values in parentheses were calculated using (SMD, MeCN) with ωB97XD/def2-QZVPP// ωB97XD/6-31+G(d,p) and def2TZVP (Bi) at 80 °C.

We evaluated the reaction pathways for 4-pyridone O- and N-arylation (**Figure S9** and **Figure S10**). Based on the method reported by Mukaiyama[30][31] and its postulated transformation for 4-pyridones, we calculated the arylation TS *via* a 3-membered TS cyclic structure. We found the N-arylation reaction pathway for 4-pyridiones is a highly unfavored transformation and, consistent with experiment, O-arylation is favored ($\Delta\Delta G^{\ddagger}$ = 12.5 kcal/mol).

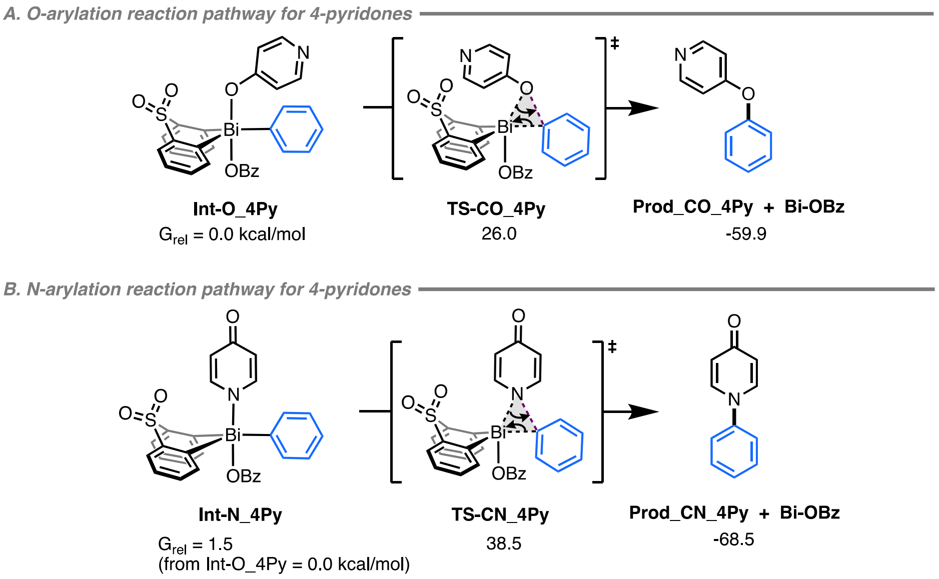

Figure S9. Computed O- (A) and N-arylation (B) reaction pathways with the bismacycle scaffold for 4-pyridione. Free energies of relevant reaction steps calculated using the SMD solvation model (solvent = MeCN) with ωB97XD/def2-QZVPP// ωB97XD/6-31+G(d,p) and def2SVP (Bi) at 80 °C.

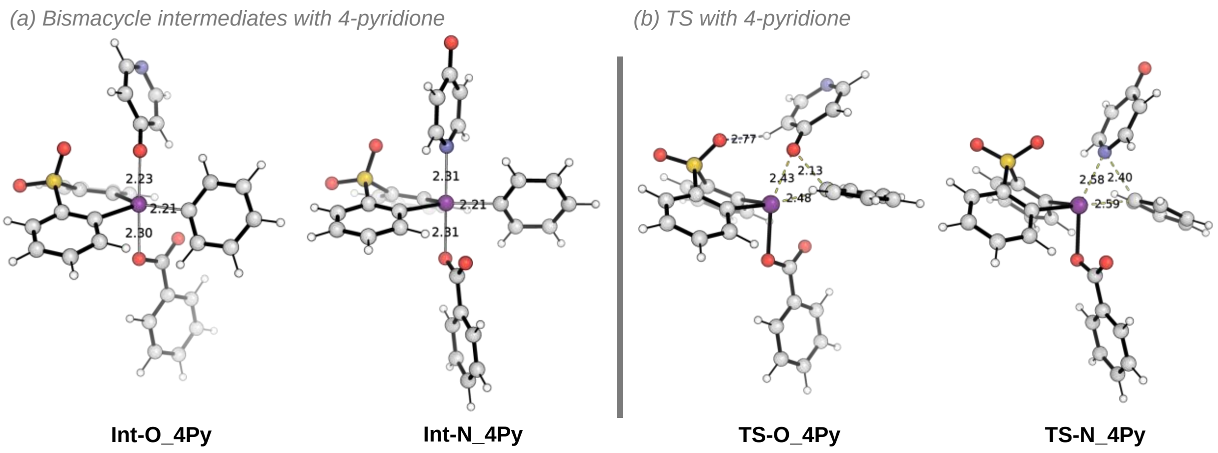

Figure S10. Representations of the Bi(V)-complex ground-state intermediates and TSs for the bismacyclic system with 4-pyridone. Dashed yellow lines show breaking/forming bonds (in Å). The blue dashed lines represent hydrogen interaction lengths in Å.

v. More O'Ferrall–Jencks Plot: High Degree of Concert

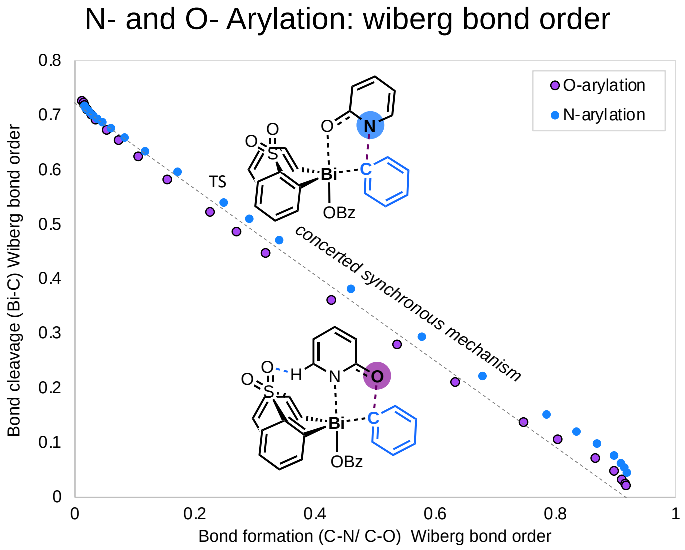

Figure S11. Computed More O'Ferrall-Jencks plots for the Intrinsic Reaction Coordinate (IRC) associated with C- and O-arylation. The calculated Wiberg bond orders reveal a high level of concert with respect to cleavage of the Bi–C bond (*y*-axis) and formation of either C-N or C-O bonds. There is no Meisenheimer dearomatized intermediate formed.

vi. Ph_3BiCl_2-Based Arylation: O- and N-Arylation Pathways

Arylation using the pentavalent organobismuth reagent, Ph_3BiCl_2 (60 °C in THF) was also studied computationally (**Figure S12**).[30][31] Experimentally, this system results in N-arylation. Again, we found 5-membered arylation TSs proceeding from either N- or O-bound pyridone in the apical position. Both reaction pathways (**Figure S12, A and B**) are irreversible, **TS-CO-BiPh$_3$** and **TS-CN-BiPh$_3$**, from the N-bound and O-bound Bi(V) intermediate, respectively. With the N-bound (**Int-N-BiPh$_3$**) and O-bound (**Int-O-BiPh$_3$**) Bi(V) intermediates in equilibrium, applying the Curtin-Hammett principle predicts selectivity in favor of N-arylation ($\Delta\Delta G^{\ddagger}$ = 2.0 kcal/mol). These results agree with the experimental findings using the Ph_3BiCl_2 system.[30][31]

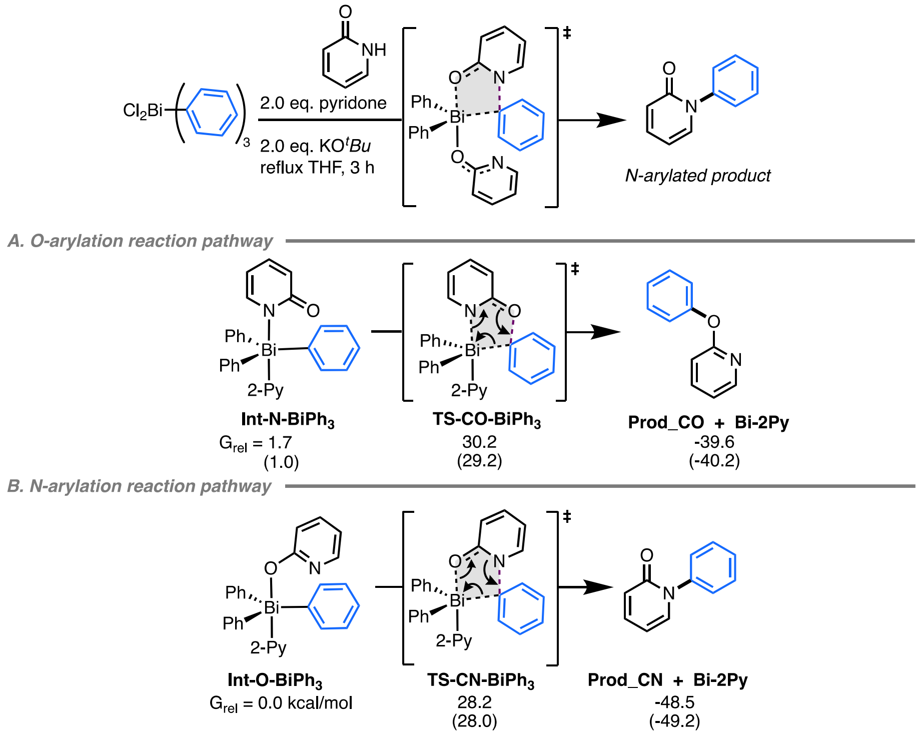

Figure S12. Computed O- (A) and N-arylation (B) reaction pathways with Ph_3BiCl_2. Boltzmann weighted G of relevant reaction steps calculated using the SMD solvation model (solvent = THF) with ωB97XD/def2-QZVPP// ωB97XD/6-31+G(d,p) and def2SVP (Bi) at 60 °C. Gibbs free energy values in parentheses were calculated using (SMD, MeCN) with ωB97XD/def2-QZVPP// ωB97XD/6-31+G(d,p) and def2TZVP (Bi) at 60 °C. 2-Py, 2-pyridyloxy.

vii. Transition State Stability Analysis

We were able to locate both 3- and 5-membered arylation TSs (shown in **Figure S13** and **Figure S15**) for both Bi-mediated arylation systems. In both cases, 5-membered structures are more stable than the corresponding 3-membered TS: for the bismacyclic system by 3.5 kcal/mol and for the Ph_3BiCl_2 mediated reaction by 4.1 kcal/mol. These results illustrate that reactivity through a 3-membered arylation TS would be possible for 4-pyridone (where a 5-membered pathway could not occur), although with a characteristically higher barrier by 3-4 kcal/mol. When in competition, the 5-membered pathway is expected to dominate over the 3-membered pathway. As stated above, the bismacyclic system exhibits selectivity for C-O arylation, whereas the acyclic, Ph_3BiCl_2 system favors C-N arylation, both via 5-membered pathways.

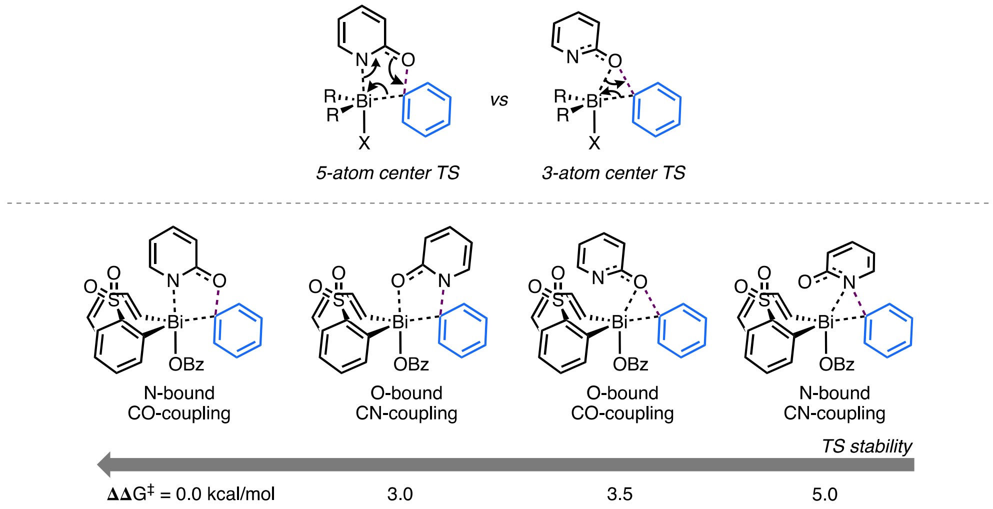

Figure S13. Relative stabilities of O- and N-arylation TSs with 5- and 3-membered cyclic structures. Boltzmann weighted G values were selected for comparison in the stability analysis.

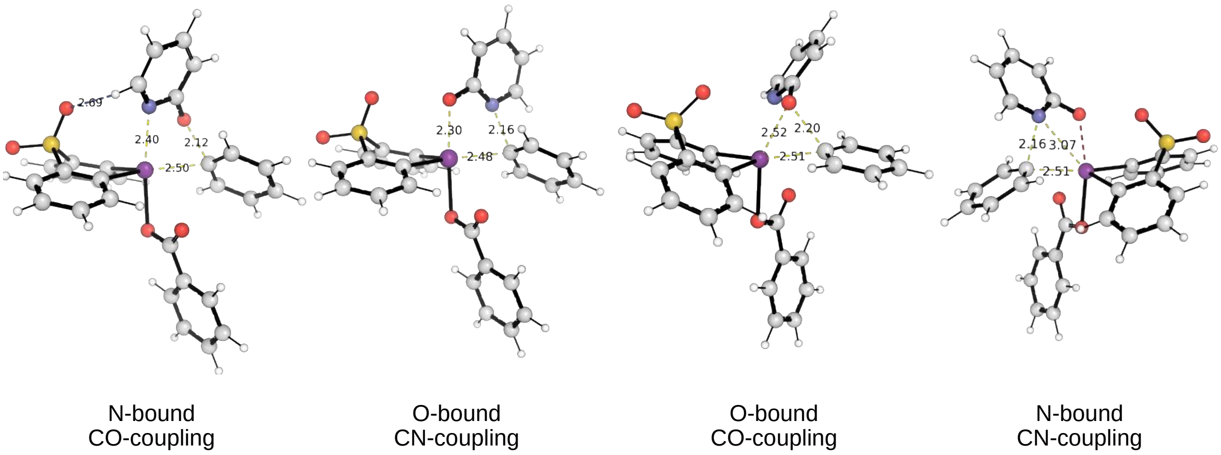

Figure S14. Most stable TS conformers for the bismacyclic system with 2-pyridone. Dashed yellow lines show breaking/forming bonds (in Å). The blue dashed lines represent hydrogen interaction lengths in Å. The red dash line shows a Bi-O interaction (2.35 Å).

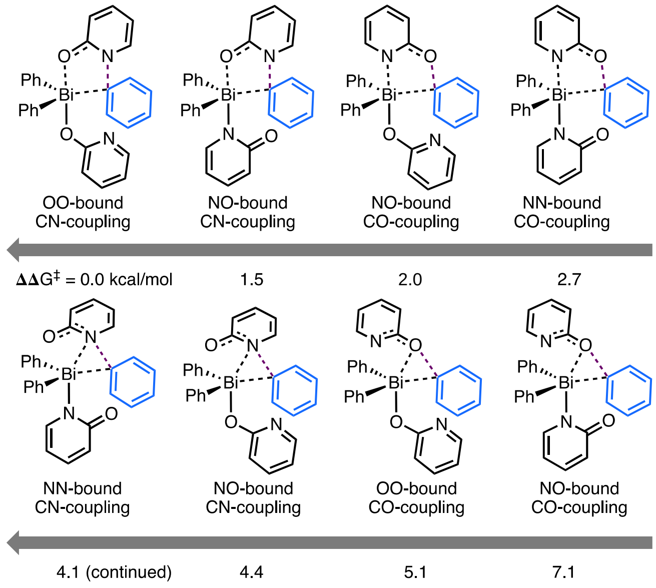

Figure S15. Relative stabilities of O- and N-arylation TSs with 5- and 3-membered cyclic structures.

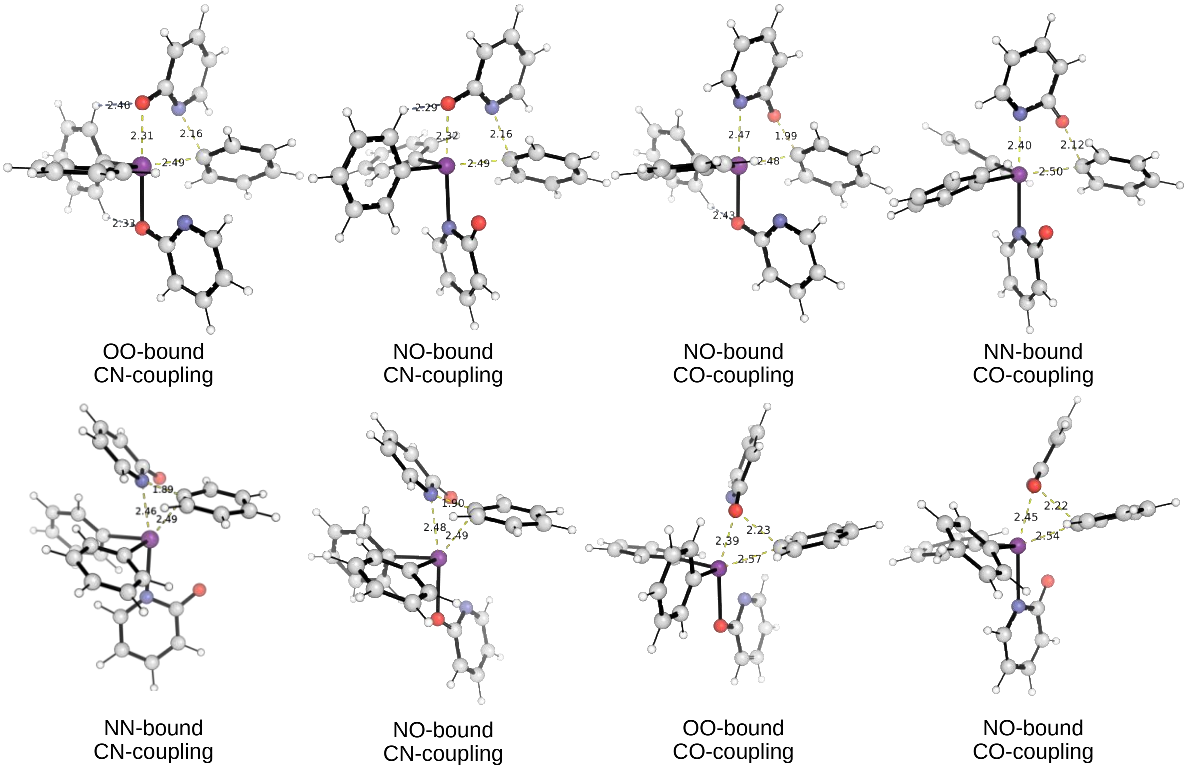

Figure S16. Representations of the most stable TS conformers for the Ph_3BiCl_2 system with 2-pyridone. The yellow dash lines represent bonds forming and breaking with corresponding bond lengths in Å. The blue dashed lines represent hydrogen interaction lengths in Å.

viii. Bi(V) Intermediates

We further investigated the stabilities for the ground-state intermediates for both model systems (shown in **Figure S17**) suggesting the most stable configurations are those that proceed through the observed selective arylated product: **N-bound Int** for the bismacycle system and **OO-bound Int** for the Ph_3BiCl_2.

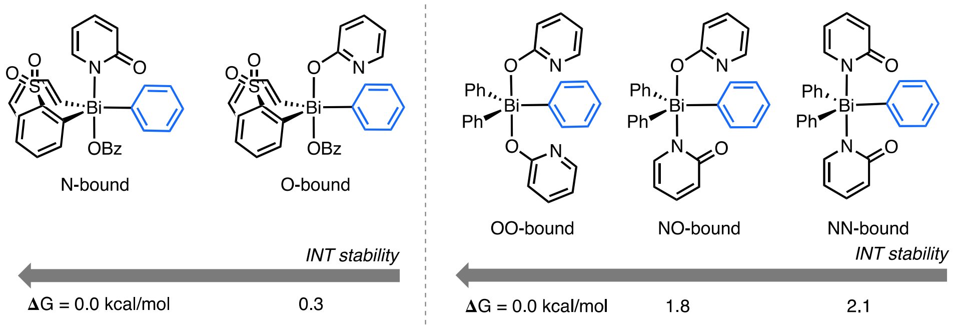

Figure S17. Ground-state Bi(V)-complex intermediates for both the bismacycle (left) and the Ph_3BiCl_2 (right). Boltzmann weighted G values were selected for comparison in the stability analysis.

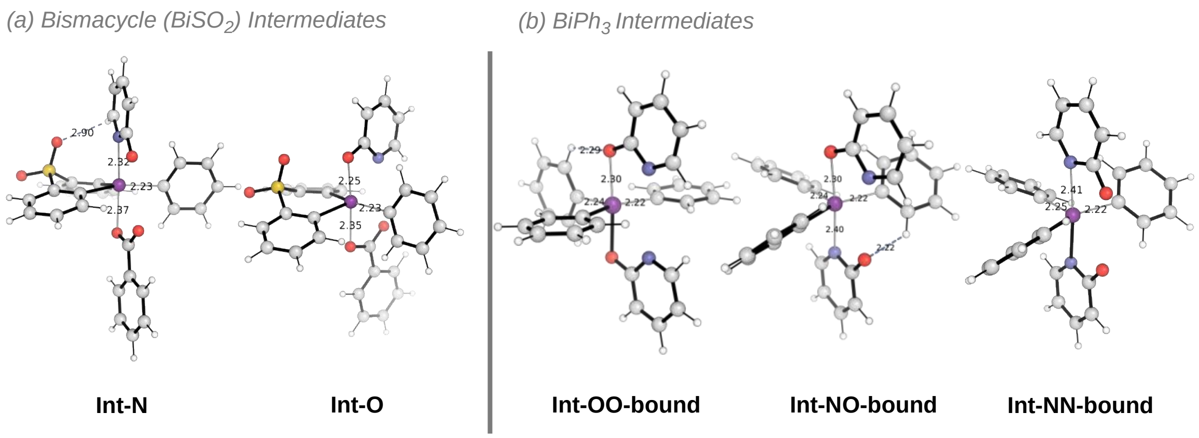

Figure S18. Representations of the most stable ground-state Bi(V)-complex intermediates for both the bismacycle (left) and the Ph_3BiCl_2 (right) with corresponding bond lengths in Å. The blue dashed lines represent hydrogen interaction lengths in Å.

ix. Favorable TS Interactions Determining Selectivity

The influence of axial spectator ion, benzoate (-OBz) and fluoride (-F) was studied (**Figure S19a**). Based on the optimized TS structures, there is a reduction in energy (1.7 kcal/mol) with the fluoride ion, which can be partially explained by the lack of a hydrogen bond interaction between the 2-pyridone and the sulfone group. The spectator ion was also studied in the $BiPh_3$ system between two bound 2-pyridones and the chloride ion, -Cl (shown in **Figure S19c**). In this bismuth system, the second equivalent of 2-pyridone stabilizes and favors the TS of the observed N-selective product by 2 kcal/mol via two strong hydrogen bonding interactions to each 2-pyridone. These interactions are visualized in the ground-state intermediates (**Figure S18**) and transition state structures (**Figure S16**). The role of the sulfone group in the chemoselectivity of the bismacycle system was analyzed by comparing the single-point energy of the removed hydrogen bond interaction and the sulfone group (shown in **Figure S19b**). When the hydrogen bond interaction is removed by deleting the proximal oxygen to 2-pyridone, the selectivity is reduced by 1.1 kcal/mol from the originally optimized N-selective TS (at 3.0 kcal/mol). When the sulfone group is completely removed, the selectivity is reduced by another 1.1 kcal/mol for a total 2.2 kcal/mol. Therefore, we can conclude that the role of both the sulfone group and the axial spectator ion contribute to the stability of the TS, determining the final observed O-selective product.

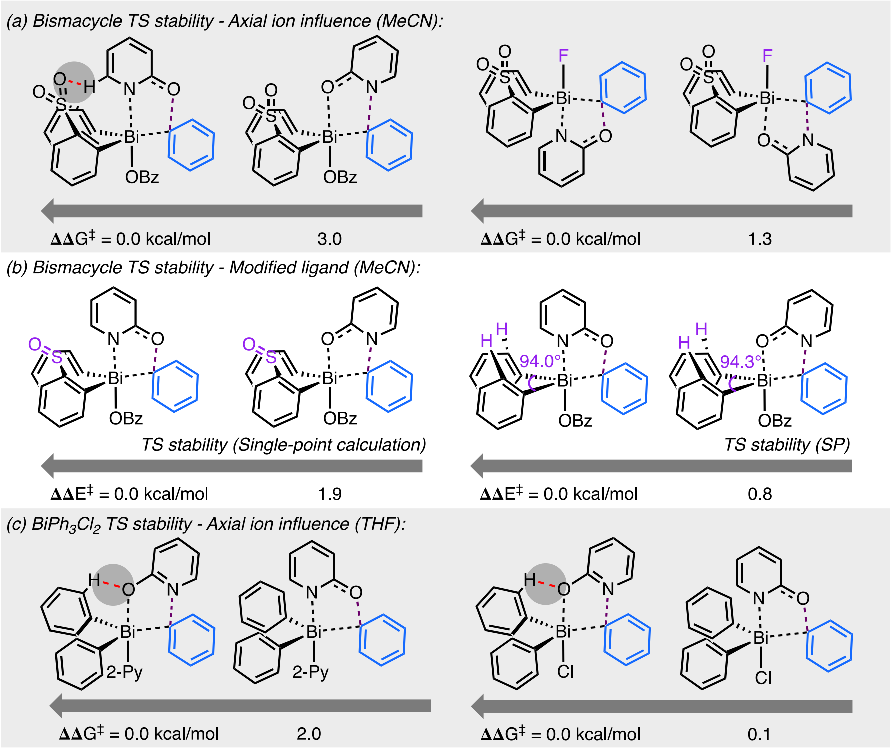

Figure S19. Comparison of the TS stabilities for both the O- and N-arylated products: (a) with benzoate and fluoride ion; (b) with a modified and removed sulfone group; (c) to the $BiPh_3$ model system with two 2-pyridones and chloride ion. The most stable TS energies were selected for comparison for the optimized structures.

x. Comparing Non-Covalent Interactions (NCI) for Both Systems

NCI isodensity plots were generated to visualize favorable interactions at the TSs for the O-arylation in the bismacycle system and N-arylation in the $BiPh_3Cl_2$ system (shown in **Figure S20** as blue dash lines). The TS for O-arylation reveals a hydrogen bond interaction between the 2-pyridone and sulfone while the TS for N-arylation reveals two hydrogen bond interactions to 2-pyridone with the phenyl group. Additionally, a relatively stronger O-Bi attractive interaction resides in the O-arylation TS.

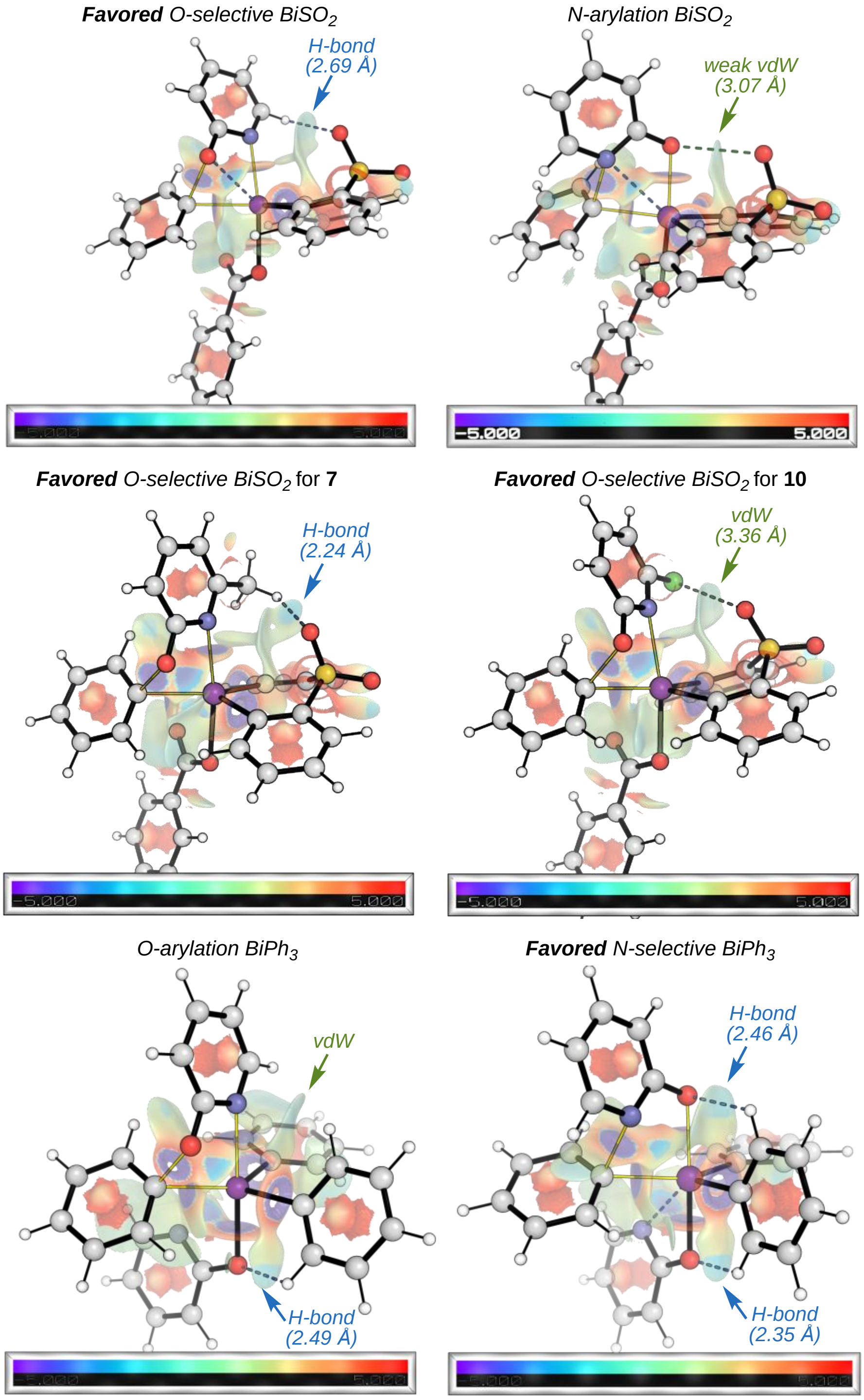

Figure S20. Non-covalent interactions (NCI) representations for the O-arylation in the bismacycle system and N-arylation in the $BiPh_3Cl_2$ system. The yellow lines represent bonds involved in the TSs and the blue dash lines represent attractive interactions.

9. References

[1] P. Petiot, A. Gagnon, *Eur. J. Org. Chem.* **2013**, 5282–5289.

[2] M. Jurrat, L. Maggi, W. Lewis, L. T. Ball, *Nat. Chem.* **2020**, *12*, 260–269.

[3] O. Planas, V. Peciukenas, J. Cornella, *J. Am. Chem. Soc.* **2020**, *142*, 11382–11387.

[4] H. Y. Chen, W. Te Peng, Y. H. Lee, Y. L. Chang, Y. J. Chen, Y. C. Lai, N. Y. Jheng, H. Y. Chen, *Organometallics* **2013**, *32*, 5514–5522.

[5] Z. Zhang, S. Wang, Y. Zhang, G. Zhang, *J. Org. Chem.* **2019**, *84*, 3919–3926.

[6] X. Liang, Y. Li, Q. Xia, L. Cheng, J. Guo, P. Zhang, W. Zhang, Q. Wang, *Green Chem.* **2021**, *23*, 8865–8870.

[7] H. Tanaka, H. Konishi, K. Manabe, *Chem. Lett.* **2019**, *48*, 760–763.

[8] C. B. De Koning, J. P. Michael, J. M. Nhlapo, R. Pathak, W. A. L. Van Otterlo, *Synlett* **2003**, *2003*, 705–707.

[9] M. Genov, A. Almorín, P. Espinet, *Chem. Eur. J.* **2006**, *12*, 9346–9352.

[10] C. C. Lynch, A. Konradi, J. Galemmo, *AMINOPYRIDINE COMPOUNDS AND METHODS FOR THE PREPARATION AND USE THEREOF*, **2018**, CORTEXYME INC, C. C. Lynch, A. Konradi, JR. Galemmo, AMINOPYRIDINE COMPOUNDS AND METHODS FOR THE PREPARATION AND USE THEREOF, WO2018209132 (A1).

[11] C. Zhang, P. Sun, *J. Org. Chem.* **2014**, *79*, 8457–8461.

[12] J. Yao, R. Feng, Z. Wu, Z. Liu, Y. Zhang, *Adv. Synth. Catal.* **2013**, *355*, 1517–1522.

[13] A. T. Londregan, S. Jennings, L. Wei, *Org. Lett.* **2011**, *13*, 1840–1843.

[14] S. D. Schimler, M. S. Sanford, *Synlett* **2016**, *27*, 2279–2284.

[15] R. Takise, R. Isshiki, K. Muto, K. Itami, J. Yamaguchi, *J. Am. Chem. Soc.* **2017**, *139*, 3340–3343.

[16] M. J. R. P. Queiroz, D. Peixoto, R. C. Calhelha, P. Soares, T. Dos Santos, R. T. Lima, J. F. Campos, R. M. V. Abreu, I. C. F. R. Ferreira, M. H. Vasconcelos, *Eur. J. Med. Chem.* **2013**, *69*, 855–862.

[17] K. Kubo, S. Ohyama, T. Shimizu, A. Takami, H. Murooka, T. Nishitoba, S. Kato, M. Yagi, Y. Kobayashi, N. Iinuma, T. Isoe, K. Nakamura, H. Iijima, T. Osawa, T. Izawa, *Bioorg. Med. Chem.* **2003**, *11*, 5117–5133.

[18] P. Hermange, A. T. Lindhardt, R. H. Taaning, K. Bjerglund, D. Lupp, T. Skrydstrup, *J.*

Am. Chem. Soc. **2011**, *133*, 6061–6071.

[19] C. J. Foster, T. Gilkerson, R. Stocker, *Herbicidal Carboxamide Derivatives*, **2002**, EP1207154 (A1), C. Foster, T. Gilkerson, R. Stocker, Herbicidial carboxamide derivatives, EP1207154 (A1).

[20] S. Inoue, Y. Yamane, S. Tsukamoto, H. Azuma, S. Nagao, N. Murai, K. Nishibata, S. Fukushima, K. Ichikawa, T. Nakagawa, N. Hata Sugi, D. Ito, Y. Kato, A. Goto, D. Kakiuchi, T. Ueno, J. Matsui, T. Matsushima, *Bioorg. Med. Chem.* **2021**, *39*, 116137.

[21] M. J. Frisch, G. W. Trucks, H. B. Schlegel, G. E. Scuseria, M. a. Robb, J. R. Cheeseman, G. Scalmani, V. Barone, G. a. Petersson, H. Nakatsuji, X. Li, M. Caricato, a. V. Marenich, J. Bloino, B. G. Janesko, R. Gomperts, B. Mennucci, H. P. Hratchian, J. V. Ortiz, a. F. Izmaylov, J. L. Sonnenberg, Williams, F. Ding, F. Lipparini, F. Egidi, J. Goings, B. Peng, A. Petrone, T. Henderson, D. Ranasinghe, V. G. Zakrzewski, J. Gao, N. Rega, G. Zheng, W. Liang, M. Hada, M. Ehara, K. Toyota, R. Fukuda, J. Hasegawa, M. Ishida, T. Nakajima, Y. Honda, O. Kitao, H. Nakai, T. Vreven, K. Throssell, J. a. Montgomery Jr., J. E. Peralta, F. Ogliaro, M. J. Bearpark, J. J. Heyd, E. N. Brothers, K. N. Kudin, V. N. Staroverov, T. a. Keith, R. Kobayashi, J. Normand, K. Raghavachari, a. P. Rendell, J. C. Burant, S. S. Iyengar, J. Tomasi, M. Cossi, J. M. Millam, M. Klene, C. Adamo, R. Cammi, J. W. Ochterski, R. L. Martin, K. Morokuma, O. Farkas, J. B. Foresman, D. J. Fox, **2016**, Gaussian 16, Revision C.01, Gaussian, Inc., Wallin.

[22] J.-D. Chai, M. Head-Gordon, *Phys. Chem. Chem. Phys.* **2008**, *10*, 6615–6620.

[23] A. V. Marenich, C. J. Cramer, D. G. Truhlar, *J. Phys. Chem. B* **2009**, *113*, 6378–6396.

[24] A. N. Bootsma, S. Wheeler, *ChemRxiv* **2019**, 10.26434/chemrxiv.8864204.v5.

[25] E. D. Glendening, J. K. Badenhoop, A. E. Reed, J. E. Carpenter, J. A. Bohmann, C. M. Morales, P. Karafiloglou, C. R. Landis, F. Weinhold, *NBO 7.0*, Theoretical Chemistry Institute, **2018**.

[26] R. A. Boto, F. Peccati, R. Laplaza, C. Quan, A. Carbone, J.-P. Piquemal, Y. Maday, J. Contreras-Garcia, *NCIPLOT4: A new step towards a fast quantification of noncovalent interactions*.

[27] Schrödinger LLC, *The PyMOL Molecular Graphics System, version 1.8*, **2015**.

[28] Paton Lab Workflows. https://github.com/patonlab/paton_group_workflows .

[29] G. Luchini, J. Alegre-Requena, I. Funes-Ardoiz, R. Paton, GoodVibes: automated thermochemistry for heterogeneous computational chemistry data [version 1; peer review: 2 approved with reservations]. *F1000Research* **2020**, *9 (291)*.

[30] K. Ikegai, T. Mukaiyama, *Chem. Lett.* **2005**, *34*, 1496–1497.

[31] K. Ikegai, Y. Nagata, T. Mukaiyama, *Bull. Chem. Soc. Jpn.* **2006**, *79*, 761–767.

10. NMR Spectra

S1 - ^{1}H NMR (500 MHz, $CDCl_3$):

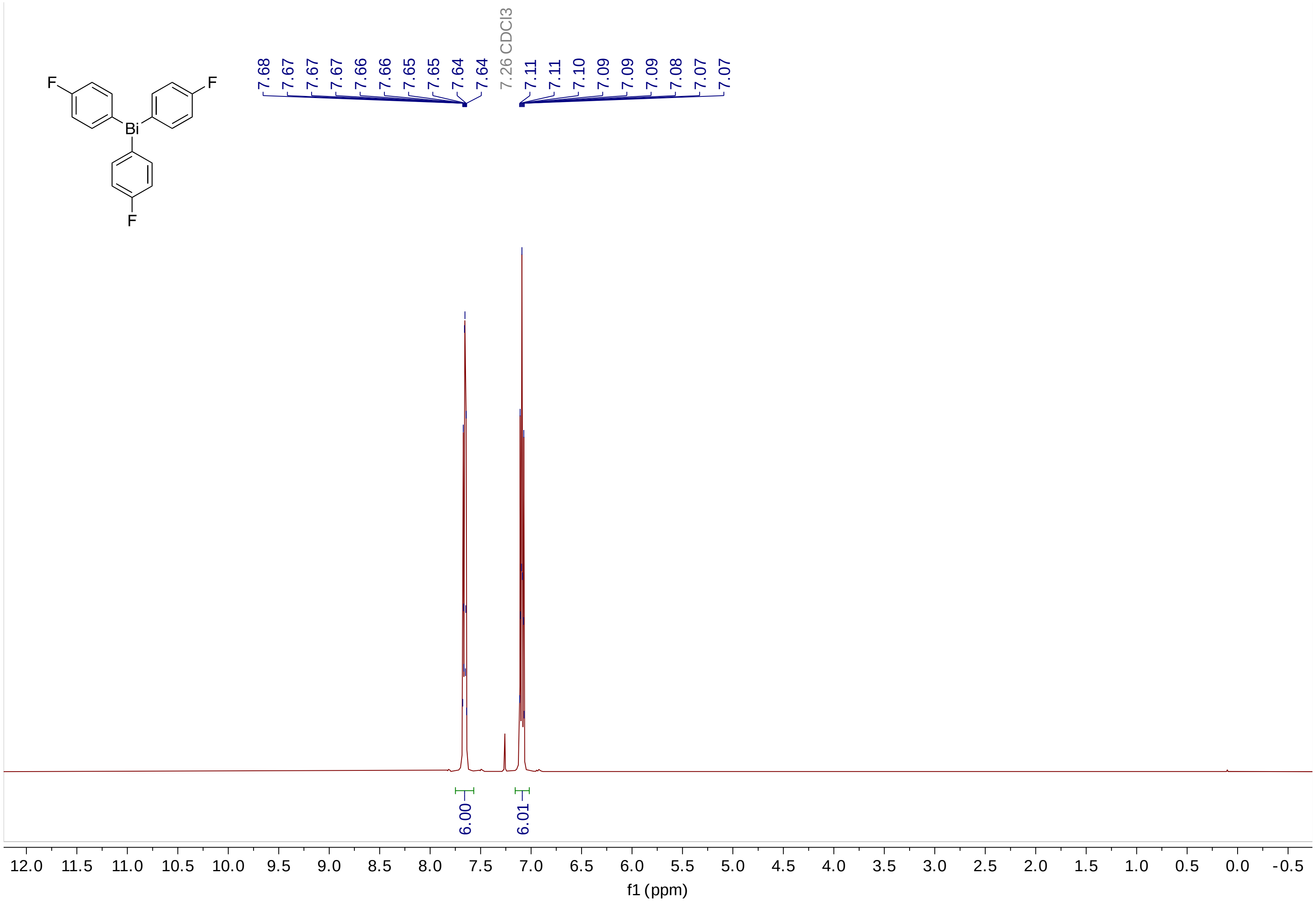

S1 - $^{13}C\{^{1}H\}$ NMR (126 MHz, $CDCl_3$):

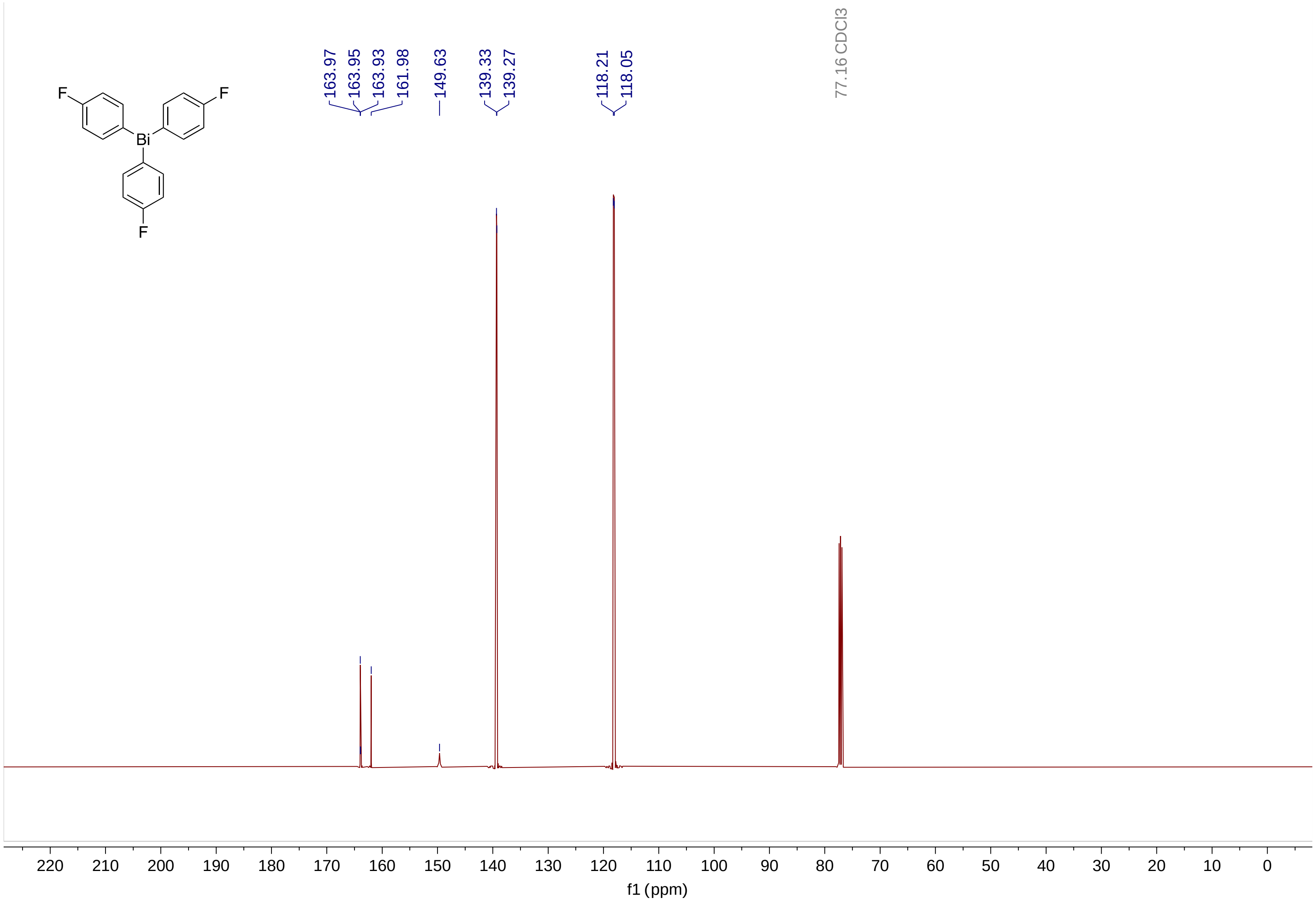

S1 - ^{19}F NMR (471 MHz, $CDCl_3$):

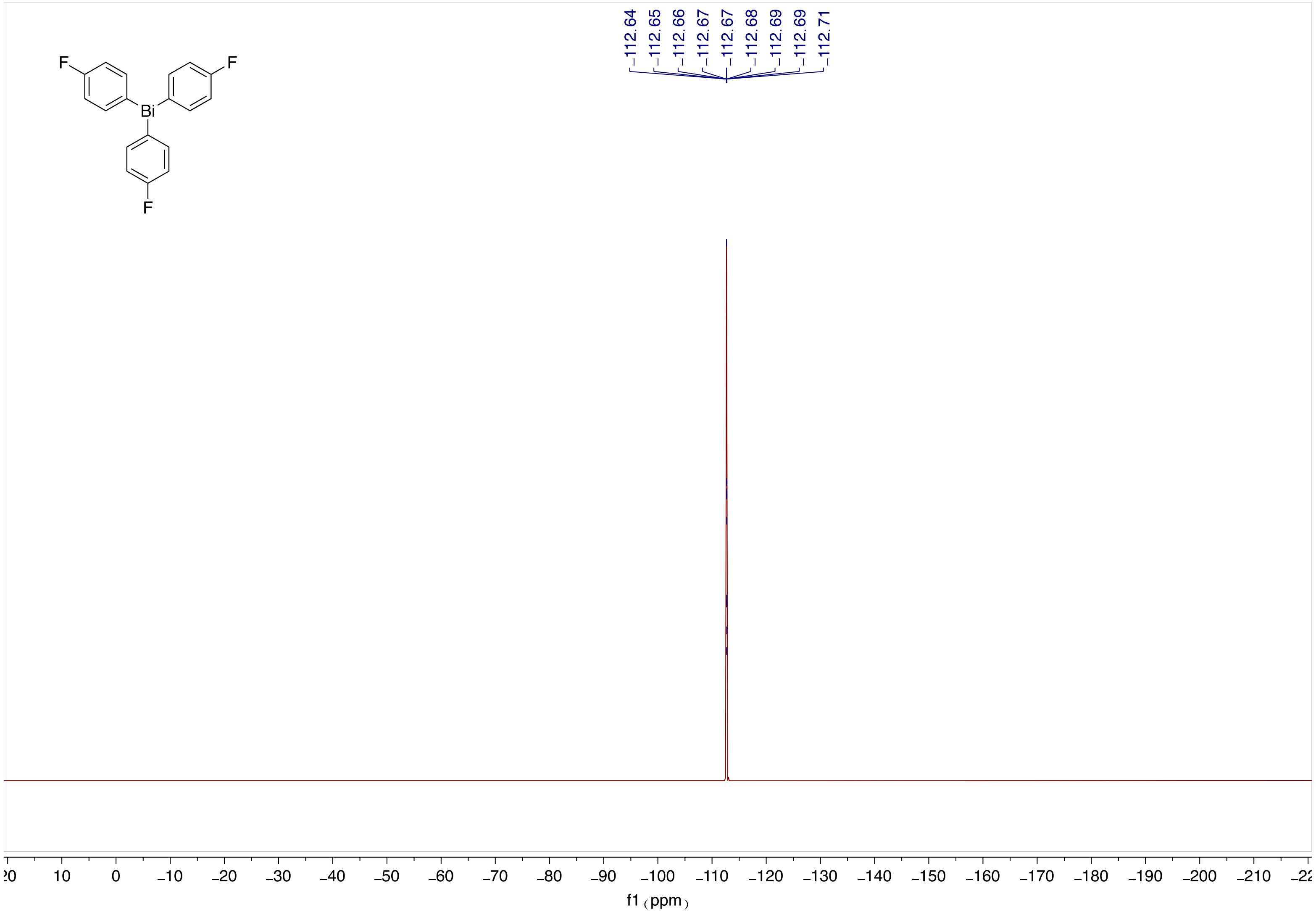

1 - ^{1}H NMR (400 MHz, $CDCl_3$):

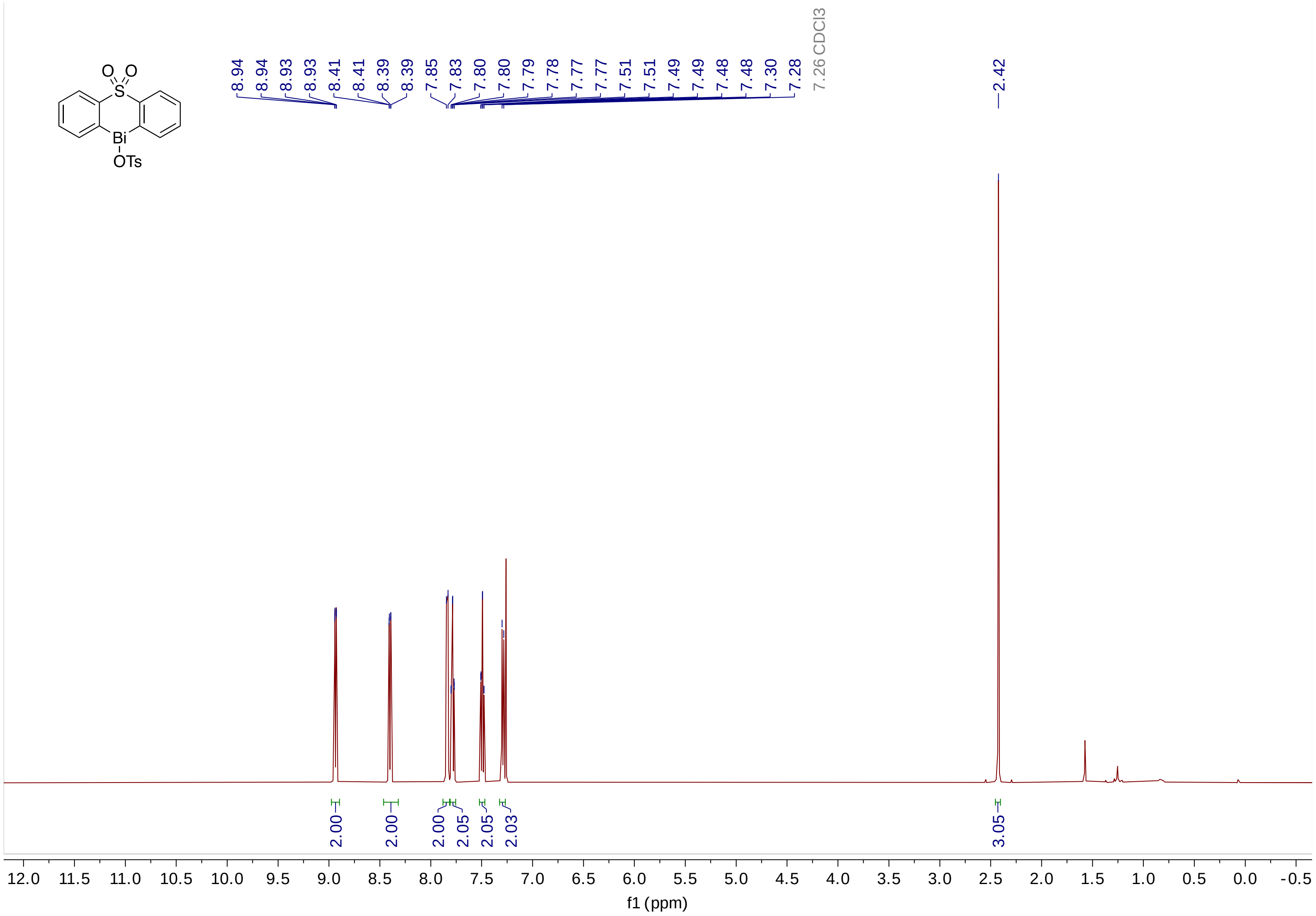

1 - $^{13}C\{^{1}H\}$ NMR (126 MHz, $CDCl_3$):

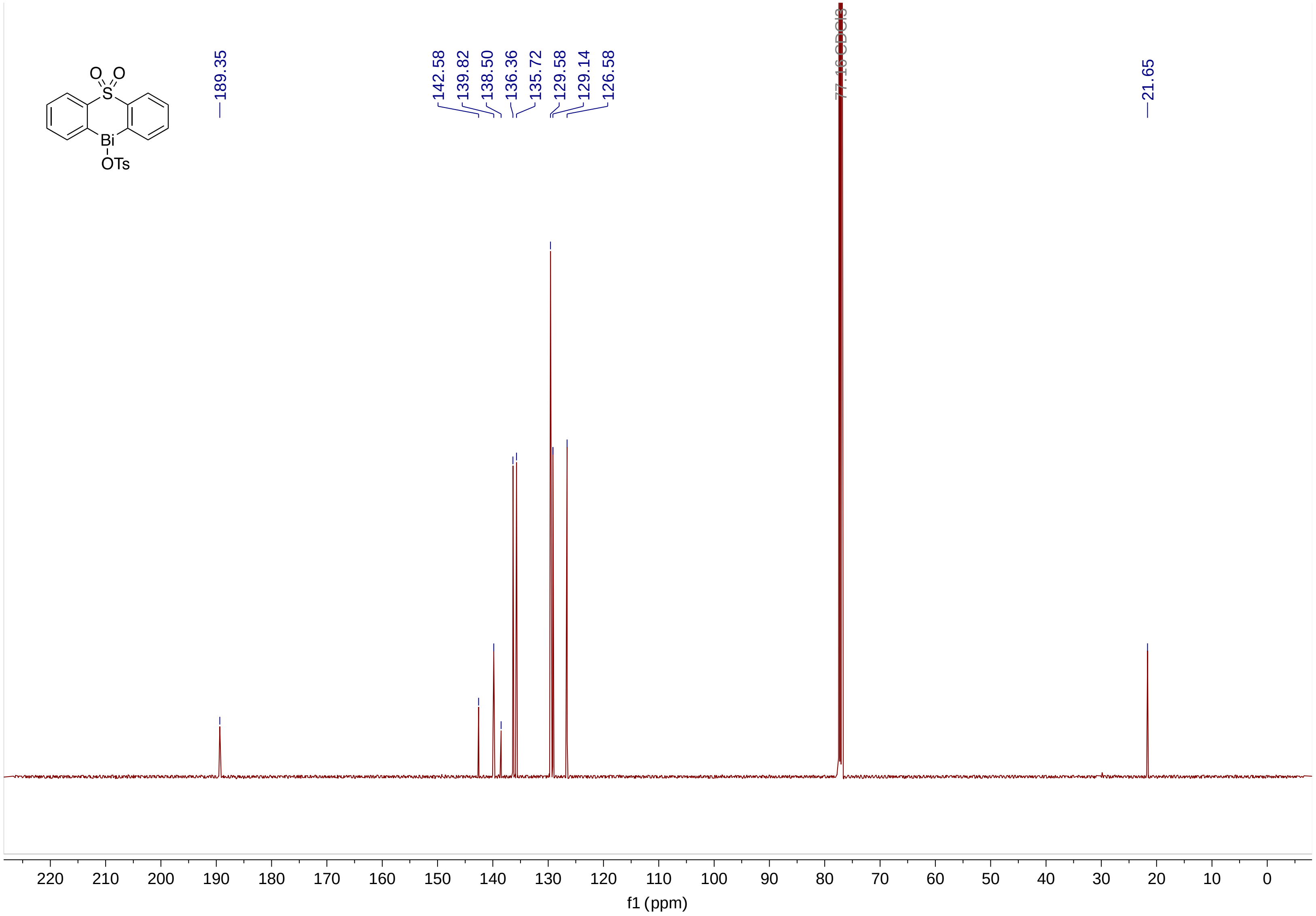

2a - ^{1}H NMR (500 MHz, CDCl3):

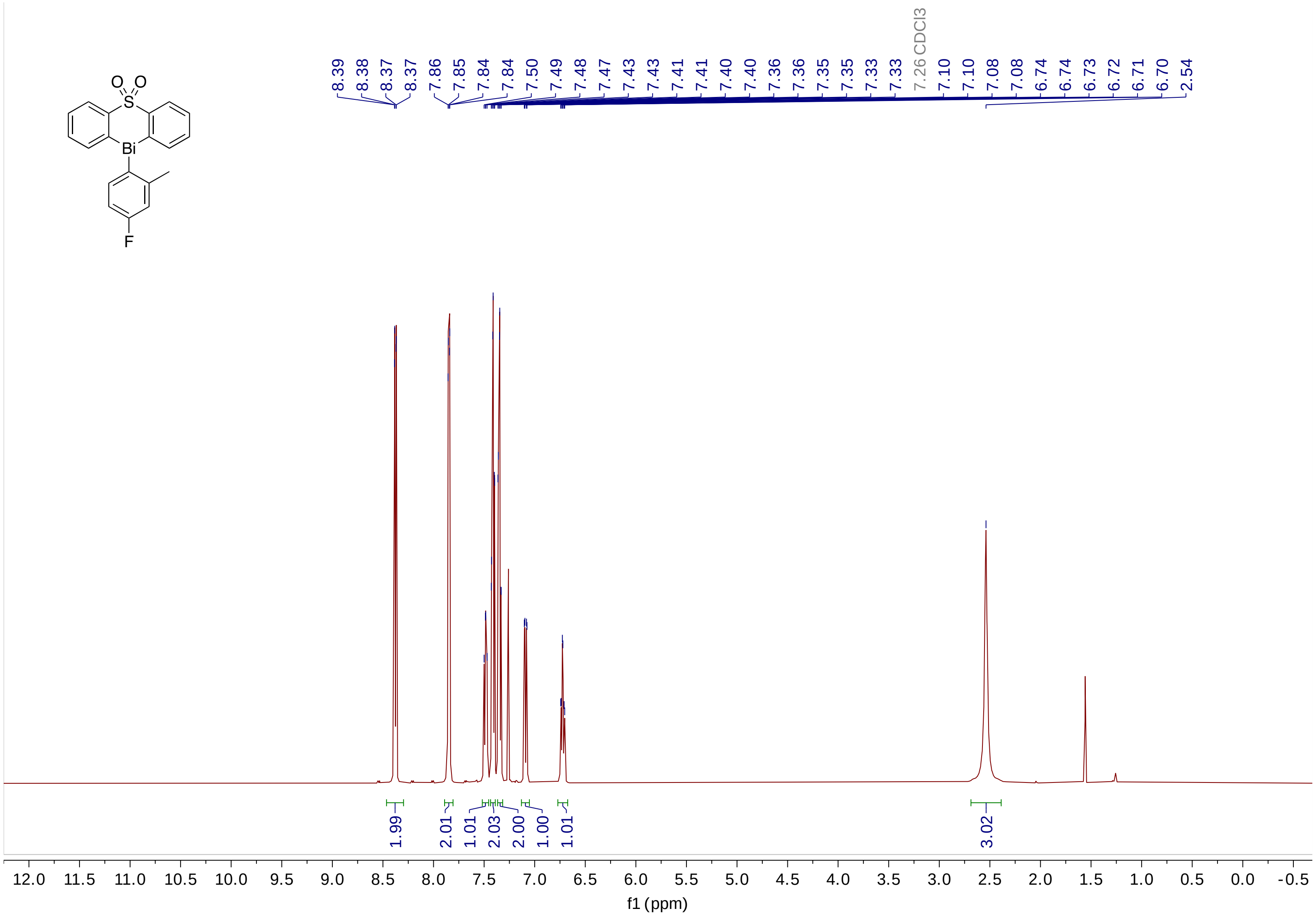

2a - $^{13}C\{^1H\}$ NMR (126 MHz, CDCl$_3$):

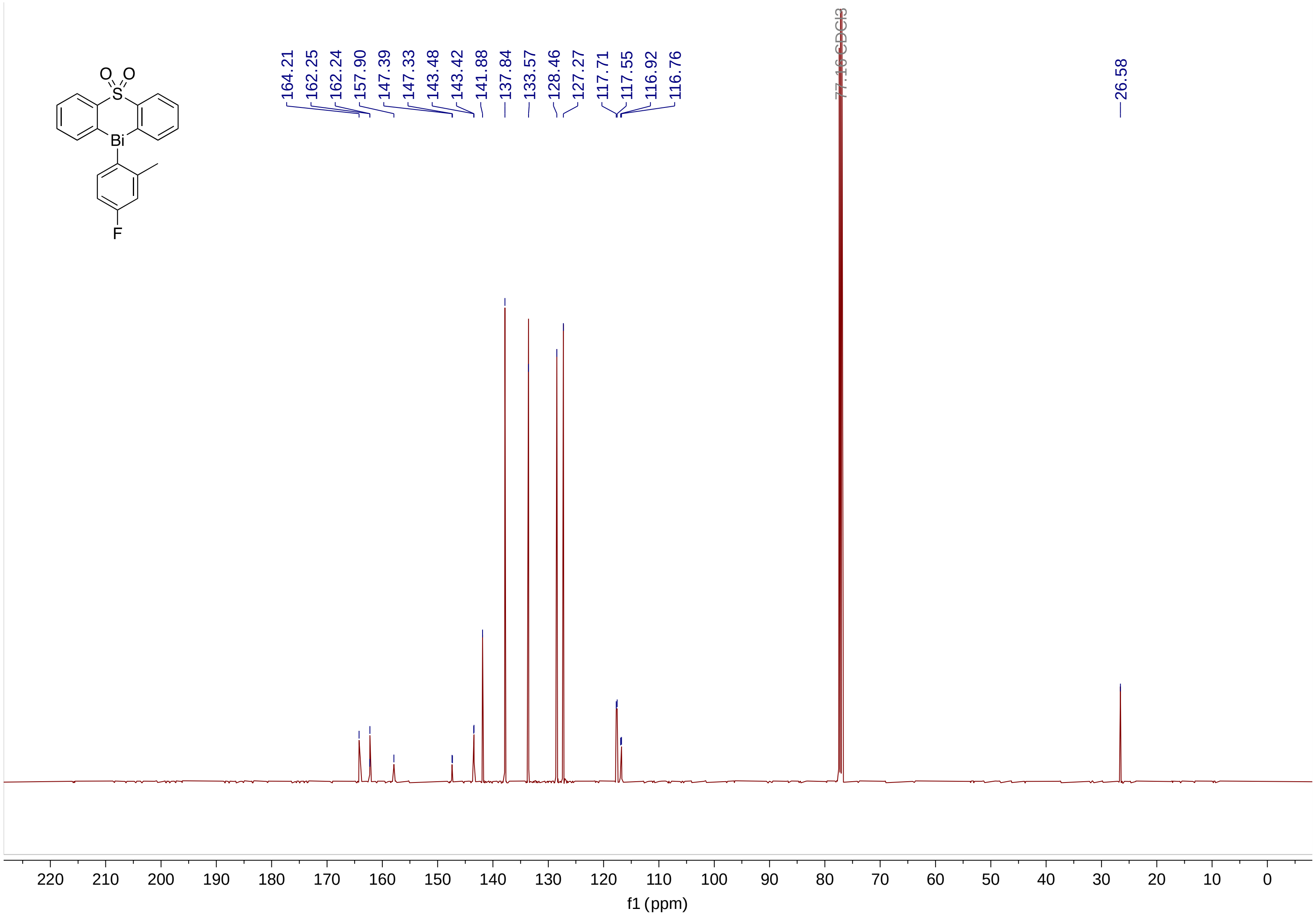

2a - ^{19}F NMR (471 MHz, $CDCl_3$):

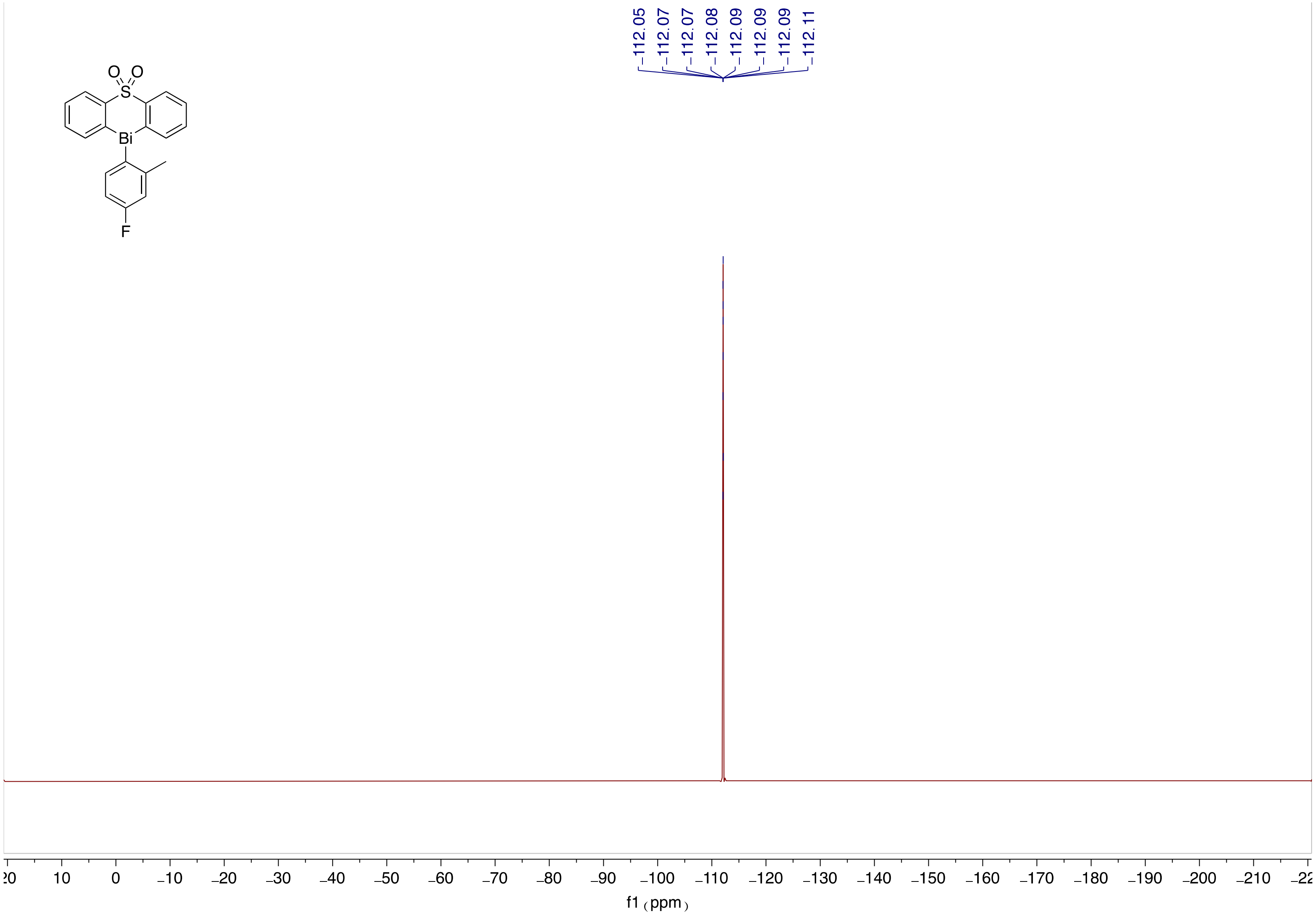

2b - ^{1}H NMR (500 MHz, $CDCl_3$):

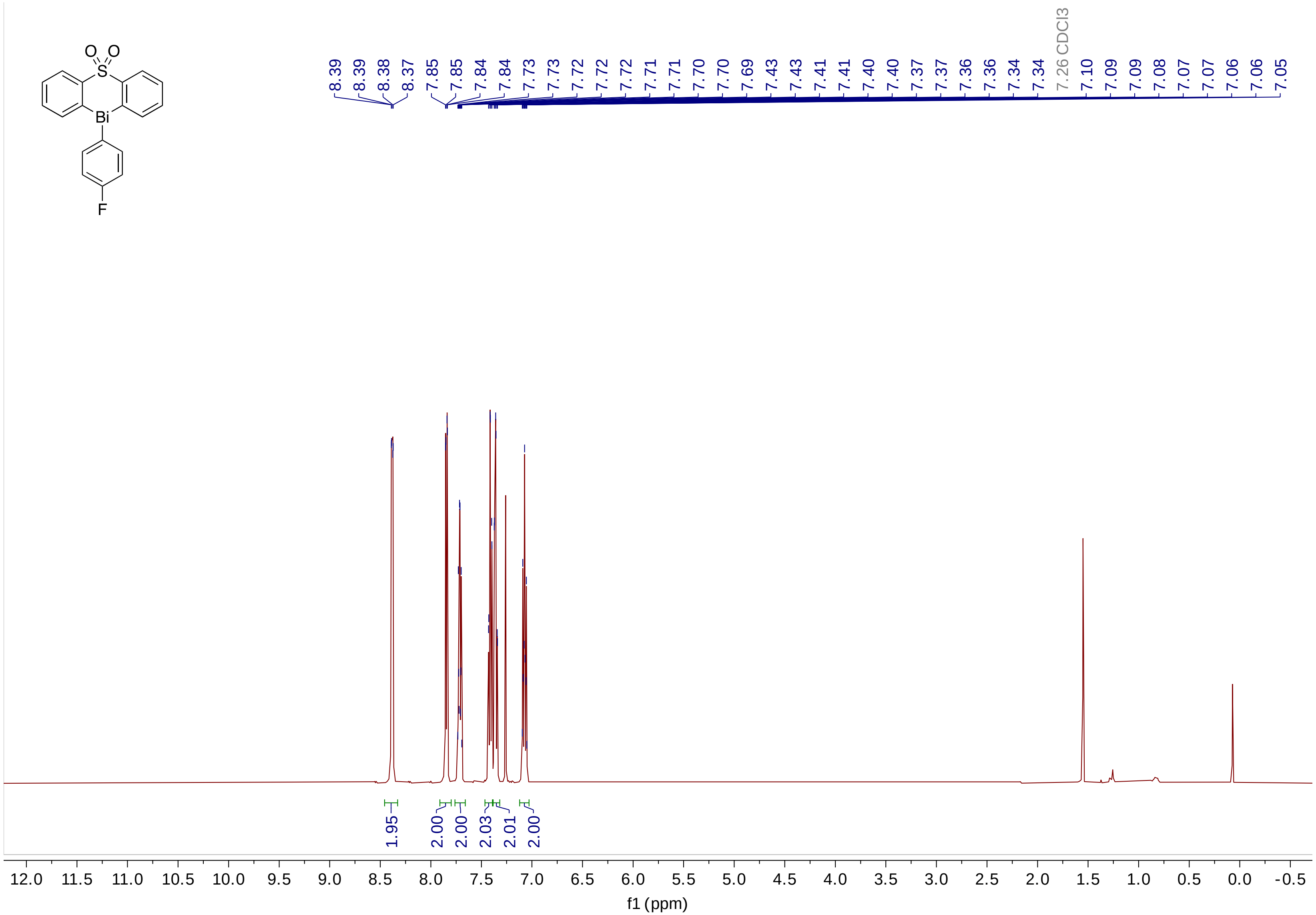

2b - $^{13}C\{^{1}H\}$ NMR (126 MHz, $CDCl_3$):

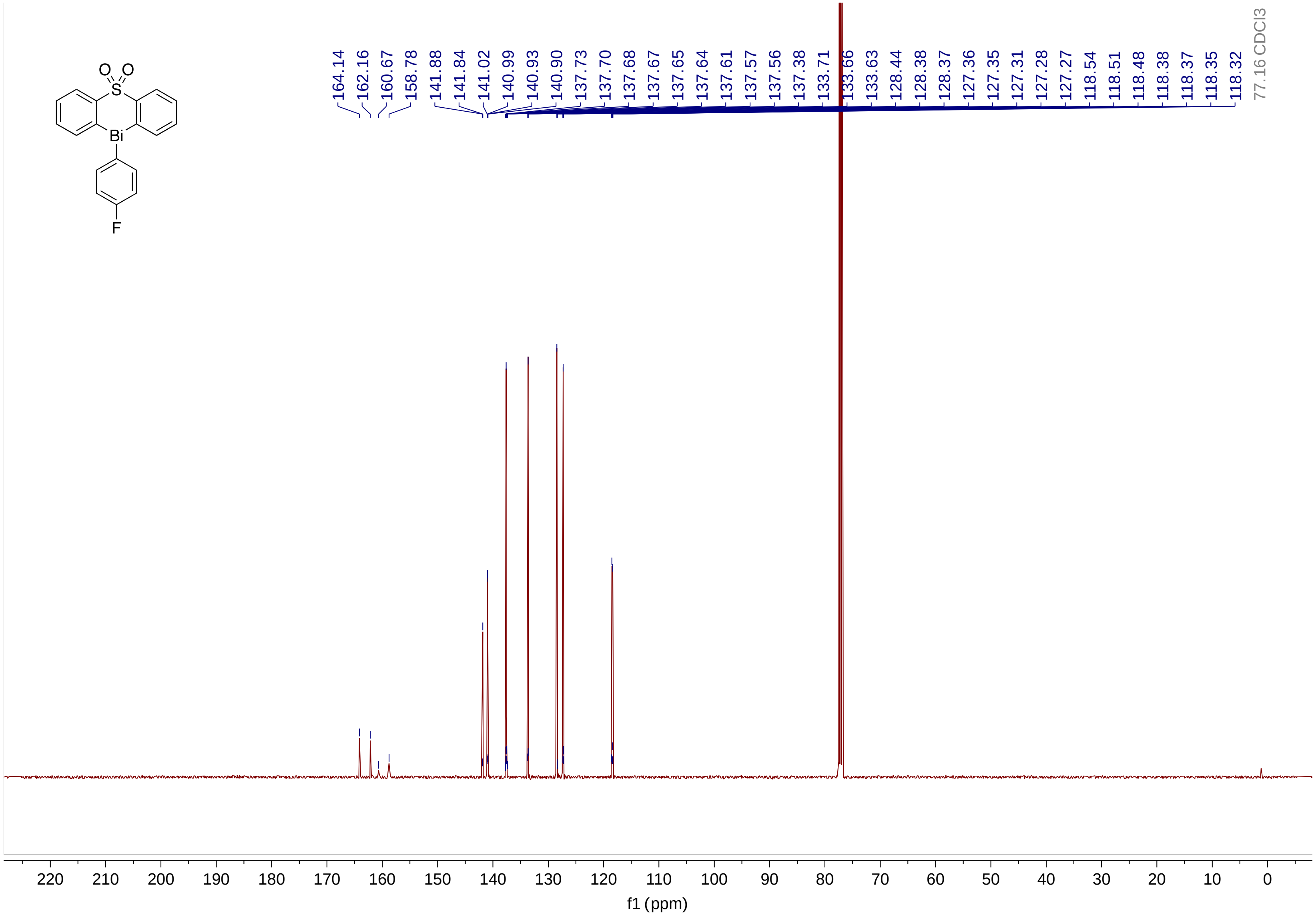

2b - ^{19}F NMR (471 MHz, $CDCl_3$):

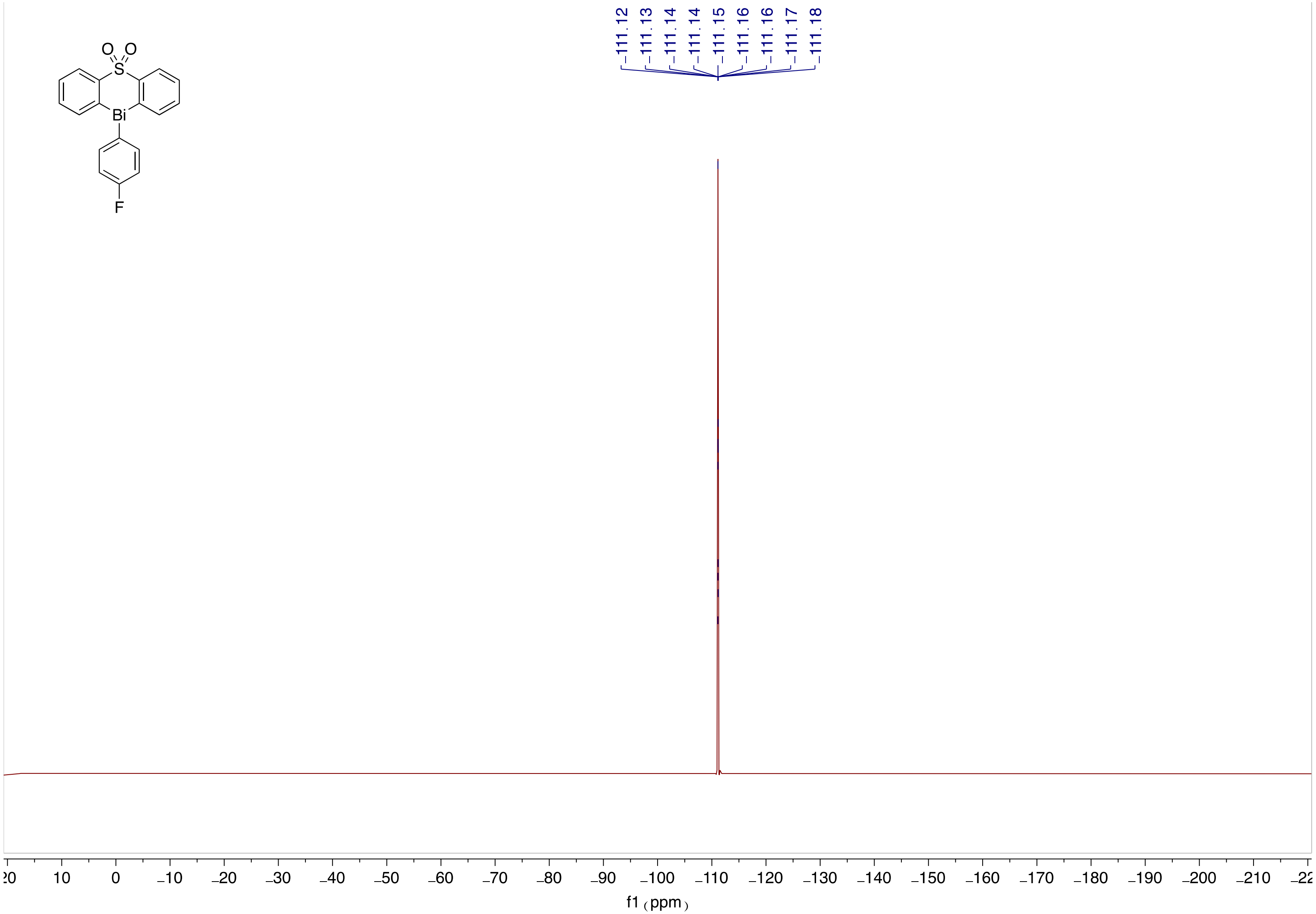

L1 - ^{1}H NMR (400 MHz, $CDCl_3$):

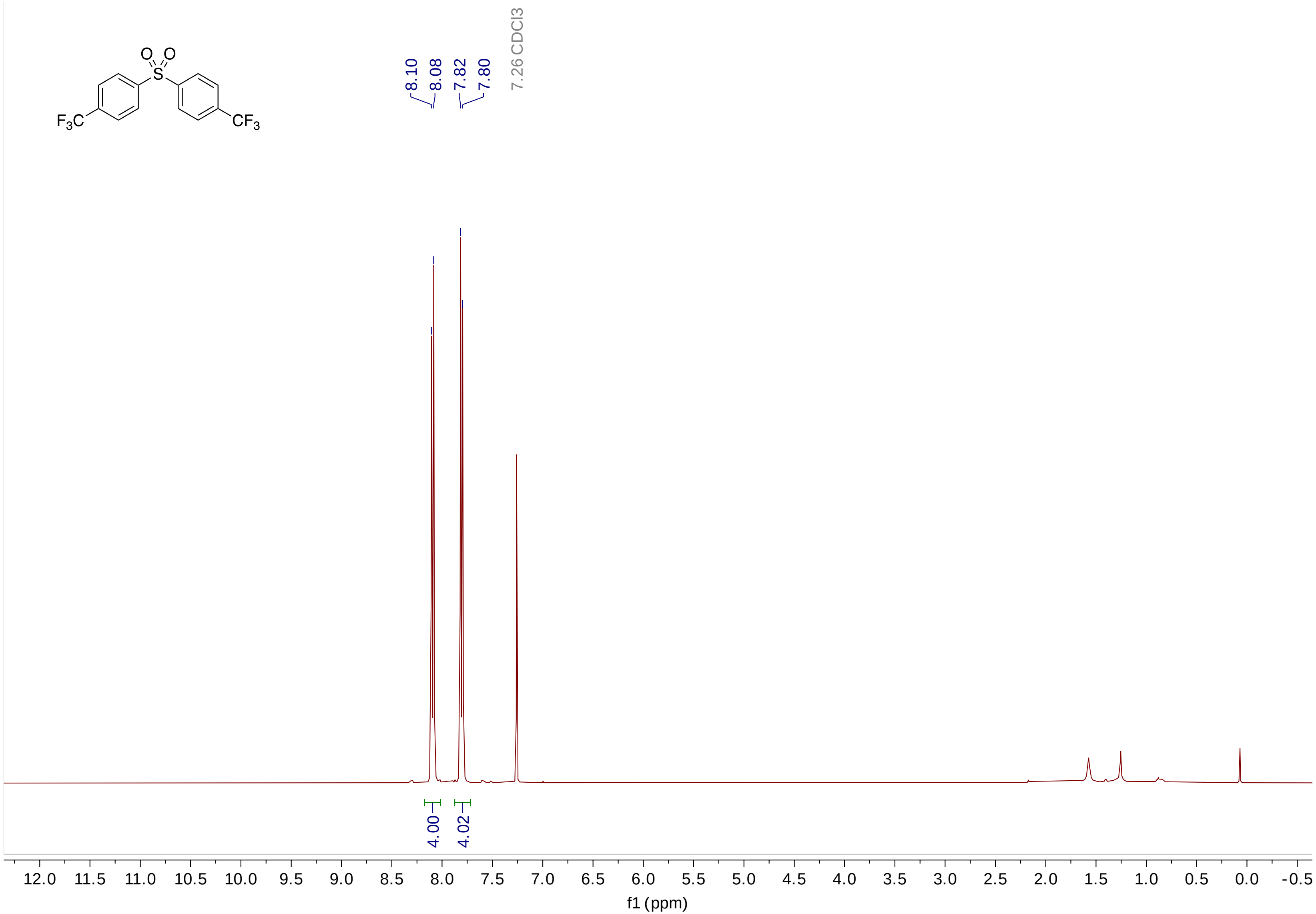

L1 - $^{13}C\{^{1}H\}$ NMR (101 MHz, $CDCl_3$):

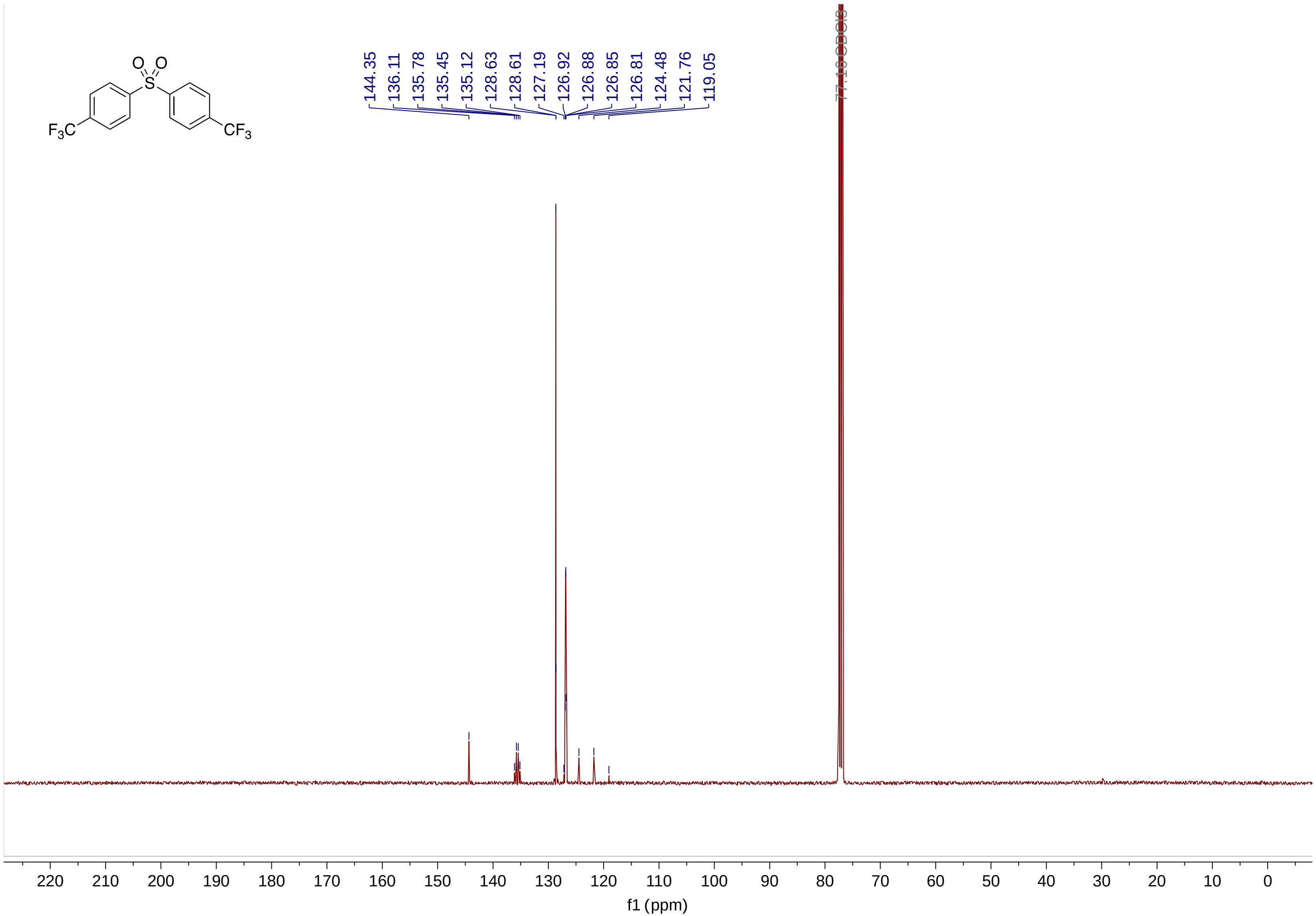

L1 - ^{19}F NMR (377 MHz, $CDCl_3$):

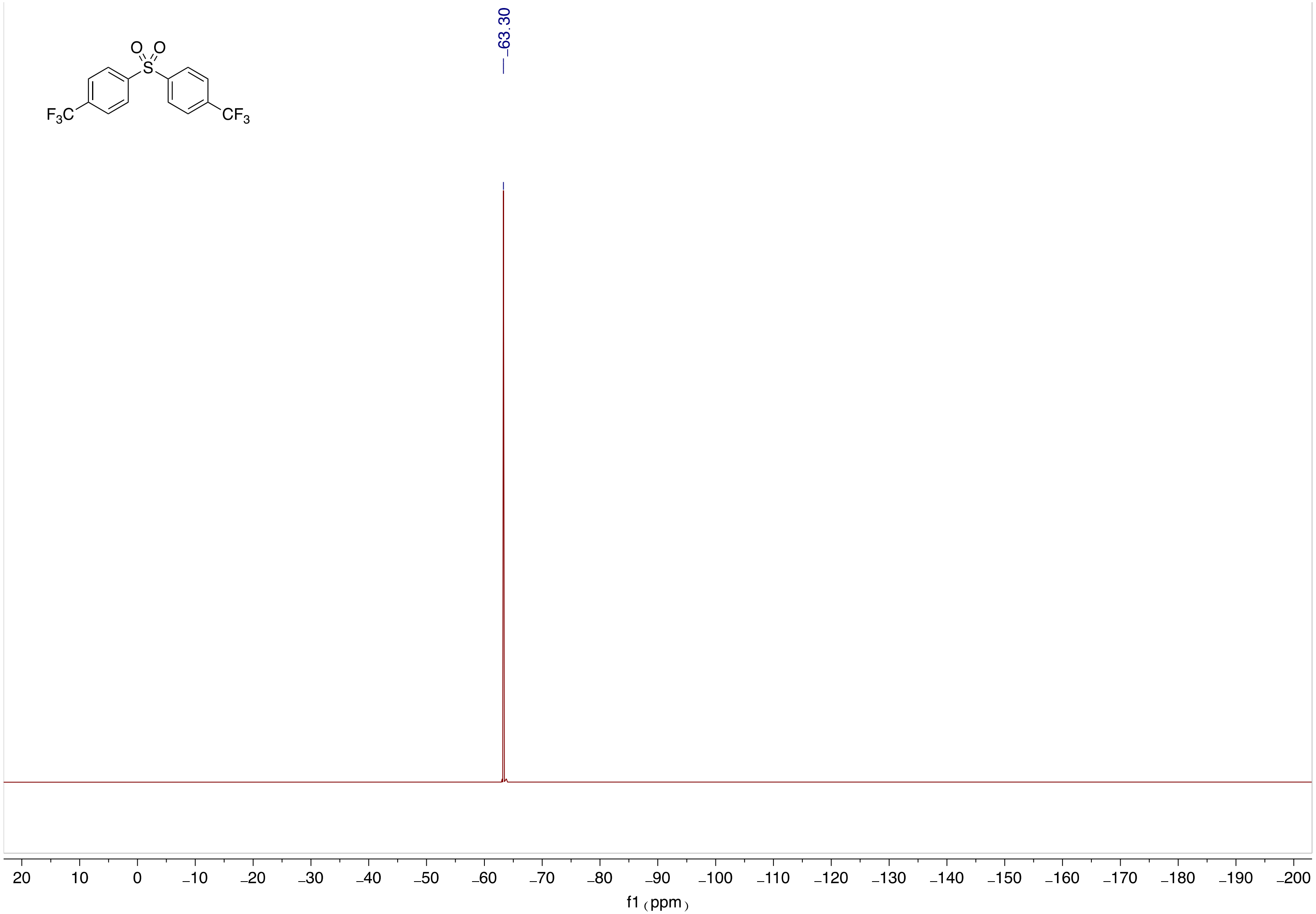

L2 - ^{1}H NMR (400 MHz, $CDCl_3$):

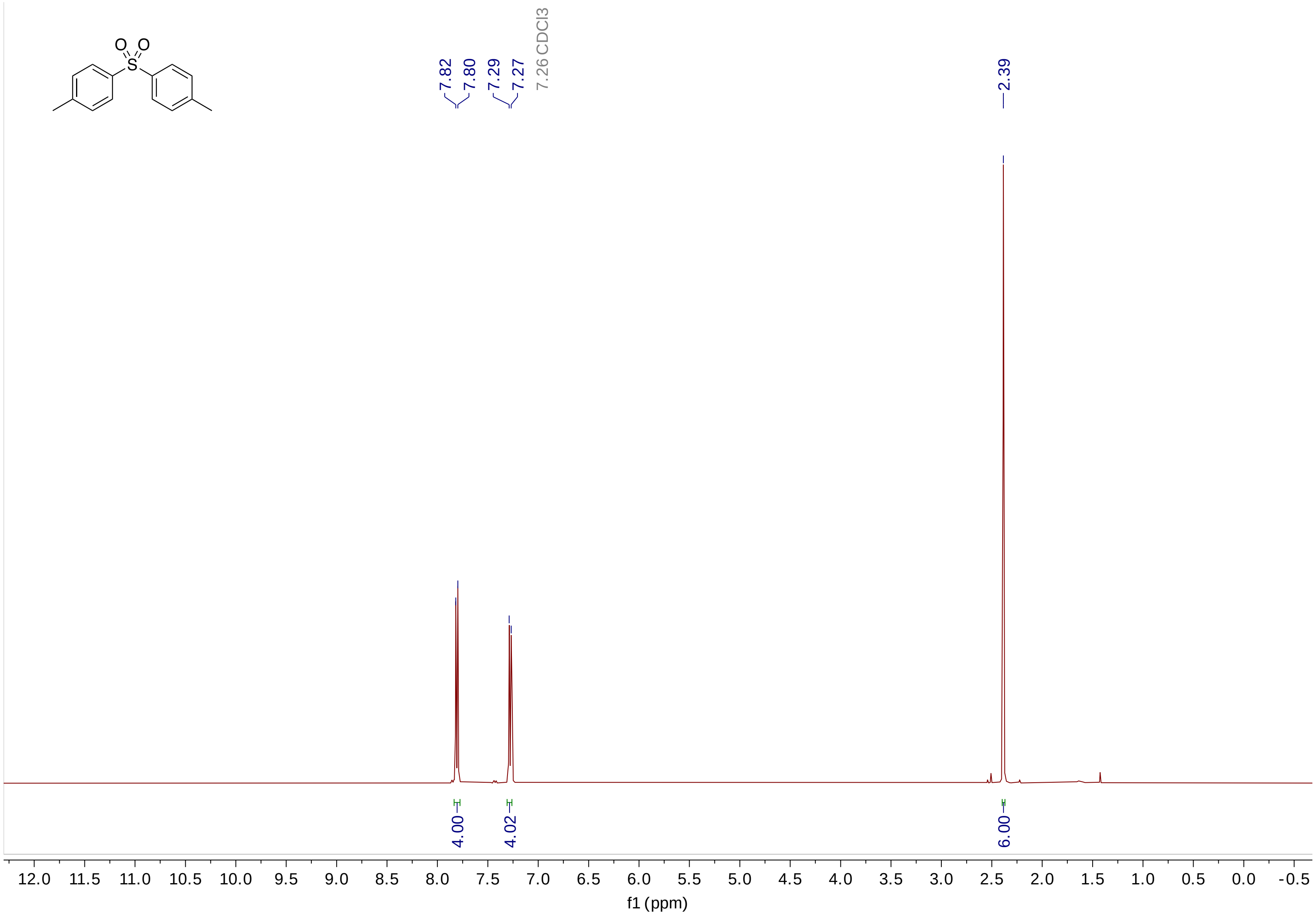

L2 - $^{13}C\{^{1}H\}$ NMR (101 MHz, $CDCl_3$):

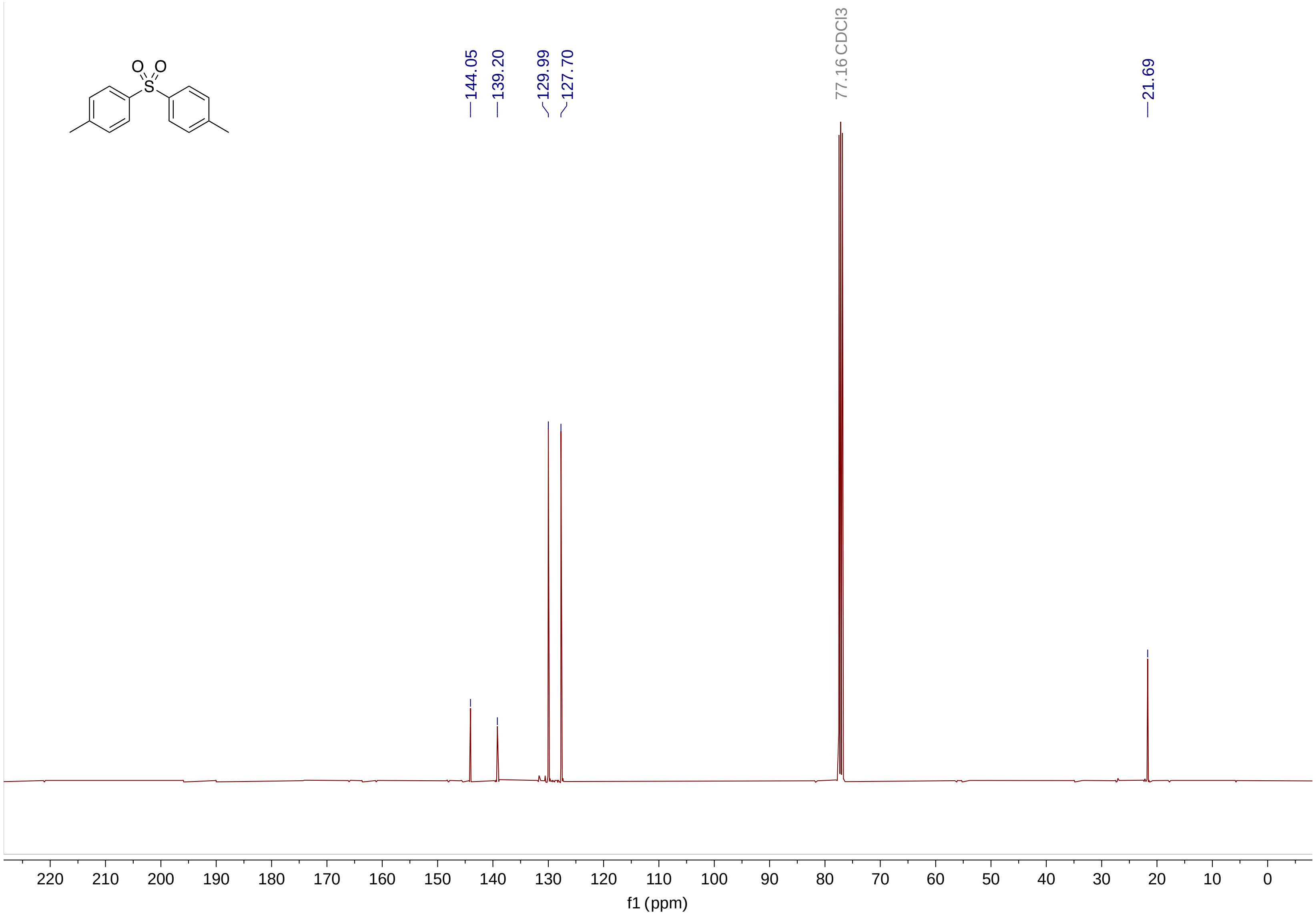

L3 - ^{1}H NMR (400 MHz, $CDCl_3$):

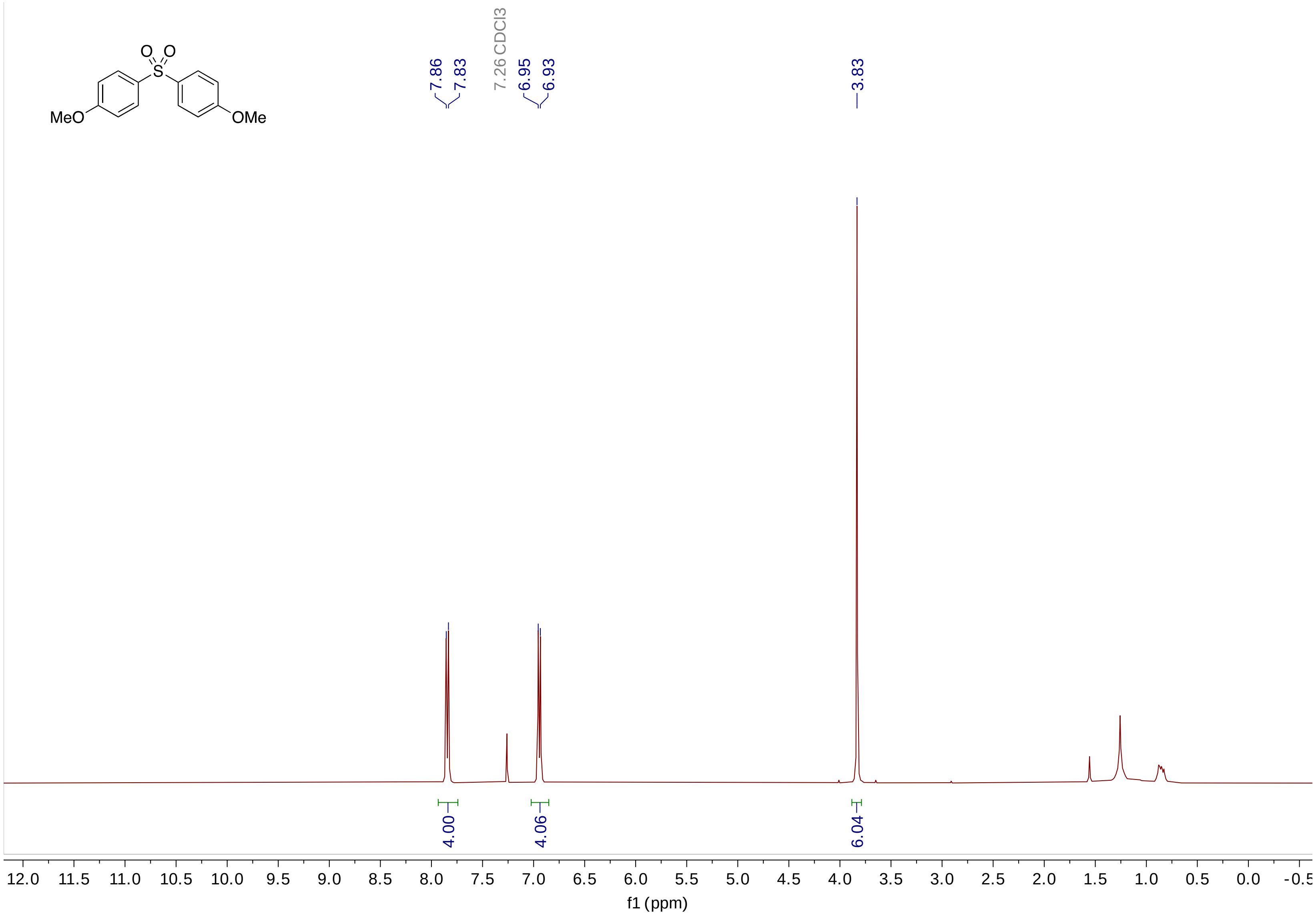

L3 - $^{13}C\{^1H\}$ NMR (101 MHz, $CDCl_3$):

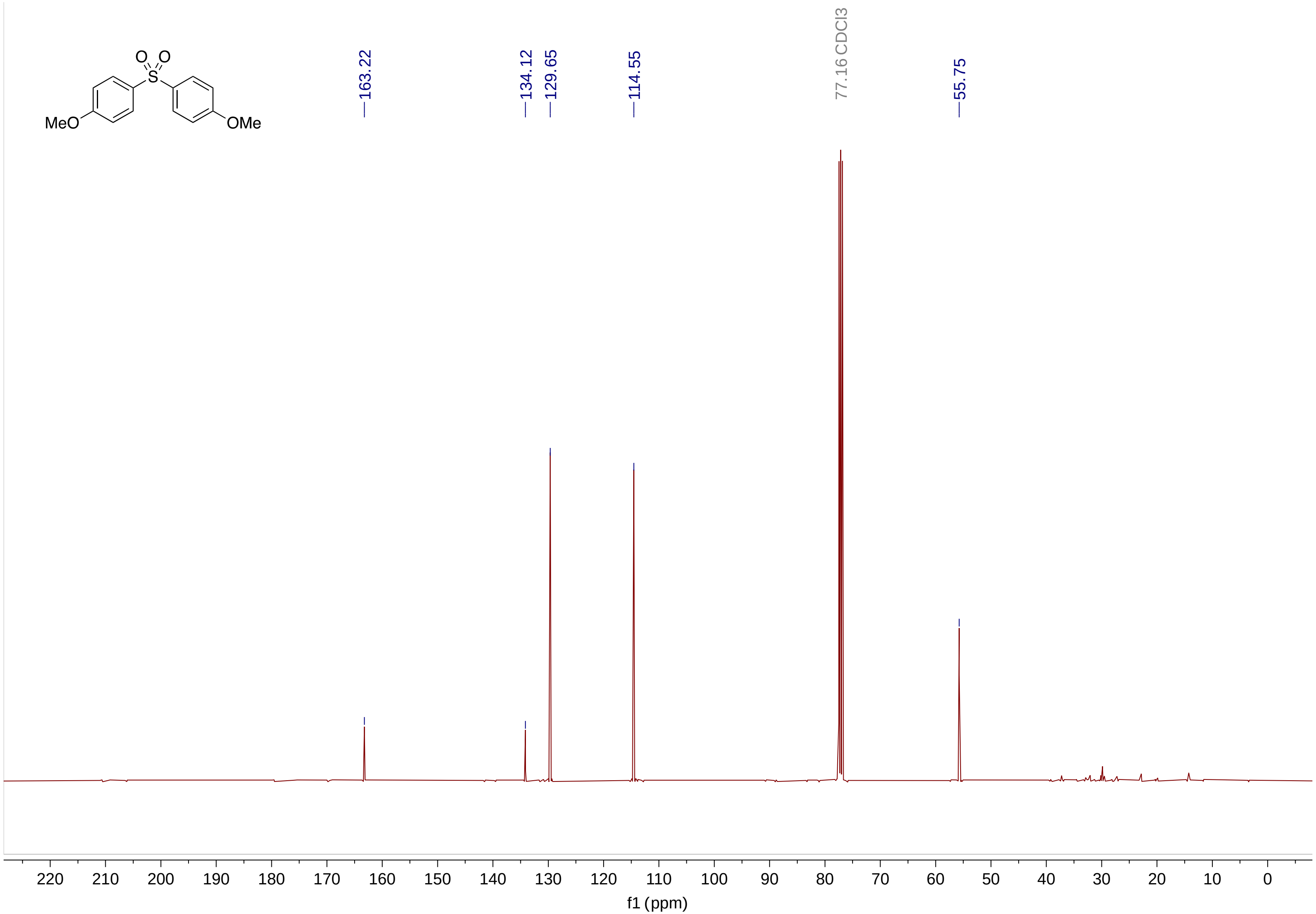

L4 - ^{1}H NMR (400 MHz, CDCl₃):

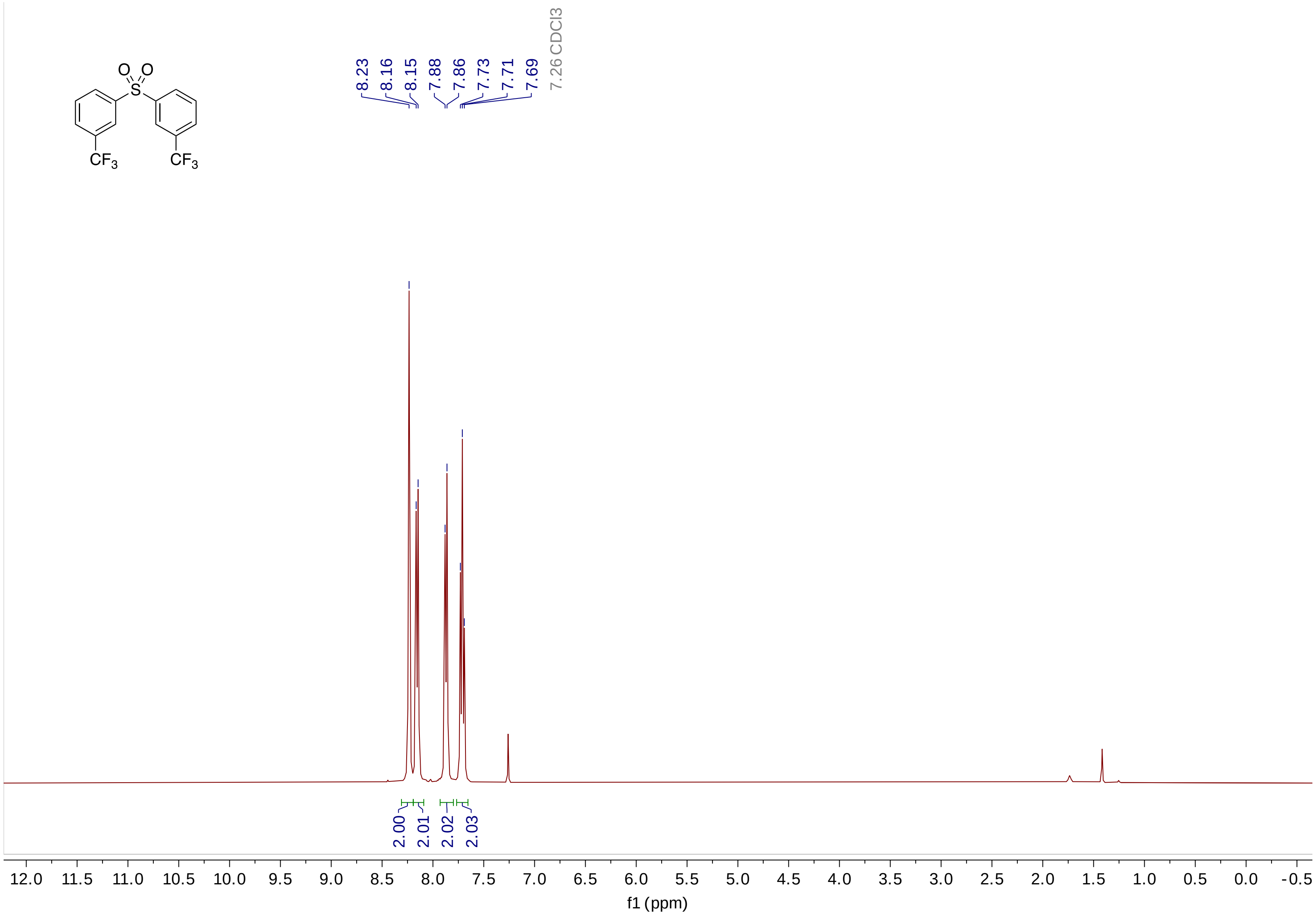

L4 - $^{13}C\{^{1}H\}$ NMR (101 MHz, $CDCl_3$):

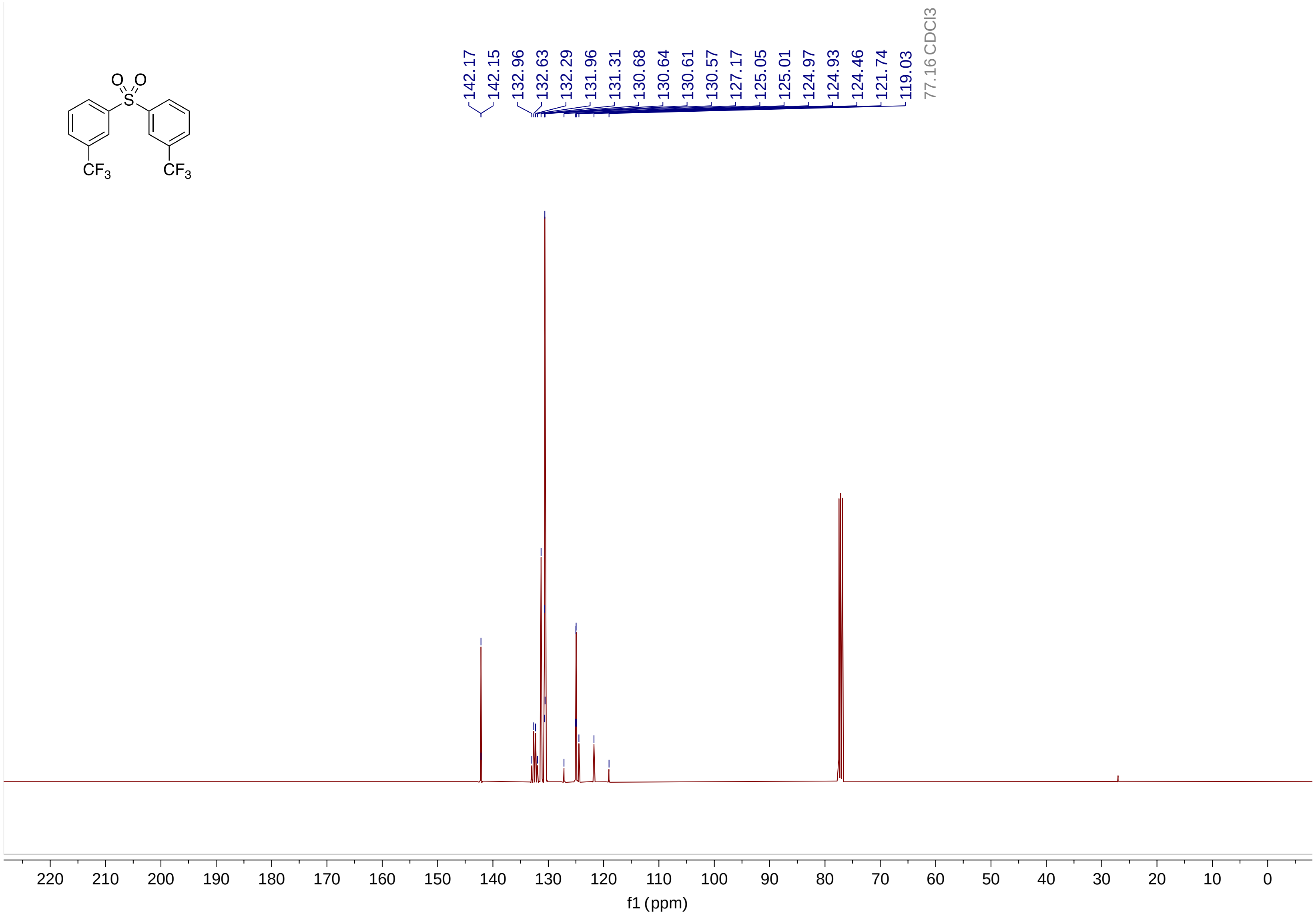

L4 - ^{19}F NMR (376 MHz, $CDCl_3$):

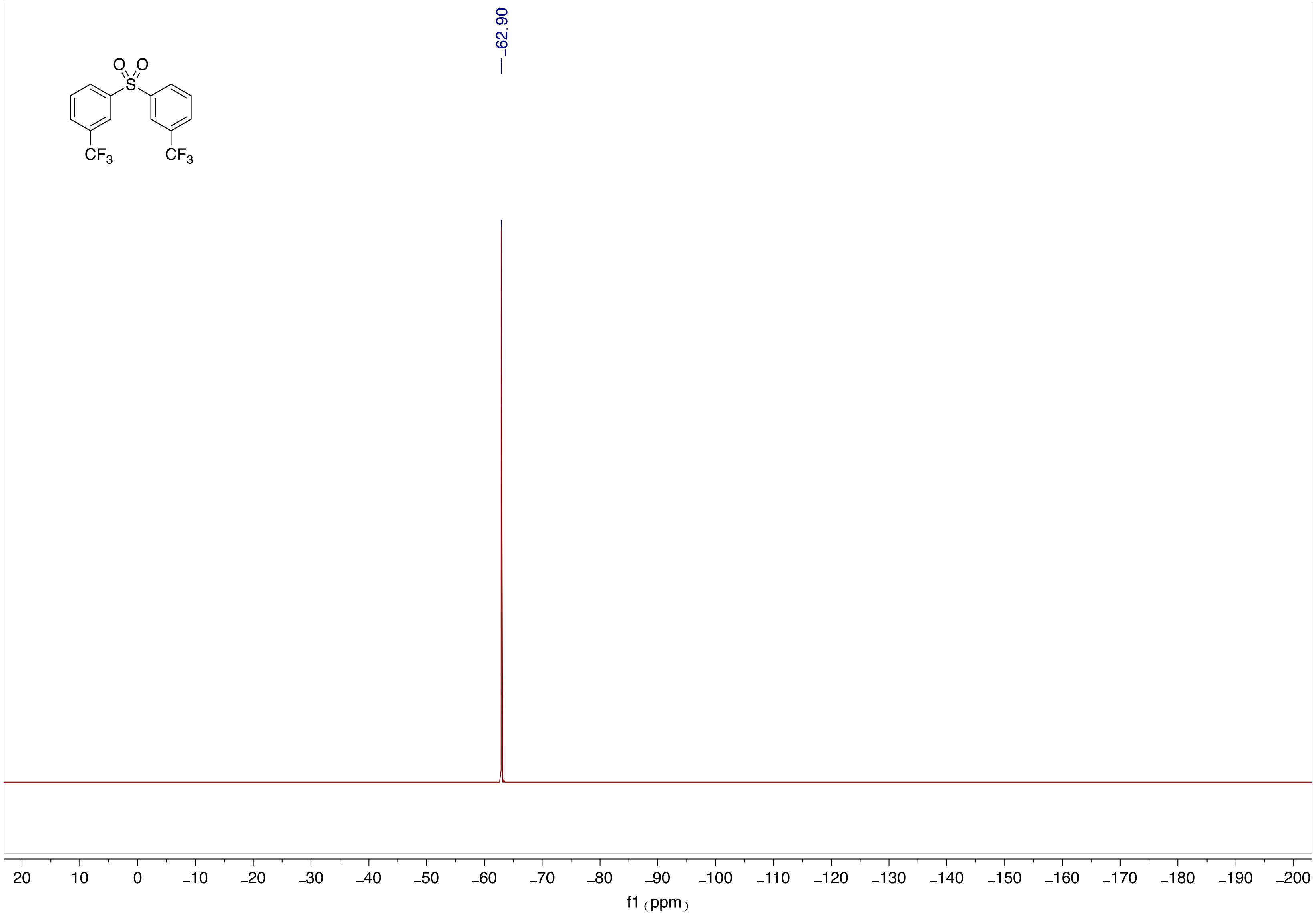

L5 - ^{1}H NMR (400 MHz, CDCl$_3$):

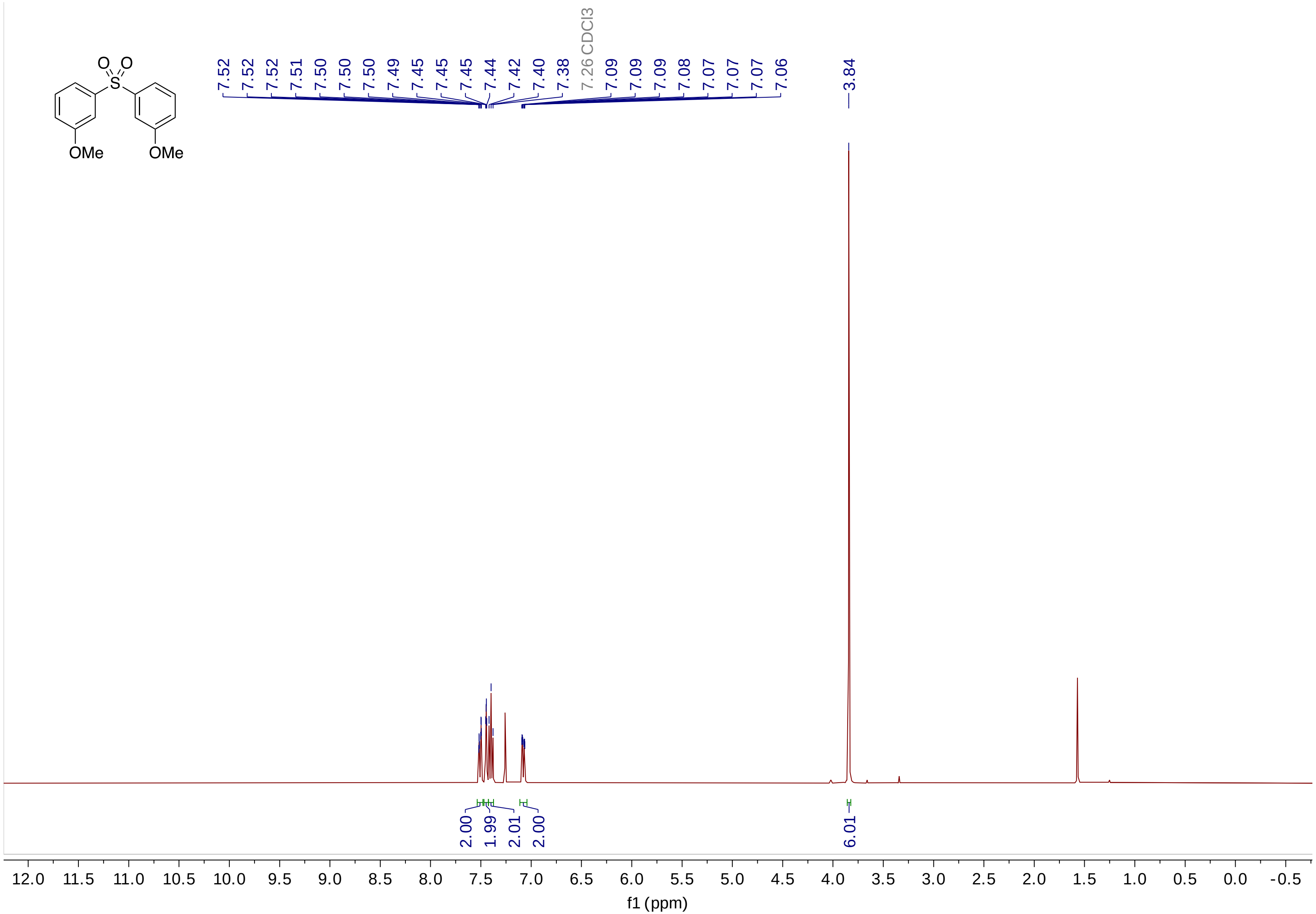

L5 - $^{13}C\{^{1}H\}$ NMR (101 MHz, $CDCl_3$):

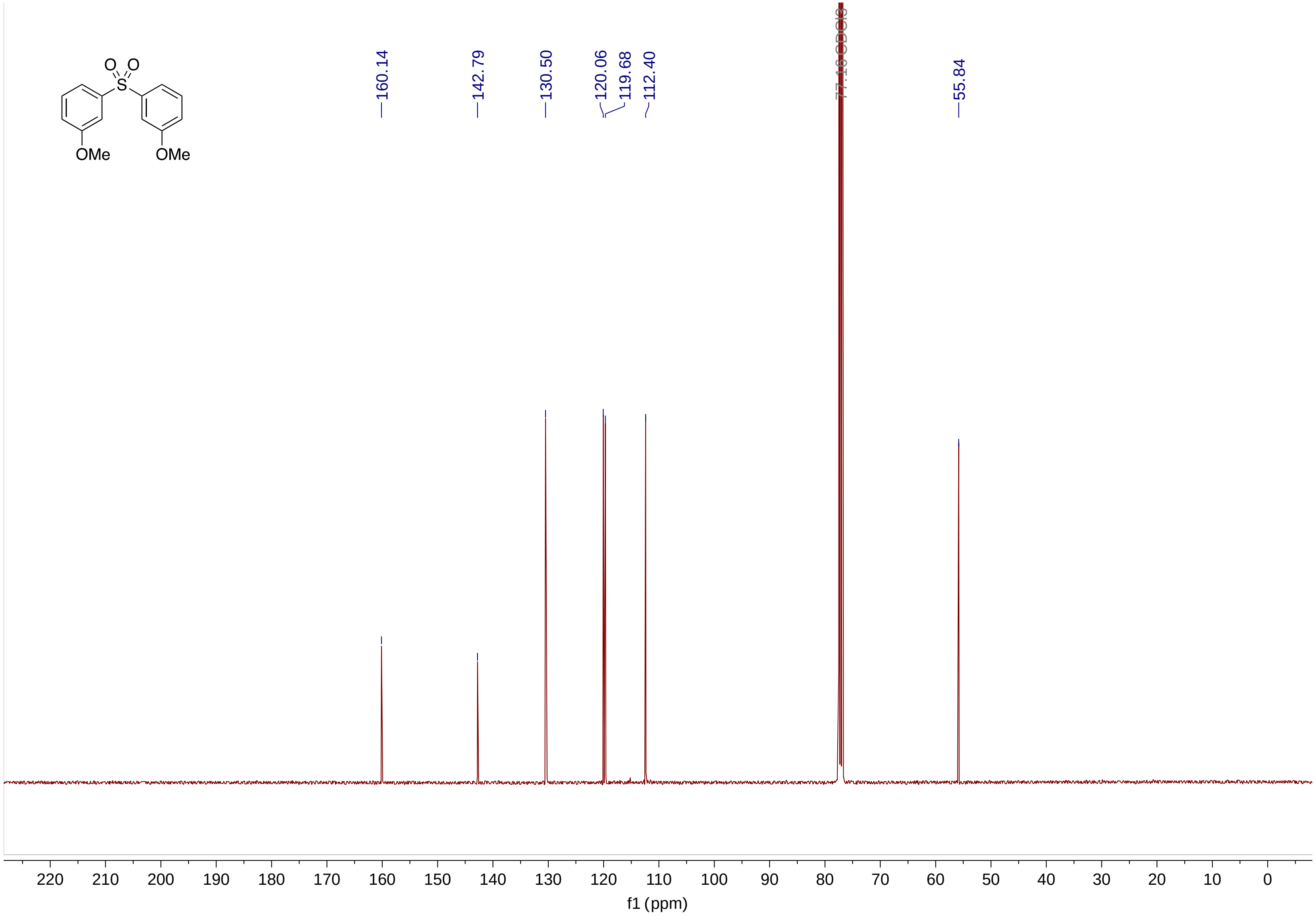

L6 - ^{1}H NMR (400 MHz, $CDCl_3$):

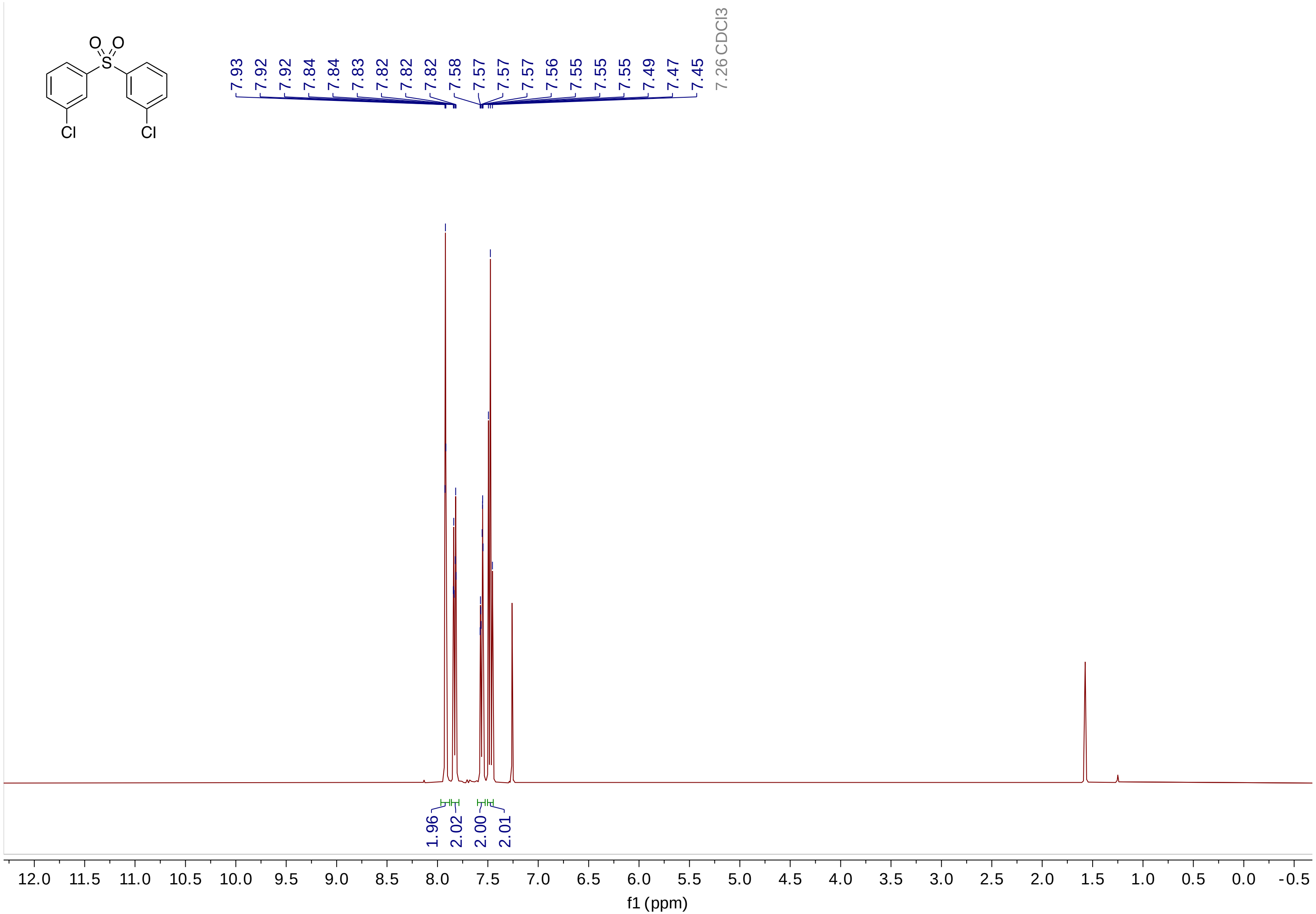

L6 - $^{13}C\{^1H\}$ NMR (101 MHz, $CDCl_3$):

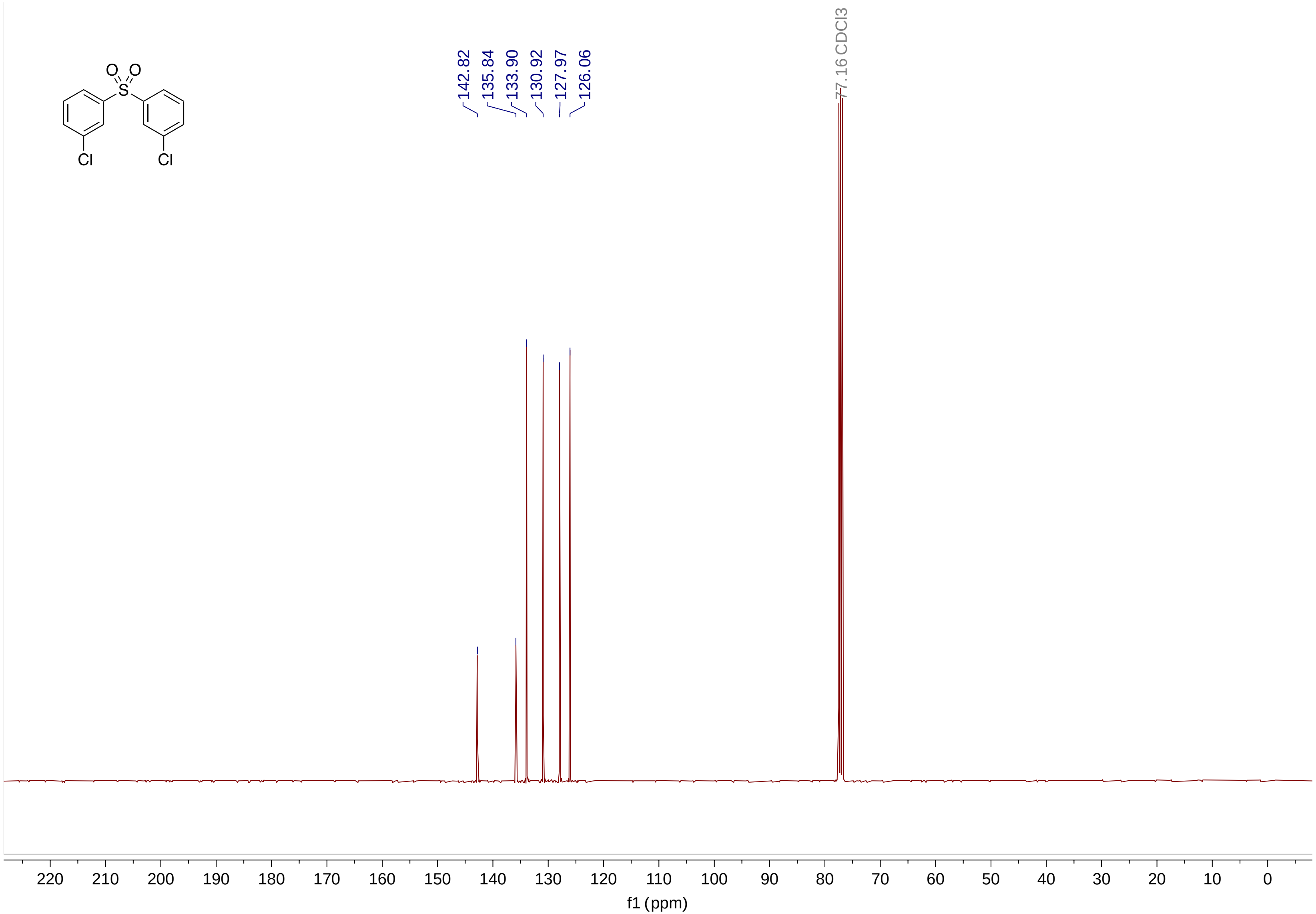

S2b - ^{1}H NMR (400 MHz, $CDCl_3$):

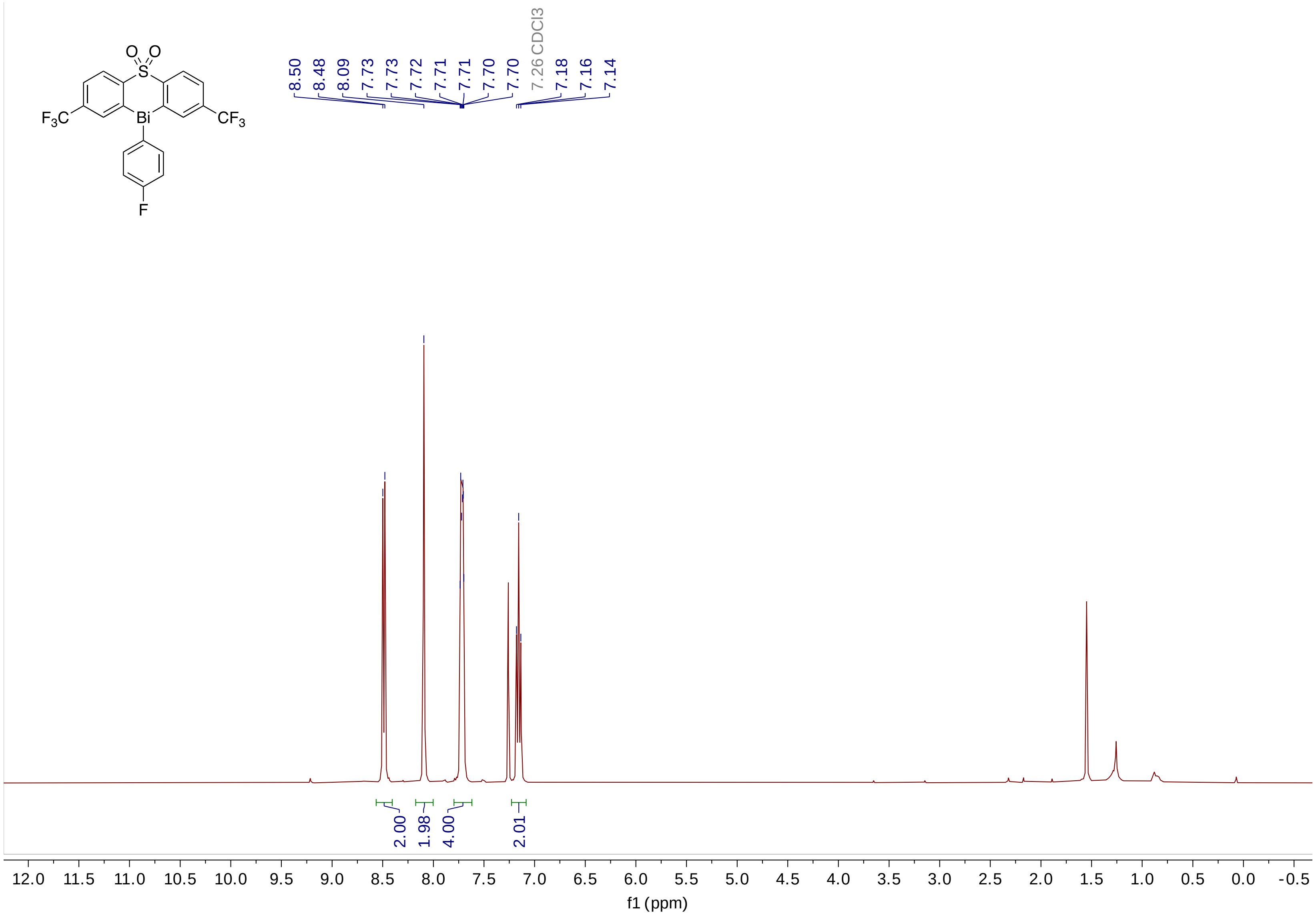

S2b - $^{13}C\{^{1}H\}$ NMR (101 MHz, $CDCl_3$):

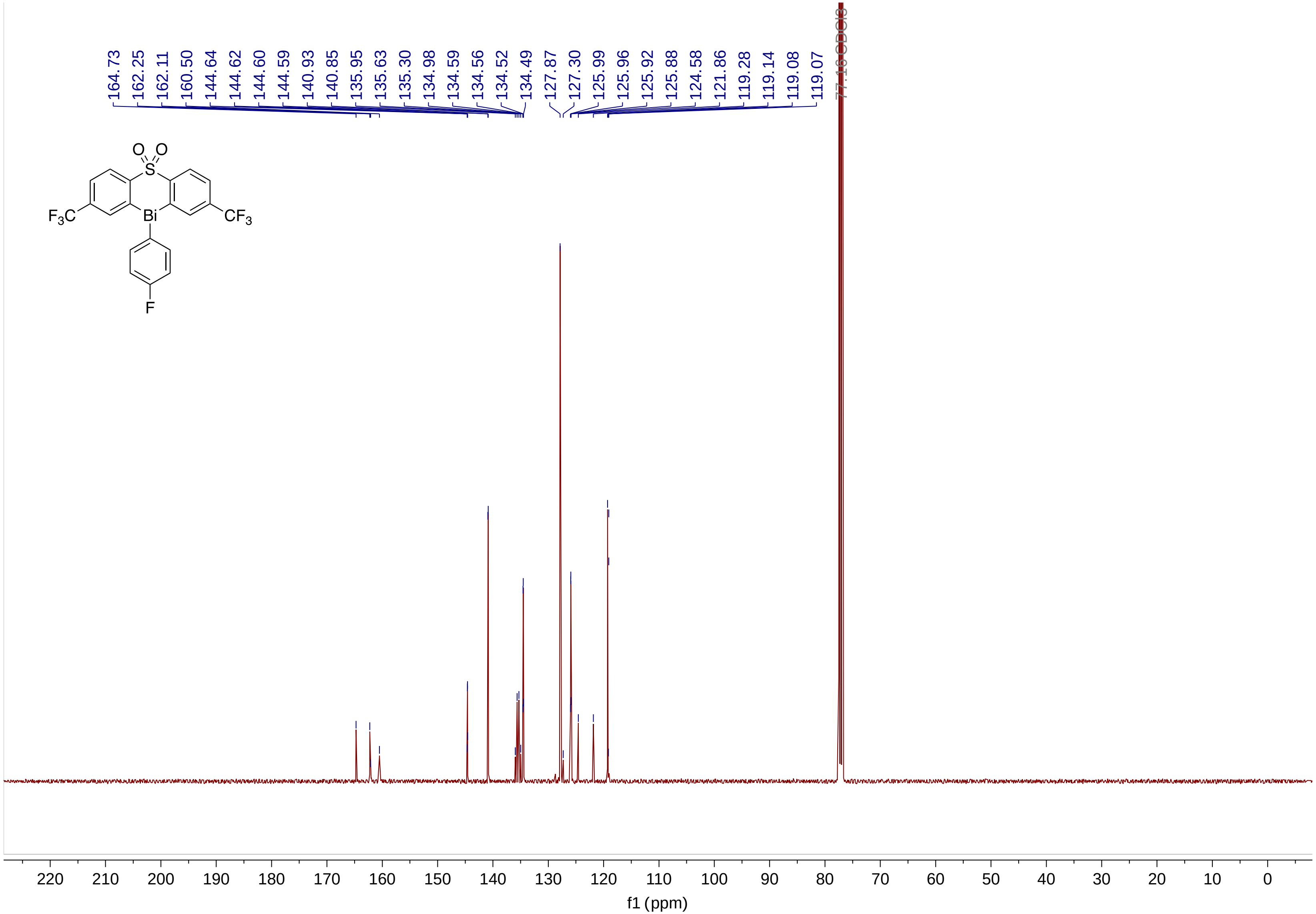

S2b - ^{19}F NMR (376 MHz, $CDCl_3$):

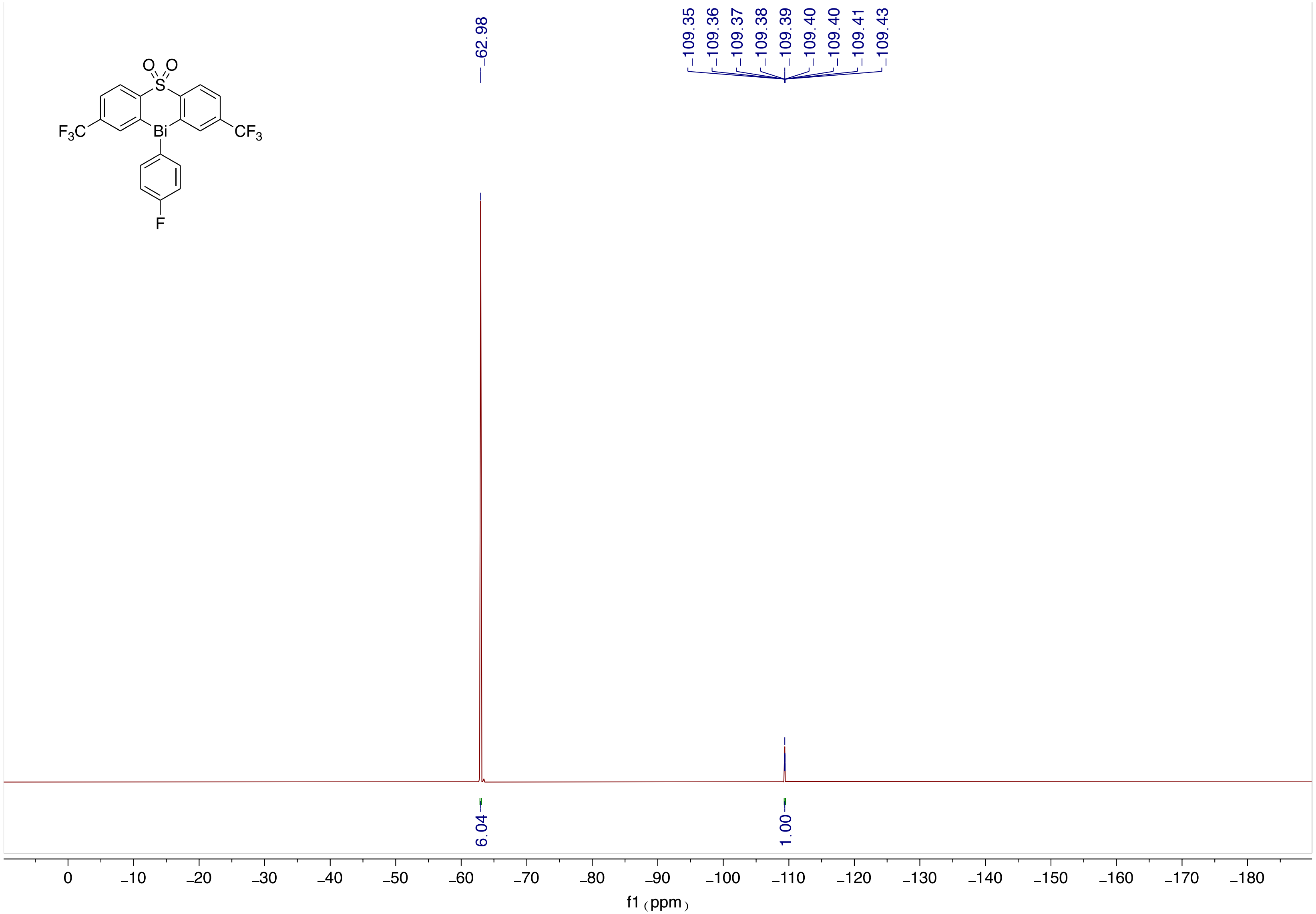

S3b - ^{1}H NMR (400 MHz, $CDCl_3$):

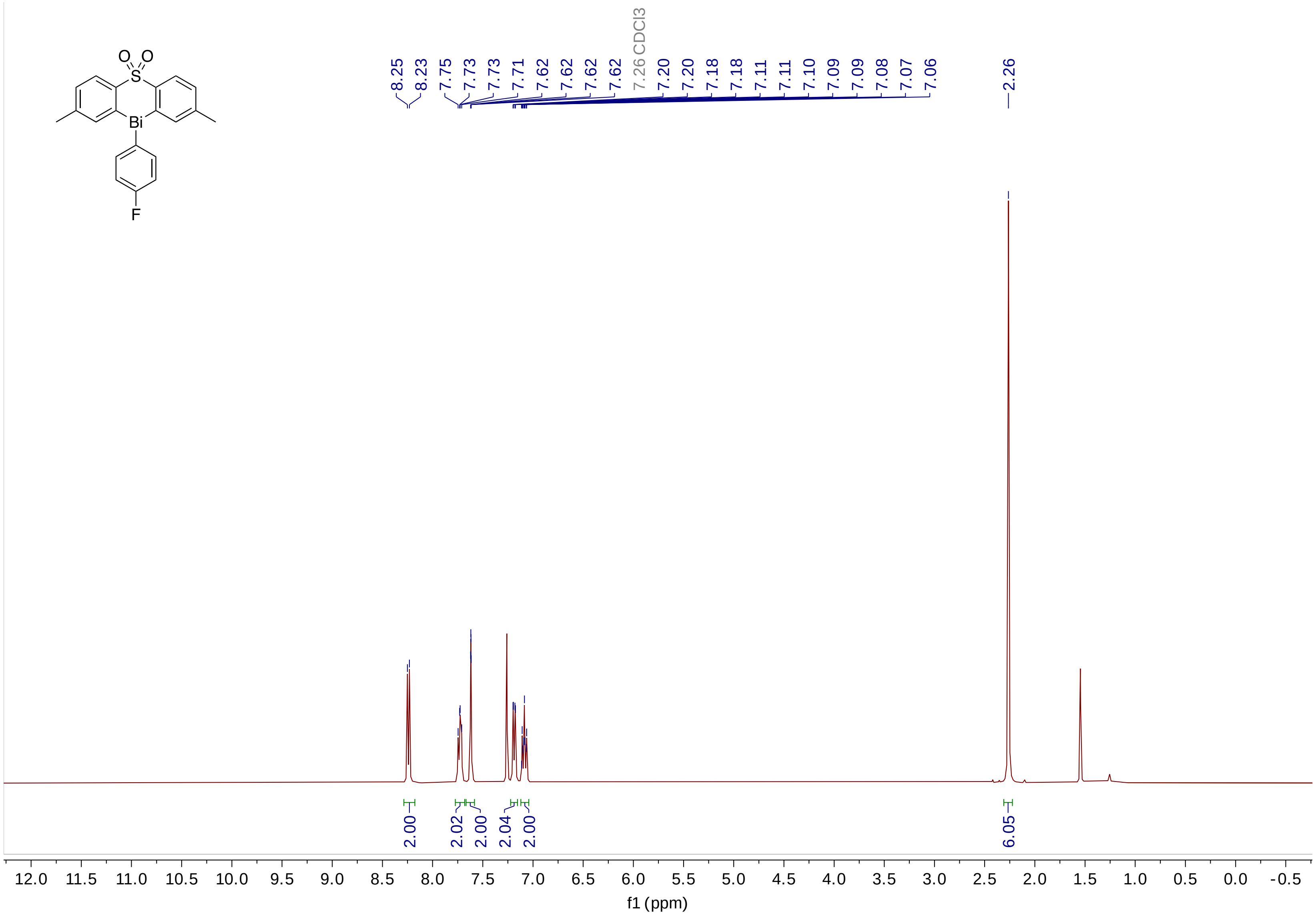

S3b - $^{13}C\{^{1}H\}$ NMR (101 MHz, $CDCl_3$):

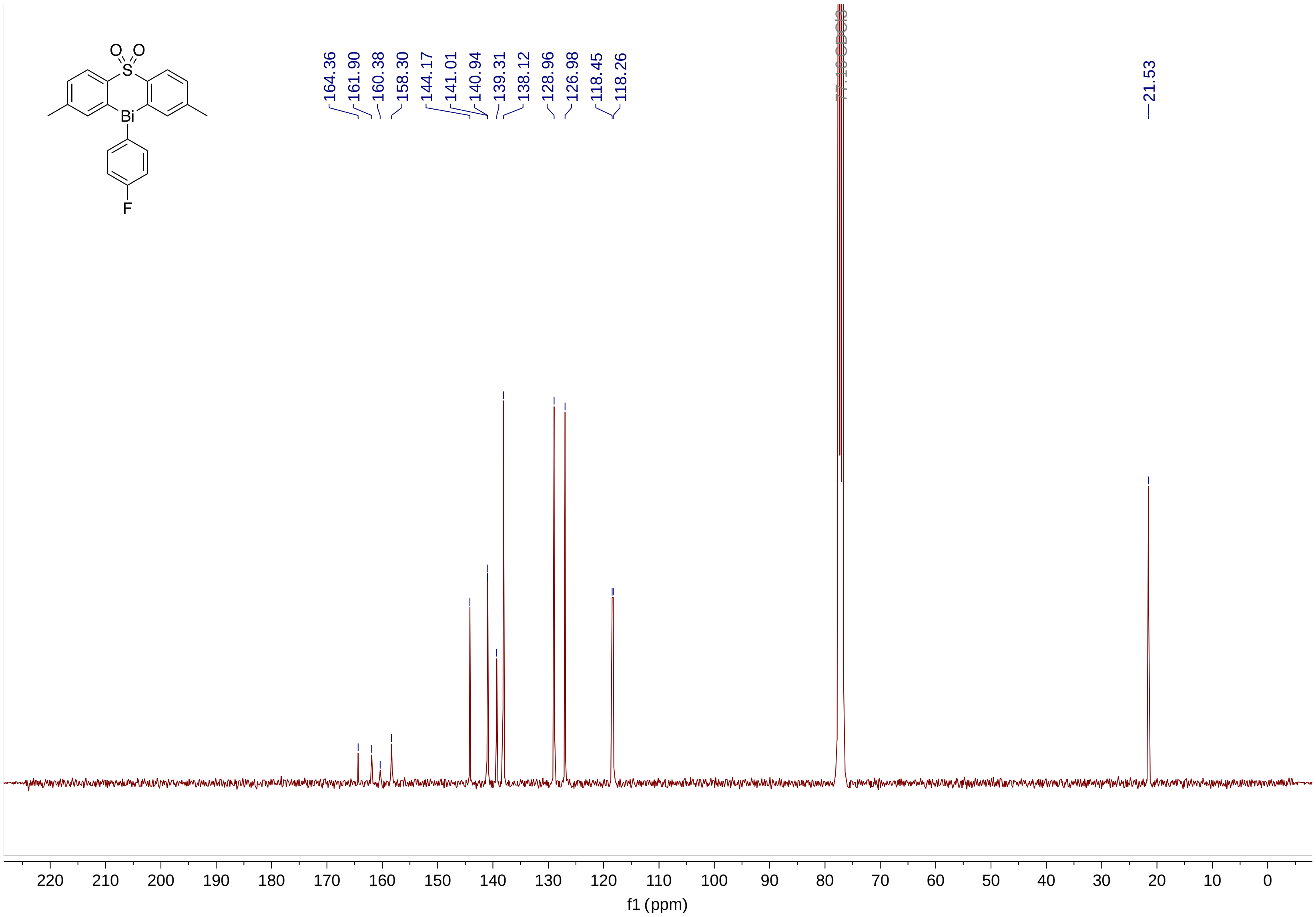

S3b - ^{19}F NMR (377 MHz, $CDCl_3$):

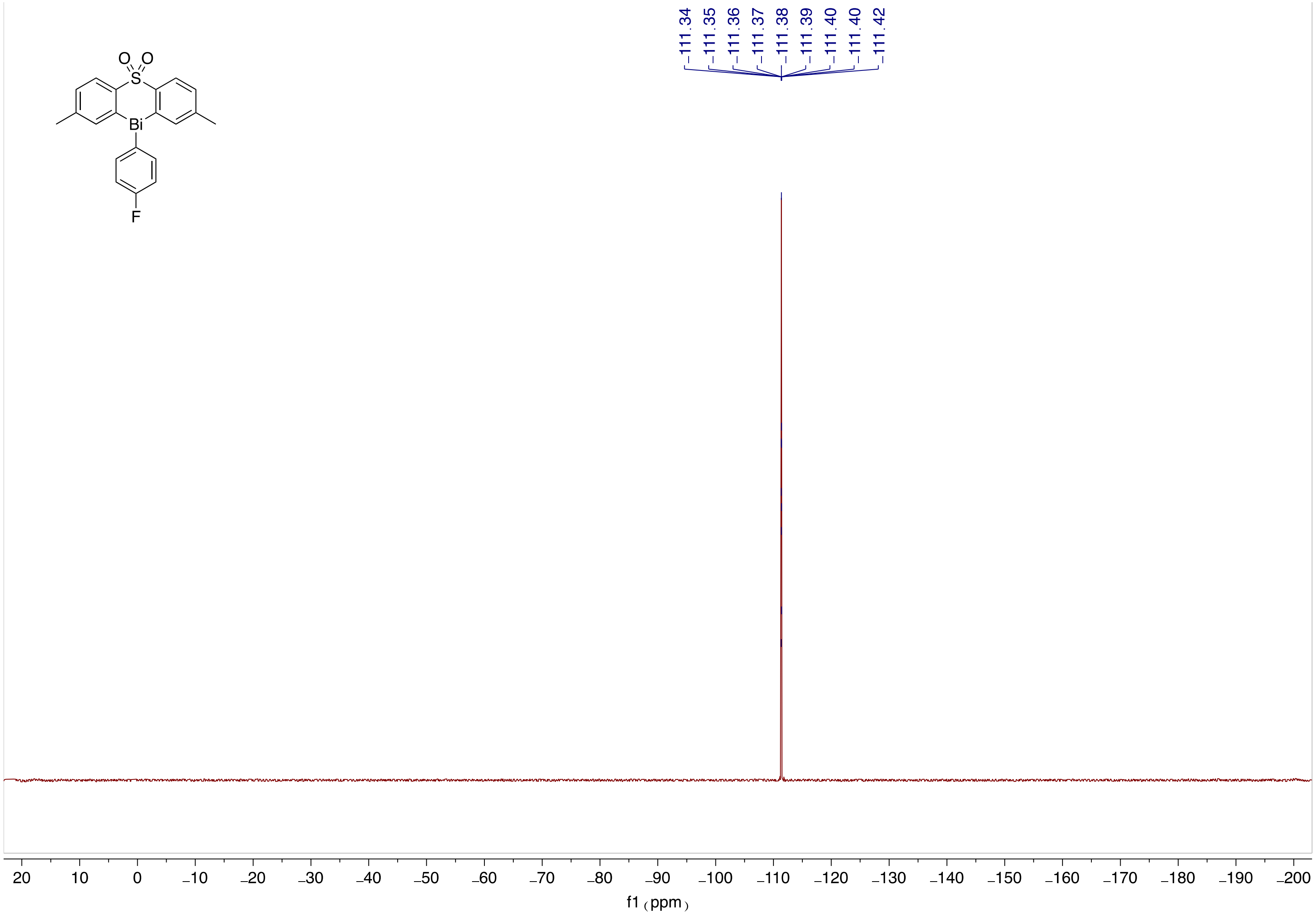

S4b - ^{1}H NMR (400 MHz, $CDCl_3$):

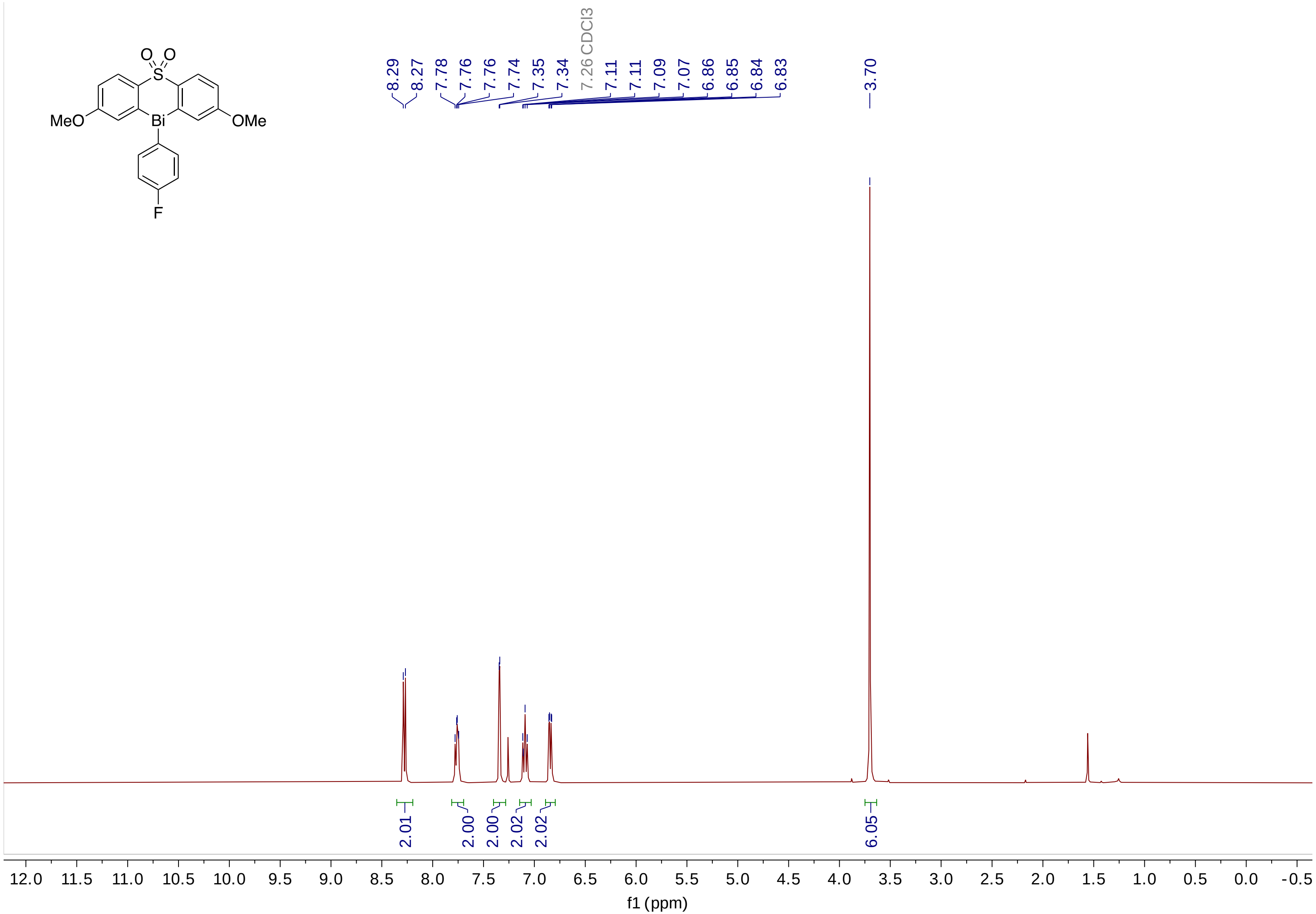

S4b - $^{13}C\{^{1}H\}$ NMR (101 MHz, $CDCl_3$):

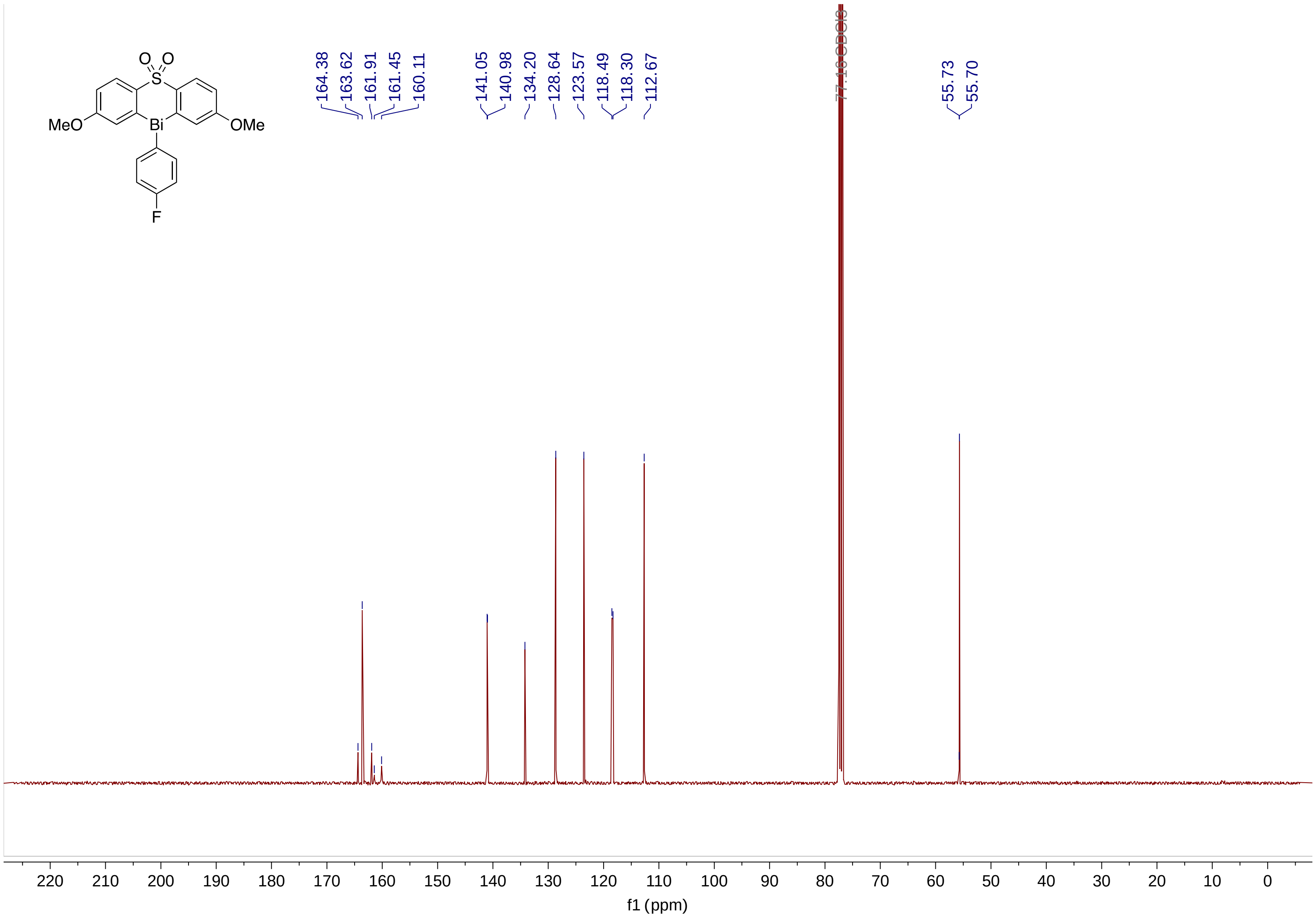

S4b - ^{19}F NMR (377 MHz, $CDCl_3$):

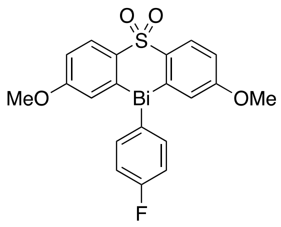

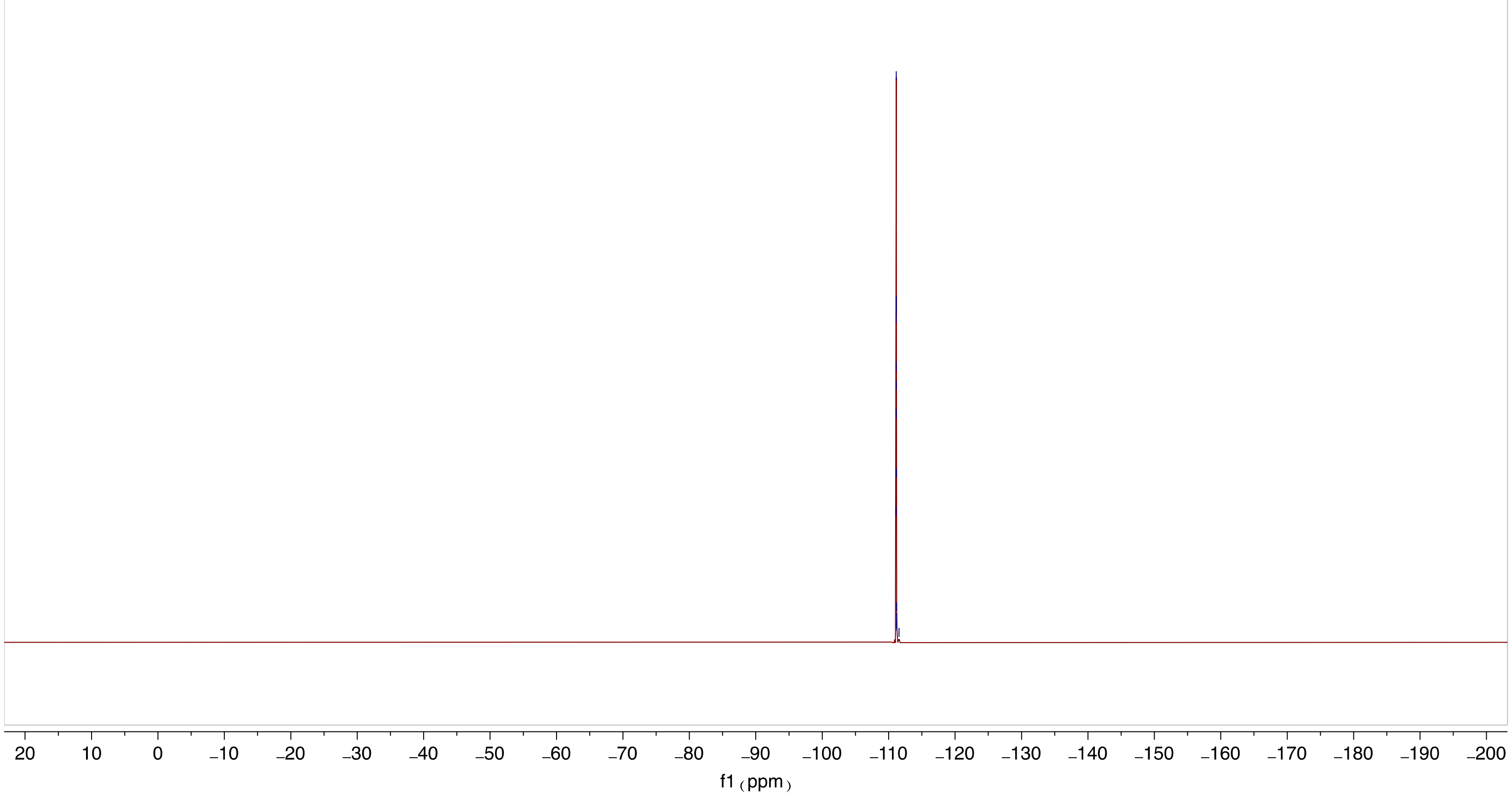

S5b - ^{1}H NMR (400 MHz, $CDCl_3$):

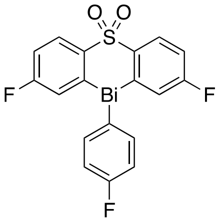

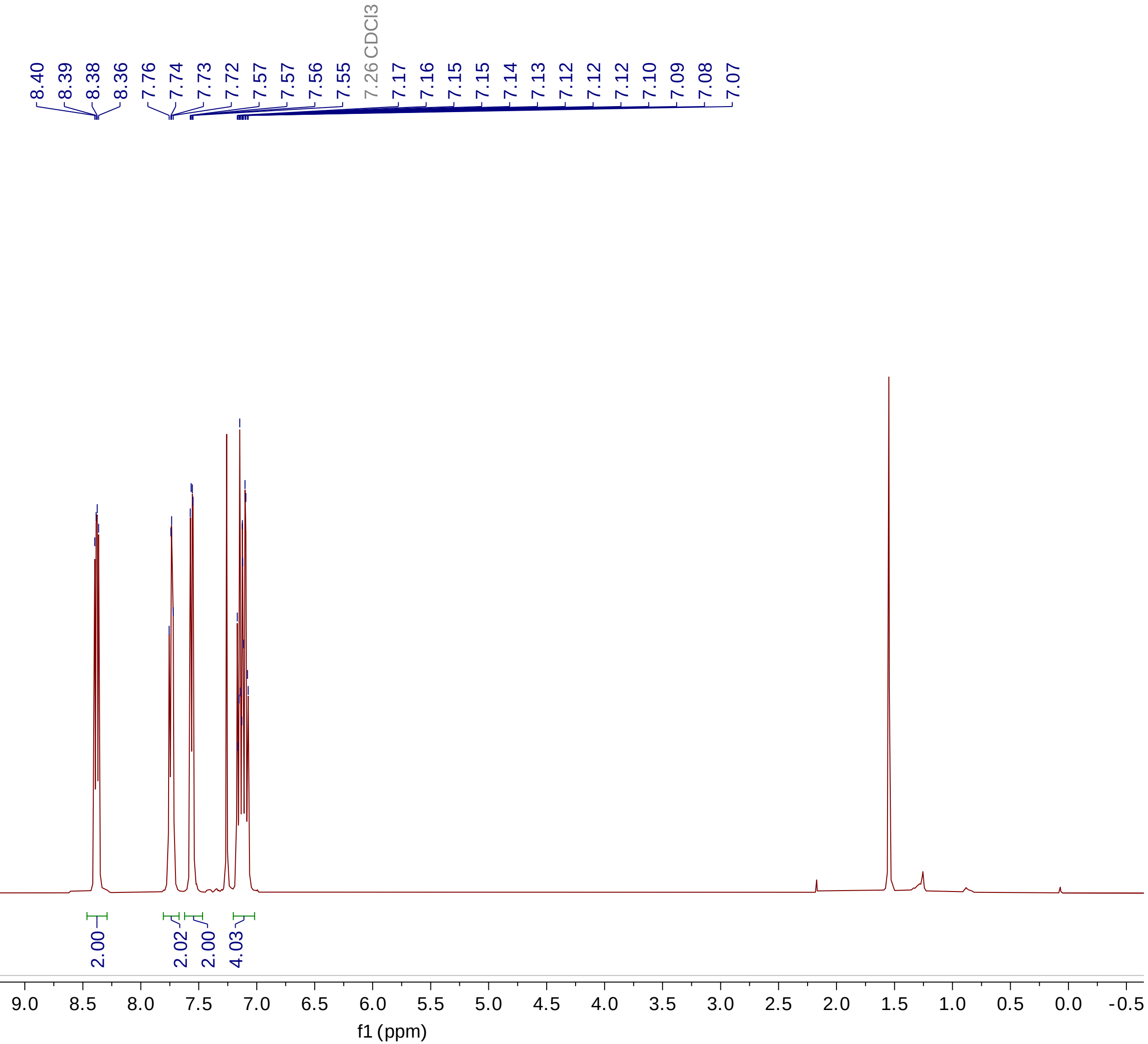

S5b - $^{13}C\{^{1}H\}$ NMR (101 MHz, $CDCl_3$):

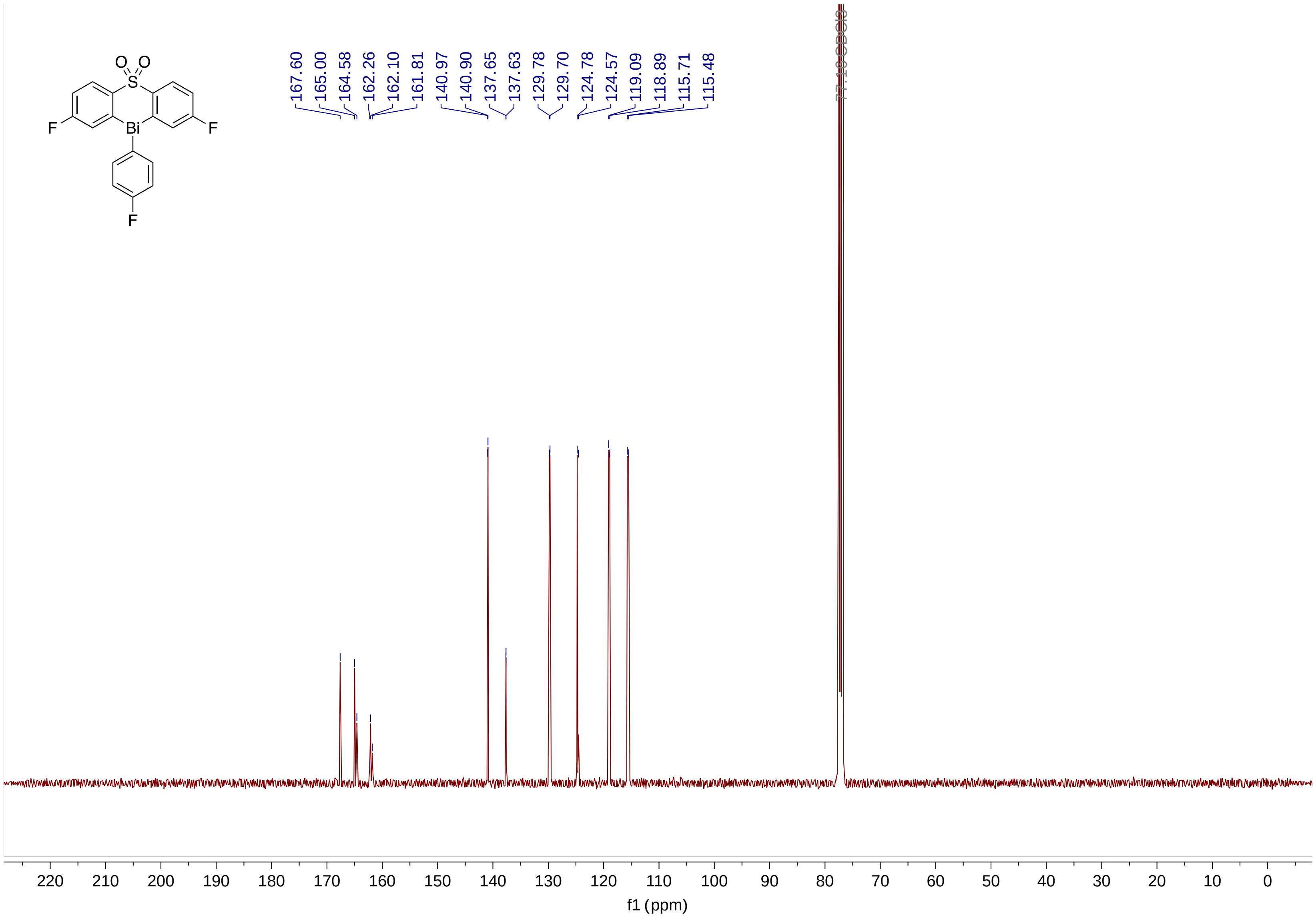

S5b - ^{19}F NMR (377 MHz, $CDCl_3$):

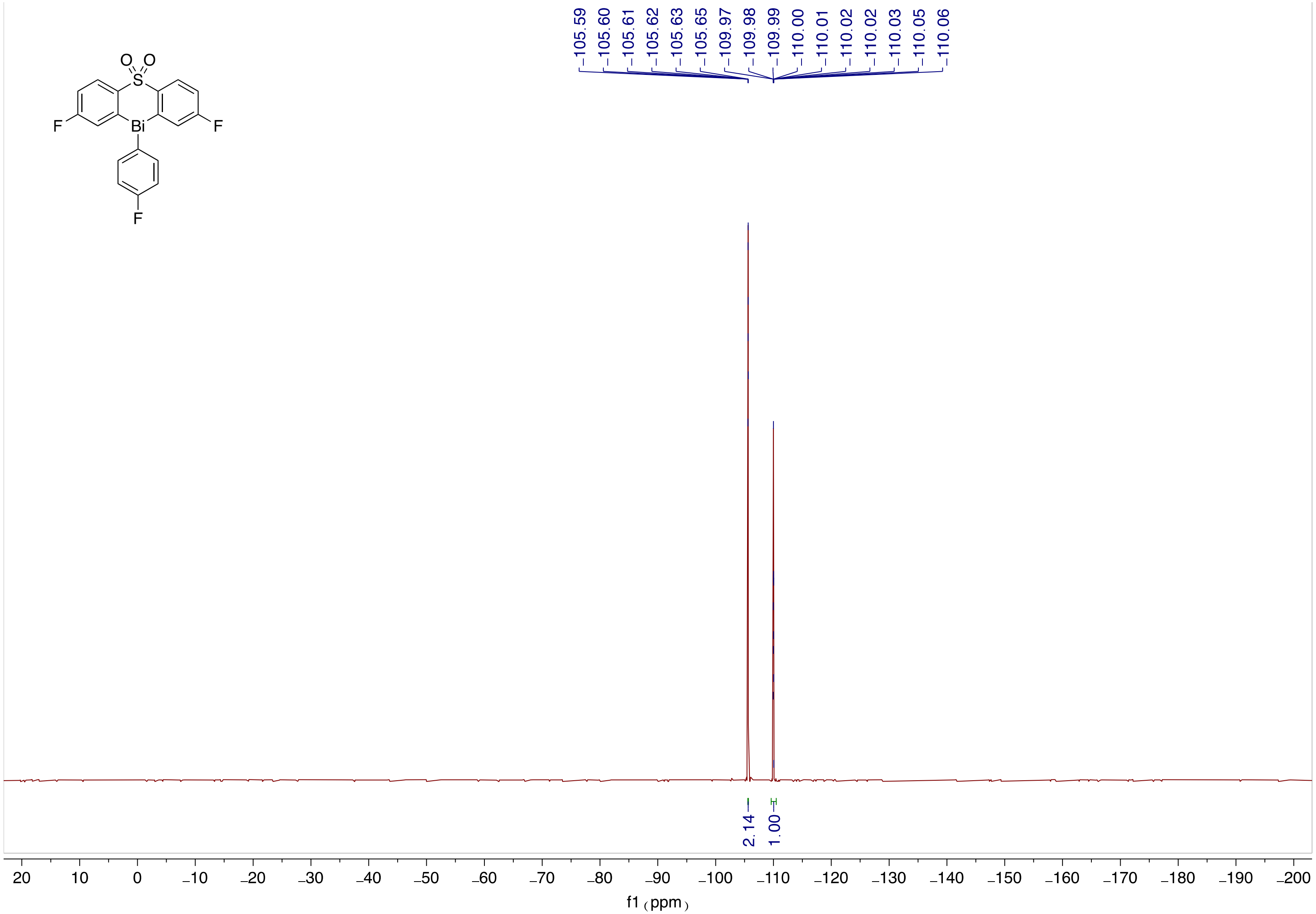

S6b - ^{1}H NMR (400 MHz, $CDCl_3$):

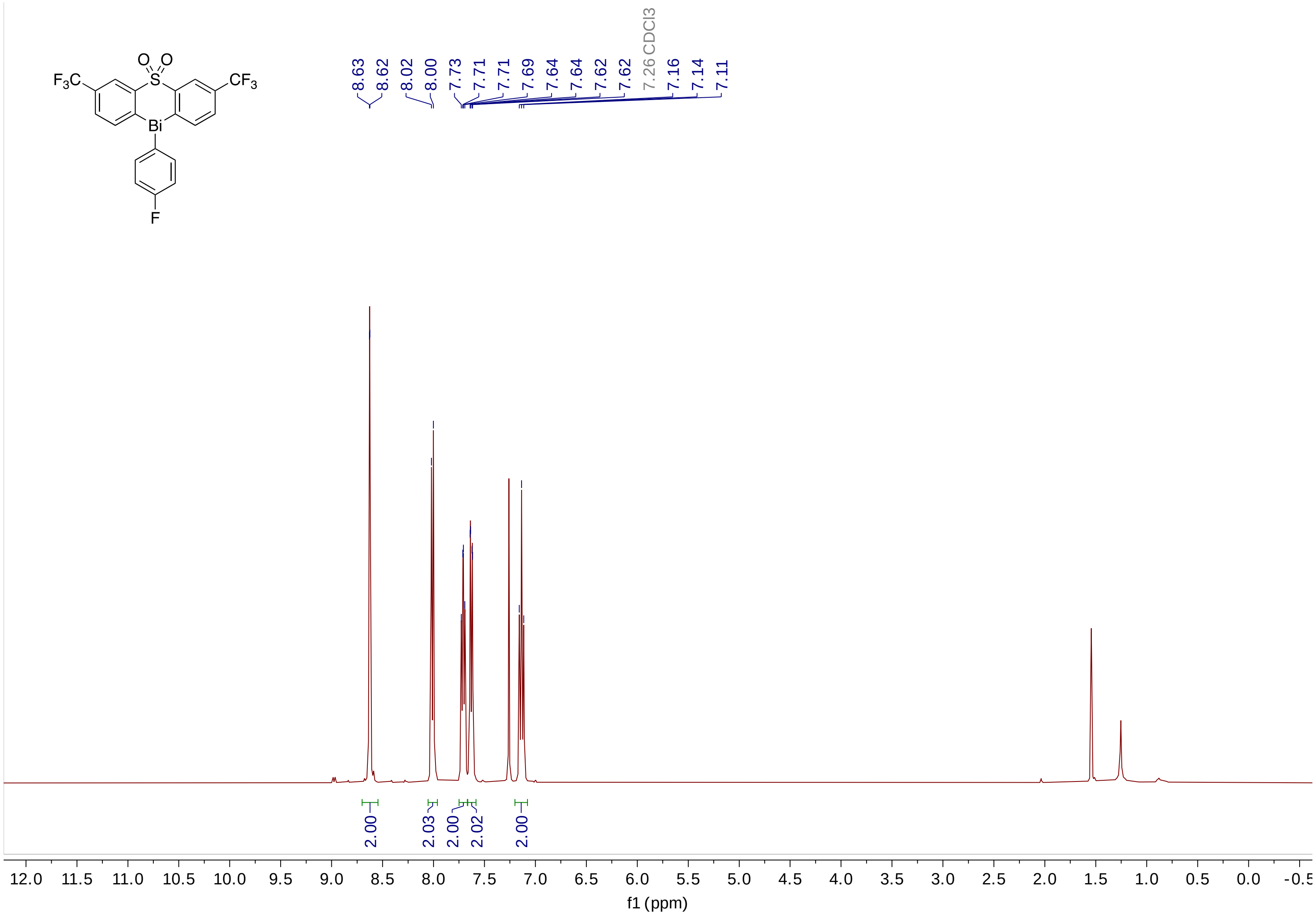

S6b - $^{13}C\{^{1}H\}$ NMR (101 MHz, $CDCl_3$):

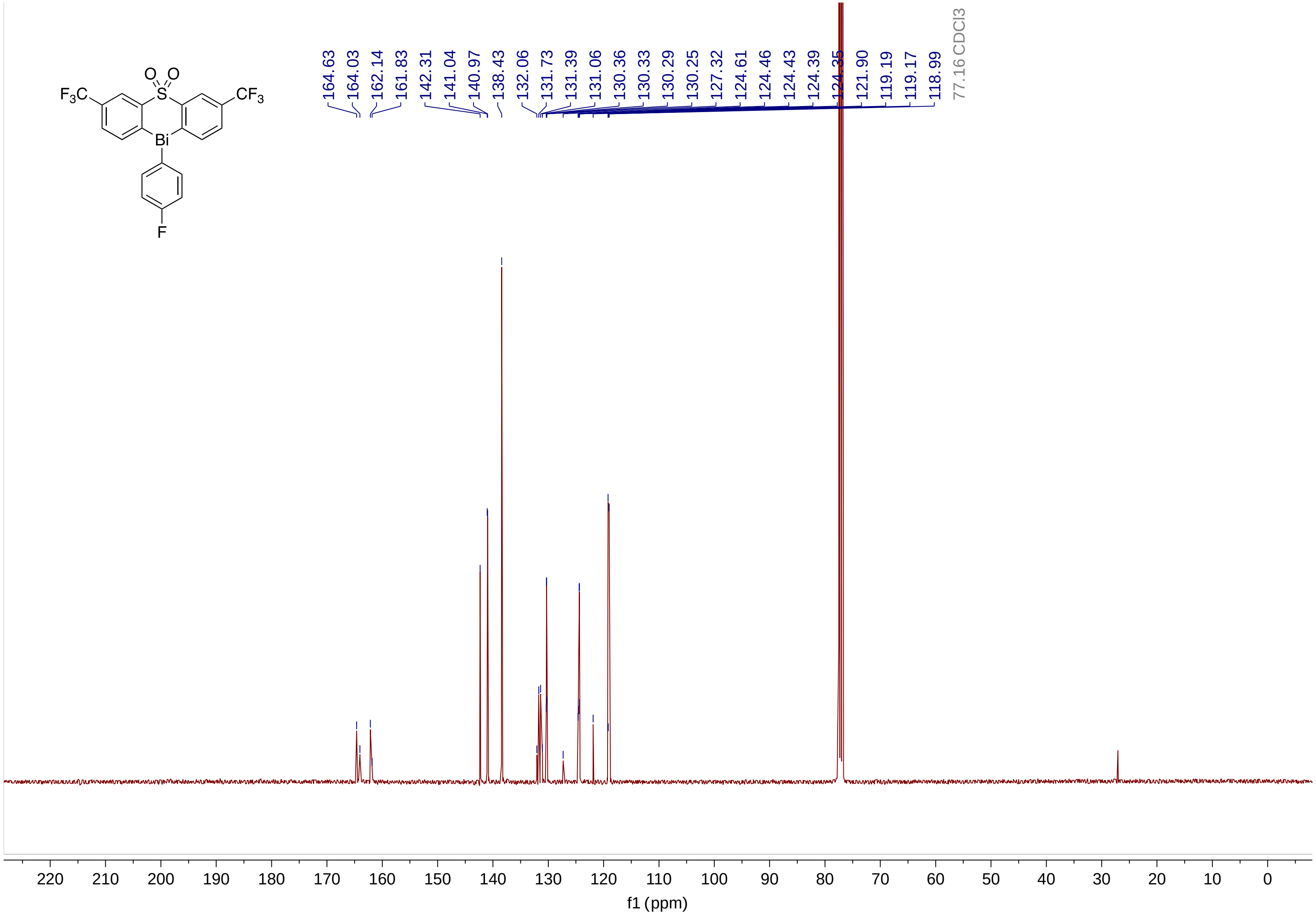

S6b - ^{19}F NMR (377 MHz, CDCl$_3$):

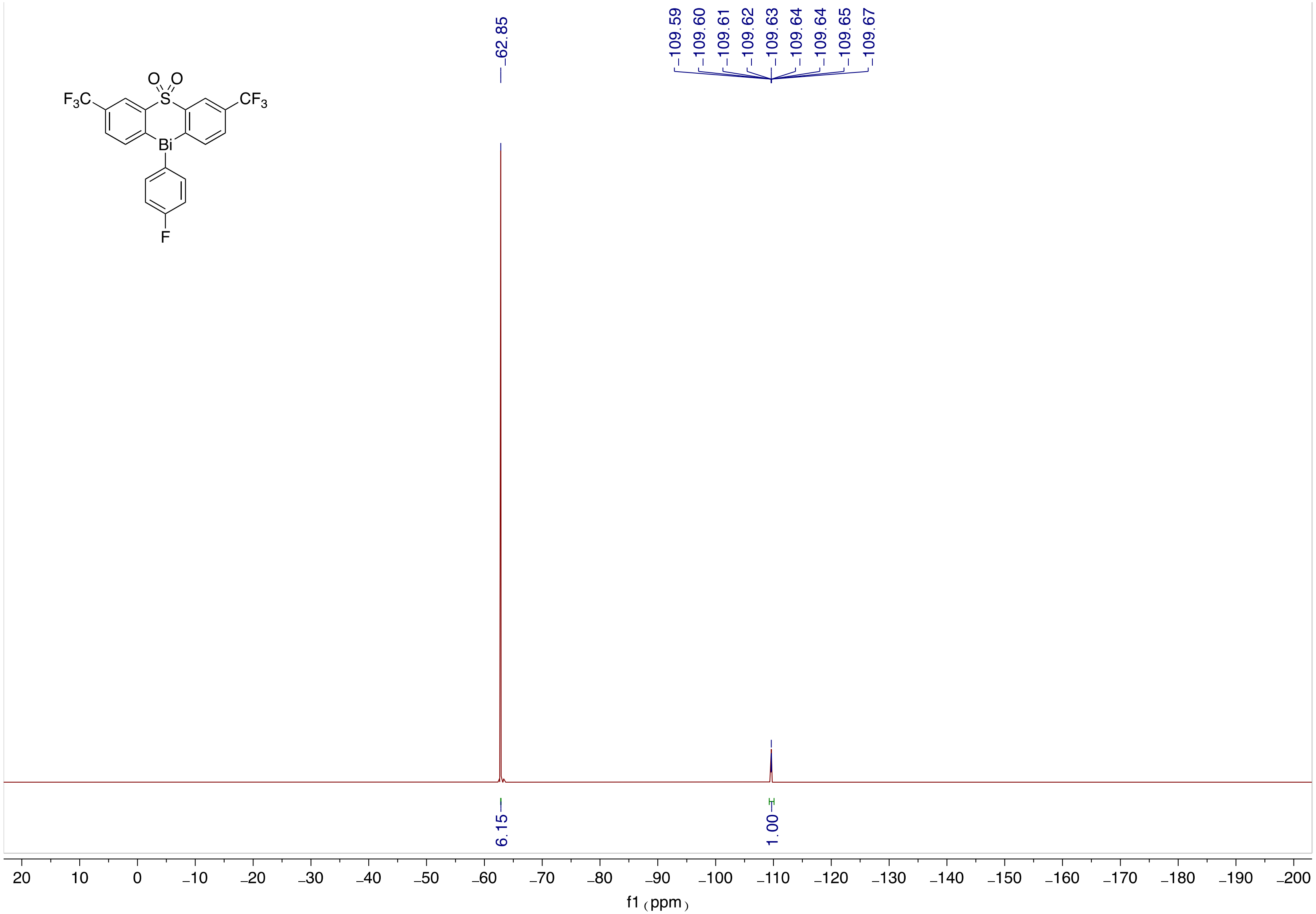

S7b - ^{1}H NMR (400 MHz, $CDCl_3$):

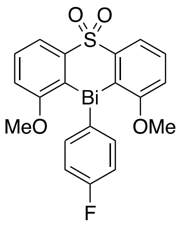

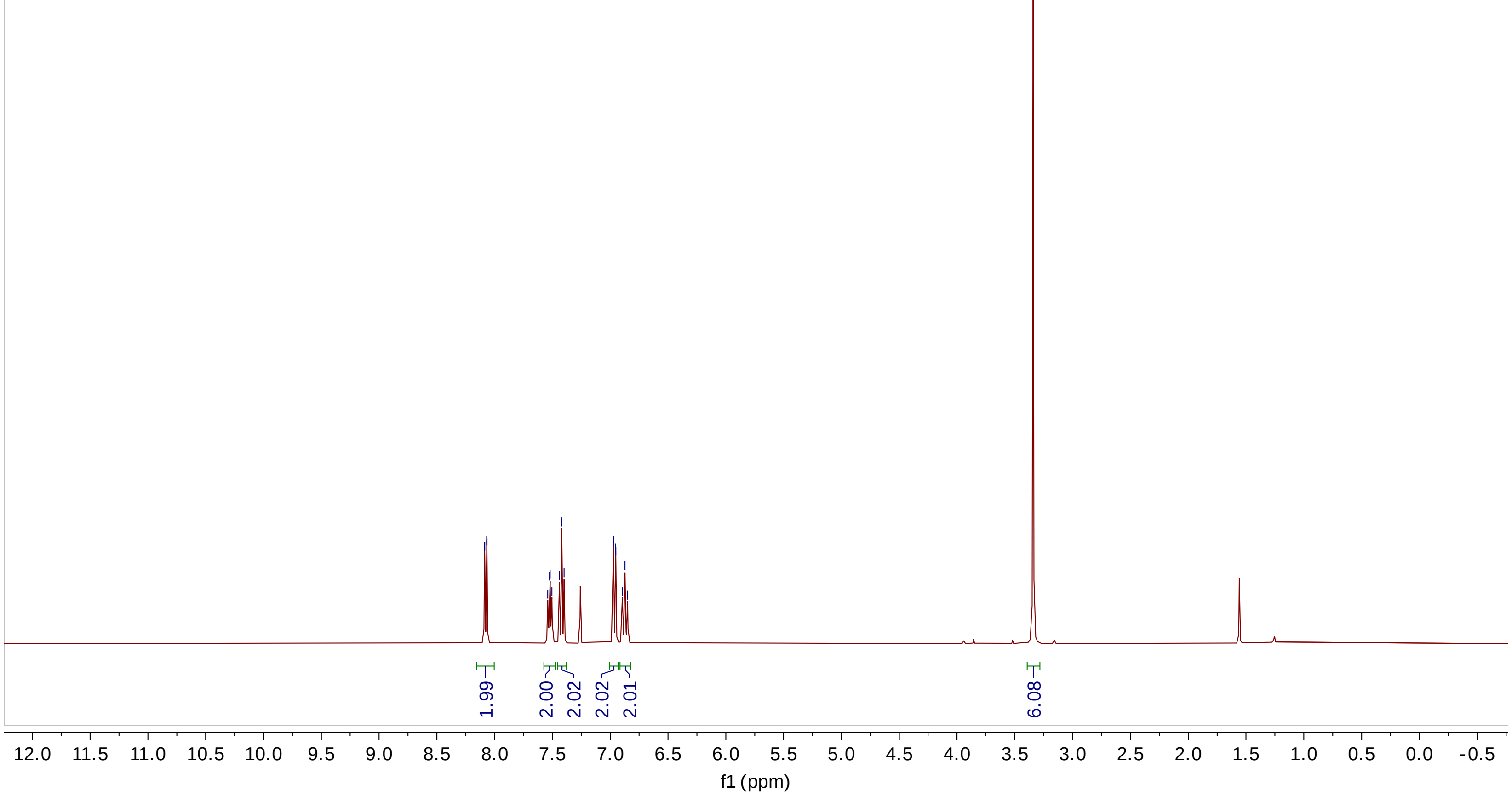

S7b - $^{13}C\{^1H\}$ NMR (101 MHz, CDCl$_3$):

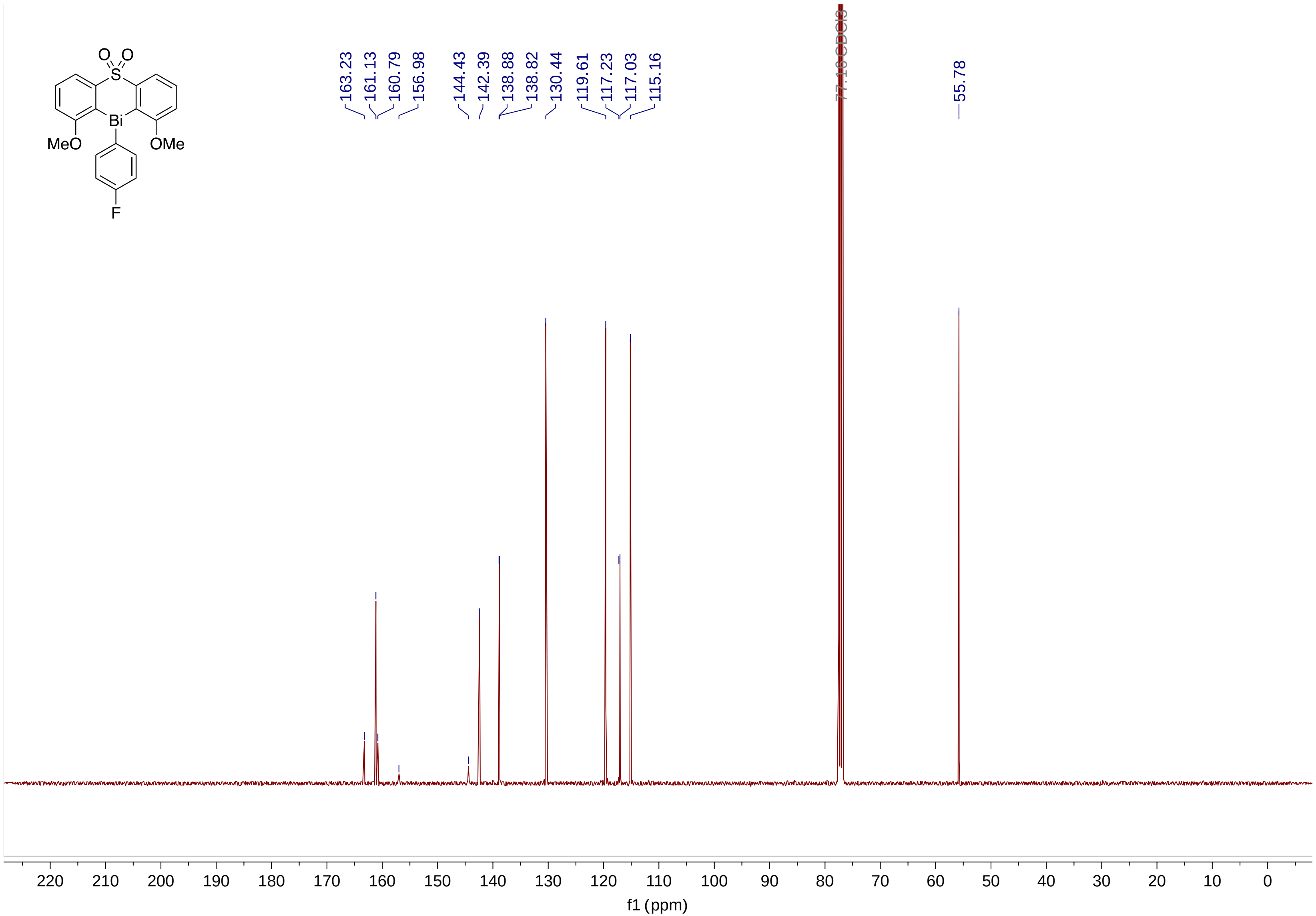

S7b - ¹⁹F NMR (377 MHz, $CDCl_3$):

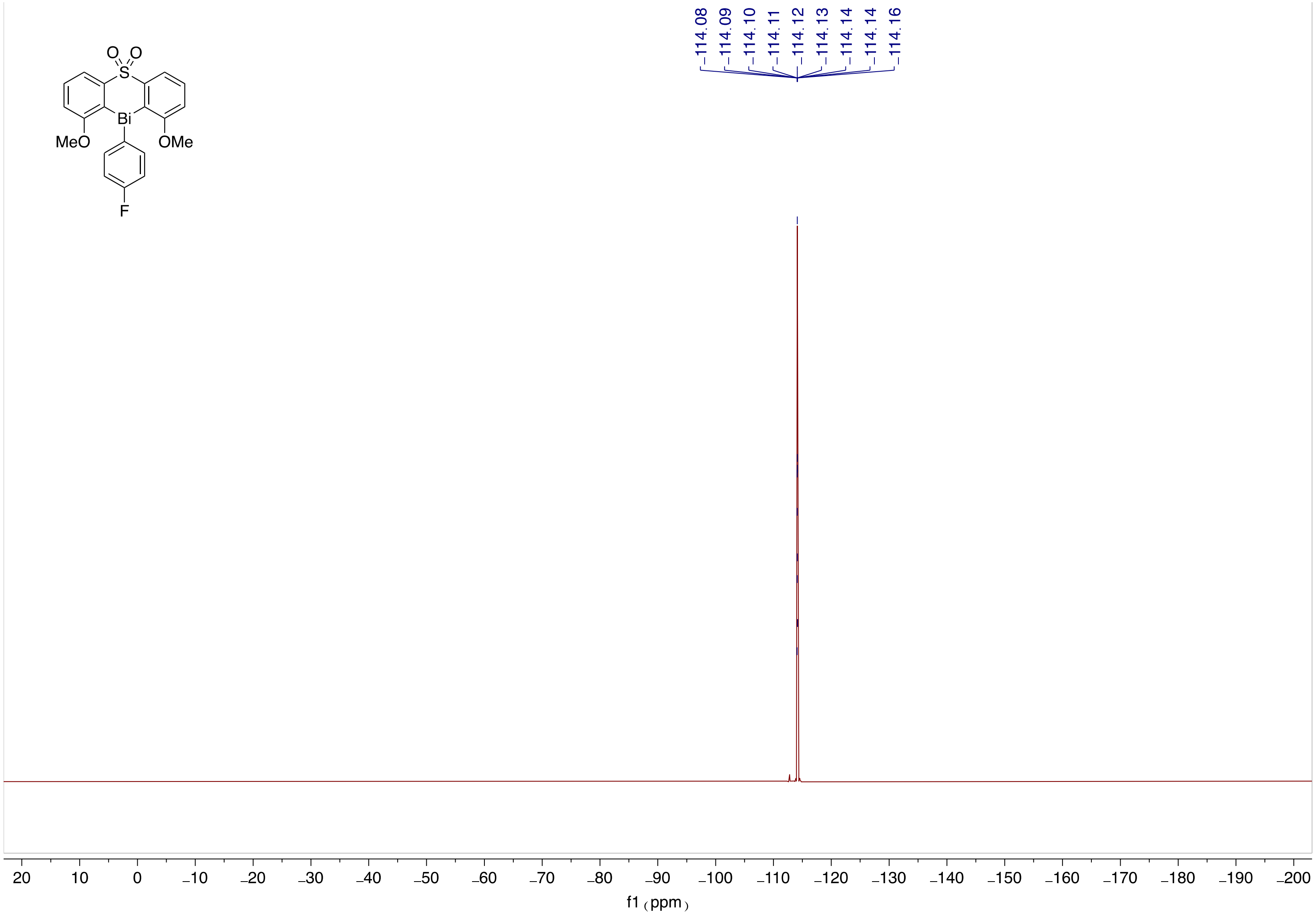

S8b - ^{1}H NMR (400 MHz, $CDCl_3$):

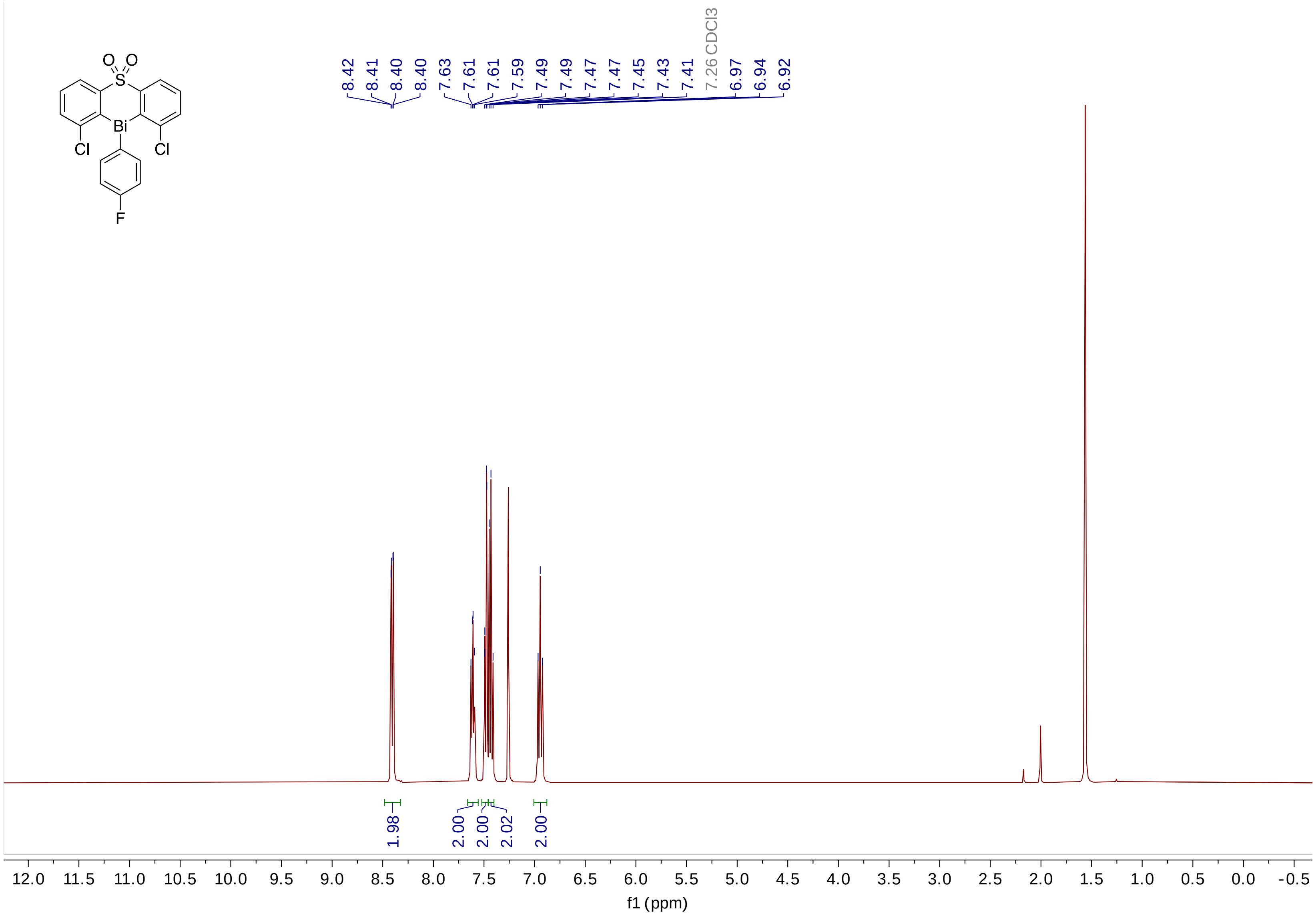

S8b - $^{13}C\{^1H\}$ NMR (101 MHz, $CDCl_3$):

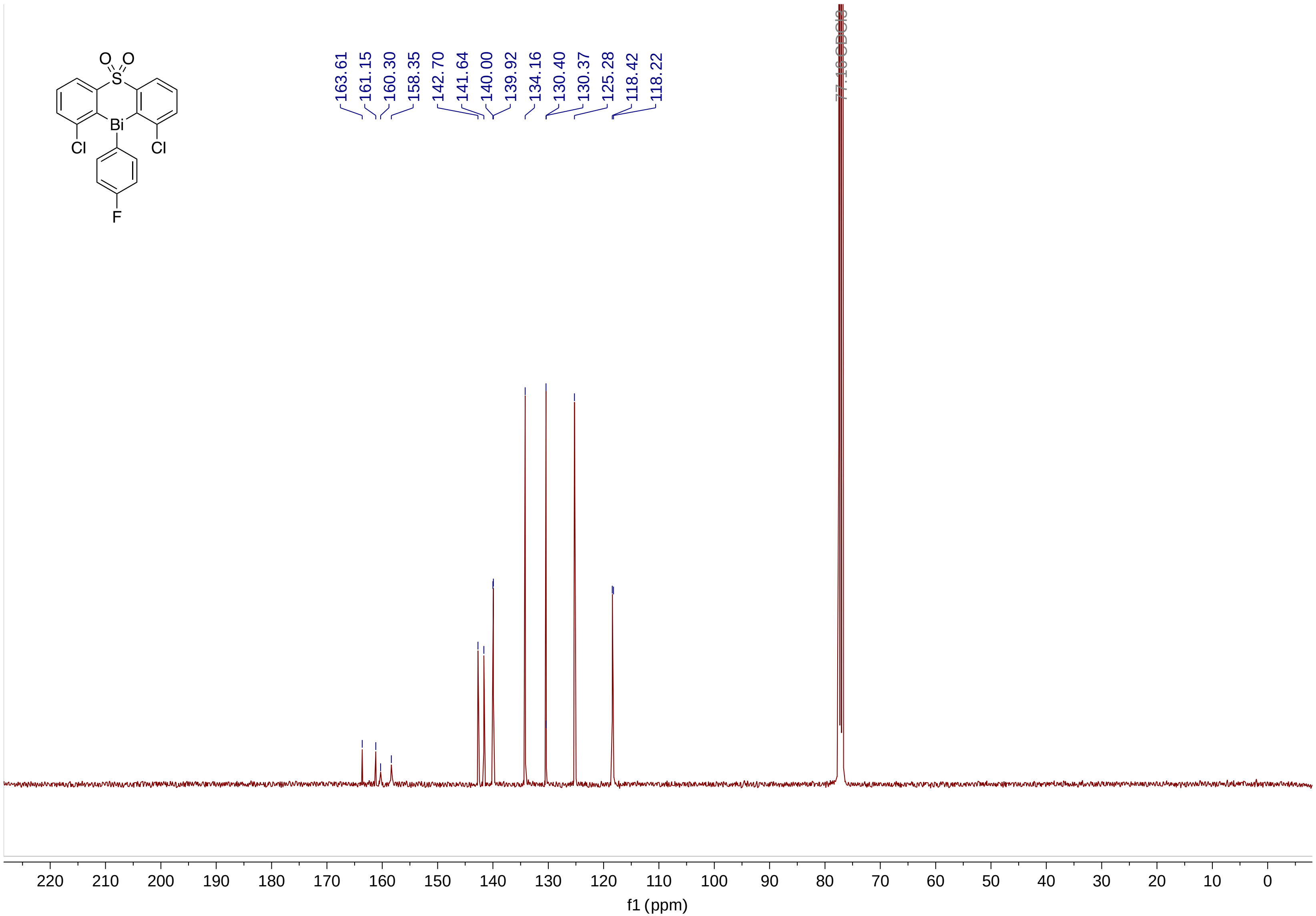

S8b - ^{19}F NMR (377 MHz, $CDCl_3$):

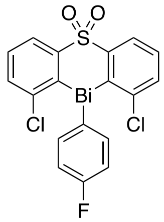

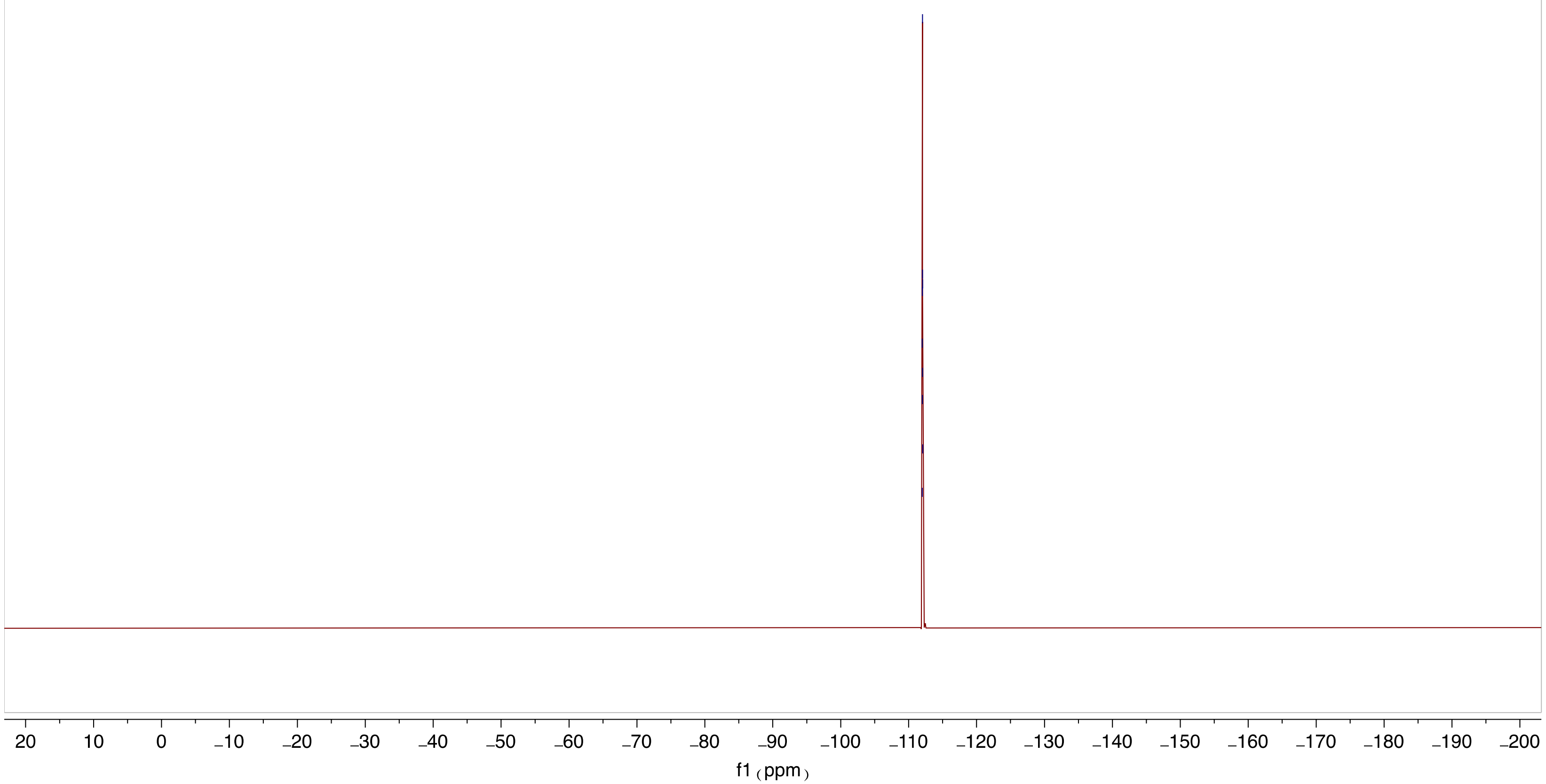

S2a - ^{1}H NMR (400 MHz, $CDCl_3$):

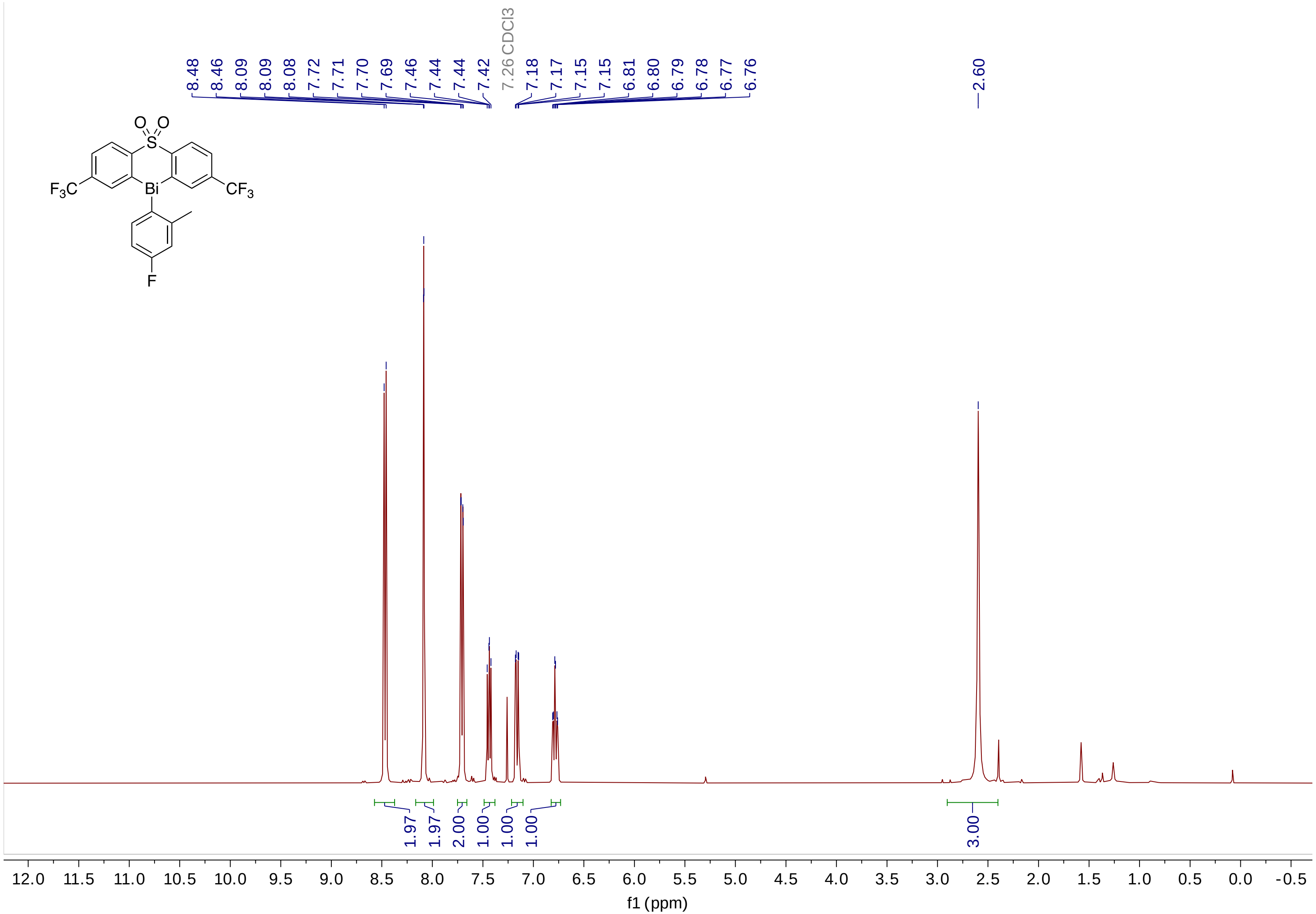

S2a - $^{13}C\{^1H\}$ NMR (101 MHz, $CDCl_3$):

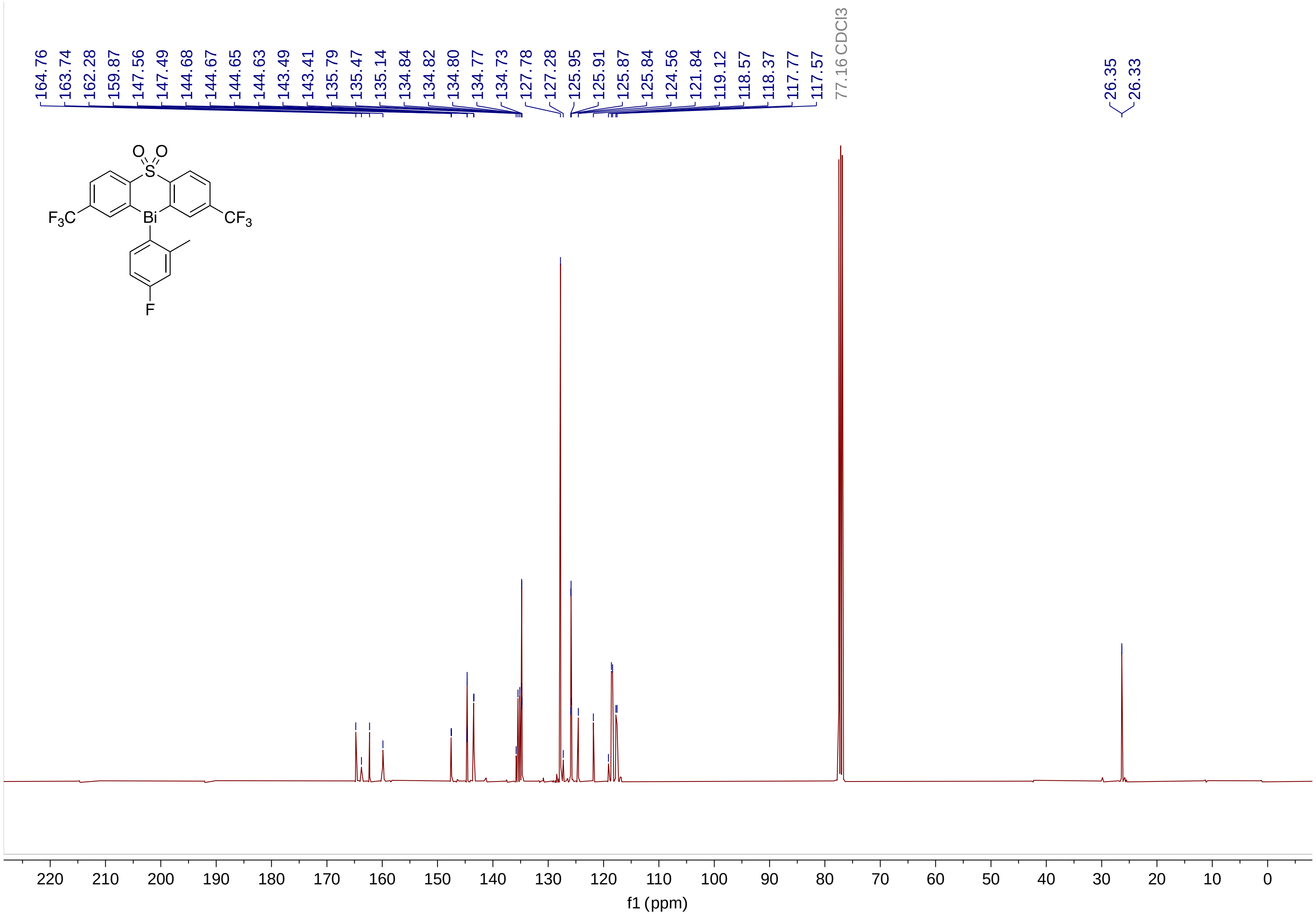

S2a - ^{19}F NMR (376 MHz, CDCl$_3$):

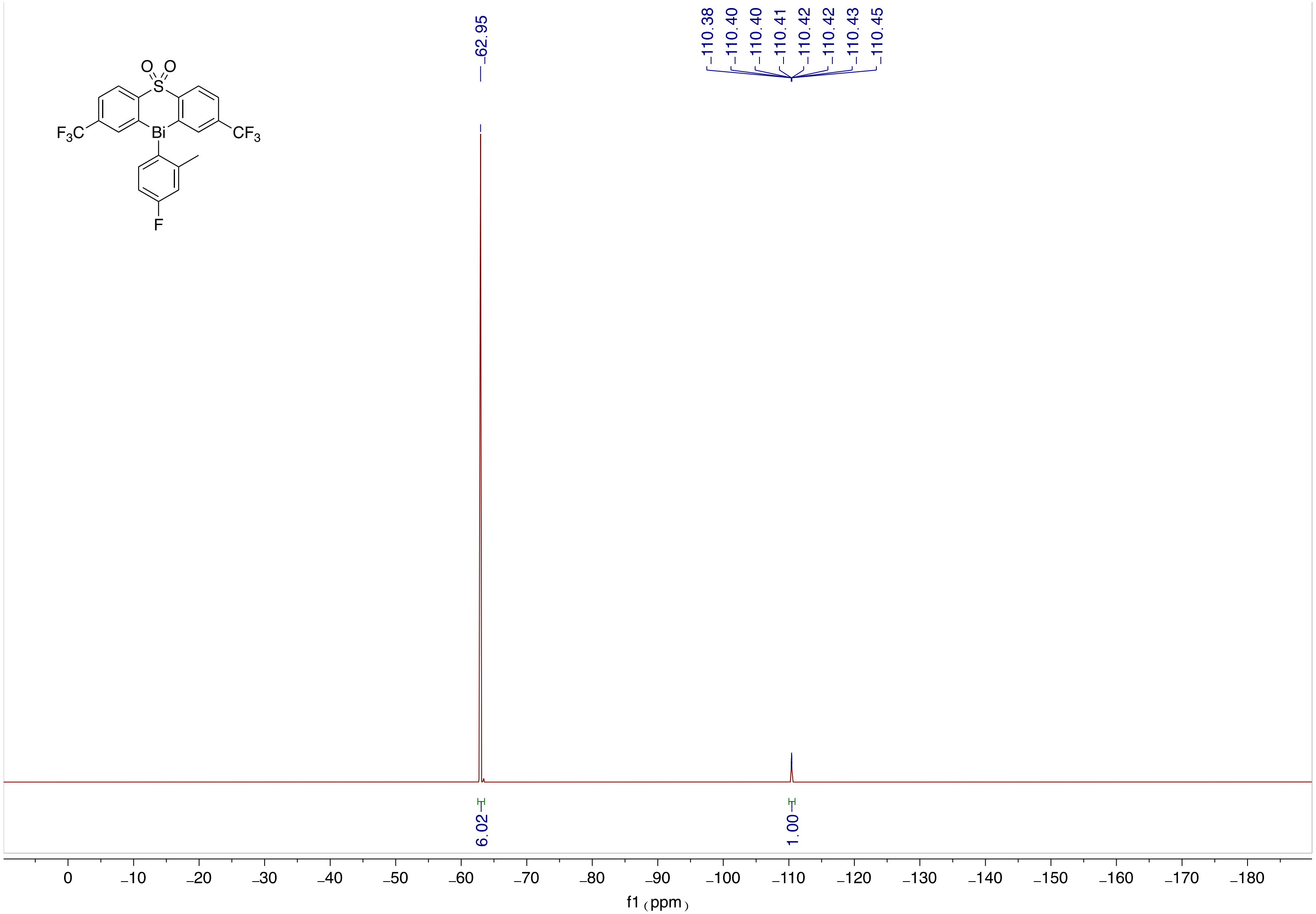

S3a - ^{1}H NMR (400 MHz, CDCl$_3$):

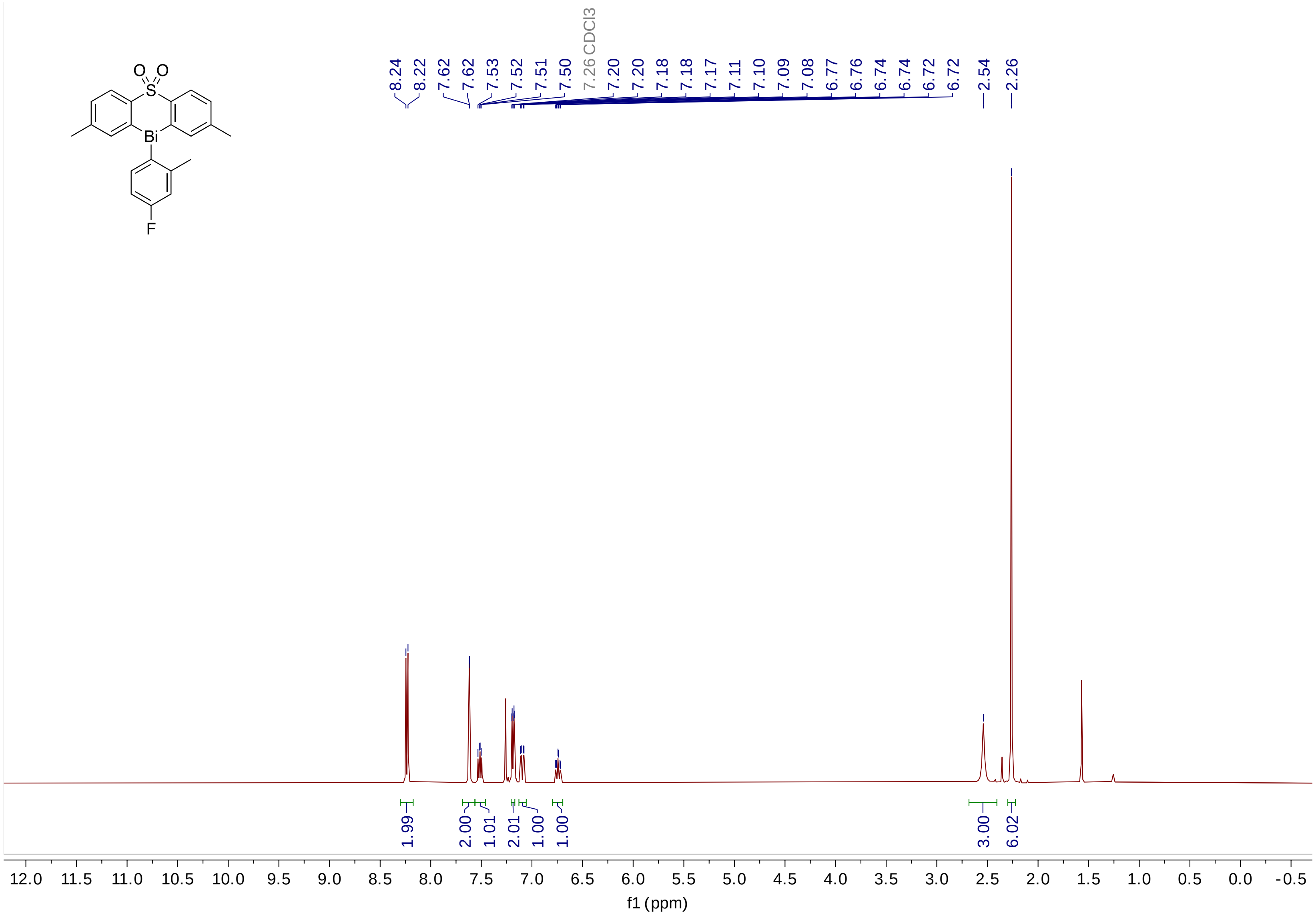

S3a - $^{13}C\{^{1}H\}$ NMR (101 MHz, $CDCl_3$):

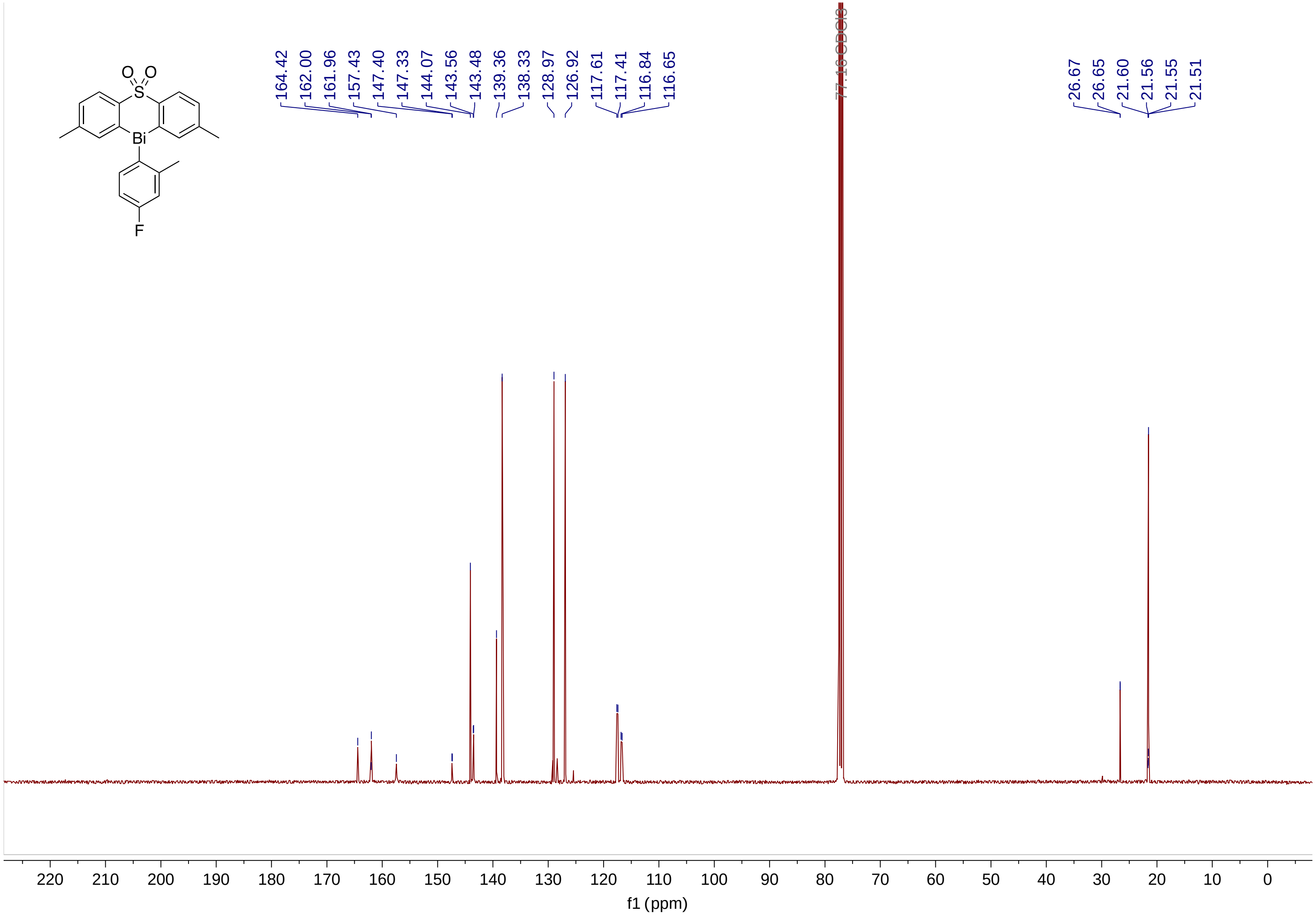

S3a - ^{19}F NMR (377 MHz, $CDCl_3$):

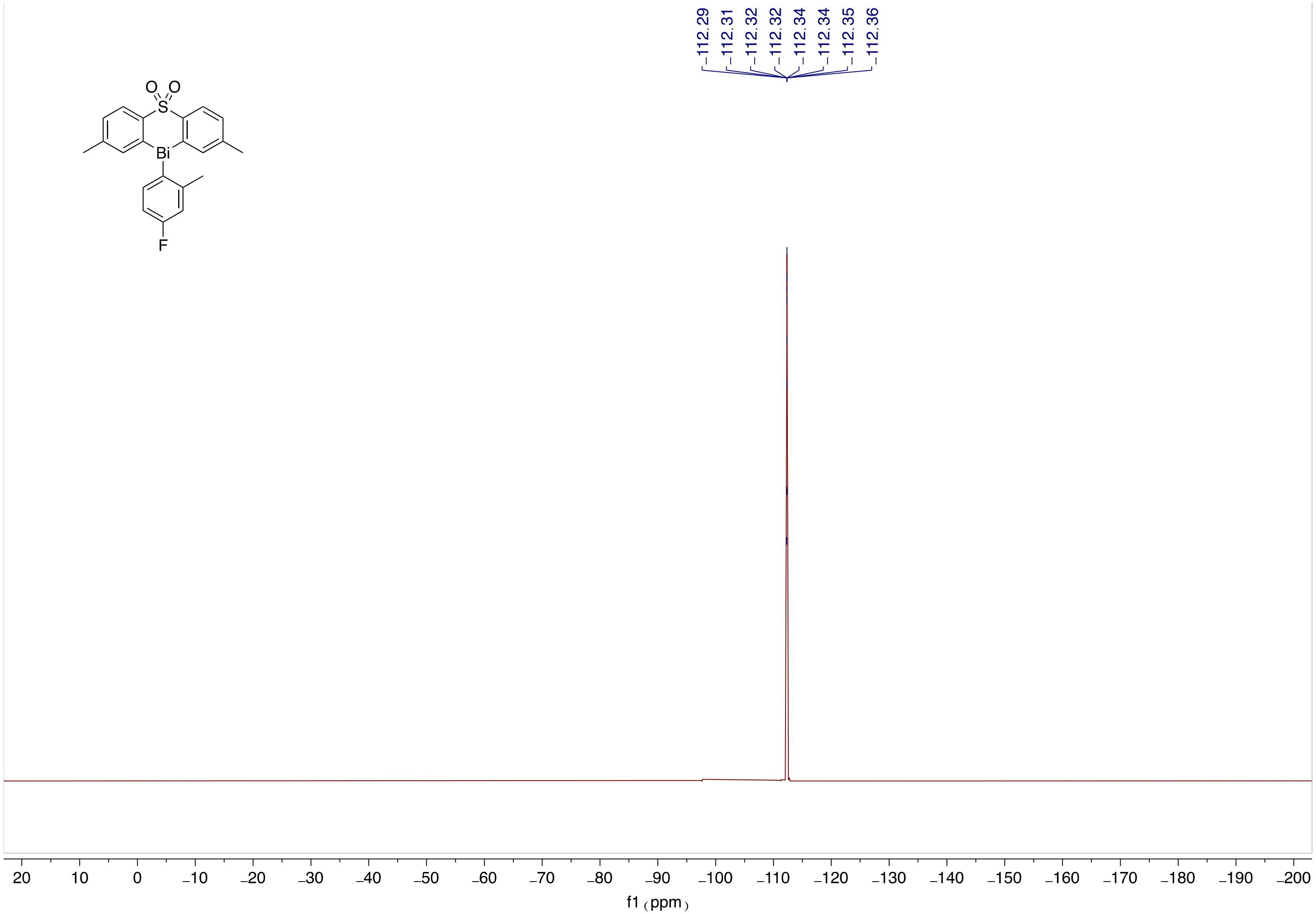

S4a - ^{1}H NMR (400 MHz, $CDCl_3$):

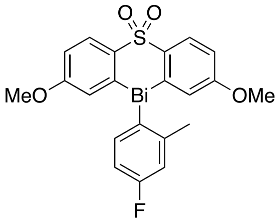

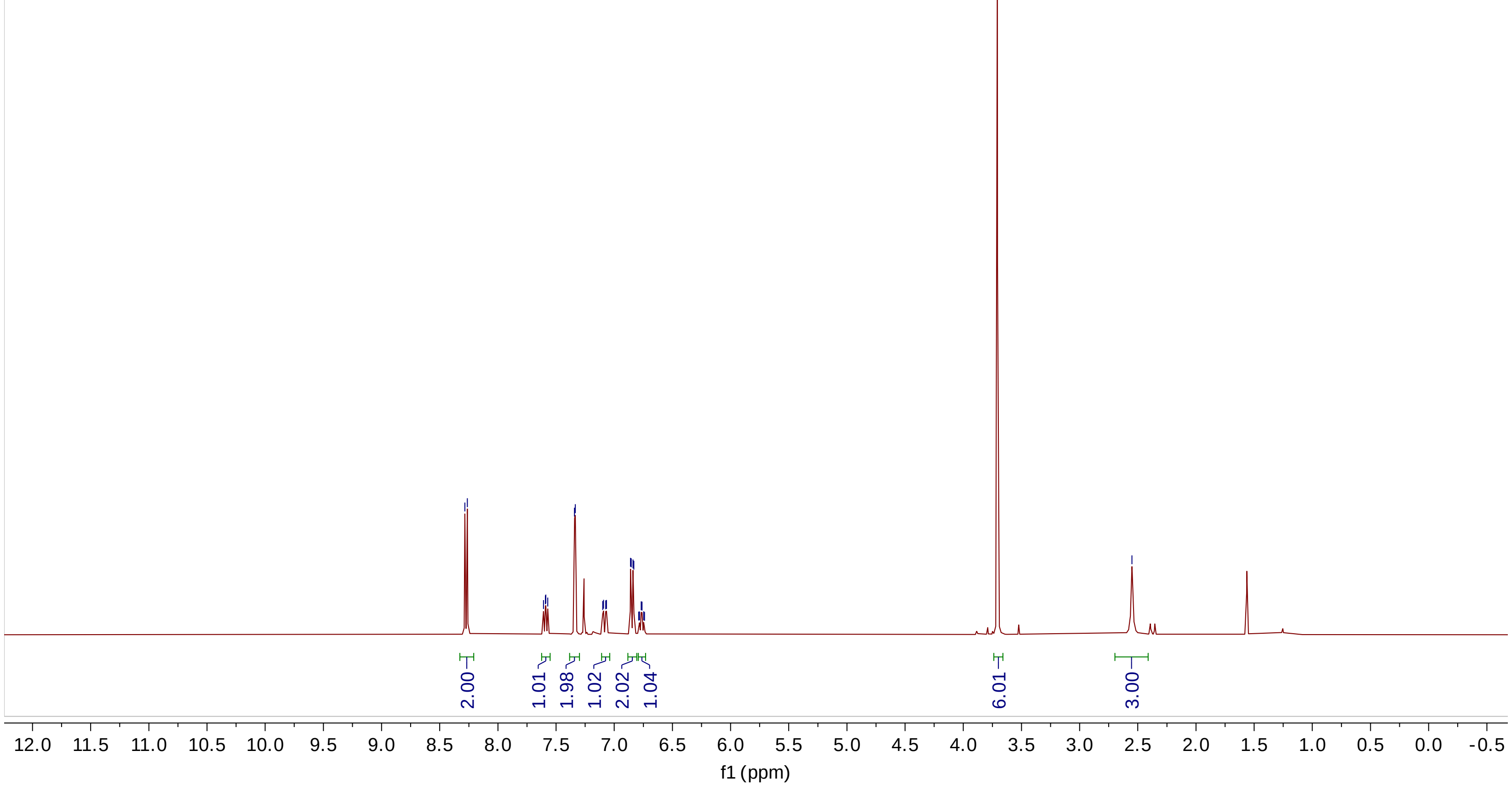

S4a - $^{13}C\{^{1}H\}$ NMR (101 MHz, CDCl$_3$):

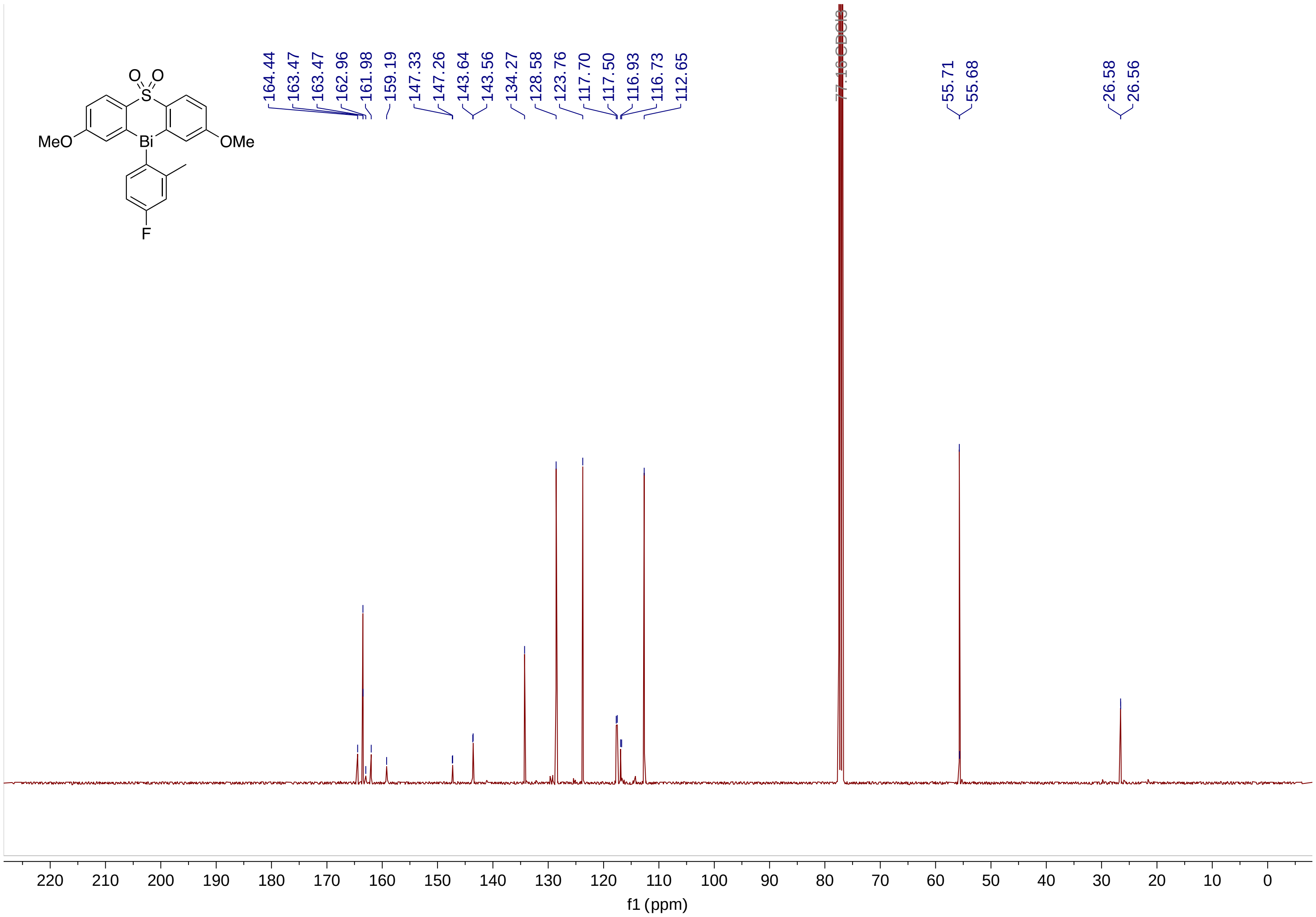

S4a - ^{19}F NMR (376 MHz, $CDCl_3$):

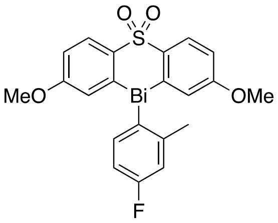

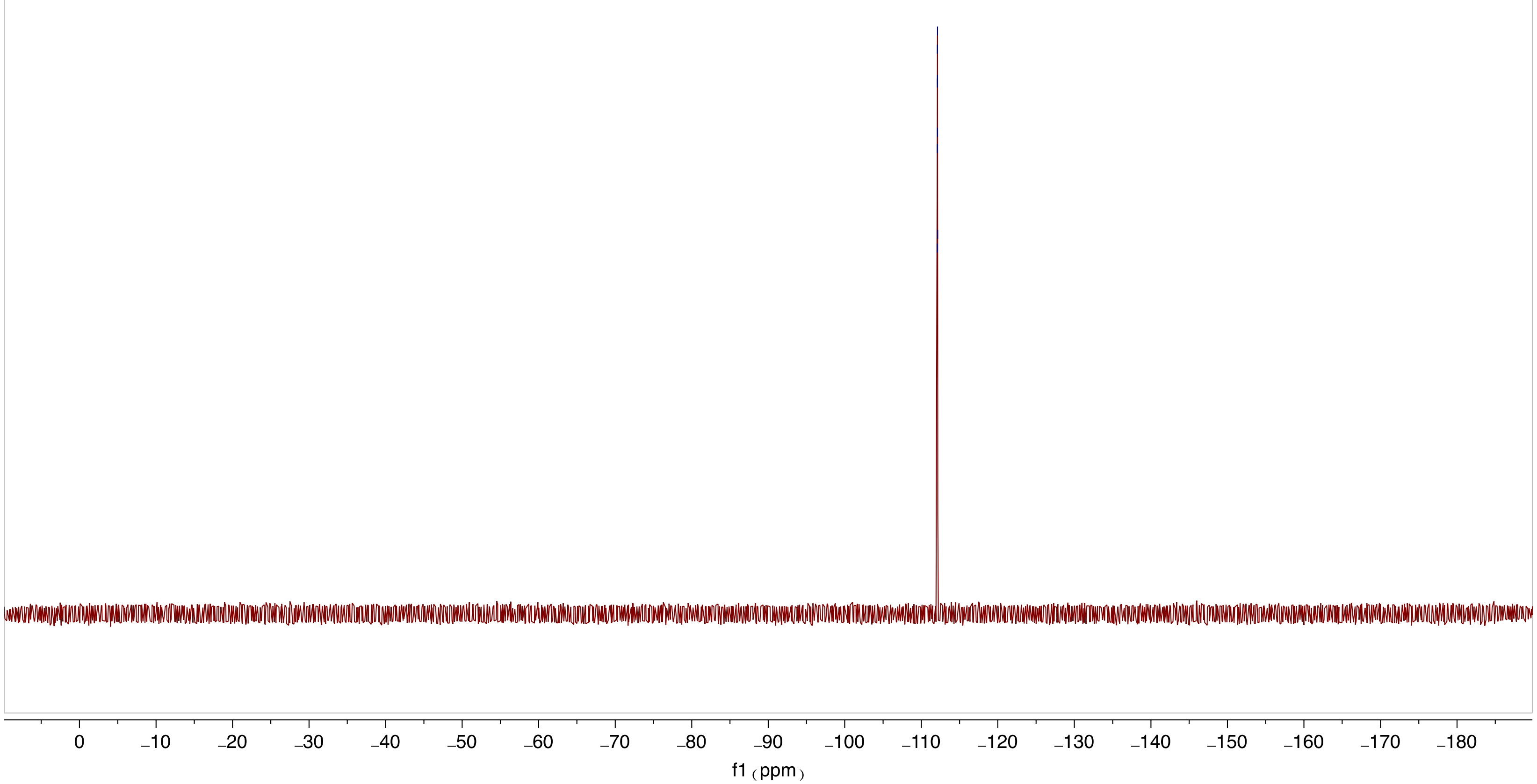

S5a - ^{1}H NMR (400 MHz, $CDCl_3$):

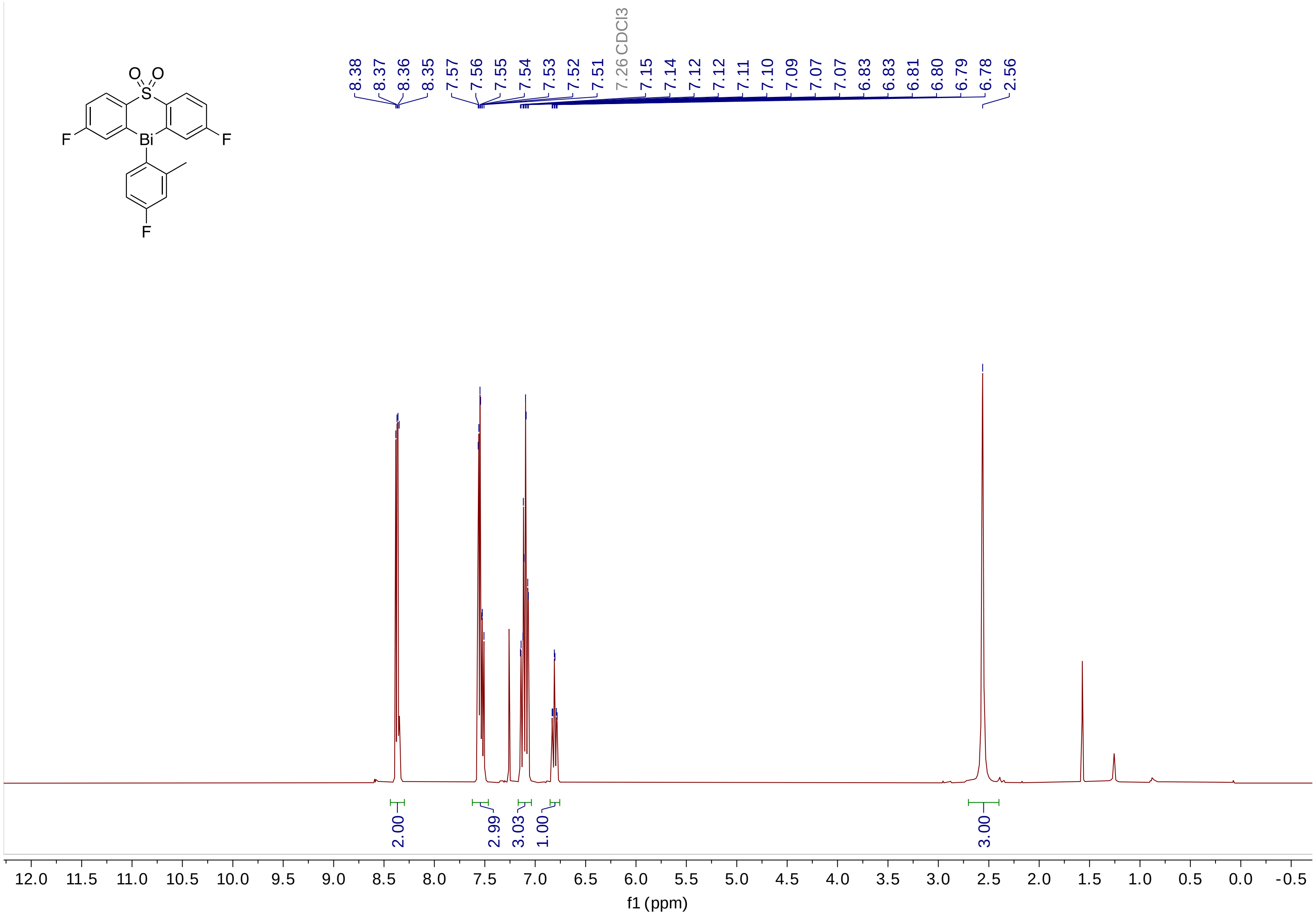

S5a - $^{13}C\{^{1}H\}$ NMR (101 MHz, CDCl$_3$):

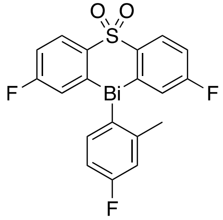

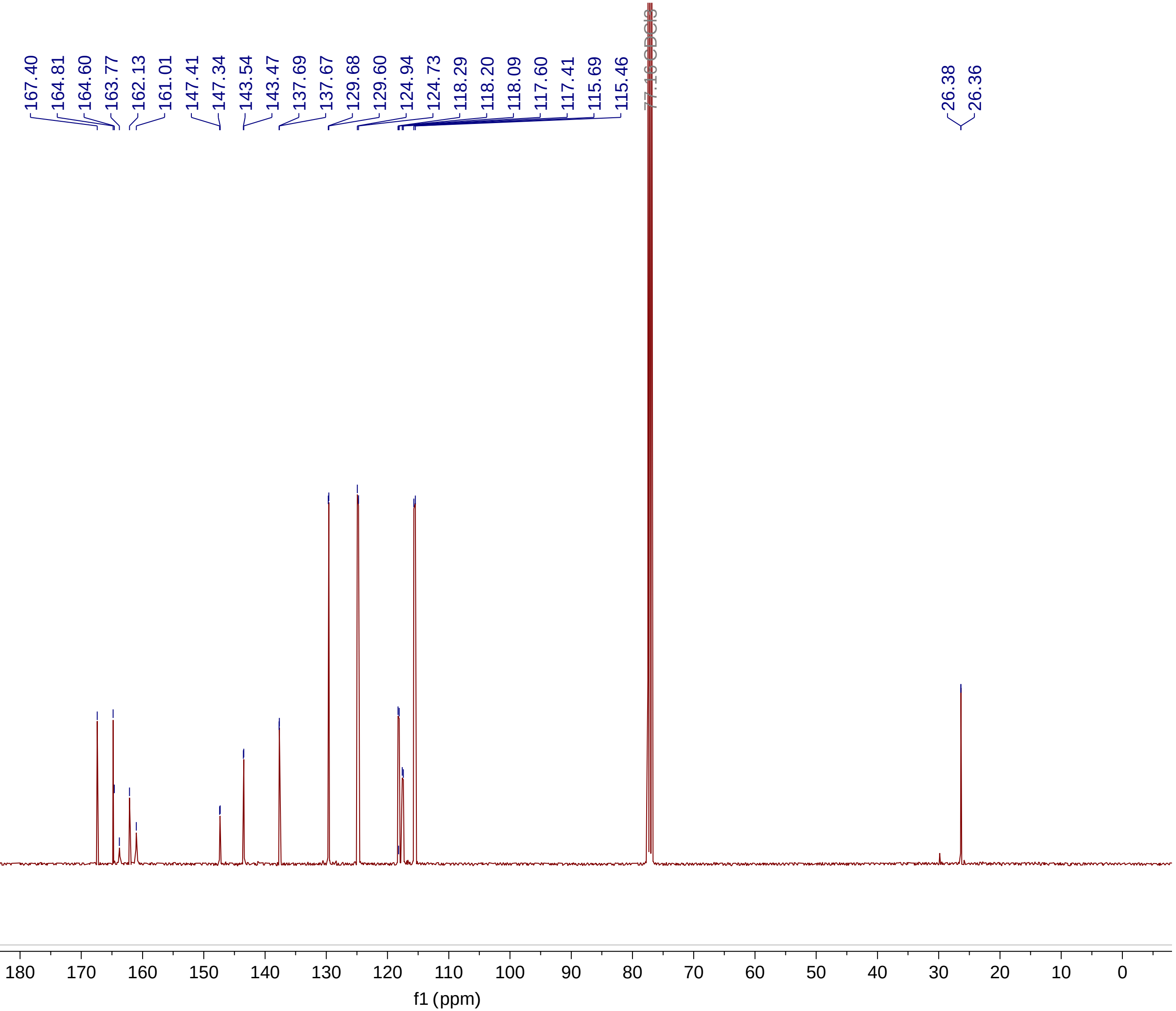

S5a - ^{19}F NMR (376 MHz, $CDCl_3$):

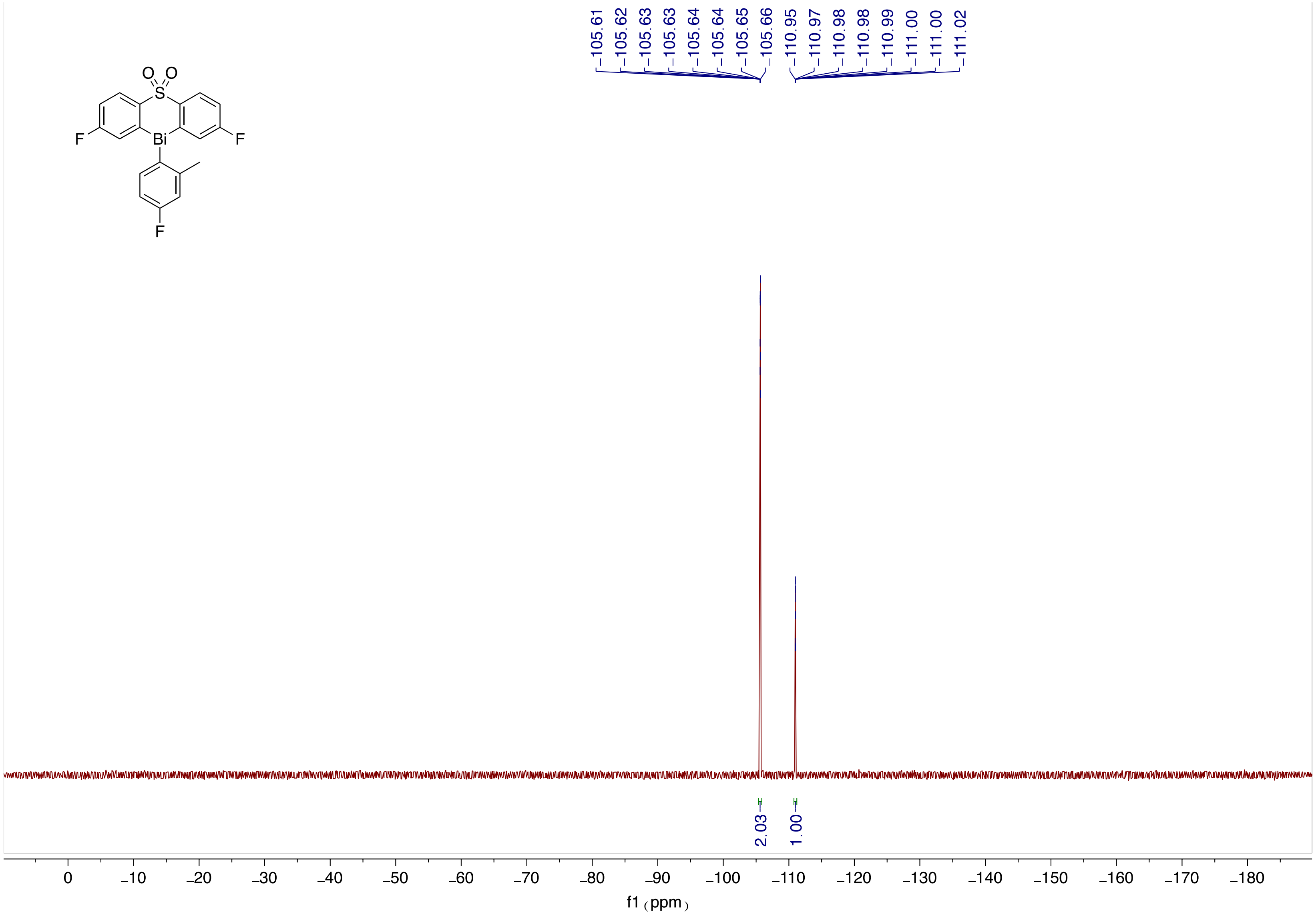

S6a - ^{1}H NMR (400 MHz, $CDCl_3$):

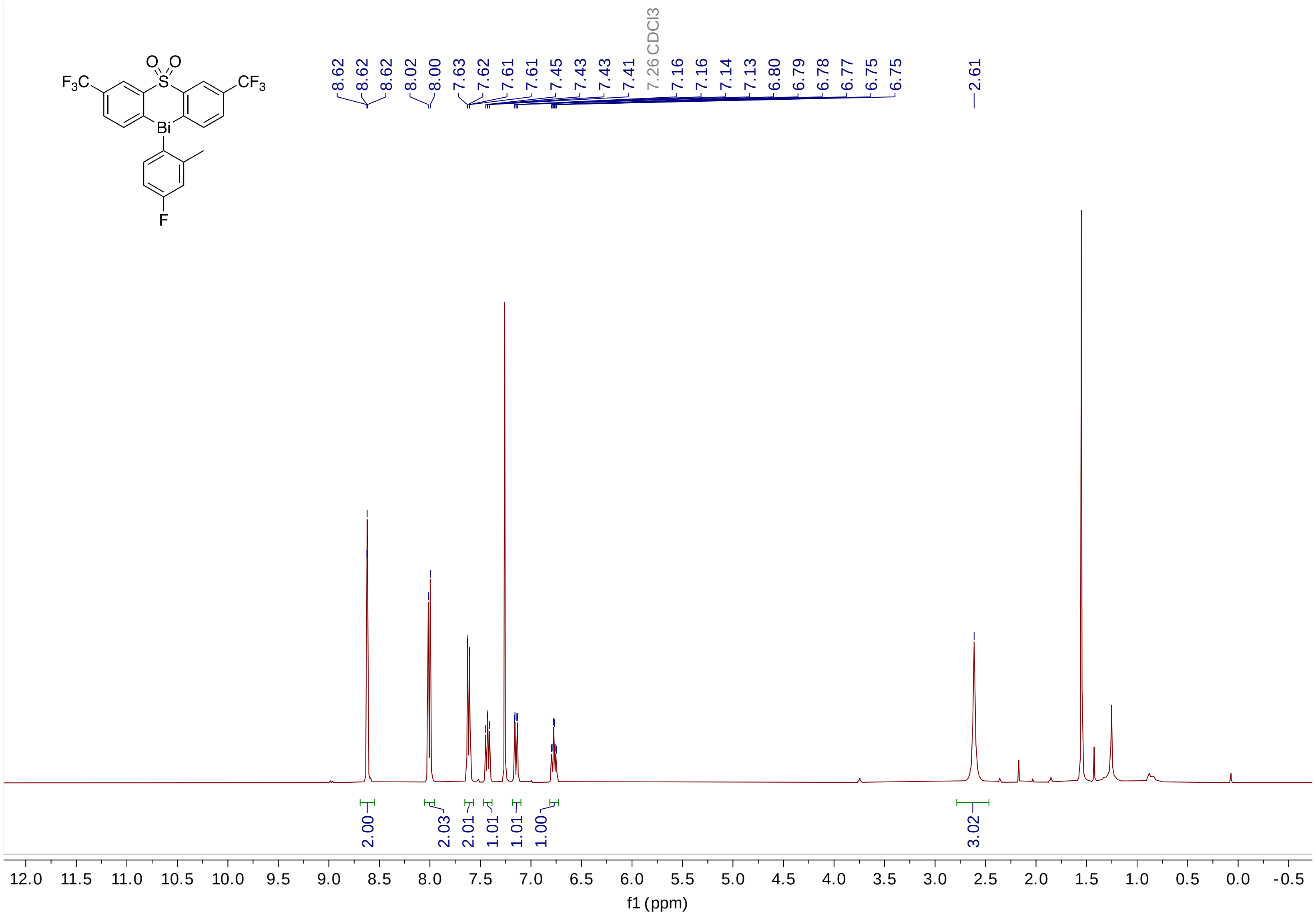

S6a - $^{13}C\{^{1}H\}$ NMR (101 MHz, $CDCl_3$):

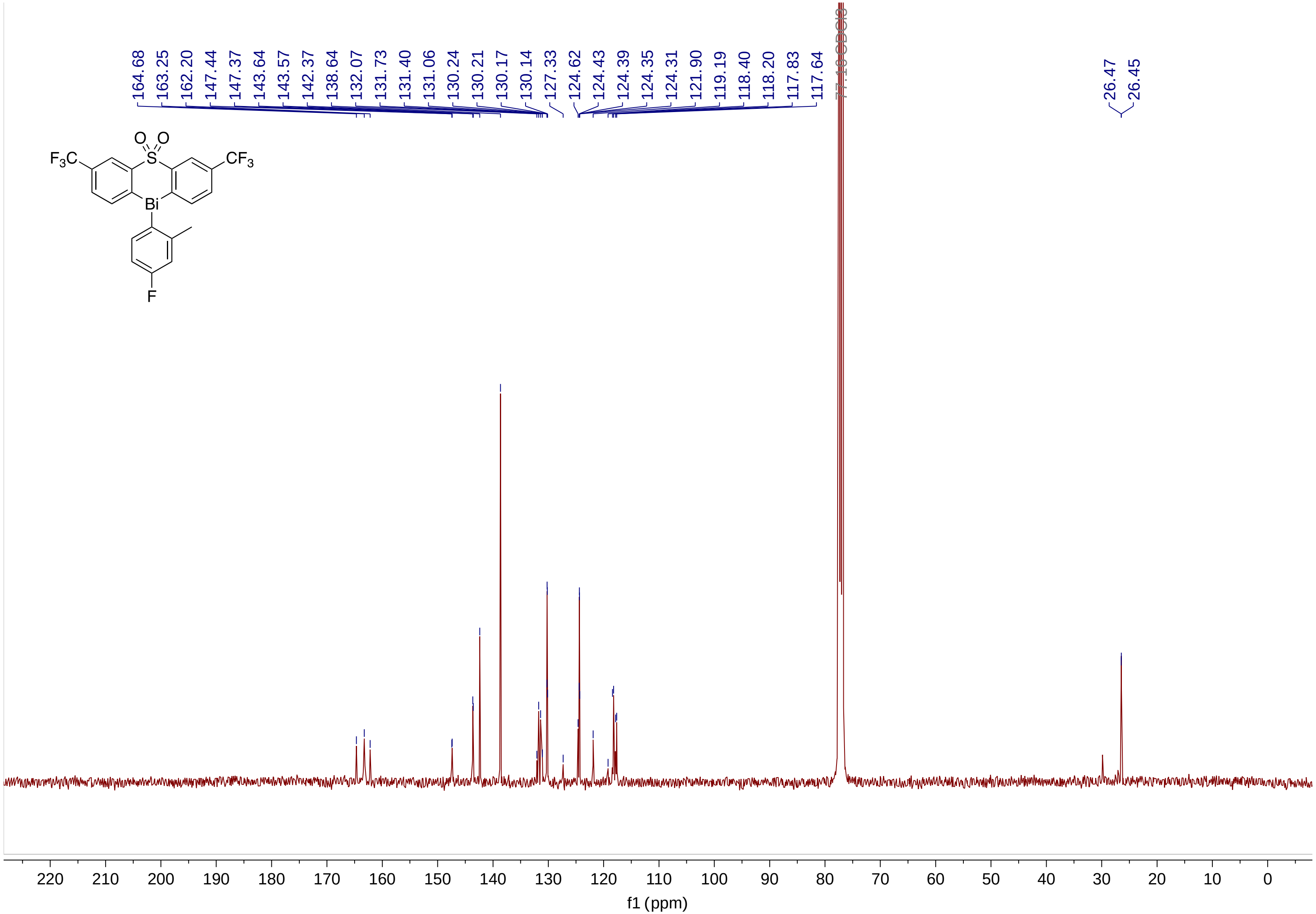

S6a - ^{19}F NMR (377 MHz, CDCl$_3$):

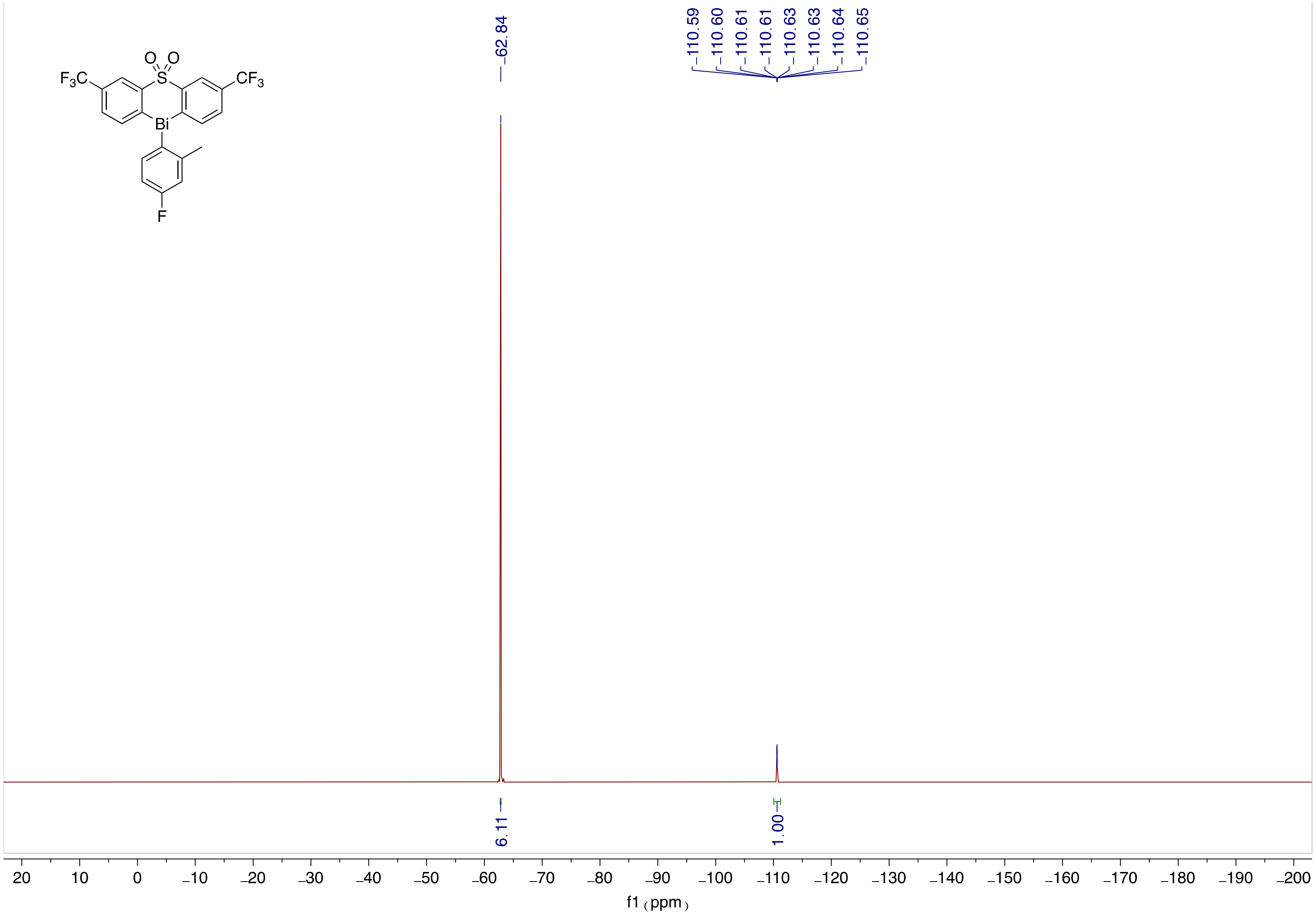

^{1}H NMR (400 MHz, $CDCl_3$):

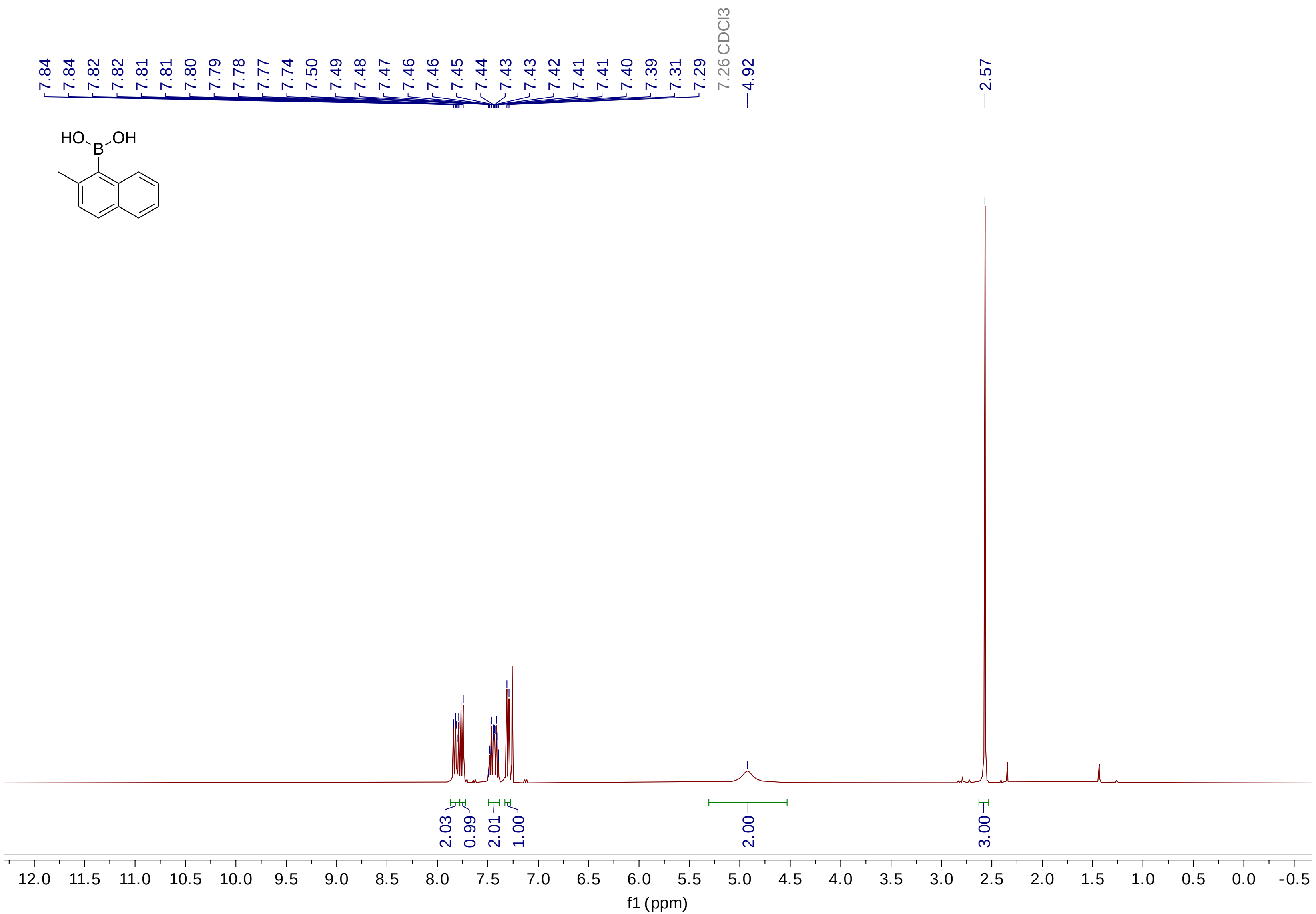

$^{13}C\{^1H\}$ NMR (101 MHz, $CDCl_3$):

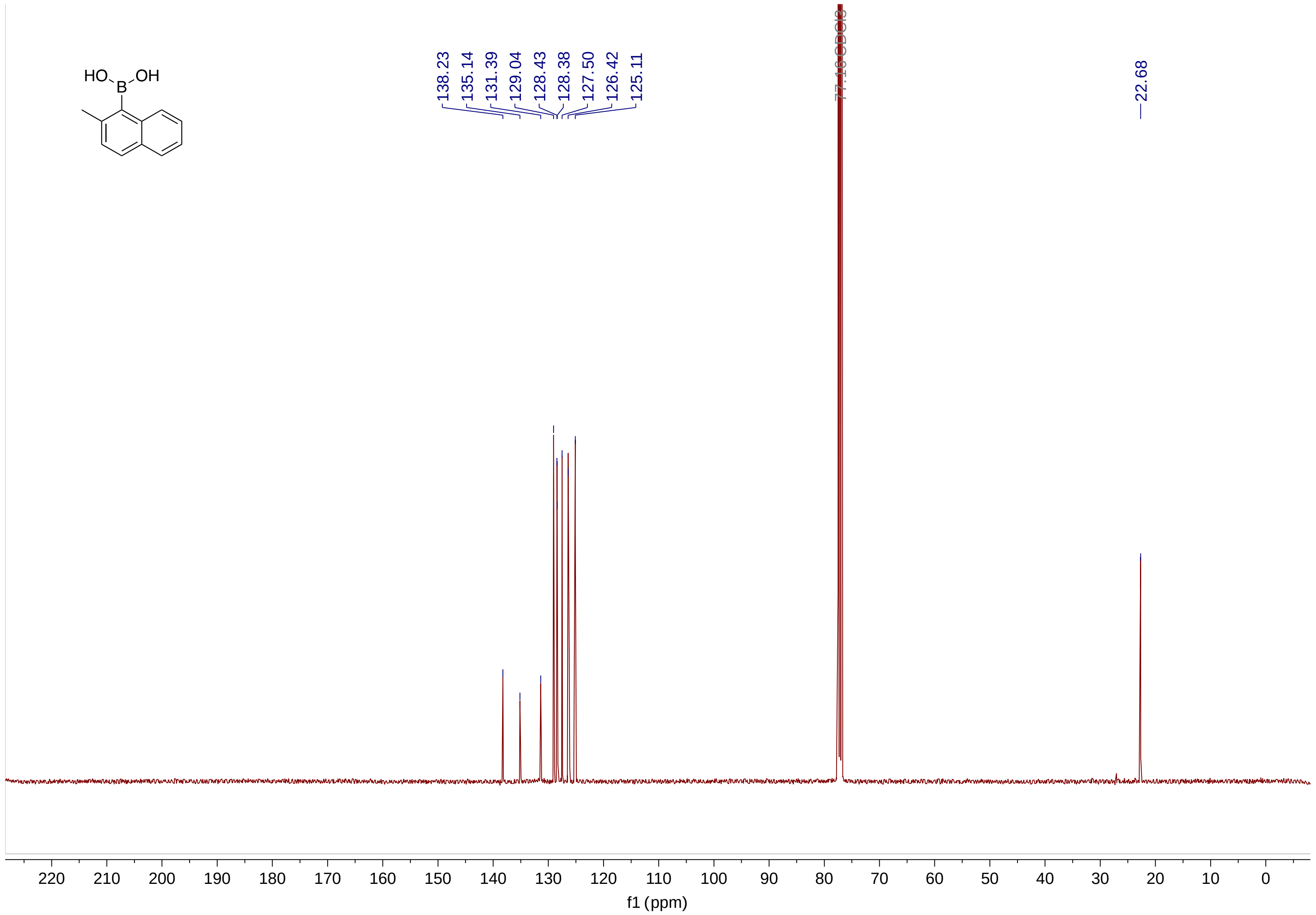

^{1}H NMR (400 MHz, CDCl3):

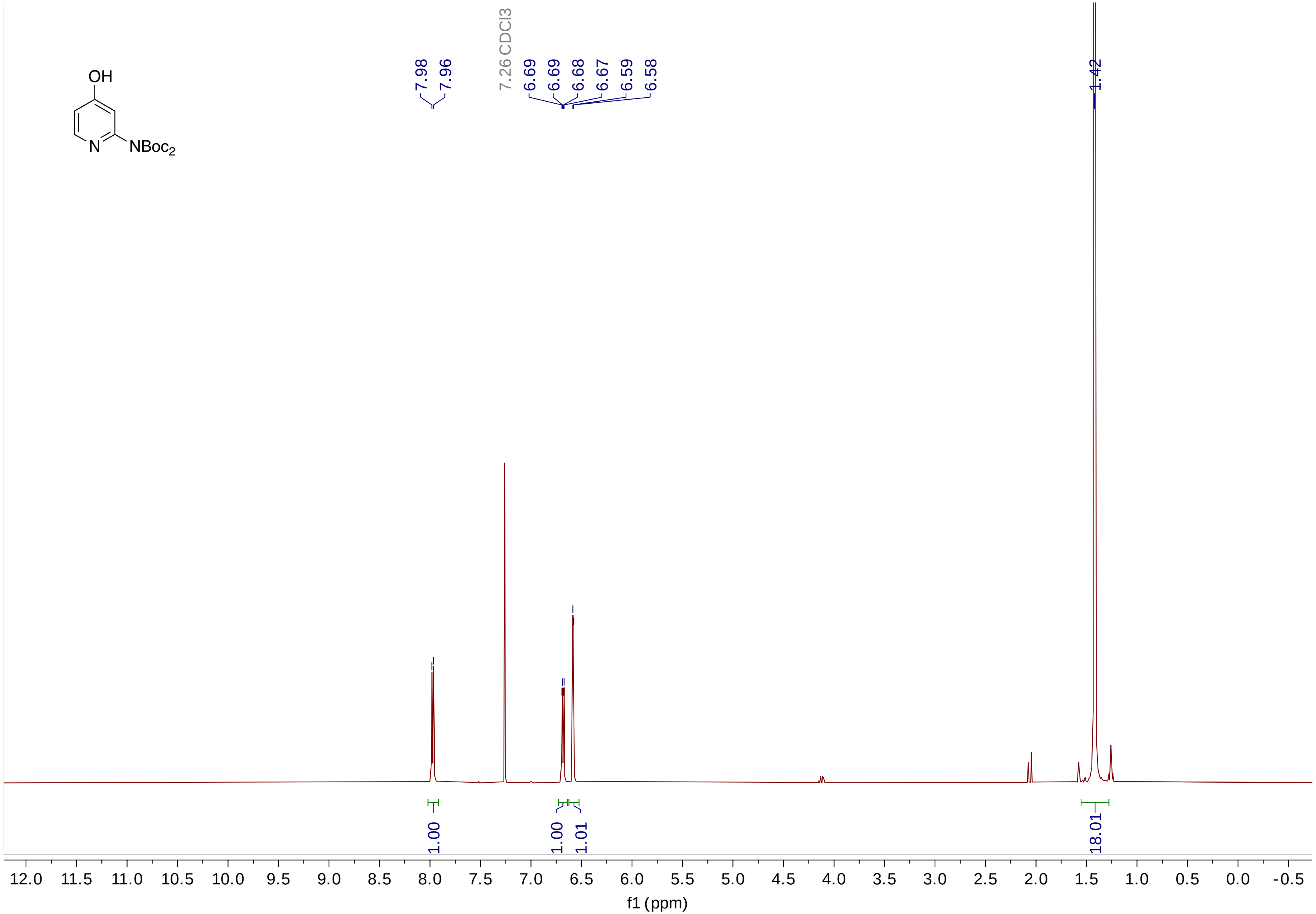

$^{13}C\{^1H\}$ NMR (101 MHz, $CDCl_3$):

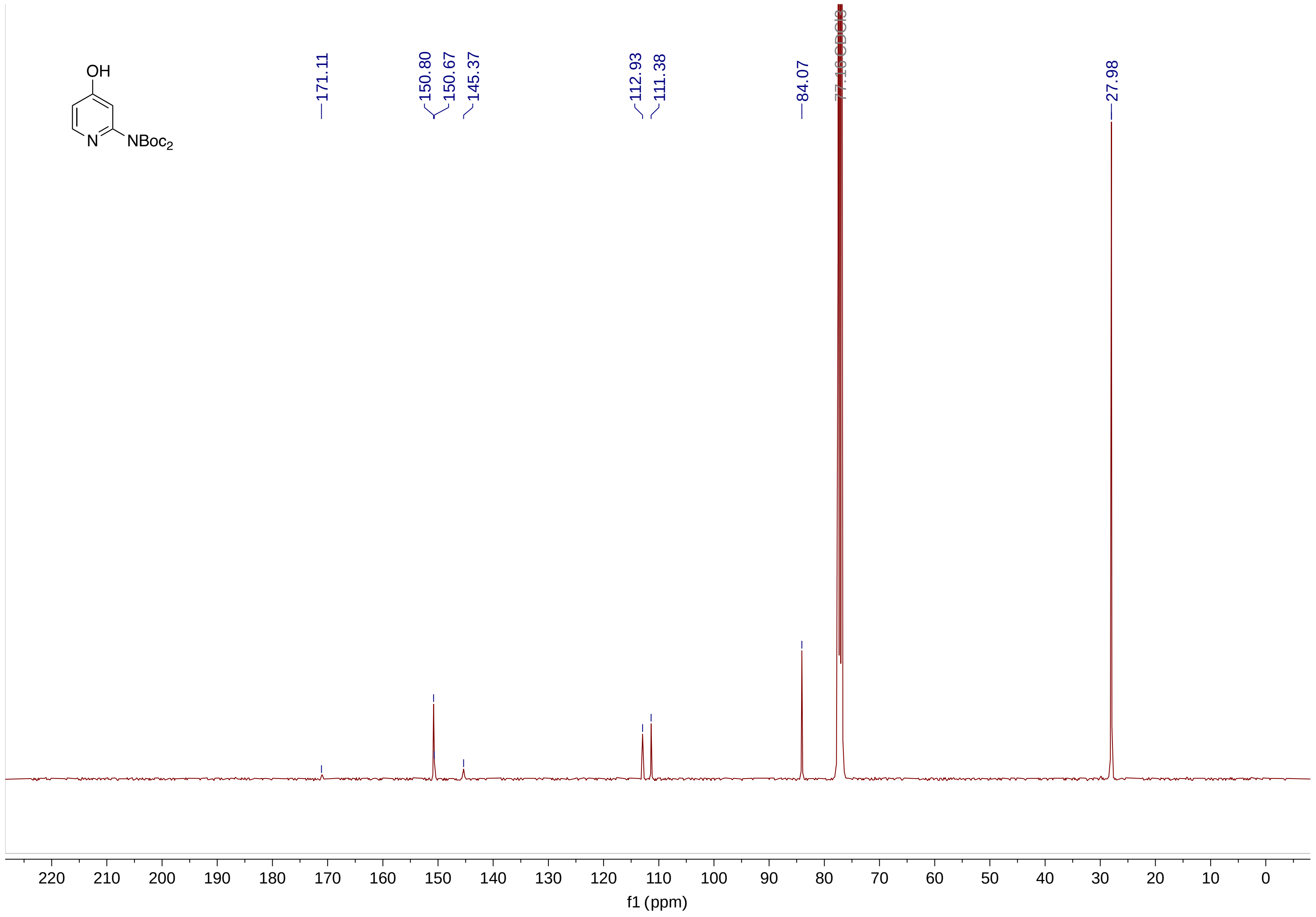

^{1}H NMR (400 MHz, CD_3CN):

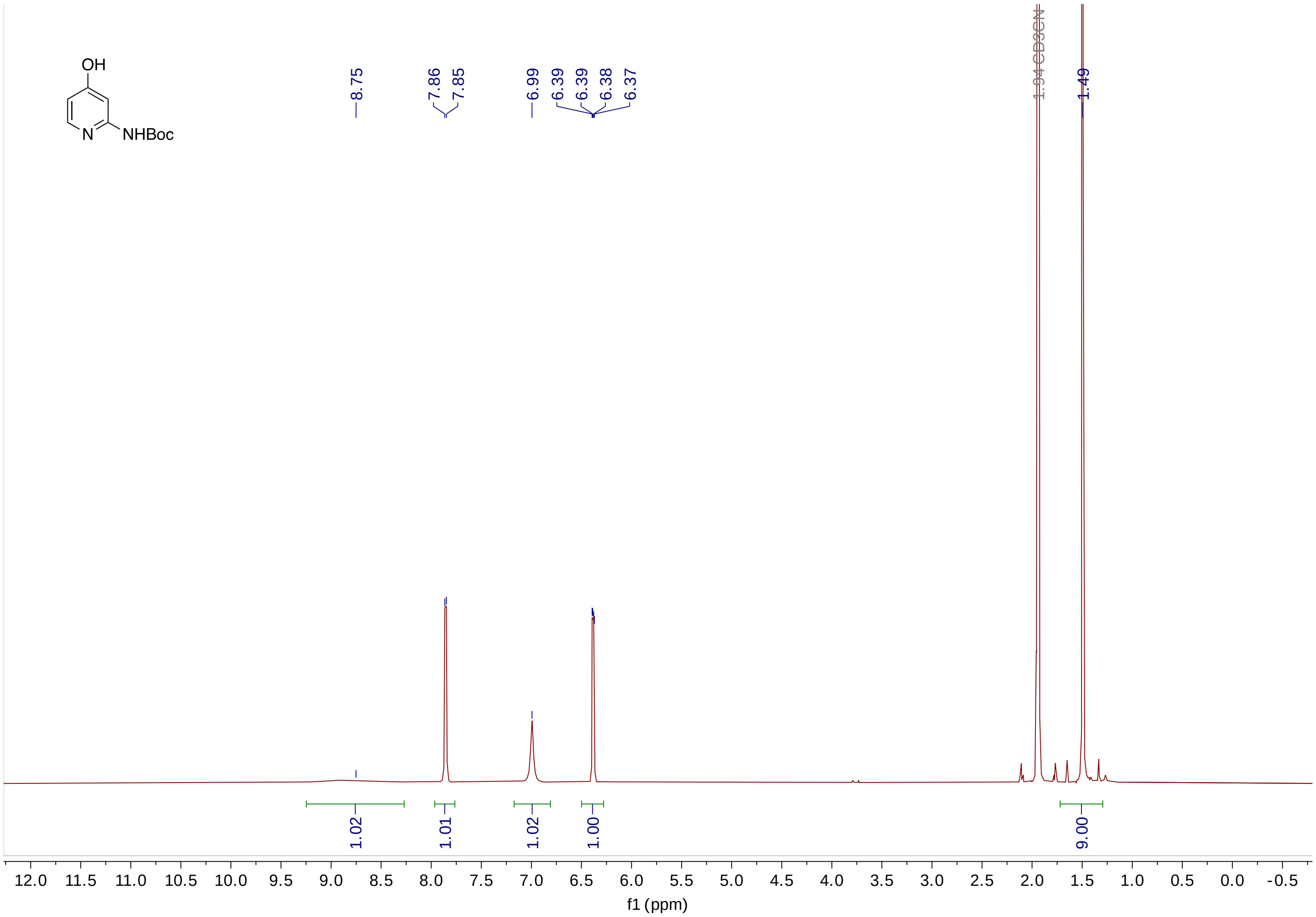

$^{13}C\{^1H\}$ NMR (101 MHz, CD_3CN):

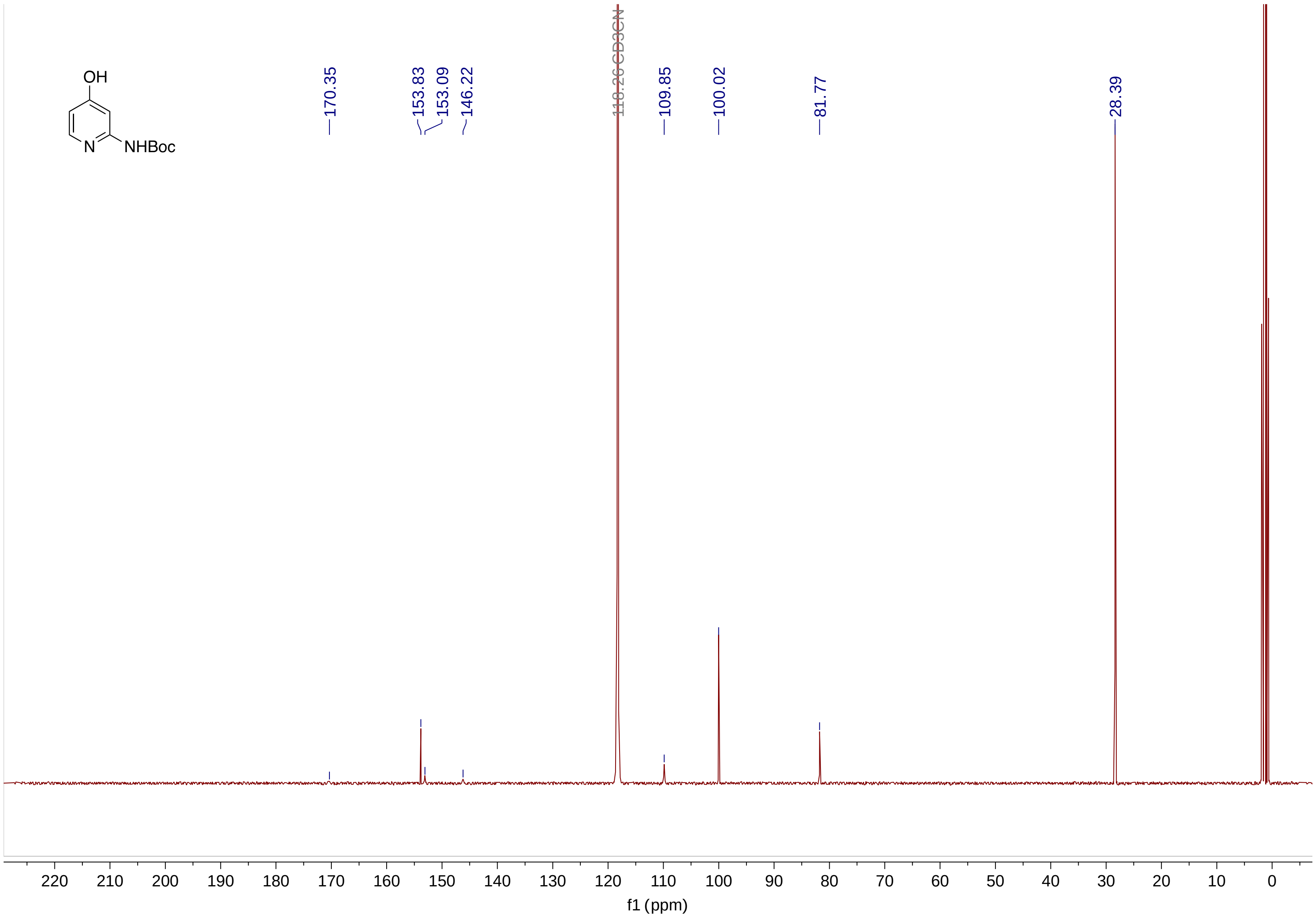

^{1}H NMR (400 MHz, DMSO-d_6):

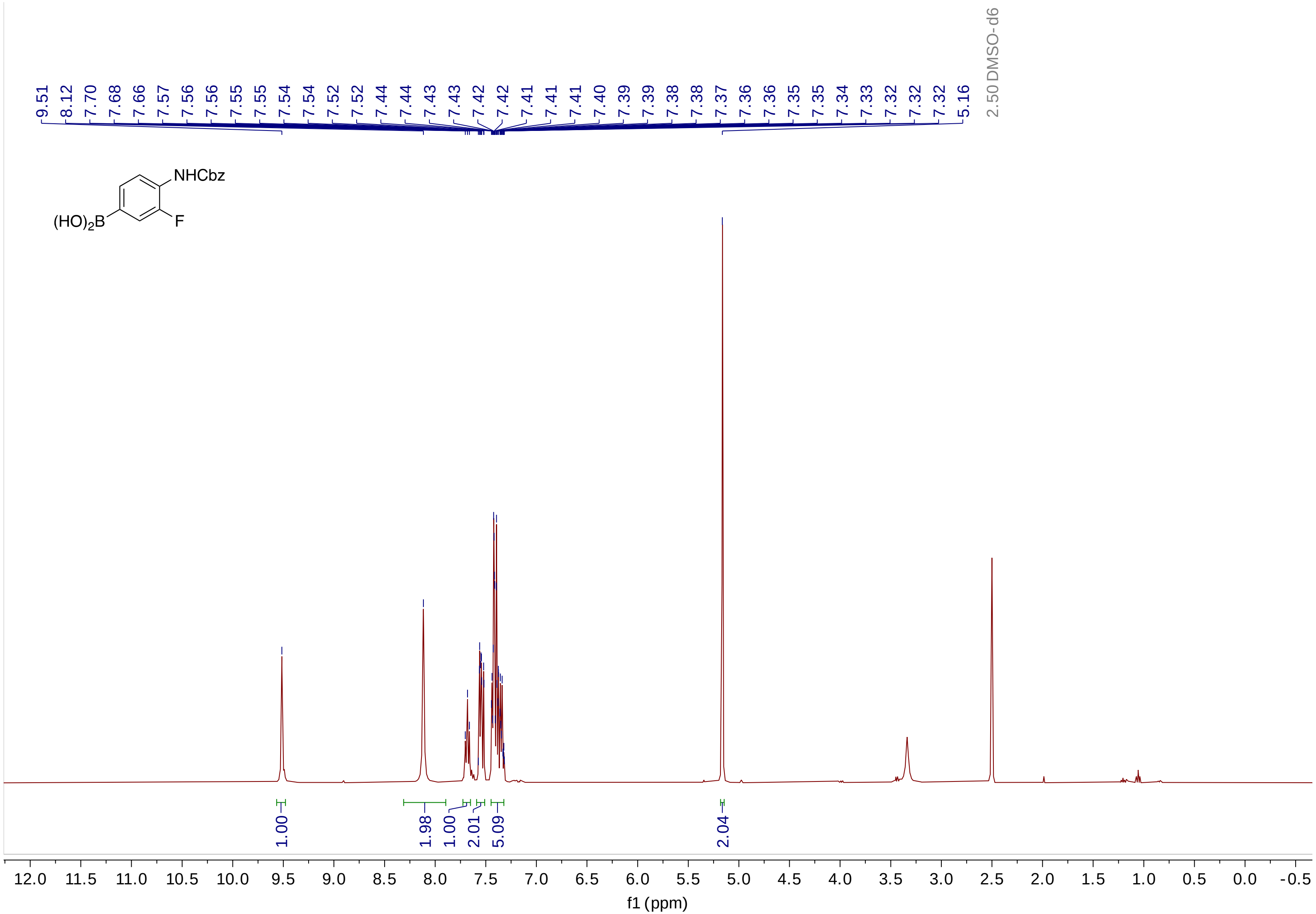

$^{13}C\{^1H\}$ NMR (101 MHz, DMSO-d_6):

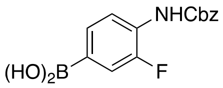

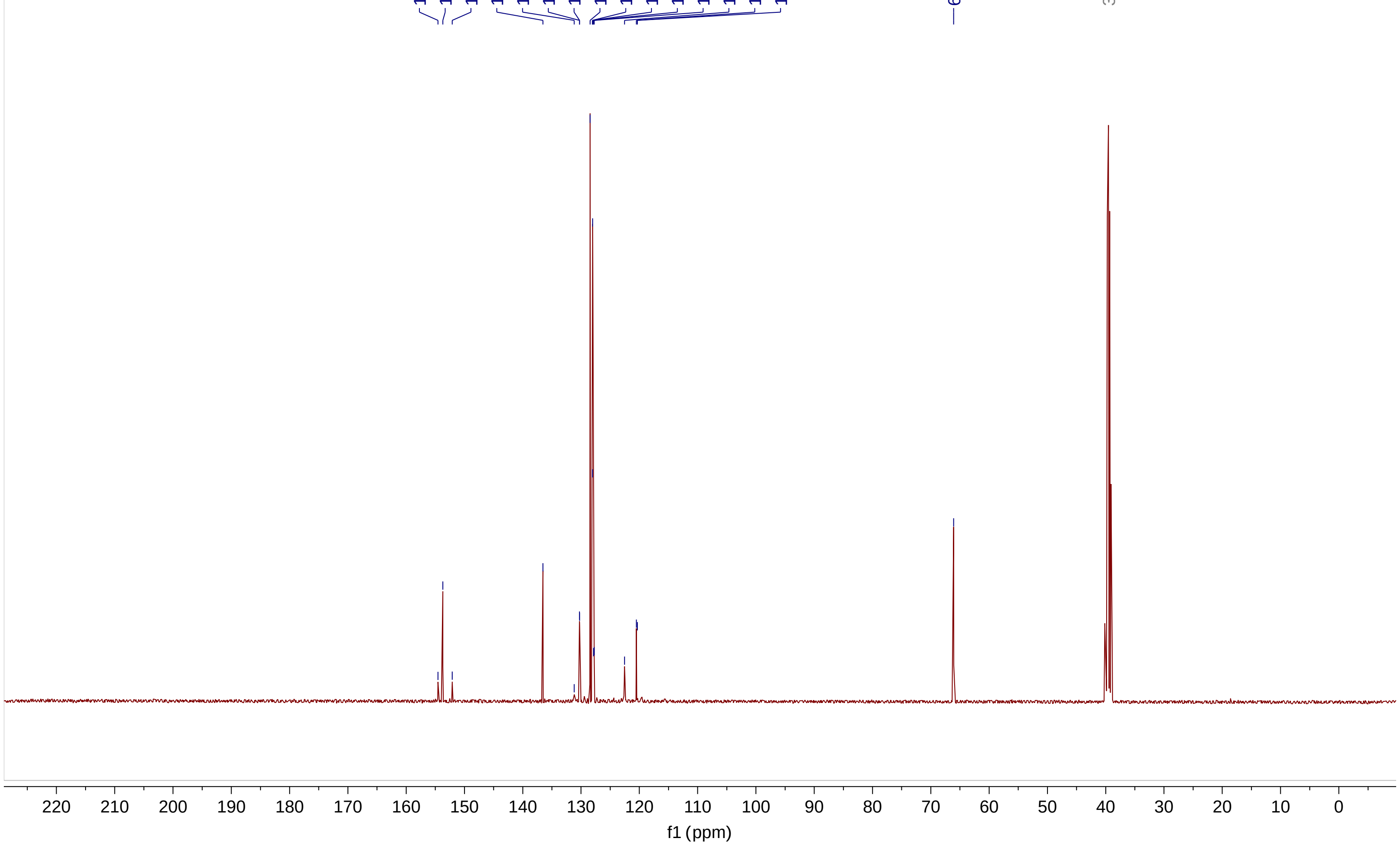

^{19}F NMR (376 MHz, DMSO-d_6):

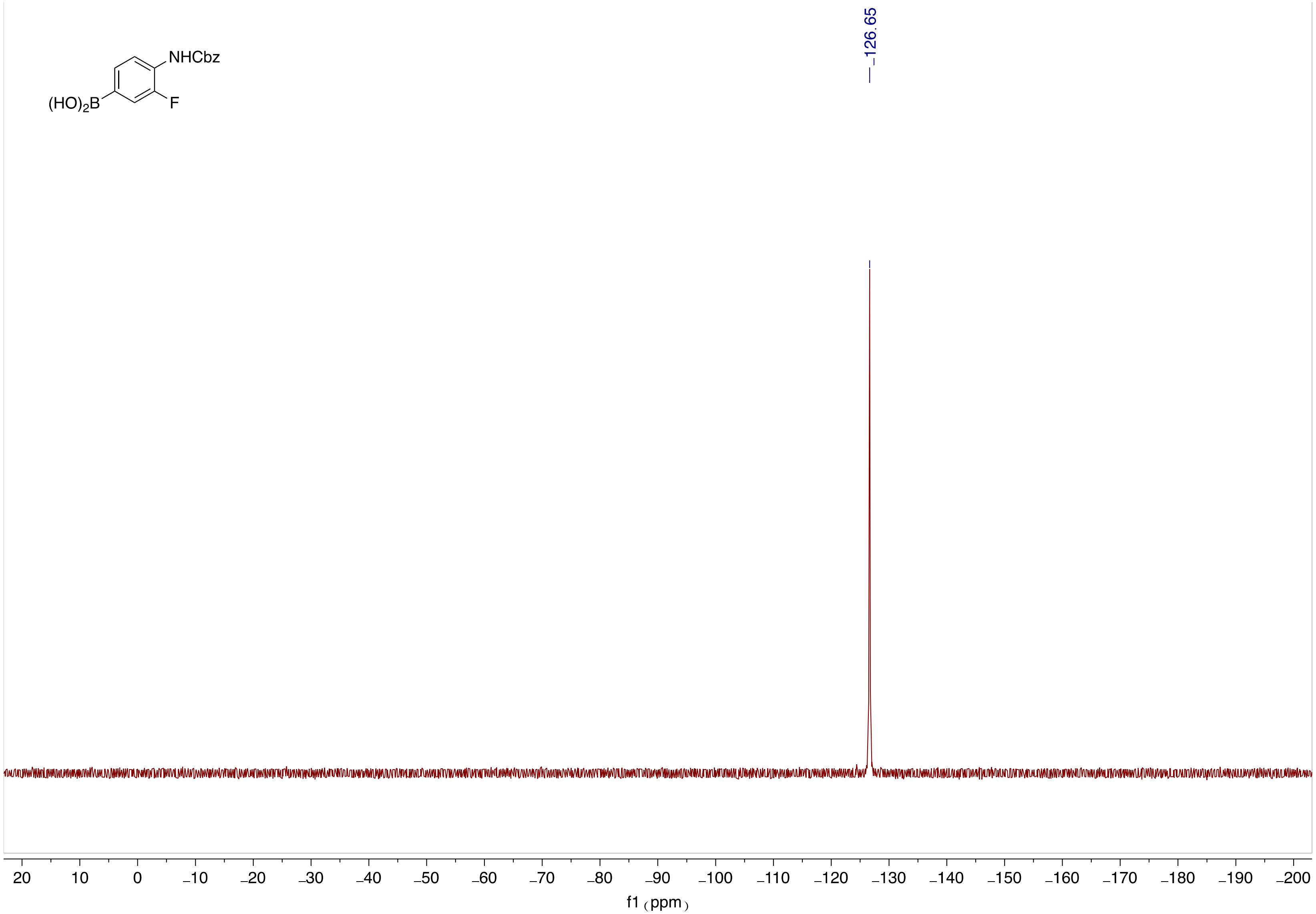

^{1}H NMR (400 MHz, DMSO-d_6):

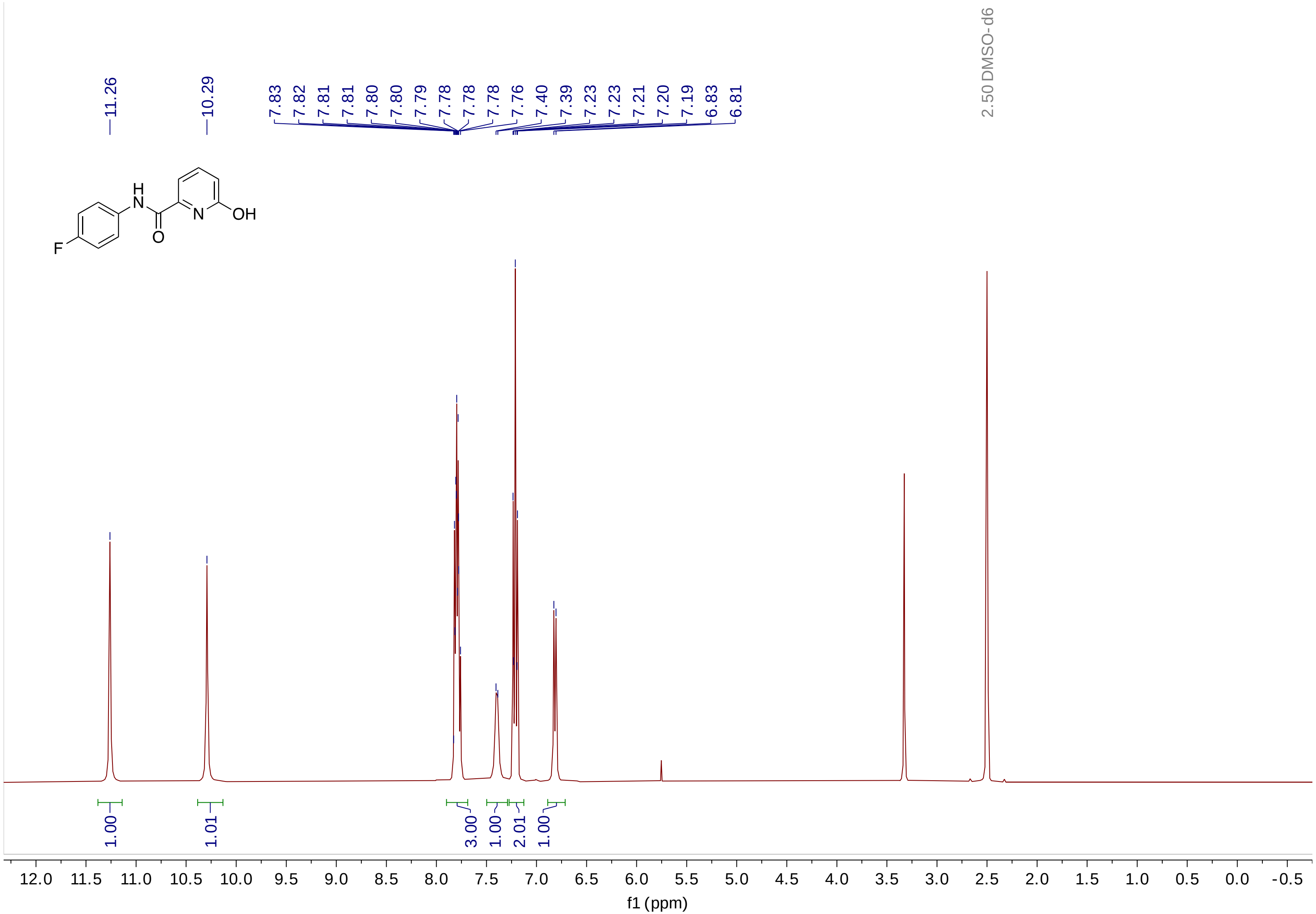

$^{13}C\{^{1}H\}$ NMR (126 MHz, DMSO-*d*$_6$):

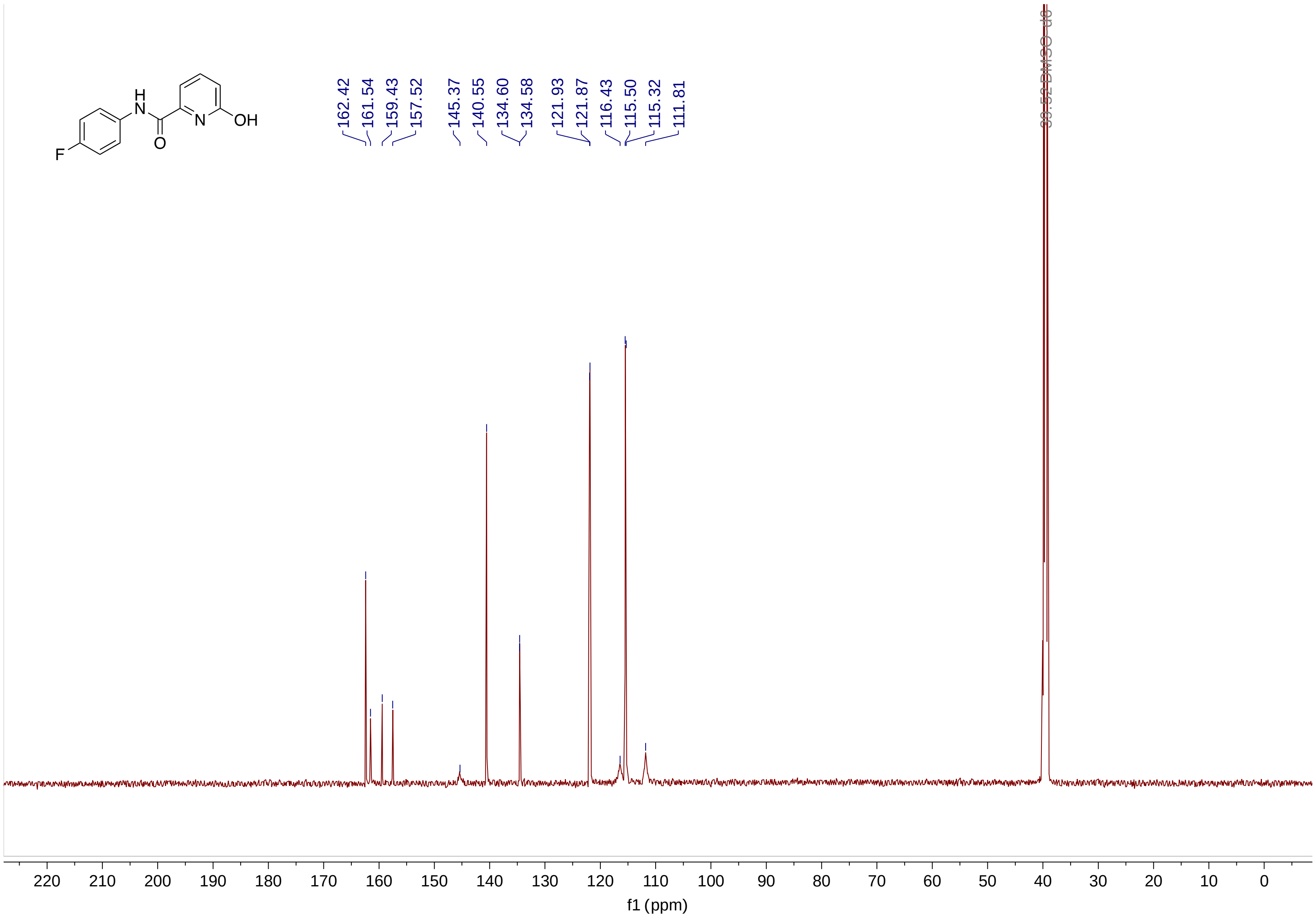

^{19}F NMR (376 MHz, DMSO-d_6):

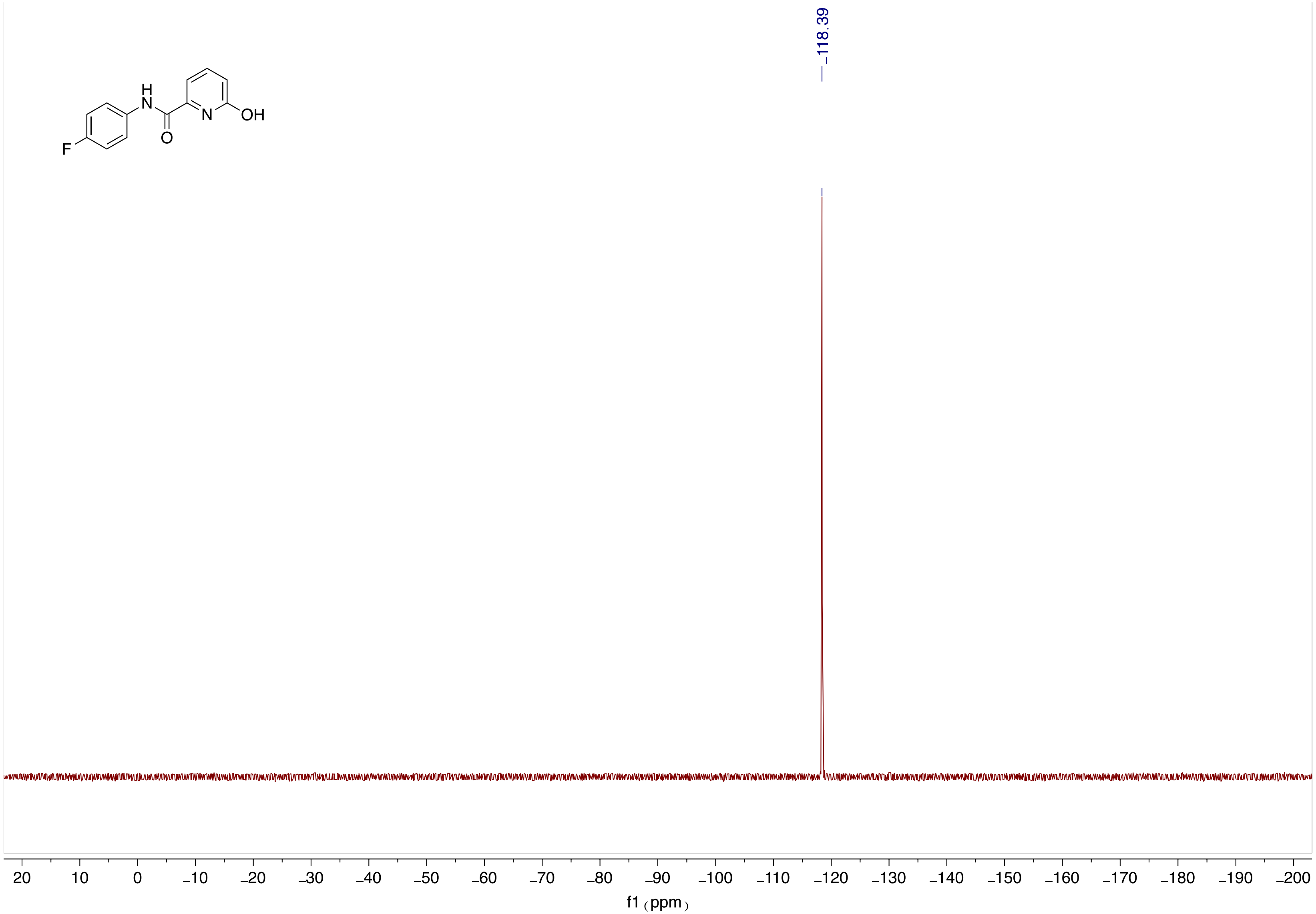

4 - ^{1}H NMR (400 MHz, $CDCl_3$):

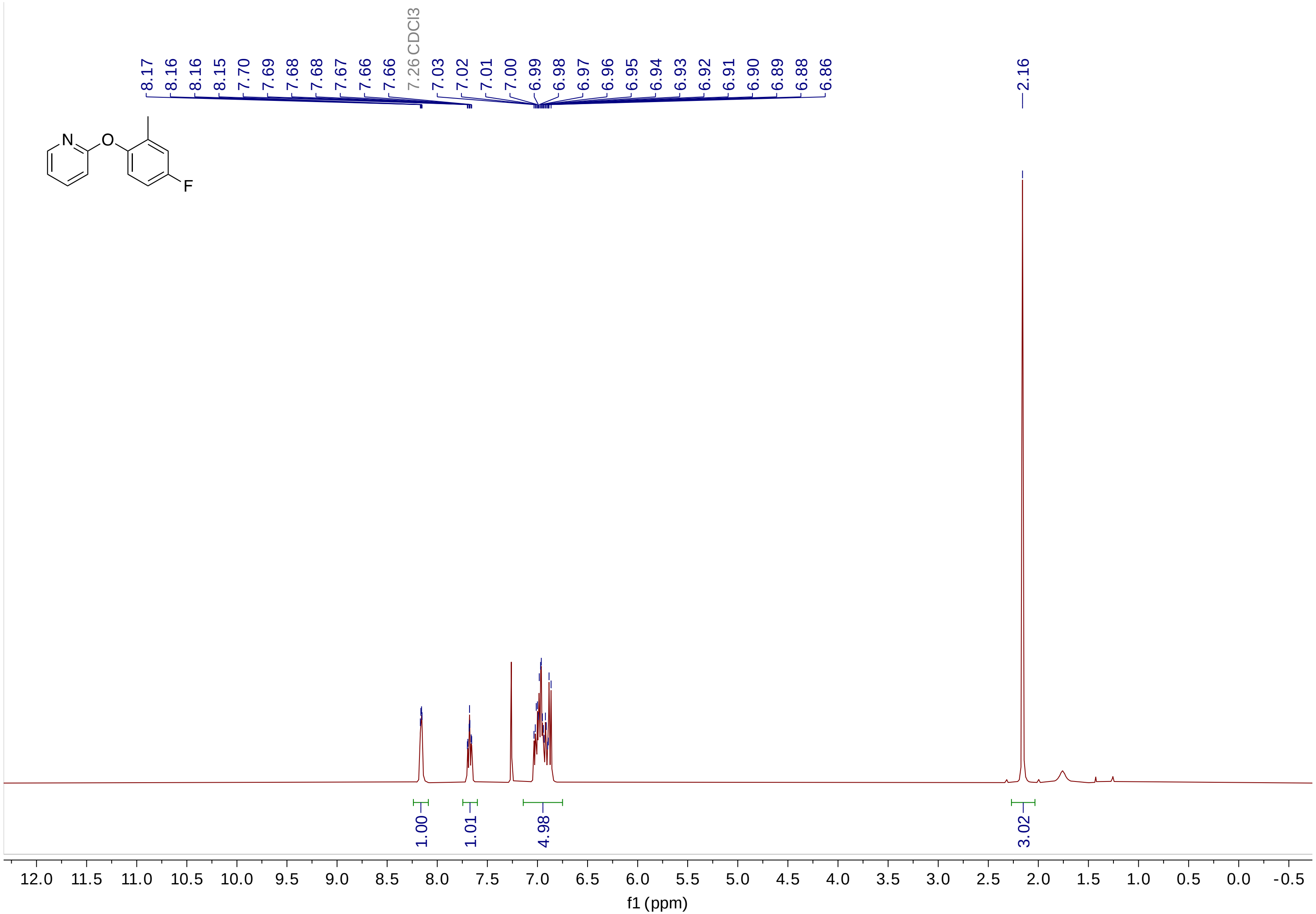

4 - $^{13}C\{^{1}H\}$ NMR (126 MHz, $CDCl_3$):

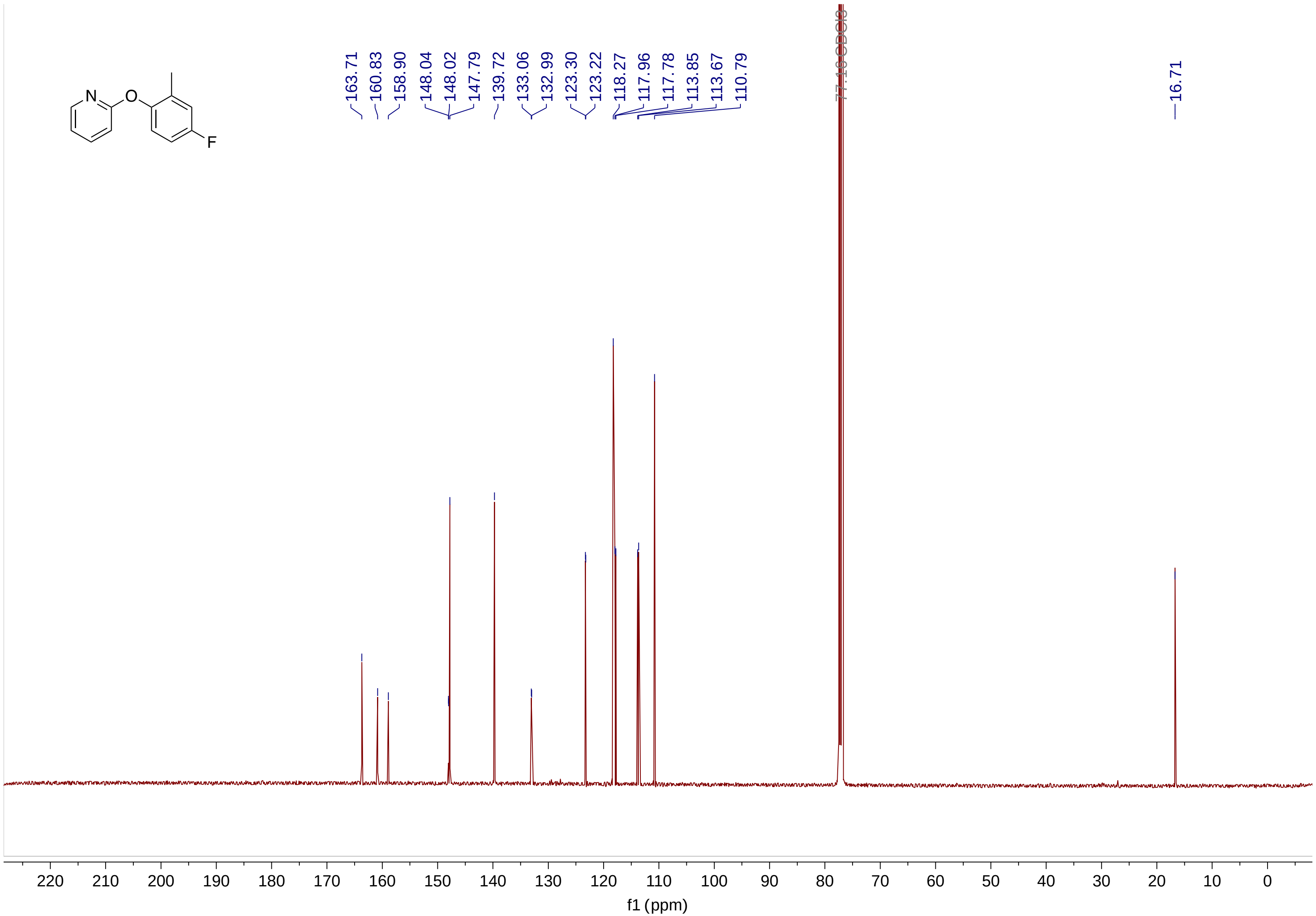

4 - ^{19}F NMR (377 MHz, $CDCl_3$):

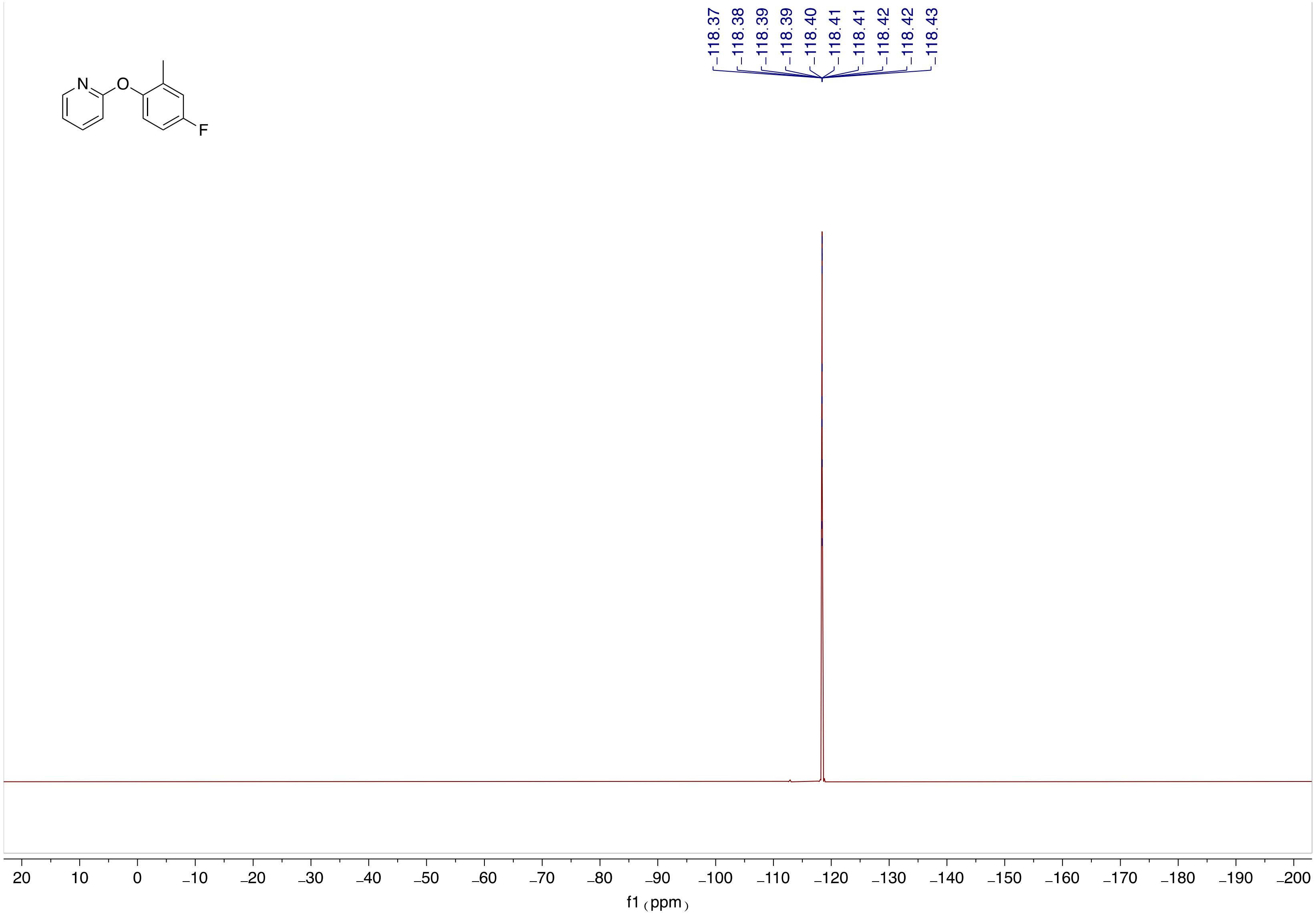

6 - ^{1}H NMR (400 MHz, CDCl$_3$):

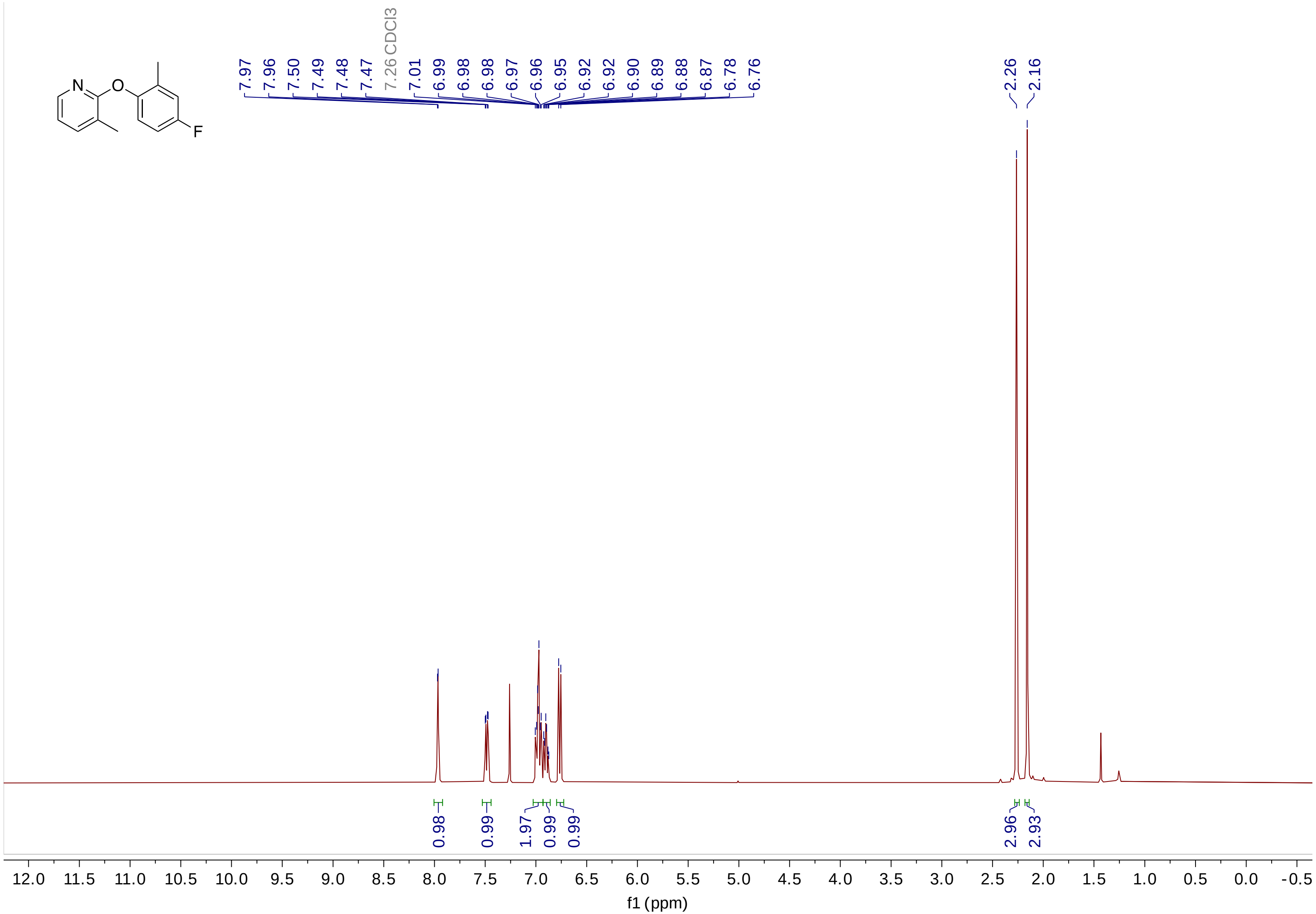

6 - $^{13}C\{^{1}H\}$ NMR (101 MHz, $CDCl_3$):

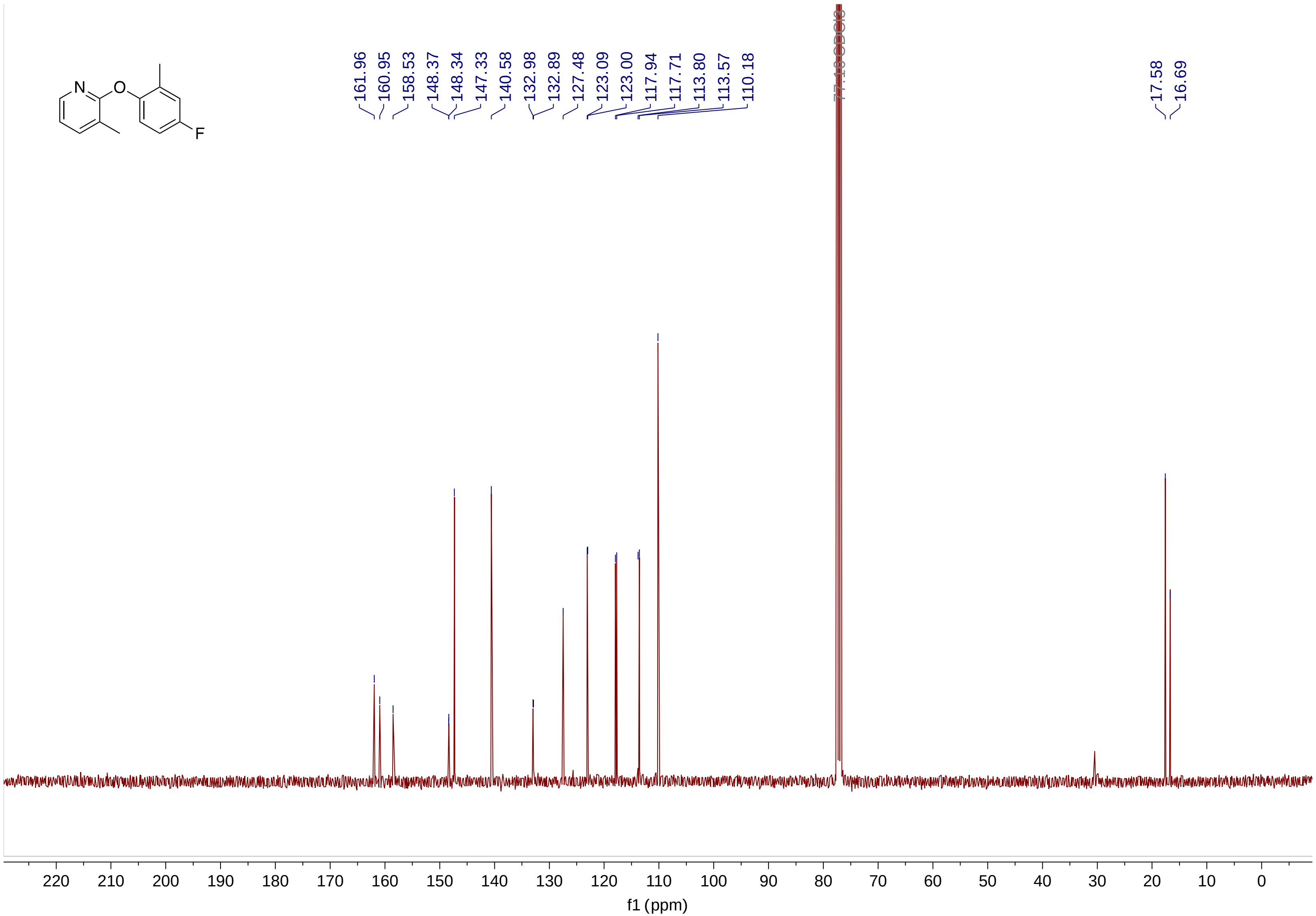

6 - ^{19}F NMR (376 MHz, $CDCl_3$):

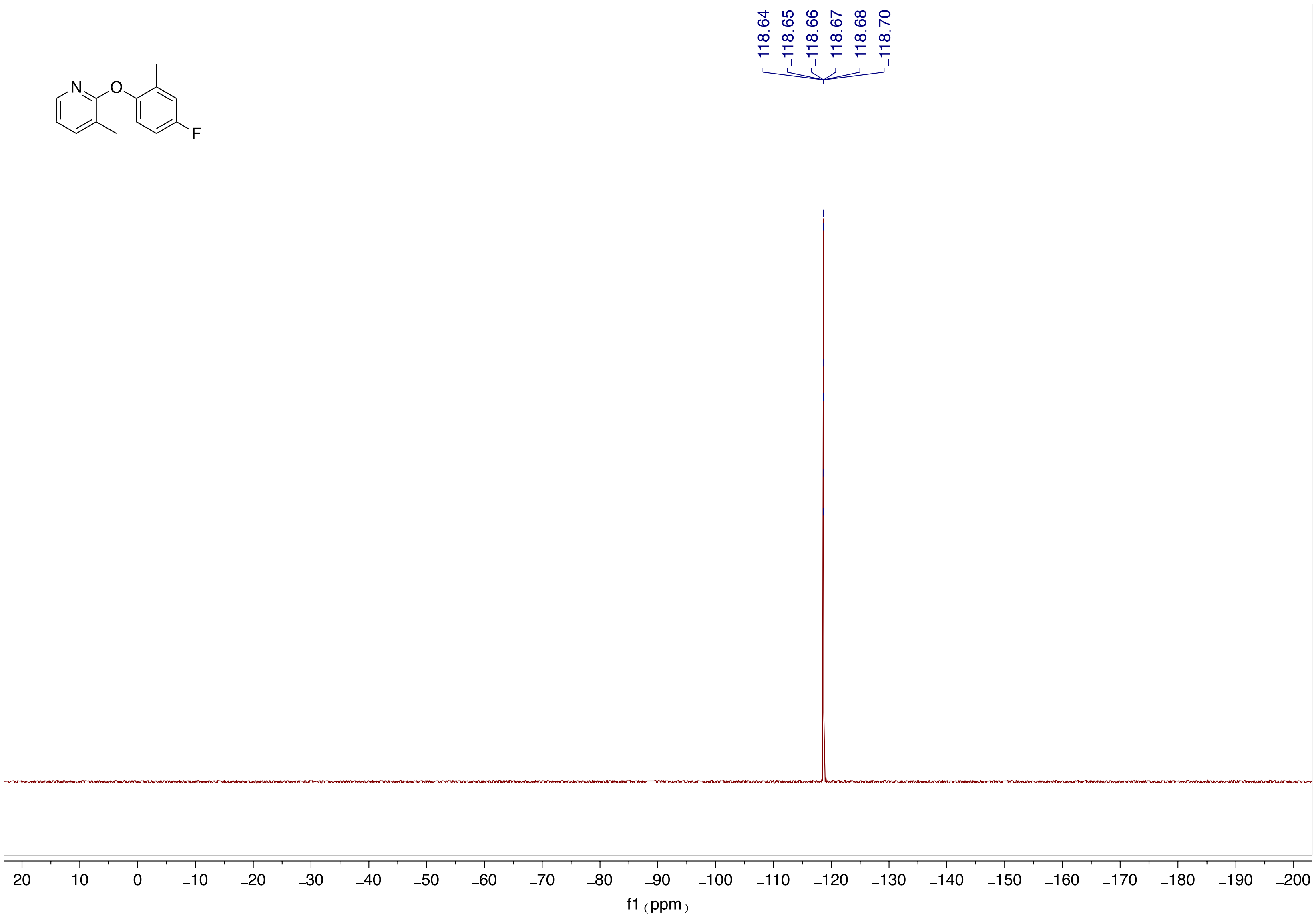

7 - ^{1}H NMR (400 MHz, $CDCl_3$):

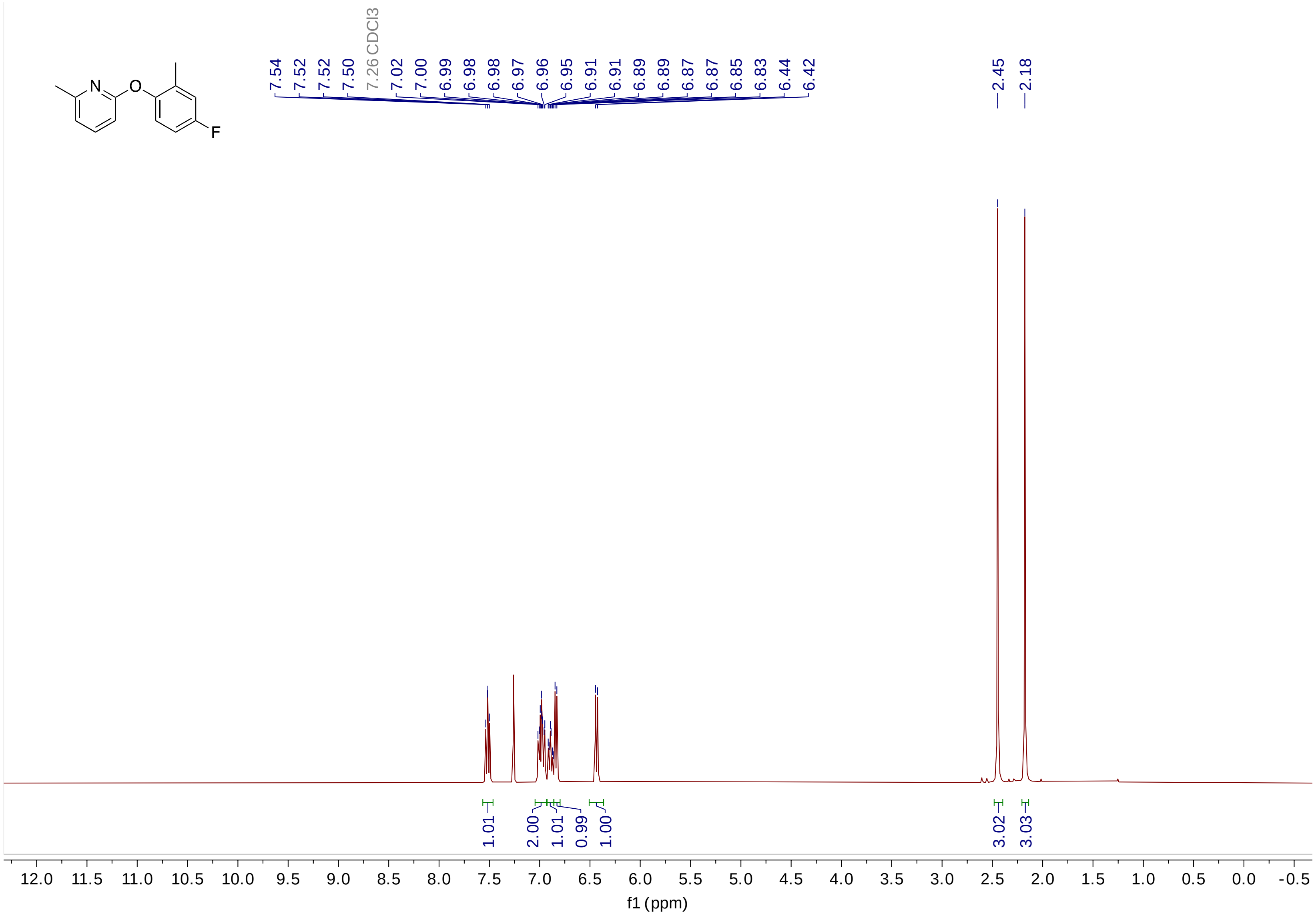

7 - $^{13}C\{^{1}H\}$ NMR (101 MHz, $CDCl_3$):

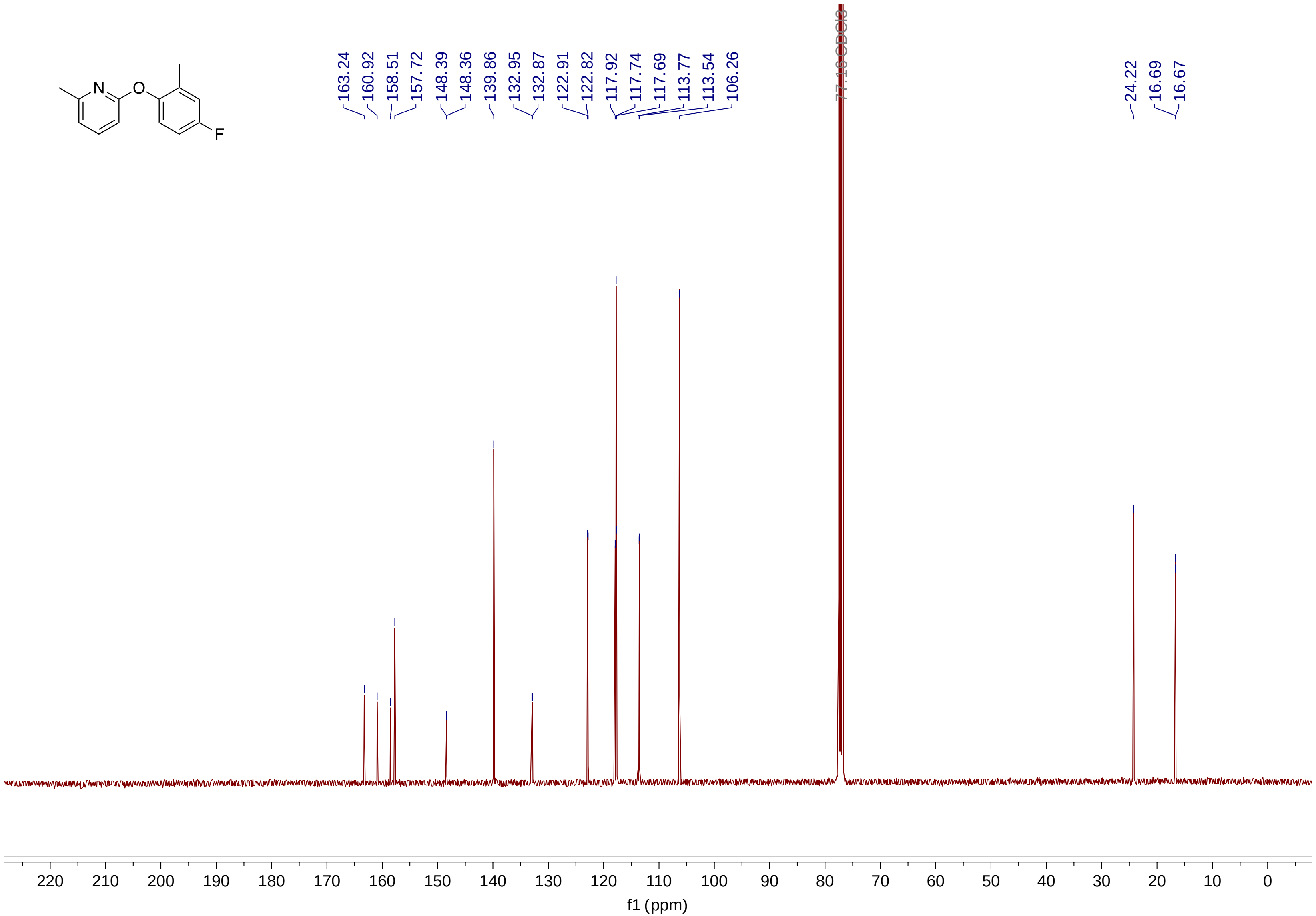

7 - ^{19}F NMR (377 MHz, $CDCl_3$):

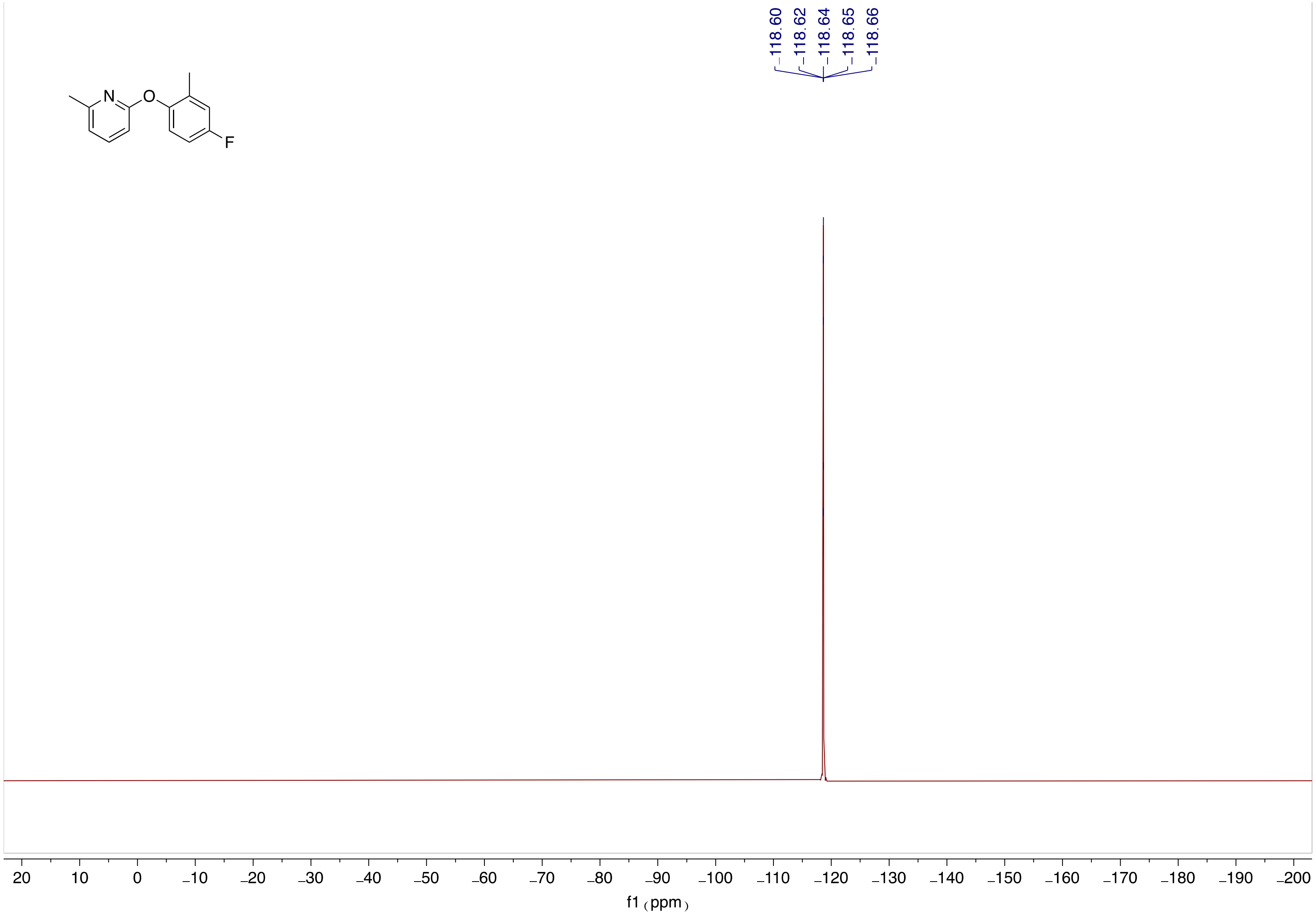

8 - ^{1}H NMR (400 MHz, $CDCl_3$):

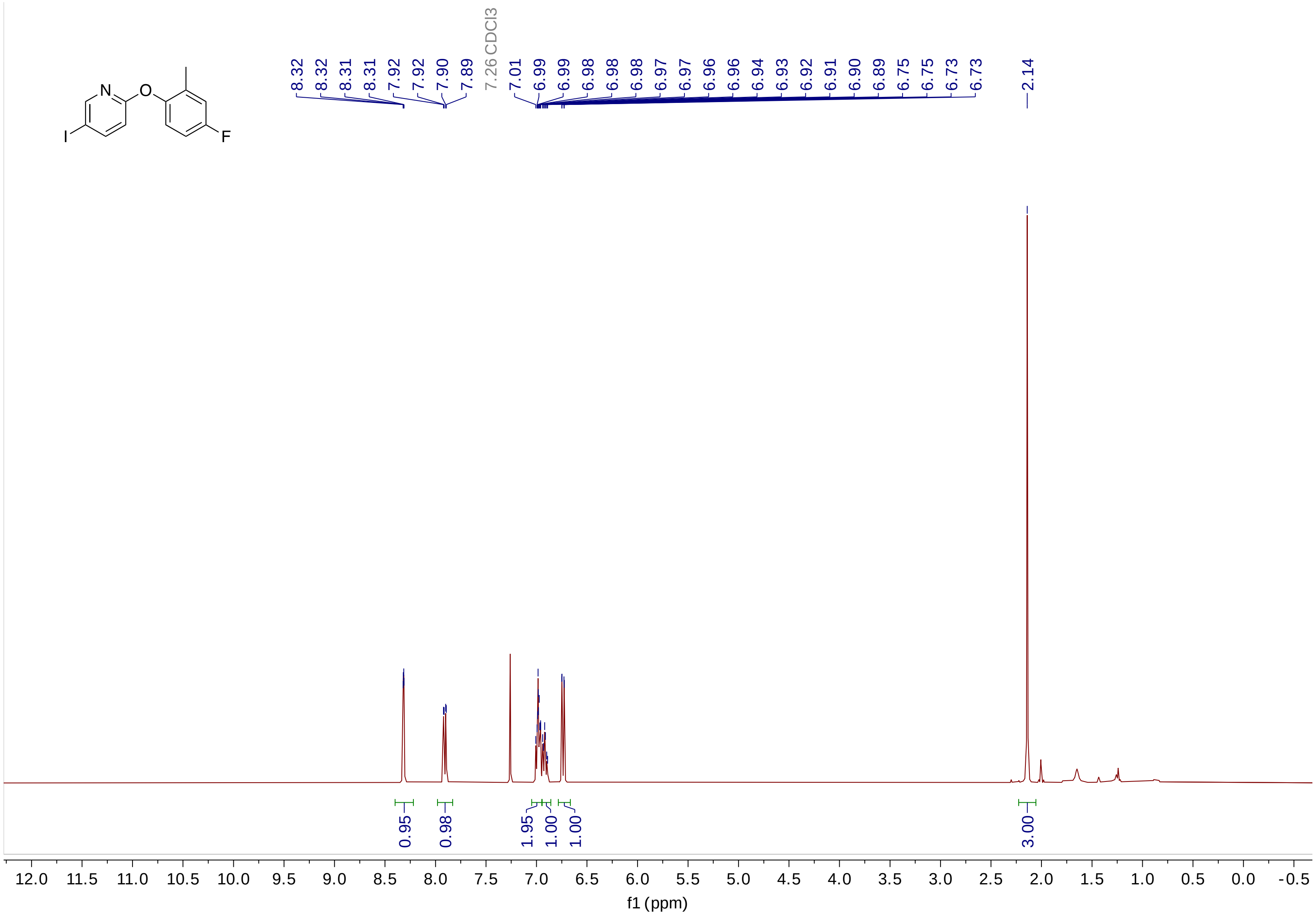

8 - $^{13}C\{^{1}H\}$ NMR (101 MHz, CDCl$_3$):

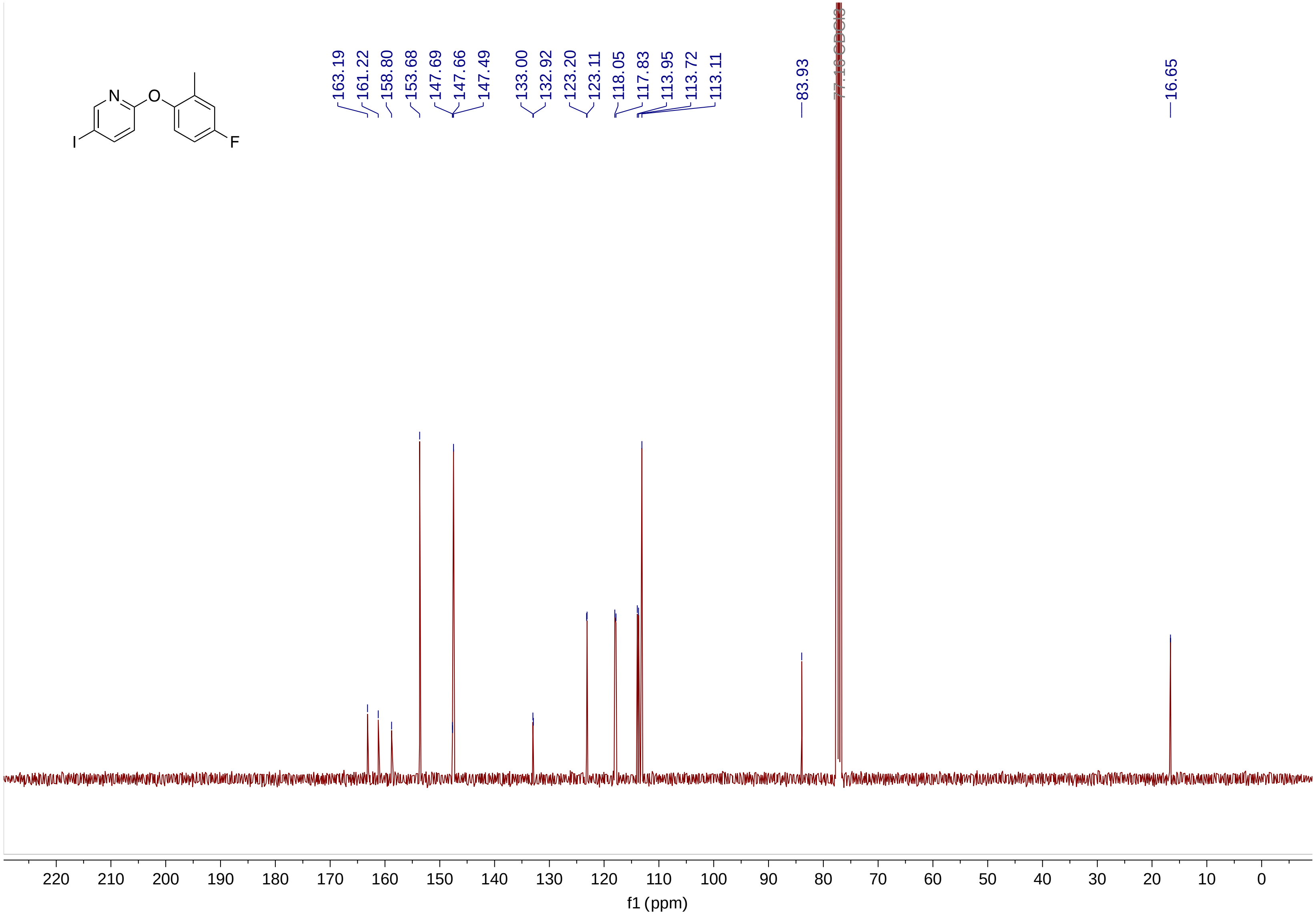

8 - ^{19}F NMR (376 MHz, $CDCl_3$):

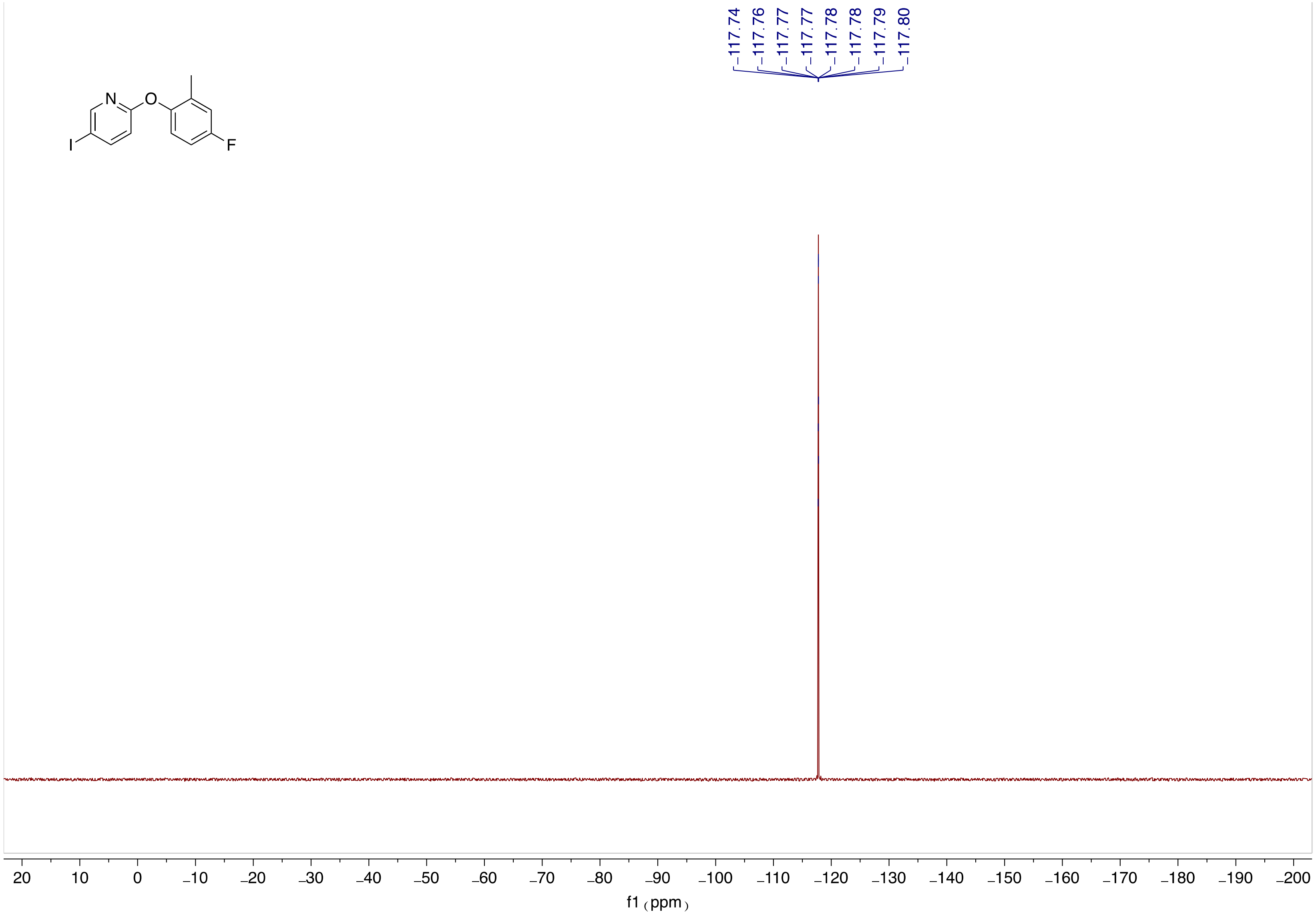

9 - ^{1}H NMR (400 MHz, $CDCl_3$):

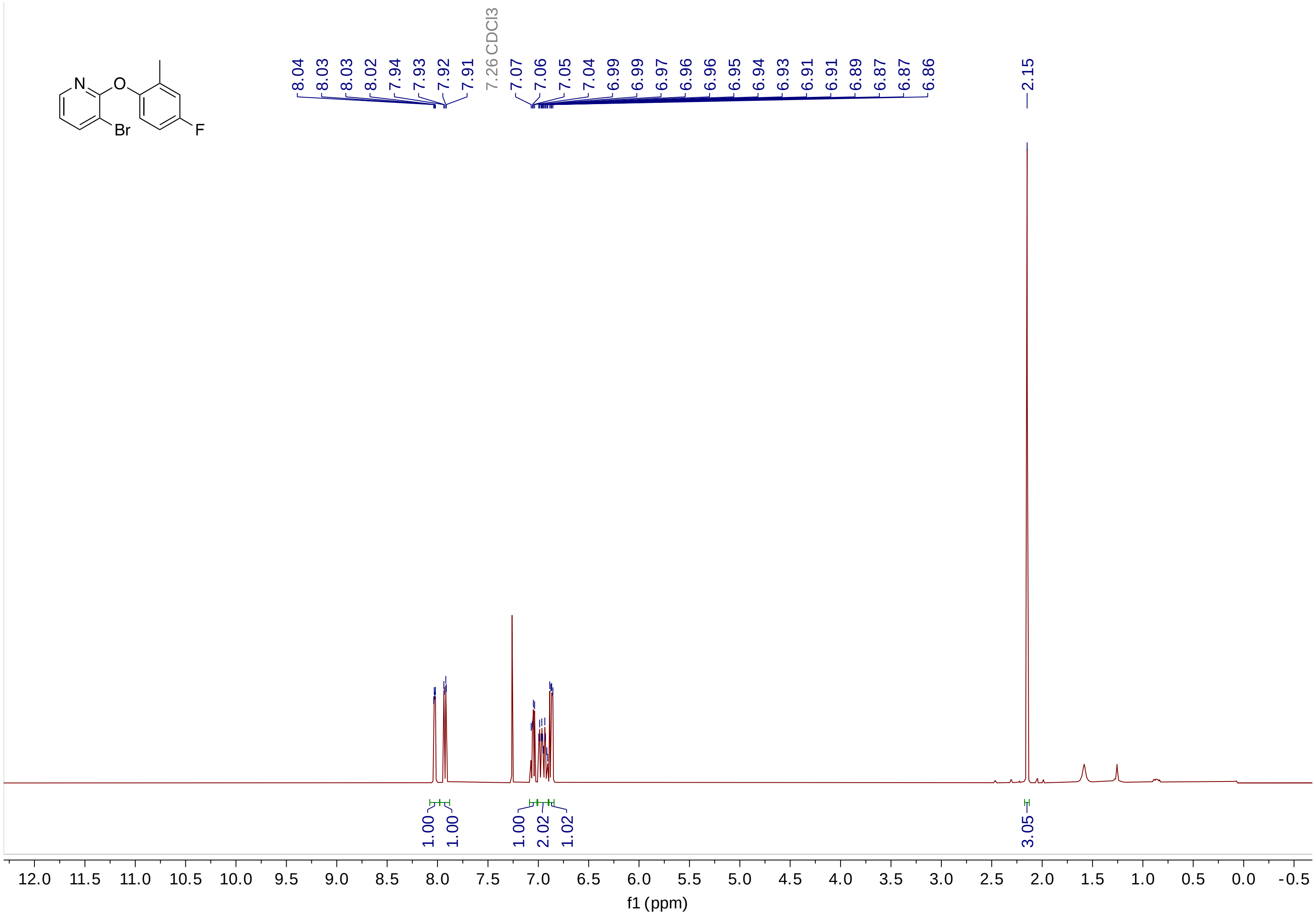

9 - $^{13}C\{^{1}H\}$ NMR (101 MHz, $CDCl_3$):

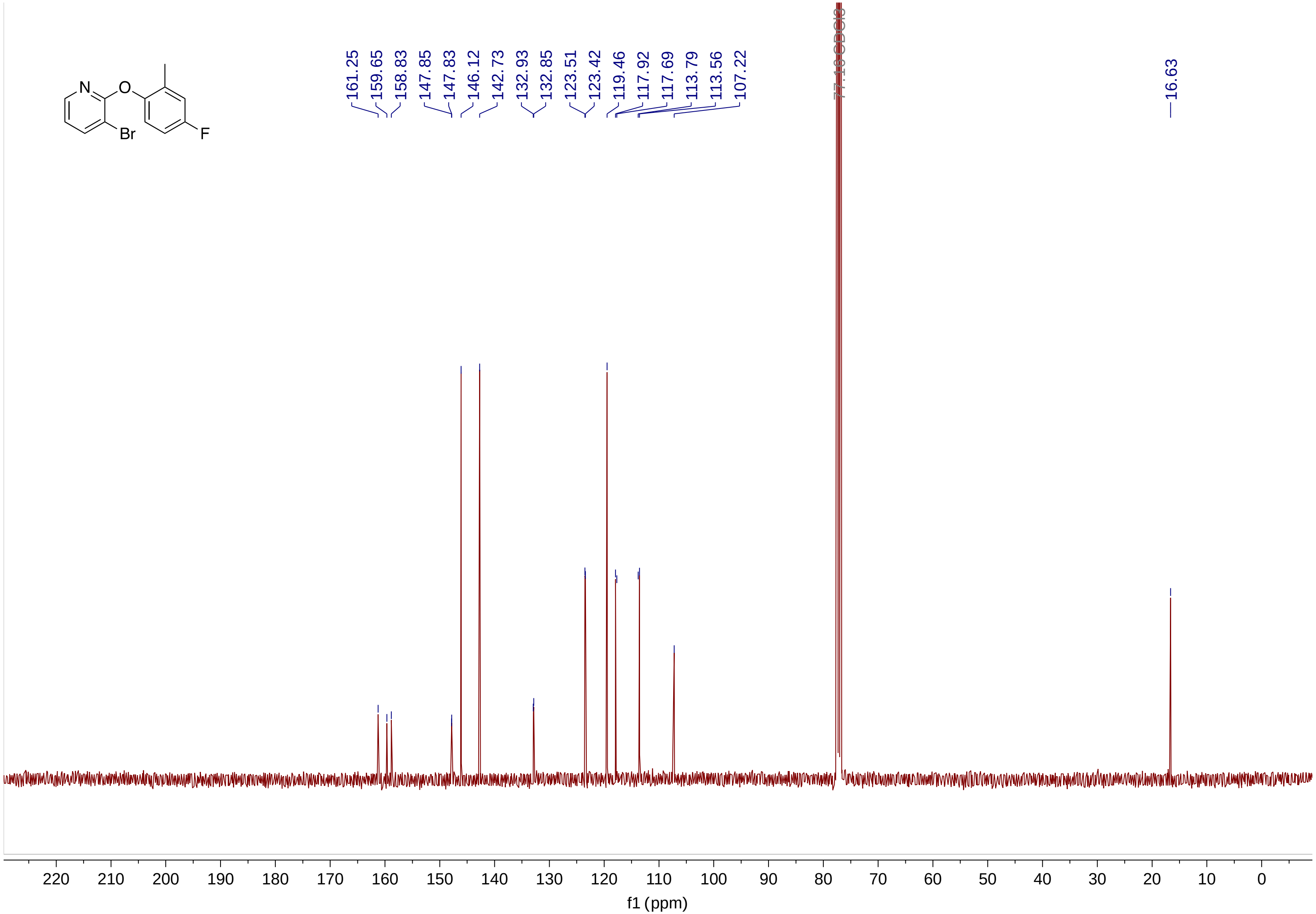

9 - ^{19}F NMR (376 MHz, $CDCl_3$):

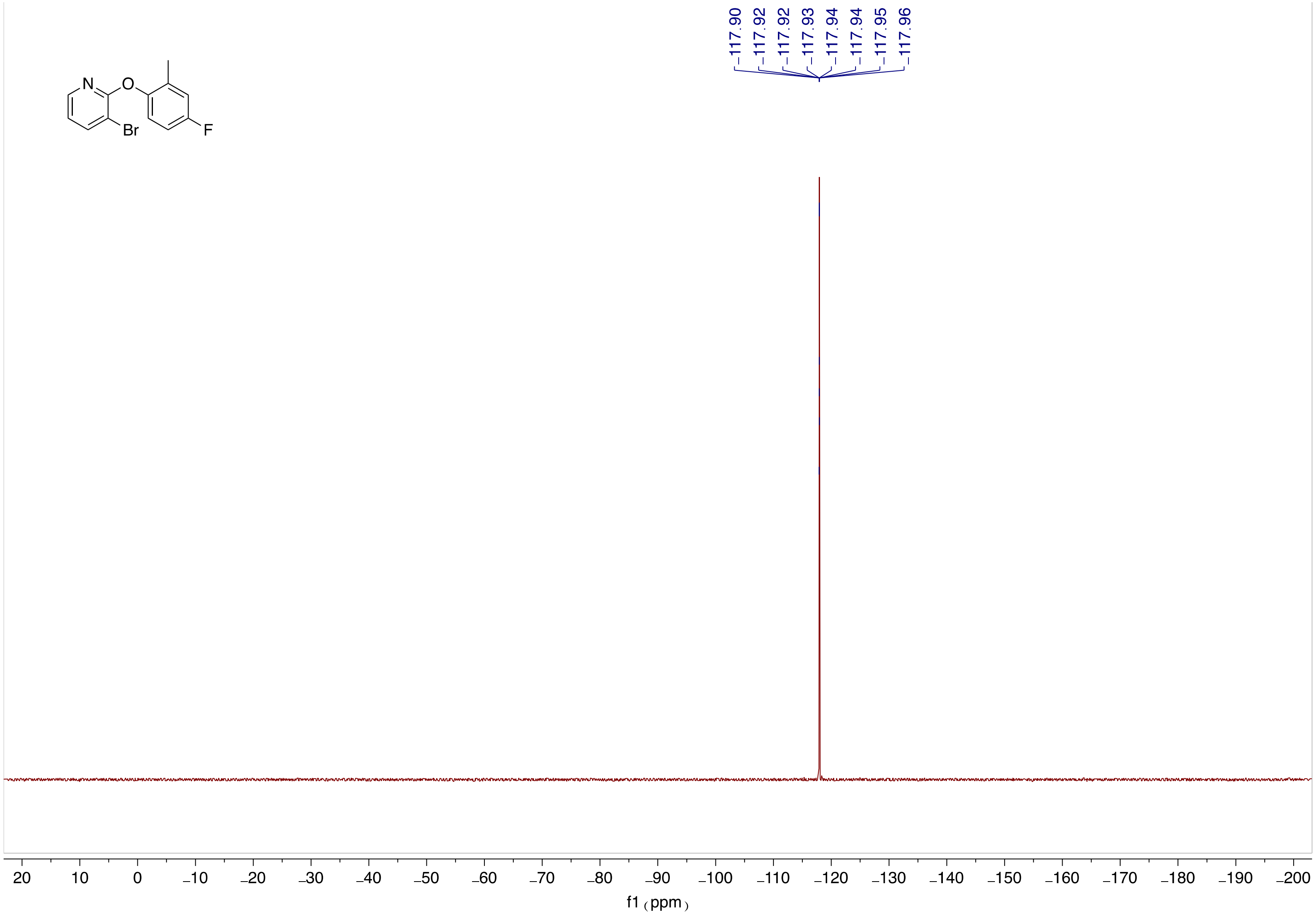

10 - ^{1}H NMR (400 MHz, $CDCl_3$):

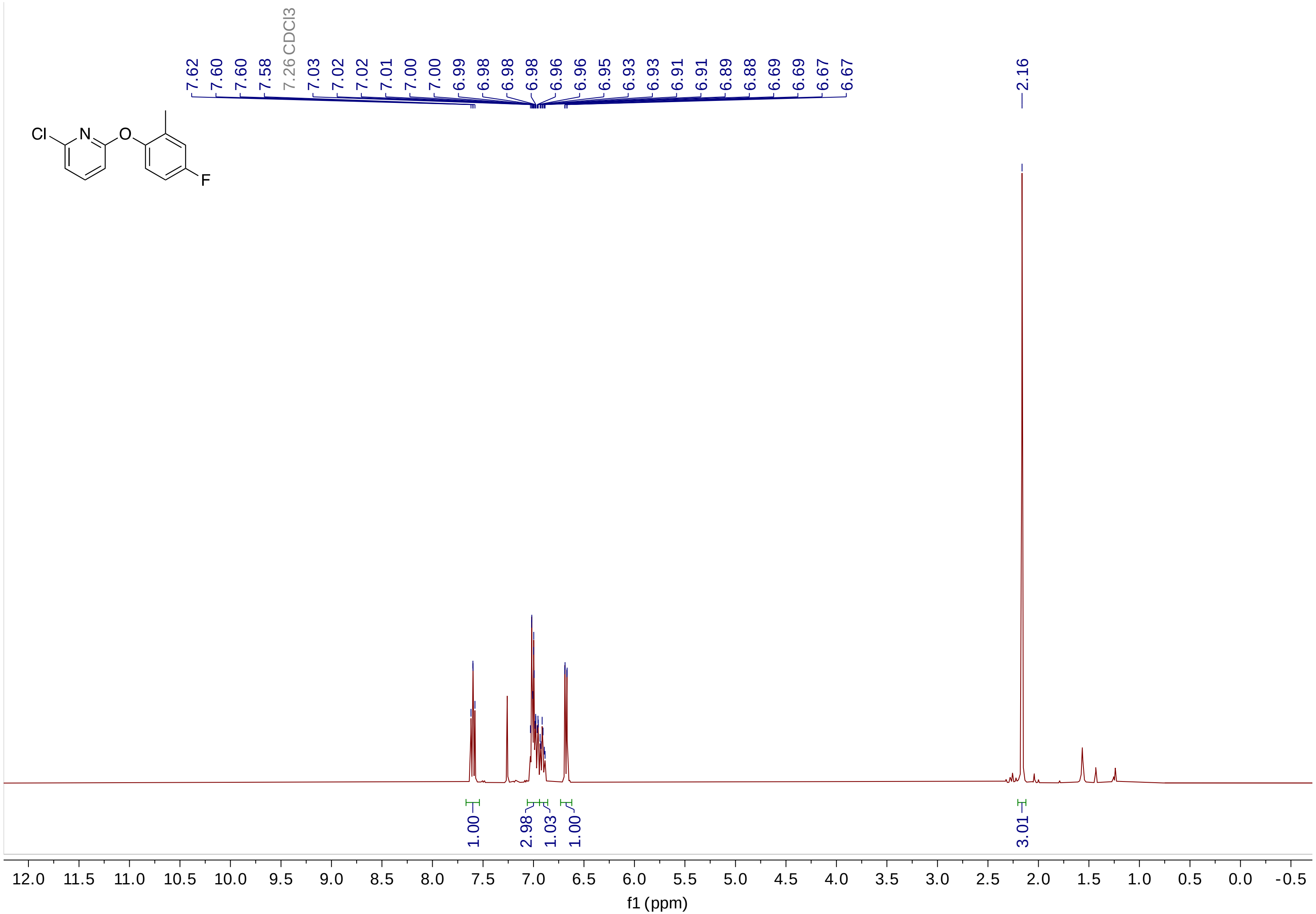

10 - $^{13}C\{^{1}H\}$ NMR (101 MHz, $CDCl_3$):

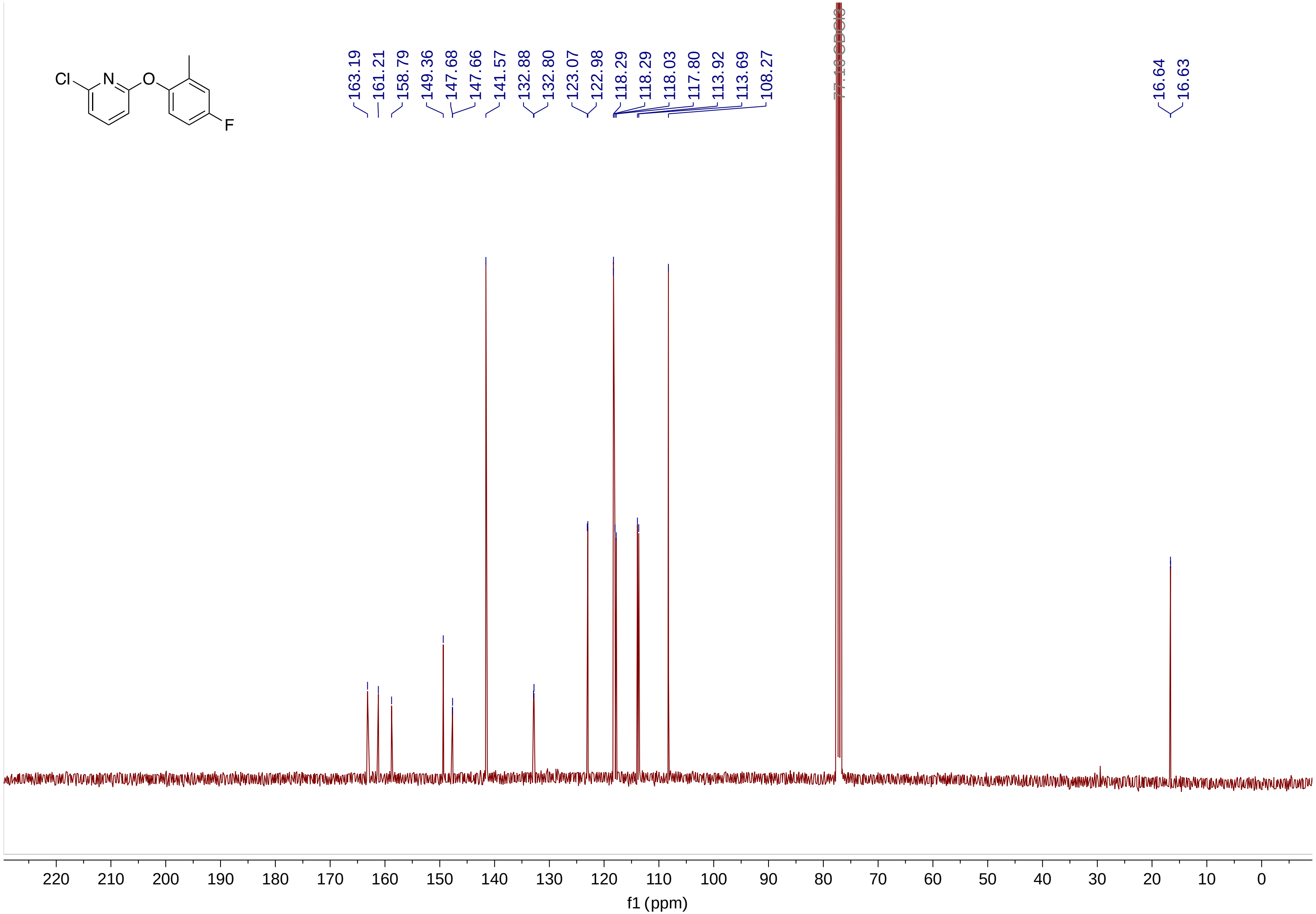

10 - ^{19}F NMR (376 MHz, CDCl$_3$):

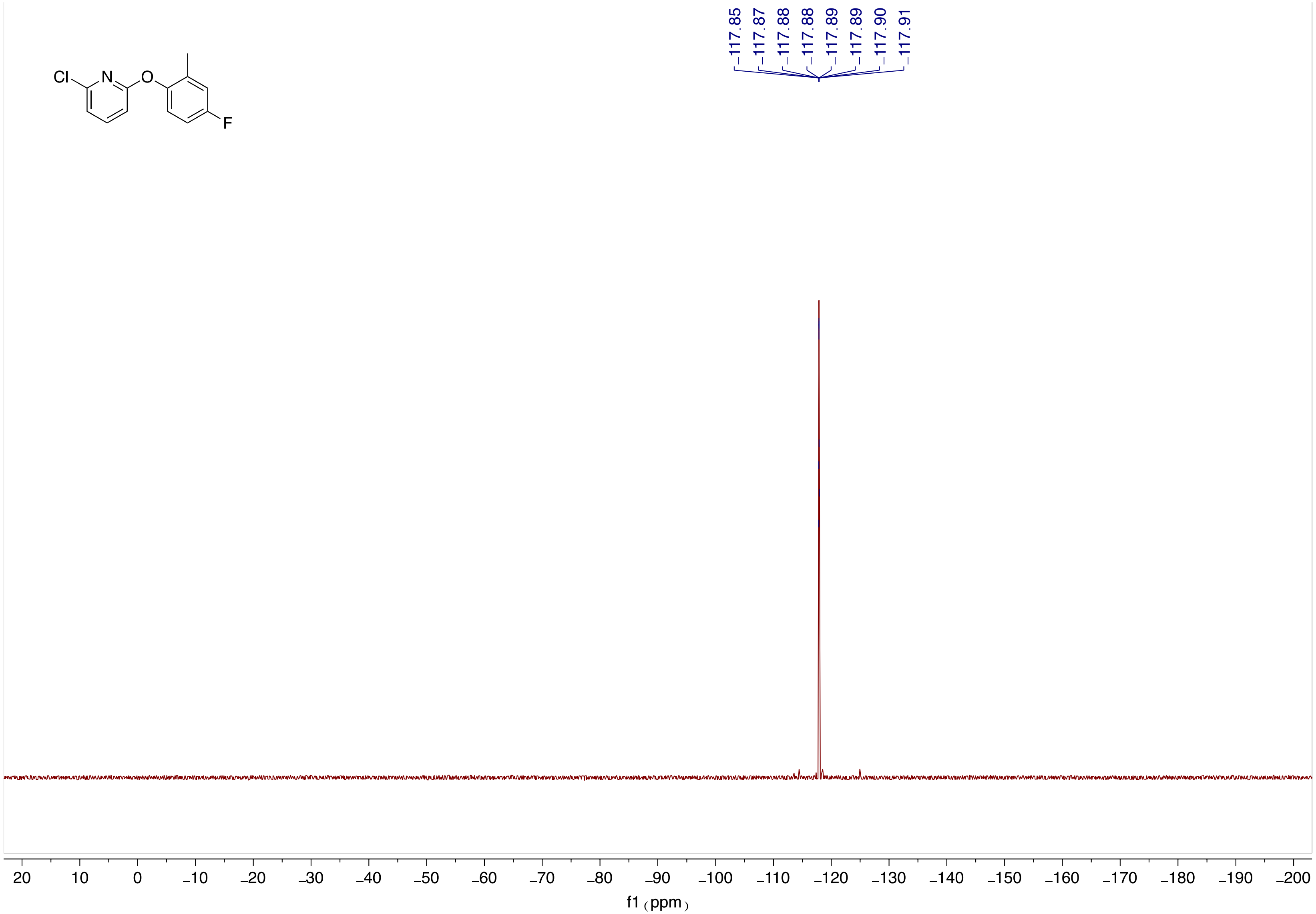

11 - ^{1}H NMR (400 MHz, $CDCl_3$):

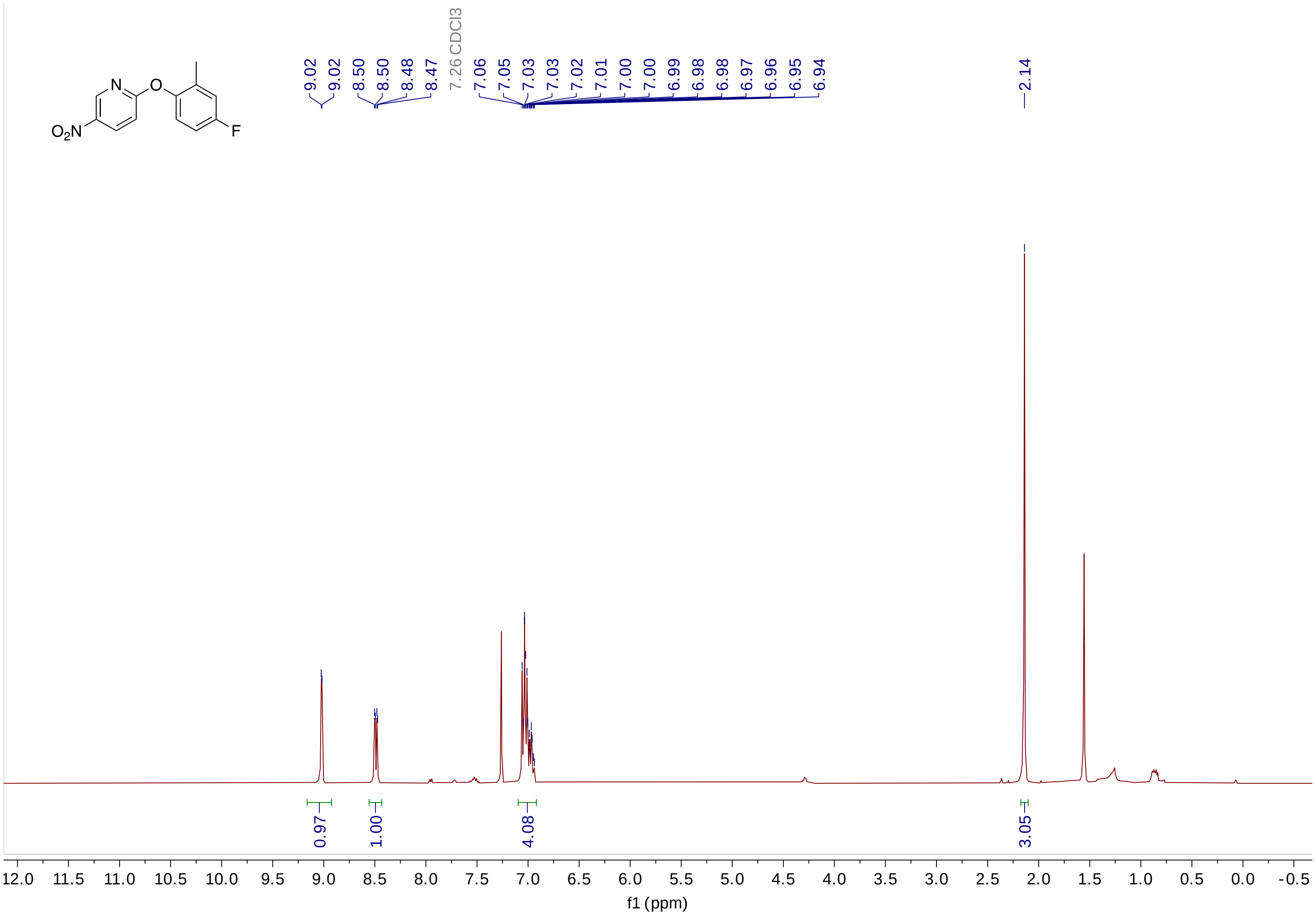

11 - $^{13}C\{^{1}H\}$ NMR (101 MHz, $CDCl_3$):

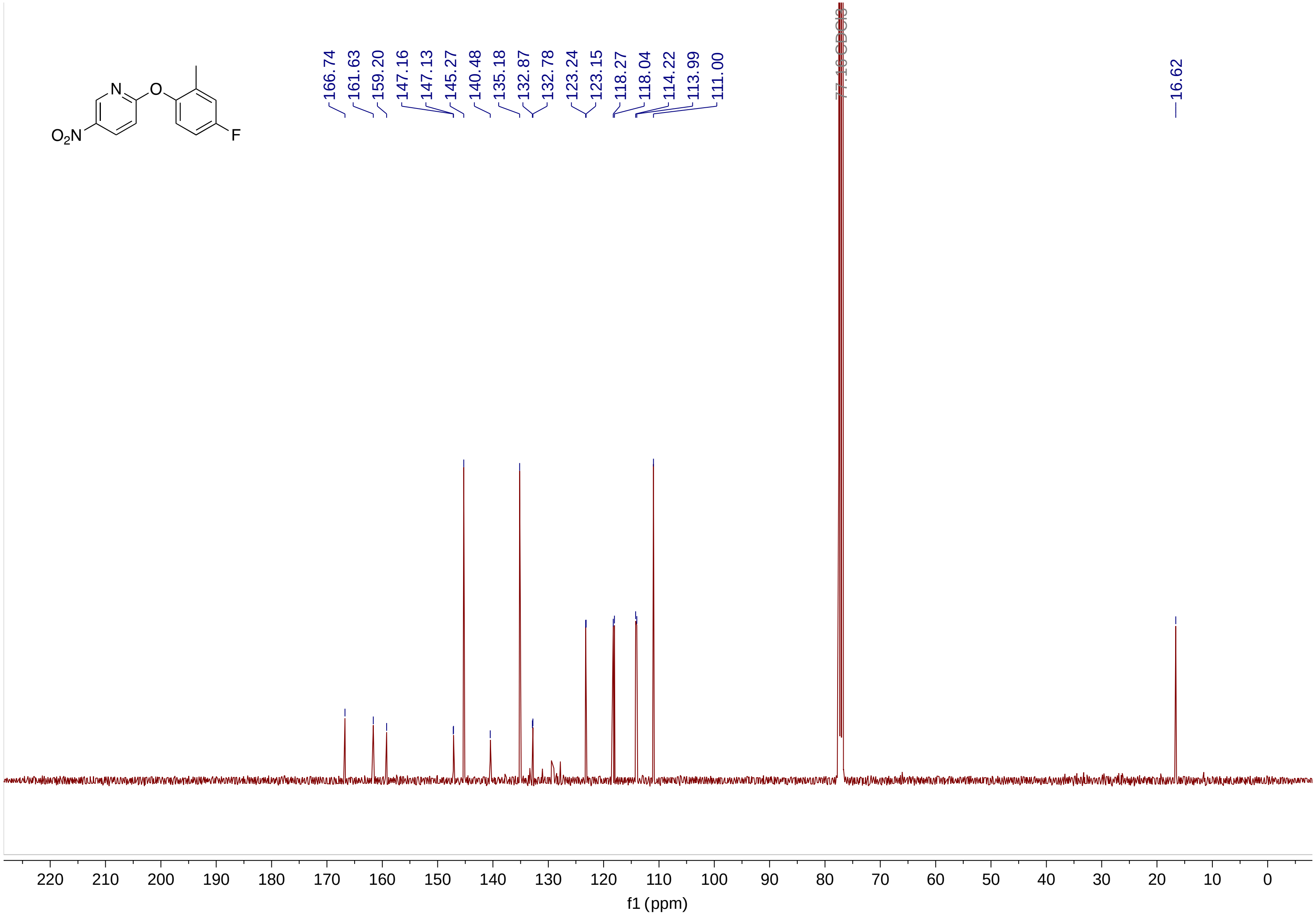

11 - ^{19}F NMR (377 MHz, $CDCl_3$):

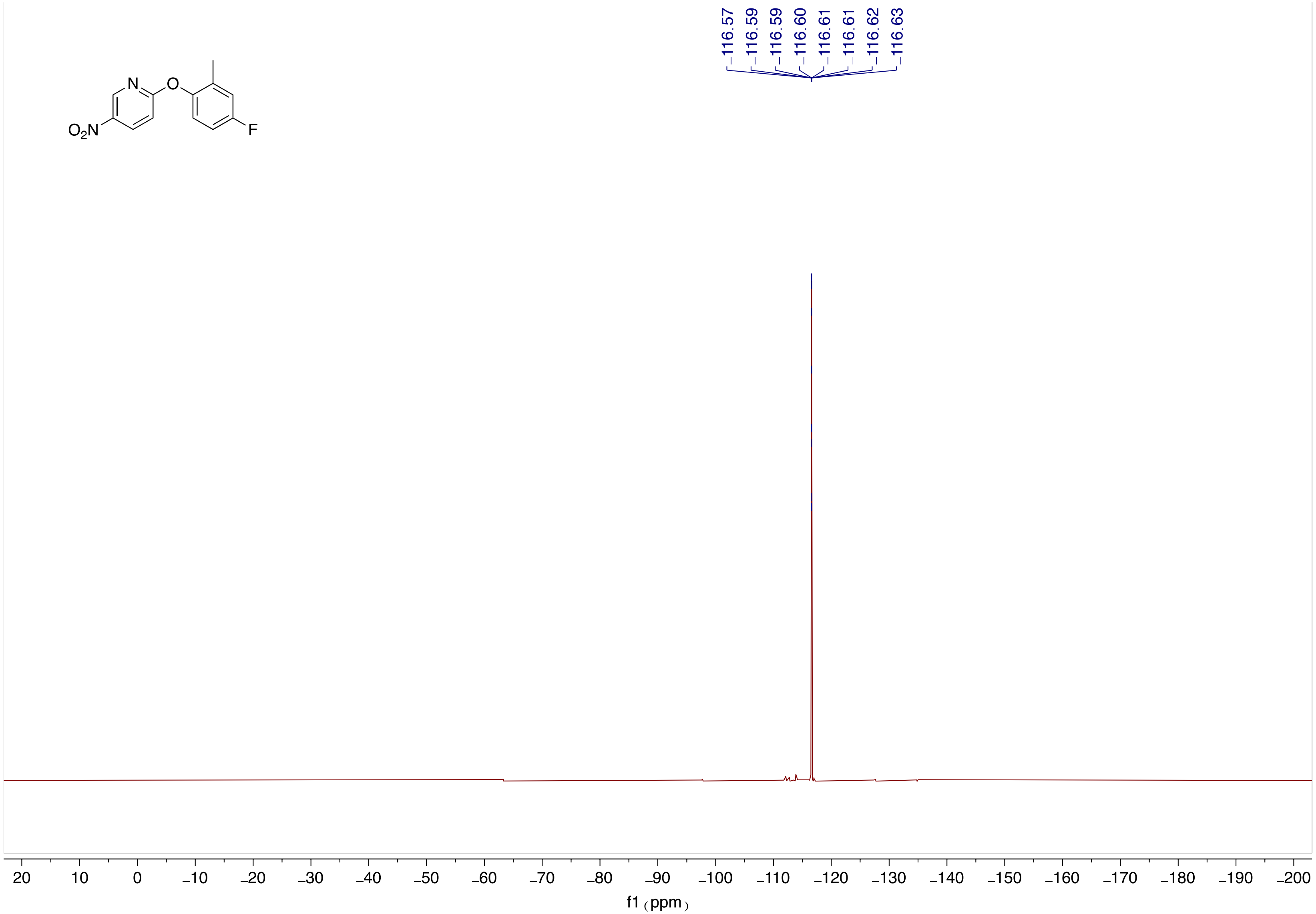

12 - ^{1}H NMR (400 MHz, $CDCl_3$):

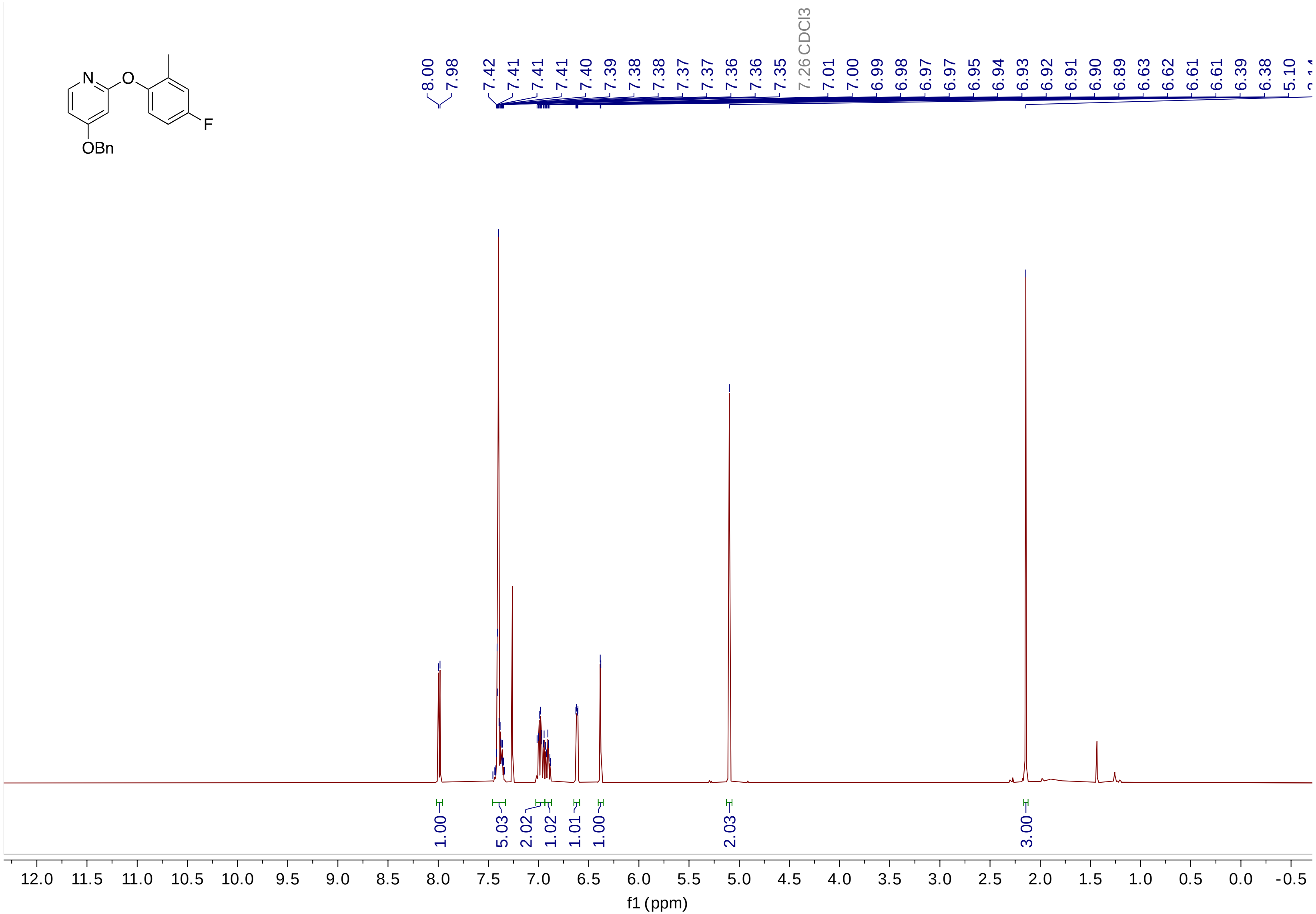

12 - $^{13}C\{^1H\}$ NMR (101 MHz, $CDCl_3$):

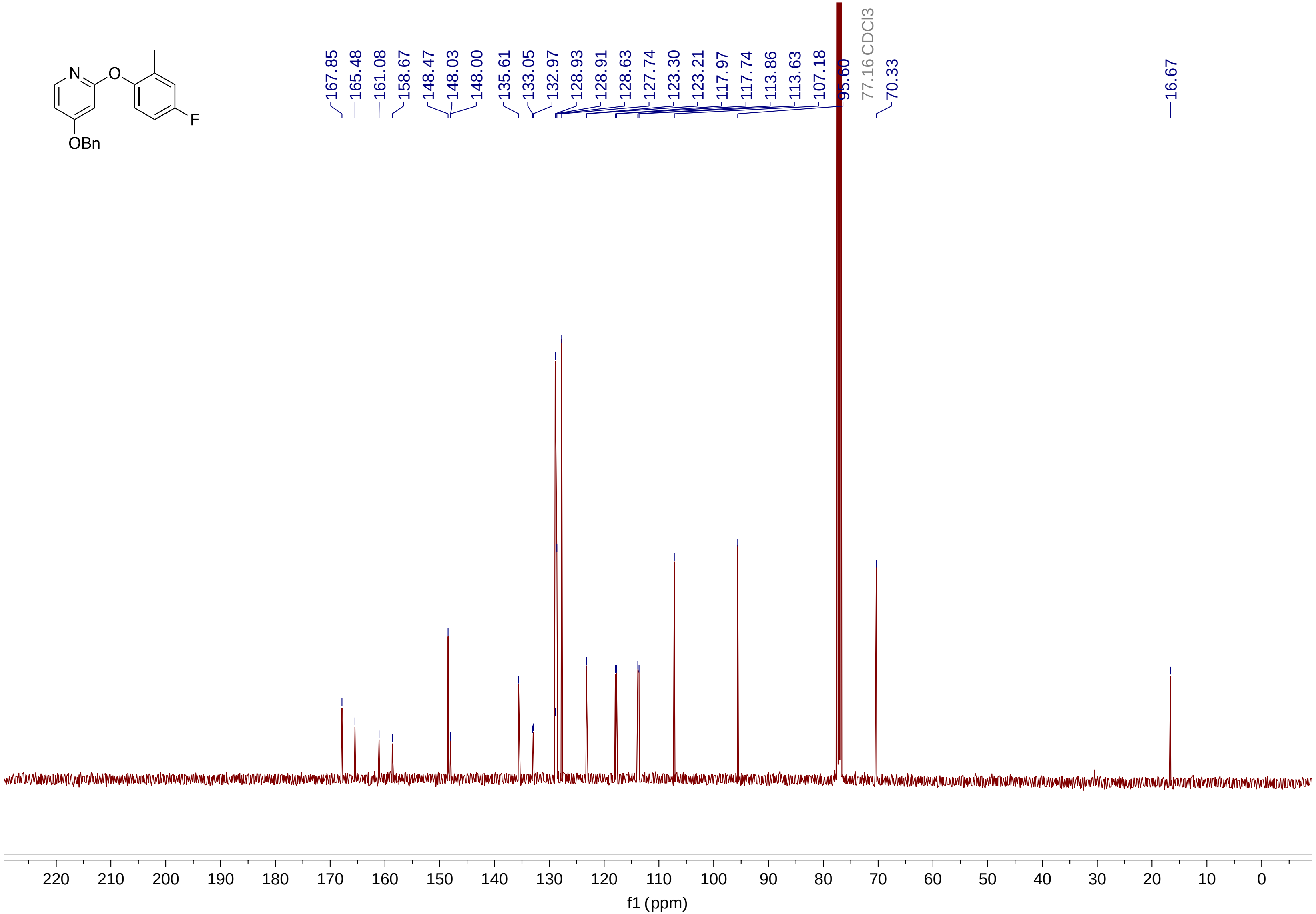

12 - ^{19}F NMR (376 MHz, $CDCl_3$):

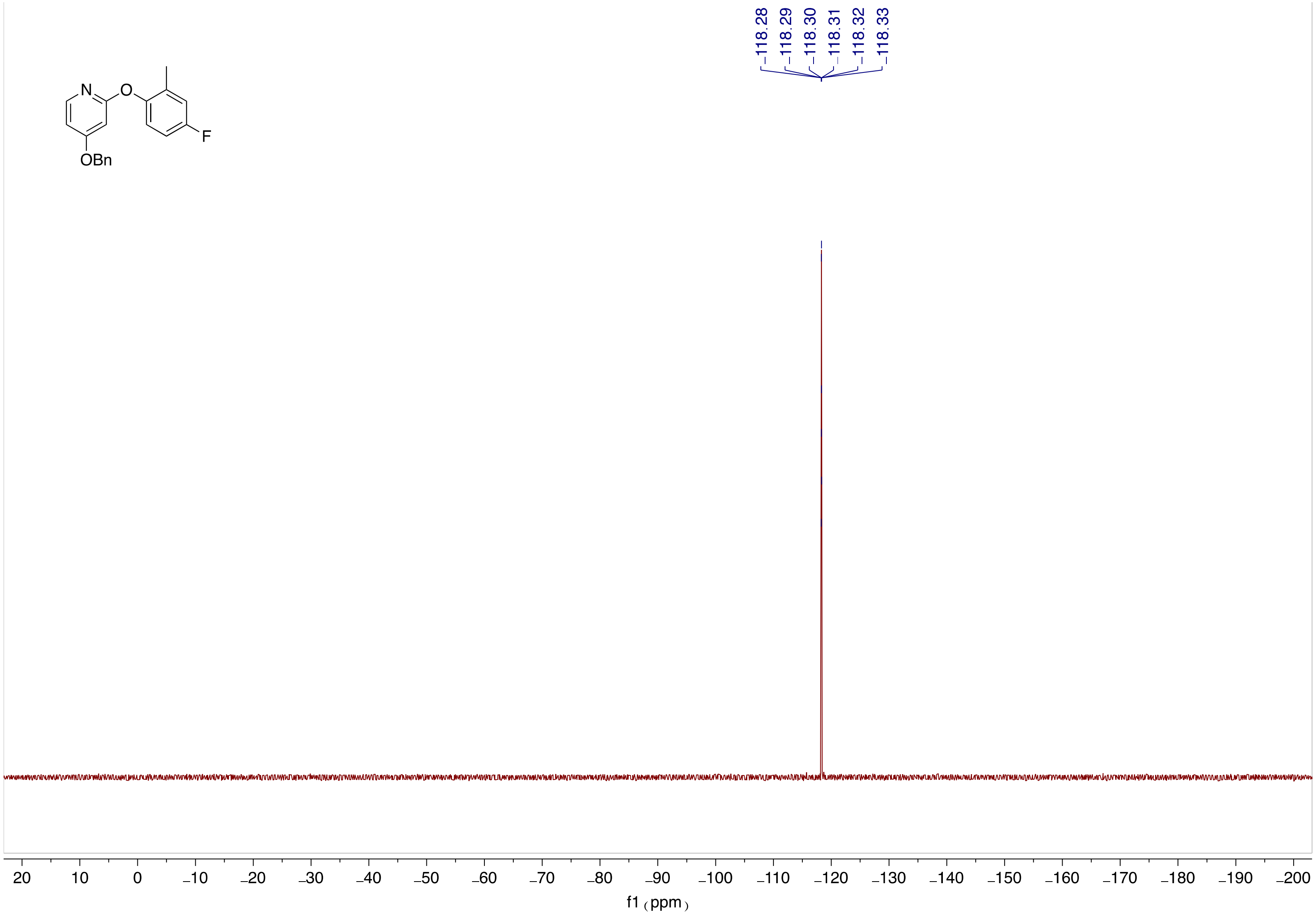

13 - ^{1}H NMR (400 MHz, $CDCl_3$):

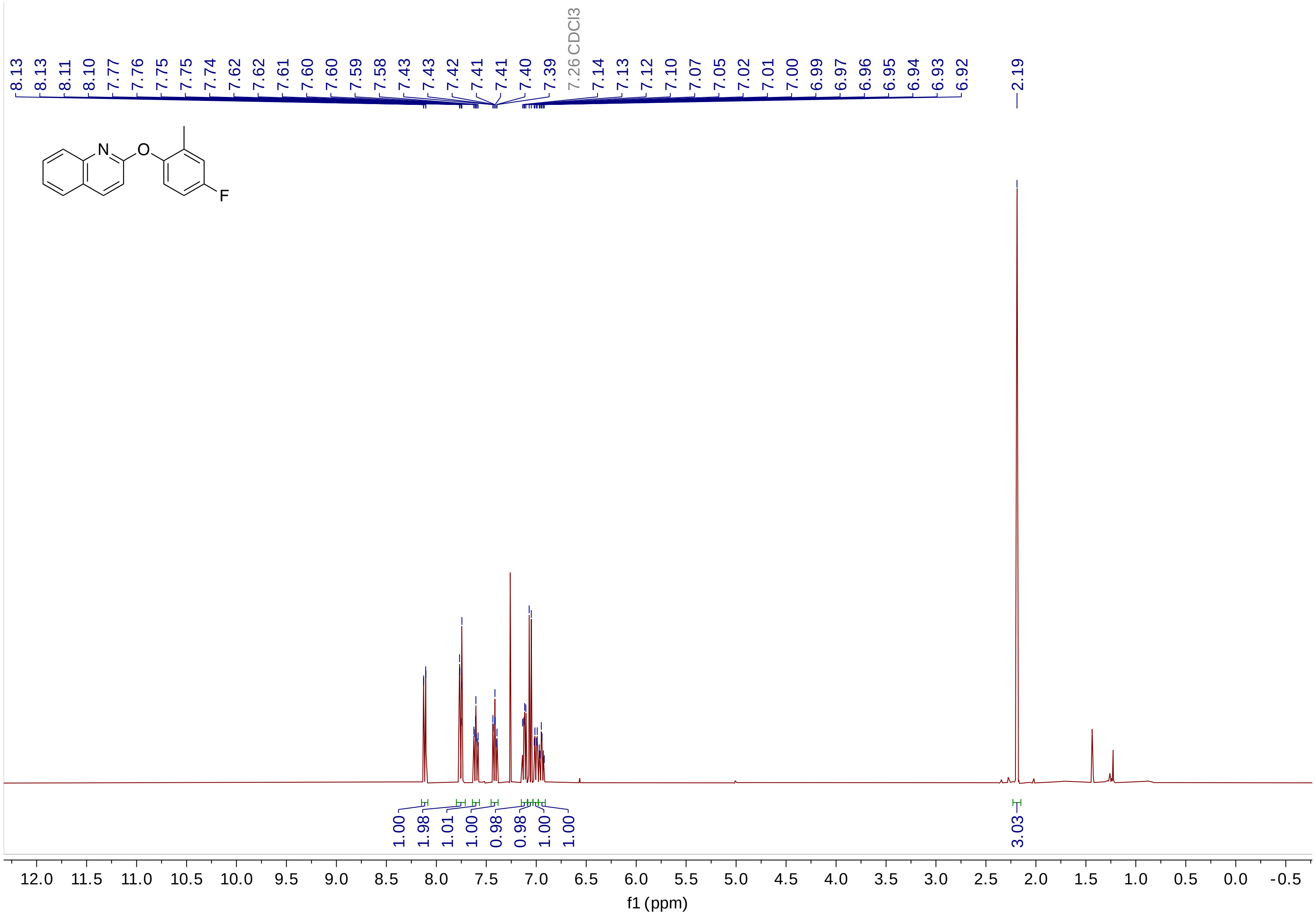

13 - $^{13}C\{^1H\}$ NMR (101 MHz, $CDCl_3$):

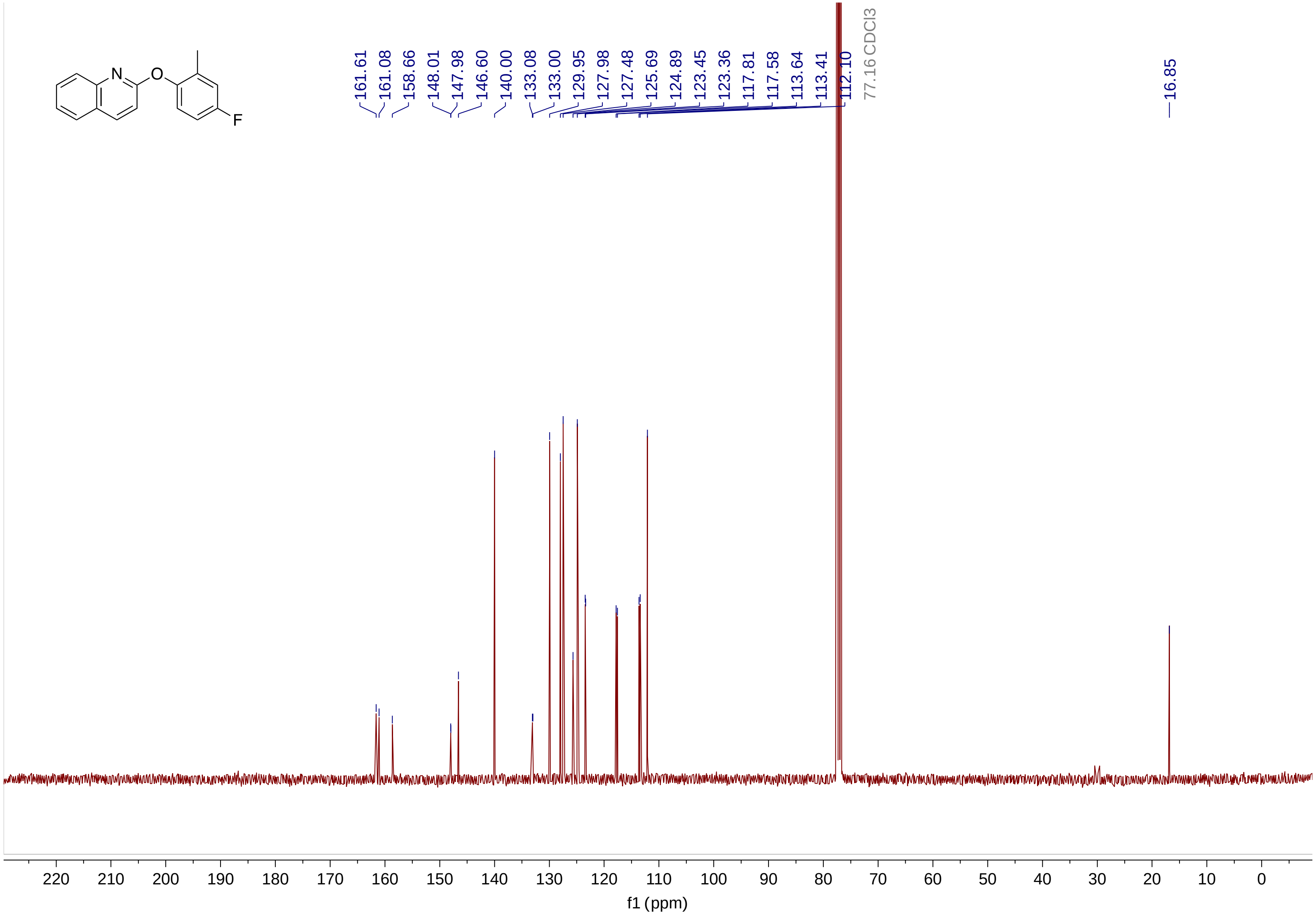

13 - ^{19}F NMR (376 MHz, $CDCl_3$):

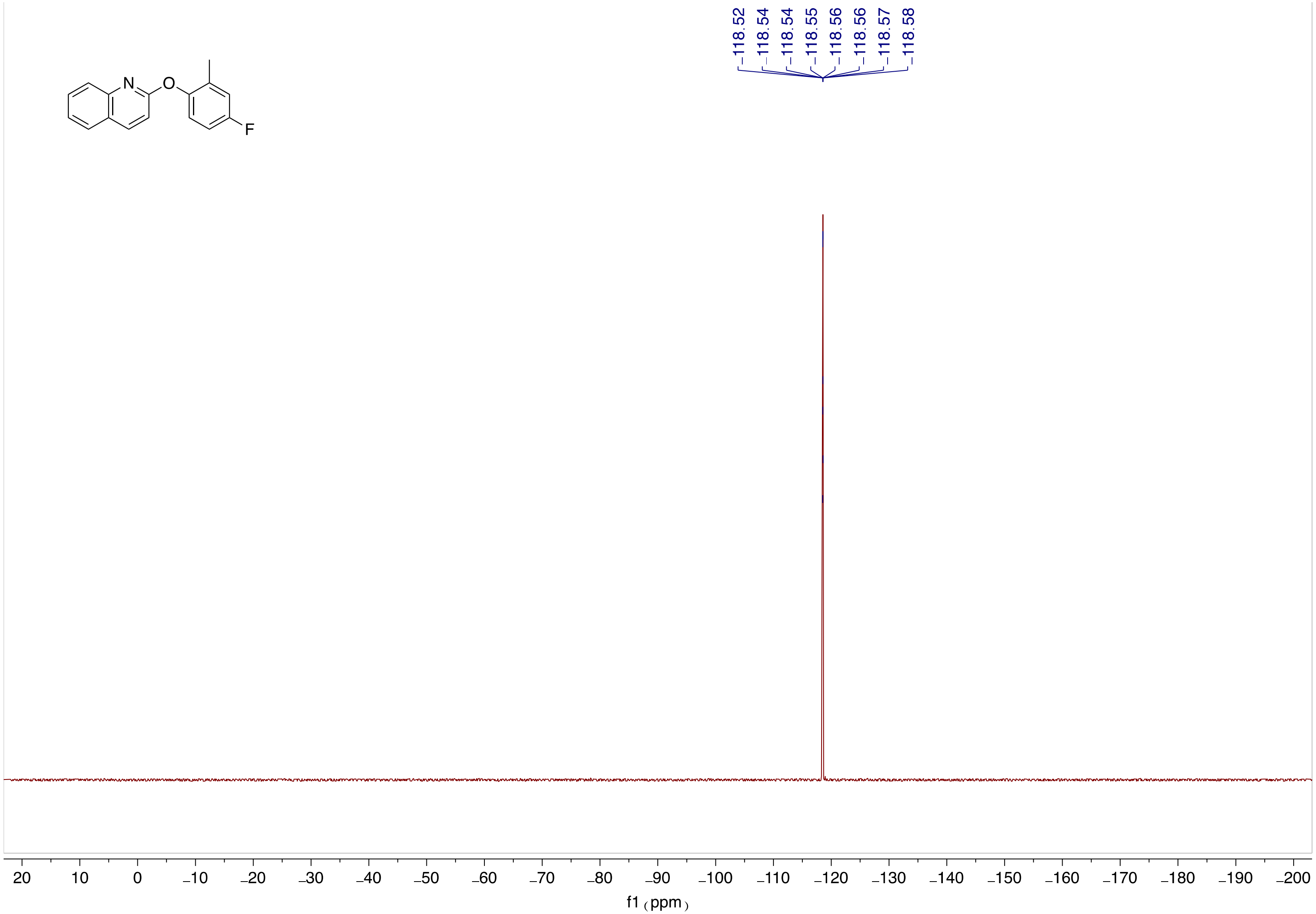

14 - ^{1}H NMR (400 MHz, $CDCl_3$):

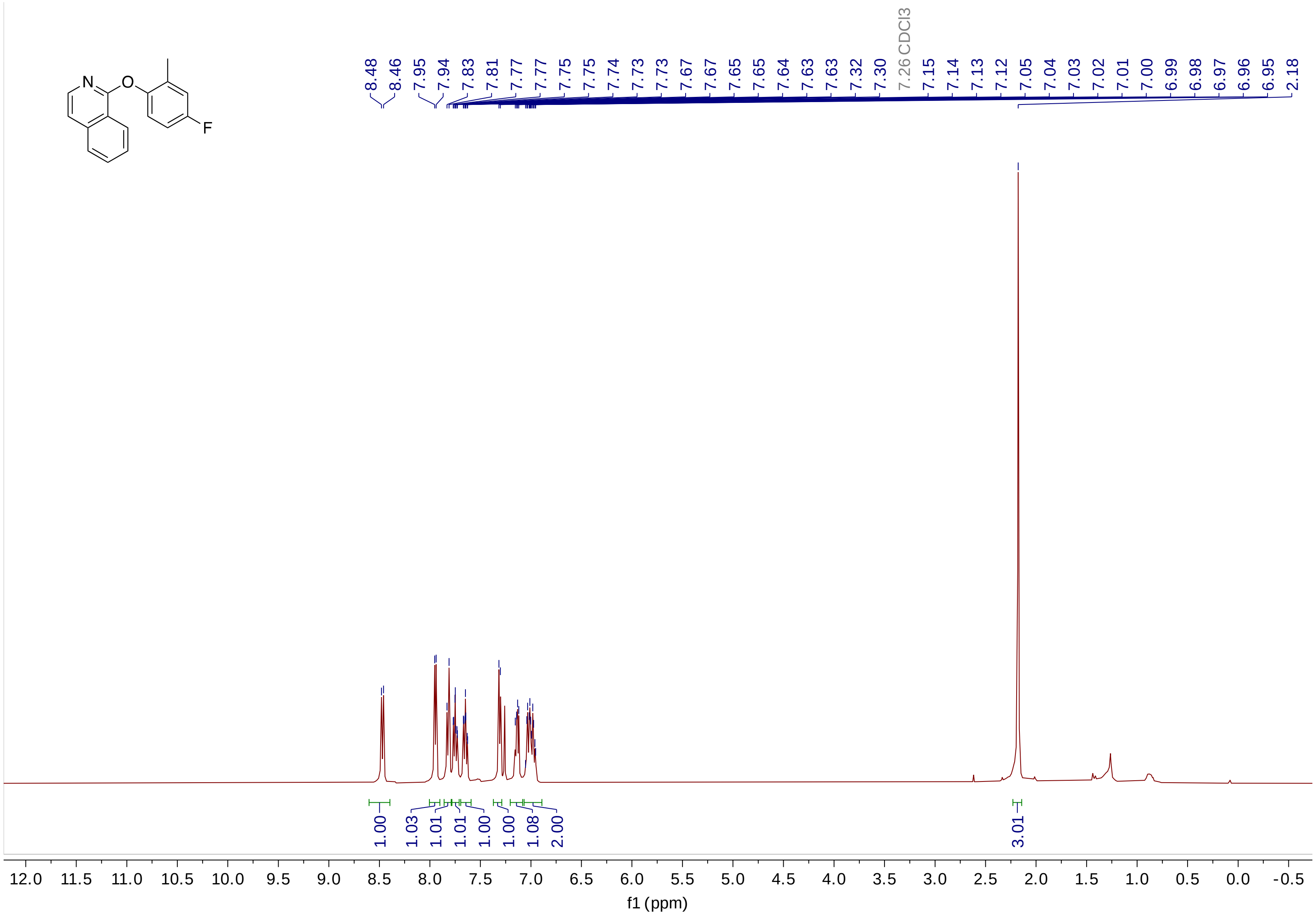

14 - $^{13}C\{^{1}H\}$ NMR (101 MHz, $CDCl_3$):

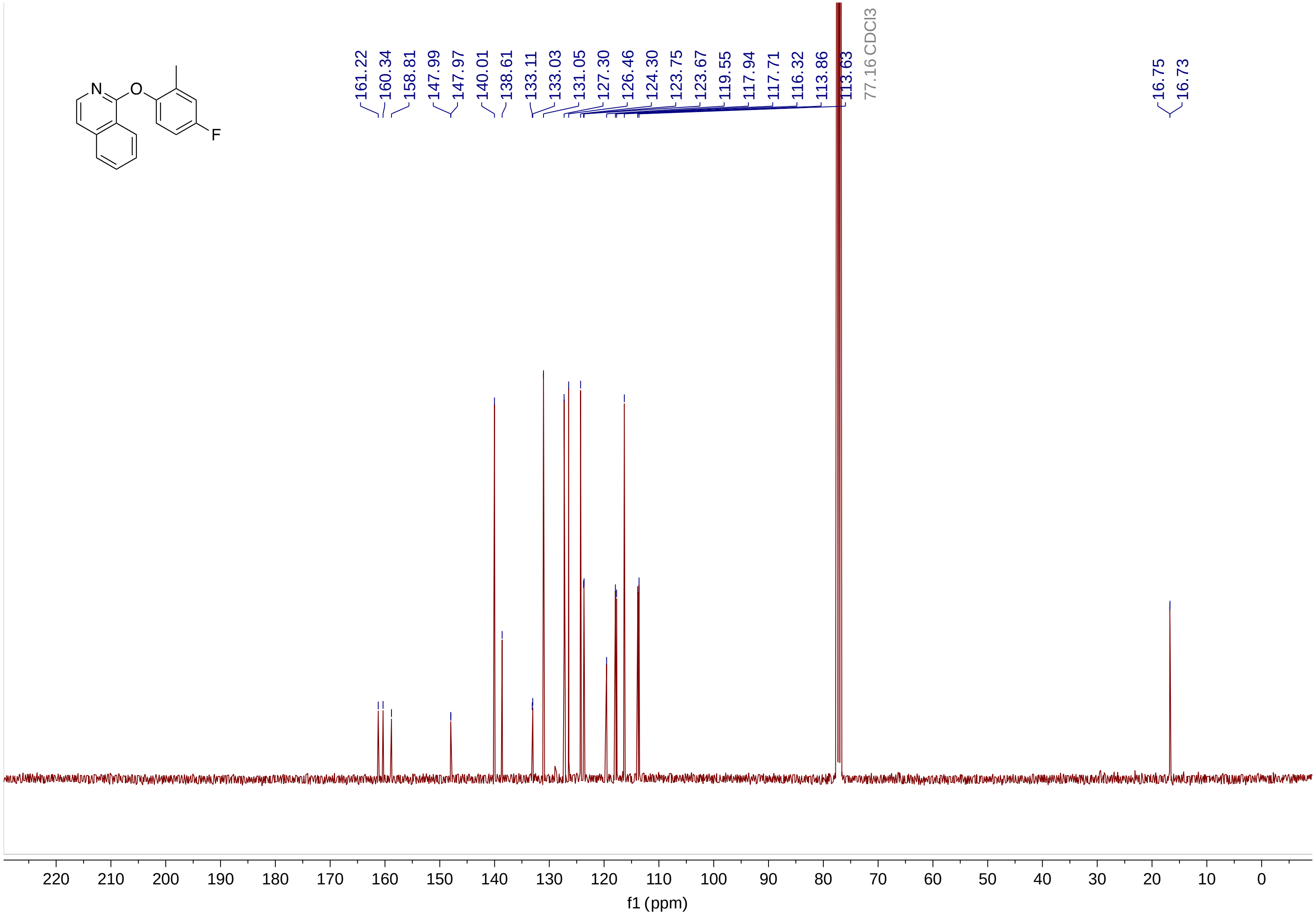

14 - ^{19}F NMR (376 MHz, $CDCl_3$):

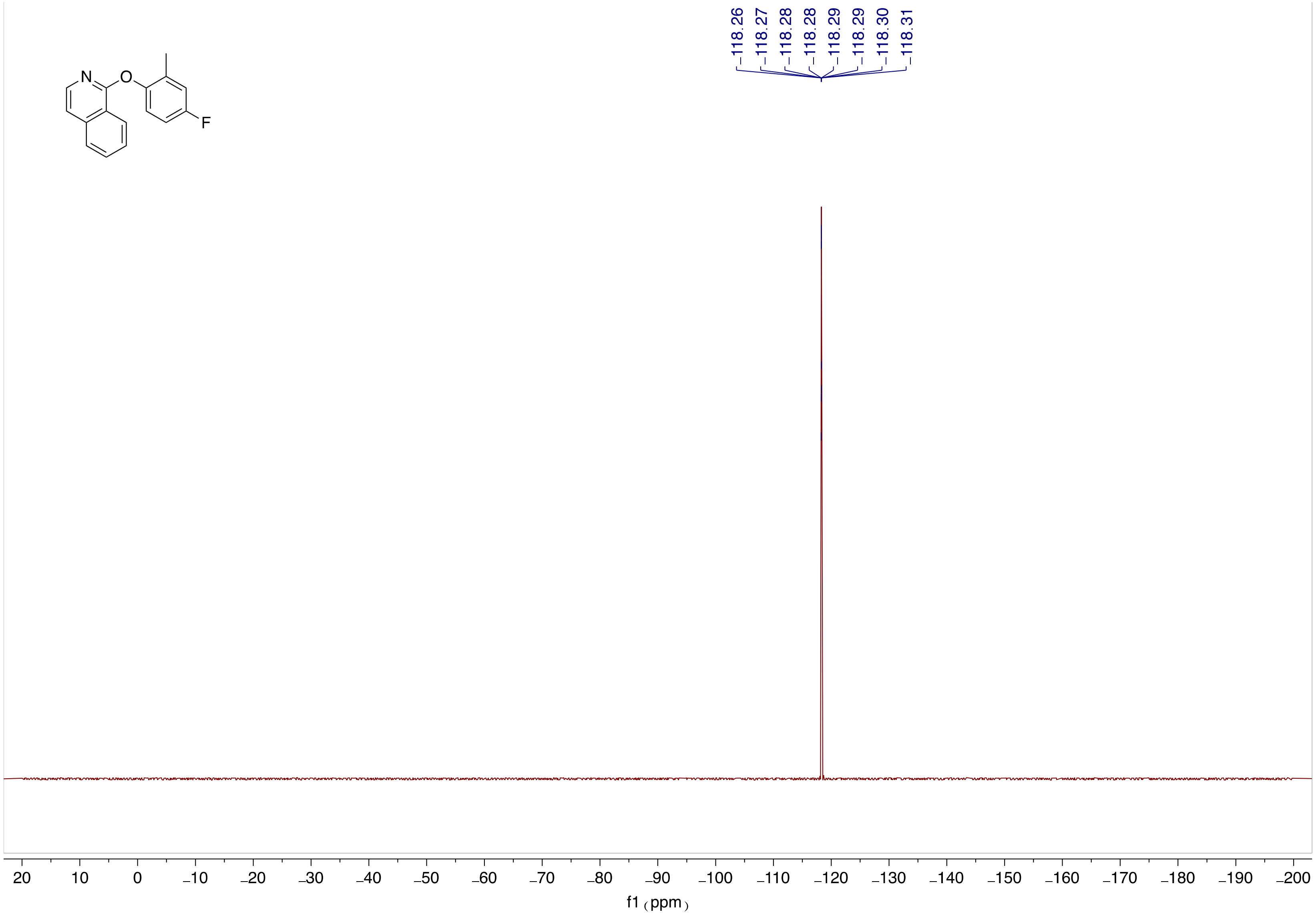

15 - ^{1}H NMR (400 MHz, $CDCl_3$):

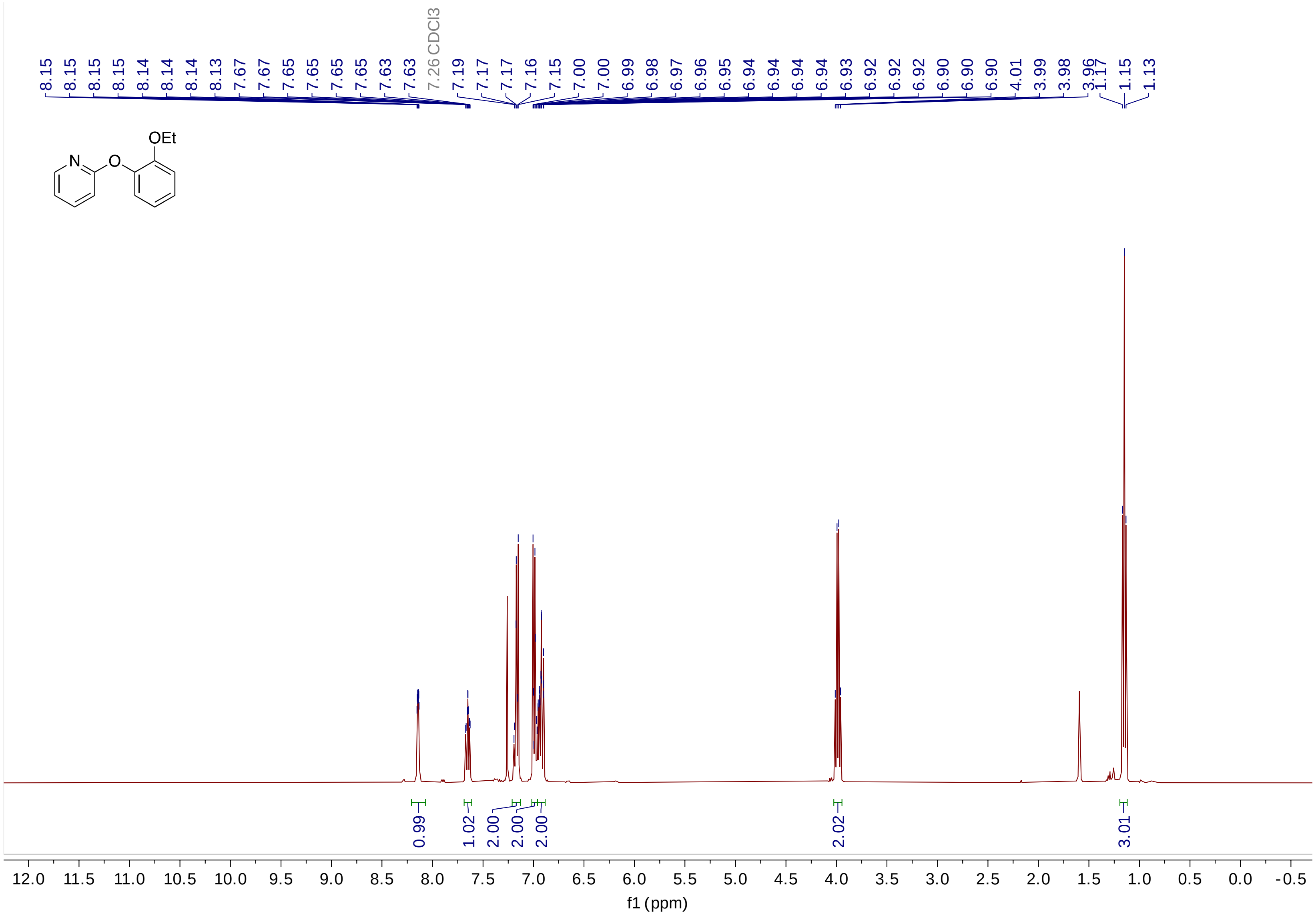

15 - $^{13}C\{^{1}H\}$ NMR (101 MHz, $CDCl_3$):

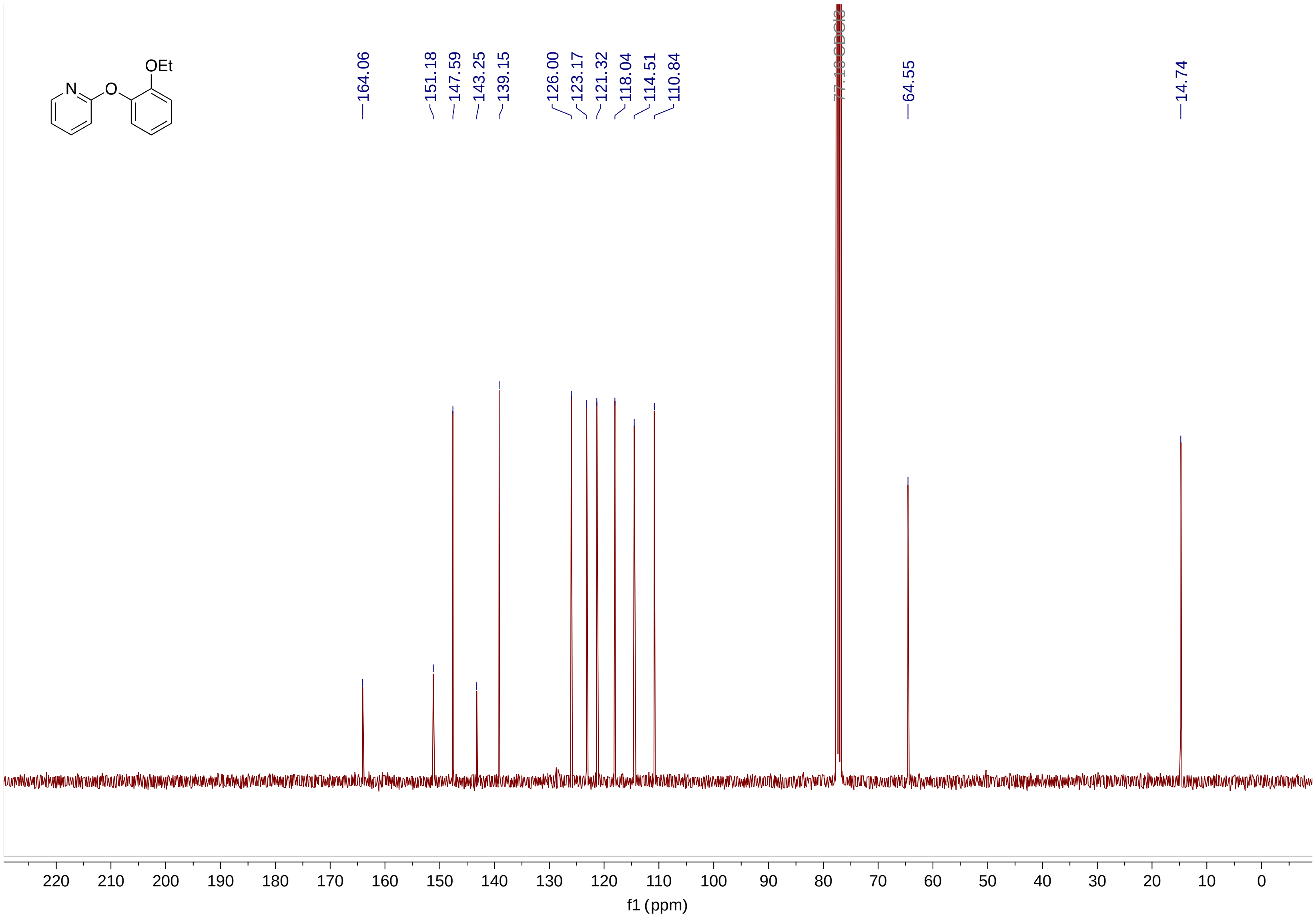

16 - ^{1}H NMR (400 MHz, $CDCl_3$):

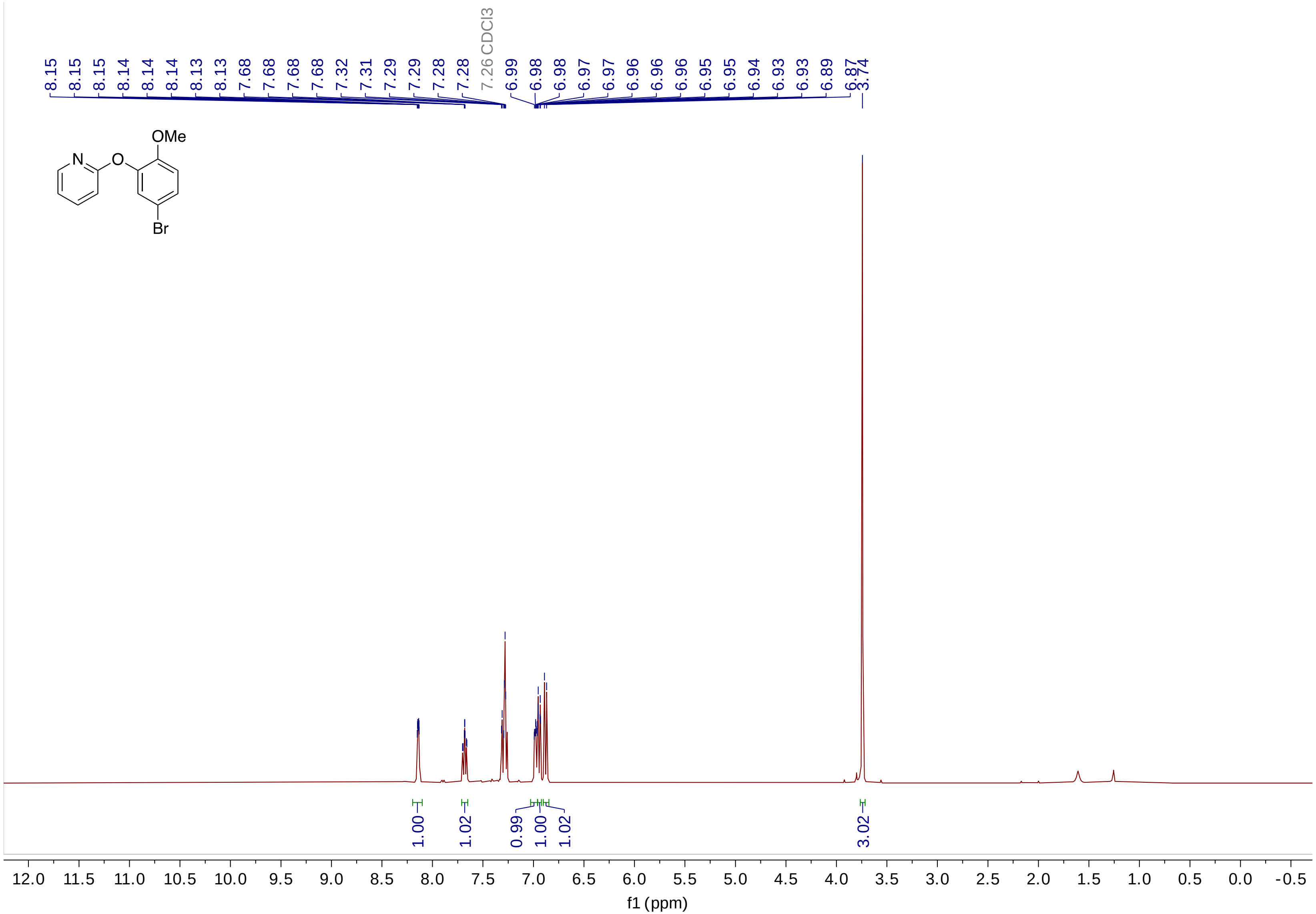

16 - $^{13}C\{^{1}H\}$ NMR (101 MHz, $CDCl_3$):

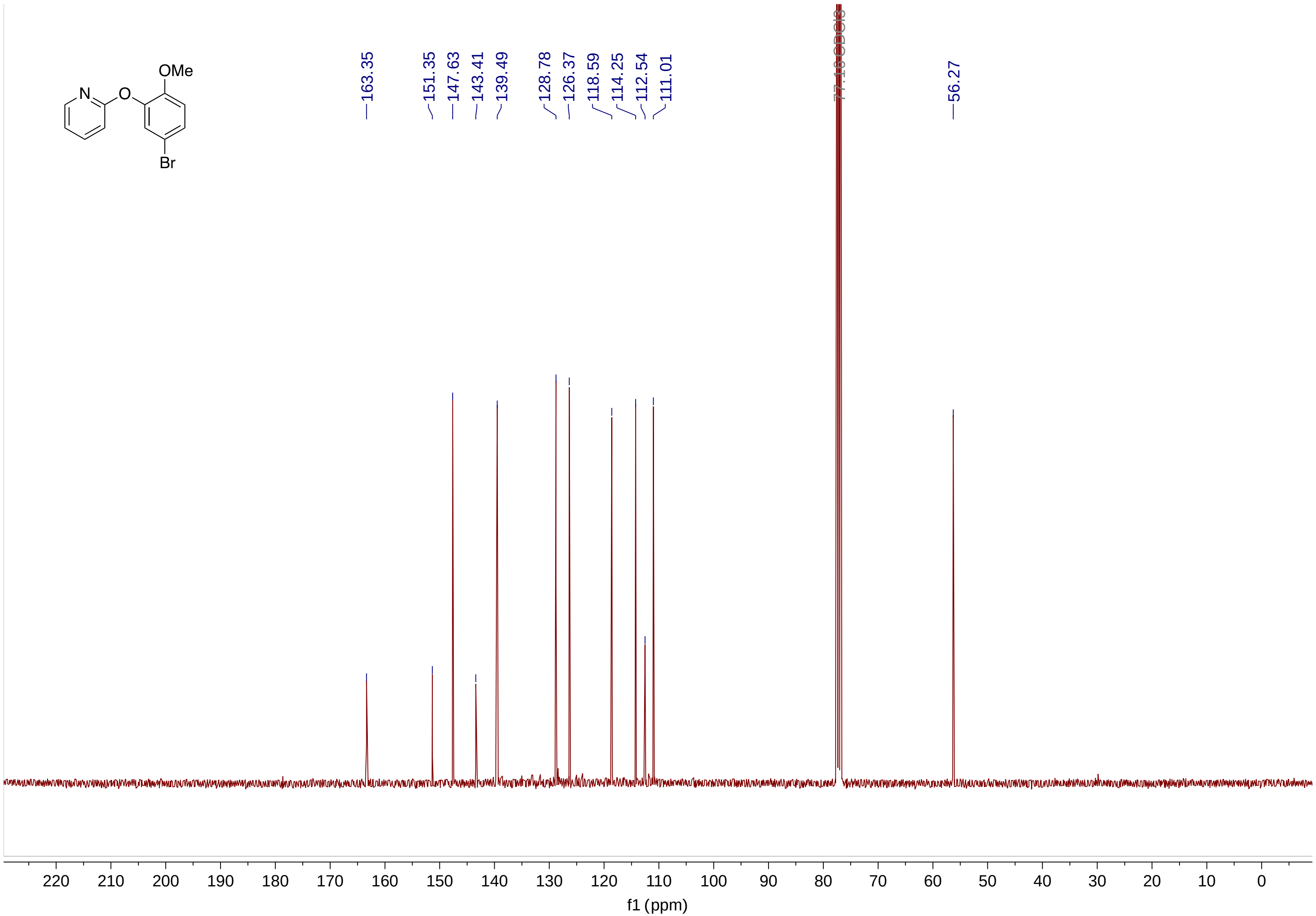

17 - ^{1}H NMR (400 MHz, $CDCl_3$):

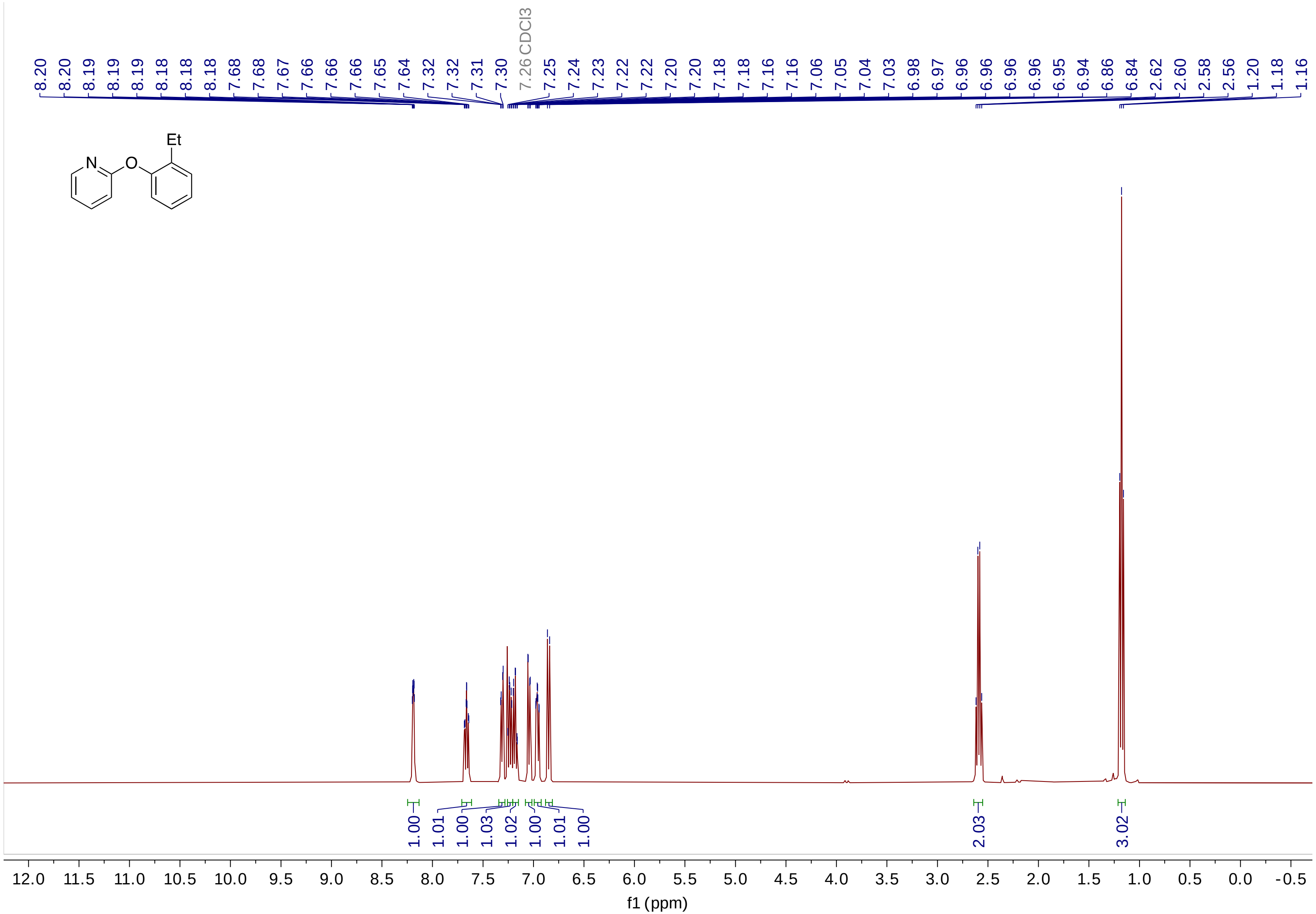

17 - $^{13}C\{^{1}H\}$ NMR (101 MHz, $CDCl_3$):

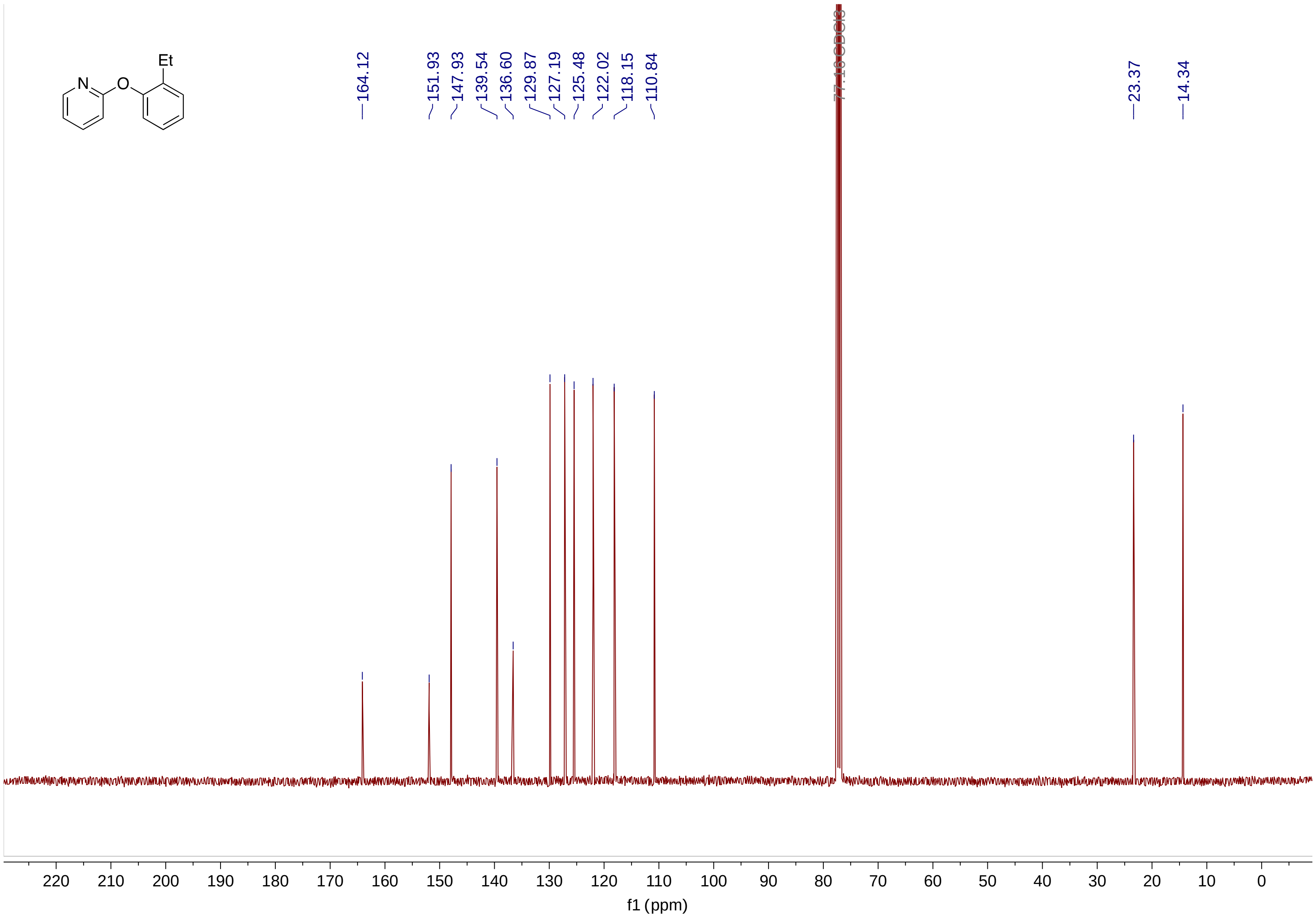

18 - ^{1}H NMR (400 MHz, CDCl$_3$):

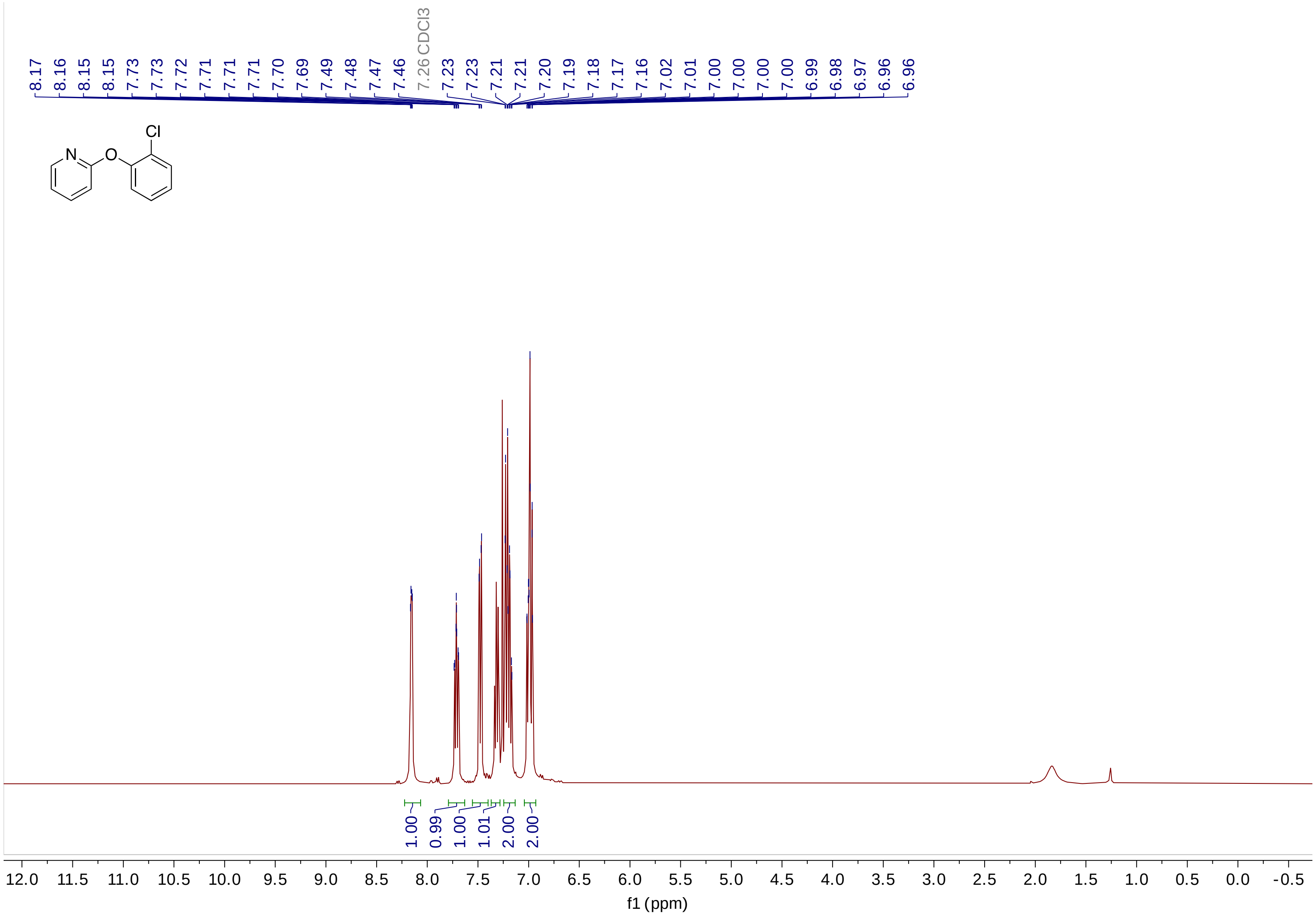

18 - $^{13}C\{^1H\}$ NMR (101 MHz, $CDCl_3$):

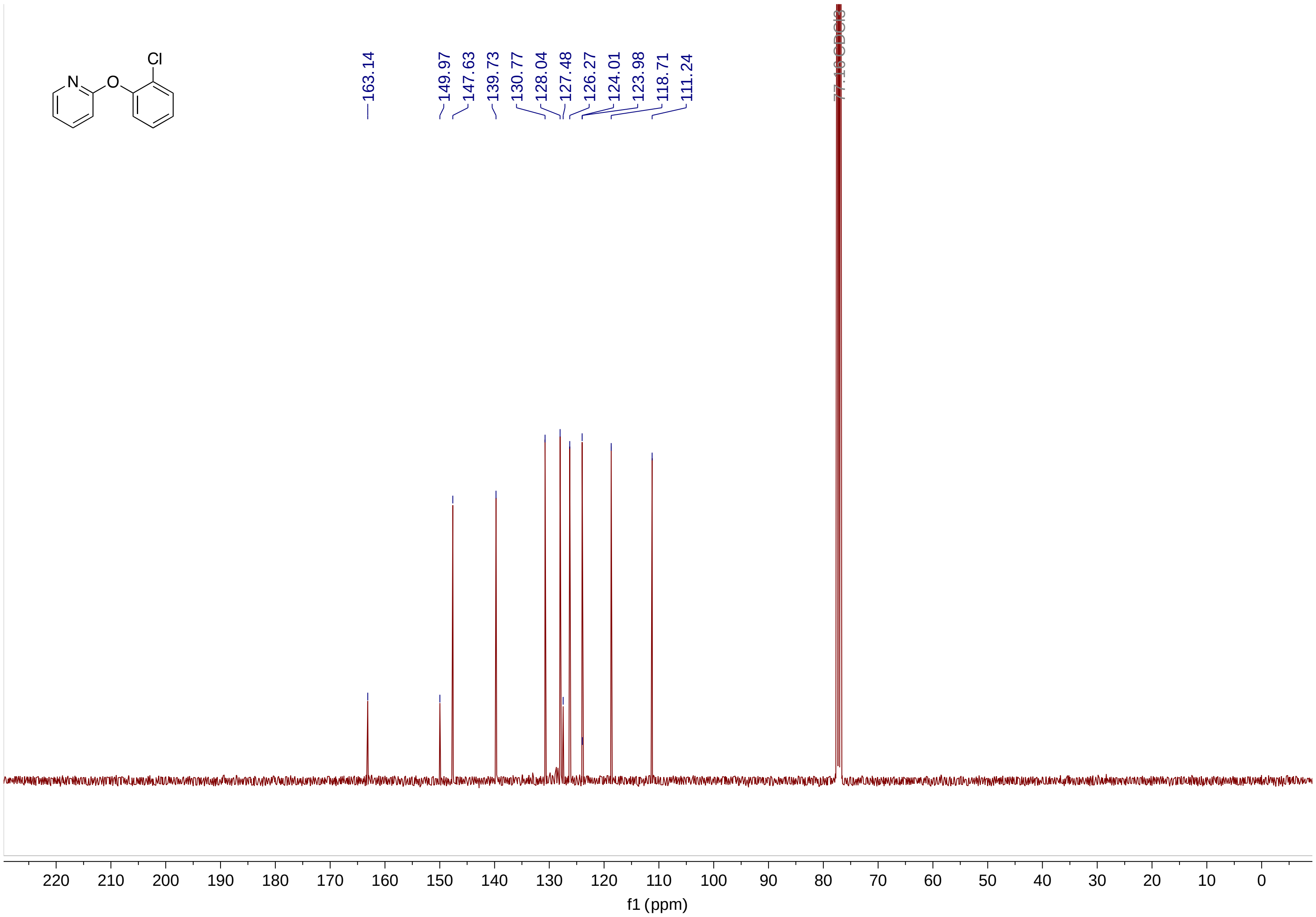

19 - ^{1}H NMR (400 MHz, $CDCl_3$):

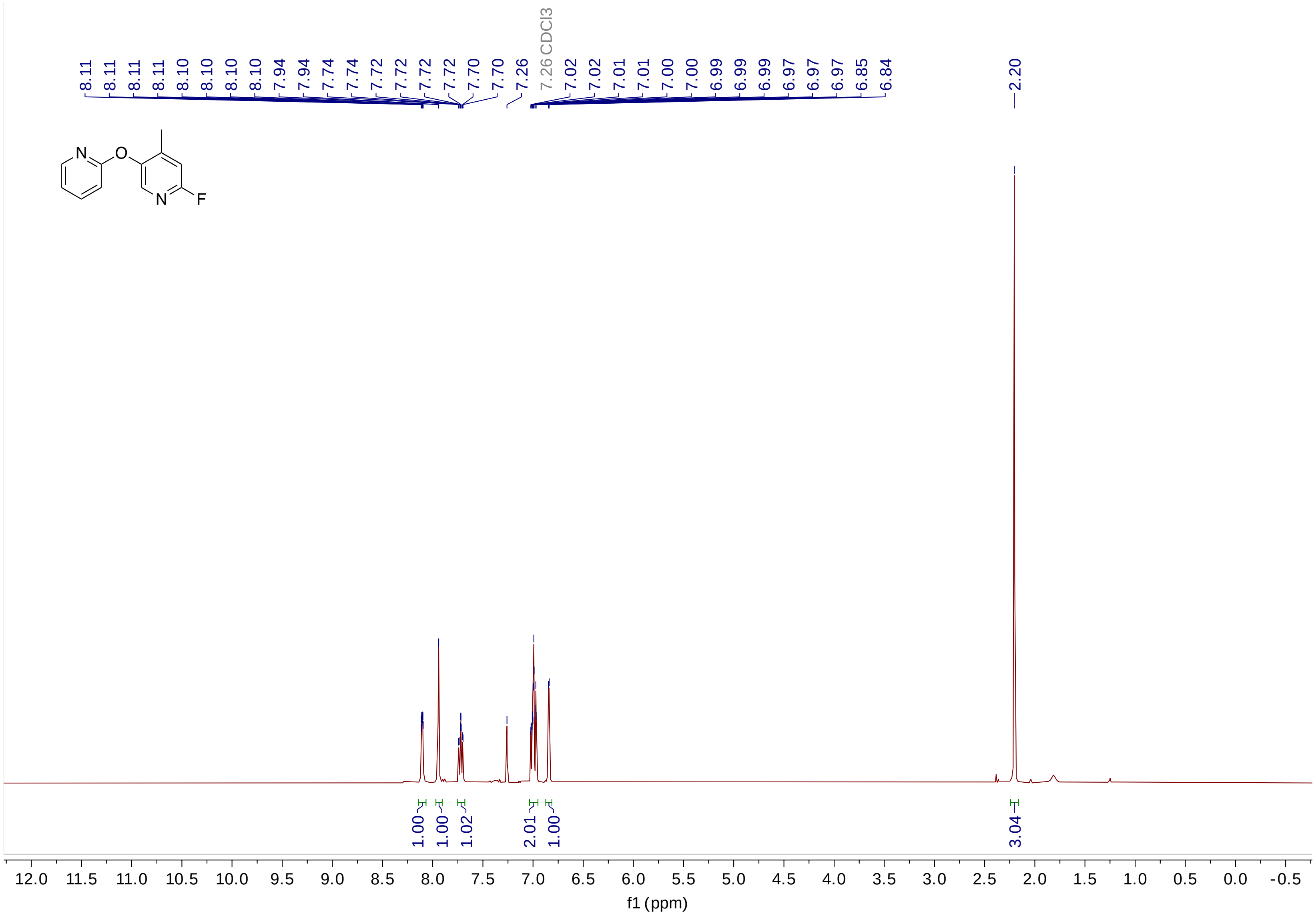

19 - $^{13}C\{^{1}H\}$ NMR (101 MHz, $CDCl_3$):

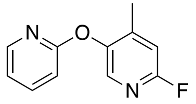

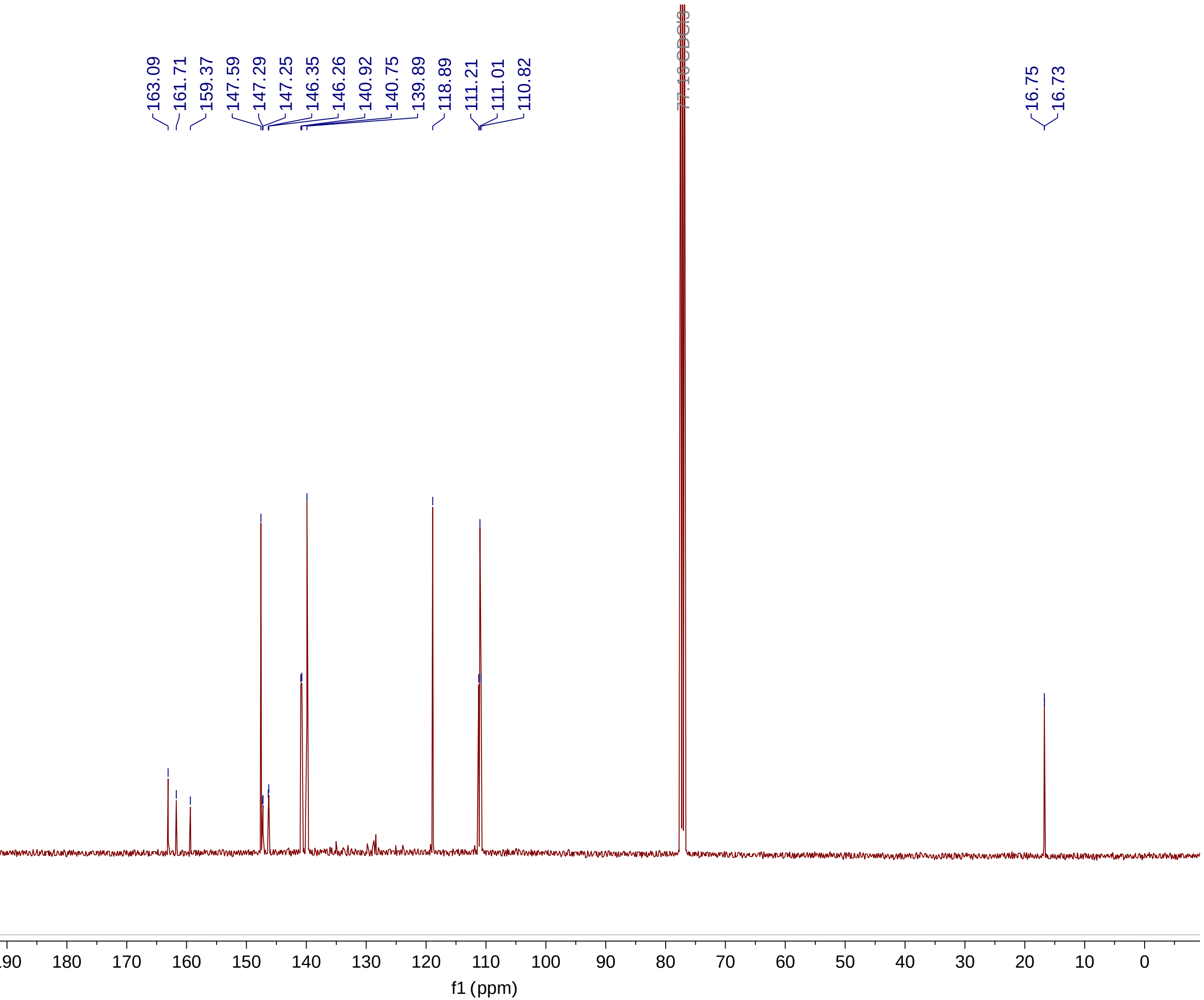

19 - ^{19}F NMR (376 MHz, $CDCl_3$):

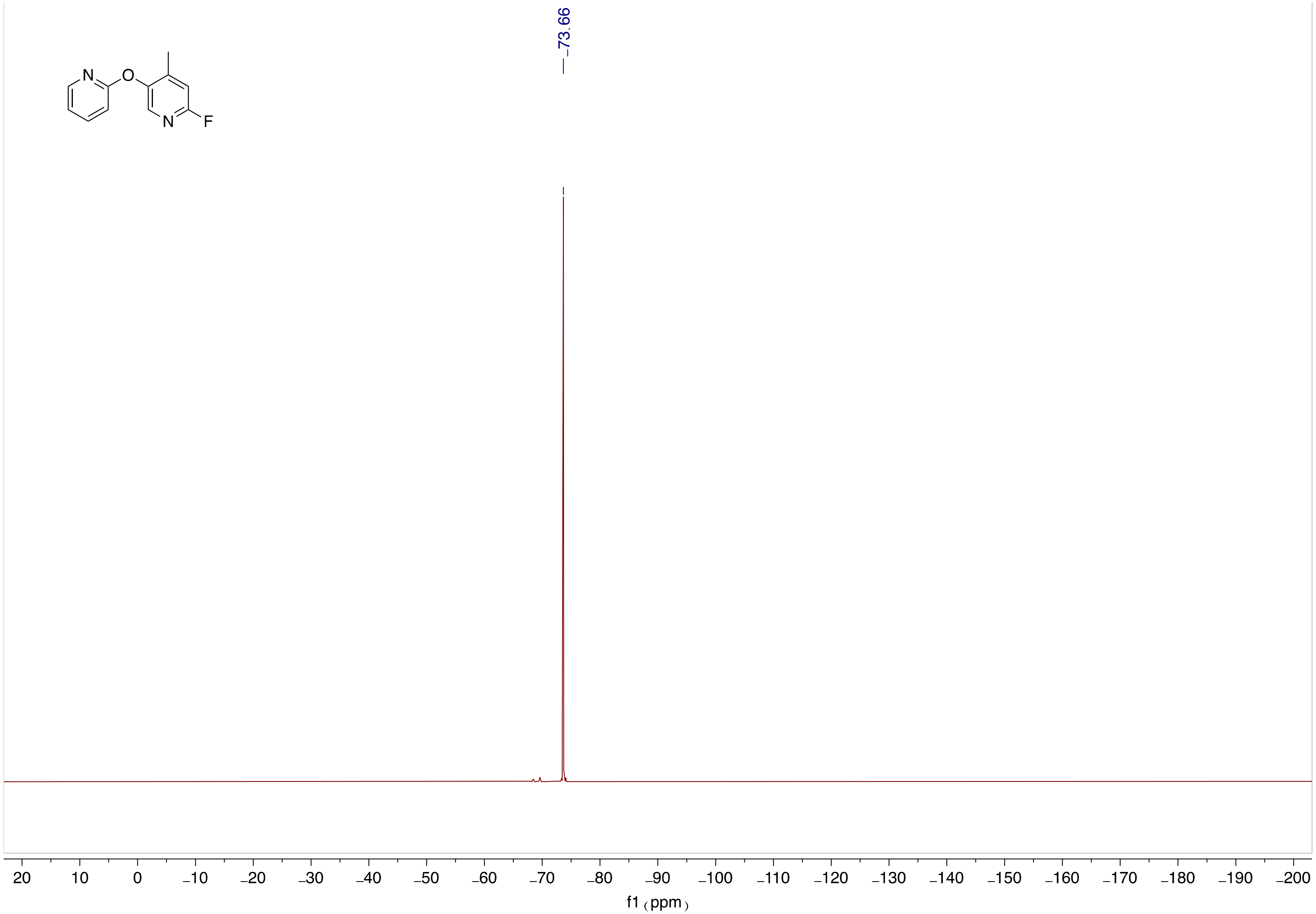

20 - ^{1}H NMR (400 MHz, $CDCl_3$):

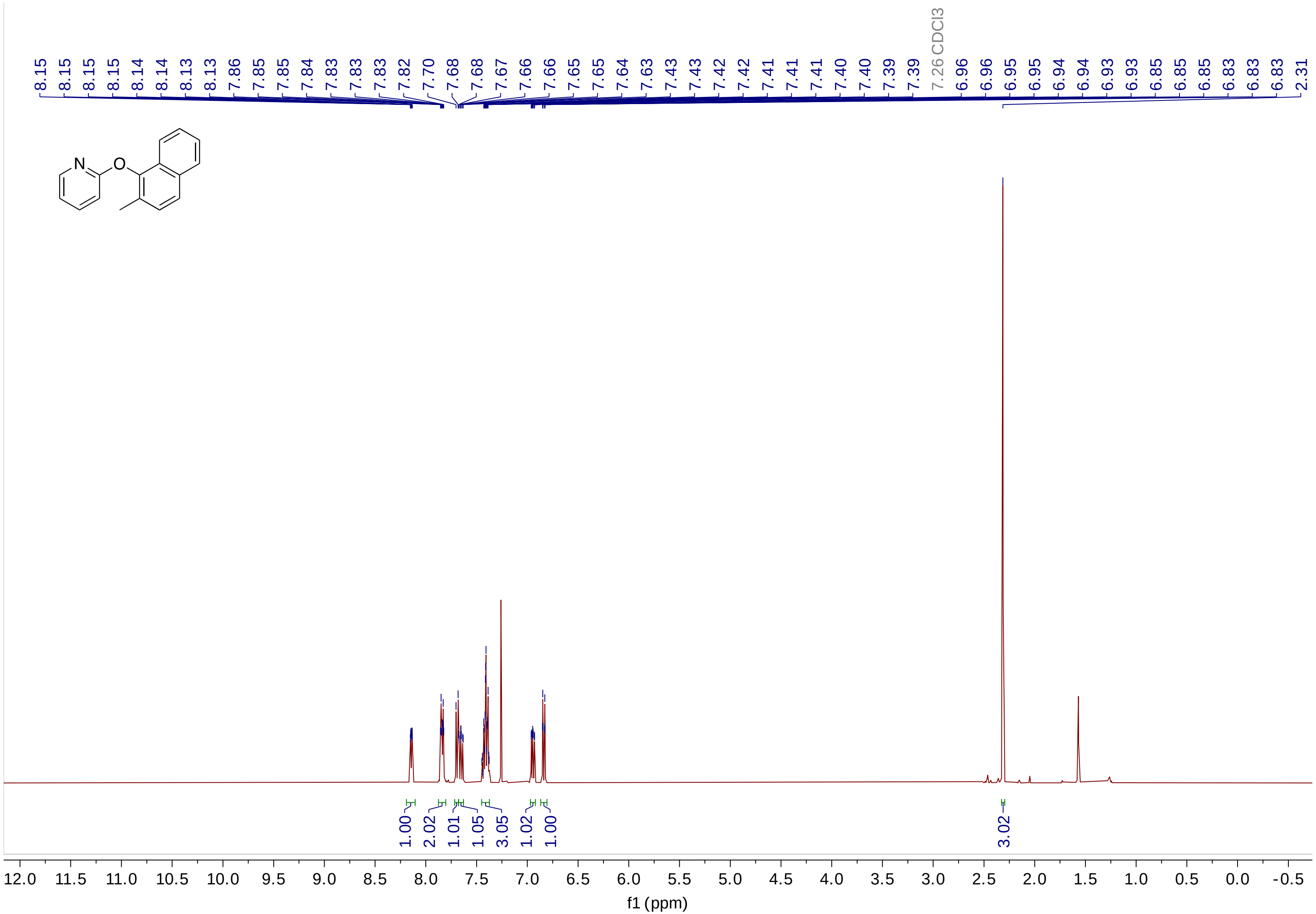

20 - $^{13}C\{^{1}H\}$ NMR (101 MHz, $CDCl_3$):

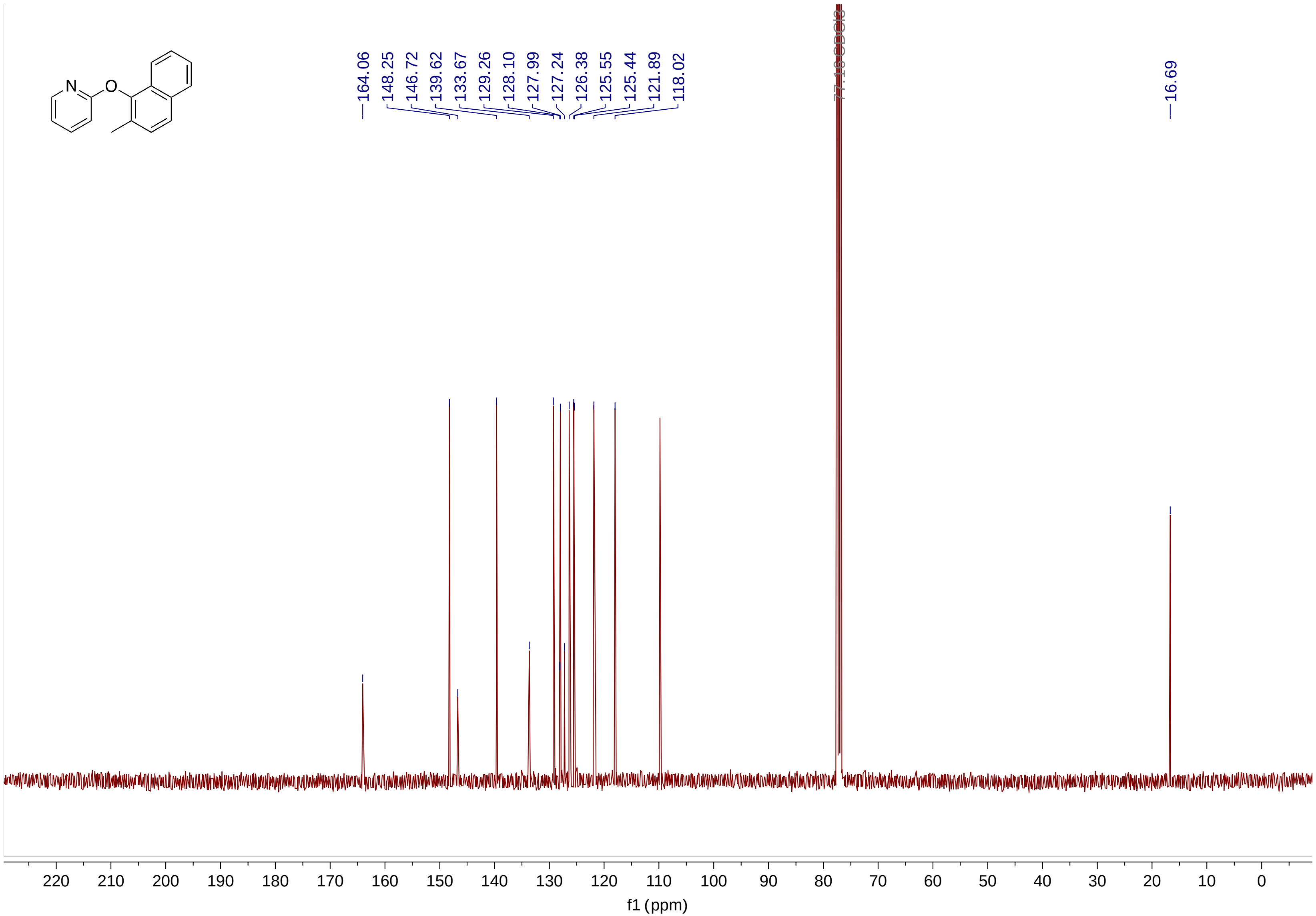

21 - ^{1}H NMR (400 MHz, $CDCl_3$):

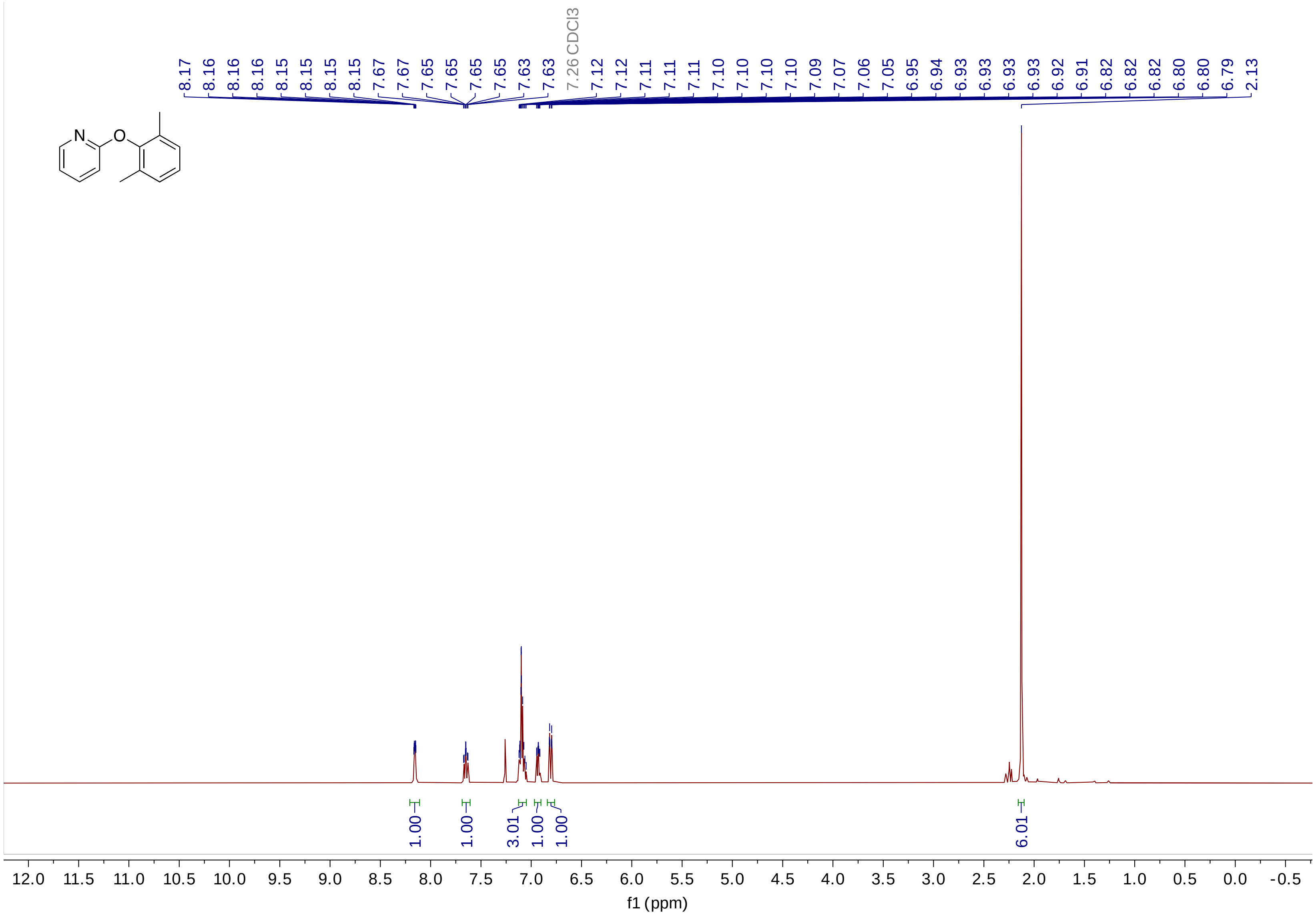

21 - $^{13}C\{^{1}H\}$ NMR (101 MHz, $CDCl_3$):

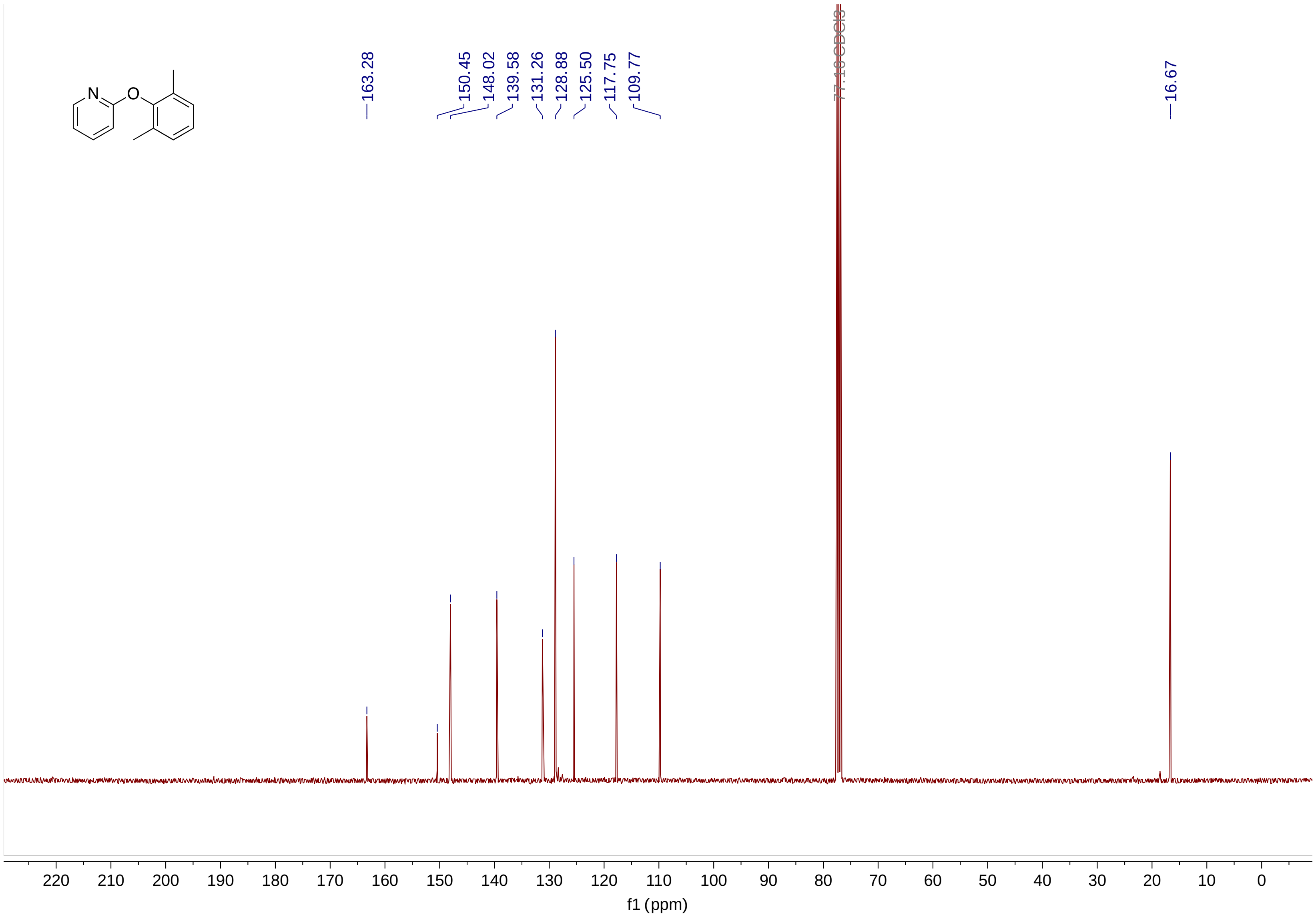

22 - ^{1}H NMR (400 MHz, $CDCl_3$):

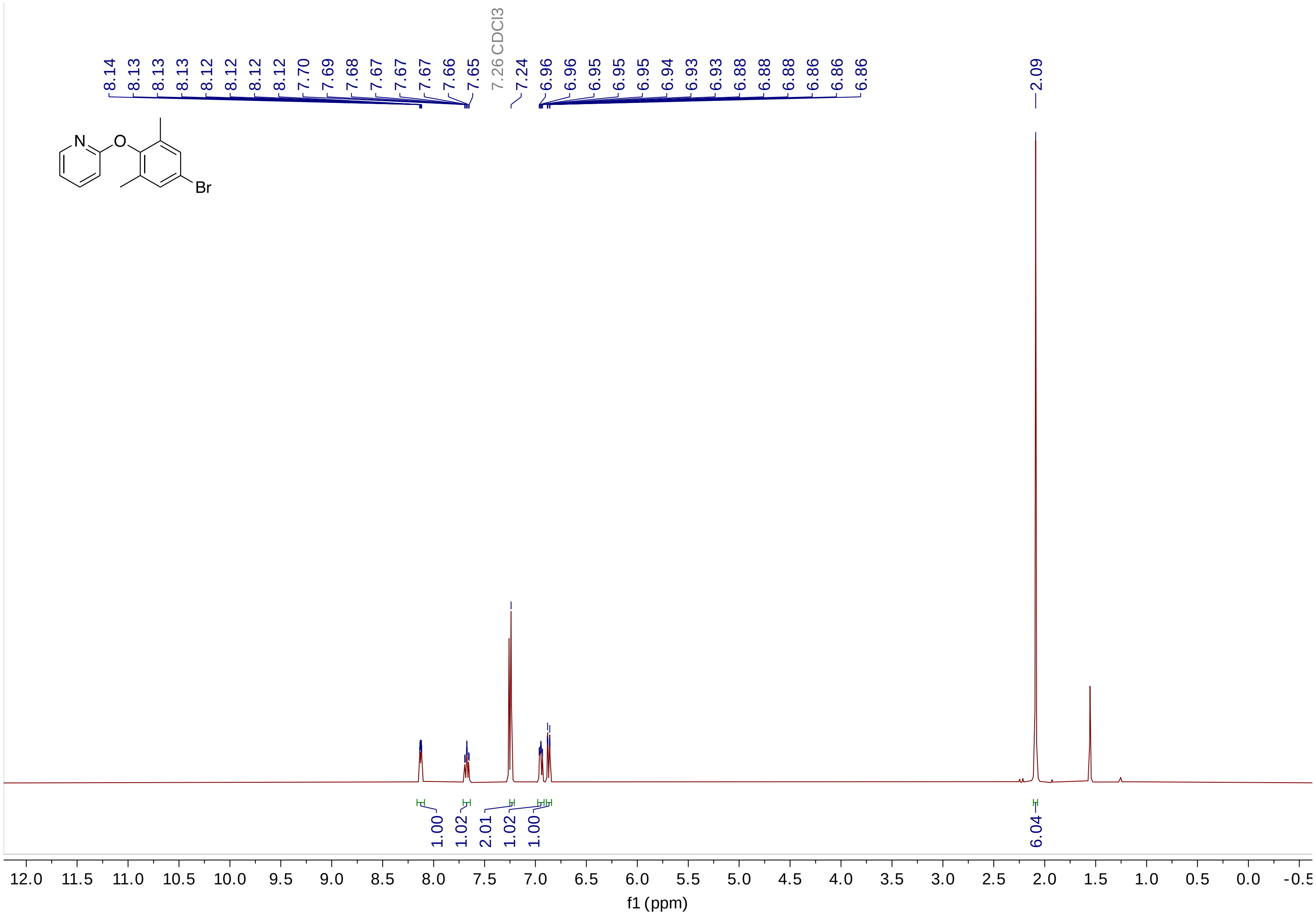

22 - $^{13}C\{^{1}H\}$ NMR (101 MHz, $CDCl_3$):

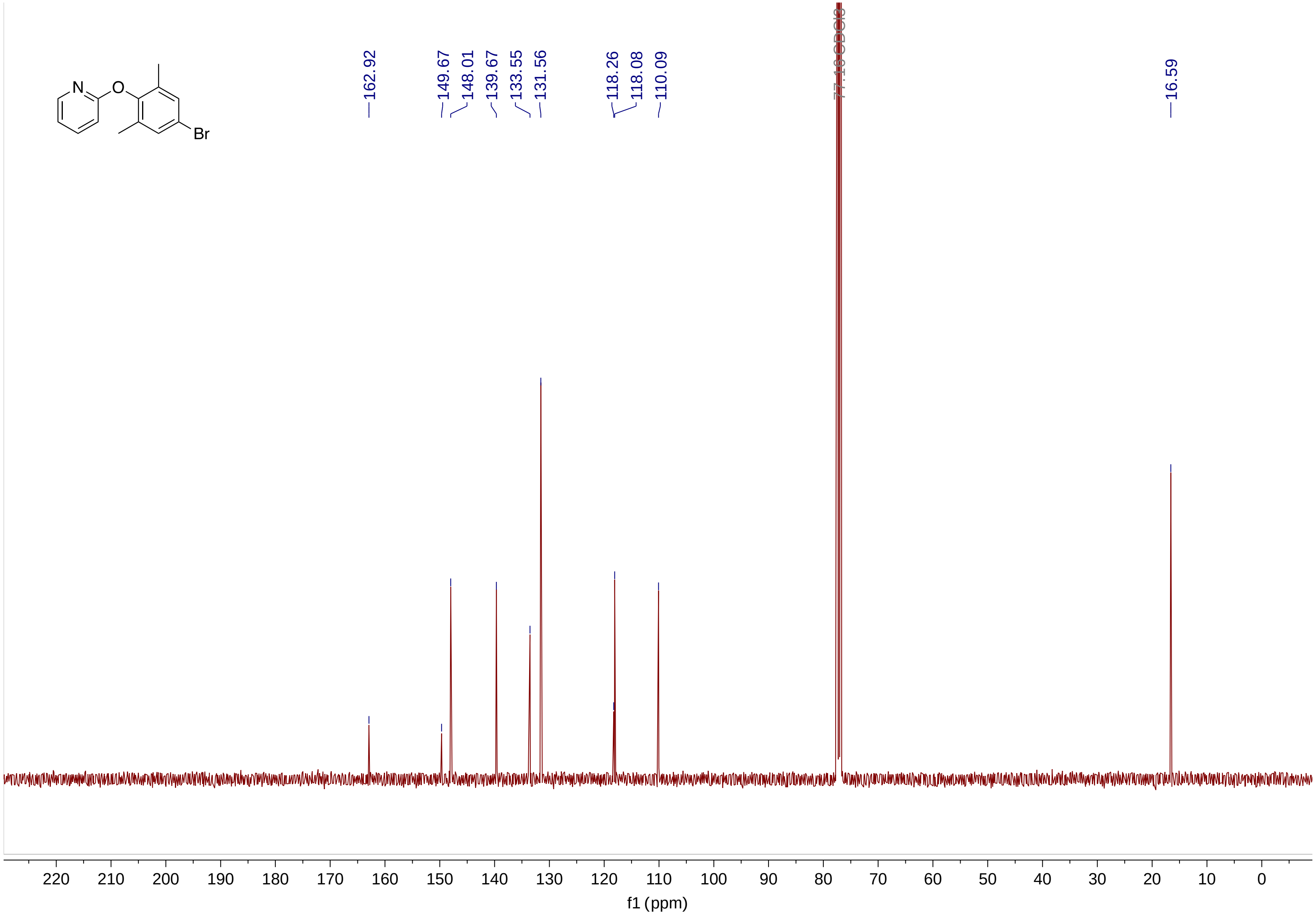

5 - ^{1}H NMR (400 MHz, CDCl₃):

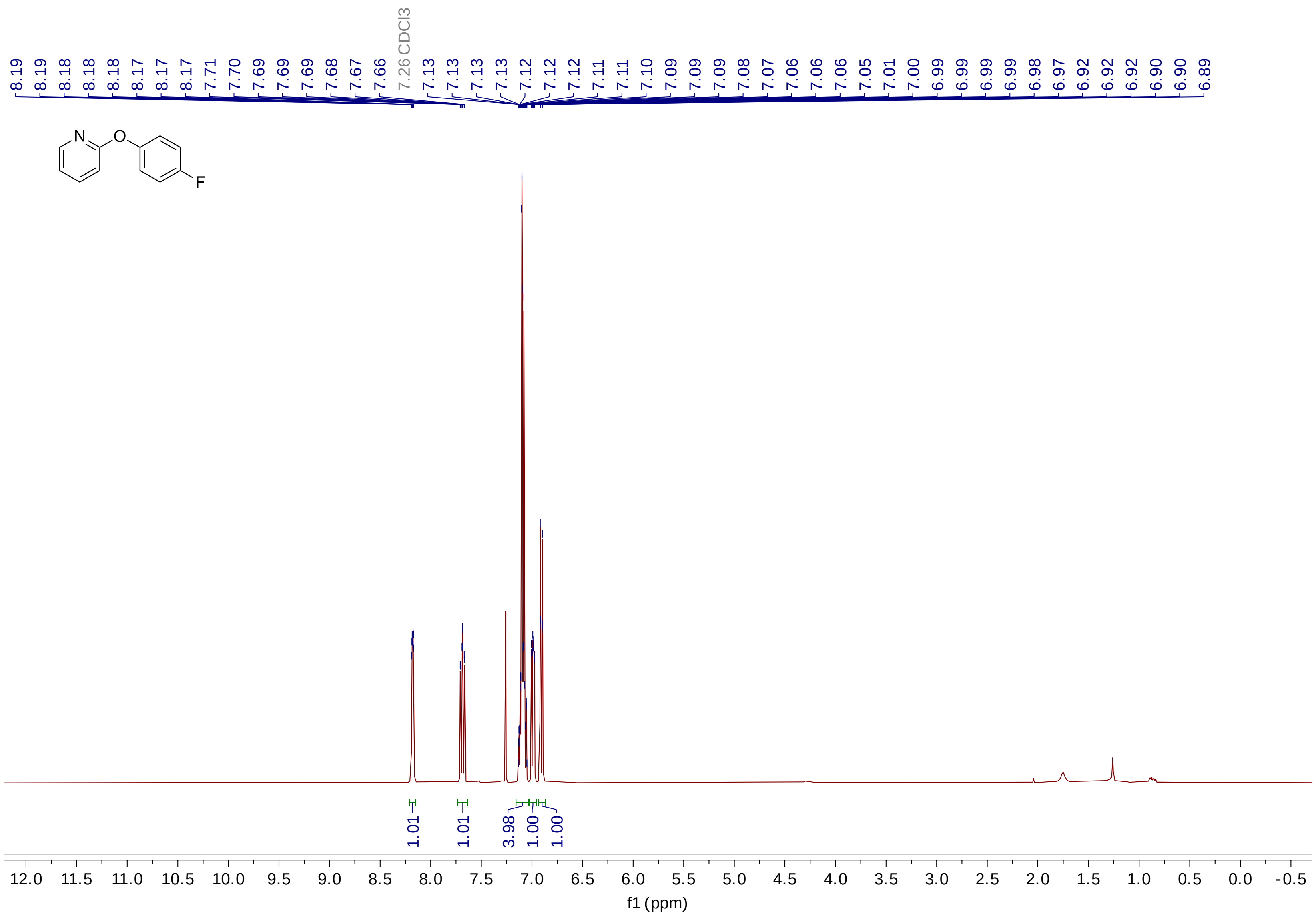

5 - $^{13}C\{^{1}H\}$ NMR (101 MHz, CDCl$_3$):

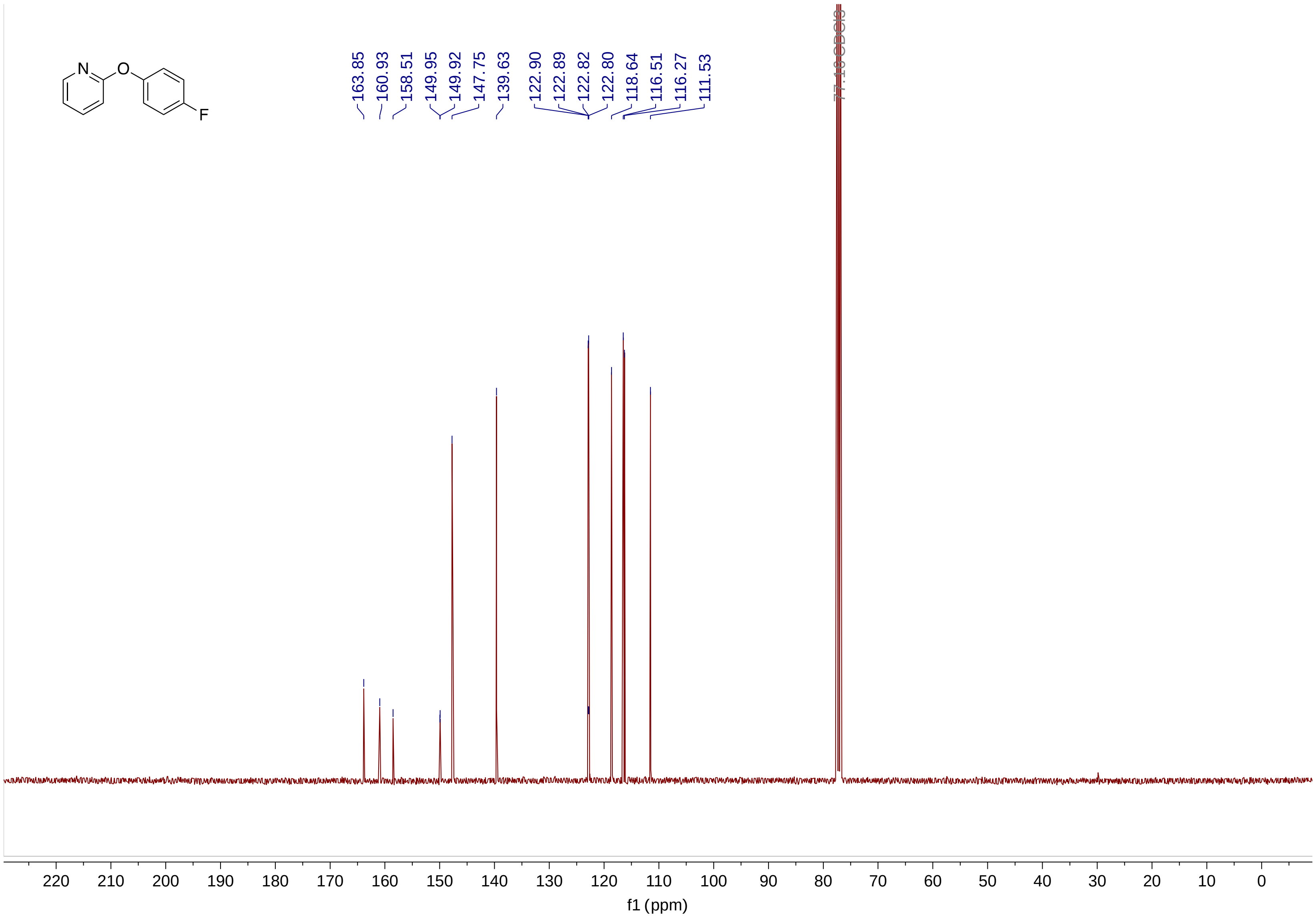

5 - ^{19}F NMR (376 MHz, $CDCl_3$):

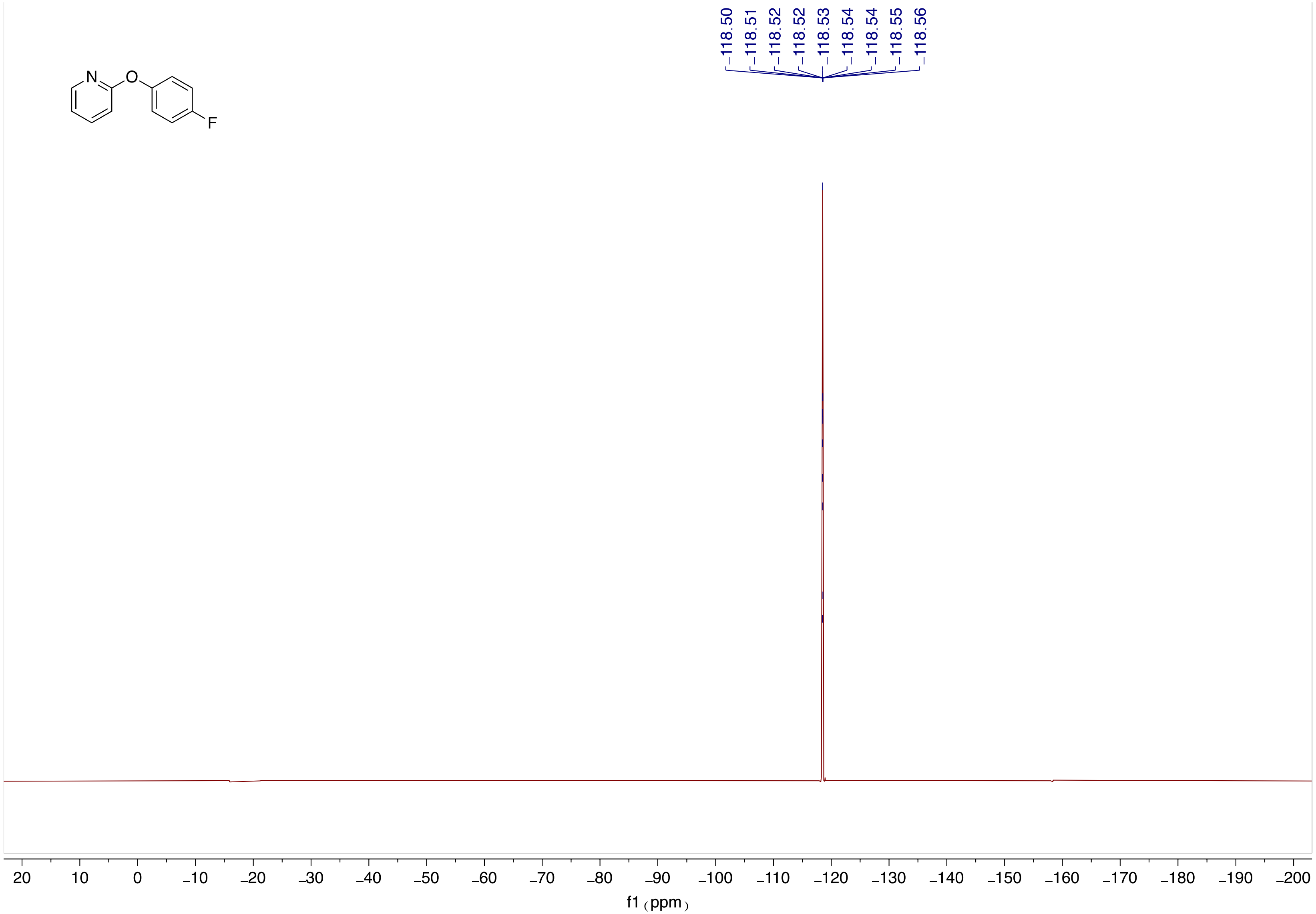

23 - ^{1}H NMR (400 MHz, $CDCl_3$):

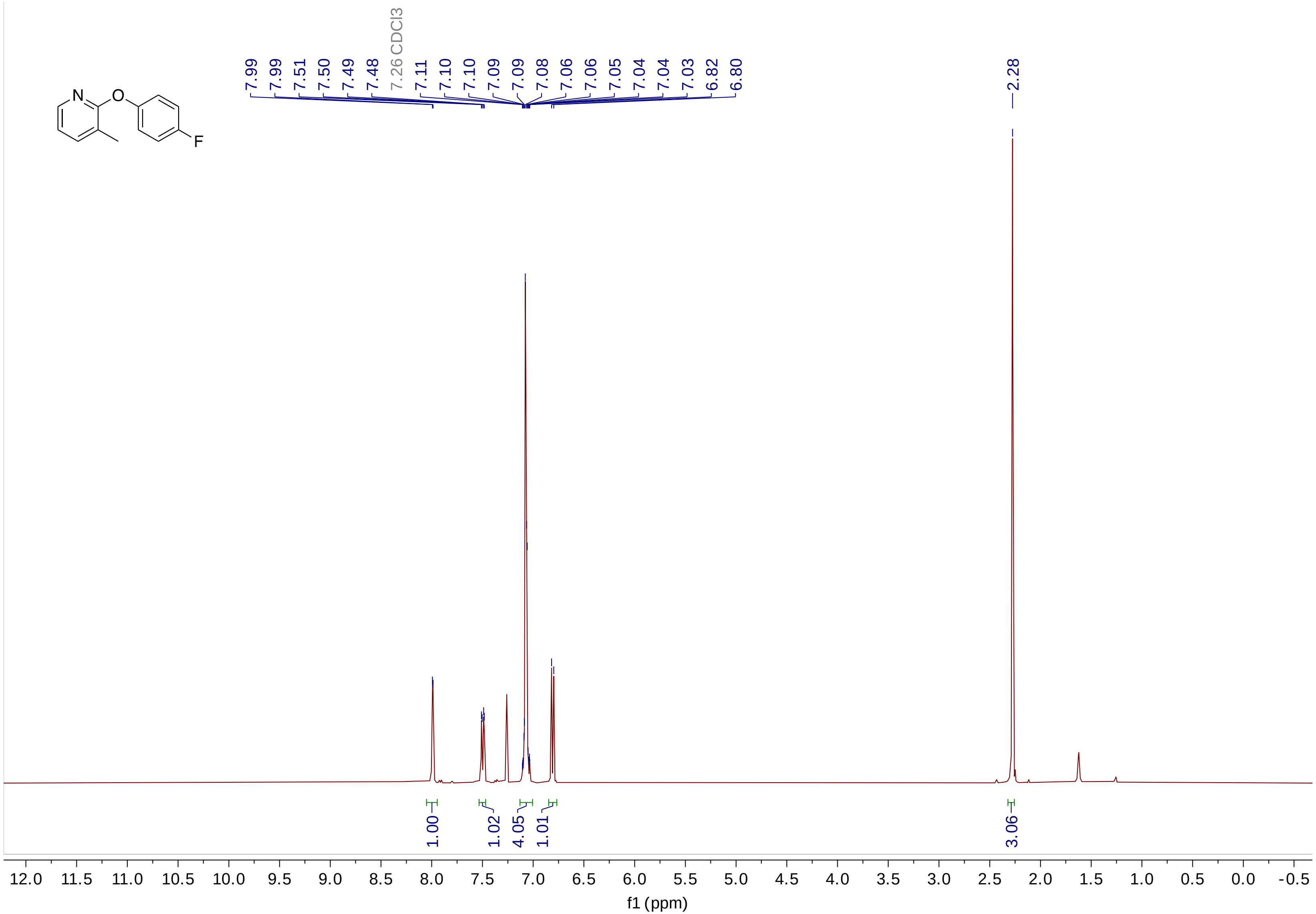

23 - $^{13}C\{^1H\}$ NMR (101 MHz, $CDCl_3$):

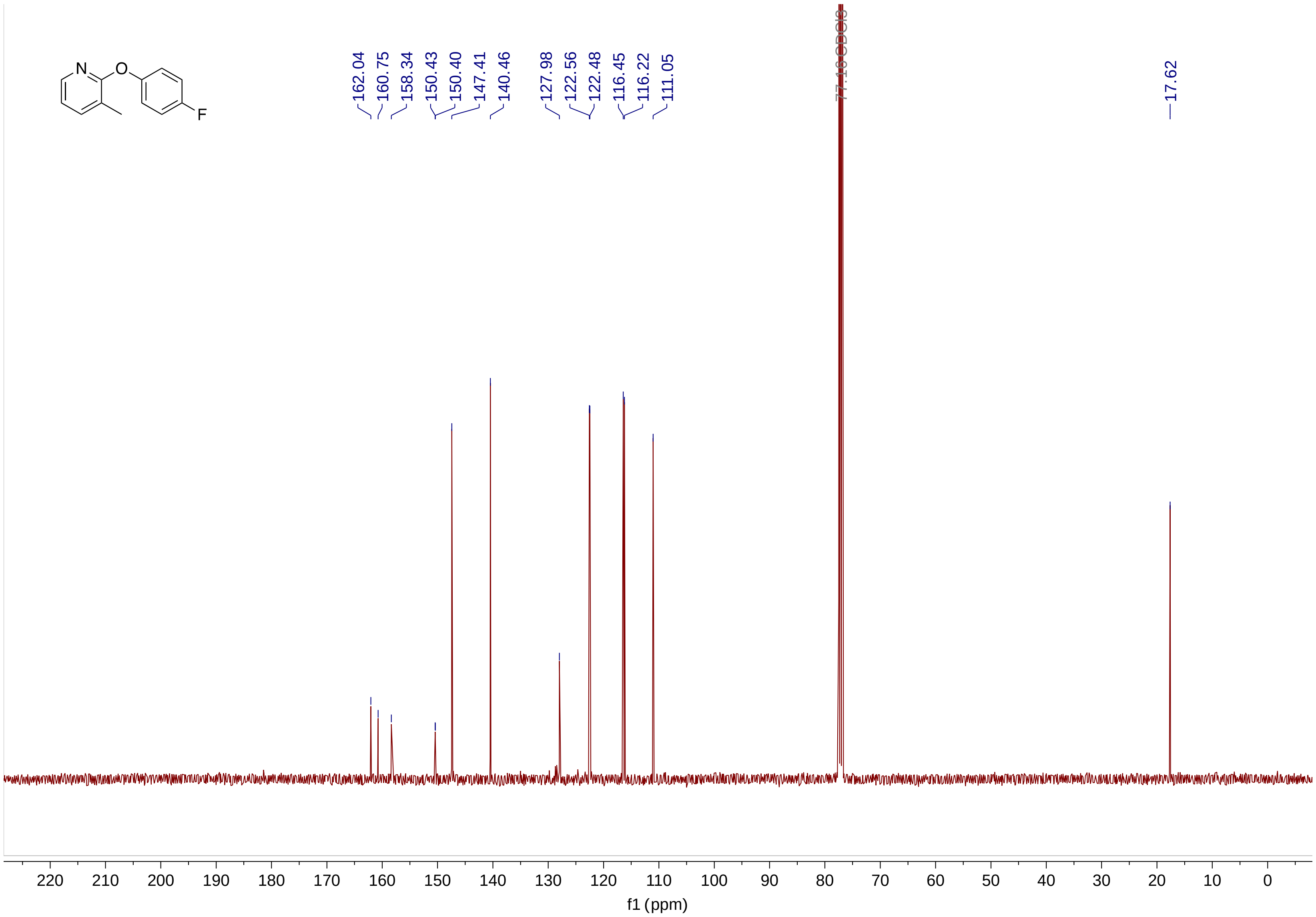

23 - ^{19}F NMR (377 MHz, $CDCl_3$):

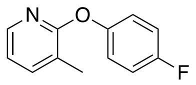

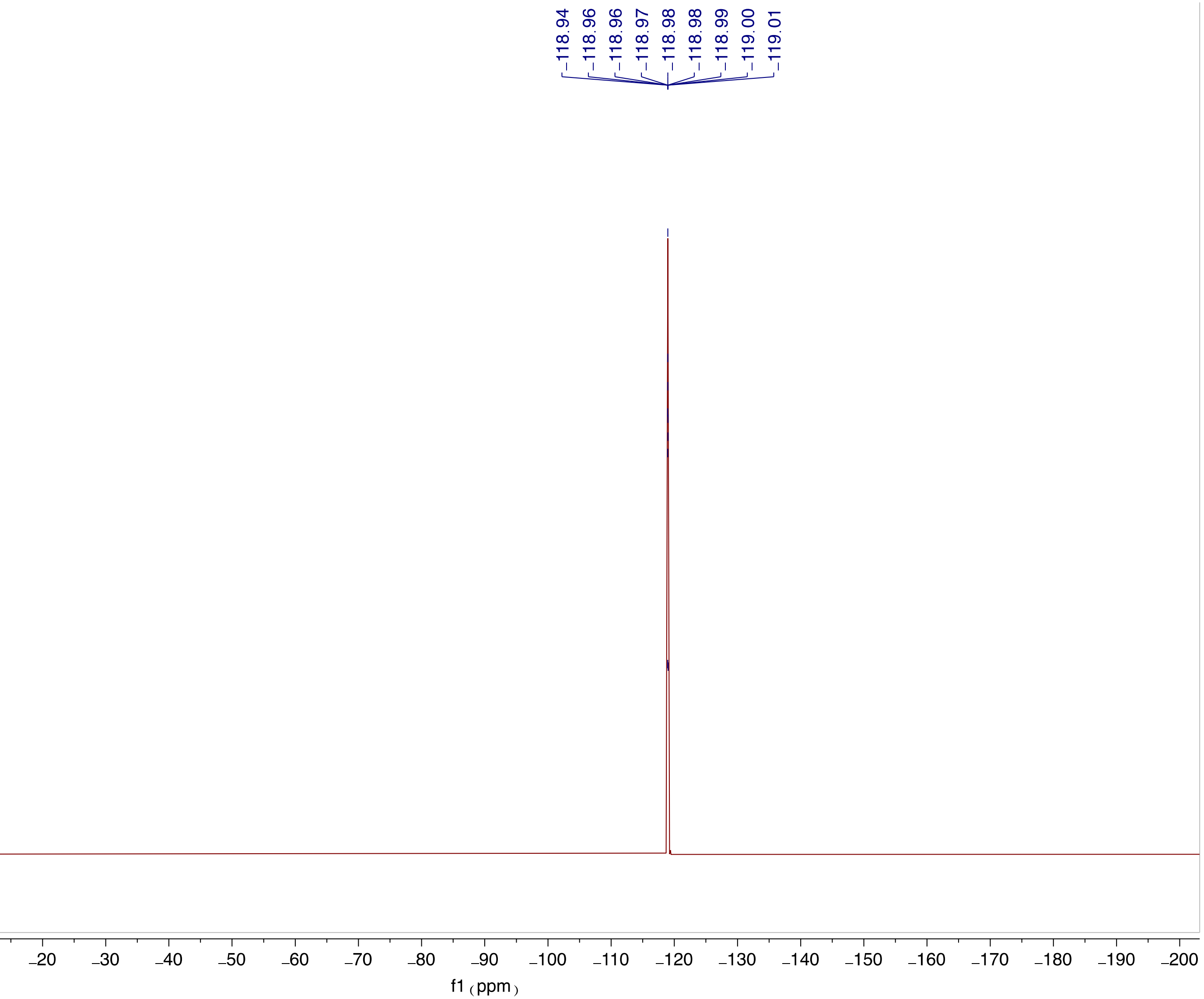

24 - ^{1}H NMR (400 MHz, $CDCl_3$):

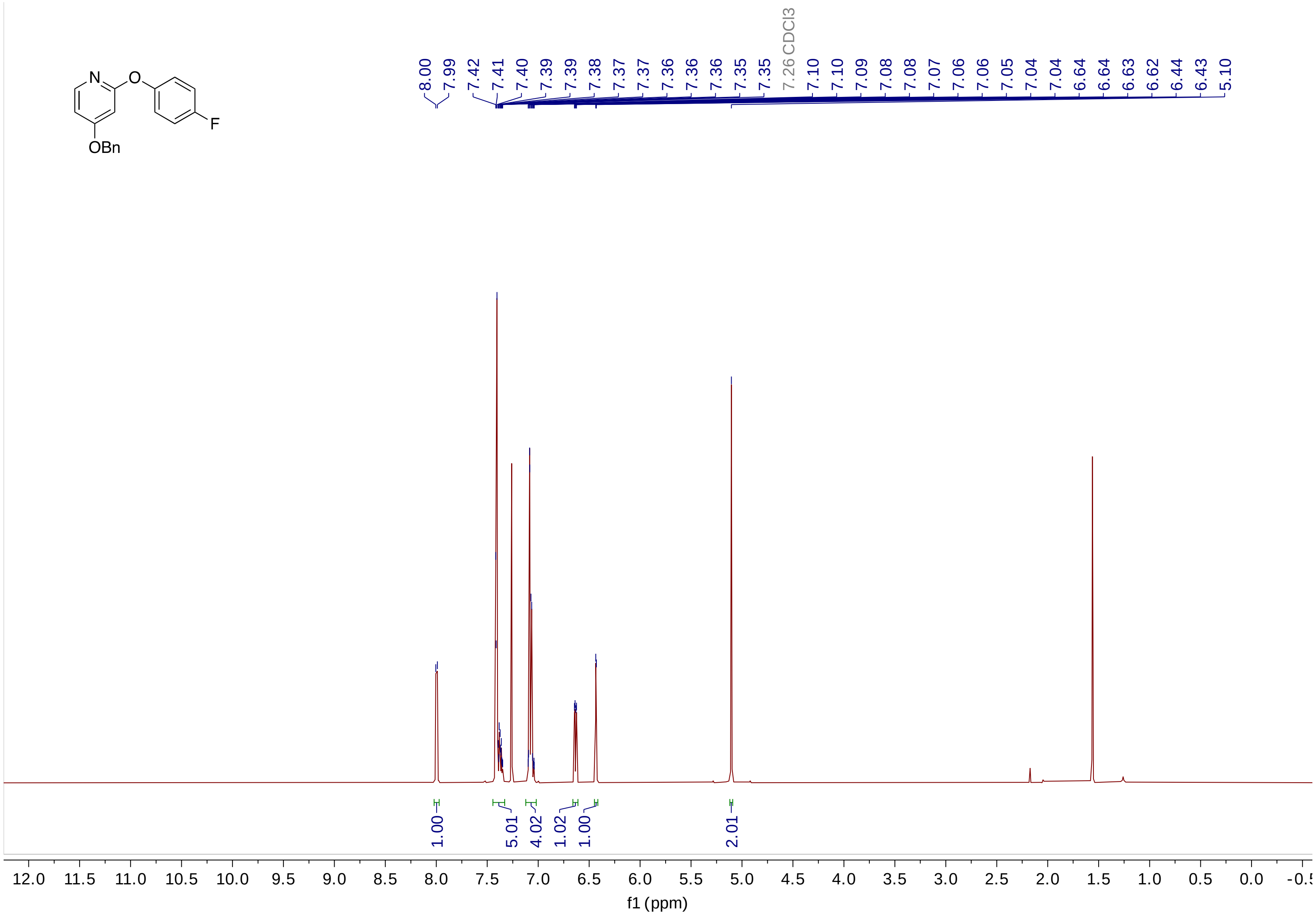

24 - $^{13}C\{^{1}H\}$ NMR (101 MHz, $CDCl_3$):

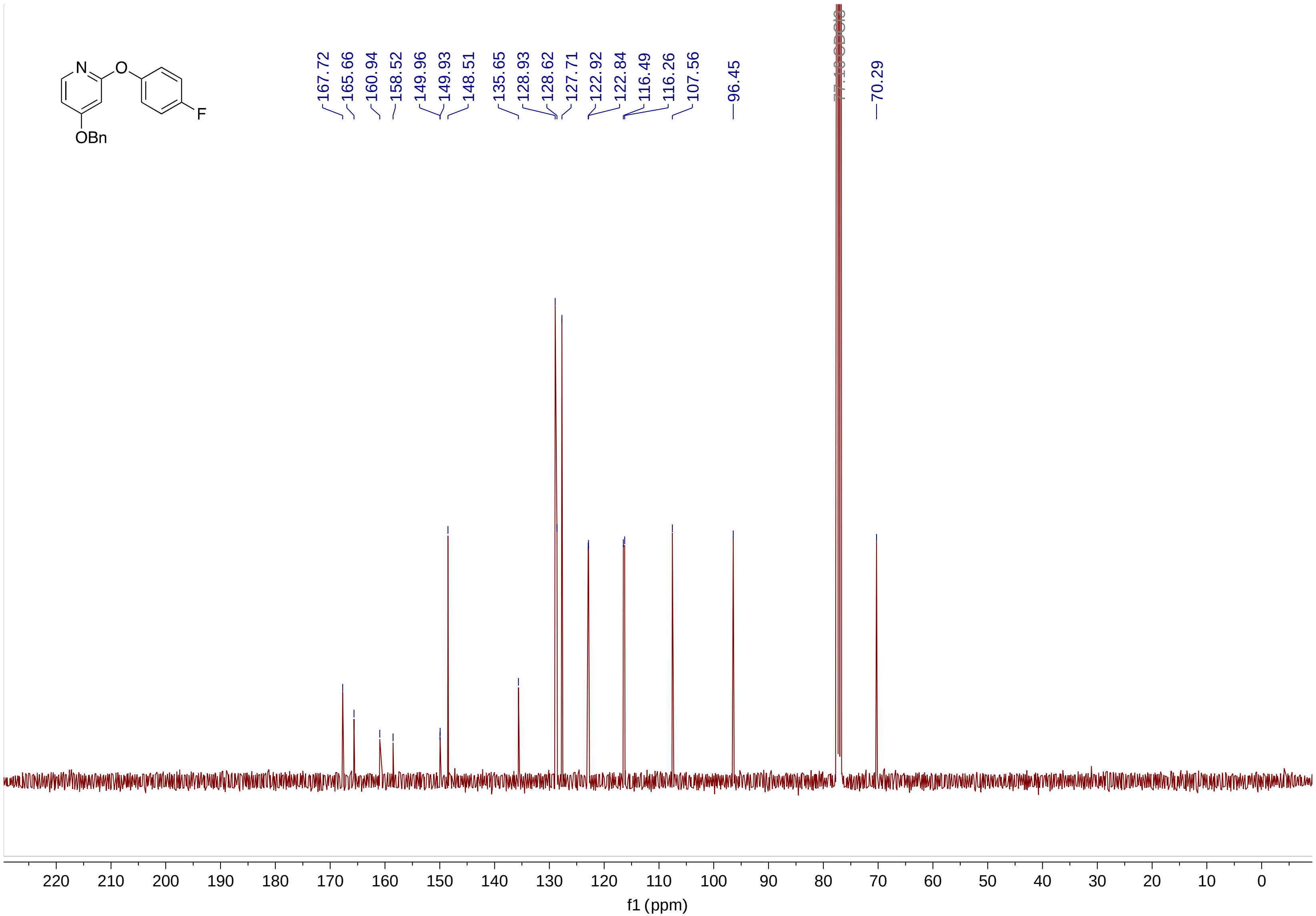

24 - ^{19}F NMR (376 MHz, CDCl$_3$):

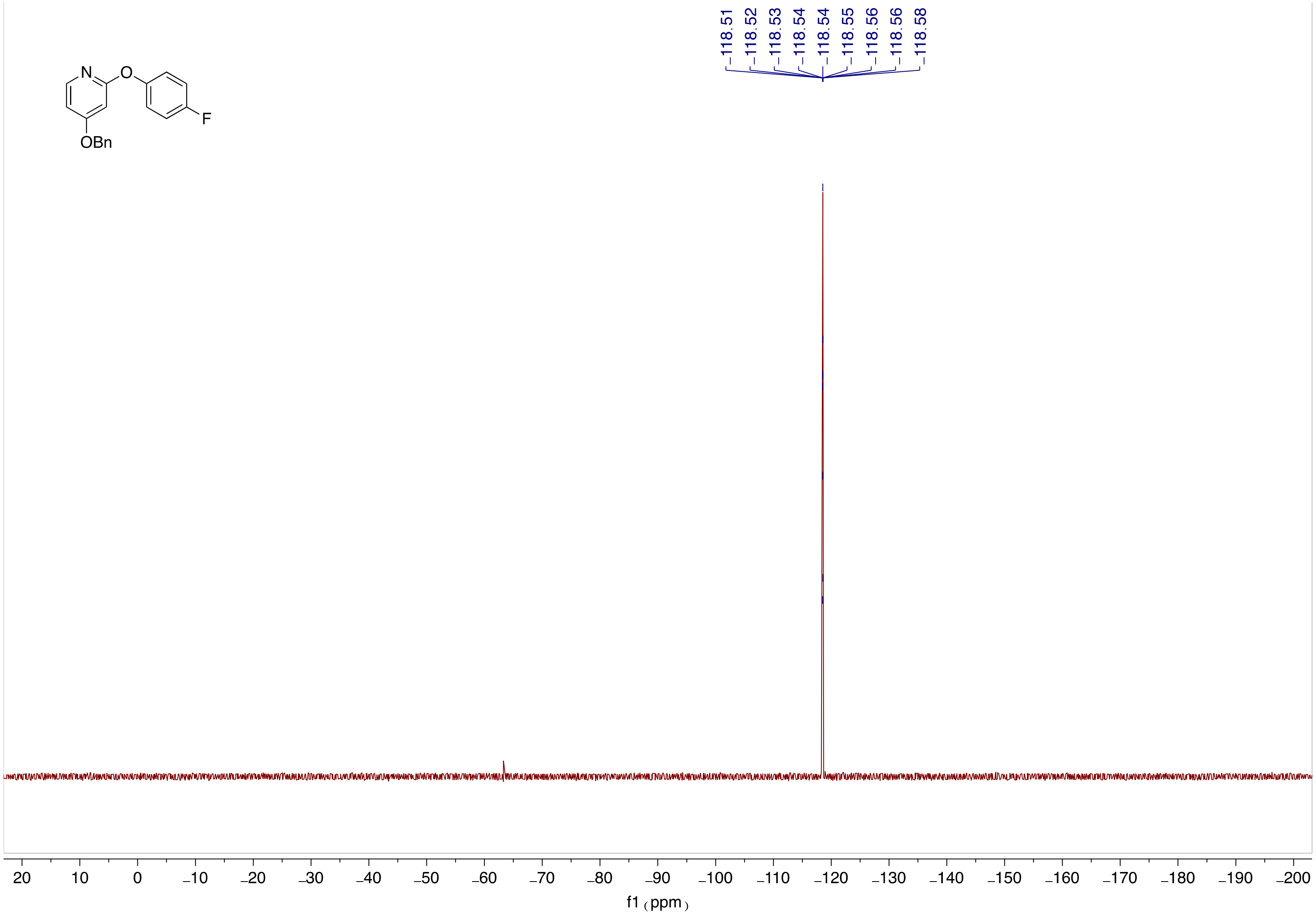

25 - ^{1}H NMR (400 MHz, $CDCl_3$):

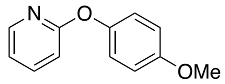

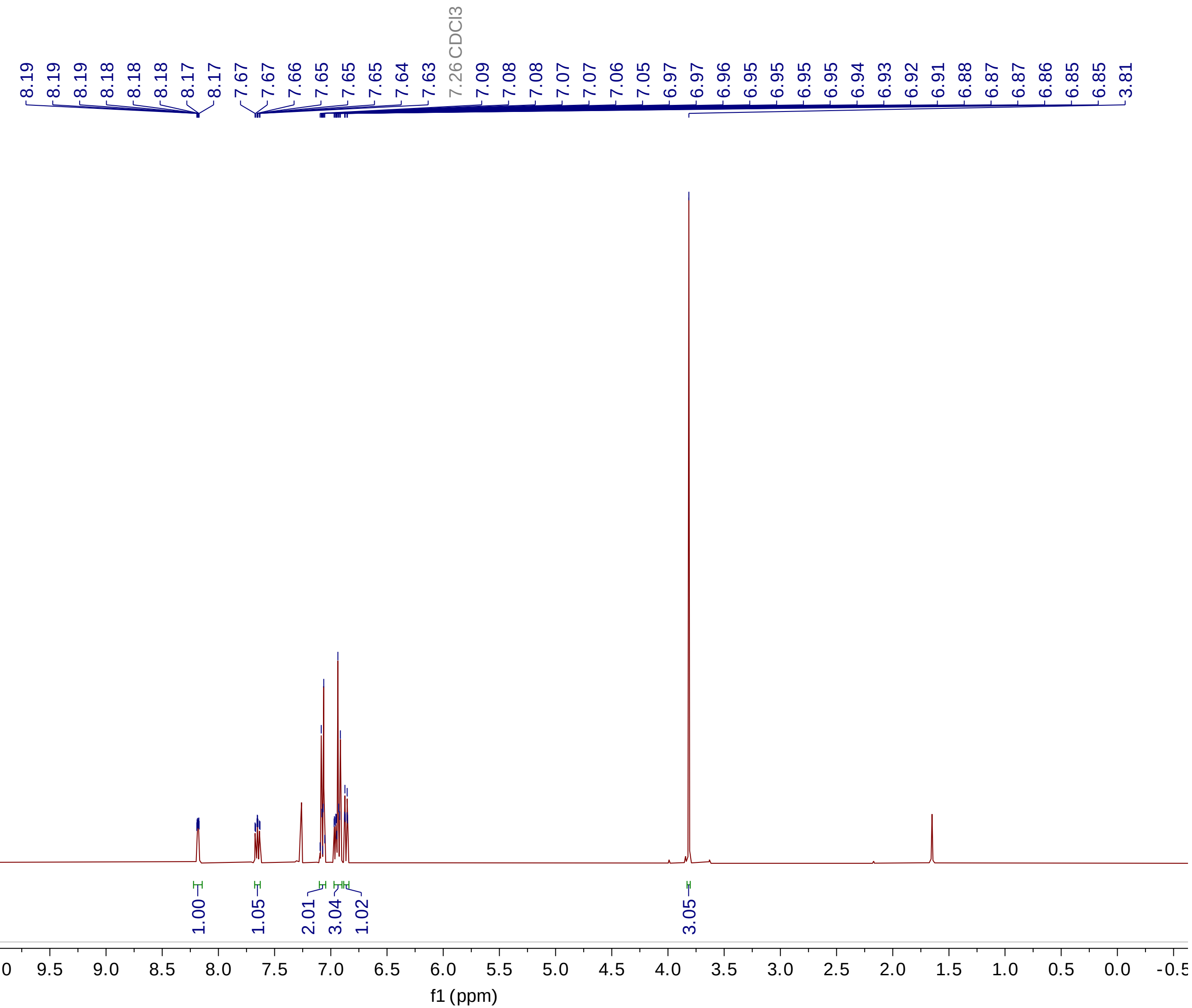

25 - $^{13}C\{^1H\}$ NMR (101 MHz, $CDCl_3$):

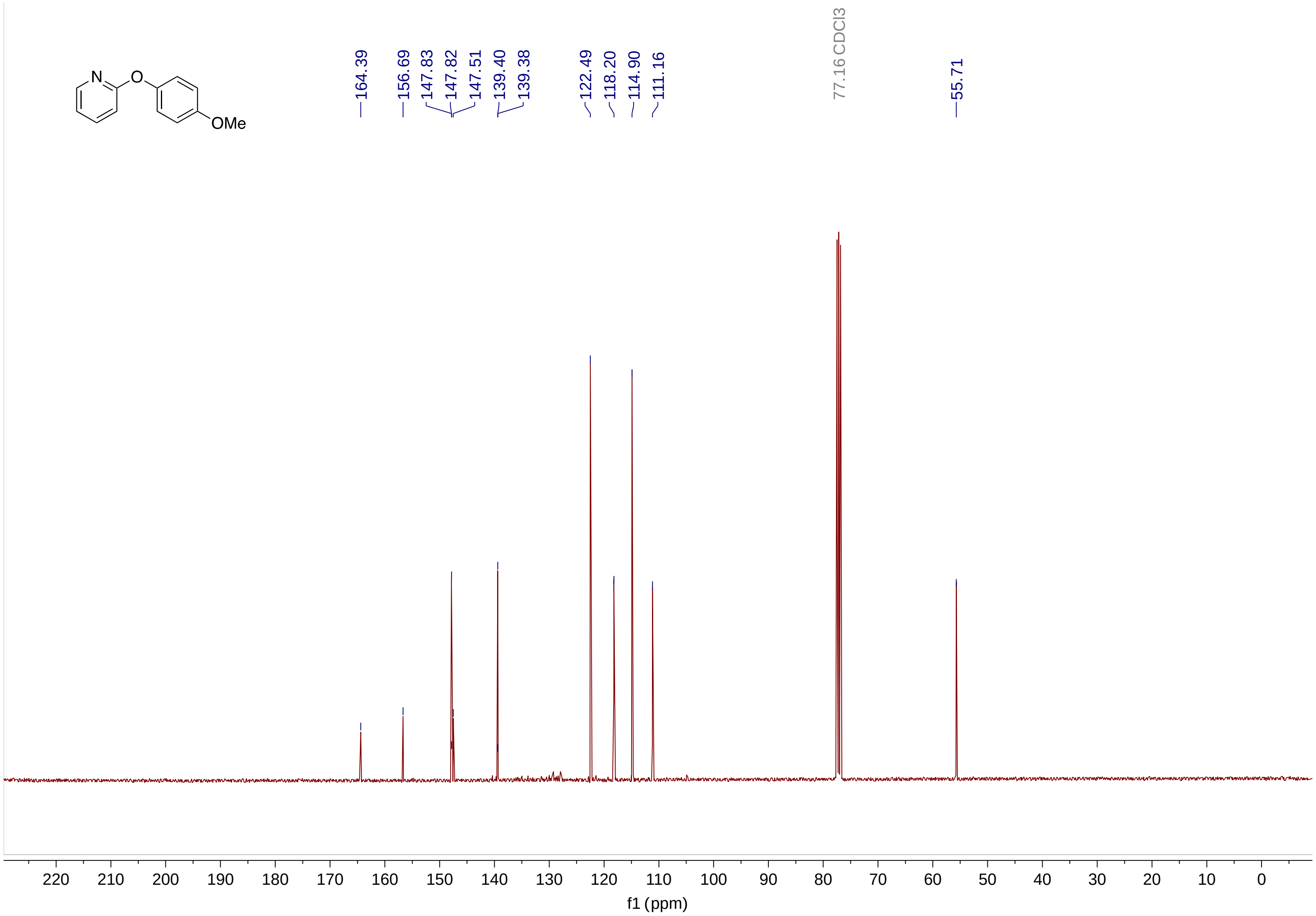

26 - ^{1}H NMR (400 MHz, $CDCl_3$):

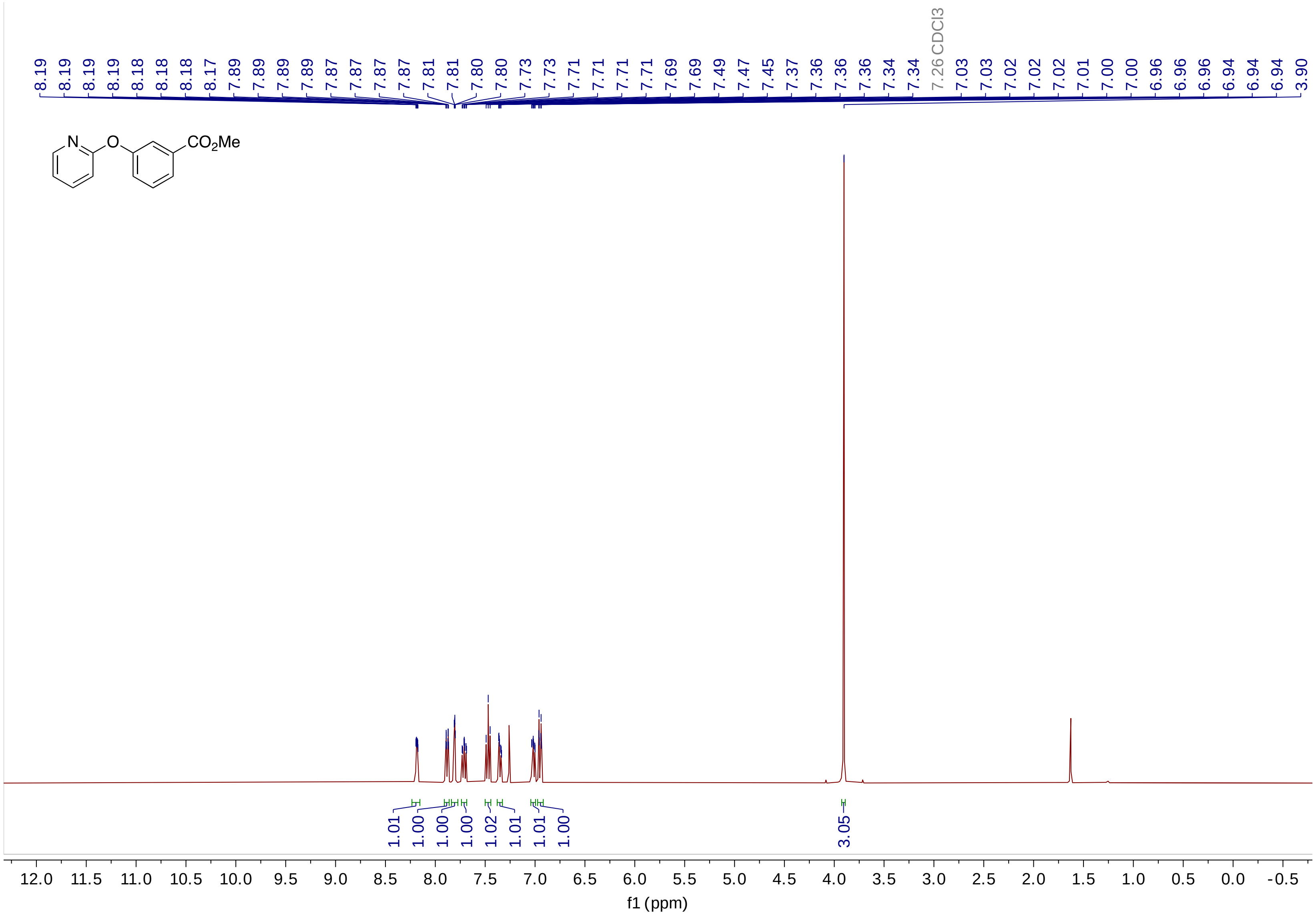

26 - $^{13}C\{^{1}H\}$ NMR (101 MHz, $CDCl_3$):

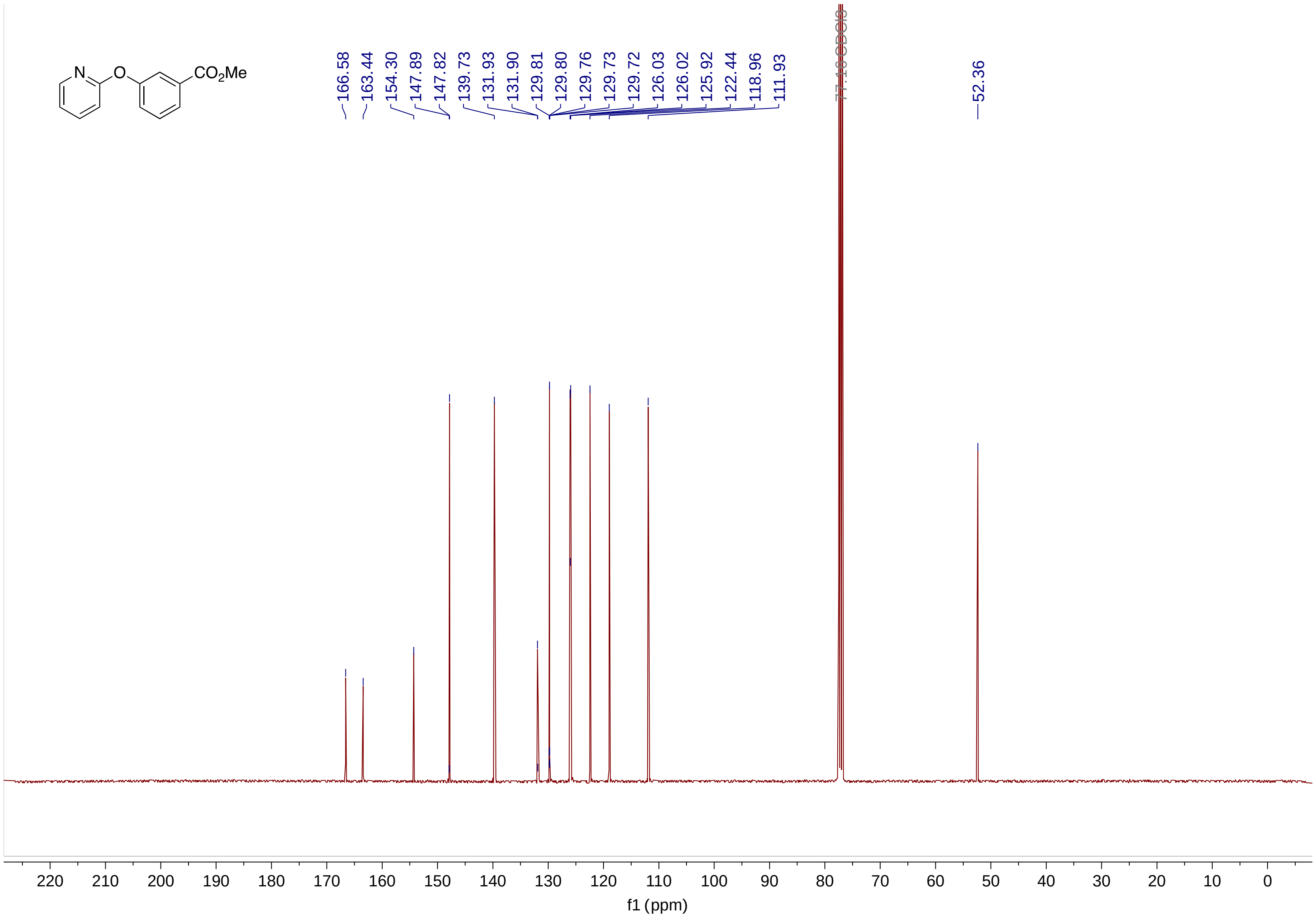

27 - ^{1}H NMR (400 MHz, $CDCl_3$):

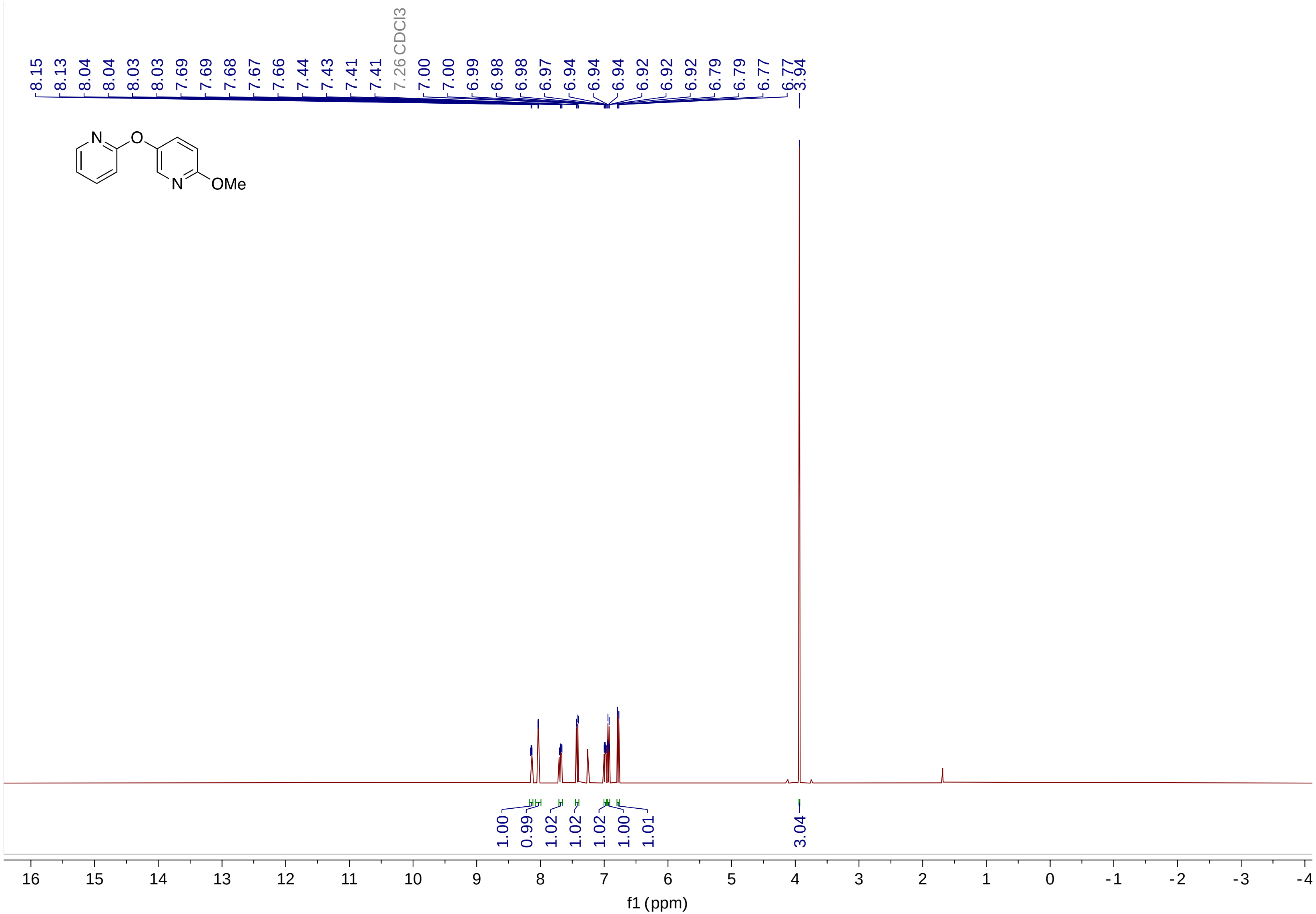

27 - $^{13}C\{^1H\}$ NMR (101 MHz, $CDCl_3$):

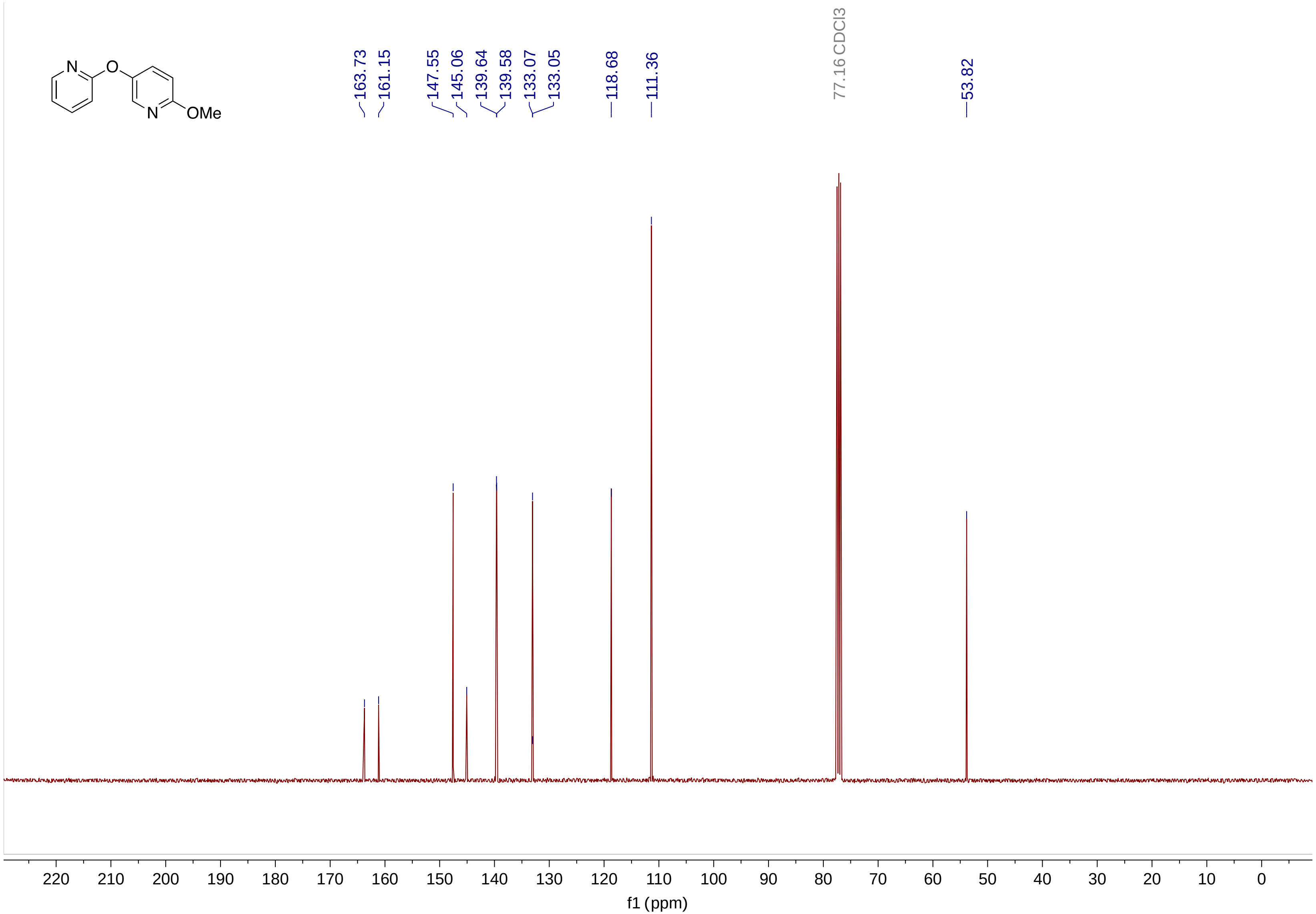

28 - ^{1}H NMR (400 MHz, $CDCl_3$):

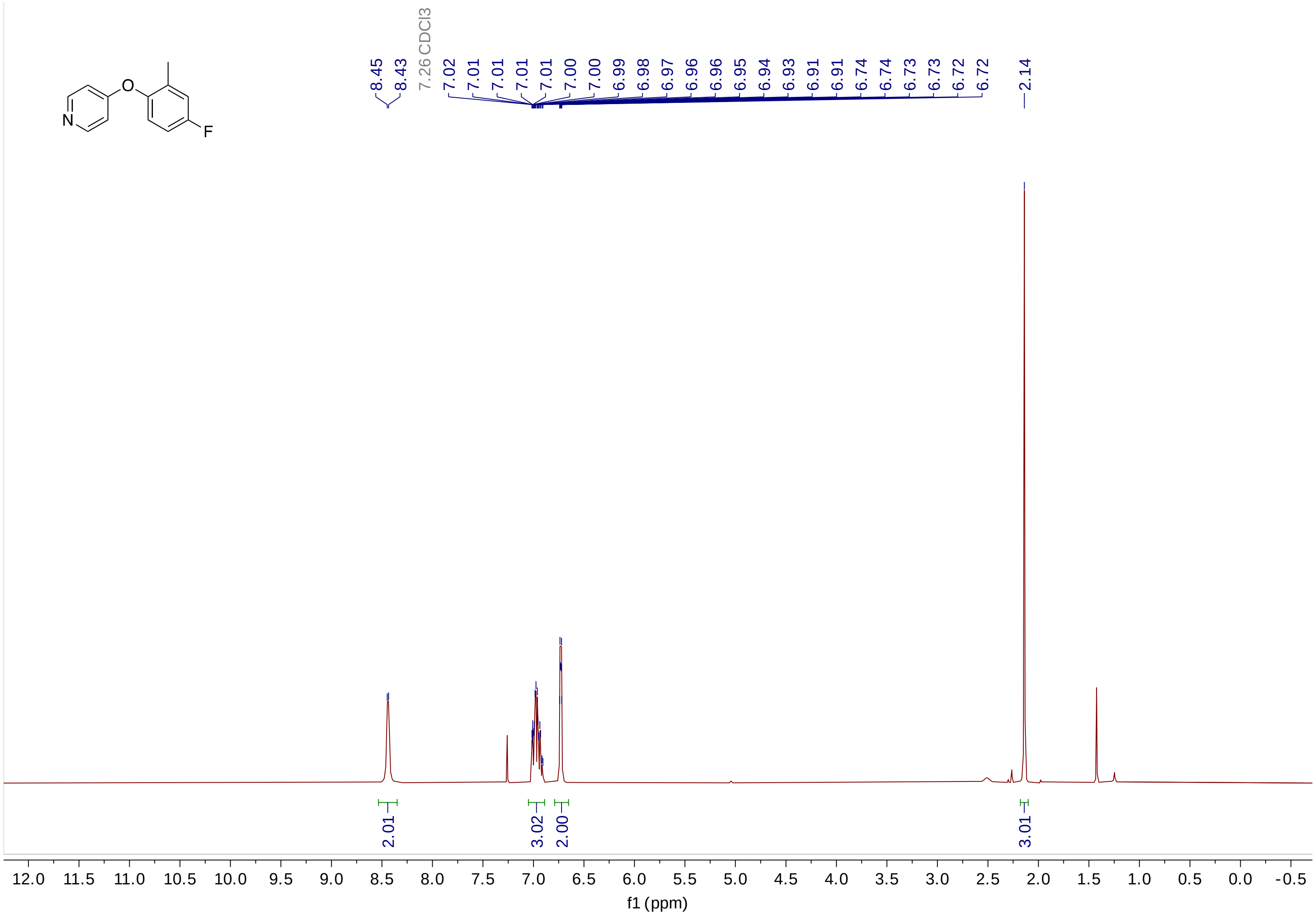

28 - $^{13}C\{^{1}H\}$ NMR (101 MHz, $CDCl_3$):

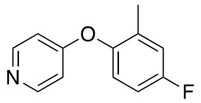

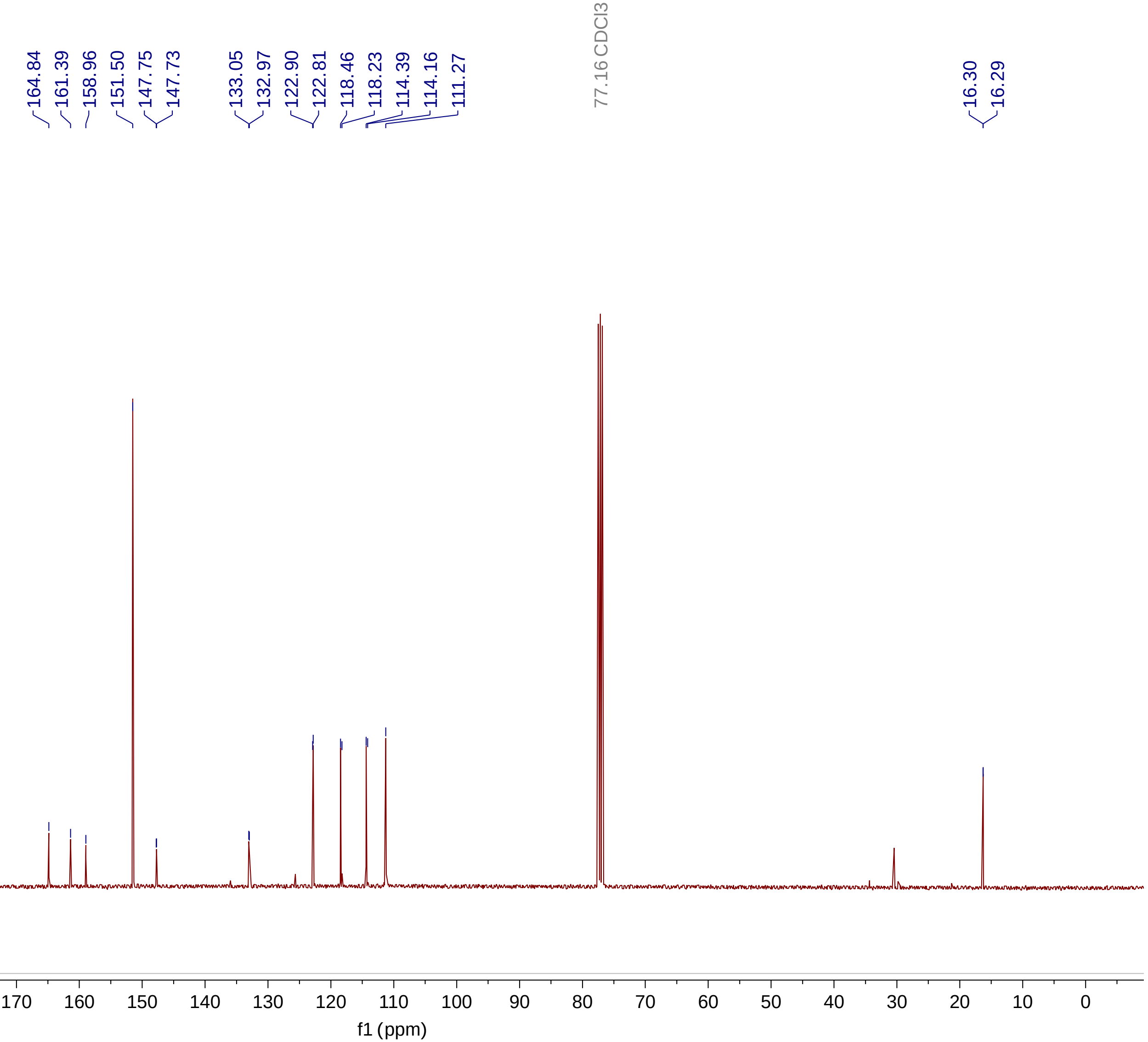

28 - ^{19}F NMR (376 MHz, $CDCl_3$):

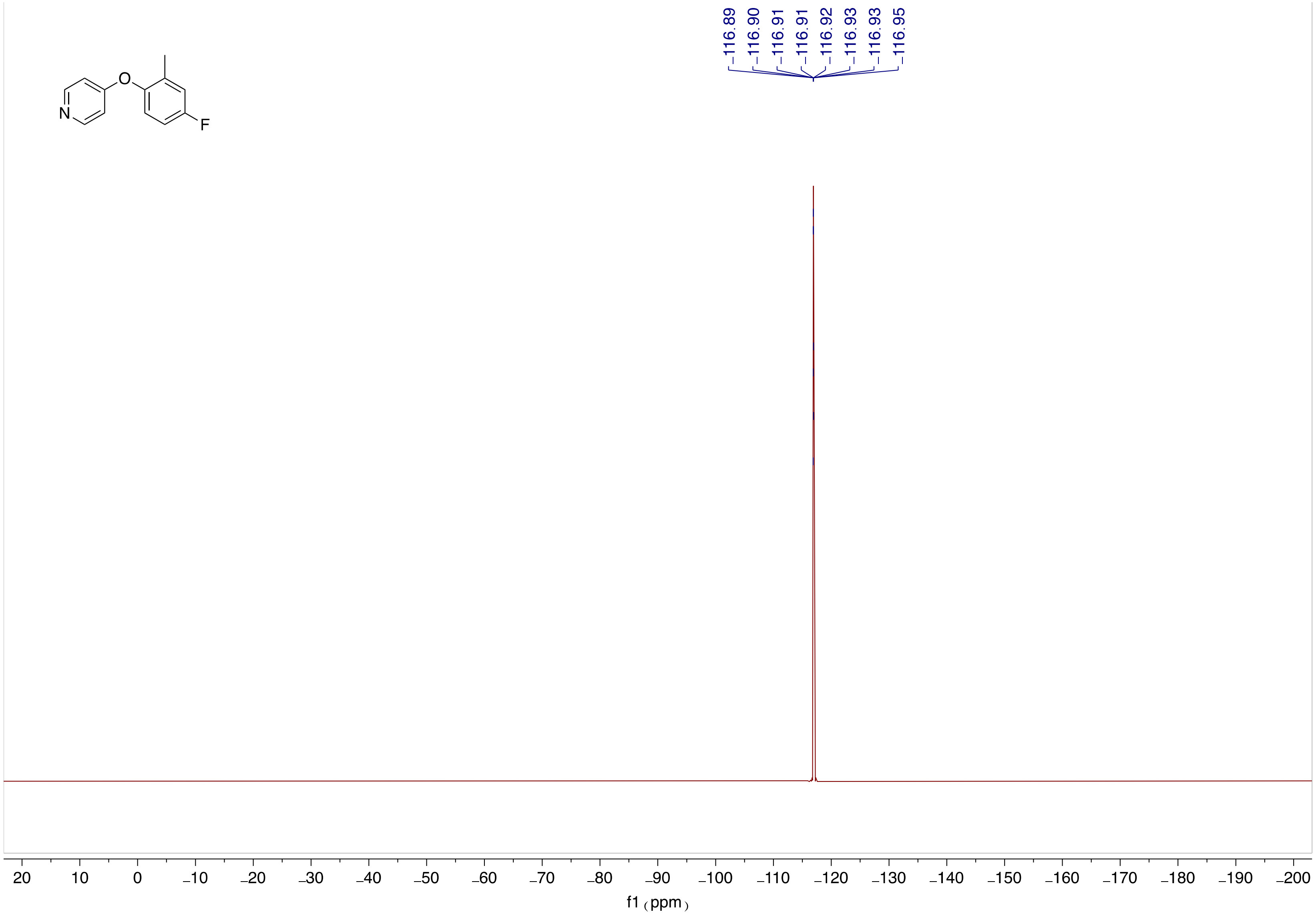

29 - ^{1}H NMR (400 MHz, CDCl$_3$):

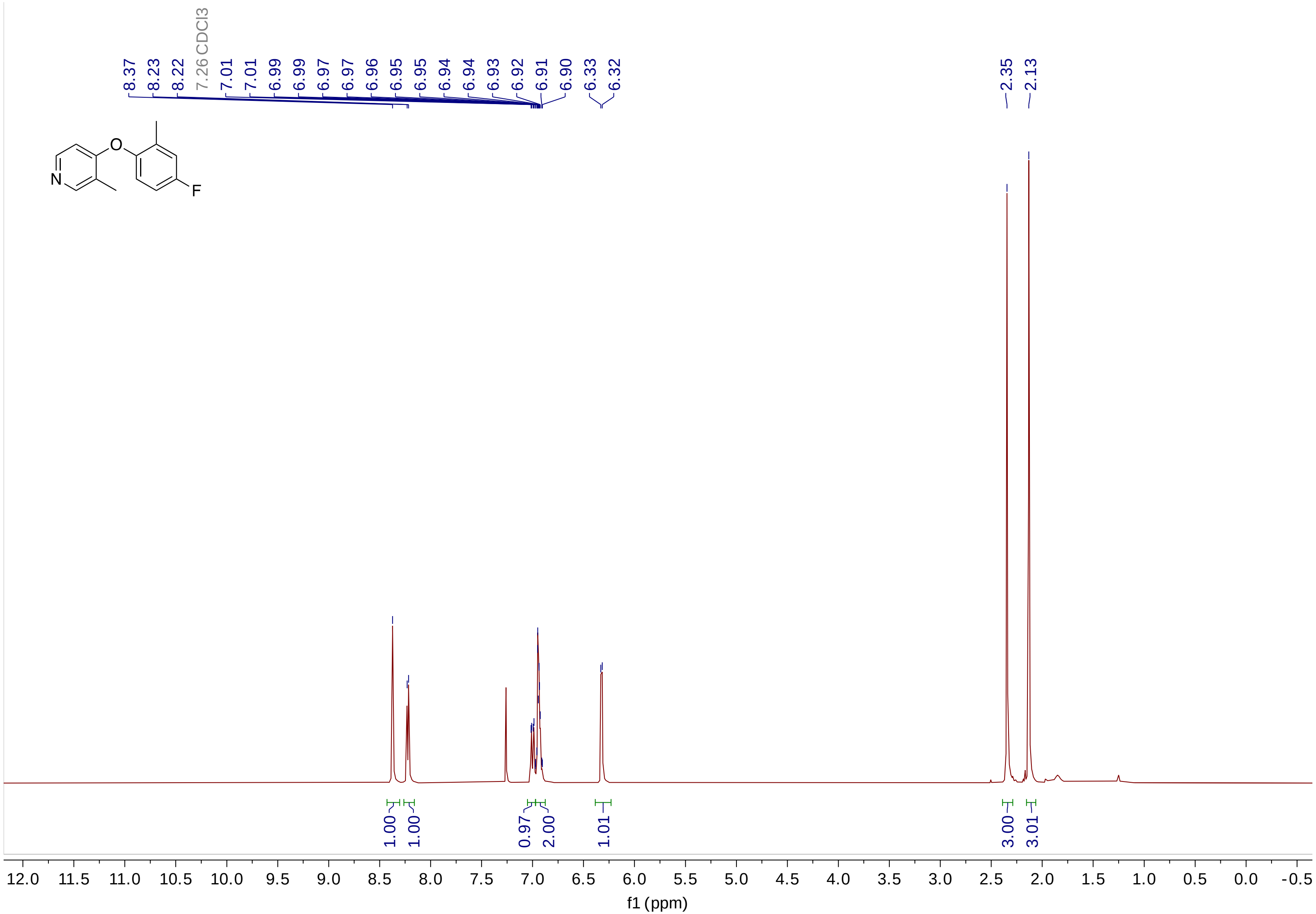

29 - $^{13}C\{^1H\}$ NMR (101 MHz, $CDCl_3$):

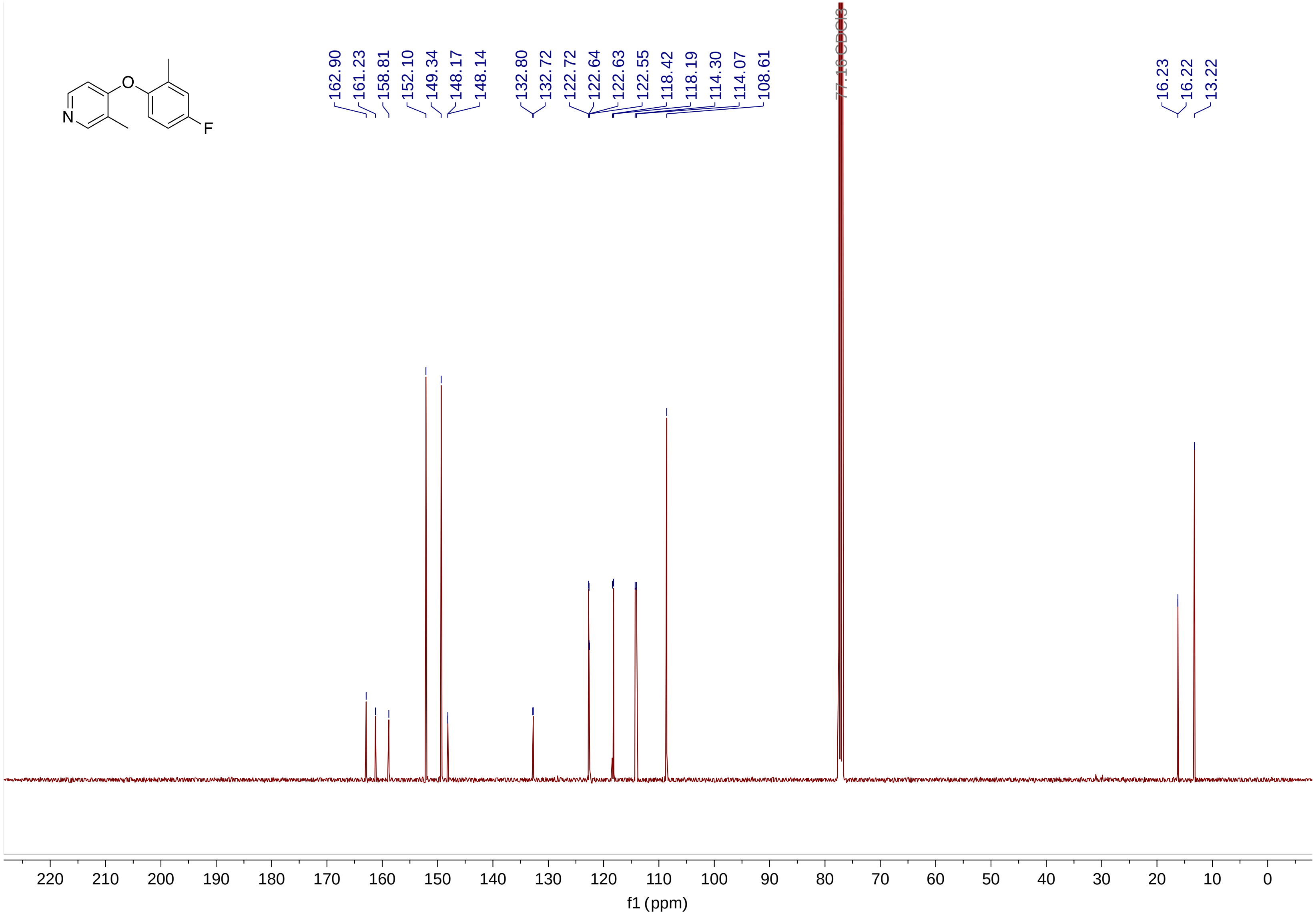

29 - ^{19}F NMR (377 MHz, CDCl$_3$):

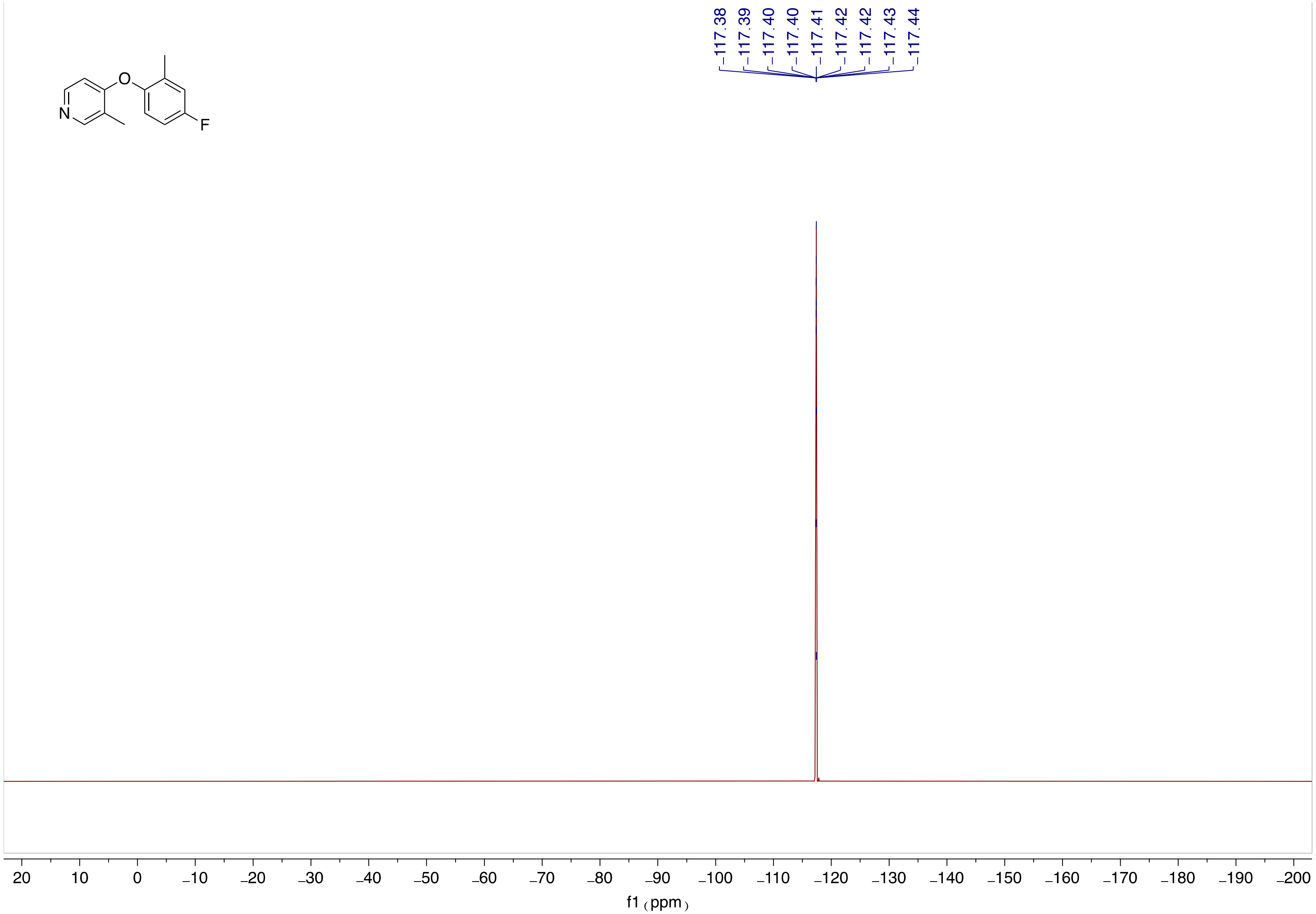

30 - ^{1}H NMR (400 MHz, $CDCl_3$):

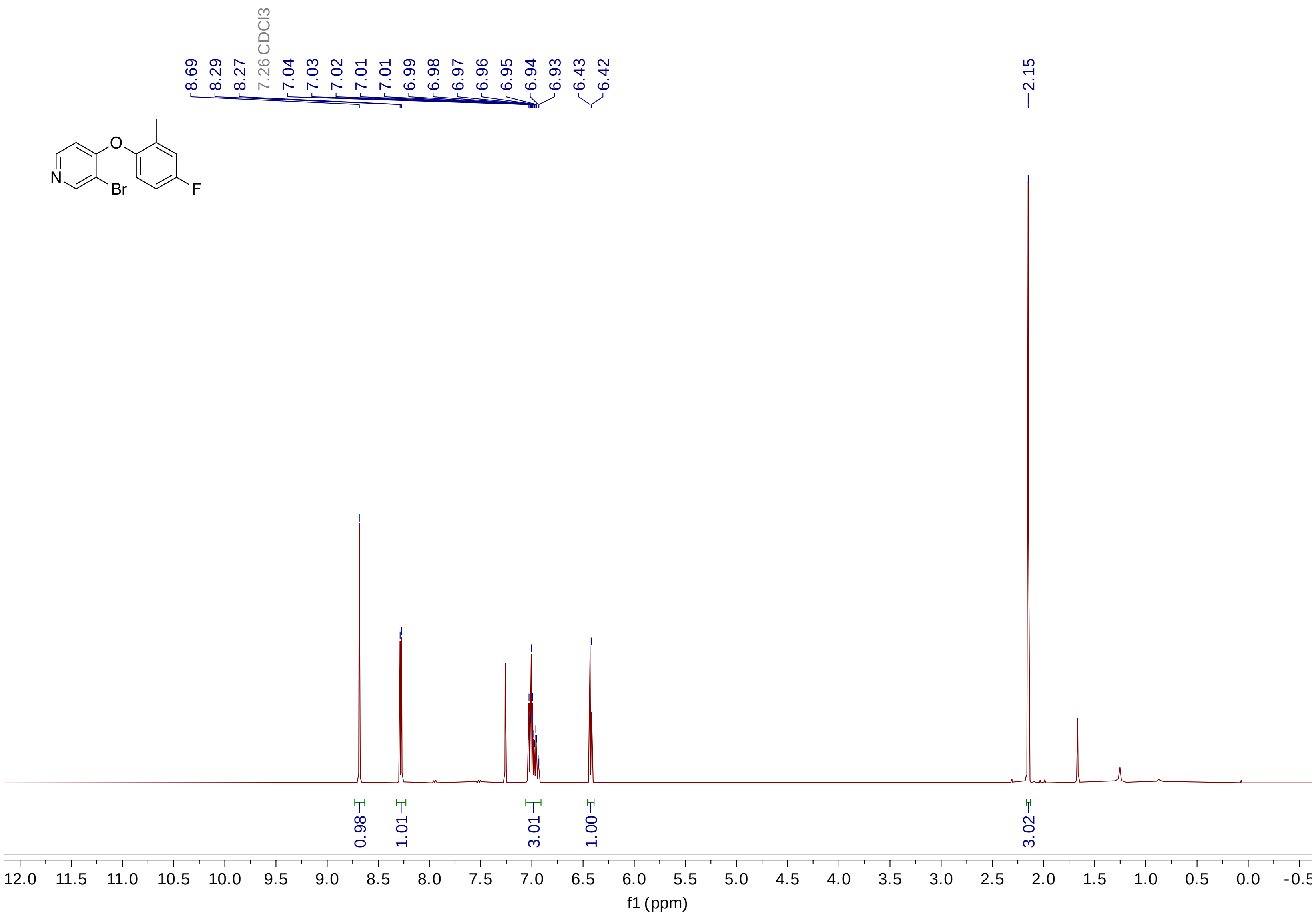

30 - $^{13}C\{^{1}H\}$ NMR (101 MHz, $CDCl_3$):

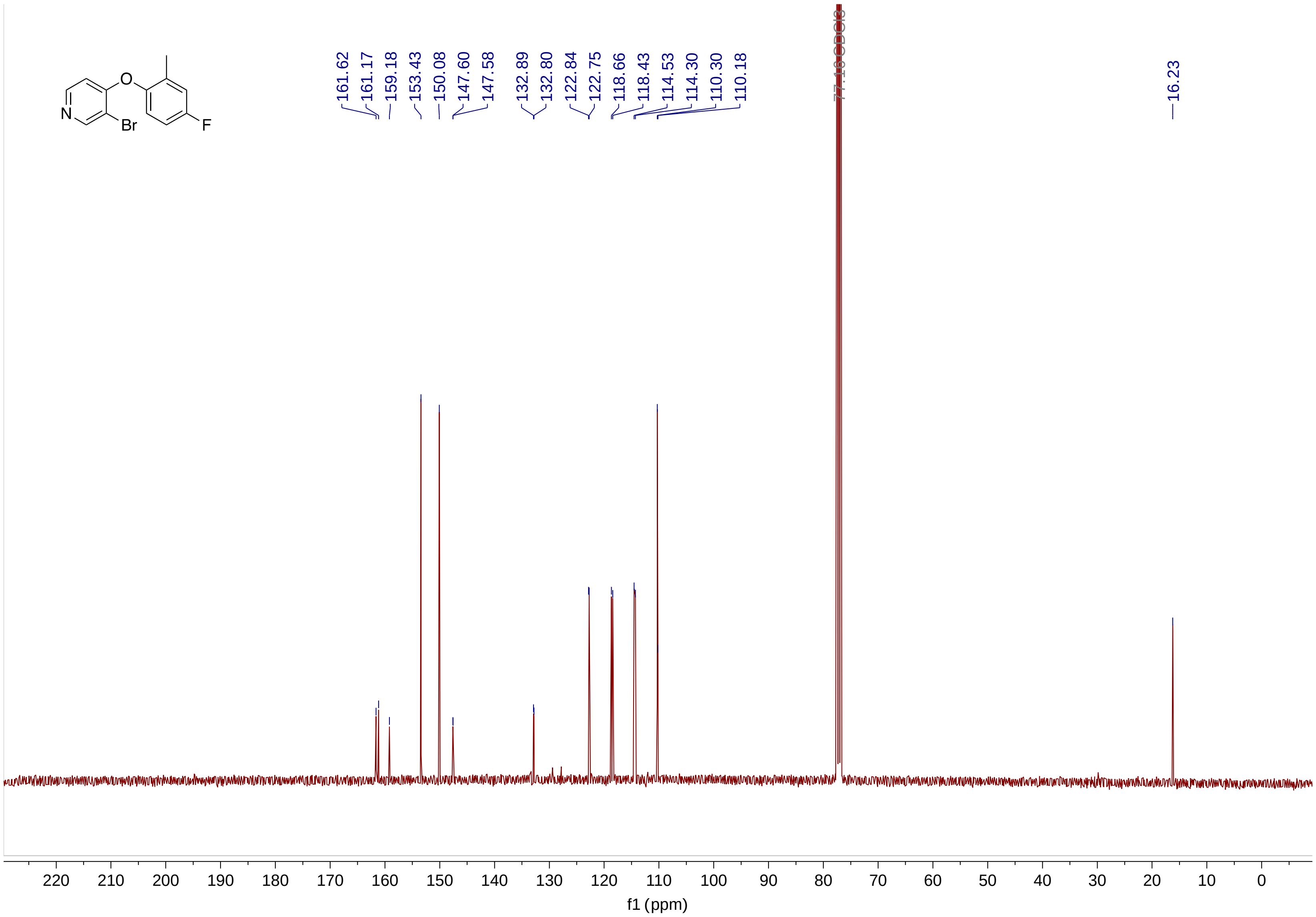

30 - ^{19}F NMR (376 MHz, $CDCl_3$):

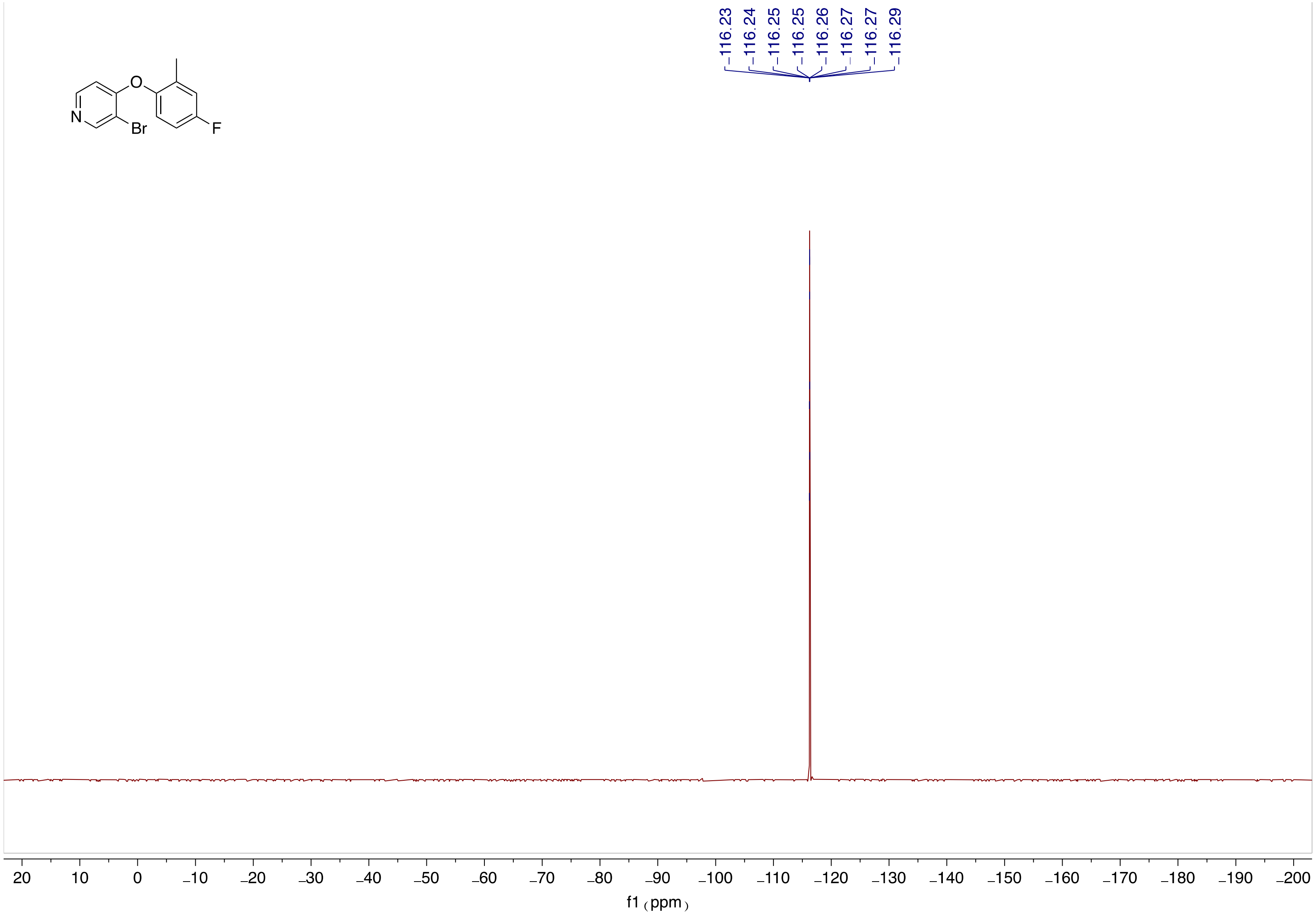

31 - ^{1}H NMR (400 MHz, $CDCl_3$):

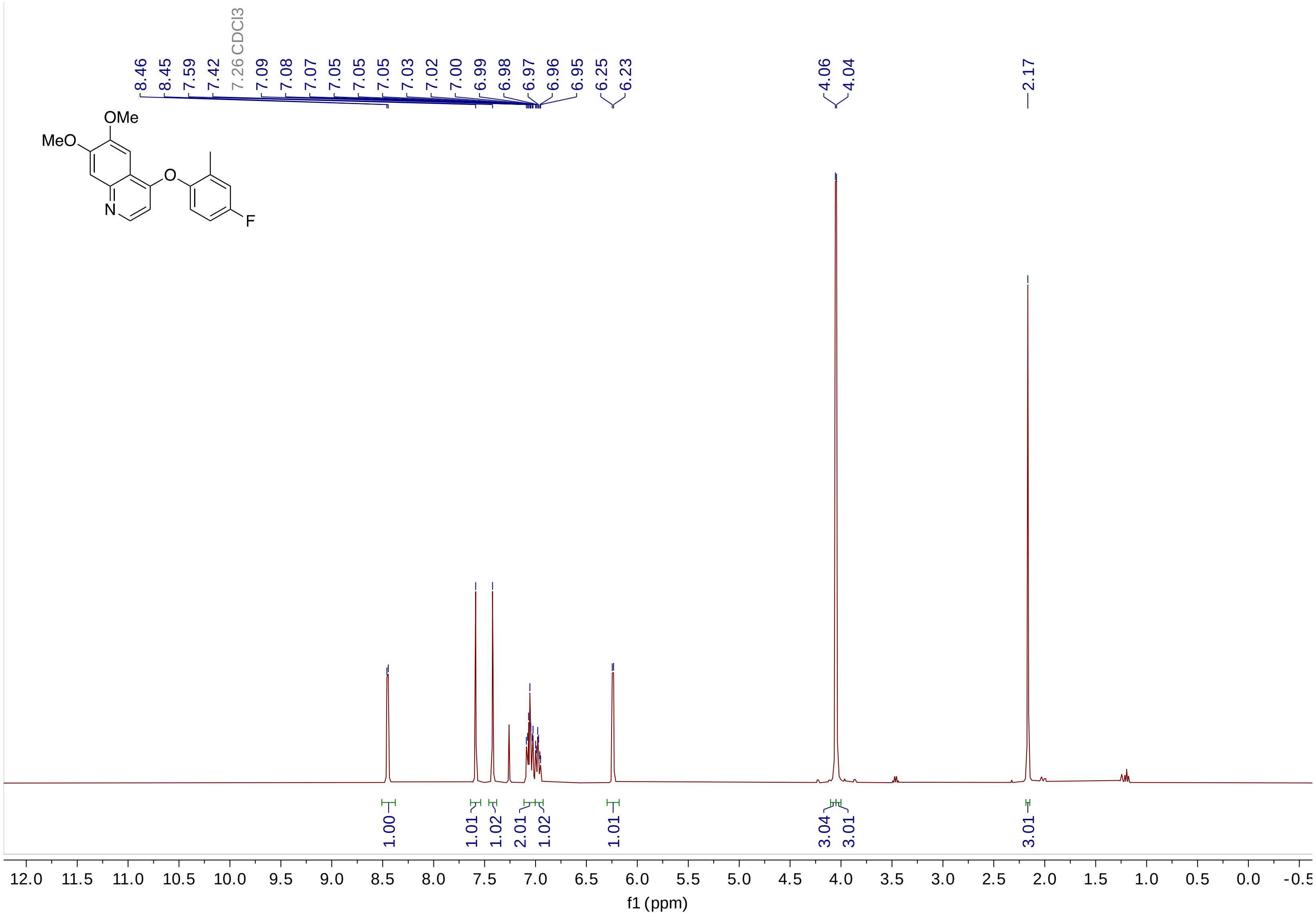

31 - $^{13}C\{^{1}H\}$ NMR (101 MHz, $CDCl_3$):

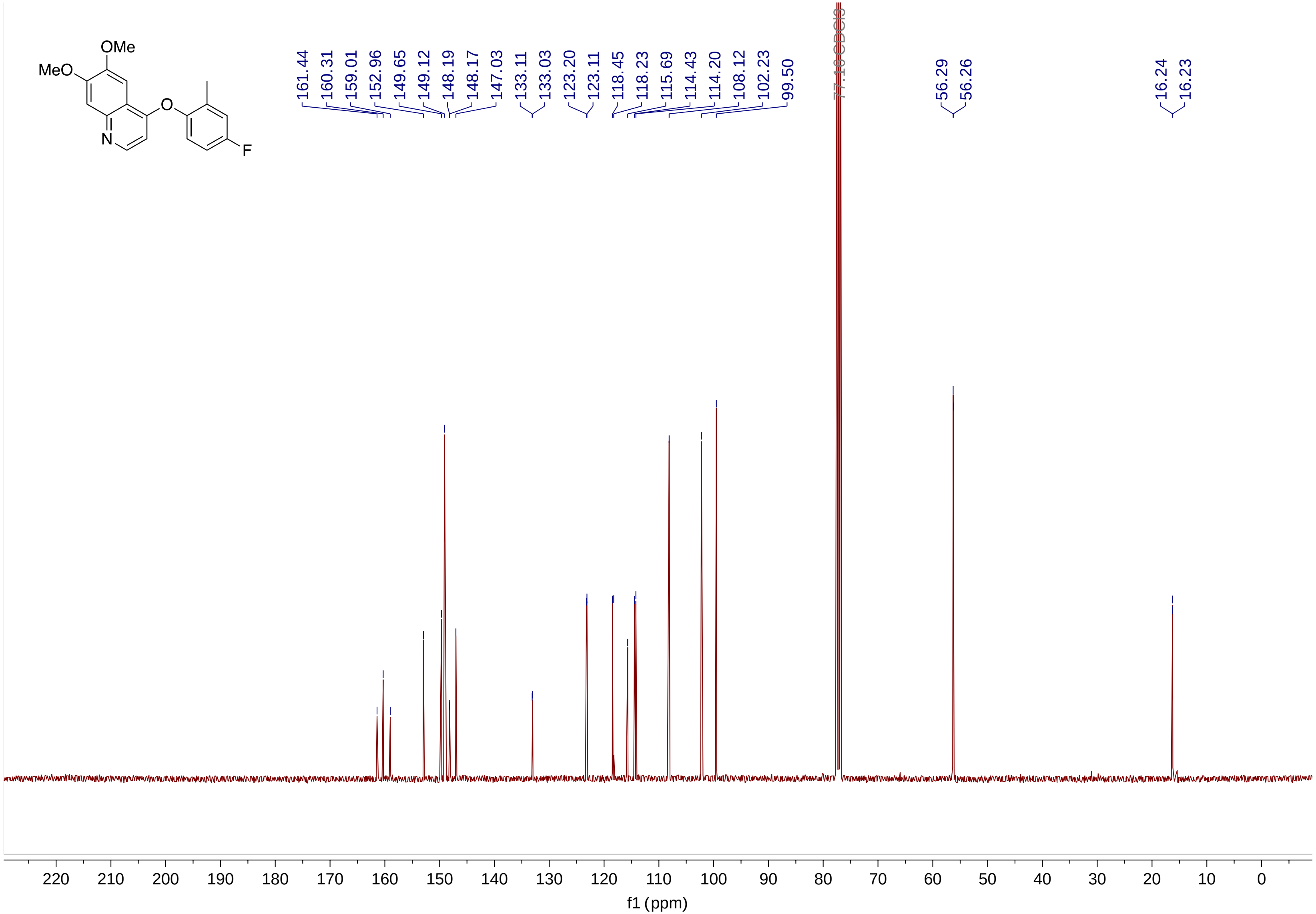

31 - ^{19}F NMR (376 MHz, $CDCl_3$):

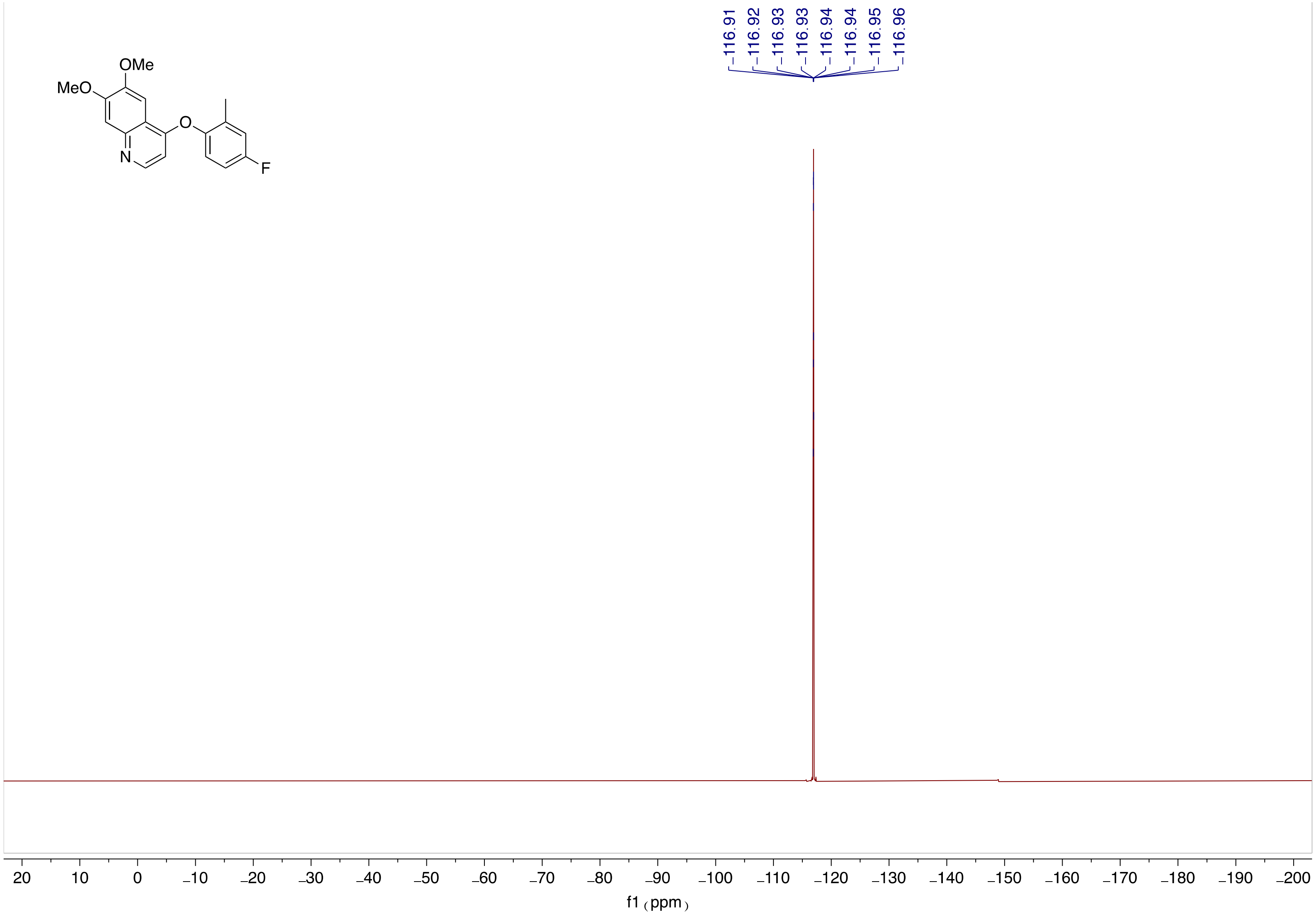

32 - ^{1}H NMR (400 MHz, CDCl$_3$):

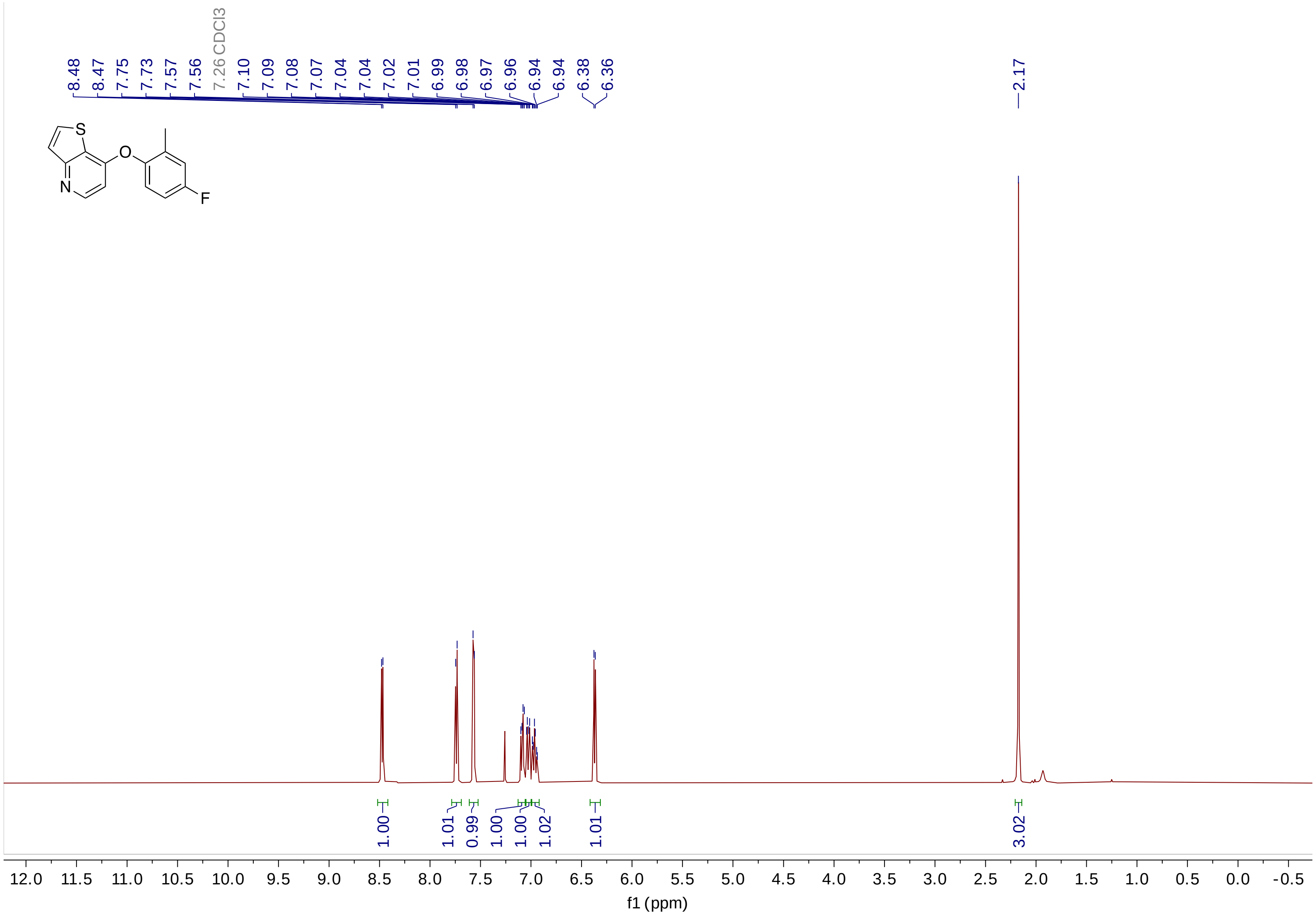

32 - $^{13}C\{^{1}H\}$ NMR (101 MHz, $CDCl_3$):

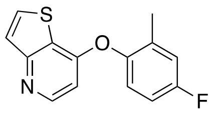

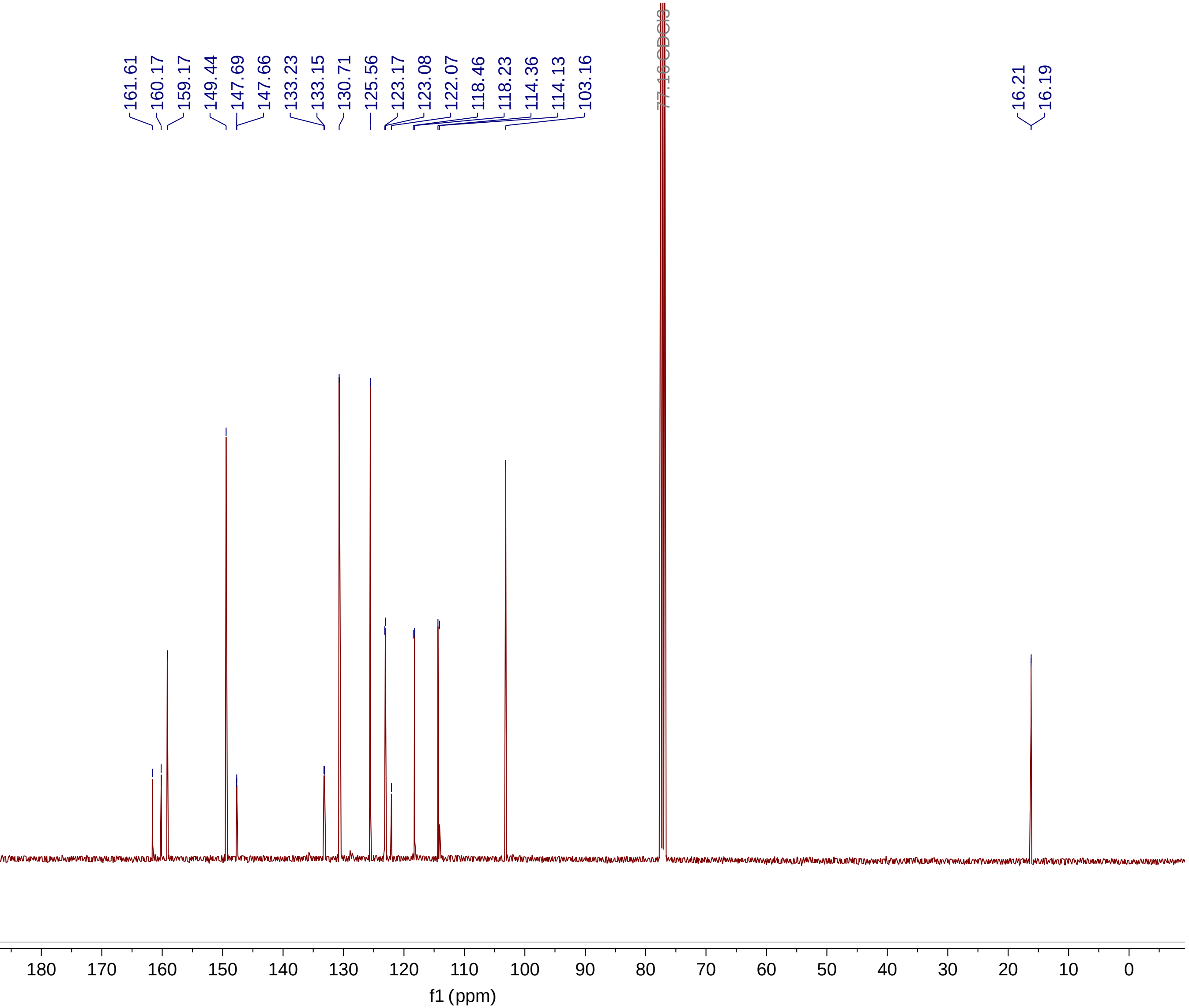

32 - ^{19}F NMR (376 MHz, $CDCl_3$):

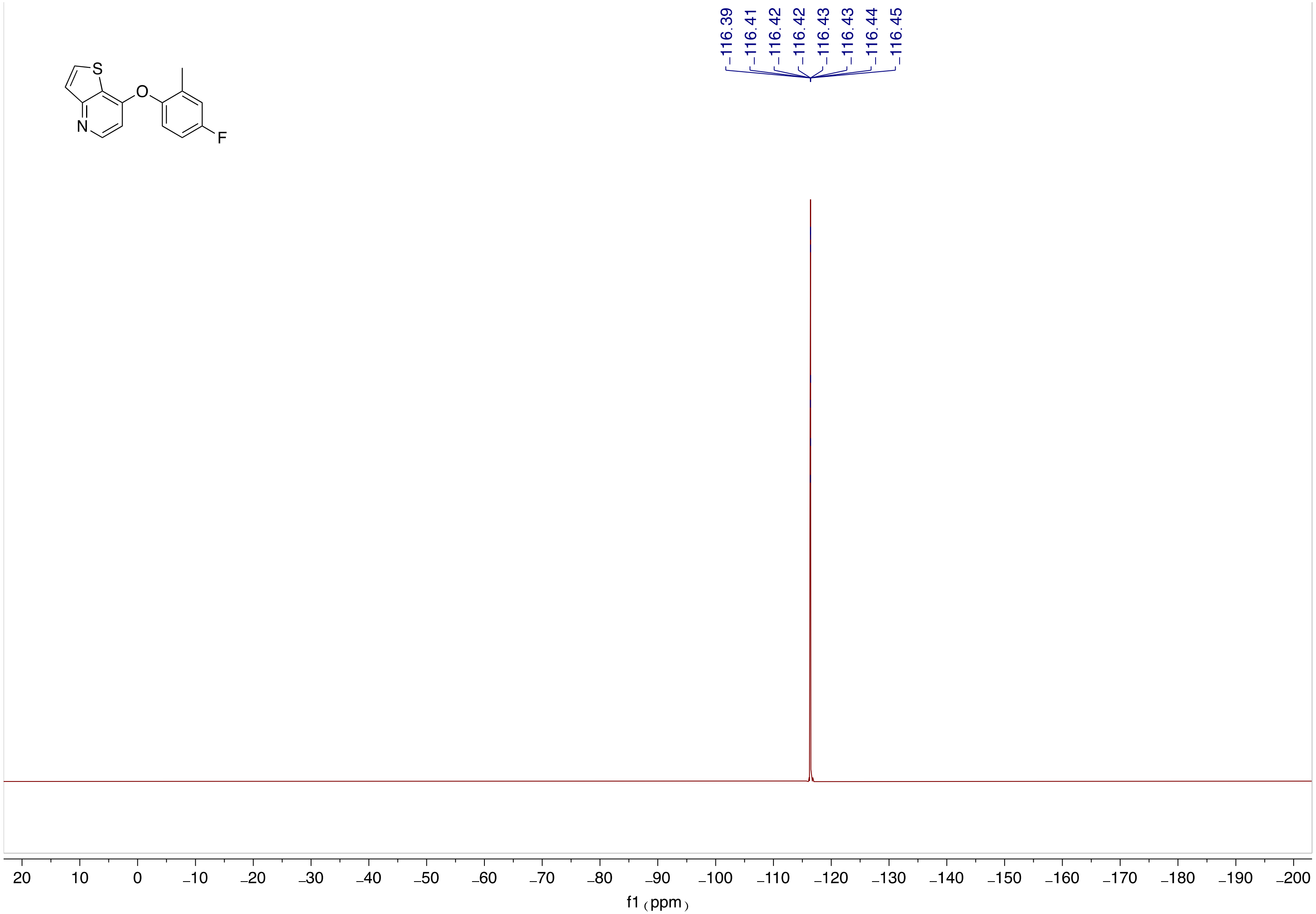

33 - ^{1}H NMR (400 MHz, $CDCl_3$):

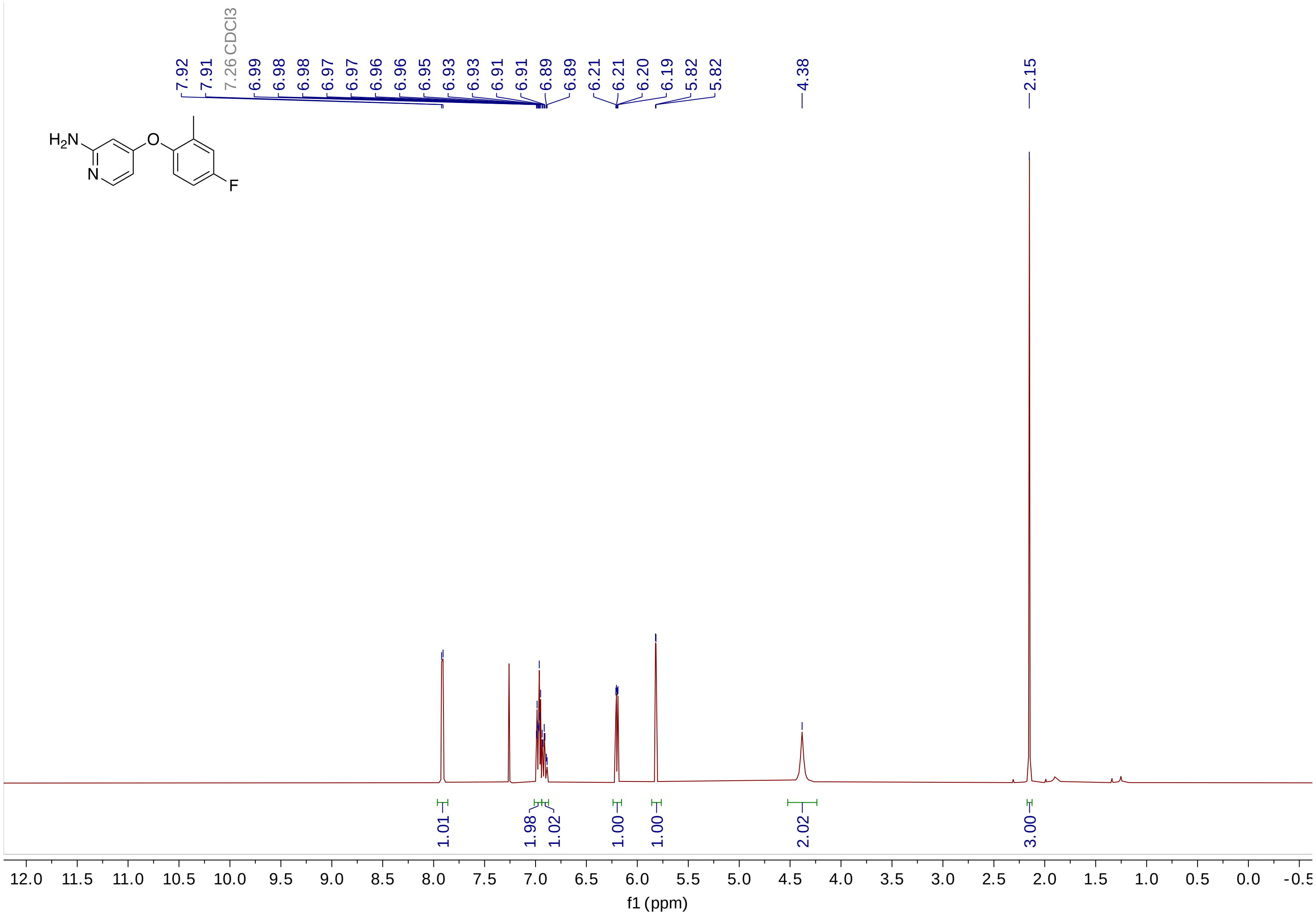

33 - $^{13}C\{^1H\}$ NMR (101 MHz, $CDCl_3$):

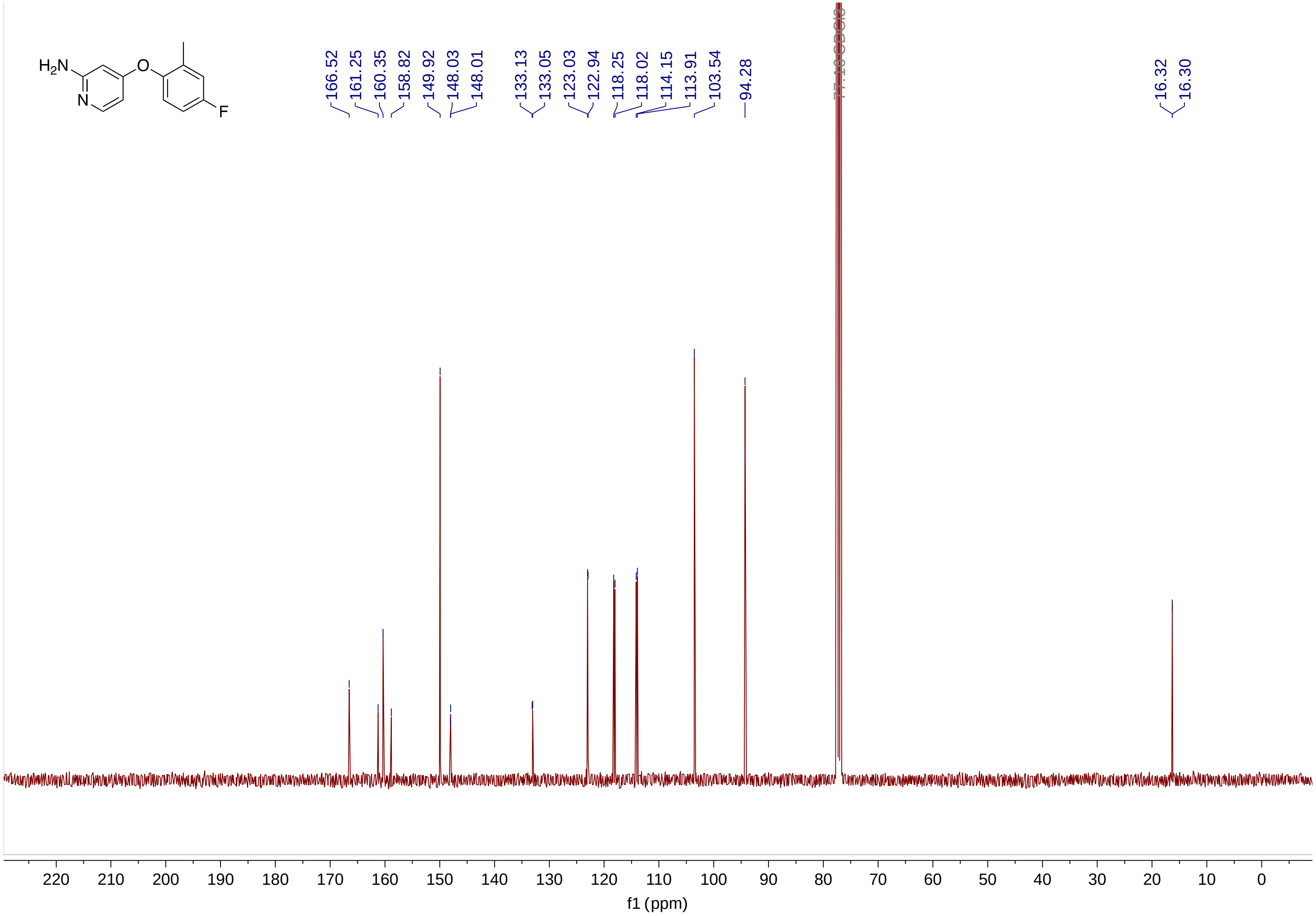

33 - ^{19}F NMR (376 MHz, $CDCl_3$):

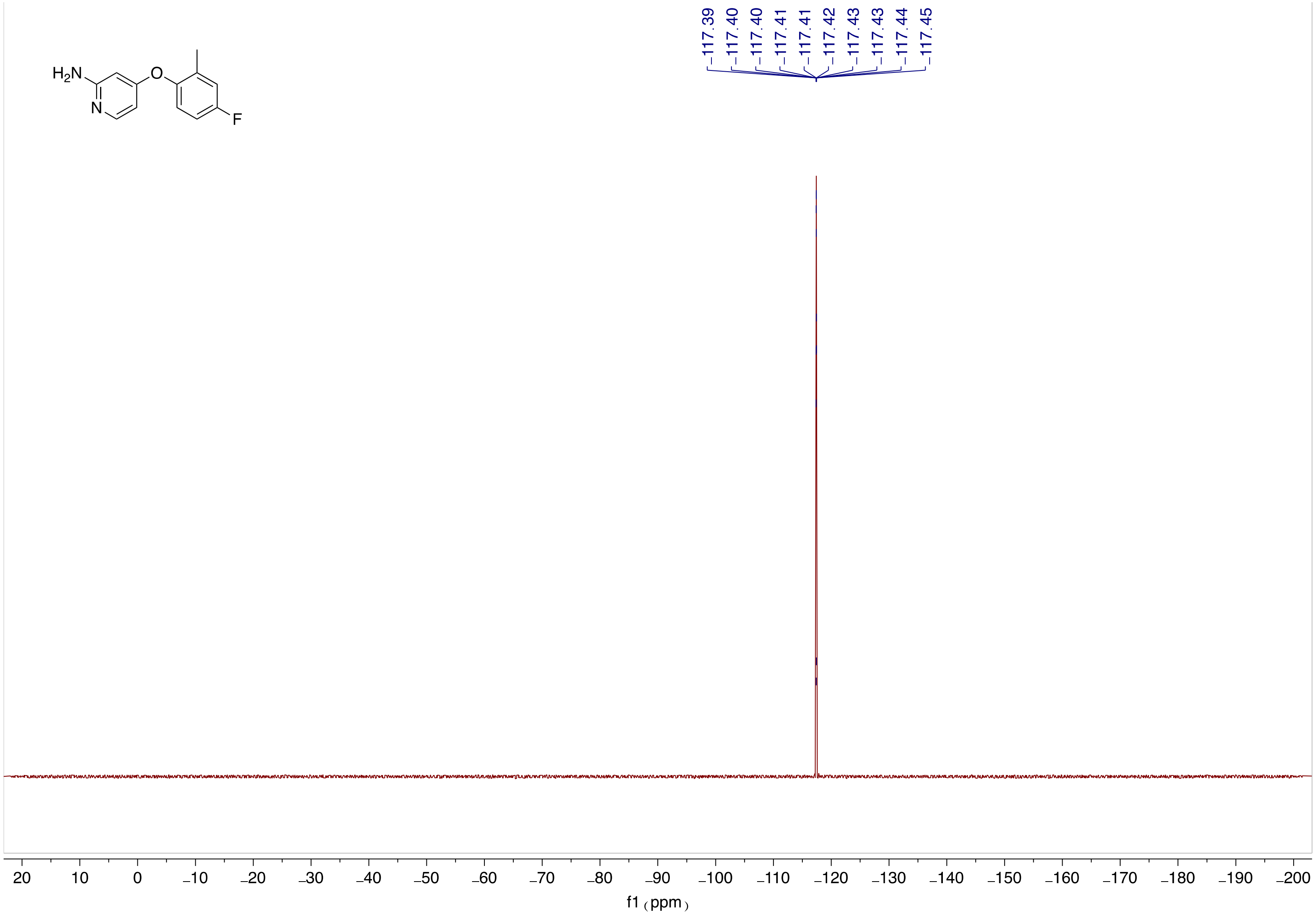

34 - ^{1}H NMR (400 MHz, $CDCl_3$):

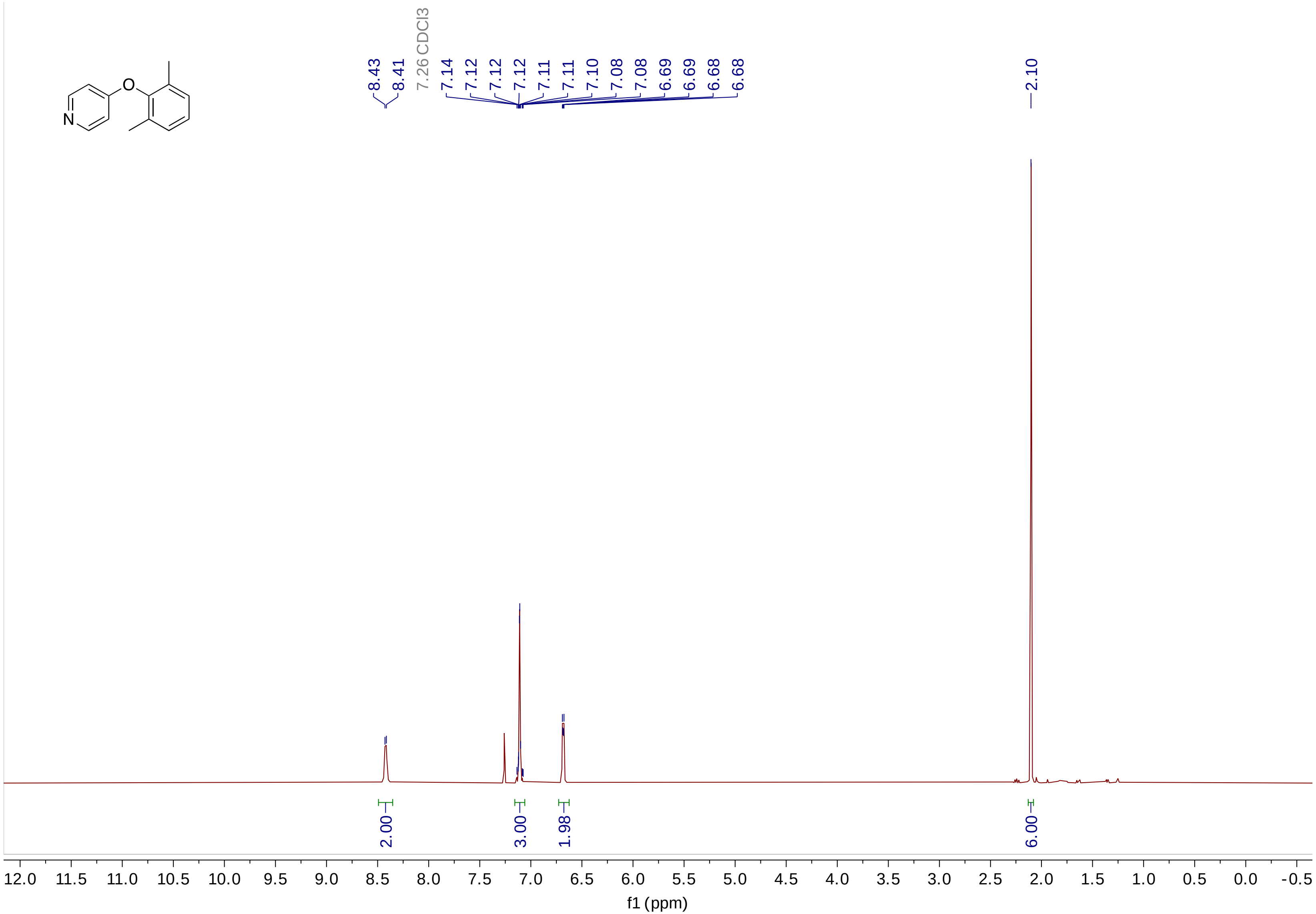

34 - $^{13}C\{^1H\}$ NMR (101 MHz, $CDCl_3$):

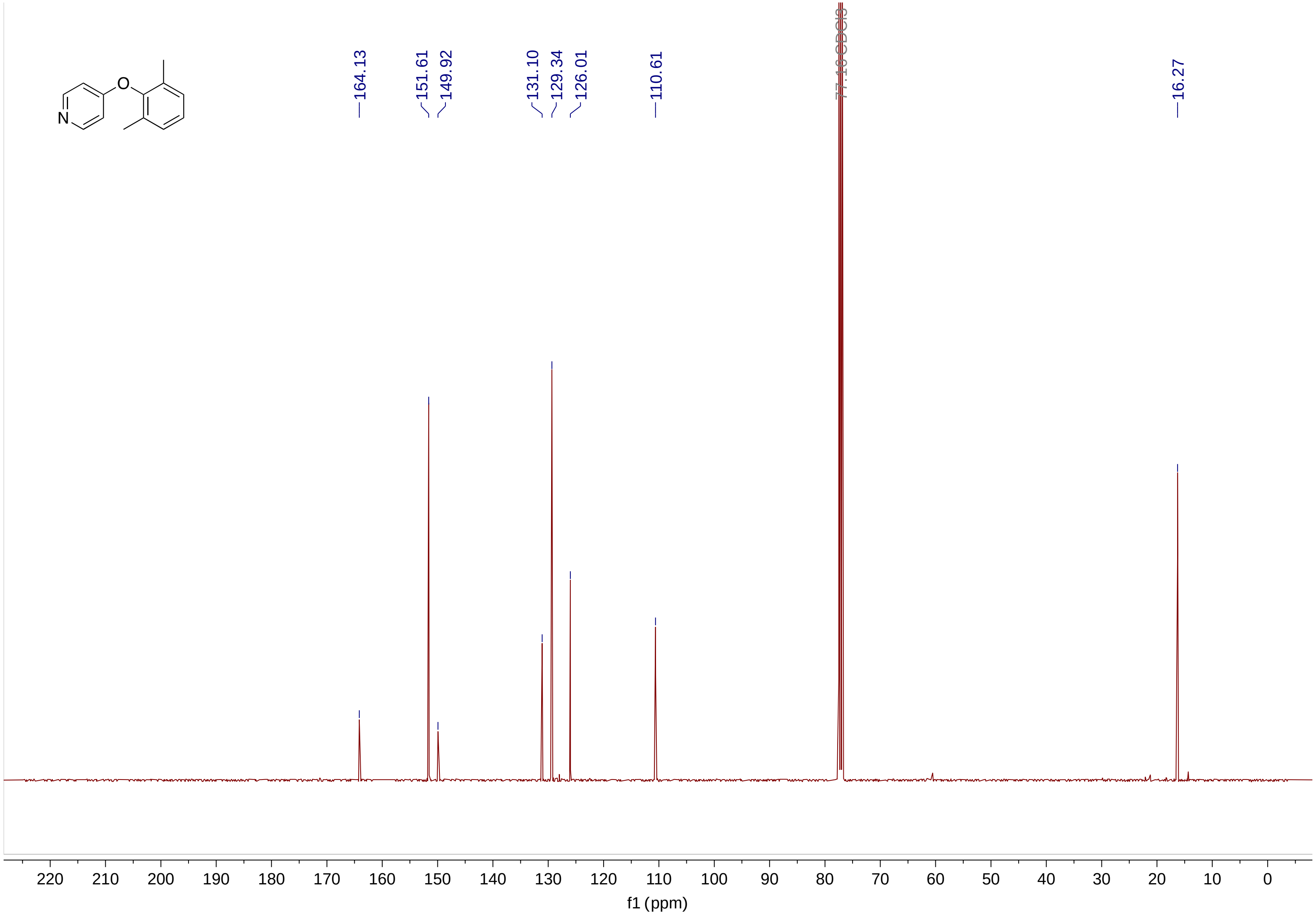

35 - ^{1}H NMR (400 MHz, CDCl$_3$):

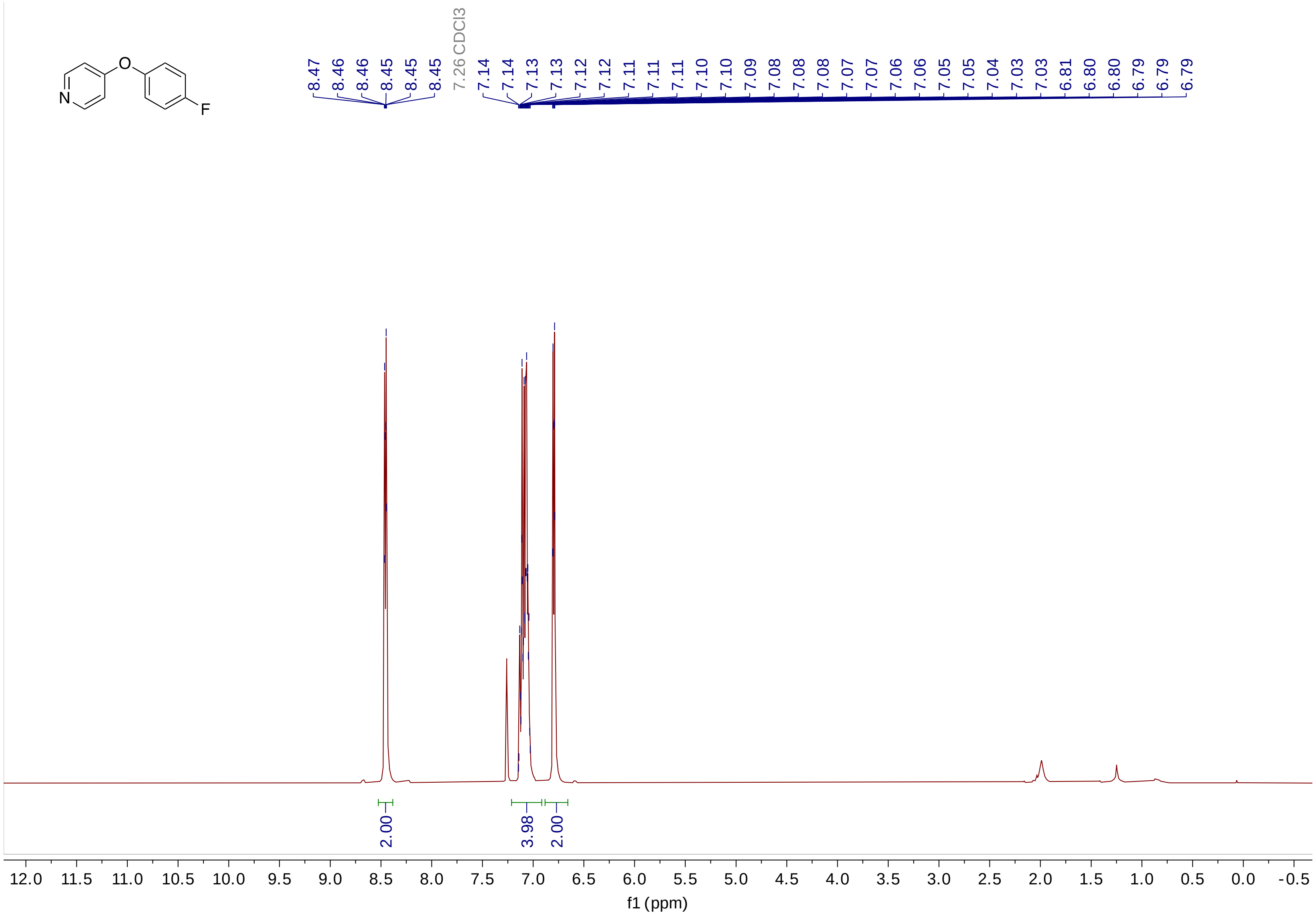

35 - $^{13}C\{^{1}H\}$ NMR (101 MHz, $CDCl_3$):

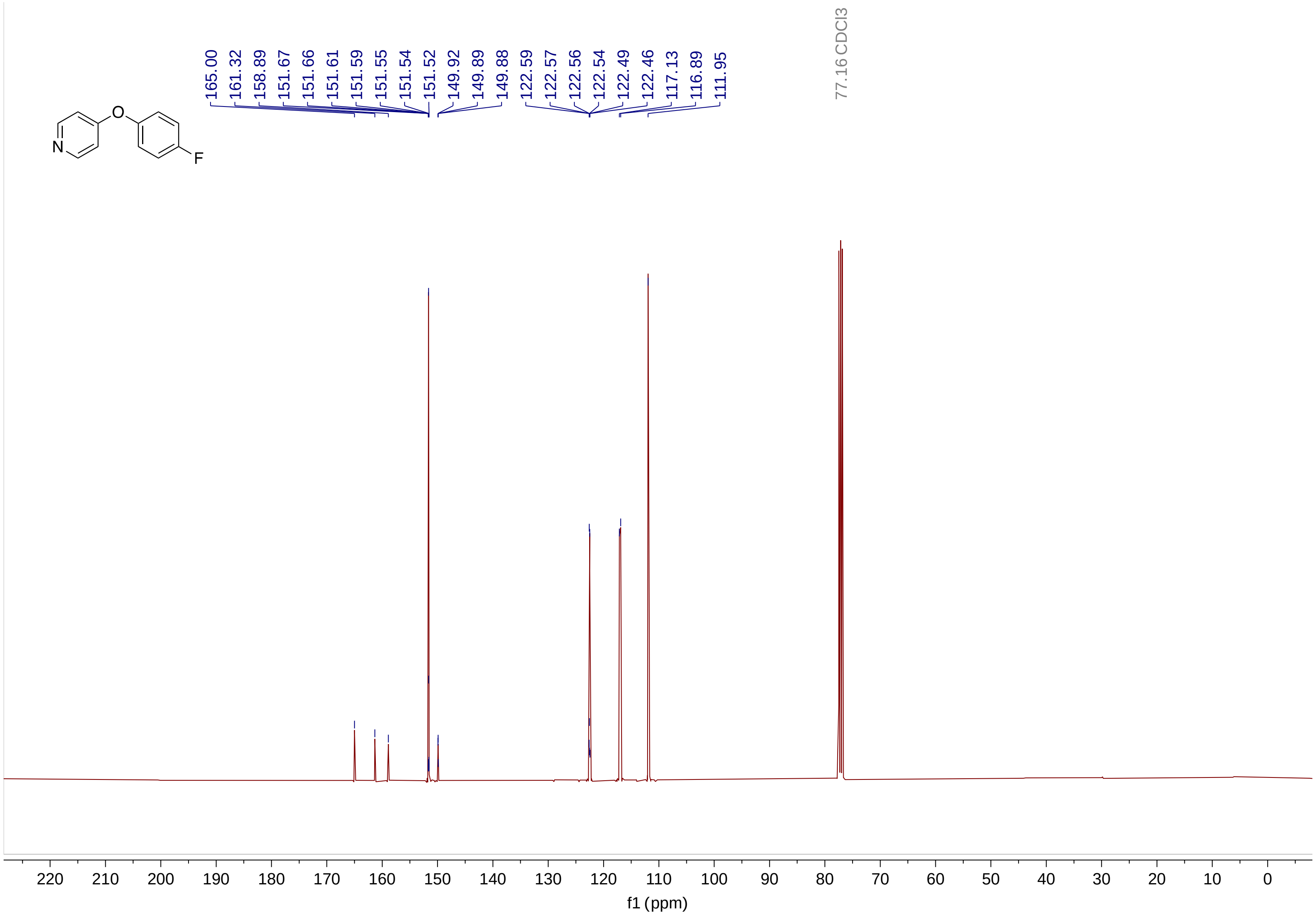

35 - ^{19}F NMR (377 MHz, $CDCl_3$):

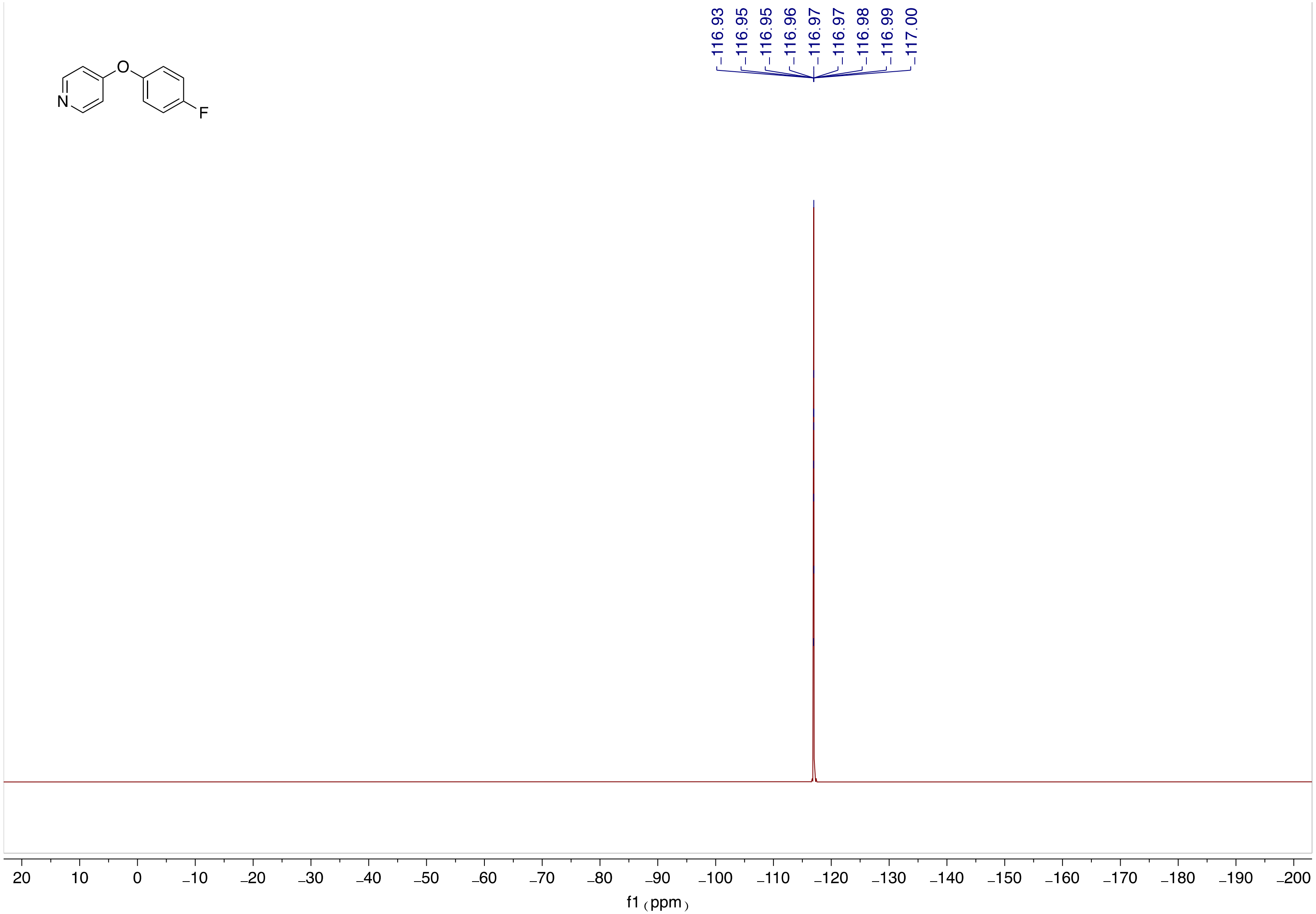

36 - ^{1}H NMR (400 MHz, CDCl₃):

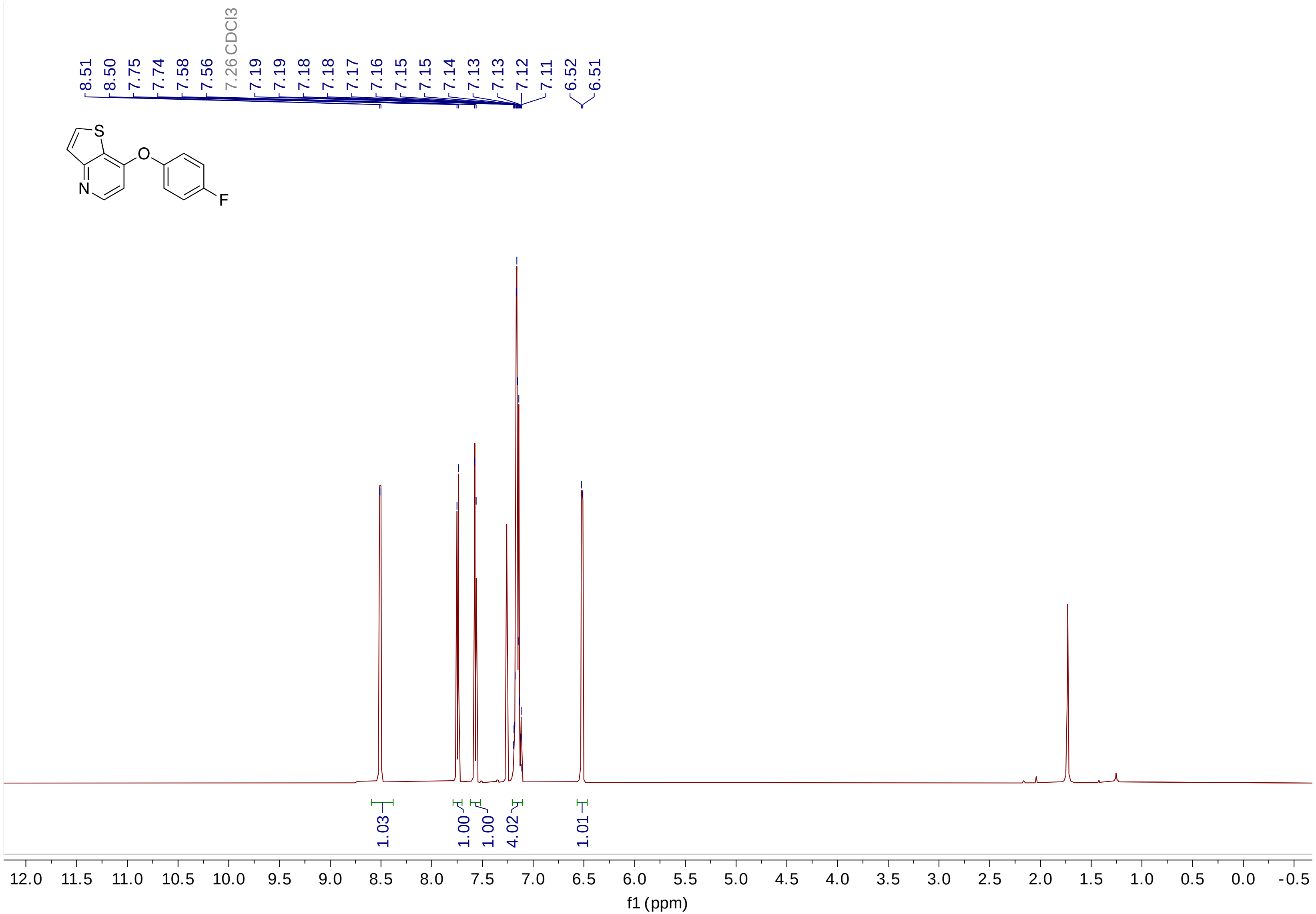

36 - $^{13}C\{^1H\}$ NMR (101 MHz, $CDCl_3$):

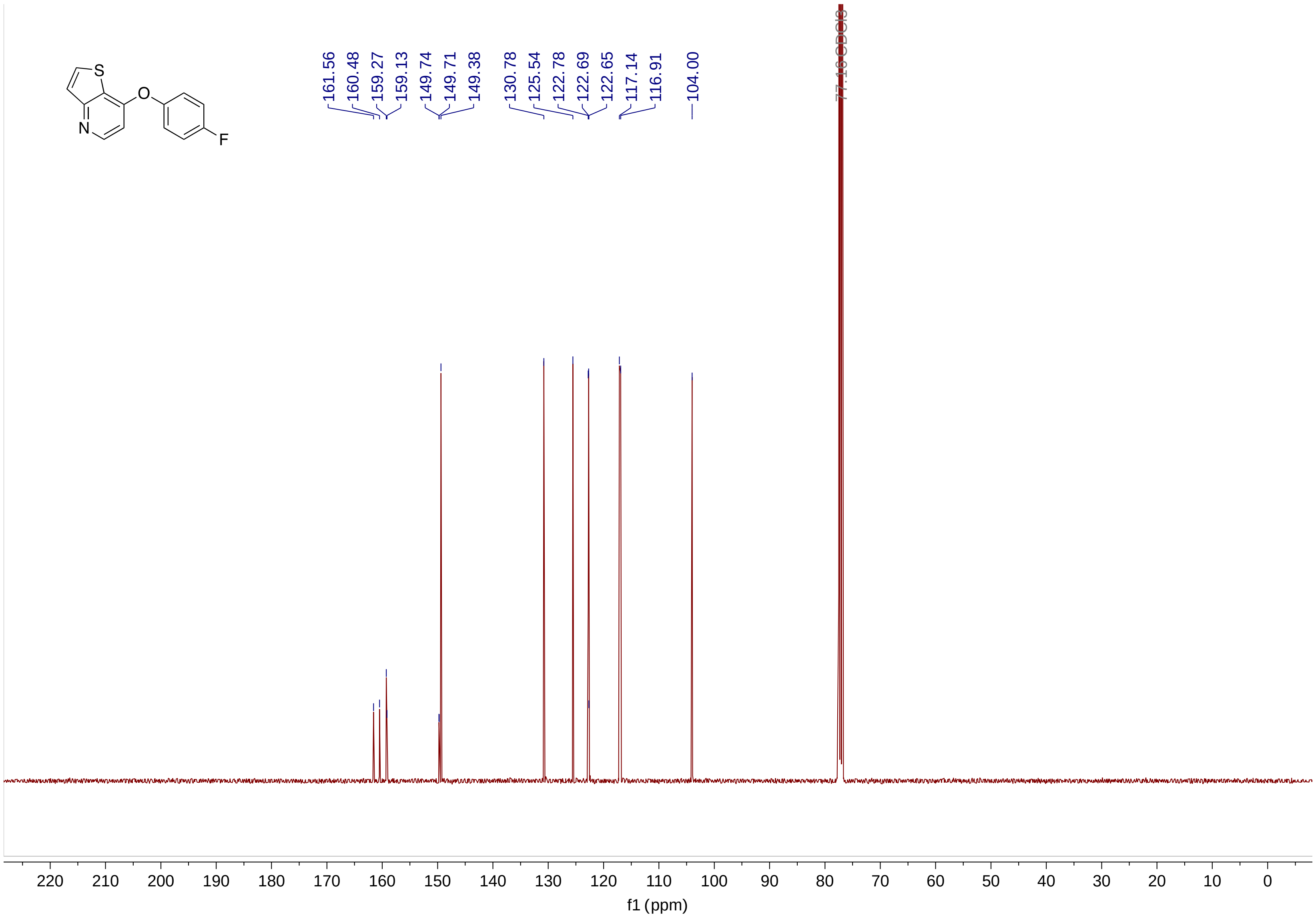

36 - ^{19}F NMR (377 MHz, CDCl$_3$):

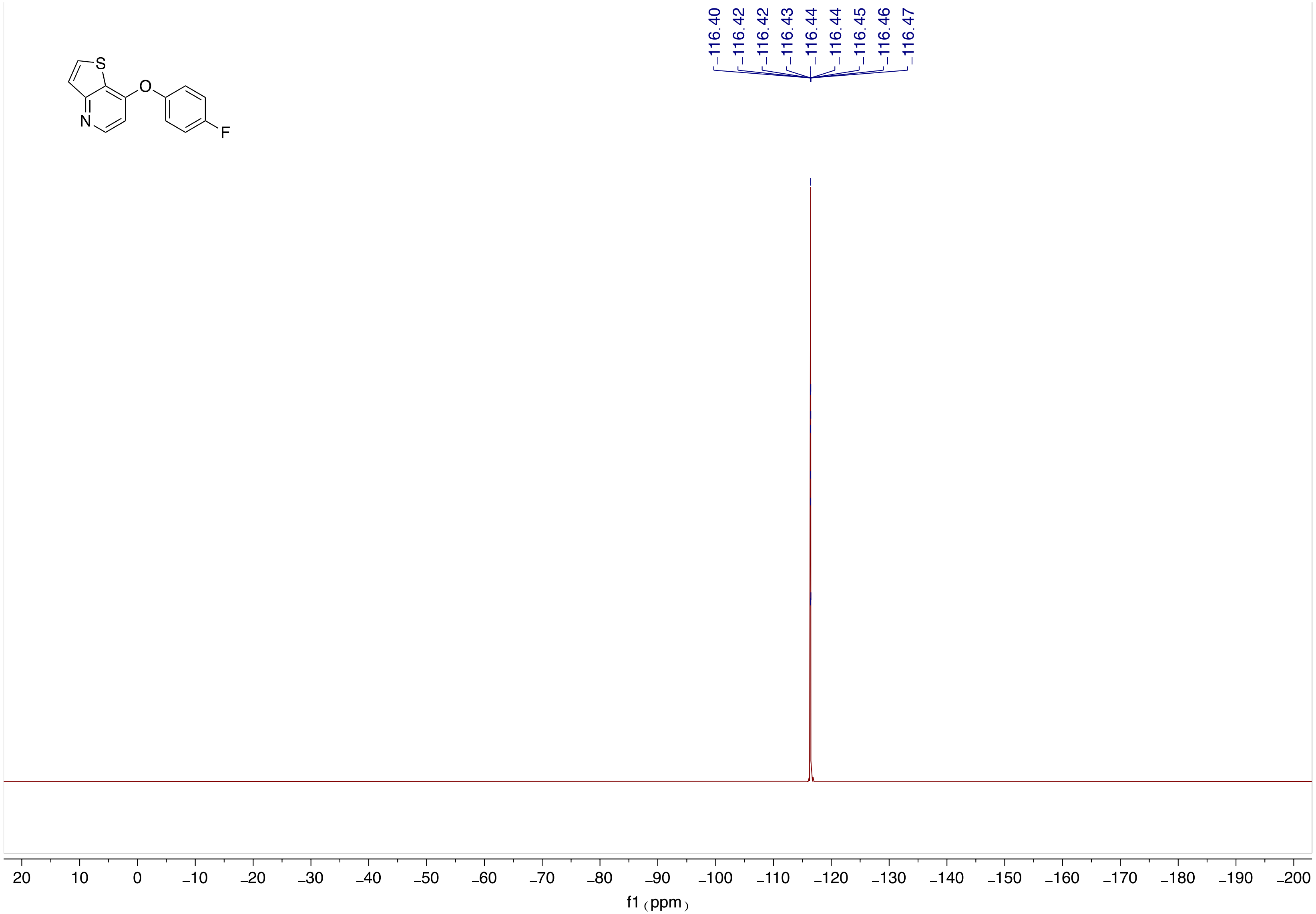

37 - ^{1}H NMR (400 MHz, CDCl$_3$):

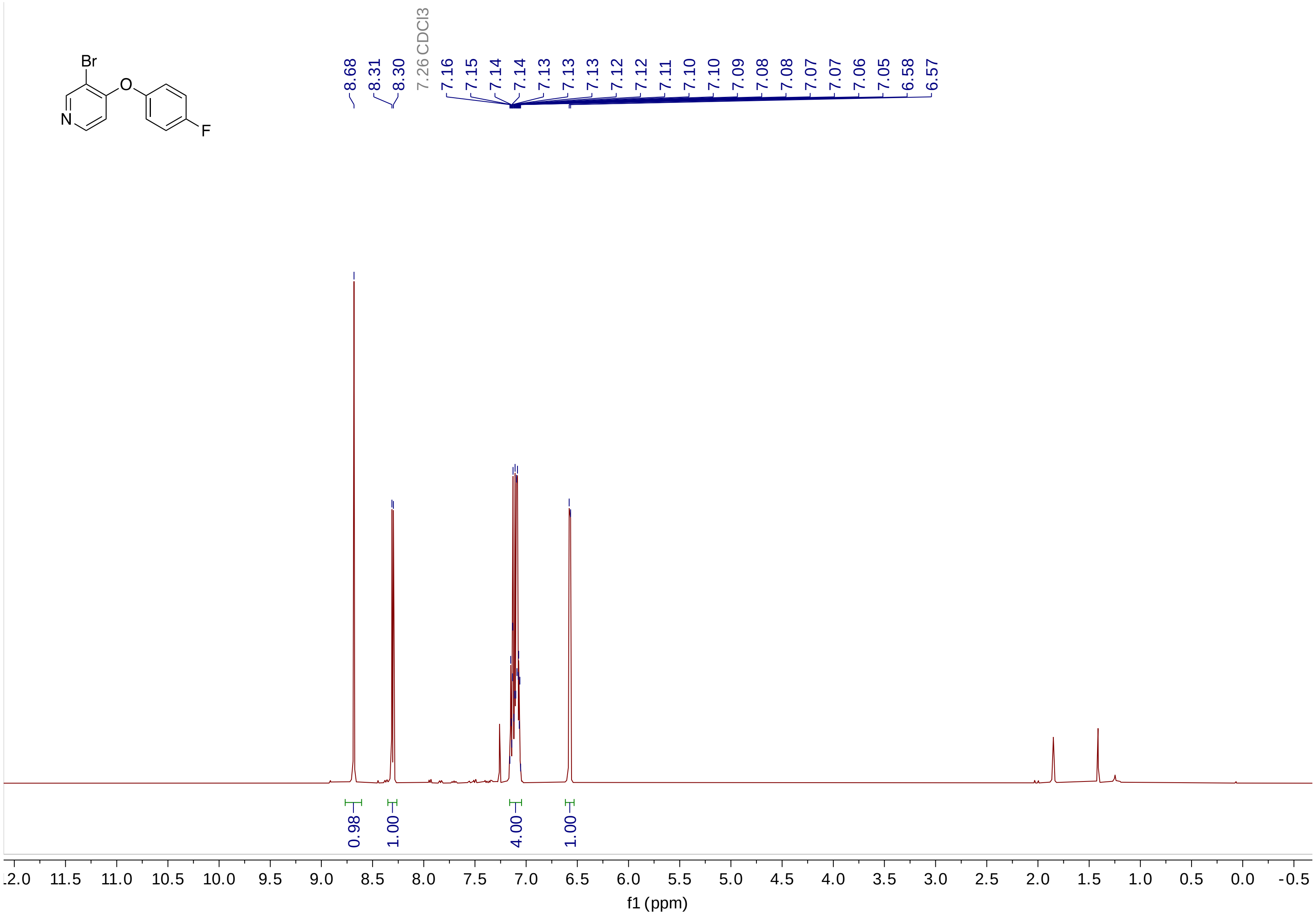

37 - $^{13}C\{^{1}H\}$ NMR (101 MHz, $CDCl_3$):

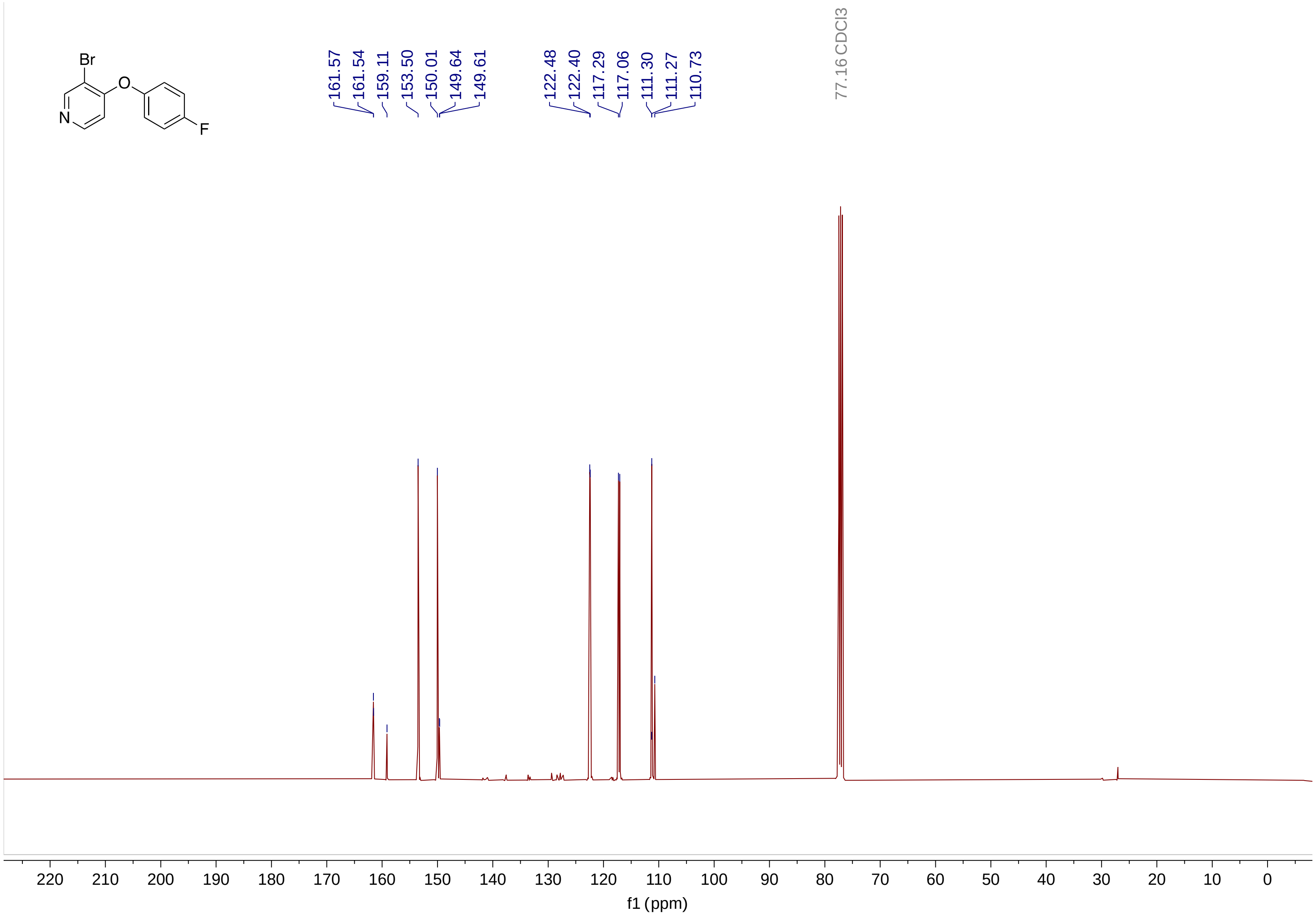

37 - ^{19}F NMR (377 MHz, $CDCl_3$):

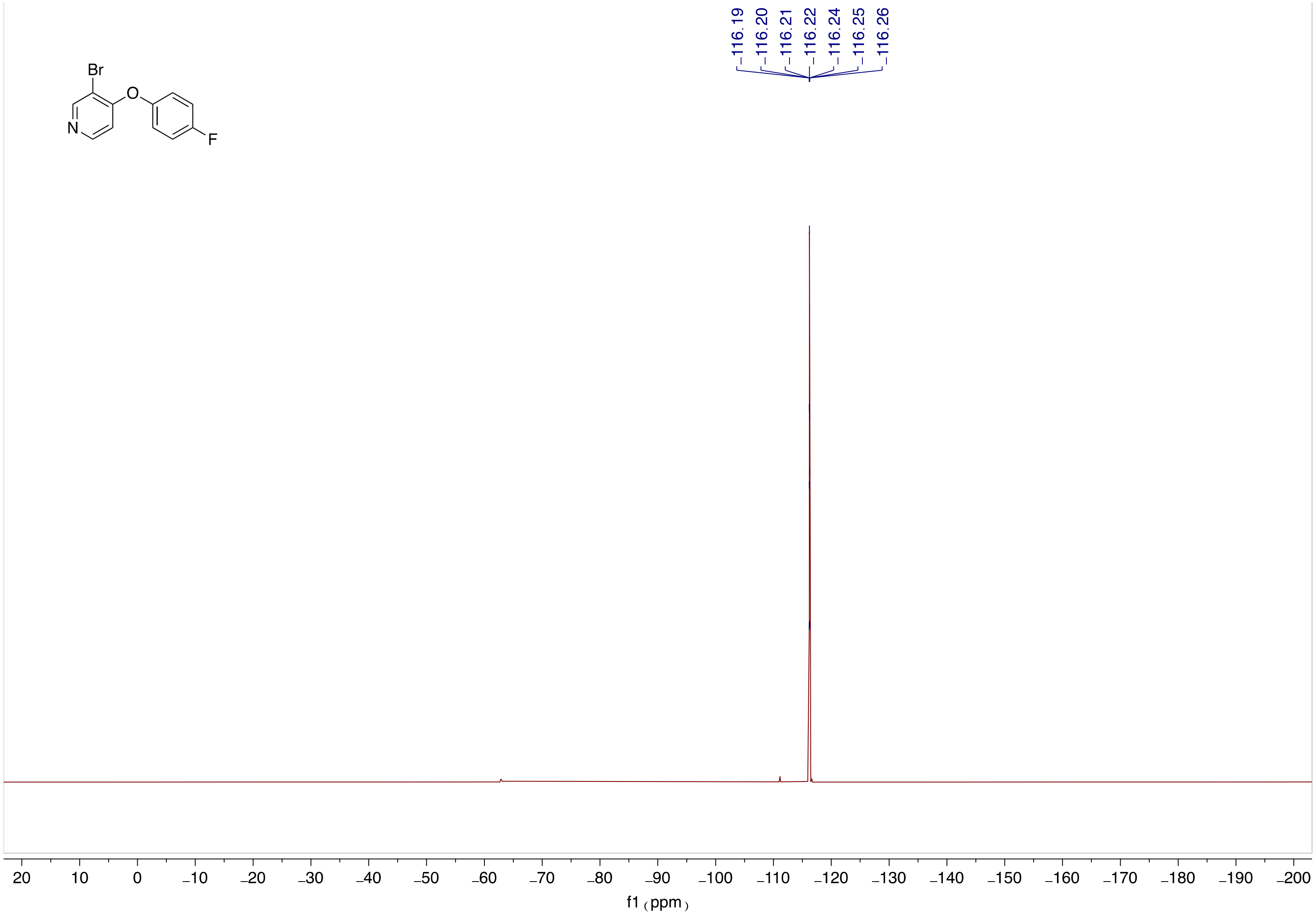

38 - ^{1}H NMR (400 MHz, $CDCl_3$):

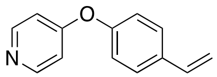

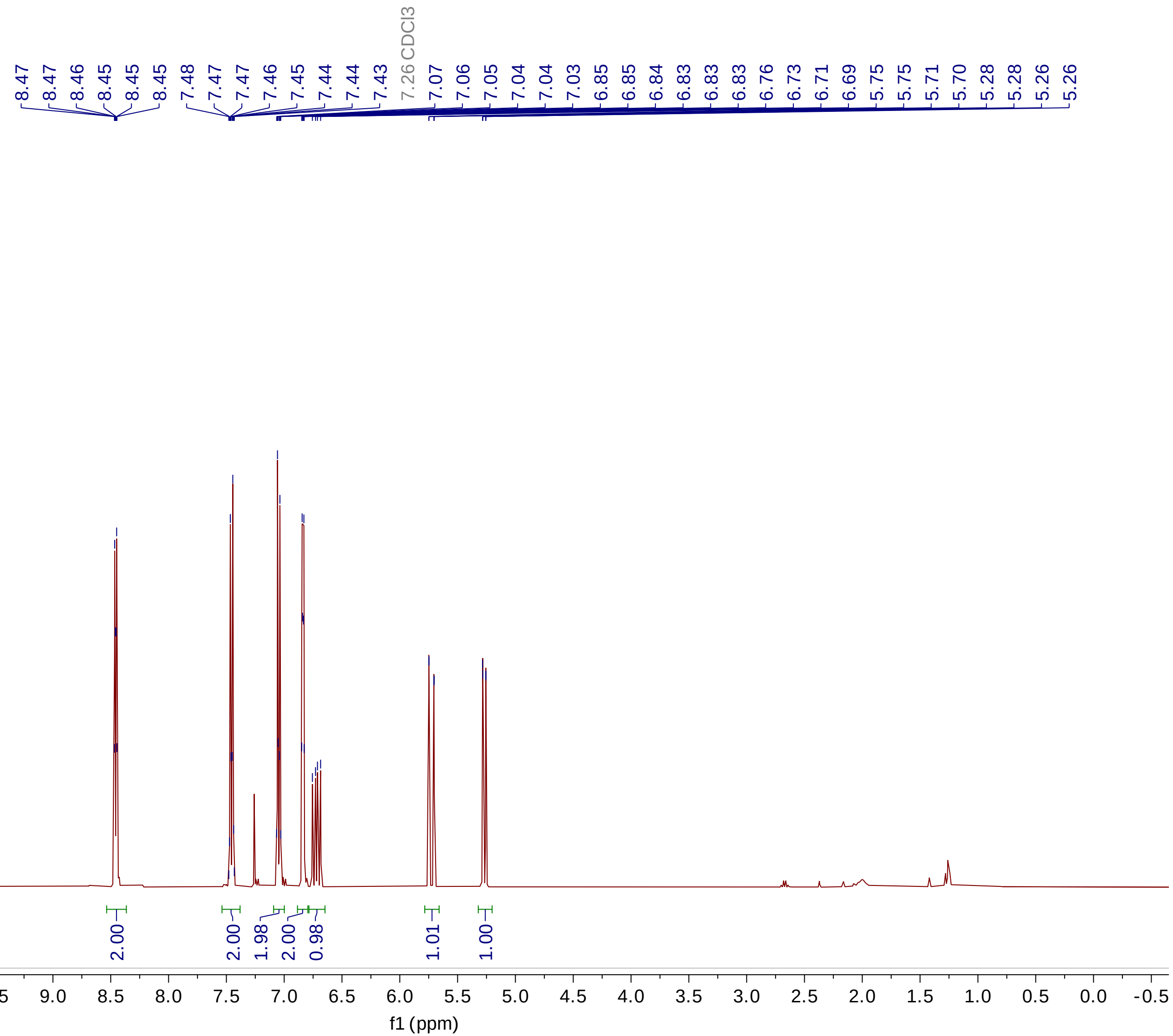

38 - $^{13}C\{^1H\}$ NMR (101 MHz, $CDCl_3$):

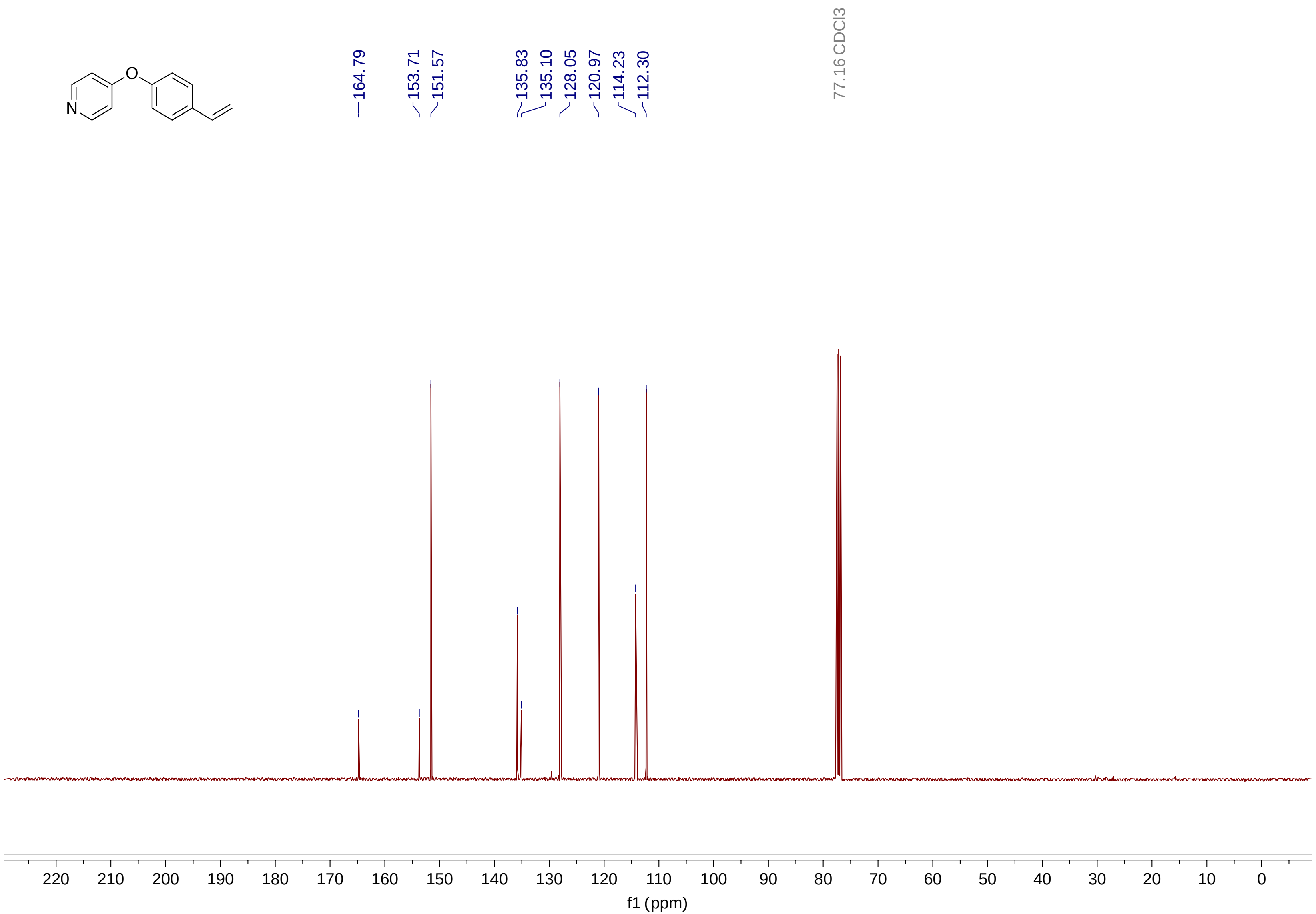

39 - ¹H NMR (400 MHz, $CDCl_3$):

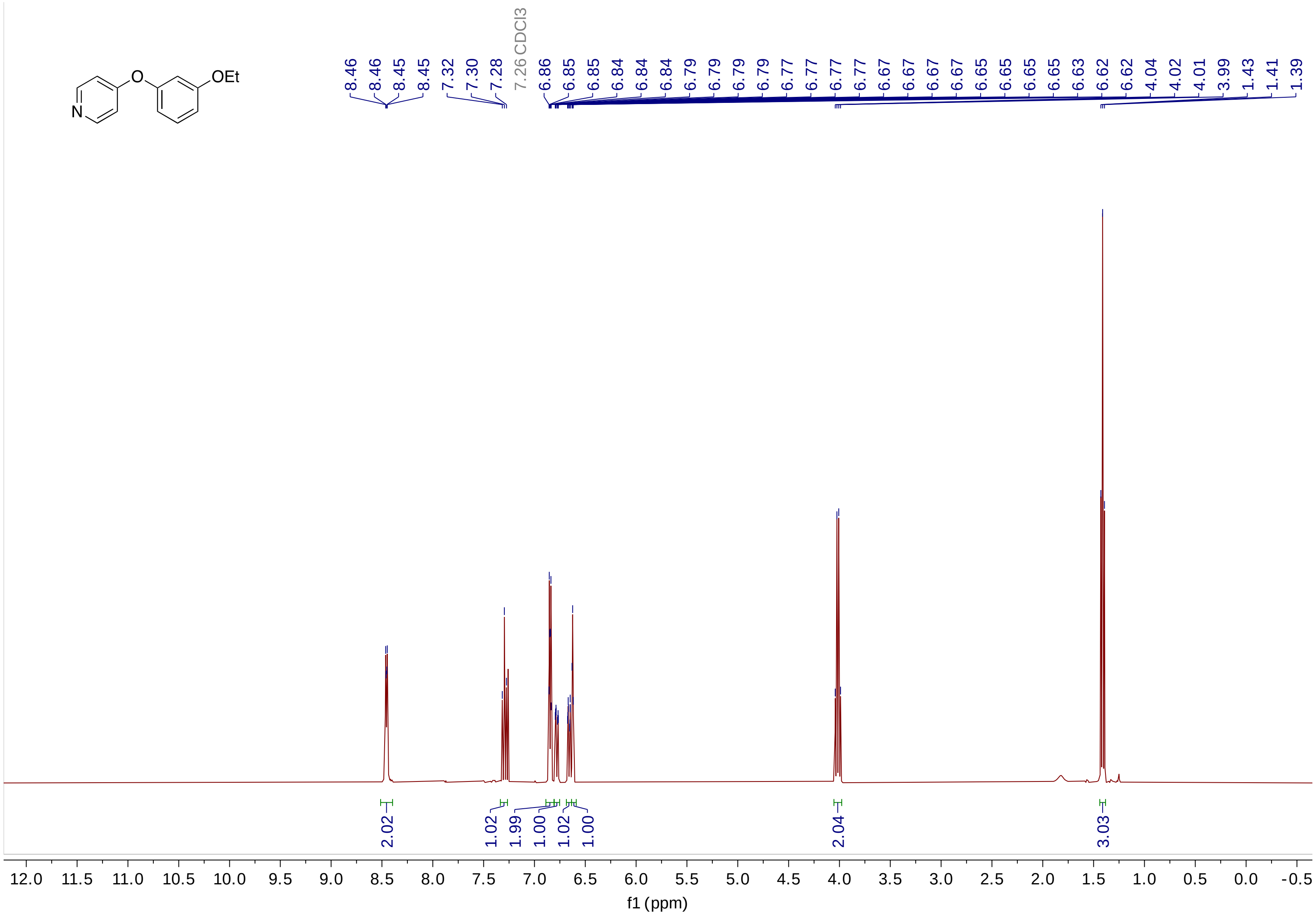

39 - $^{13}C\{^{1}H\}$ NMR (101 MHz, $CDCl_3$):

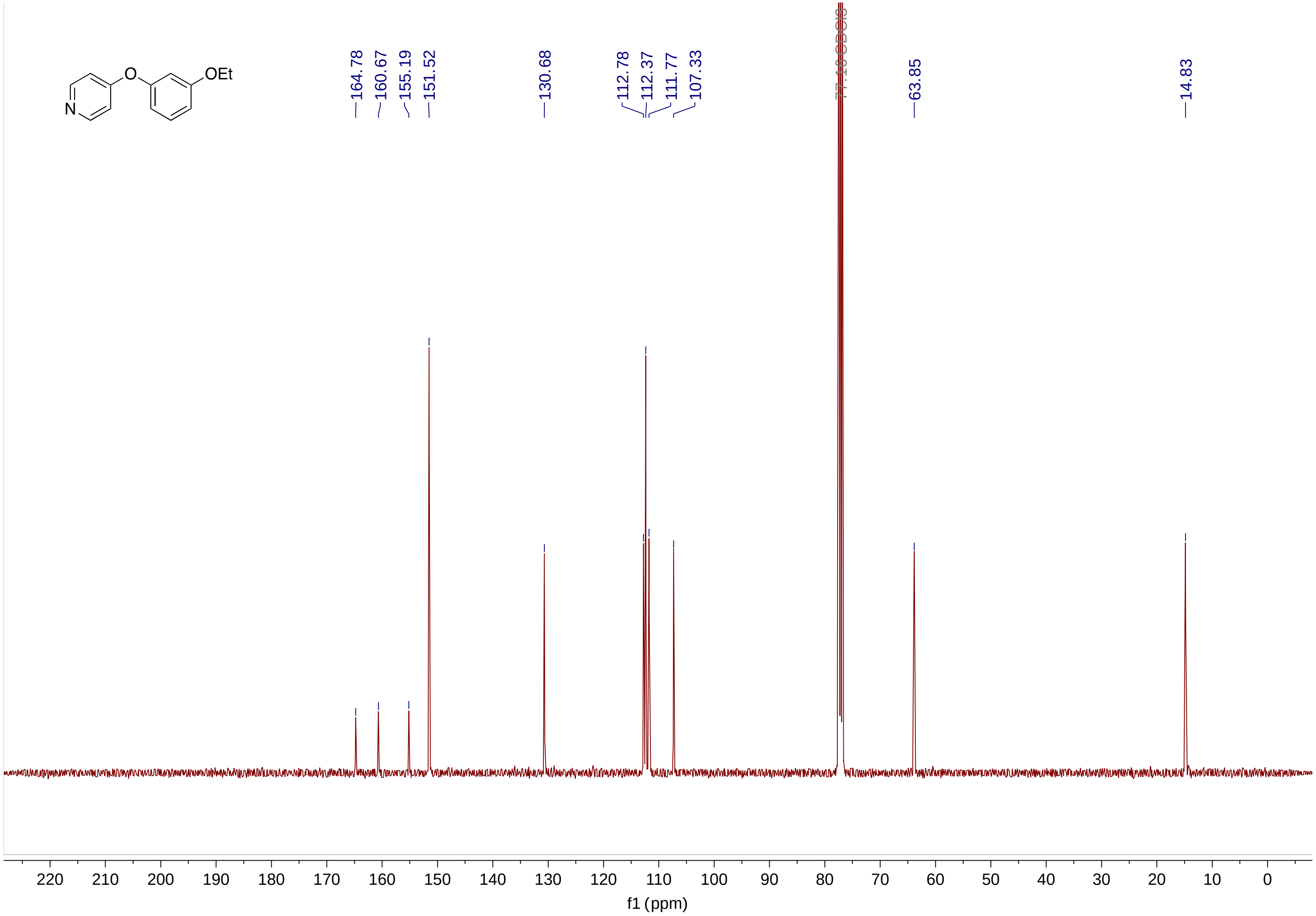

40 - ^{1}H NMR (400 MHz, $CDCl_3$):

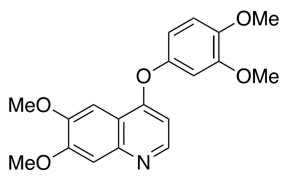

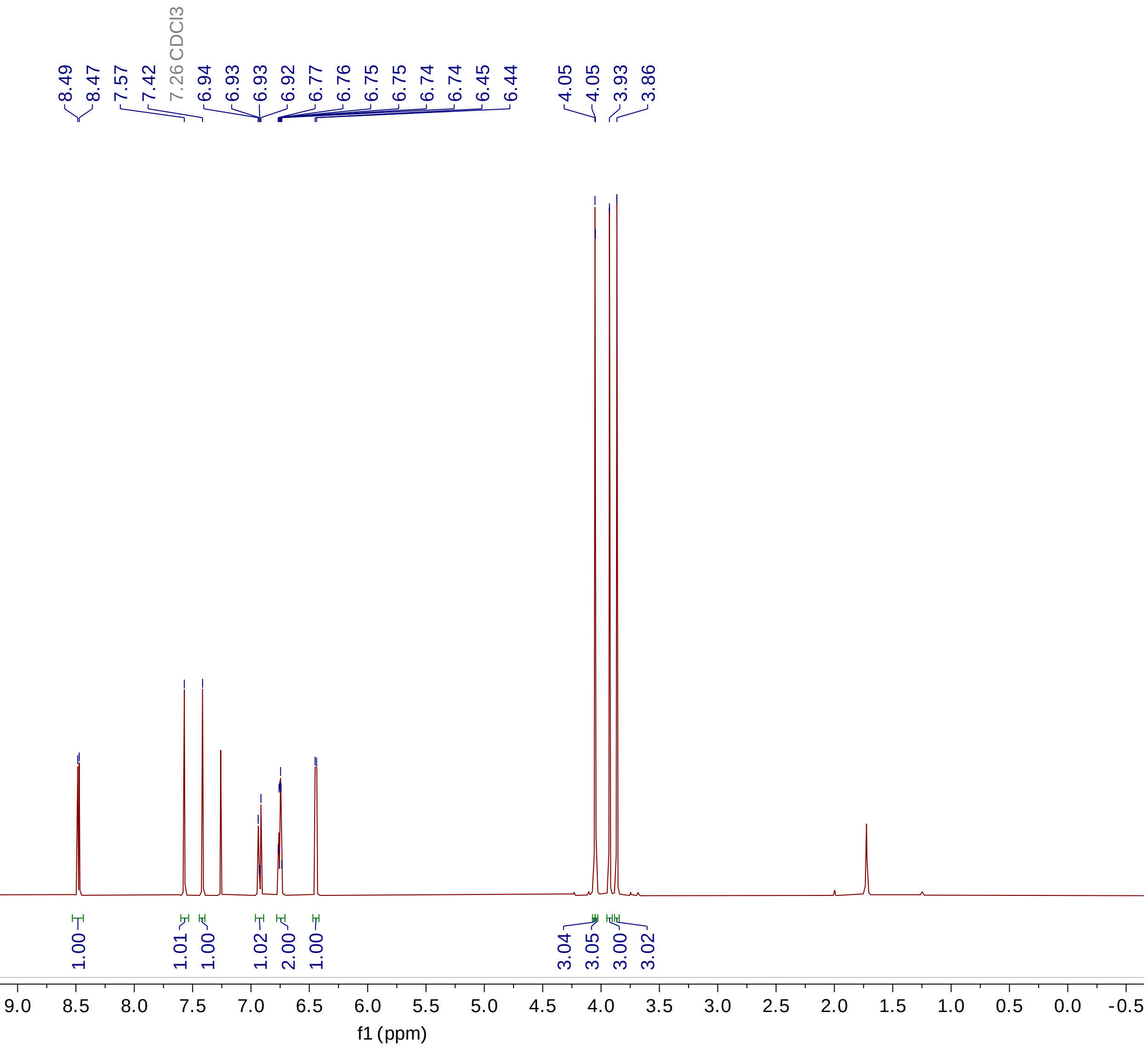

40 - $^{13}C\{^1H\}$ NMR (101 MHz, $CDCl_3$):

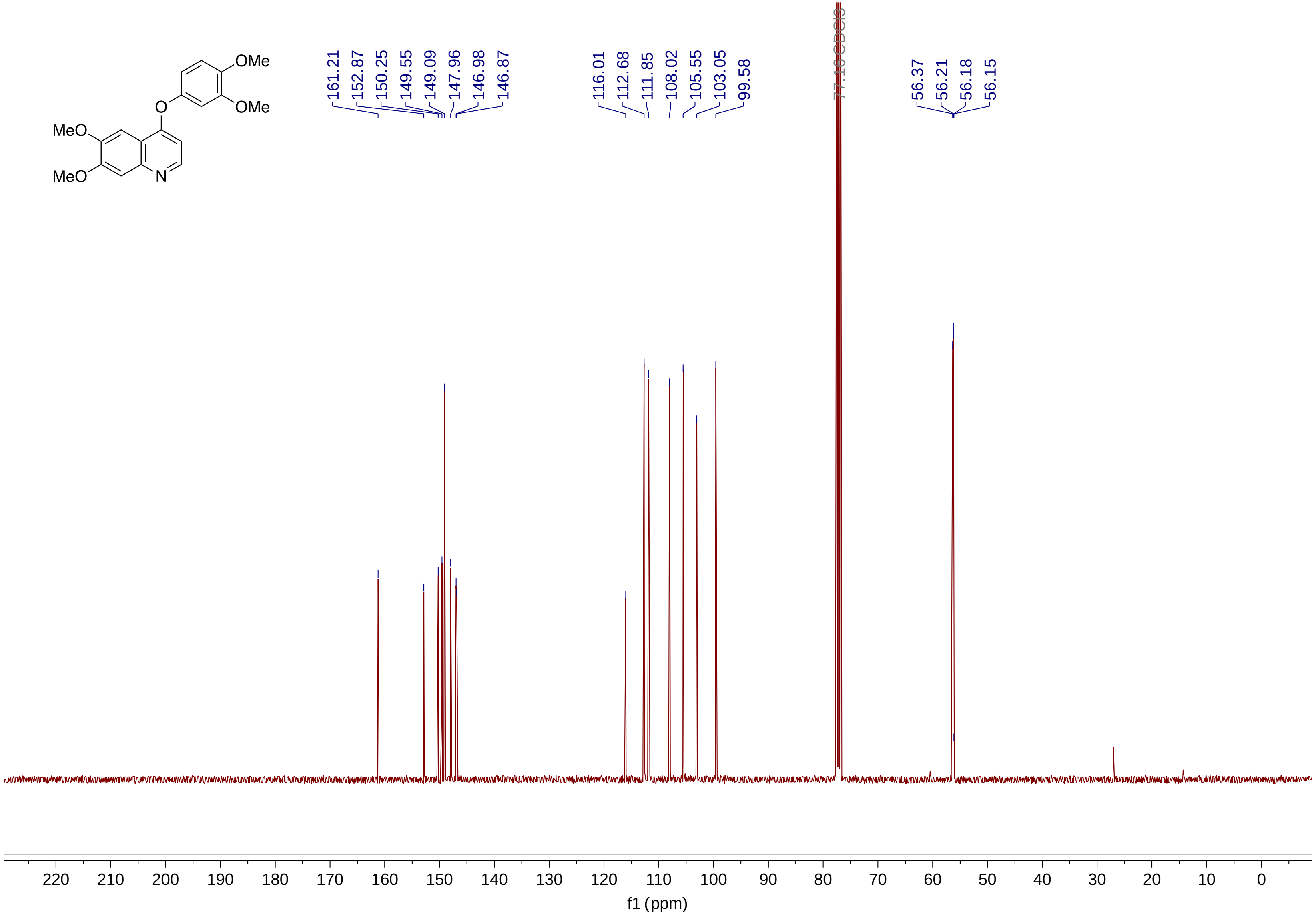

41 - ^{1}H NMR (400 MHz, $CDCl_3$):

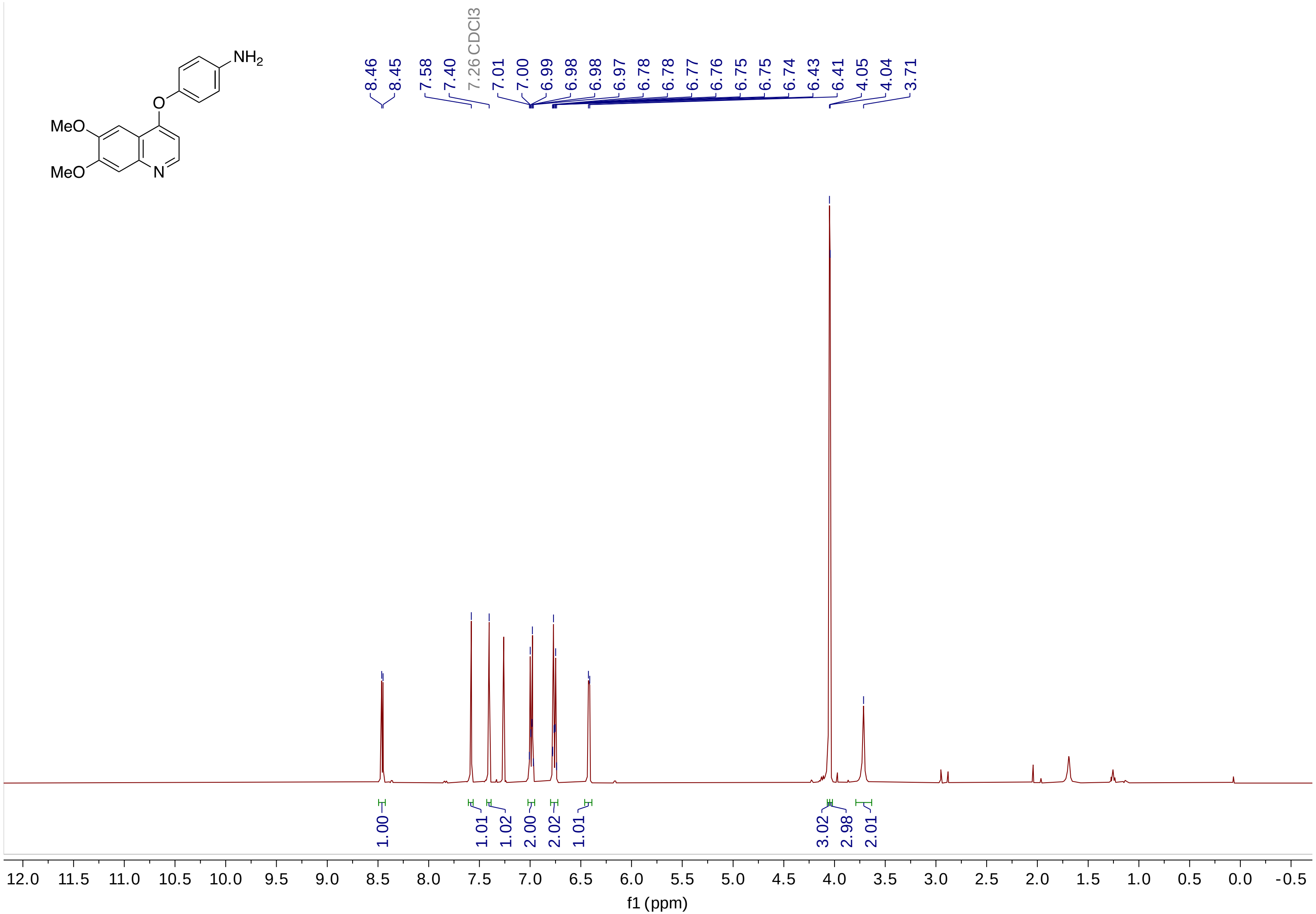

41 - $^{13}C\{^1H\}$ NMR (101 MHz, $CDCl_3$):

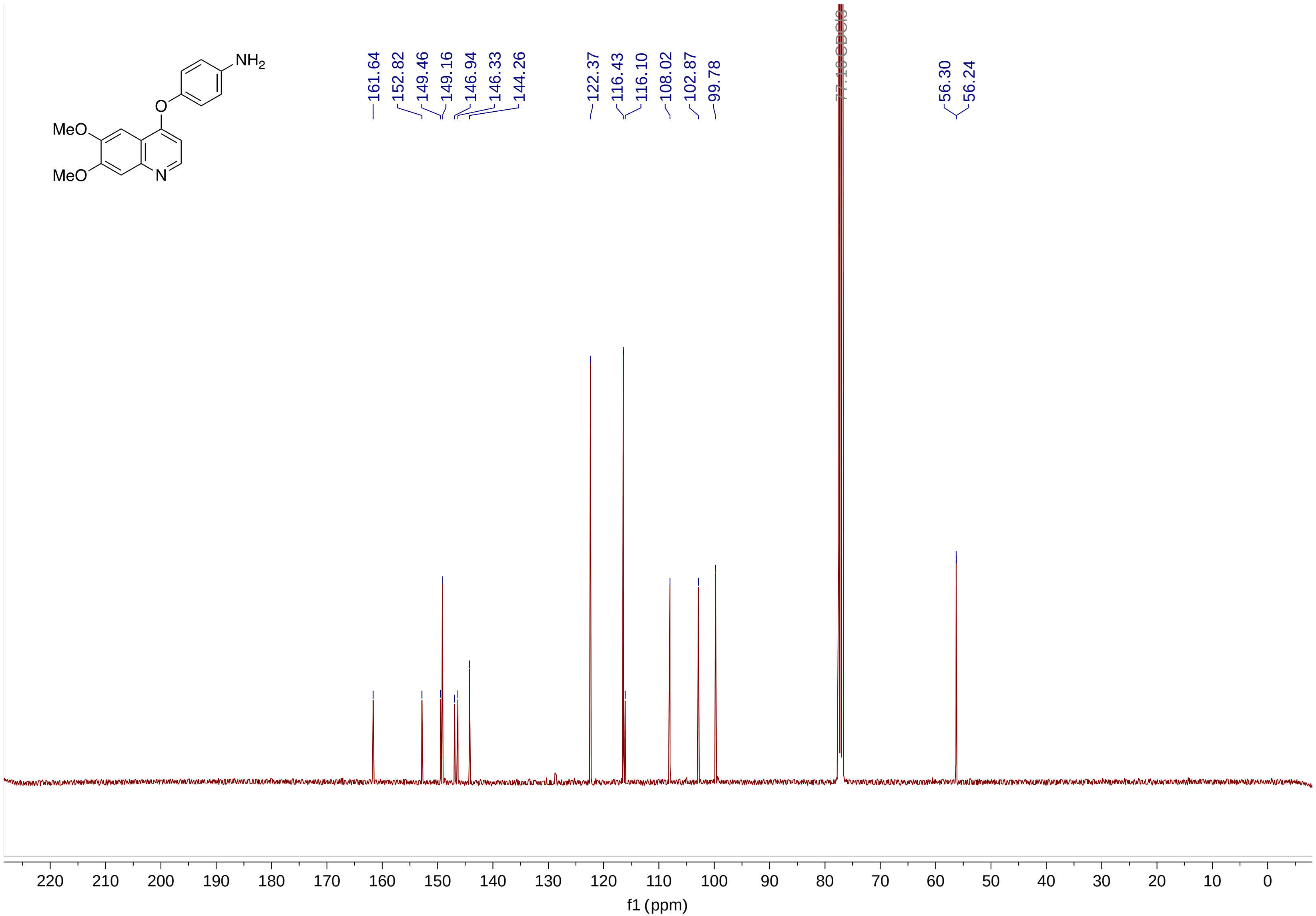

42 - ^{1}H NMR (400 MHz, $CDCl_3$):

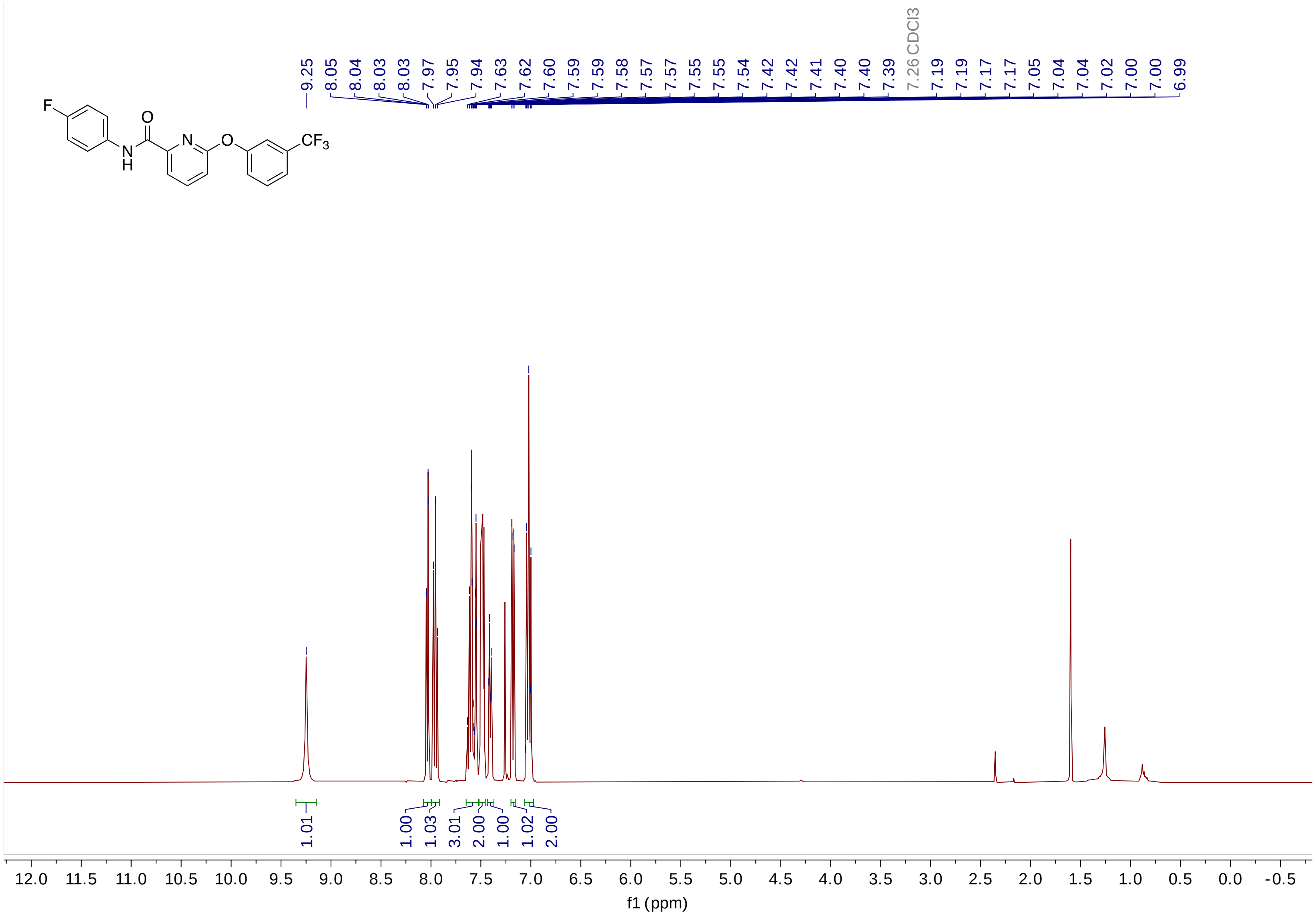

42 - $^{13}C\{^{1}H\}$ NMR (101 MHz, $CDCl_3$):

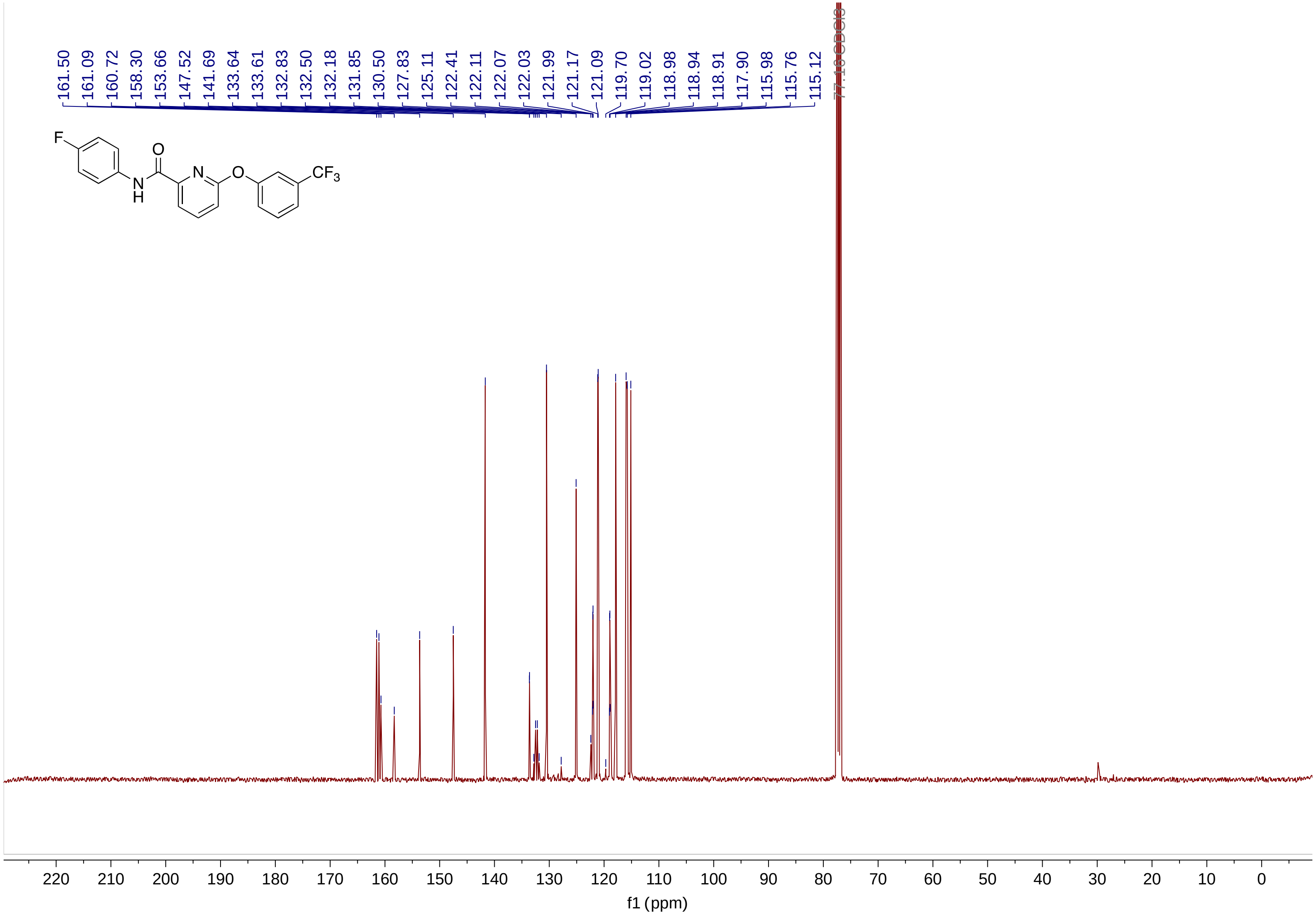

42 - ^{19}F NMR (376 MHz, $CDCl_3$):

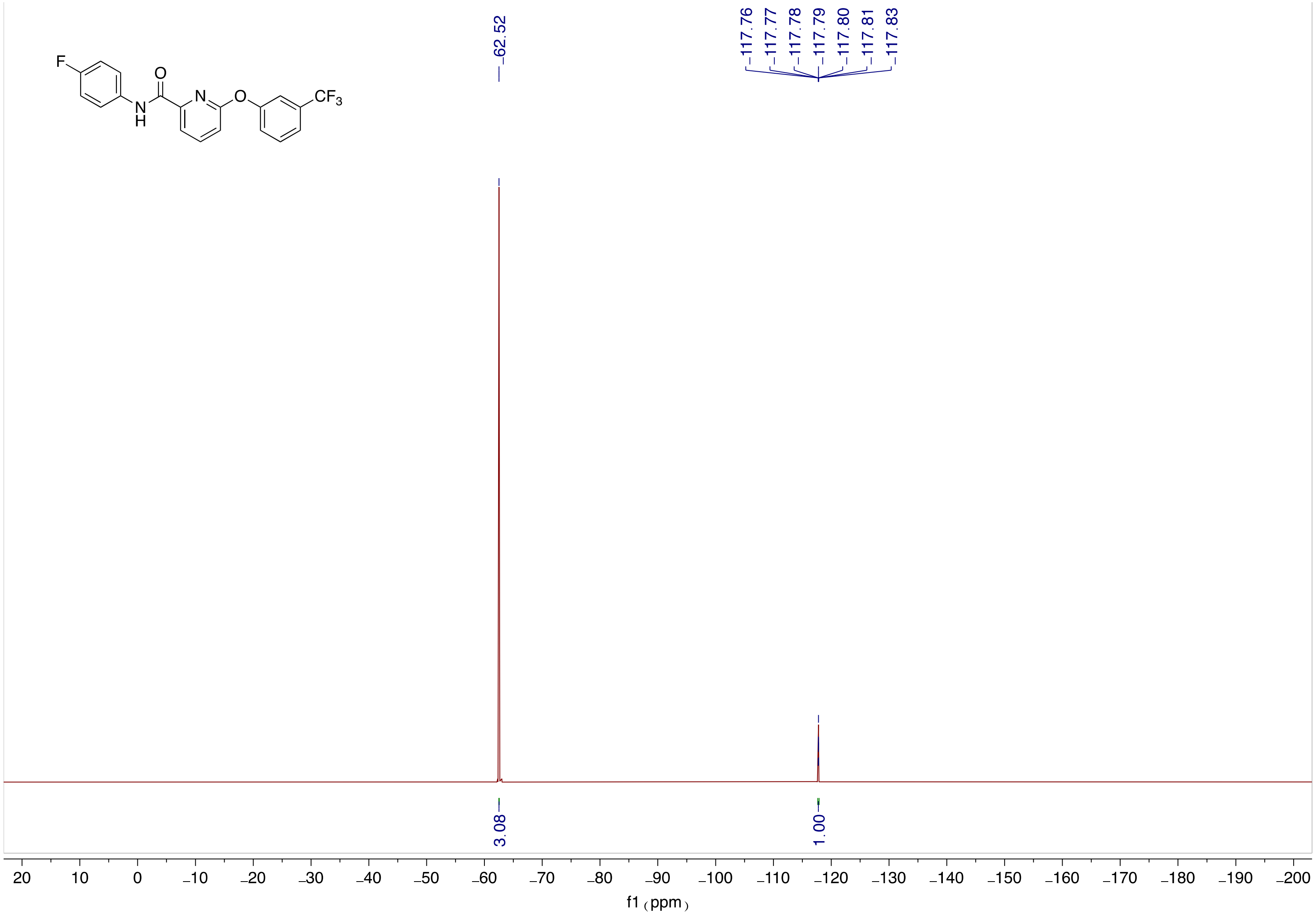

43 - ^{1}H NMR (400 MHz, CDCl$_3$):

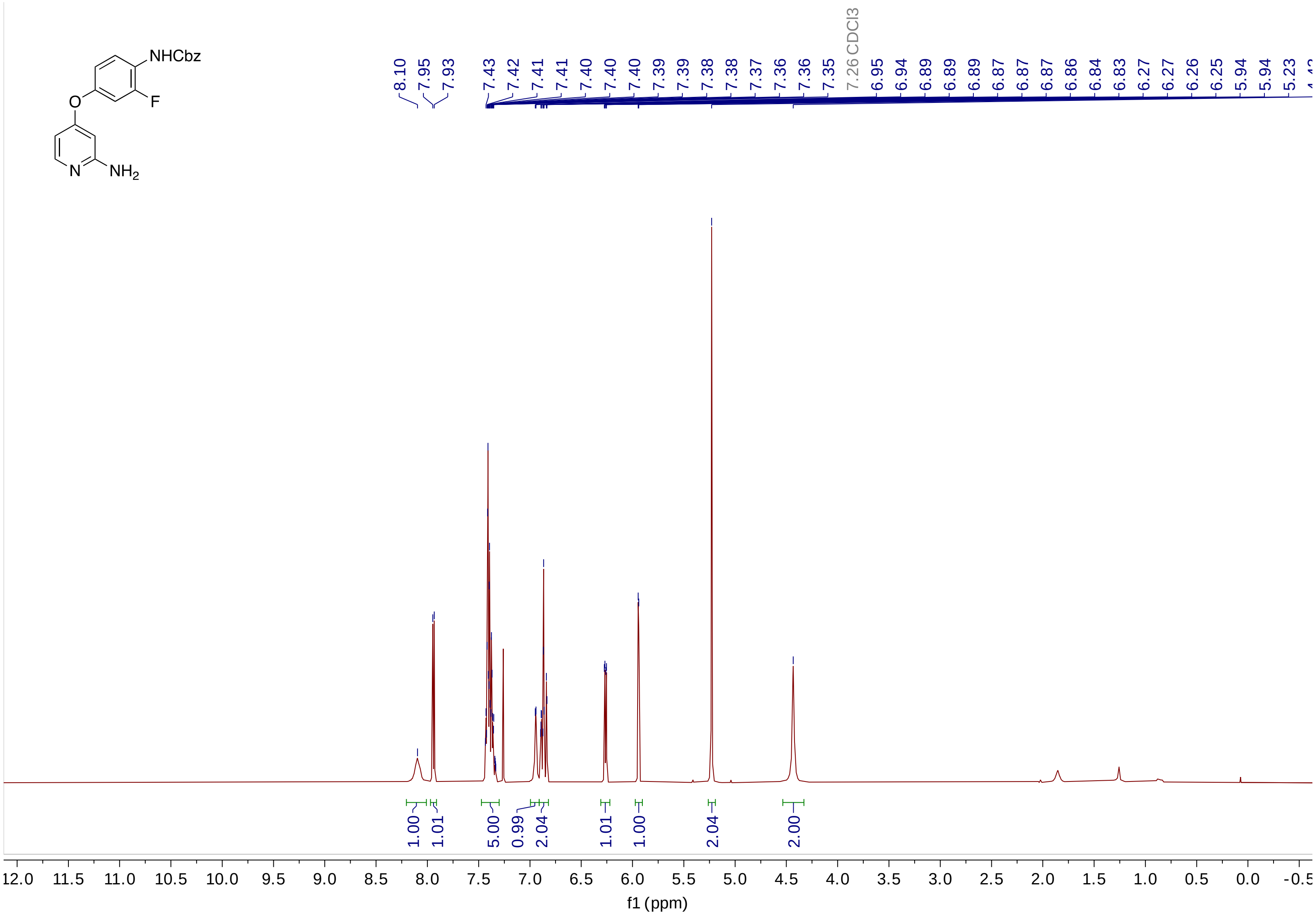

43 - $^{13}C\{^{1}H\}$ NMR (126 MHz, $CDCl_3$):

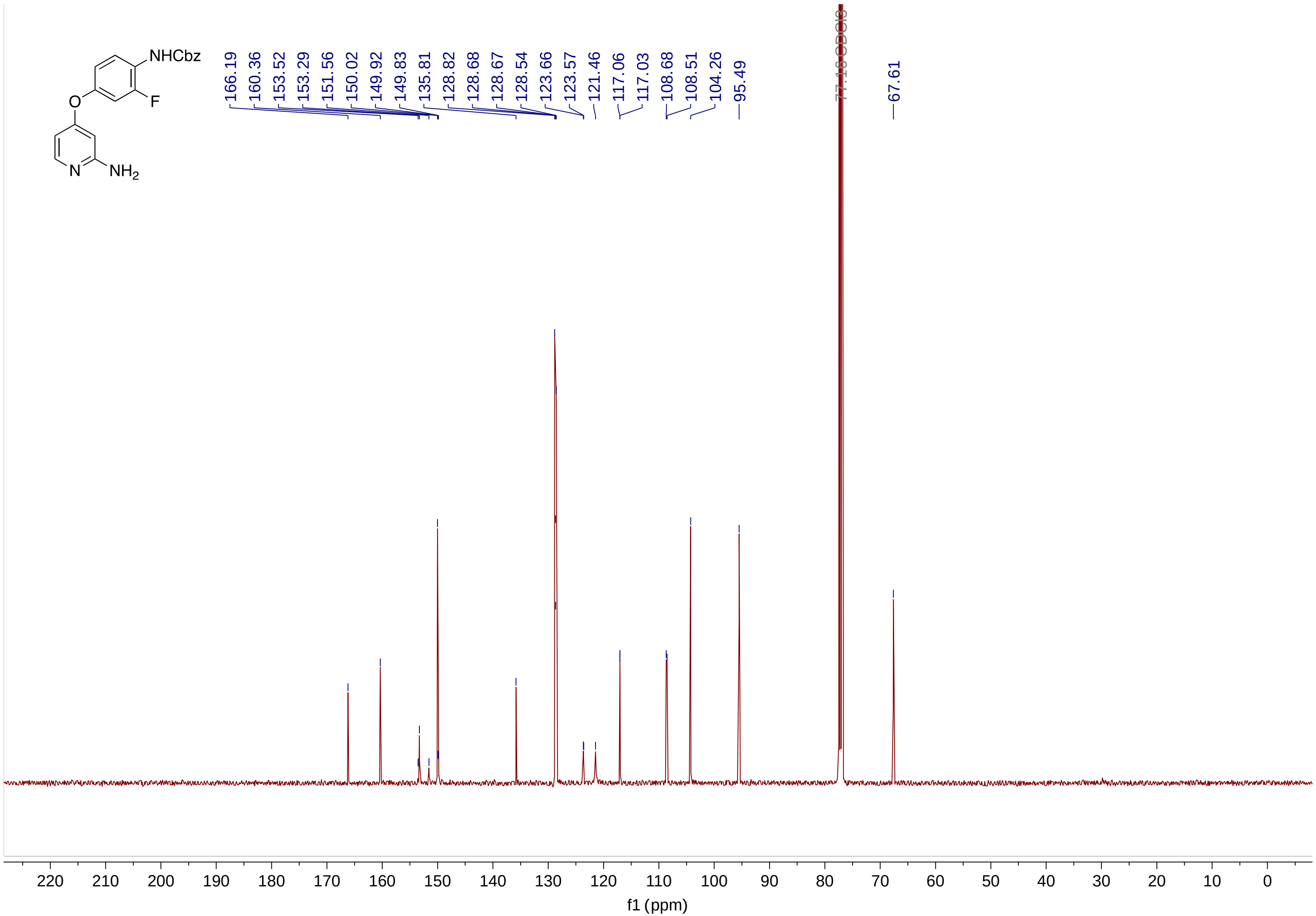

43 - ^{19}F NMR (376 MHz, $CDCl_3$):

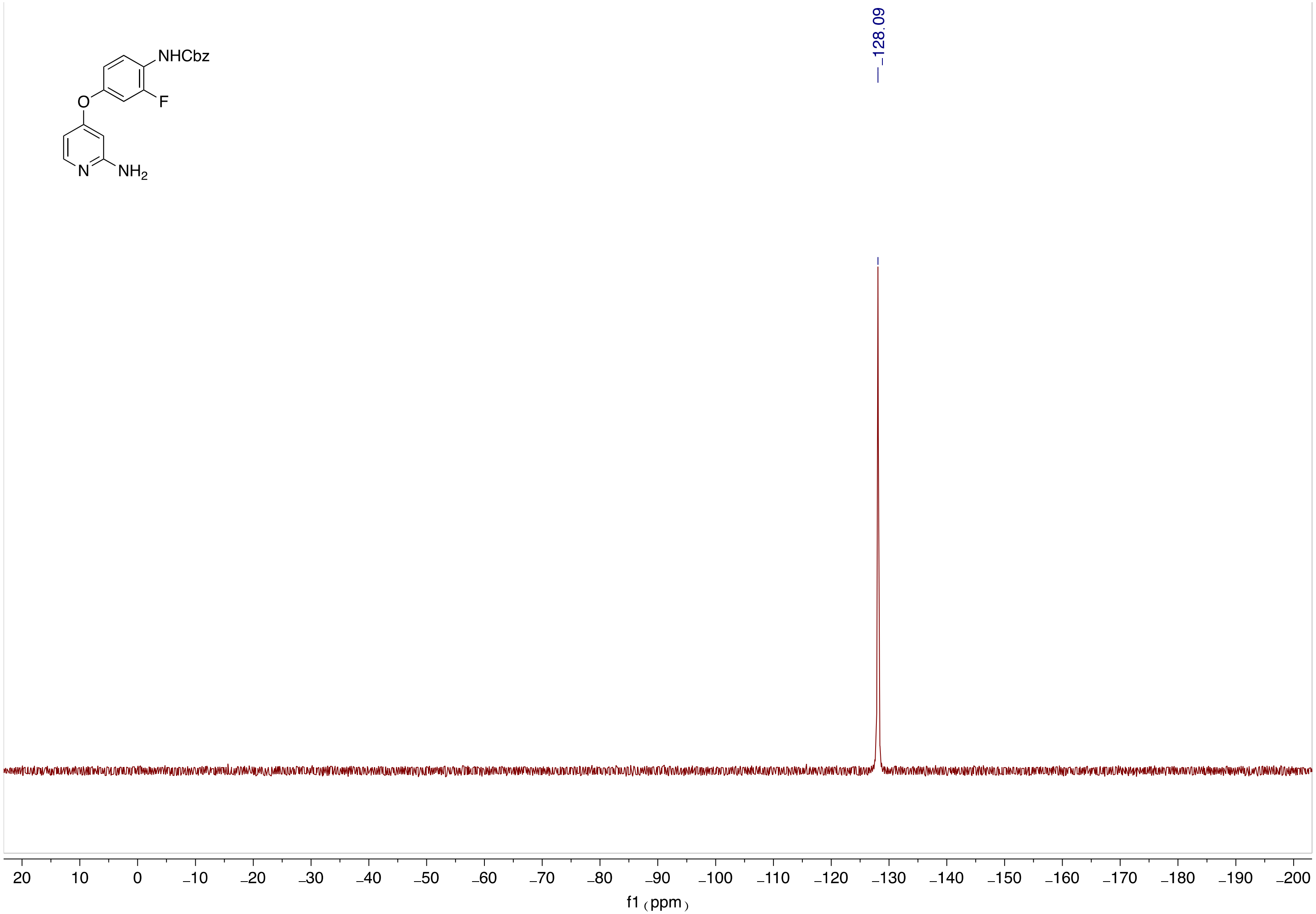

Supplement: Supplementary file 1 — Supporting Information [file ANIE-61-0-s002.pdf]
